# High-risk human papillomavirus cervical infection prevalence: a nationwide retrospective study comparing opportunistic and organised screening, France, 2020 to 2023

**DOI:** 10.2807/1560-7917.ES.2025.30.28.2400689

**Published:** 2025-07-17

**Authors:** Olivier Supplisson, Nicolas Tessandier, Mathilde Roussel, Stéphanie Haim-Boukobza, Sonia Burrel, Mircea T Sofonea, Samuel Alizon

**Affiliations:** 1 Center for Interdisciplinary Research in Biology (CIRB), Collège de France, CNRS, INSERM, Université PSL, Paris, France; 2 Sorbonne University, Paris, France; 3 ExposUM Institute, Université de Montpellier, MIVEGEC, Montpellier, France; 4 Laboratoire Cerba, pole infectiologie, Frépillon, France; 5 Cerba HealthCare, Issy-les-Moulineaux, France; 6 CHU de Bordeaux, Service de virologie, Bordeaux, France; 7 CNRS UMR 5234, Fundamental Microbiology and Pathogenicity, Université de Bordeaux, Bordeaux, France; 8 PCCEI, Université de Montpellier, INSERM, Montpellier, France; 9 Department of Anesthesiology, Critical Care, Intensive Care, Pain and Emergency Medicine, CHU Nîmes, Nîmes, France

**Keywords:** high-risk HPV, cervical infection prevalence, organised screening, opportunistic screening, Gaussian random fields, Gaussian Markov random fields

## Abstract

**BACKGROUND:**

In France, cervical cancer screening for females aged 30­–65 years primarily tests for high-risk (HR) human papillomavirus (HPV) infections.

**AIM:**

We aimed to map the prevalence of cervical infections caused by HPV16 and/or 18, or by any of 12 other carcinogenic HPV genotypes and compare prevalence estimates from tests from spontaneous medical visits (opportunistic screening) or the national screening programme (organised screening).

**METHODS:**

We extracted data from a large network of biology laboratories, containing all available results from HR HPV tests performed between 1 January 2020 and 30 November 2023 in metropolitan France. A full hierarchical Bayesian model was used to compute spatially resolved expected prevalence maps at the postcode level.

**RESULTS:**

The analytic sample contained results of 362,963 HR HPV tests. Among samples positive for HPV16 and/or 18, 2.9% and 3.8% were from organised and opportunistic screening, respectively. Samples positive for other genotypes were 6.9% and 9.4%, respectively.

During the last week of the study (week 48 2023), among females aged 30 years, opportunistic screening was associated with a greater expected prevalence of HPV16 and/or 18 and other genotypes in 97.2% and 99.9% of postcodes, respectively. The probability this percentage was lower among females aged 66 years was below 95% for both genotype groups.

For organised screening, a pronounced north-west/south-east gradient in infection prevalence was found across France for both genotype groups, with hotspots located at the border with Italy, Spain and Switzerland.

**CONCLUSION:**

Opportunistic screening is associated with systematic inflation of HR HPV infection prevalence.

**Key public health message**

**What did you want to address in this study and why?**

Most cervical cancers are caused by persistent infections by some type of human papillomavirus (HPV). We estimated the variation of the proportion of females infected by HPV16 and/or HPV18, or at least one of 12 other carcinogenic HPV types, across metropolitan France using tests from spontaneous medical visits or from the national screening programme. Better knowledge of HPV prevalence from screening type could help target intervention in hotspot areas.

**What have we learnt from this study?**

Among the 362,963 HPV tests analysed, 3.7% were positive for HPV16 and/or 18 and 9.2% were positive for at least one of the 12 other types of HPV. Tests following a spontaneous visit to a primary care provider were associated with a greater proportion of females infected than those collected through the national organised screening programme. Infection rates of high-risk HPV types in females varied geographically, decreasing from north-west to south-east.

**What are the implications of your findings for public health?**

Screening guidelines for HPV are standard for all of France. The identification of areas with a high proportion of females infected with cancerogenic HPV can help adjust screening efforts to better match local needs.

## Introduction

Human papillomaviruses (HPV) cause a massive public health burden worldwide. In addition to anogenital warts and papillomatosis, persistent HPV infections caused by oncogenic (high-risk (HR)) genotypes are responsible for nearly all cervical cancers and a substantial proportion of penile, vulval, vaginal, anal and oropharyngeal cancers [1]. In 2020, an estimated 600,000 new cases of cervical cancer were reported globally, making it the fourth most common cancer among females [2,3]. In the European Union, cervical cancer ranks as the second most prevalent cancer among females aged 15–44 years, after breast cancer [4]. The World Health Organization (WHO)’s global strategy to accelerate the elimination of cervical cancer as a public health problem promotes HR HPV screening as one of the key interventions to prevent and control the burden associated with this disease [5].

In 2019, France updated its cervical cancer screening guidelines, shifting from cytology-based screening to a combination of cytological analysis and HPV DNA detection testing (hereafter referred to as HR HPV test). These updated guidelines keep cytological analysis as the primary screening tool for females aged under 30 years, but now recommend HR HPV testing as the primary screening method for females aged 30 to 65 years. The first HR HPV test should be performed as early as 30-years-old, or 3 years after the last cytological analysis. If this test is negative for all HR HPV, the next test should be performed 5 years later. In the case of a positive result for one of the targeted HPV genotypes, a cytological analysis should be performed within the year. If the cytological analysis is normal, a new HR HPV test should be performed 1 year later [6,7].

The national cervical cancer screening programme was updated in July 2020 with these new guidelines. This programme, which was launched in 2018 following encouraging results from modelling and pilot studies [8,9], aims to reduce the incidence of cervical cancer and associated mortality by 30% over 10 years through an increase in screening coverage rate of up to 80% [6,7,10]. It was partly motivated by concerns about the observed decreasing trend in ‘opportunistic screening’ uptake, i.e. prescription-based screening following spontaneous medical consultations [11]. The new version of the French organised screening programme targets females aged 30 to 65 years who do not follow French screening guidelines by inviting them to consult a primary care provider and undergo a free HR HPV test (see Supplement S1 for additional details).

As of 2025, many countries have implemented similar organised cervical cancer screening programmes [12,13]. As in France, these programmes typically rely on a combination of cytological analyses and HR HPV tests. Beyond their contribution to reducing the incidence and mortality of cervical cancer [14-16], screening programmes relying on HR HPV tests offer a unique window on HR HPV cervical infection prevalence. Furthermore, due to different selection mechanisms in screening uptake, prevalence estimates obtained through organised screening programmes are expected to differ from those derived from opportunistic screening [17,18].

This study aims to provide the first spatially-resolved picture of cervical infections caused by HPV16 and/or HPV18, or caused by at least one of 12 other carcinogenic HPV genotypes (31, 33, 35, 39, 45, 51, 52, 56, 58, 59, 66, and 68), according to the WHO classification [19], in metropolitan France. It also aims to study whether opportunistic and organised screening are associated with systematic differences in prevalence estimates.

## Methods

### Data

The initial extraction of data contained all available results from HR HPV tests performed on cervical samples between 1 January 2020 and 30 November 2023 by Cerba (Cerba Laboratory, Frépillon, France), one of the largest networks of medical biology laboratories in the country.

Regardless of the screening pathway, two types of laboratory-validated assays were used to analyse collected samples: either the Alinity m high-risk assay (Abbot Molecular, Abbot Park, United States) or the Roche Cobas HR HPV test (Roche Diagnostics, Rotkreutz, Switzerland) [20]. Both assays target the viral DNA of 14 HPV genotypes: 16, 18, 31, 33, 35, 39, 45, 51, 52, 56, 58, 59, 66, and 68. Alinity m high-risk can distinguish between HPV16, HPV18 and HPV45, but not between other genotypes. Roche’s Cobas provides distinct results only for HPV16 and 18 and cannot distinguish between other HR genotypes. Therefore, the data extracted by Cerba provided binary results (i.e. positive or negative) for HPV16 and HPV18 for both assays, for HPV45 only for Alinity m high-risk, and for all other HR genotypes for both assays.

In addition to the test results, the dataset included the screening pathway, week and year of the sample analysis, individual’s age and postcode of the city of residence. Details about the French administrative organisation, including a map of postcodes, are provided in Supplement S2. No additional variables were available.

### Analytic sample selection

The detailed description of the sample selection steps and corresponding flowchart are provided in Supplement S3. In brief, we only included test results from females living in France, aged between 15 and 79 years, and for whom a non-identifying personal code and information about their age, postcode of residence and type of screening was available.

We considered all individuals aged 15 to 79 years, living in metropolitan France and in French overseas territories for descriptive statistics purposes only (reported in Supplement S4). The statistical analysis, aimed at fulfilling the study objectives, was conducted solely on patients from metropolitan France within the age range of 30 to 66 years, which we refer to as the ‘analytic sample’.

The upper age limit was set at 66 years to account for the delay between the receipt of the invitation from the organised screening programme and test uptake, which may lead females of this age to benefit from the free HR HPV test provided by the organised screening programme. The lower age limit aligns with the recommended age for HR HPV testing.

### Statistical inference

We performed a disease mapping statistical analysis [21], which aims to provide disease-related metrics at small-area levels, such as postcodes.

The number of tests and positive tests were aggregated at the stratum level. Strata were based on postcode, age, week-year of screening, genotype group and screening pathway. Two groups of genotypes were considered: HPV16 and/or HPV18 (HPV16/18) and genotypes other than HPV16/18 (other genotypes).

The number of positive tests within each stratum was assigned a binomial distribution, the size parameter of which was the total number of tests performed within each stratum. The expected proportion of positive tests within a stratum was linked, through the standard logistic function, to a set of predictors using the stratum’s characteristics as input.

Each stratum’s predictor included a global intercept and additional parameters associated with the full two-way interaction between dummy variables for the group of other genotypes and opportunistic screening. Other parameters were divided into two groups. The first group was common to data collected through opportunistic and organised screening, while the second was specific to data collected through opportunistic screening. Each group of parameters contained spatially varying, age-varying and time-varying parameters to which we assigned multivariate hierarchical priors. Six different models were considered, each with a different prior spatial structure. Among these six spatial models, four used reparametrised and scaled Besag York Mollié (BYM2) spatial priors [22]. For each model, the BYM2 priors assumed a different adjacency matrix (Delaunay triangulation, sphere of influence subgraph of the Delaunay triangulation, and first and second order Queen contiguity). The other two spatial models were Gaussian random fields (GRF), one was an isotropic and stationary GRF [23] and the other was the Barrier model [24]. The GRF models used the centroid of each postcode as location of sampling.

Competing models were discriminated through their log-scores [25], which were computed using the leave-one-group-out cross-validation approach described in [26,27]. All models were estimated using the inlabru package [28,29], a wrapper around the R-INLA package [30,31], in R software version 4.4.0 [32]. Further details are provided in Supplement S5.

## Results

### Descriptive reporting for the analytic sample

The test dates ranged from 17 August 2020 (week 33 2020) to 30 November 2023 (week 48 2023). The analytic sample contained the results of 362,963 HR HPV tests. The repartition of the sample between postcodes showed a low sampling density across space except in some areas located around large cities, see Supplement S6. Of these, 3.7% and 9.2% were positive for HPV16/18 and for other genotypes, respectively.

Of the total number of tests, 343,042 (94.5%) came from opportunistic screening and 19,921 (5.5%) from organised screening. For opportunistic screening, these percentages were 3.8% and 9.4%, for HPV16/18 and other genotypes, respectively. For organised screening, they were 2.9% and 6.9% for HPV16/18 and other genotypes, respectively. Stratified observed prevalence is reported in Supplement S7.

### Posterior predictive check for the selected model

The selected model’s spatial components were specified through the BYM2 prior, with the first-order Queen contiguity structure as the neighbourhood matrix. It reproduced the observed prevalence and found a posterior average expected (95% equal-tailed interval (ETI)) cervical infection prevalence in the analytic sample of 3.7% (95% ETI: 3.7–3.8) for HPV16/18 and 9.3% (95% ETI: 9.2–9.4) for other genotypes. The posterior average expected cervical infection prevalence among opportunistic tests was 3.8% (95% ETI: 3.7–3.8) for HPV16/18 and 9.4% (95% ETI: 9.3–9.5) for other genotypes. For organised screening, they were 2.9% (95% ETI: 2.7–3.1) and 6.9% (95% ETI: 6.6–7.3), respectively. Additional stratification can be found in Supplement S7.

### Effect of screening pathway on expected prevalence of high-risk human papillomavirus among females aged 30 years

The posterior distribution of the spatial-specific difference between expected HR HPV cervical infection prevalence generated using the model specific to each type of screening made it possible to quantify the difference in expected prevalence associated with opportunistic screening relative to organised screening. For comparison purposes and given the nonlinear relationship between the expected prevalence and strata characteristics, we focused on females aged 30 years, as of the study’s last observed week at the end of November 2023 (week 48 2023).

At the end of the study period, opportunistic screening was associated with a greater HPV infection prevalence among females aged 30 years, compared with organised screening, in more than the majority of postcodes, as exhibited by the lower bound value of the 95% ETI. This was identified on average in 97.2% (95% ETI: 72.9–100) of postcodes for HPV16/18 and 99.9% (95% ETI: 99.3–100) of postcodes for other genotypes.

**Figure 1:**
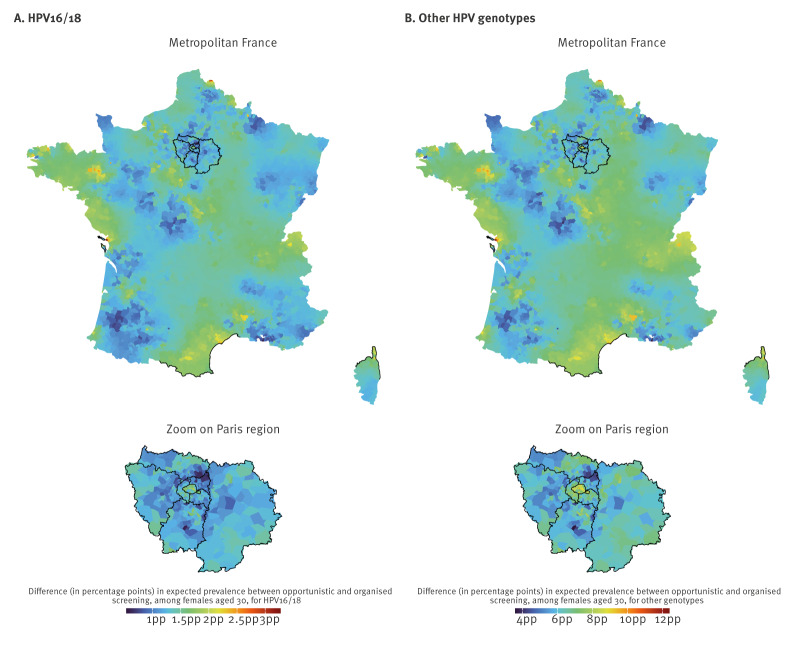
Posterior average difference in expected prevalence of high-risk human papillomavirus cervical infection between opportunistic vs organised screening in females aged 30 years, France, at week 48 2023

The difference in HR HPV infection prevalence between opportunistic and organised screening, among females aged 30 years in the postcode with the greatest 95% ETI lower bound value was on average 3.3 percentage points (pp) (95% ETI: 1.1–5.6) for HPV16/18 and 12.4 pp (95% ETI: 7.3–18.3) for other genotypes (Figure 1).

HPV: human papillomavirus.

Values are in percentage points (pp), among females aged 30 years at week 48 2023. Maps with 95% equal-tailed interval are provided in Supplement S8.

### Maps of the expected high-risk human papillomavirus cervical infection prevalence among females aged 30 years

The joint posterior distribution of the model specific to organised screening allowed us to generate the spatial-specific expected HR HPV cervical infection prevalence from organised screening of females aged 30 years at the end of November 2023 (week 48 2023). This made it possible to produce what, to our knowledge, is the first spatially resolved nationwide mapping of this quantity for France.

Once we removed the systematic inflation associated with opportunistic screening, by considering only the model components specific to organised screenings, a pronounced north-west/south-east gradient of HR HPV infection prevalence was found across France for both genotype groups. Hotspots were identified at the borders with Italy, Spain and Switzerland.

**Figure 2:**
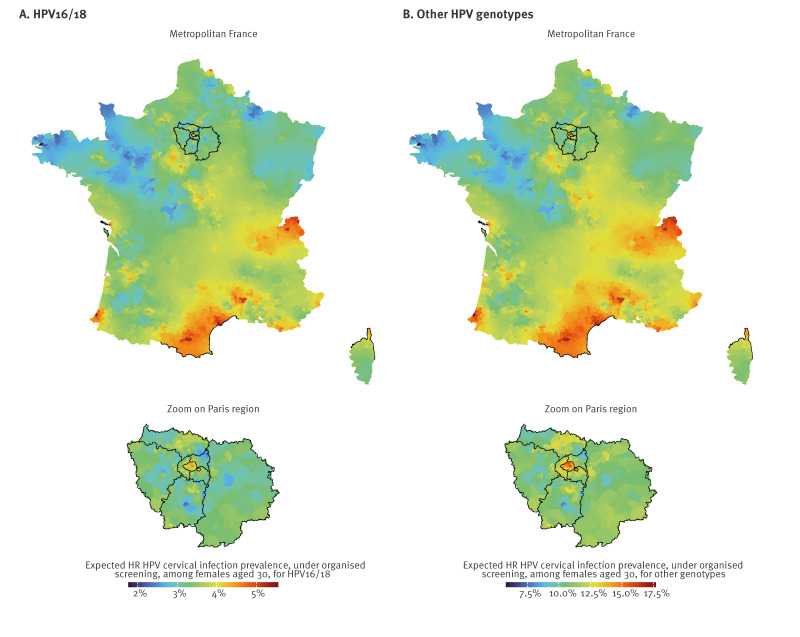
Posterior average expected high-risk human papillomavirus cervical infection prevalence during organised screening among females aged 30 years, France, at week 48 2023

HPV: human papillomavirus; HR: high-risk.

The areas in which we detected the lowest 95% ETI lower bound value for expected prevalence had a posterior average expected prevalence of 2.4% (95% ETI: 0.8–5.6) for HPV16/18 and 8.1% (95% ETI: 3.0–16.8) for other genotypes. By contrast, the area in which the highest lower bound was detected had a posterior average expected prevalence of 8.6% (95% ETI: 6.3–11.2) for HPV16/18 and 29.5% (95% ETI: 23.7–35.8) for other genotypes (Figure 2A and B). Maps with 95% ETI upper and lower bounds are provided in Supplement S9.

Maps with 95% equal-tailed interval upper and lower bounds are provided in Supplement S9.

### Age-specific expected high-risk human papillomavirus cervical infection prevalence in major French cities

To study how expected HR HPV cervical infection prevalence changes with age, we restricted the evaluation of posterior expected prevalence at the end of November 2023 to eleven major French cities spread around mainland France (see Supplement S2 for their locations).

The likely systematic upward inflation identified in opportunistic screening prevalence among females aged 30 years extended to almost all ages in the major French cities included in our analysis. In Paris, the posterior 95% ETI lower bound value for this upward inflation decreased from 2.0pp (95% ETI: 0.2–3.7) for HPV16/18 and 9.4pp (95% ETI: 5.1–13.8) for other genotypes among females aged 30 years to 1.0pp (95% ETI: -0.3–2.3) and 2.7pp (95% ETI: 0.1–5.4), respectively, among females aged 66 years.

**Figure 3:**
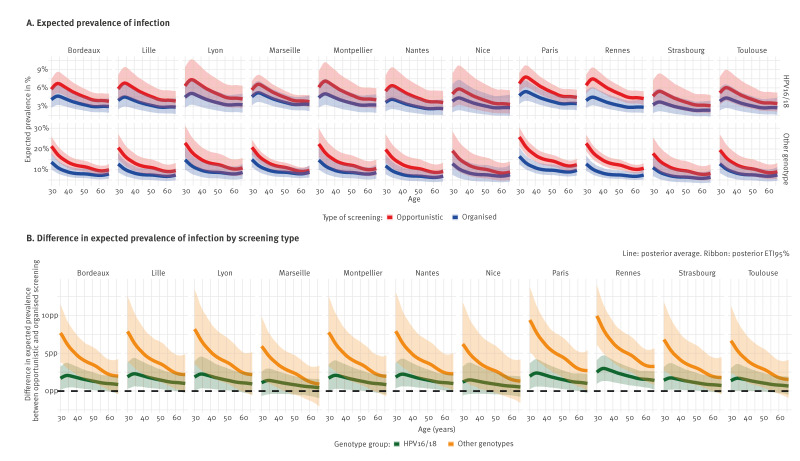
Summary of the posterior distribution of the expected prevalence of high-risk human papillomavirus (HPV) cervical infection from organised and opportunistic screening for genotype (HPV16/18 or other), in major French cities, at week 48 2023

When removing the systematic inflation associated with opportunistic screening, posterior average expected HPV16/18 cervical infection prevalence in Paris decreased from 4.7% (95% ETI: 3.3–6.6) among females aged 30 years to 3.4% (95% ETI: 2.3–4.9) among females aged 66 years. For other genotypes, it decreased from 16.4% (95% ETI: 12.6–20.7) to 9.7% (95% ETI: 7.1–12.8), respectively (Figure 3A and B). Maps for the entire country are shown in Supplement S10.

**Figure 4:**
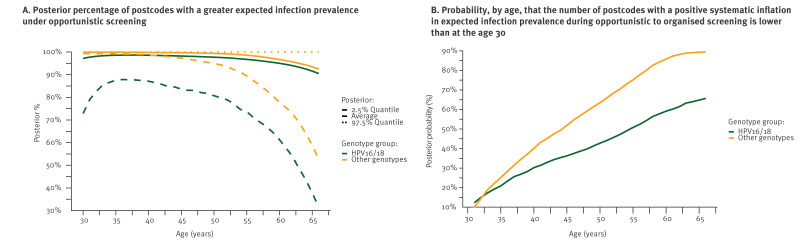
Summary of the distribution of the number of postcodes for which opportunistic screening was associated with a systematic inflation in the expected high-risk human papillomavirus, France, at week 48 2023

Among females aged 66 years, opportunistic screening was associated with a systematic inflation in expected infection prevalence in 90.5% (95% ETI: 31.4–100) of postcodes for HPV16/18 and 92.5% (95% ETI: 52.7–100) of postcodes for other genotypes, see Figure 4A. There was a 65.7% and 89.4% chance for these percentages to be lower than these computed for HPV16/18 and other genotypes, respectively, among females aged 30 years, see Figure 4B.

HPV: human papillomavirus.

Table counterparts for these plots are provided in Supplement S11. All variables were computed at the end of November 2023.

### Difference in expected infection prevalence between the two groups of genotypes within each screening pathway

For each screening pathway, the posterior mean difference in infection prevalence between the two groups of oncogenic genotypes followed a north-east-to-south-west gradient. In all included major French cities, the prevalence of HPV16/18 was almost certainly lower than that of the 12 other genotypes. Moreover, both screening pathways tended to yield smaller differences in expected prevalence between the two genotype groups among older females compared with younger ones (see Supplement S12).

### Temporal changes in expected high-risk human papillomavirus cervical infection prevalence

**Figure 5:**
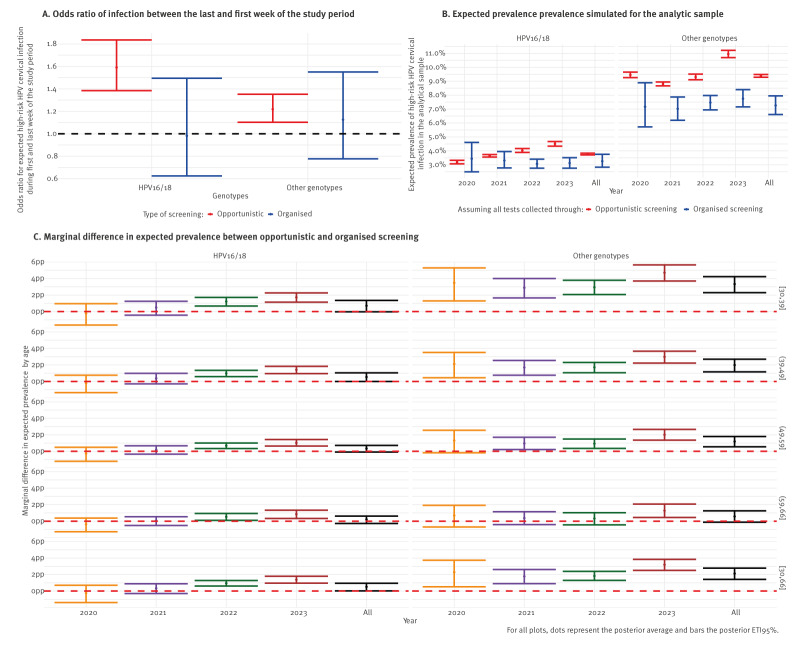
Summary of the posterior distribution for the marginal difference in expected high-risk human papillomavirus cervical infection prevalence associated with organised and opportunistic screening, in the analytic sample, France, 2020–2023

The posterior distribution for expected HR HPV cervical infection prevalence from opportunistic screening showed an increase in expected infection prevalence in week 48 2023, the last week of the study compared to week 33 2020, the first week of the study for both groups of genotypes. However, when considering organised screening, this positive temporal trend was not found with high certainty (Figure 5A and Supplement S13).

HPV: human papillomavirus.

Panels B and C are available in table format in Supplement S14.

### Marginal difference in expected high-risk human papillomavirus cervical infection prevalence associated with screening pathway

To go beyond the conditional picture explored so far, we computed the Marginal Difference in Expected Prevalence (MDEP), which measures the marginal difference in expected prevalence had all data been collected via opportunistic rather than organised screening. It accounts for all interactions and reflects how the within-sample expected prevalence would change depending on screening pathway.

For exclusive collection of data via organised screening, posterior average expected prevalence was 3.3% (95% ETI: 2.8–3.8) for HPV16/18 and 7.3% (95% ETI: 6.6–7.9) for other genotypes. The alternative situation, in which all strata would have been collected through opportunistic screening, was associated with an average change in the expected prevalence of 0.5 pp (95% ETI: 0.0–0.9) and 2.1 pp (95% ETI: 1.4–2.8) for HPV16/18 and other genotypes, respectively (Figure 5A and B). The MDEP stratified by age and year is provided in Figure 5C.

### Sensitivity analysis

Detailed results of the sensitivity analysis can be found in Supplement S15. In brief, the 95% ETI for the prevalence profiles in the major French cities and for the MDEP exhibited the same patterns, regardless of spatial prior and prior for correlation parameters.

## Discussion

Our study’s primary objective was to determine whether expected prevalence of HR HPV infection changed with the screening pathway. The analysis highlighted a positive systematic inflation of HR HPV cervical infection prevalence when using test results collected through opportunistic screening compared with those collected through organised screening in more than the majority of French postcodes, among 30-year-olds during week 48 2023. We could not identify with great certainty (probability > 0.95) that the number of districts with such a systematic positive inflation was lower among 66-year-olds.

The difference between the two screening pathways may be explained by different selection mechanisms leading to different types of individuals being screened through each pathway. In France, Kelly et al. [17] showed that opportunistic cervical cancer screening is associated with persistent socioeconomic inequalities, while Le Bihan-Benjamin et al. [6] concluded that females participating in the organised screening programme are less likely to comply with the French screening recommendation. More generally, Gianino et al. [18] highlighted that organised screening programmes contribute to gradually reducing the socioeconomic inequalities in younger people’s use of preventive services. Our results shed light on the possible consequences of using data from opportunistic screening, which may contribute to bias in prospective modelling studies using such routinely collected data and not accounting for this effect.

Our study’s secondary aim was to provide spatially-resolved maps of HR HPV cervical infection prevalence. For organised screening, these maps exhibited a north-east/south-west gradient in HR HPV infection prevalence, with prevalence hotspots identified near the border with Italy, Spain and Switzerland. Many countries, especially in Europe, have implemented nationwide organised HR HPV testing programmes, with the intent to increase screening uptake [13,33]. In France, according to the latest assessment carried out by the national public health institute Santé publique France (SpF), the coverage of cervical cancer screening uptake among females aged 25–65 years between 2020 and 2022 was 59.5%, below the 80% targeted by the programme [10]. In most of these organised screening programmes, including in France, invitations are dispatched based on the time since the previous screening, regardless of residence [13].

The heavy burden of cervical cancer and the benefits associated with HR HPV screening call for HR HPV testing uptake to be encouraged nationwide [14-16]. Our maps can, however, aid in identifying areas where screening and vaccination can be most effective, contributing towards optimising screening and vaccination strategy, which are two of the interventions recommended by the WHO strategy to eliminate cervical cancer [2,3,34]. The prevalence hotspots could, for example, be pinpointed as areas where increasing screening efforts might enhance the public health impact of the organised screening programme while limiting additional costs. The combination of a coherent spatially-targeted screening and vaccination strategy appears even more important as the identified spatial gradient is similar to the spatial gradient in HR HPV vaccine uptake reported by Ribassin-Majed et al. [35], providing a possible explanation for this pattern.

In the analytic sample for HPV16/18, for samples collected through organised screening, our model produced a posterior average expected cervical infection prevalence of 2.9% (95% ETI: 2.7–3.2). For other genotypes, this was 6.9% (95% ETI: 6.6–7.3). The 2023 Human Papillomavirus and Related Diseases World Report published by the Catalan Institute of Oncology (ICO) and the International Agency for Research on Cancer (IARC) (HPV Information Centre) found a pooled 95% confidence interval (CI) of 2.5–2.7 for the prevalence of HPV16/18 cervical infection among females with normal cytology in western Europe [36]. In France, the same report identified 12 studies estimating HR HPV cervical infection prevalence in females with normal cervical cytology, all published in 2014 or before [36]. Among these, Heard et al. [37] stands out with a dataset collected at 16 sampling sites between July 2009 and November 2012 as part of a pilot for the initial version of the French organised screening programme for HPV. The study implemented a stratified sampling scheme according to age group (25–29, 30–39, 40–49, 50–65 years), resulting in a total of 3,037 cervical samples. Of these samples, HPV16/18 was found in 3.9% (95% CI: 2.8–5.1).

Our 95% ETI closely aligns with the 95% CI reported by Head et al. [37], differing by only 0.1 at the lower bound. Similarly, the lower bound of our ETI coincides with the upper bound of the 95% CI reported by the ICO/IARC, although their lower bound is compatible with prevalence up to 0.2 lower.

The differences between our 95% ETI upper bound and those from Heard et al. [37] could primarily be due to differences in sample composition. The first difference lies in age composition. Heard et al. [37] selected participants based on a stratified sampling scheme. Conversely, we used all the tests performed by one of the leading networks of biology laboratories in France but provided model-based age-specific infection prevalence estimates. A second source of heterogeneity is related to vaccination, the rate of which increased between the previous study and ours. In 2022, 33.8% of girls aged 16 were fully vaccinated according to the French vaccination guidelines, compared with 15.5% in 2017 [38]. A third source of heterogeneity originates from differences in spatial sampling. We used test results from all over France whereas Heard et al. [37] included samples from only five of the 22 French regions, none of which were located in the east of France where we found the lowest posterior average expected infection prevalence.

Our study provides the first spatially resolved picture of HR HPV cervical infection prevalence in metropolitan France. It did so despite scarce sampling and data dependency across space and time. Scarce sampling may prevent the computation of the quantities of interest or may be the source of unstable estimates with large, implausible, uncertainty intervals [39]. Data dependency manifests itself through spatial and temporal autocorrelation and must be accounted for when computing uncertainty intervals [40]. State-of-the-art disease mapping analysis uses hierarchical models estimated within a full Bayesian framework, which allow us to capture both the relationship between data points and to leverage information from densely sampled areas to enhance estimates in sparsely sampled regions [21,41,42]. Our modelling approach was designed to handle nonlinear age-specific prevalence patterns, the spatial structure of the dataset and multiple genotype groups and screening types within a unified statistical modelling approach.

Our work has three main limitations. First, the large grid used to define strata caused high computational and storage costs, preventing us from accounting for interactions between dimensions. As a result, all models, despite their flexibility, accounted for each dimension independently on the logit scale.

Second, the dataset did not allow for the study of organised screening uptake among eligible females. A report by SpF found that 11.6% of screening tests for females aged 30–65 years in 2020–2022 were performed through the organised screening programme, i.e. following the receipt of an invitation letter [10]. The launch of the updated version of the organised screening programme amidst the COVID-19 pandemic, in July 2020, may have slowed down the roll-out of these invitations and their uptake. However, reported screening coverage for females aged 25–65 years was higher in 2020–2022 than in 2017–2019, indicating a plausible limited impact or a catch-up dynamic during time periods with limited disruption of the healthcare sector [10]. Similarly, we were unable to assess the coverage of our dataset relative to the total population of women participating in the organised screening programme.

Third, we showed that data from opportunistic screening were likely associated with a systematic inflation in HR HPV infection prevalence across mainland France compared with organised screening. However, because screening uptake following receipt of an invitation is not mandatory, data collected through organised screening may not give a more accurate reflection of the true prevalence than opportunistic screening. Therefore, residual selection biases affect both estimated prevalence. Modelling the selection process to address this concern is typically not possible using data routinely collected by medical biology laboratories.

## Conclusion

This study provides an original, national and spatially resolved picture of HR HPV cervical infection prevalence in France, identifying a north-west/south-east gradient and hotspots located at the border with Italy, Spain and Switzerland. It also highlights the likely systematic upward inflation in expected prevalence associated with opportunistic screening compared with organised screening.

Similar quantification of the differences between screening pathways for other infections, whose prevalence studies heavily rely on opportunistic sampling, should be performed to improve the reliability of future prospective modelling work assessing the effect of public health interventions.

## Data Availability

Data cannot be shared due to legal constraints associated with the use of the French reference methodology MR-004. Codes are available on the first author’s GitHub page: https://github.com/osupplisson/hpv_prevalence.

## Supplementary Data

65114000 Supplementary Material
