# Supplementary material for: High-risk human papillomavirus cervical infection prevalence: a nationwide retrospective study comparing opportunistic and organised screening, France, 2020 to 2023
Source: Euro Surveill. 2025 Jul 17;30(28):2400689. doi: 10.2807/1560-7917.ES.2025.30.28.2400689 (PMC12273260; doi:10.2807/1560-7917.ES.2025.30.28.2400689)
Supplement: Supplementary Material [file 24-00689_SUPPLISSON_Supplement.pdf]

Supplementary Files

This supplementary material is hosted by Eurosurveillance as supporting information alongside the article ‘High-risk human papillomavirus cervical infection prevalence: a nationwide retrospective study comparing opportunistic and organised screening, France, 2020 to 2023’, on behalf of the authors, who remain responsible for the accuracy and appropriateness of the content.

The same standards for ethics, copyright, attributions and permissions as for the article apply.

Supplements are not edited by Eurosurveillance and the journal is not responsible for the maintenance of any links or email addresses provided therein.

Contents

S1 Additional details about the French cervical cancer screening guidelines 6
S2 Details about the French administrative organisation 7
S3 Analytical sample selection flowchart 12
S4 Descriptive reporting for the 'Descriptive samples' 14
S4.1 Descriptive reporting among females aged [15-79] in metropolitan France 14
S4.2 Descriptive reporting among females aged [15-79] in the overseas territories . . . 20
S5 Additional statistical details 33
S5.1 Complete textual description of the statistical model 33
S5.2 Mathematical details of the statistical model 36
S5.3 Details about the sensitivity analyses 47
S5.4 Details about the Marginal Difference in Expected Prevalence (MDEP) 50
S6 Descriptive reporting for the analytical sample 51
S7 Performance of the selected model 57
S7.1 Model selection and raw reporting of the selected model 57
S7.2 Posterior predictive check from the selected model 62
S8 Systematic difference in expected HR HPV cervical infection prevalence between opportunistic and organised screening, among females aged 30, at the end of November 2023 114
S9 Expected HR HPV cervical infection prevalence, among females aged 30, at week 48 of 2023, under organised screening 123
S10 Maps of the posterior average expected infection prevalence stratified by age, at week 48 of 2023, under organised screening 129
S11 Figure 3A and B and 4A and B in table format 131
S12 Difference in expected HR HPV cervical infection prevalence, between the two groups of genotypes 222
S13 Difference in expected HR HPV cervical prevalence between the last and first week of the study period, stratified by age 228
S14 Figure 5B and C in table format 229
S15 Sensitivity analyses 235
S15.1 Change in the variance of priors assigned to the (latent) correlation parameters . 235
S15.2 Non-selected competing models 237
S16 References for the Supplementary Files 239

List of Figures

S1 Layout of postcodes in metropolitan France. 8
S2 Centroid of the major French cities in metropolitan France. For Paris, Lyon, and Marseille, the centroid of the 1st arrondissement is displayed. 9
S3 Administrative subdivisions of metropolitan France. 10
S4 Administrative subdivisions of Paris region (Île-de-France). 11
S5 Analytical sample selection flowchart. 13
S6 Descriptive reporting for females aged [15-79] in metropolitan France. A) Number of HR HPV tests, stratified by year and screening pathway. B) Number of tests, stratified by age, and screening pathway. C) Proportion of tests by result (positive for HPV16, positive for HPV18, positive for other genotypes, and negative), stratified by year and screening pathway. D) Proportion of tests by result, stratified by age and screening pathway. 16
S7 Number of tests performed in each time step (year-week), stratified by screening pathway. 18
S8 Spatial location of all data points (green dots) and mesh (grey edges) used for the finite-elements decomposition approximation of the Gaussian random fields. The blue line delineates the areas in which the density of the mesh could change. It was defined to be finer within metropolitan France than outside, where no data could be observed. 45
S9 Prior density for a Gaussian prior distribution with PC(1,0.5) hyperprior assigned to the precision parameter (blue curve) compared with a standard Gaussian distribution (red curve) 46
S10 PC prior for the standard deviation of GMRFs. 47
S11 Precision for the prior distribution of the latent correlation parameters. 48
S12 Spatial neighbourhood matrices considered for BYM2 priors. Black squares indicate that the two areas are neighbours. 49
S13 Postcodes with at least one test result observed in the analytical sample. 51
S14 Postcodes stratified by the number of HR HPV tests in the analytical sample. 52
S15 Postcodes stratified by the number of HR HPV tests in the analytical sample, zoom on the Paris region. 53
S16 Postcodes only observed through opportunistic screening, organised screening, both opportunistic and organised screening, and not observed in the analytical sample. . . . 54
S17 Postcodes only observed through opportunistic screening, organised screening, both opportunistic and organised screening, and not observed in the analytical sample, zoom on Paris region. 55
S18 Empirical cumulative distribution function of the number of tests within postcodes in the analytical sample. 56
S19 Posterior and prior distribution for the correlations between the two groups of genotypes for components specific to organised screening. 60
S20 Posterior and prior distribution for the correlations between the two groups of genotypes for components specific to opportunistic screening. 61

S21 Posterior difference between the expected HR HPV cervical infection prevalence from opportunistic and organised screening, among females aged 30, at week 48 of 2023. . . . 116
S22 Posterior difference between the expected HR HPV between screening pathways, among females aged 30, at week 48 of 2023, in Paris region. 117
S23 Posterior probability of a positive difference in expected HR HPV between opportunistic and organised screening, stratified by genotype group, among females aged 30, at week 48 of 2023. 118
S24 Posterior systematic inflation in HR HPV cervical infection prevalence under opportunistic screening, compared with organised screening, in each of the Paris arrondissements, among females aged 30, at week 48 of 2023. 119
S25 Posterior systematic inflation in HR HPV cervical infection prevalence under opportunistic screening, compared with organised screening, in each of the Lyon arrondissements, among females aged 30, at week 48 of 2023. 120
S26 Posterior systematic inflation in HR HPV cervical infection prevalence under opportunistic screening, compared with organised screening, in each of the Marseille arrondissements, among females aged 30, at week 48 of 2023. 121
S27 Posterior HR HPV cervical infection prevalence in metropolitan France, among females aged 30, at week 48 of 2023, correcting for the systematic inflation associated with opportunistic screening, stratified by genotype group. 123
S28 Posterior HR HPV cervical infection prevalence in Paris region, among females aged 30, at week 48 of 2023, correcting for the systematic inflation associated with opportunistic screening, stratified by genotype group. 124
S29 Posterior HR HPV cervical infection prevalence in each of the Paris arrondissements, among females aged 30, at week 48 of 2023, correcting for the systematic inflation associated with opportunistic screening, stratified by genotype group. 125
S30 Posterior HR HPV cervical infection prevalence in each of the Lyon arrondissements, among females aged 30, at week 48 of 2023, correcting for the systematic inflation associated with opportunistic screening, stratified by genotype group. 126
S31 Posterior HR HPV cervical infection prevalence in each of the Marseille arrondissements, among females aged 30, at week 48 of 2023, correcting for the systematic inflation associated with opportunistic screening, stratified by genotype group. 127
S32 Posterior average expected infection prevalence for HPV16/18 under organised screening, across metropolitan France, stratified by age. 129
S33 Posterior average expected infection prevalence for Other genotypes, under organised screening, across metropolitan France, stratified by age. 130
S34 Summary of the posterior distribution of the difference (in percentage points) in infection prevalence caused by HPV16/18 and other genotypes, under organised screening, among females aged 30, at week 48 of 2023. 222
S35 Summary of the posterior distribution of the difference (in percentage points) in infection prevalence caused by HPV16/18 and other genotypes, under opportunistic screening, among females aged 30, at week 48 of 2023. 223
S36 Posterior average expected difference in infection prevalence between other genotypes and HPV16/18, across metropolitan France, stratified by age, under organised screening.224
S37 Posterior average expected difference in infection prevalence between other genotypes and HPV16/18, across metropolitan France, stratified by age, under opportunistic screening. 225
S38 Summary of the posterior distribution of the difference (in percentage points), between opportunistic and organised screening, in the expected HR HPV cervical infection prevalence, in major French cities, at week 48 of 2023, stratified by age and genotype group. 226

S39 Difference in expected HR HPV cervical infection prevalence, between the last and first week of the study period in major French cities, stratified by screening pathway and age, for HPV16/18. . . . 228
S40 Posterior average [ETI95%] for the expected HR HPV cervical infection prevalence in 11 major cities in France. The precision is defined as 1/variance: the greater the precision, the lower the variance. The baseline case, precision = 0.2, has a U-shape. A precision of 0.4 looks like a uniform distribution on [-1,1]. At a precision of 0.8, extreme values for the correlation are very unlikely. . . . 235
S41 Posterior average [ETI95%] for the Marginal Difference in Expected Prevalence, stratified by precision for the priors of the latent correlation parameters, age, and year. The precision is defined as 1/variance: the greater the precision, the lower the variance. The baseline case, precision = 0.2, has a U-shape. A precision of 0.4 looks like a uniform distribution on [-1,1]. At a precision of 0.8, extreme values for the correlation are very unlikely. . . . 236
S42 Posterior average [ETI95%] for the conditional prevalence in major French cities, at week 48 of 2023, stratified by genotype group and type of Matérn covariance function (stationary or non-stationary model). . . . 237
S43 Posterior average [ETI95%] for the Marginal Difference in Expected Prevalence, stratified by the type of spatial component, age, and year. . . . 238

List of Tables

S1 Number of tests and observed cervical infection prevalence, stratified by year and screening pathway. Table counterpart to Supplementary Figure S6A and C. 17
S2 Proportion of tests with coinfection, stratified by screening pathway and age. 19
S3 Number of tests and observed infection prevalence, stratified by age and screening pathway. Table counterpart to Figure S6B and D. 19
S4 Number of tests and observed prevalence, stratified by genotype group, screening pathway, year, age, and district. 20
S5 Logarithmic-score based on LOGO-CV. The model with the minimum score was selected. 57
S6 Raw summary provided by `R-INLA` of the posterior distribution for the latent parameters. 57
S7 Raw summary provided by `R-INLA` of the posterior distribution for the hyperparameters. 58
S8 Observed HR HPV cervical infection prevalence, posterior predictive HR HPV cervical infection prevalence, and posterior expected HR HPV cervical infection prevalence, stratified by various dimensions. 62
S9 Posterior expected HR HPV cervical infection prevalence (in %) in major French cities, stratified by type of test, city, and age. Table counterpart to Figure 3A. 131
S10 Posterior difference in expected HR HPV cervical infection prevalence (in percentage points) in major French cities, between opportunistic and organised screening, stratified by type of test, city, and age. Table counterpart to Figure 3B. 187
S11 Posterior percentage of postcodes with a greater expected infection prevalence under opportunistic screening, than under organised screening, stratified by age and genotype group, as of November 2023. Table counterpart to Figure 4A. 215
S12 Posterior distribution of the difference in the number of postcodes with a higher expected prevalence under opportunistic screening versus organised screening at each age, relative to females aged 30, as of end of November 2023. Table counterpart to Figure 4B. 218
S13 Posterior expected HR HPV infection prevalence (in %) assuming all data would have been collected with opportunistic or organised screening, not stratified or stratified by age, year, or age and year. Provides values from Figure 5B in table format. 229
S14 Marginal Difference in Expected Prevalence (in percentage points), not stratified or stratified by age, year, or age and year. Provides values from Figure 5C in table format. 233

S1 Additional details about the French cervical cancer screening guidelines

The latest French guidelines for cervical cancer screening were updated in 2019. These guidelines recommend, for females aged 25 to 29, 2 cytological analyses at a one-year interval if the first result is normal, and, in the absence of abnormality on both cytological analyses, a repeat cytological analysis three years later. In the case of an abnormal cytology result, an HR HPV test should be performed.

For females aged 30 to 65, an HR HPV test should be performed as the first-line test. The first HR HPV test should be performed three years after the last normal cytology result, or as early as age 30 in the absence of a prior cytology result. If the HR HPV test is negative, the next HR HPV test should be performed 5 years later. In case of a positive HR HPV test for HR HPV, a cytological analysis should be performed within the year. If the cytology result is normal, an HR HPV test should be performed a year later [1, 2]. A detailed note about the management of females with abnormal cervical cytology and positive HR HPV is available in [3].

In France, females can undergo cervical cancer screening through two pathways. First, upon receipt of an invitation from the French National Social Security System, patients can be screened as part of the French National Cervical Cancer Screening programme.

The current version of this national organised screening programme was set up 30 July 2020. The legal document creating this new national screening programme can be accessed on the following link: https://www.legifrance.gouv.fr/loda/id/JORFTEXT000042238343/ (accessed 15th January 2025).

The national organised screening programme proactively invites, by postal mail, females who do not follow French screening guidelines to consult a doctor, a midwife, or a gynaecologist for a medical examination. As part of this medical examination, a cervical smear can be collected, which is then sent to medical biology or anatomical pathology laboratories for analysis. Alternatively, the biological sampling and analysis can be performed at a medical biology laboratory, following prescription from the primary care provider. Under this screening pathway, referred to as 'organised screening', the biological analysis is free for the patient and the primary care consultation is reimbursed according to standard nationwide rules.

Starting in mid-2024, invitations are also sent online, and the visit to a primary care provider is no longer required: participants can directly visit a medical biology laboratory for biological sampling and analysis.

As an alternative to the organised screening pathway, patients may undergo 'opportunistic screening', meaning that patients spontaneously consult a primary care provider without any invitation from the French National Social Security System. Any subsequent HR HPV test after this initial consultation is then subject to an out-of-pocket payment.

S2 Details about the French administrative organisation

One key issue we had to address was the misalignment between two available spatial IDs:

- The France administrative dataset identifies cities using a single unique ID, known as the code commune;
- The dataset extracted from Cerba provided the postcode of each patient. Postcodes are defined based on postal delivery routes;

Three cases could occur:

- the postcode corresponds to a single unique ID in its entirety (one-to-one relationship);
- the postcode corresponds to several unique IDs in their entirety (one-to-many relationship);
- the postcode corresponds to a single unique ID but not in its entirety (many-to-one relationship);

To deal with this issue, we relied on the only available open-source dataset linking both spatial dimensions, as of 2014, available on https://www.data.gouv.fr/fr/datasets/fond-de-carte-des-codes-postaux/. As a result of this discrepancy between these two spatial IDs, one postcode may include several cities.

The dataset contained only postcodes for metropolitan France. We are not aware of a similar dataset for Overseas territories.

The initial data contained 6,048 non-overlapping spatial areas, each associated with a unique postcode in metropolitan France. Among these, we kept only cities not located on small islands around metropolitan France. We ended up with 6,033 unique postcodes, which were used to build the spatial neighbourhood matrix.

Supplementary Figure S1 shows the spatial delimitation of all cities considered in the study.

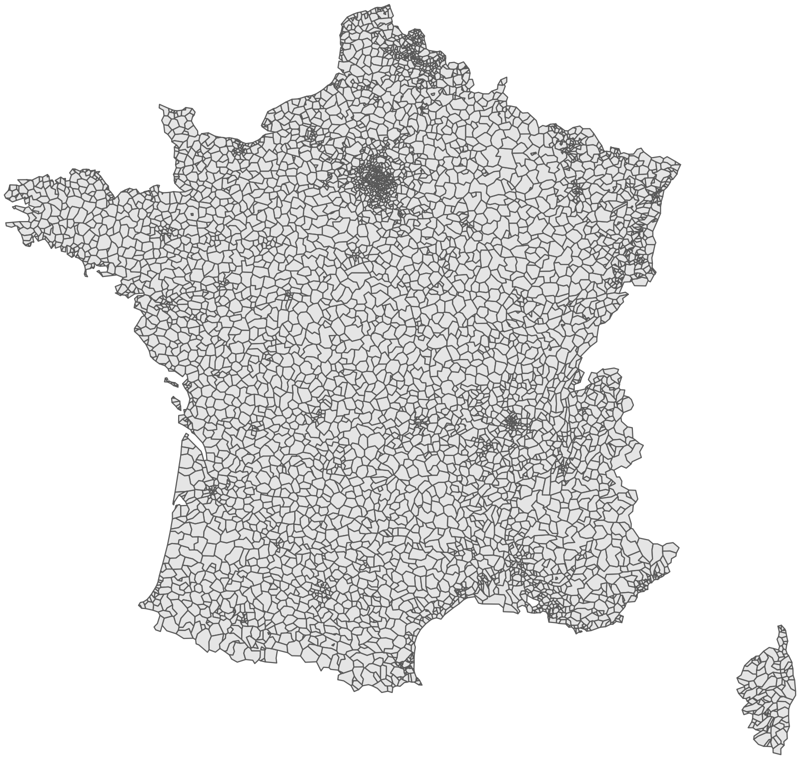

Figure S1 Layout of postcodes in metropolitan France.

Among all these cities, we considered specifically 11 of them, for computing the conditional prevalence (Supplementary Figues S2.

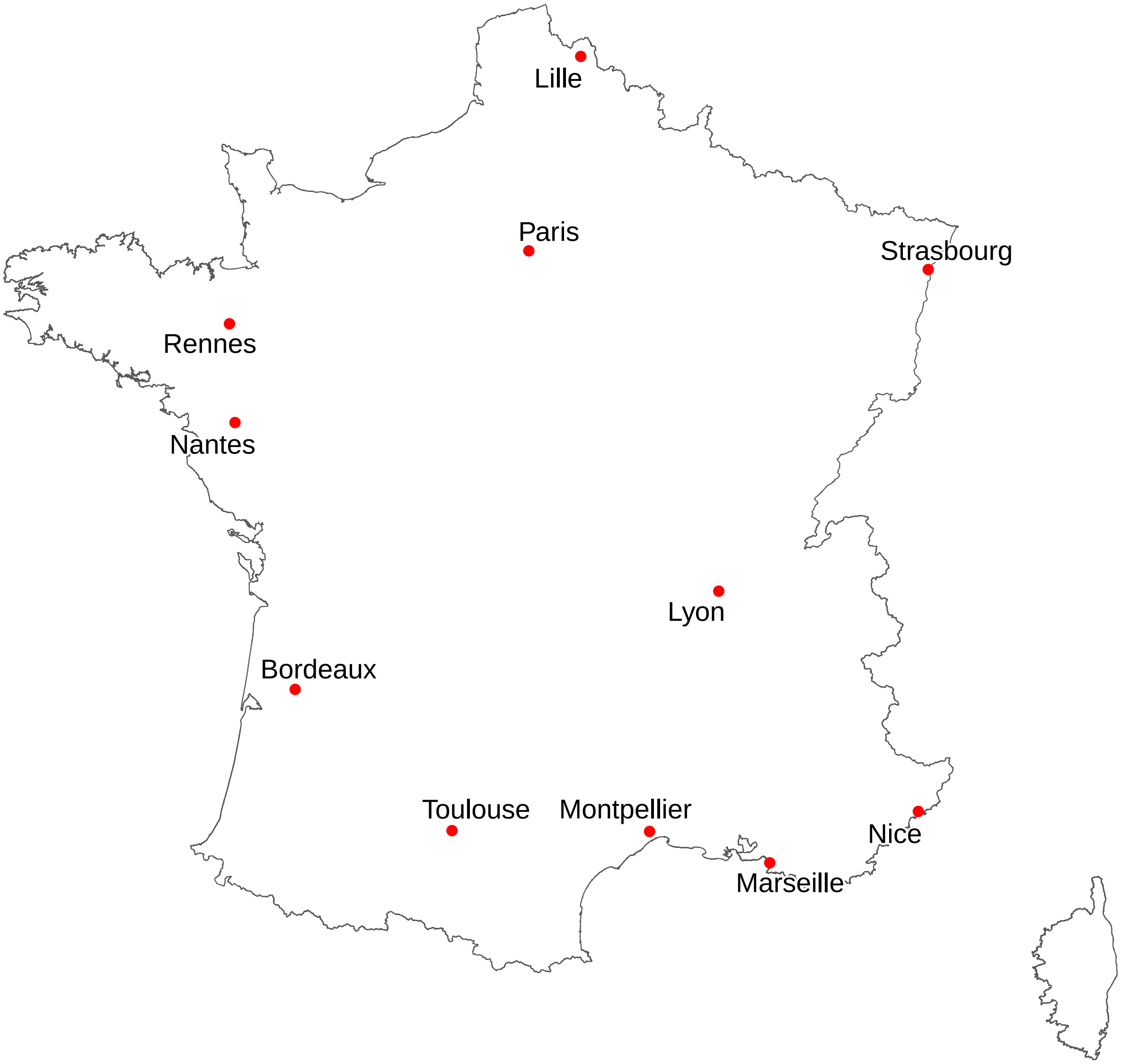

Figure S2 Centroid of the major French cities in metropolitan France. For Paris, Lyon, and Marseille, the centroid of the 1st arrondissement is displayed.

In France, cities are organised within administrative divisions known as 'départements' ('districts', in the main text). metropolitan France comprises 96 districts, grouped into 13 regions. Two of these districts are situated on Corsica, an island located between France and Italy. France's administrative structure is illustrated in Supplementary Figure S3. Paris, France's capital city, lies at the centre of the Île-de-France region, depicted in Supplementary Figure S4. In addition to districts located in metropolitan France, 5 districts are located in the overseas territories: Martinique, Guadeloupe, Guyane (French Guiana), Réunion and Mayotte. Each of these districts is also a region.

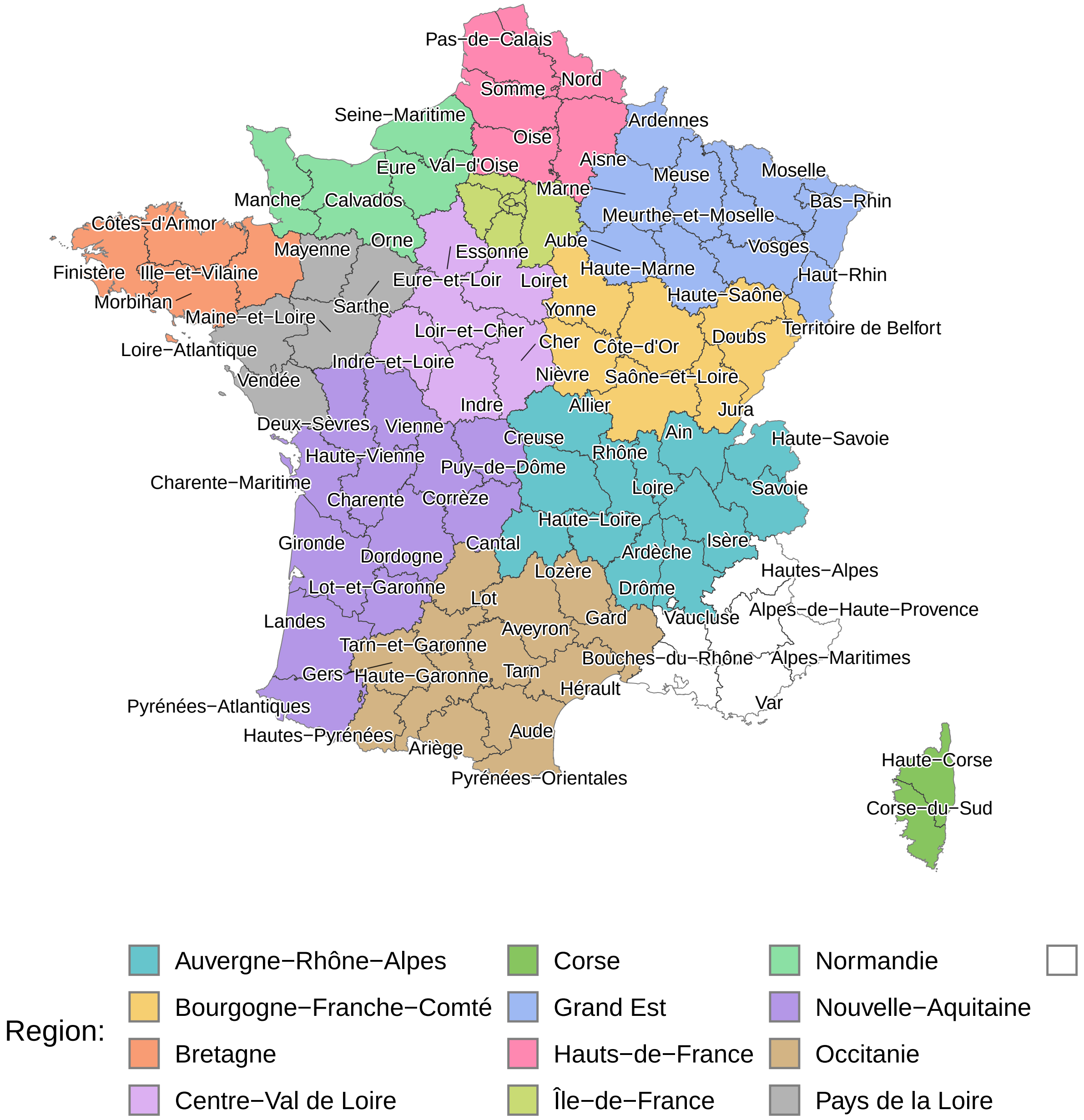

Figure S3 **Administrative subdivisions of metropolitan France.**

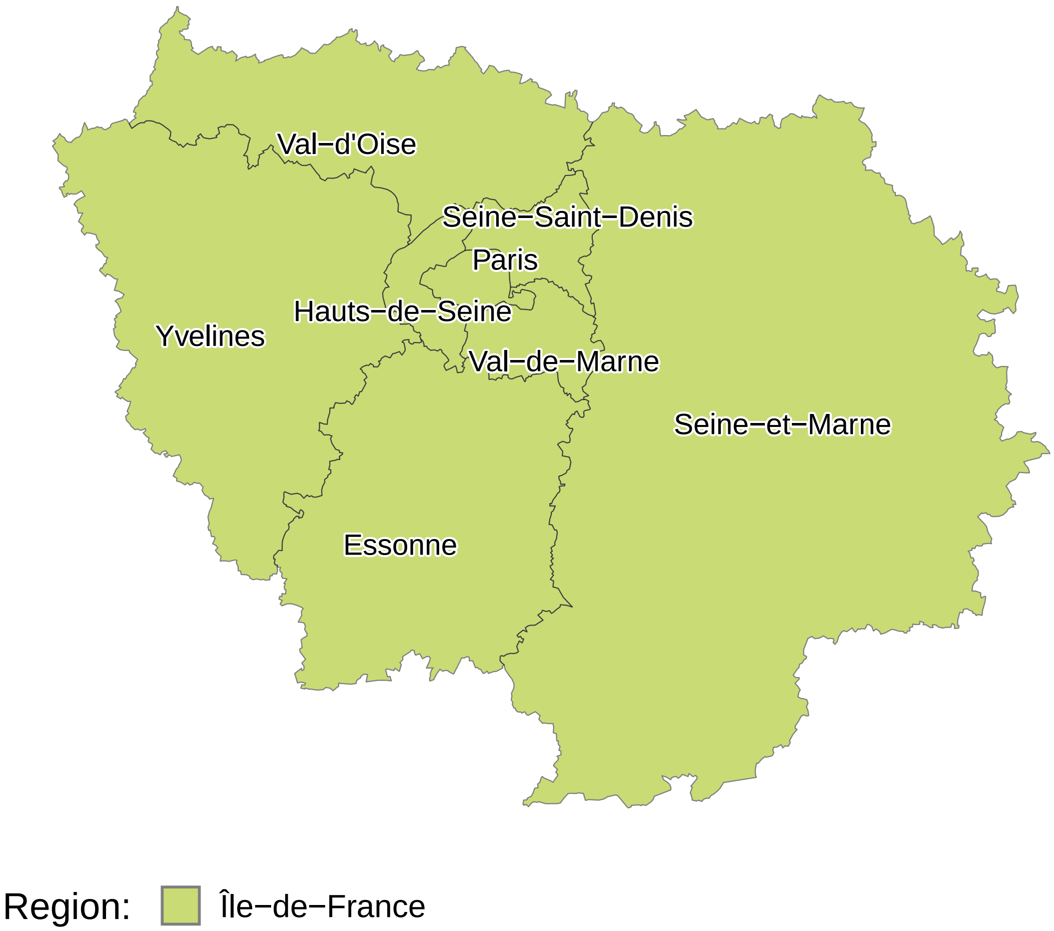

Figure S4 **Administrative subdivisions of Paris region (Île-de-France).**

S3 Analytical sample selection flowchart

All missing and erroneous items were due to issues at the time of patients' registration, before test uptake, and therefore could be considered as Missing Completely at Random with respect to test results.

Starting from the initial dataset, we excluded all test results associated with patients who had missing age or anonymous ID, because age was required for modelling, and an anonymous ID was necessary to ensure we did not consider the same individuals multiple times in the analysis.

We further restricted the dataset to include only patients aged 15 to 79. We did so because the number of tests outside this age range was small and therefore the descriptive reporting of the results were not meaningful due to a likely extreme selection process. We also suspected that patients recorded as aged 0 likely had erroneous birthdate entries in Cerba's database.

We then filtered out all tests with missing postcodes or associated with persons living outside France. We did this because our research focused on France.

As a next step, we filtered out tests recorded as both organised and opportunistic screening, which indicated a potential issue at the data reporting step.

Because invitations to take part in the organised screening programme are theoretically restricted to females aged 25 to 65, we removed all tests labelled as organised screening for patients younger than 25 or older than 66. We considered a one-year slack (66 instead of 65 years old) to allow for a delay between screening invitation and screening uptake.

We then filtered out tests associated with postcodes unmatched in the French administrative dataset.

In the case of individuals testing multiple times, we only kept the most recent test. We did so for two reasons. Focusing on the most recent test helped to slightly alleviate the computational burden associated with model fitting. Second, there were almost no individuals testing multiple times within the organised screening pathway, preventing us from considering individual-specific modelling that accounts for screening pathways.

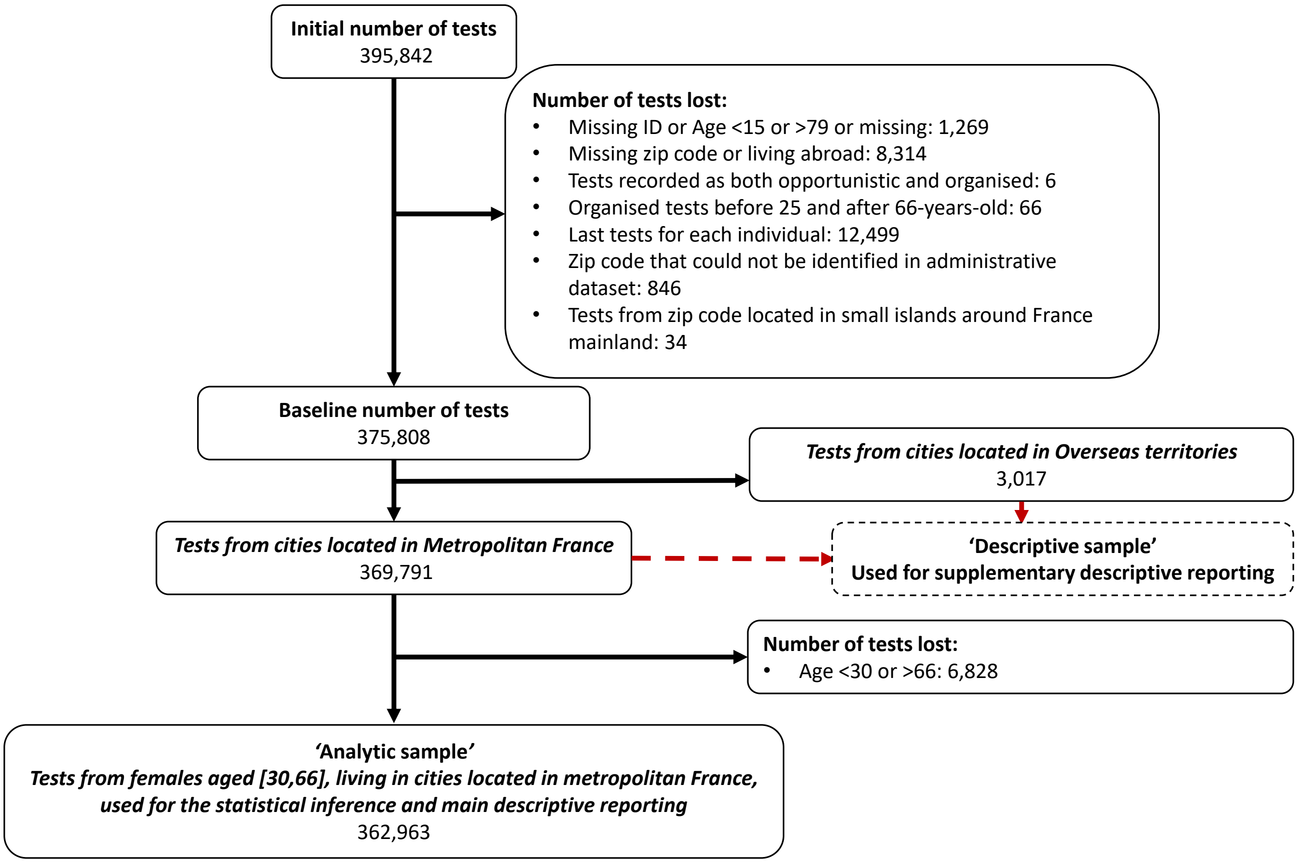

Figure S5 Analytical sample selection flowchart.

S4 Descriptive reporting for the 'Descriptive samples'

S4.1 Descriptive reporting among females aged [15-79] in metropolitan France

The descriptive sample contained the results of 369,695 tests, most of which (around 95%) were collected during opportunistic screening. Test dates ranged from the week of 2020-08-17 to that of 2023-11-27 (i.e., a total of 174 weeks). Supplementary Figure S7 reports the number of tests each week.

The breakdown of these tests by age was highly unbalanced for opportunistic screening but less so for organised screening (Supplementary Figure S6A and B). The number of tests was highly variable between years (Supplementary Figure S6A). This should not be interpreted as a variation in screening effort. First, the lower testing levels in 2020 are attributable to the COVID-19 pandemic context (social distancing measures such as lockdowns, disrupted medical follow-up, and avoidance of medical facilities for fear of higher transmission risk). Second, Cerba and other medical medical laboratories started to implement the 2019 guidelines during 2020. Third, the sharp drop for 2022 and 2023, compared with 2021, resulted from Cerba's internal organisation, with an important proportion of collected samples being sent for analysis to another entity of the Cerba group, for which we could not export the data.

During the entire study period, 88.2% of the tests were negative, 2.8% were positive for HPV16, 1.0% were positive for HPV18, and 9.3% were positive for other genotypes. The overall raw prevalence was greater for opportunistic screening (12.0%) than for the organised screening (9.1%). A similar result was found when stratifying by age and year (Supplementary Figure S6D). Details about coinfections are reported in Supplementary Table S2.

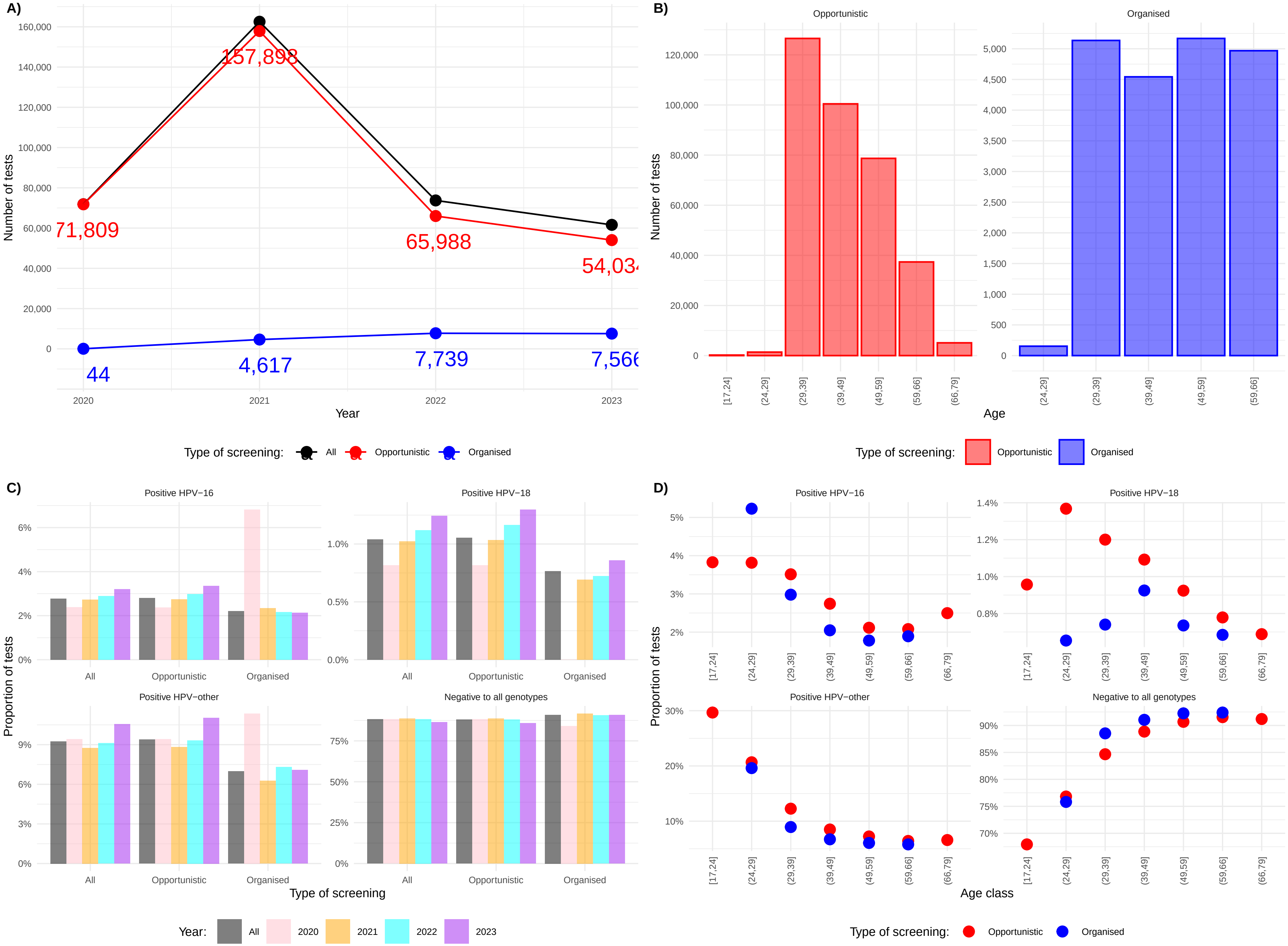

Figure S6 Descriptive reporting for females aged [15-79] in metropolitan France. A) Number of HR HPV tests, stratified by year and screening pathway. B) Number of tests, stratified by age, and screening pathway. C) Proportion of tests by result (positive for HPV16, positive for HPV18, positive for other genotypes, and negative), stratified by year and screening pathway. D) Proportion of tests by result, stratified by age and screening pathway.

Table S1 Number of tests and observed cervical infection prevalence, stratified by year and screening pathway. Table counterpart to Supplementary Figure S6A and C.

				Results (% of tests)			
				Positive			
Screening pathway	Year	Number of tests	% of total test	HPV16	HPV18	Other genotypes	Negative
All	All	369,695		2.78	1.04	9.26	88.16
All	2020	71,853	19.44	2.38	0.82	9.42	88.22
All	2021	162,515	43.96	2.73	1.02	8.75	88.73
All	2022	73,727	19.94	2.90	1.12	9.12	88.30
All	2023	61,600	16.66	3.21	1.24	10.55	86.46
Opportunistic	All	349,729		2.81	1.06	9.38	88.01
Opportunistic	2020	71,809	20.53	2.38	0.82	9.42	88.22
Opportunistic	2021	157,898	45.15	2.75	1.03	8.83	88.64
Opportunistic	2022	65,988	18.87	2.98	1.17	9.33	88.02
Opportunistic	2023	54,034	15.45	3.36	1.30	11.04	85.86
Organised	All	19,966		2.20	0.77	6.99	90.92
Organised	2020	44	0.22	6.82	0.00	11.36	84.09
Organised	2021	4,617	23.12	2.34	0.69	6.26	91.75
Organised	2022	7,739	38.76	2.16	0.72	7.30	90.63
Organised	2023	7,566	37.89	2.14	0.86	7.08	90.76

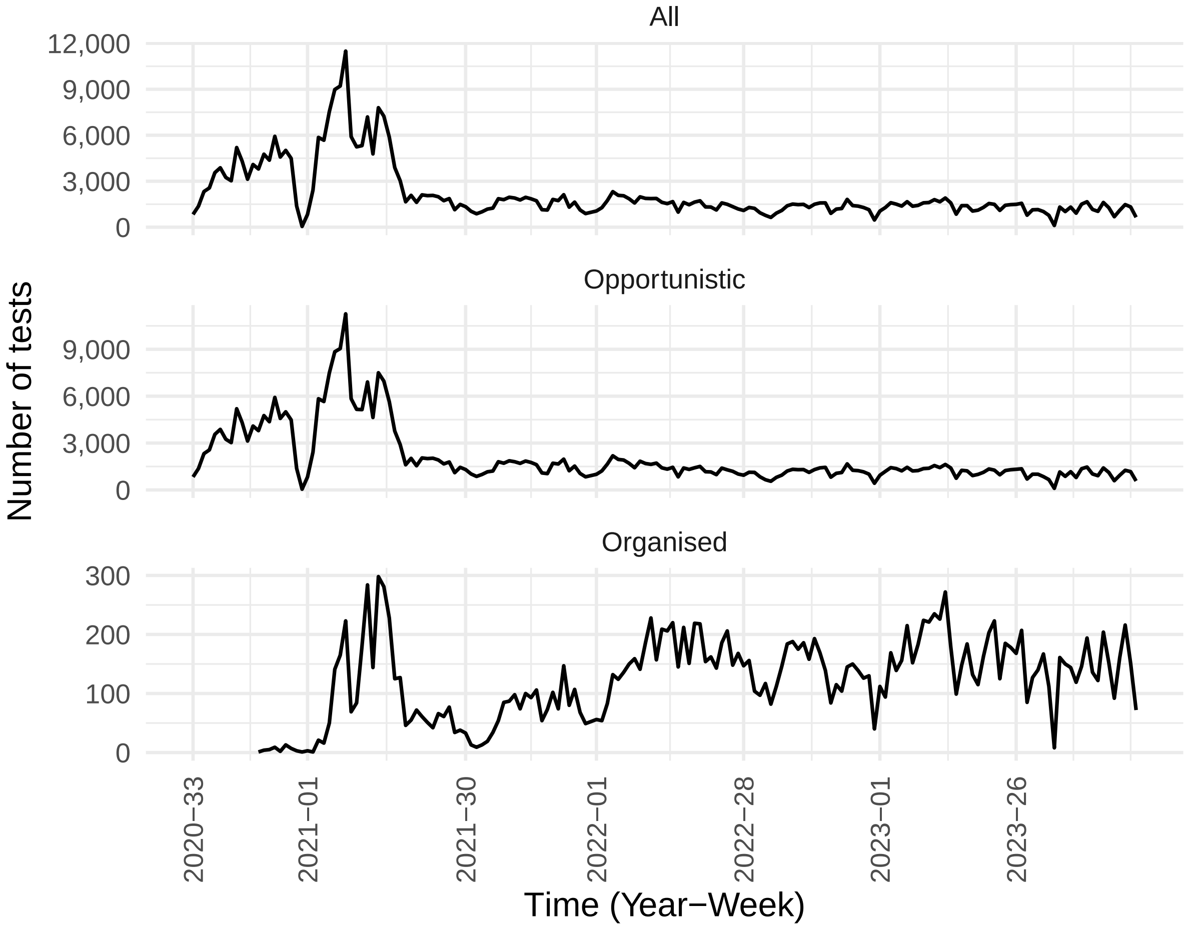

Figure S7 **Number of tests performed in each time step (year-week), stratified by screening pathway.**

Table S2 Proportion of tests with coinfection, stratified by screening pathway and age.

Age	Screening pathway	Proportion in % (Order: HPV16, HPV18, Other genotypes)*								Number of tests
		000	001	010	100	011	101	110	111	
All	All	88.17	8.12	0.62	1.89	0.30	0.77	0.06	0.05	369,791
All	Organised	90.93	6.16	0.48	1.58	0.24	0.56	0.02	0.02	20,075
All	Opportunistic	88.02	8.24	0.63	1.91	0.31	0.78	0.06	0.05	349,716
(66,79]	All	91.20	5.65	0.41	1.79	0.24	0.69	0.02	0.00	5,078
(66,79]	Opportunistic	91.20	5.65	0.41	1.79	0.24	0.69	0.02	0.00	5,078
(59,66]	All	91.63	5.60	0.49	1.49	0.22	0.51	0.03	0.03	42,356
(59,66]	Organised	92.38	5.09	0.42	1.36	0.22	0.48	0.02	0.02	4,987
(59,66]	Opportunistic	91.53	5.67	0.50	1.51	0.22	0.51	0.03	0.03	37,369
(49,59]	All	90.79	6.30	0.57	1.45	0.25	0.55	0.04	0.04	83,824
(49,59]	Organised	92.28	5.29	0.44	1.21	0.21	0.48	0.04	0.04	5,197
(49,59]	Opportunistic	90.69	6.37	0.58	1.47	0.25	0.56	0.04	0.04	78,627
(39,49]	All	88.96	7.37	0.66	1.90	0.31	0.69	0.06	0.05	104,989
(39,49]	Organised	91.02	6.04	0.61	1.60	0.28	0.42	0.02	0.00	4,567
(39,49]	Opportunistic	88.86	7.43	0.66	1.92	0.31	0.70	0.07	0.05	100,422
(29,39]	All	84.83	10.65	0.68	2.30	0.36	1.05	0.07	0.07	131,794
(29,39]	Organised	88.55	7.79	0.46	2.05	0.23	0.85	0.02	0.04	5,170
(29,39]	Opportunistic	84.68	10.76	0.69	2.31	0.36	1.05	0.07	0.07	126,624
(24,29]	All	76.77	18.17	0.45	2.08	0.65	1.69	0.19	0.00	1,541
(24,29]	Organised	75.97	18.18	0.00	4.55	0.65	0.65	0.00	0.00	154
(24,29]	Opportunistic	76.86	18.17	0.50	1.80	0.65	1.80	0.22	0.00	1,387
[17,24]	All	67.94	27.75	0.00	2.39	0.48	0.96	0.00	0.48	209
[17,24]	Opportunistic	67.94	27.75	0.00	2.39	0.48	0.96	0.00	0.48	209

* *: Positive tests are flagged with 1 and negative tests by 0.

Table S3 Number of tests and observed infection prevalence, stratified by age and screening pathway. Table counterpart to Figure S6B and D.

Screening pathway	Age	Number of tests	% of total test	Results (% of tests)			
				Positive			
				HPV16	HPV18	Other genotypes	Negative
All	[17,24]	209	0.06	3.83	0.96	29.67	67.94
All	(24,29]	1,542	0.42	3.96	1.30	20.51	76.77
All	(29,39]	131,684	35.62	3.48	1.18	12.12	84.83
All	(39,49]	104,981	28.40	2.71	1.08	8.42	88.96
All	(49,59]	83,859	22.68	2.09	0.91	7.14	90.79
All	(59,66]	42,334	11.45	2.06	0.77	6.36	91.63
All	(66,79]	5,086	1.38	2.50	0.67	6.58	91.20
Opportunistic	[17,24]	209	0.06	3.83	0.96	29.67	67.94
Opportunistic	(24,29]	1,389	0.40	3.82	1.37	20.62	76.86
Opportunistic	(29,39]	126,549	36.18	3.51	1.20	12.25	84.68
Opportunistic	(39,49]	100,439	28.72	2.74	1.09	8.49	88.86
Opportunistic	(49,59]	78,691	22.50	2.11	0.92	7.22	90.69
Opportunistic	(59,66]	37,366	10.68	2.08	0.78	6.43	91.53
Opportunistic	(66,79]	5,086	1.45	2.50	0.67	6.58	91.20
Organised	(24,29]	153	0.77	5.19	0.65	19.48	75.97
Organised	(29,39]	5,135	25.72	2.96	0.75	8.92	88.55
Organised	(39,49]	4,542	22.75	2.04	0.92	6.74	91.02
Organised	(49,59]	5,168	25.88	1.77	0.73	6.02	92.28
Organised	(59,66]	4,968	24.88	1.88	0.68	5.82	92.38

S4.2 Descriptive reporting among females aged [15-79] in the overseas territories

The number of tests for people living in the overseas territories was `formating_text(sum(df_dom_tom$N),0)`, among which the share of tests collected through opportunistic screening was 99.0%. A complete reporting of the descriptive statistics is provided in the Supplementary Table S4.

Table S4 Number of tests and observed prevalence, stratified by genotype group, screening pathway, year, age, and district.

					Positive for...									
Variables					HPV16		HPV18		HPV16/18		HPV-other		At least one HR HPV	
Screening	Year	Age	District	Number of tests (N)	N	%	N	%	N	%	N	%	N	%
Opportunistic				2,988	67	2.2	50	1.7	116	3.9	411	13.8	500	16.7
Organised				29	1	3.4	1	3.4	2	6.9	2	6.9	4	13.8
	2020			1,296	26	2.0	25	1.9	51	3.9	182	14.0	222	17.1
	2021			1,654	37	2.2	25	1.5	61	3.7	218	13.2	265	16.0
	2022			35	3	8.6	0	0.0	3	8.6	9	25.7	10	28.6
	2023			32	2	6.2	1	3.1	3	9.4	4	12.5	7	21.9
		[24,25]		1	0	0.0	0	0.0	0	0.0	0	0.0	0	0.0
		(25,29]		3	1	33.3	0	0.0	1	33.3	2	66.7	2	66.7
		(29,39]		775	29	3.7	13	1.7	41	5.3	152	19.6	182	23.5
		(39,49]		1,043	17	1.6	18	1.7	35	3.4	123	11.8	154	14.8
		(49,59]		818	15	1.8	14	1.7	29	3.5	94	11.5	116	14.2
		(59,66]		339	6	1.8	3	0.9	9	2.7	36	10.6	43	12.7
		(66,76]		38	0	0.0	3	7.9	3	7.9	6	15.8	7	18.4
			Guadeloupe	1,775	41	2.3	31	1.7	71	4.0	241	13.6	298	16.8
			Guyane	26	0	0.0	0	0.0	0	0.0	2	7.7	2	7.7
			La Réunion	32	1	3.1	0	0.0	1	3.1	7	21.9	8	25.0
			Martinique	1,162	26	2.2	19	1.6	45	3.9	156	13.4	188	16.2
			Mayotte	21	0	0.0	1	4.8	1	4.8	6	28.6	7	33.3
				1	0	0.0	0	0.0	0	0.0	1	100.0	1	100.0
Opportunistic	2020			1,295	26	2.0	25	1.9	51	3.9	182	14.1	222	17.1
Opportunistic	2021			1,630	37	2.3	25	1.5	61	3.7	216	13.3	263	16.1
Opportunistic	2022			34	3	8.8	0	0.0	3	8.8	9	26.5	10	29.4
Opportunistic	2023			29	1	3.4	0	0.0	1	3.4	4	13.8	5	17.2

Table S4 **Number of tests and observed prevalence, stratified by genotype group, screening pathway, year, age, and district.** ***(continued)***

Screening	Year	Age	District	Number of tests (N)	N	%	N	%	N	%	N	%	N	%
Organised	2020			1	0	0.0	0	0.0	0	0.0	0	0.0	0	0.0
Organised	2021			24	0	0.0	0	0.0	0	0.0	2	8.3	2	8.3
Organised	2022			1	0	0.0	0	0.0	0	0.0	0	0.0	0	0.0
Organised	2023			3	1	33.3	1	33.3	2	66.7	0	0.0	2	66.7
Opportunistic		(25,29]		2	1	50.0	0	0.0	1	50.0	2	100.0	2	100.0
Opportunistic		(29,39]		773	29	3.8	13	1.7	41	5.3	152	19.7	182	23.5
Opportunistic		(39,49]		1,041	17	1.6	17	1.6	34	3.3	122	11.7	152	14.6
Opportunistic		(49,59]		807	15	1.9	14	1.7	29	3.6	94	11.6	116	14.4
Opportunistic		(59,66]		327	5	1.5	3	0.9	8	2.4	35	10.7	41	12.5
Opportunistic		(66,76]		38	0	0.0	3	7.9	3	7.9	6	15.8	7	18.4
Organised		[24,25]		1	0	0.0	0	0.0	0	0.0	0	0.0	0	0.0
Organised		(25,29]		1	0	0.0	0	0.0	0	0.0	0	0.0	0	0.0
Organised		(29,39]		2	0	0.0	0	0.0	0	0.0	0	0.0	0	0.0
Organised		(39,49]		2	0	0.0	1	50.0	1	50.0	1	50.0	2	100.0
Organised		(49,59]		11	0	0.0	0	0.0	0	0.0	0	0.0	0	0.0
Organised		(59,66]		12	1	8.3	0	0.0	1	8.3	1	8.3	2	16.7
Opportunistic			Guadeloupe	1,773	40	2.3	31	1.7	70	3.9	241	13.6	297	16.8
Opportunistic			Guyane	25	0	0.0	0	0.0	0	0.0	2	8.0	2	8.0
Opportunistic			La Réunion	31	1	3.2	0	0.0	1	3.2	7	22.6	8	25.8
Opportunistic			Martinique	1,139	26	2.3	19	1.7	45	4.0	155	13.6	187	16.4
Opportunistic			Mayotte	19	0	0.0	0	0.0	0	0.0	5	26.3	5	26.3
Opportunistic				1	0	0.0	0	0.0	0	0.0	1	100.0	1	100.0
Organised			Guadeloupe	2	1	50.0	0	0.0	1	50.0	0	0.0	1	50.0
Organised			Guyane	1	0	0.0	0	0.0	0	0.0	0	0.0	0	0.0
Organised			La Réunion	1	0	0.0	0	0.0	0	0.0	0	0.0	0	0.0
Organised			Martinique	23	0	0.0	0	0.0	0	0.0	1	4.3	1	4.3
Organised			Mayotte	2	0	0.0	1	50.0	1	50.0	1	50.0	2	100.0
	2020	(25,29]		2	1	50.0	0	0.0	1	50.0	2	100.0	2	100.0
	2020	(29,39]		346	10	2.9	6	1.7	16	4.6	63	18.2	75	21.7
	2020	(39,49]		447	5	1.1	9	2.0	14	3.1	58	13.0	71	15.9
	2020	(49,59]		385	10	2.6	8	2.1	18	4.7	51	13.2	64	16.6

Table S4 **Number of tests and observed prevalence, stratified by genotype group, screening pathway, year, age, and district.** ***(continued)***

Screening	Year	Age	District	Number of tests (N)	N	%	N	%	N	%	N	%	N	%
	2020	(59,66]		112	0	0.0	2	1.8	2	1.8	7	6.2	9	8.0
	2020	(66,76]		4	0	0.0	0	0.0	0	0.0	1	25.0	1	25.0
	2021	[24,25]		1	0	0.0	0	0.0	0	0.0	0	0.0	0	0.0
	2021	(25,29]		1	0	0.0	0	0.0	0	0.0	0	0.0	0	0.0
	2021	(29,39]		408	17	4.2	7	1.7	23	5.6	83	20.3	100	24.5
	2021	(39,49]		582	11	1.9	8	1.4	19	3.3	63	10.8	79	13.6
	2021	(49,59]		418	4	1.0	6	1.4	10	2.4	39	9.3	48	11.5
	2021	(59,66]		212	5	2.4	1	0.5	6	2.8	28	13.2	32	15.1
	2021	(66,76]		32	0	0.0	3	9.4	3	9.4	5	15.6	6	18.8
	2022	(29,39]		15	2	13.3	0	0.0	2	13.3	5	33.3	6	40.0
	2022	(39,49]		6	0	0.0	0	0.0	0	0.0	2	33.3	2	33.3
	2022	(49,59]		6	1	16.7	0	0.0	1	16.7	2	33.3	2	33.3
	2022	(59,66]		6	0	0.0	0	0.0	0	0.0	0	0.0	0	0.0
	2022	(66,76]		2	0	0.0	0	0.0	0	0.0	0	0.0	0	0.0
	2023	(29,39]		6	0	0.0	0	0.0	0	0.0	1	16.7	1	16.7
	2023	(39,49]		8	1	12.5	1	12.5	2	25.0	0	0.0	2	25.0
	2023	(49,59]		9	0	0.0	0	0.0	0	0.0	2	22.2	2	22.2
	2023	(59,66]		9	1	11.1	0	0.0	1	11.1	1	11.1	2	22.2
	2020		Guadeloupe	759	15	2.0	15	2.0	30	4.0	108	14.2	133	17.5
	2020		Guyane	7	0	0.0	0	0.0	0	0.0	1	14.3	1	14.3
	2020		La Réunion	11	0	0.0	0	0.0	0	0.0	4	36.4	4	36.4
	2020		Martinique	515	11	2.1	10	1.9	21	4.1	69	13.4	84	16.3
	2020		Mayotte	4	0	0.0	0	0.0	0	0.0	0	0.0	0	0.0
	2021		Guadeloupe	988	23	2.3	16	1.6	38	3.8	127	12.9	157	15.9
	2021		Guyane	12	0	0.0	0	0.0	0	0.0	0	0.0	0	0.0
	2021		La Réunion	13	1	7.7	0	0.0	1	7.7	3	23.1	4	30.8
	2021		Martinique	627	13	2.1	9	1.4	22	3.5	83	13.2	99	15.8
	2021		Mayotte	13	0	0.0	0	0.0	0	0.0	4	30.8	4	30.8
	2021			1	0	0.0	0	0.0	0	0.0	1	100.0	1	100.0
	2022		Guadeloupe	17	2	11.8	0	0.0	2	11.8	4	23.5	5	29.4
	2022		Guyane	3	0	0.0	0	0.0	0	0.0	1	33.3	1	33.3

Table S4 **Number of tests and observed prevalence, stratified by genotype group, screening pathway, year, age, and district.** ***(continued)***

Screening	Year	Age	District	Number of tests (N)	N	%	N	%	N	%	N	%	N	%
	2022		La Réunion	5	0	0.0	0	0.0	0	0.0	0	0.0	0	0.0
	2022		Martinique	8	1	12.5	0	0.0	1	12.5	2	25.0	2	25.0
	2022		Mayotte	2	0	0.0	0	0.0	0	0.0	2	100.0	2	100.0
	2023		Guadeloupe	11	1	9.1	0	0.0	1	9.1	2	18.2	3	27.3
	2023		Guyane	4	0	0.0	0	0.0	0	0.0	0	0.0	0	0.0
	2023		La Réunion	3	0	0.0	0	0.0	0	0.0	0	0.0	0	0.0
	2023		Martinique	12	1	8.3	0	0.0	1	8.3	2	16.7	3	25.0
	2023		Mayotte	2	0	0.0	1	50.0	1	50.0	0	0.0	1	50.0
		[24,25]	Martinique	1	0	0.0	0	0.0	0	0.0	0	0.0	0	0.0
		(25,29]	Guadeloupe	1	1	100.0	0	0.0	1	100.0	1	100.0	1	100.0
		(25,29]	Martinique	2	0	0.0	0	0.0	0	0.0	1	50.0	1	50.0
		(29,39]	Guadeloupe	448	18	4.0	6	1.3	23	5.1	88	19.6	106	23.7
		(29,39]	Guyane	8	0	0.0	0	0.0	0	0.0	0	0.0	0	0.0
		(29,39]	La Réunion	13	0	0.0	0	0.0	0	0.0	4	30.8	4	30.8
		(29,39]	Martinique	299	11	3.7	7	2.3	18	6.0	57	19.1	69	23.1
		(29,39]	Mayotte	7	0	0.0	0	0.0	0	0.0	3	42.9	3	42.9
		(39,49]	Guadeloupe	658	10	1.5	13	2.0	23	3.5	72	10.9	93	14.1
		(39,49]	Guyane	8	0	0.0	0	0.0	0	0.0	0	0.0	0	0.0
		(39,49]	La Réunion	12	1	8.3	0	0.0	1	8.3	3	25.0	4	33.3
		(39,49]	Martinique	353	6	1.7	4	1.1	10	2.8	44	12.5	52	14.7
		(39,49]	Mayotte	11	0	0.0	1	9.1	1	9.1	3	27.3	4	36.4
		(39,49]		1	0	0.0	0	0.0	0	0.0	1	100.0	1	100.0
		(49,59]	Guadeloupe	457	8	1.8	8	1.8	16	3.5	60	13.1	72	15.8
		(49,59]	Guyane	6	0	0.0	0	0.0	0	0.0	2	33.3	2	33.3
		(49,59]	La Réunion	4	0	0.0	0	0.0	0	0.0	0	0.0	0	0.0
		(49,59]	Martinique	349	7	2.0	6	1.7	13	3.7	32	9.2	42	12.0
		(49,59]	Mayotte	2	0	0.0	0	0.0	0	0.0	0	0.0	0	0.0
		(59,66]	Guadeloupe	190	4	2.1	2	1.1	6	3.2	18	9.5	23	12.1
		(59,66]	Guyane	4	0	0.0	0	0.0	0	0.0	0	0.0	0	0.0
		(59,66]	La Réunion	3	0	0.0	0	0.0	0	0.0	0	0.0	0	0.0
		(59,66]	Martinique	141	2	1.4	1	0.7	3	2.1	18	12.8	20	14.2

Table S4 **Number of tests and observed prevalence, stratified by genotype group, screening pathway, year, age, and district.** ***(continued)***

Screening	Year	Age	District	Number of tests (N)	N	%	N	%	N	%	N	%	N	%
		(59,66]	Mayotte	1	0	0.0	0	0.0	0	0.0	0	0.0	0	0.0
		(66,76]	Guadeloupe	21	0	0.0	2	9.5	2	9.5	2	9.5	3	14.3
		(66,76]	Martinique	17	0	0.0	1	5.9	1	5.9	4	23.5	4	23.5
Opportunistic	2020	(25,29]		2	1	50.0	0	0.0	1	50.0	2	100.0	2	100.0
Opportunistic	2020	(29,39]		346	10	2.9	6	1.7	16	4.6	63	18.2	75	21.7
Opportunistic	2020	(39,49]		447	5	1.1	9	2.0	14	3.1	58	13.0	71	15.9
Opportunistic	2020	(49,59]		385	10	2.6	8	2.1	18	4.7	51	13.2	64	16.6
Opportunistic	2020	(59,66]		111	0	0.0	2	1.8	2	1.8	7	6.3	9	8.1
Opportunistic	2020	(66,76]		4	0	0.0	0	0.0	0	0.0	1	25.0	1	25.0
Opportunistic	2021	(29,39]		407	17	4.2	7	1.7	23	5.7	83	20.4	100	24.6
Opportunistic	2021	(39,49]		581	11	1.9	8	1.4	19	3.3	62	10.7	78	13.4
Opportunistic	2021	(49,59]		408	4	1.0	6	1.5	10	2.5	39	9.6	48	11.8
Opportunistic	2021	(59,66]		202	5	2.5	1	0.5	6	3.0	27	13.4	31	15.3
Opportunistic	2021	(66,76]		32	0	0.0	3	9.4	3	9.4	5	15.6	6	18.8
Opportunistic	2022	(29,39]		14	2	14.3	0	0.0	2	14.3	5	35.7	6	42.9
Opportunistic	2022	(39,49]		6	0	0.0	0	0.0	0	0.0	2	33.3	2	33.3
Opportunistic	2022	(49,59]		6	1	16.7	0	0.0	1	16.7	2	33.3	2	33.3
Opportunistic	2022	(59,66]		6	0	0.0	0	0.0	0	0.0	0	0.0	0	0.0
Opportunistic	2022	(66,76]		2	0	0.0	0	0.0	0	0.0	0	0.0	0	0.0
Opportunistic	2023	(29,39]		6	0	0.0	0	0.0	0	0.0	1	16.7	1	16.7
Opportunistic	2023	(39,49]		7	1	14.3	0	0.0	1	14.3	0	0.0	1	14.3
Opportunistic	2023	(49,59]		8	0	0.0	0	0.0	0	0.0	2	25.0	2	25.0
Opportunistic	2023	(59,66]		8	0	0.0	0	0.0	0	0.0	1	12.5	1	12.5
Organised	2020	(59,66]		1	0	0.0	0	0.0	0	0.0	0	0.0	0	0.0
Organised	2021	[24,25]		1	0	0.0	0	0.0	0	0.0	0	0.0	0	0.0
Organised	2021	(25,29]		1	0	0.0	0	0.0	0	0.0	0	0.0	0	0.0
Organised	2021	(29,39]		1	0	0.0	0	0.0	0	0.0	0	0.0	0	0.0
Organised	2021	(39,49]		1	0	0.0	0	0.0	0	0.0	1	100.0	1	100.0
Organised	2021	(49,59]		10	0	0.0	0	0.0	0	0.0	0	0.0	0	0.0
Organised	2021	(59,66]		10	0	0.0	0	0.0	0	0.0	1	10.0	1	10.0
Organised	2022	(29,39]		1	0	0.0	0	0.0	0	0.0	0	0.0	0	0.0

Table S4 **Number of tests and observed prevalence, stratified by genotype group, screening pathway, year, age, and district.** ***(continued)***

Screening	Year	Age	District	Number of tests (N)	N	%	N	%	N	%	N	%	N	%
Organised	2023	(39,49]		1	0	0.0	1	100.0	1	100.0	0	0.0	1	100.0
Organised	2023	(49,59]		1	0	0.0	0	0.0	0	0.0	0	0.0	0	0.0
Organised	2023	(59,66]		1	1	100.0	0	0.0	1	100.0	0	0.0	1	100.0
Opportunistic	2020		Guadeloupe	759	15	2.0	15	2.0	30	4.0	108	14.2	133	17.5
Opportunistic	2020		Guyane	7	0	0.0	0	0.0	0	0.0	1	14.3	1	14.3
Opportunistic	2020		La Réunion	11	0	0.0	0	0.0	0	0.0	4	36.4	4	36.4
Opportunistic	2020		Martinique	514	11	2.1	10	1.9	21	4.1	69	13.4	84	16.3
Opportunistic	2020		Mayotte	4	0	0.0	0	0.0	0	0.0	0	0.0	0	0.0
Opportunistic	2021		Guadeloupe	987	23	2.3	16	1.6	38	3.9	127	12.9	157	15.9
Opportunistic	2021		Guyane	12	0	0.0	0	0.0	0	0.0	0	0.0	0	0.0
Opportunistic	2021		La Réunion	13	1	7.7	0	0.0	1	7.7	3	23.1	4	30.8
Opportunistic	2021		Martinique	605	13	2.1	9	1.5	22	3.6	82	13.6	98	16.2
Opportunistic	2021		Mayotte	12	0	0.0	0	0.0	0	0.0	3	25.0	3	25.0
Opportunistic	2021			1	0	0.0	0	0.0	0	0.0	1	100.0	1	100.0
Opportunistic	2022		Guadeloupe	17	2	11.8	0	0.0	2	11.8	4	23.5	5	29.4
Opportunistic	2022		Guyane	3	0	0.0	0	0.0	0	0.0	1	33.3	1	33.3
Opportunistic	2022		La Réunion	4	0	0.0	0	0.0	0	0.0	0	0.0	0	0.0
Opportunistic	2022		Martinique	8	1	12.5	0	0.0	1	12.5	2	25.0	2	25.0
Opportunistic	2022		Mayotte	2	0	0.0	0	0.0	0	0.0	2	100.0	2	100.0
Opportunistic	2023		Guadeloupe	10	0	0.0	0	0.0	0	0.0	2	20.0	2	20.0
Opportunistic	2023		Guyane	3	0	0.0	0	0.0	0	0.0	0	0.0	0	0.0
Opportunistic	2023		La Réunion	3	0	0.0	0	0.0	0	0.0	0	0.0	0	0.0
Opportunistic	2023		Martinique	12	1	8.3	0	0.0	1	8.3	2	16.7	3	25.0
Opportunistic	2023		Mayotte	1	0	0.0	0	0.0	0	0.0	0	0.0	0	0.0
Organised	2020		Martinique	1	0	0.0	0	0.0	0	0.0	0	0.0	0	0.0
Organised	2021		Guadeloupe	1	0	0.0	0	0.0	0	0.0	0	0.0	0	0.0
Organised	2021		Martinique	22	0	0.0	0	0.0	0	0.0	1	4.5	1	4.5
Organised	2021		Mayotte	1	0	0.0	0	0.0	0	0.0	1	100.0	1	100.0
Organised	2022		La Réunion	1	0	0.0	0	0.0	0	0.0	0	0.0	0	0.0
Organised	2023		Guadeloupe	1	1	100.0	0	0.0	1	100.0	0	0.0	1	100.0
Organised	2023		Guyane	1	0	0.0	0	0.0	0	0.0	0	0.0	0	0.0

Table S4 **Number of tests and observed prevalence, stratified by genotype group, screening pathway, year, age, and district.** ***(continued)***

Screening	Year	Age	District	Number of tests (N)	N	%	N	%	N	%	N	%	N	%
Organised	2023		Mayotte	1	0	0.0	1	100.0	1	100.0	0	0.0	1	100.0
Opportunistic		(25,29]	Guadeloupe	1	1	100.0	0	0.0	1	100.0	1	100.0	1	100.0
Opportunistic		(25,29]	Martinique	1	0	0.0	0	0.0	0	0.0	1	100.0	1	100.0
Opportunistic		(29,39]	Guadeloupe	448	18	4.0	6	1.3	23	5.1	88	19.6	106	23.7
Opportunistic		(29,39]	Guyane	8	0	0.0	0	0.0	0	0.0	0	0.0	0	0.0
Opportunistic		(29,39]	La Réunion	12	0	0.0	0	0.0	0	0.0	4	33.3	4	33.3
Opportunistic		(29,39]	Martinique	298	11	3.7	7	2.3	18	6.0	57	19.1	69	23.2
Opportunistic		(29,39]	Mayotte	7	0	0.0	0	0.0	0	0.0	3	42.9	3	42.9
Opportunistic		(39,49]	Guadeloupe	658	10	1.5	13	2.0	23	3.5	72	10.9	93	14.1
Opportunistic		(39,49]	Guyane	8	0	0.0	0	0.0	0	0.0	0	0.0	0	0.0
Opportunistic		(39,49]	La Réunion	12	1	8.3	0	0.0	1	8.3	3	25.0	4	33.3
Opportunistic		(39,49]	Martinique	353	6	1.7	4	1.1	10	2.8	44	12.5	52	14.7
Opportunistic		(39,49]	Mayotte	9	0	0.0	0	0.0	0	0.0	2	22.2	2	22.2
Opportunistic		(39,49]		1	0	0.0	0	0.0	0	0.0	1	100.0	1	100.0
Opportunistic		(49,59]	Guadeloupe	457	8	1.8	8	1.8	16	3.5	60	13.1	72	15.8
Opportunistic		(49,59]	Guyane	5	0	0.0	0	0.0	0	0.0	2	40.0	2	40.0
Opportunistic		(49,59]	La Réunion	4	0	0.0	0	0.0	0	0.0	0	0.0	0	0.0
Opportunistic		(49,59]	Martinique	339	7	2.1	6	1.8	13	3.8	32	9.4	42	12.4
Opportunistic		(49,59]	Mayotte	2	0	0.0	0	0.0	0	0.0	0	0.0	0	0.0
Opportunistic		(59,66]	Guadeloupe	188	3	1.6	2	1.1	5	2.7	18	9.6	22	11.7
Opportunistic		(59,66]	Guyane	4	0	0.0	0	0.0	0	0.0	0	0.0	0	0.0
Opportunistic		(59,66]	La Réunion	3	0	0.0	0	0.0	0	0.0	0	0.0	0	0.0
Opportunistic		(59,66]	Martinique	131	2	1.5	1	0.8	3	2.3	17	13.0	19	14.5
Opportunistic		(59,66]	Mayotte	1	0	0.0	0	0.0	0	0.0	0	0.0	0	0.0
Opportunistic		(66,76]	Guadeloupe	21	0	0.0	2	9.5	2	9.5	2	9.5	3	14.3
Opportunistic		(66,76]	Martinique	17	0	0.0	1	5.9	1	5.9	4	23.5	4	23.5
Organised		[24,25]	Martinique	1	0	0.0	0	0.0	0	0.0	0	0.0	0	0.0
Organised		(25,29]	Martinique	1	0	0.0	0	0.0	0	0.0	0	0.0	0	0.0
Organised		(29,39]	La Réunion	1	0	0.0	0	0.0	0	0.0	0	0.0	0	0.0
Organised		(29,39]	Martinique	1	0	0.0	0	0.0	0	0.0	0	0.0	0	0.0
Organised		(39,49]	Mayotte	2	0	0.0	1	50.0	1	50.0	1	50.0	2	100.0

Table S4 **Number of tests and observed prevalence, stratified by genotype group, screening pathway, year, age, and district.** ***(continued)***

Screening	Year	Age	District	Number of tests (N)	N	%	N	%	N	%	N	%	N	%
Organised		(49,59]	Guyane	1	0	0.0	0	0.0	0	0.0	0	0.0	0	0.0
Organised		(49,59]	Martinique	10	0	0.0	0	0.0	0	0.0	0	0.0	0	0.0
Organised		(59,66]	Guadeloupe	2	1	50.0	0	0.0	1	50.0	0	0.0	1	50.0
Organised		(59,66]	Martinique	10	0	0.0	0	0.0	0	0.0	1	10.0	1	10.0
	2020	(25,29]	Guadeloupe	1	1	100.0	0	0.0	1	100.0	1	100.0	1	100.0
	2020	(25,29]	Martinique	1	0	0.0	0	0.0	0	0.0	1	100.0	1	100.0
	2020	(29,39]	Guadeloupe	210	6	2.9	3	1.4	9	4.3	36	17.1	43	20.5
	2020	(29,39]	Guyane	4	0	0.0	0	0.0	0	0.0	0	0.0	0	0.0
	2020	(29,39]	La Réunion	4	0	0.0	0	0.0	0	0.0	2	50.0	2	50.0
	2020	(29,39]	Martinique	127	4	3.1	3	2.4	7	5.5	25	19.7	30	23.6
	2020	(29,39]	Mayotte	1	0	0.0	0	0.0	0	0.0	0	0.0	0	0.0
	2020	(39,49]	Guadeloupe	288	4	1.4	7	2.4	11	3.8	39	13.5	50	17.4
	2020	(39,49]	La Réunion	5	0	0.0	0	0.0	0	0.0	2	40.0	2	40.0
	2020	(39,49]	Martinique	152	1	0.7	2	1.3	3	2.0	17	11.2	19	12.5
	2020	(39,49]	Mayotte	2	0	0.0	0	0.0	0	0.0	0	0.0	0	0.0
	2020	(49,59]	Guadeloupe	207	4	1.9	4	1.9	8	3.9	29	14.0	35	16.9
	2020	(49,59]	Guyane	2	0	0.0	0	0.0	0	0.0	1	50.0	1	50.0
	2020	(49,59]	La Réunion	1	0	0.0	0	0.0	0	0.0	0	0.0	0	0.0
	2020	(49,59]	Martinique	175	6	3.4	4	2.3	10	5.7	21	12.0	28	16.0
	2020	(59,66]	Guadeloupe	52	0	0.0	1	1.9	1	1.9	3	5.8	4	7.7
	2020	(59,66]	Guyane	1	0	0.0	0	0.0	0	0.0	0	0.0	0	0.0
	2020	(59,66]	La Réunion	1	0	0.0	0	0.0	0	0.0	0	0.0	0	0.0
	2020	(59,66]	Martinique	57	0	0.0	1	1.8	1	1.8	4	7.0	5	8.8
	2020	(59,66]	Mayotte	1	0	0.0	0	0.0	0	0.0	0	0.0	0	0.0
	2020	(66,76]	Guadeloupe	1	0	0.0	0	0.0	0	0.0	0	0.0	0	0.0
	2020	(66,76]	Martinique	3	0	0.0	0	0.0	0	0.0	1	33.3	1	33.3
	2021	[24,25]	Martinique	1	0	0.0	0	0.0	0	0.0	0	0.0	0	0.0
	2021	(25,29]	Martinique	1	0	0.0	0	0.0	0	0.0	0	0.0	0	0.0
	2021	(29,39]	Guadeloupe	231	11	4.8	3	1.3	13	5.6	49	21.2	59	25.5
	2021	(29,39]	Guyane	2	0	0.0	0	0.0	0	0.0	0	0.0	0	0.0
	2021	(29,39]	La Réunion	4	0	0.0	0	0.0	0	0.0	2	50.0	2	50.0

Table S4 **Number of tests and observed prevalence, stratified by genotype group, screening pathway, year, age, and district.** ***(continued)***

Screening	Year	Age	District	Number of tests (N)	N	%	N	%	N	%	N	%	N	%
	2021	(29,39]	Martinique	166	6	3.6	4	2.4	10	6.0	30	18.1	37	22.3
	2021	(29,39]	Mayotte	5	0	0.0	0	0.0	0	0.0	2	40.0	2	40.0
	2021	(39,49]	Guadeloupe	367	6	1.6	6	1.6	12	3.3	33	9.0	43	11.7
	2021	(39,49]	Guyane	6	0	0.0	0	0.0	0	0.0	0	0.0	0	0.0
	2021	(39,49]	La Réunion	5	1	20.0	0	0.0	1	20.0	1	20.0	2	40.0
	2021	(39,49]	Martinique	196	4	2.0	2	1.0	6	3.1	26	13.3	31	15.8
	2021	(39,49]	Mayotte	7	0	0.0	0	0.0	0	0.0	2	28.6	2	28.6
	2021	(39,49]		1	0	0.0	0	0.0	0	0.0	1	100.0	1	100.0
	2021	(49,59]	Guadeloupe	242	3	1.2	4	1.7	7	2.9	29	12.0	35	14.5
	2021	(49,59]	Guyane	2	0	0.0	0	0.0	0	0.0	0	0.0	0	0.0
	2021	(49,59]	La Réunion	3	0	0.0	0	0.0	0	0.0	0	0.0	0	0.0
	2021	(49,59]	Martinique	170	1	0.6	2	1.2	3	1.8	10	5.9	13	7.6
	2021	(49,59]	Mayotte	1	0	0.0	0	0.0	0	0.0	0	0.0	0	0.0
	2021	(59,66]	Guadeloupe	129	3	2.3	1	0.8	4	3.1	14	10.9	17	13.2
	2021	(59,66]	Guyane	2	0	0.0	0	0.0	0	0.0	0	0.0	0	0.0
	2021	(59,66]	La Réunion	1	0	0.0	0	0.0	0	0.0	0	0.0	0	0.0
	2021	(59,66]	Martinique	80	2	2.5	0	0.0	2	2.5	14	17.5	15	18.8
	2021	(66,76]	Guadeloupe	19	0	0.0	2	10.5	2	10.5	2	10.5	3	15.8
	2021	(66,76]	Martinique	13	0	0.0	1	7.7	1	7.7	3	23.1	3	23.1
	2022	(29,39]	Guadeloupe	6	1	16.7	0	0.0	1	16.7	3	50.0	4	66.7
	2022	(29,39]	Guyane	1	0	0.0	0	0.0	0	0.0	0	0.0	0	0.0
	2022	(29,39]	La Réunion	3	0	0.0	0	0.0	0	0.0	0	0.0	0	0.0
	2022	(29,39]	Martinique	4	1	25.0	0	0.0	1	25.0	1	25.0	1	25.0
	2022	(29,39]	Mayotte	1	0	0.0	0	0.0	0	0.0	1	100.0	1	100.0
	2022	(39,49]	Guadeloupe	1	0	0.0	0	0.0	0	0.0	0	0.0	0	0.0
	2022	(39,49]	Guyane	1	0	0.0	0	0.0	0	0.0	0	0.0	0	0.0
	2022	(39,49]	La Réunion	2	0	0.0	0	0.0	0	0.0	0	0.0	0	0.0
	2022	(39,49]	Martinique	1	0	0.0	0	0.0	0	0.0	1	100.0	1	100.0
	2022	(39,49]	Mayotte	1	0	0.0	0	0.0	0	0.0	1	100.0	1	100.0
	2022	(49,59]	Guadeloupe	5	1	20.0	0	0.0	1	20.0	1	20.0	1	20.0
	2022	(49,59]	Guyane	1	0	0.0	0	0.0	0	0.0	1	100.0	1	100.0

Table S4 **Number of tests and observed prevalence, stratified by genotype group, screening pathway, year, age, and district.** ***(continued)***

Screening	Year	Age	District	Number of tests (N)	N	%	N	%	N	%	N	%	N	%
	2022	(59,66]	Guadeloupe	4	0	0.0	0	0.0	0	0.0	0	0.0	0	0.0
	2022	(59,66]	Martinique	2	0	0.0	0	0.0	0	0.0	0	0.0	0	0.0
	2022	(66,76]	Guadeloupe	1	0	0.0	0	0.0	0	0.0	0	0.0	0	0.0
	2022	(66,76]	Martinique	1	0	0.0	0	0.0	0	0.0	0	0.0	0	0.0
	2023	(29,39]	Guadeloupe	1	0	0.0	0	0.0	0	0.0	0	0.0	0	0.0
	2023	(29,39]	Guyane	1	0	0.0	0	0.0	0	0.0	0	0.0	0	0.0
	2023	(29,39]	La Réunion	2	0	0.0	0	0.0	0	0.0	0	0.0	0	0.0
	2023	(29,39]	Martinique	2	0	0.0	0	0.0	0	0.0	1	50.0	1	50.0
	2023	(39,49]	Guadeloupe	2	0	0.0	0	0.0	0	0.0	0	0.0	0	0.0
	2023	(39,49]	Guyane	1	0	0.0	0	0.0	0	0.0	0	0.0	0	0.0
	2023	(39,49]	Martinique	4	1	25.0	0	0.0	1	25.0	0	0.0	1	25.0
	2023	(39,49]	Mayotte	1	0	0.0	1	100.0	1	100.0	0	0.0	1	100.0
	2023	(49,59]	Guadeloupe	3	0	0.0	0	0.0	0	0.0	1	33.3	1	33.3
	2023	(49,59]	Guyane	1	0	0.0	0	0.0	0	0.0	0	0.0	0	0.0
	2023	(49,59]	Martinique	4	0	0.0	0	0.0	0	0.0	1	25.0	1	25.0
	2023	(49,59]	Mayotte	1	0	0.0	0	0.0	0	0.0	0	0.0	0	0.0
	2023	(59,66]	Guadeloupe	5	1	20.0	0	0.0	1	20.0	1	20.0	2	40.0
	2023	(59,66]	Guyane	1	0	0.0	0	0.0	0	0.0	0	0.0	0	0.0
	2023	(59,66]	La Réunion	1	0	0.0	0	0.0	0	0.0	0	0.0	0	0.0
	2023	(59,66]	Martinique	2	0	0.0	0	0.0	0	0.0	0	0.0	0	0.0
Opportunistic	2020	(25,29]	Guadeloupe	1	1	100.0	0	0.0	1	100.0	1	100.0	1	100.0
Opportunistic	2020	(25,29]	Martinique	1	0	0.0	0	0.0	0	0.0	1	100.0	1	100.0
Opportunistic	2020	(29,39]	Guadeloupe	210	6	2.9	3	1.4	9	4.3	36	17.1	43	20.5
Opportunistic	2020	(29,39]	Guyane	4	0	0.0	0	0.0	0	0.0	0	0.0	0	0.0
Opportunistic	2020	(29,39]	La Réunion	4	0	0.0	0	0.0	0	0.0	2	50.0	2	50.0
Opportunistic	2020	(29,39]	Martinique	127	4	3.1	3	2.4	7	5.5	25	19.7	30	23.6
Opportunistic	2020	(29,39]	Mayotte	1	0	0.0	0	0.0	0	0.0	0	0.0	0	0.0
Opportunistic	2020	(39,49]	Guadeloupe	288	4	1.4	7	2.4	11	3.8	39	13.5	50	17.4
Opportunistic	2020	(39,49]	La Réunion	5	0	0.0	0	0.0	0	0.0	2	40.0	2	40.0
Opportunistic	2020	(39,49]	Martinique	152	1	0.7	2	1.3	3	2.0	17	11.2	19	12.5
Opportunistic	2020	(39,49]	Mayotte	2	0	0.0	0	0.0	0	0.0	0	0.0	0	0.0

Table S4 **Number of tests and observed prevalence, stratified by genotype group, screening pathway, year, age, and district.** ***(continued)***

Screening	Year	Age	District	Number of tests (N)	N	%	N	%	N	%	N	%	N	%
Opportunistic	2020	(49,59]	Guadeloupe	207	4	1.9	4	1.9	8	3.9	29	14.0	35	16.9
Opportunistic	2020	(49,59]	Guyane	2	0	0.0	0	0.0	0	0.0	1	50.0	1	50.0
Opportunistic	2020	(49,59]	La Réunion	1	0	0.0	0	0.0	0	0.0	0	0.0	0	0.0
Opportunistic	2020	(49,59]	Martinique	175	6	3.4	4	2.3	10	5.7	21	12.0	28	16.0
Opportunistic	2020	(59,66]	Guadeloupe	52	0	0.0	1	1.9	1	1.9	3	5.8	4	7.7
Opportunistic	2020	(59,66]	Guyane	1	0	0.0	0	0.0	0	0.0	0	0.0	0	0.0
Opportunistic	2020	(59,66]	La Réunion	1	0	0.0	0	0.0	0	0.0	0	0.0	0	0.0
Opportunistic	2020	(59,66]	Martinique	56	0	0.0	1	1.8	1	1.8	4	7.1	5	8.9
Opportunistic	2020	(59,66]	Mayotte	1	0	0.0	0	0.0	0	0.0	0	0.0	0	0.0
Opportunistic	2020	(66,76]	Guadeloupe	1	0	0.0	0	0.0	0	0.0	0	0.0	0	0.0
Opportunistic	2020	(66,76]	Martinique	3	0	0.0	0	0.0	0	0.0	1	33.3	1	33.3
Opportunistic	2021	(29,39]	Guadeloupe	231	11	4.8	3	1.3	13	5.6	49	21.2	59	25.5
Opportunistic	2021	(29,39]	Guyane	2	0	0.0	0	0.0	0	0.0	0	0.0	0	0.0
Opportunistic	2021	(29,39]	La Réunion	4	0	0.0	0	0.0	0	0.0	2	50.0	2	50.0
Opportunistic	2021	(29,39]	Martinique	165	6	3.6	4	2.4	10	6.1	30	18.2	37	22.4
Opportunistic	2021	(29,39]	Mayotte	5	0	0.0	0	0.0	0	0.0	2	40.0	2	40.0
Opportunistic	2021	(39,49]	Guadeloupe	367	6	1.6	6	1.6	12	3.3	33	9.0	43	11.7
Opportunistic	2021	(39,49]	Guyane	6	0	0.0	0	0.0	0	0.0	0	0.0	0	0.0
Opportunistic	2021	(39,49]	La Réunion	5	1	20.0	0	0.0	1	20.0	1	20.0	2	40.0
Opportunistic	2021	(39,49]	Martinique	196	4	2.0	2	1.0	6	3.1	26	13.3	31	15.8
Opportunistic	2021	(39,49]	Mayotte	6	0	0.0	0	0.0	0	0.0	1	16.7	1	16.7
Opportunistic	2021	(39,49]		1	0	0.0	0	0.0	0	0.0	1	100.0	1	100.0
Opportunistic	2021	(49,59]	Guadeloupe	242	3	1.2	4	1.7	7	2.9	29	12.0	35	14.5
Opportunistic	2021	(49,59]	Guyane	2	0	0.0	0	0.0	0	0.0	0	0.0	0	0.0
Opportunistic	2021	(49,59]	La Réunion	3	0	0.0	0	0.0	0	0.0	0	0.0	0	0.0
Opportunistic	2021	(49,59]	Martinique	160	1	0.6	2	1.2	3	1.9	10	6.2	13	8.1
Opportunistic	2021	(49,59]	Mayotte	1	0	0.0	0	0.0	0	0.0	0	0.0	0	0.0
Opportunistic	2021	(59,66]	Guadeloupe	128	3	2.3	1	0.8	4	3.1	14	10.9	17	13.3
Opportunistic	2021	(59,66]	Guyane	2	0	0.0	0	0.0	0	0.0	0	0.0	0	0.0
Opportunistic	2021	(59,66]	La Réunion	1	0	0.0	0	0.0	0	0.0	0	0.0	0	0.0
Opportunistic	2021	(59,66]	Martinique	71	2	2.8	0	0.0	2	2.8	13	18.3	14	19.7

Table S4 **Number of tests and observed prevalence, stratified by genotype group, screening pathway, year, age, and district.** ***(continued)***

Screening	Year	Age	District	Number of tests (N)	N	%	N	%	N	%	N	%	N	%
Opportunistic	2021	(66,76]	Guadeloupe	19	0	0.0	2	10.5	2	10.5	2	10.5	3	15.8
Opportunistic	2021	(66,76]	Martinique	13	0	0.0	1	7.7	1	7.7	3	23.1	3	23.1
Opportunistic	2022	(29,39]	Guadeloupe	6	1	16.7	0	0.0	1	16.7	3	50.0	4	66.7
Opportunistic	2022	(29,39]	Guyane	1	0	0.0	0	0.0	0	0.0	0	0.0	0	0.0
Opportunistic	2022	(29,39]	La Réunion	2	0	0.0	0	0.0	0	0.0	0	0.0	0	0.0
Opportunistic	2022	(29,39]	Martinique	4	1	25.0	0	0.0	1	25.0	1	25.0	1	25.0
Opportunistic	2022	(29,39]	Mayotte	1	0	0.0	0	0.0	0	0.0	1	100.0	1	100.0
Opportunistic	2022	(39,49]	Guadeloupe	1	0	0.0	0	0.0	0	0.0	0	0.0	0	0.0
Opportunistic	2022	(39,49]	Guyane	1	0	0.0	0	0.0	0	0.0	0	0.0	0	0.0
Opportunistic	2022	(39,49]	La Réunion	2	0	0.0	0	0.0	0	0.0	0	0.0	0	0.0
Opportunistic	2022	(39,49]	Martinique	1	0	0.0	0	0.0	0	0.0	1	100.0	1	100.0
Opportunistic	2022	(39,49]	Mayotte	1	0	0.0	0	0.0	0	0.0	1	100.0	1	100.0
Opportunistic	2022	(49,59]	Guadeloupe	5	1	20.0	0	0.0	1	20.0	1	20.0	1	20.0
Opportunistic	2022	(49,59]	Guyane	1	0	0.0	0	0.0	0	0.0	1	100.0	1	100.0
Opportunistic	2022	(59,66]	Guadeloupe	4	0	0.0	0	0.0	0	0.0	0	0.0	0	0.0
Opportunistic	2022	(59,66]	Martinique	2	0	0.0	0	0.0	0	0.0	0	0.0	0	0.0
Opportunistic	2022	(66,76]	Guadeloupe	1	0	0.0	0	0.0	0	0.0	0	0.0	0	0.0
Opportunistic	2022	(66,76]	Martinique	1	0	0.0	0	0.0	0	0.0	0	0.0	0	0.0
Opportunistic	2023	(29,39]	Guadeloupe	1	0	0.0	0	0.0	0	0.0	0	0.0	0	0.0
Opportunistic	2023	(29,39]	Guyane	1	0	0.0	0	0.0	0	0.0	0	0.0	0	0.0
Opportunistic	2023	(29,39]	La Réunion	2	0	0.0	0	0.0	0	0.0	0	0.0	0	0.0
Opportunistic	2023	(29,39]	Martinique	2	0	0.0	0	0.0	0	0.0	1	50.0	1	50.0
Opportunistic	2023	(39,49]	Guadeloupe	2	0	0.0	0	0.0	0	0.0	0	0.0	0	0.0
Opportunistic	2023	(39,49]	Guyane	1	0	0.0	0	0.0	0	0.0	0	0.0	0	0.0
Opportunistic	2023	(39,49]	Martinique	4	1	25.0	0	0.0	1	25.0	0	0.0	1	25.0
Opportunistic	2023	(49,59]	Guadeloupe	3	0	0.0	0	0.0	0	0.0	1	33.3	1	33.3
Opportunistic	2023	(49,59]	Martinique	4	0	0.0	0	0.0	0	0.0	1	25.0	1	25.0
Opportunistic	2023	(49,59]	Mayotte	1	0	0.0	0	0.0	0	0.0	0	0.0	0	0.0
Opportunistic	2023	(59,66]	Guadeloupe	4	0	0.0	0	0.0	0	0.0	1	25.0	1	25.0
Opportunistic	2023	(59,66]	Guyane	1	0	0.0	0	0.0	0	0.0	0	0.0	0	0.0
Opportunistic	2023	(59,66]	La Réunion	1	0	0.0	0	0.0	0	0.0	0	0.0	0	0.0

Table S4 **Number of tests and observed prevalence, stratified by genotype group, screening pathway, year, age, and district.** ***(continued)***

Screening	Year	Age	District	Number of tests (N)	N	%	N	%	N	%	N	%	N	%
Opportunistic	2023	(59,66]	Martinique	2	0	0.0	0	0.0	0	0.0	0	0.0	0	0.0
Organised	2020	(59,66]	Martinique	1	0	0.0	0	0.0	0	0.0	0	0.0	0	0.0
Organised	2021	[24,25]	Martinique	1	0	0.0	0	0.0	0	0.0	0	0.0	0	0.0
Organised	2021	(25,29]	Martinique	1	0	0.0	0	0.0	0	0.0	0	0.0	0	0.0
Organised	2021	(29,39]	Martinique	1	0	0.0	0	0.0	0	0.0	0	0.0	0	0.0
Organised	2021	(39,49]	Mayotte	1	0	0.0	0	0.0	0	0.0	1	100.0	1	100.0
Organised	2021	(49,59]	Martinique	10	0	0.0	0	0.0	0	0.0	0	0.0	0	0.0
Organised	2021	(59,66]	Guadeloupe	1	0	0.0	0	0.0	0	0.0	0	0.0	0	0.0
Organised	2021	(59,66]	Martinique	9	0	0.0	0	0.0	0	0.0	1	11.1	1	11.1
Organised	2022	(29,39]	La Réunion	1	0	0.0	0	0.0	0	0.0	0	0.0	0	0.0
Organised	2023	(39,49]	Mayotte	1	0	0.0	1	100.0	1	100.0	0	0.0	1	100.0
Organised	2023	(49,59]	Guyane	1	0	0.0	0	0.0	0	0.0	0	0.0	0	0.0
Organised	2023	(59,66]	Guadeloupe	1	1	100.0	0	0.0	1	100.0	0	0.0	1	100.0

S5 Additional statistical details

S5.1 Complete textual description of the statistical model

The statistical inference relied on a full Bayesian approach. We aggregated the number of tests and positive test results at the stratum level. Strata were defined by a grid based on the screening pathway (opportunistic or organised), age (30 to 66 years), week-year of screening, postcode, and genotype groups. Two groups of genotypes were considered: HPV16 and/or HPV18 (hereafter denoted as 'HPV16/18') and genotypes other than HPV16/18 (hereafter denoted 'Other genotypes'). The aggregation of the number of tests positive for HPV16/18 was motivated by the fact that HPV16 and HPV18 stand out in terms of oncogenicity compared with other genotypes [4], such that public health policies often do not distinguish between them. For example, these two genotypes are the only ones included in all the licensed vaccines available in France [5, 6].

The number of positive tests within each stratum was assigned a binomial distribution, the size parameter of which was the total number of tests performed within each stratum. The expected proportion of positive tests within a stratum was linked, through the standard logistic function (i.e., the inverse logit function), to a set of predictors using each stratum's characteristics as input.

Each stratum's linear predictor included a common intercept and additional parameters associated with the full two-way interaction between dummy variables associated with strata related to other genotypes and opportunistic screening (minus one of those interactions, for identifiability of the intercept). Other components were split into two groups. Each group of parameters contained spatially-varying, age-varying, and time-varying parameters to which we assigned multivariate hierarchical priors. The first group was common to both data collected through opportunistic and organised screening. The second group was specific to data collected through opportunistic screening and allowed us to capture the potential systematic difference in expected prevalence associated with this screening pathway.

Spatially-varying parameters were specified through two-dimensional continuous functions, to which we assigned a Gaussian random field (GRF) prior, i.e., a bivariate Gaussian process (GP) [7–9] (Models 1 and 2). Alternatively, for models 3 to 6, the spatially-varying parameters were specified through discrete spatially-indexed parameters to which we assigned a scaled reparameterised Besag-York-Mollié (BYM2) prior, a Gaussian Markov random field prior (GMRF) which is the current workhorse in spatial statistics for areal data [10–13]. Age-specific and time-specific components were each specified through ordered parameters to which we assigned a Random Walk of order 2 (RW2), a GMRF also known as Bayesian P-splines, as a prior [10, 14].

For models involving spatial GRFs, we assigned to each stratum the centroid of the corresponding postcode. We used the stochastic partial differential equation approach (SPDE) to build computationally efficient representations of all the GPs [8, 9]. In two dimensions, the approach relies on the use of a triangular mesh, which was built using the French coordinate reference system, Lambert-93 (EPSG:2154), with kilometre as the distance unit. We tested two different GRFs: one GRF with a stationary Matérn covariance function [8] (model 1) and the non-stationary Barrier model [15] (model 2). The latter model accounted for the Mediterranean Sea and neighbouring countries as a natural barrier, making Corsica a separate area from mainland France. For this model, the fraction of the range parameter for the barrier domain was set to 0.1 of that of the land domain [15]. The final mesh is available in Supplementary Files S5.

Conversely, for the BYM2 priors, the spatial index was built from the list of French postcodes (see Supplementary Files S2). Four different neighbourhood matrices were considered: the Delaunay Triangulation structure (model 3, [16]), the Sphere of Influence (SOI) subgraph of the Delaunay Triangulation structure (model 4, [17]), and the first-order or second-order Queen contiguity structure (model 5 and 6, [18]).

All models accounted for the bivariate nature of the outcome through an exchangeable separable correlation structure between the two groups of genotypes superimposed on each age, space, and time-specific components following [19]. The approach of Riebler et al. [19] relies on the use of a Kronecker product between two precision matrices, thereby assuming a multiplicative separable covariance function [20, 21]. This is a classical approach in the analysis of multidimensional data [21]. This bivariate structure allowed us to test for between-genotype differences while accounting for the fact that infection prevalences were computed from tests performed on the same participants and originated from similar contact and transmission patterns.

All hyperparameters of G(M)RF components, except those for the correlation structure, were assigned Penalising Complexity (PC) priors, to mitigate the risk of overfitting inherent in such highly flexible model components [22–24]. Similarly, each parameter specific to other genotypes, opportunistic screening, and the interaction between these two dimensions received a hierarchical prior. The standard deviation for this prior was, in turn, assigned a parameterised PC prior. These PC priors allowed the posterior of these components to shrink towards 0 if not supported by the data. In addition, they allowed us to consider larger tails, while ensuring regularisation, compared with a standard Gaussian distribution (Supplementary Figure S9). PC priors are intuitively parameterised through two parameters, specifying the probability for the parameter of interest to be greater or lower than a threshold, depending on the type of PC prior. These parameters enable the control of the strength of the penalty applied to the deviation from the base model and hence the amount of support that should be provided by the data to support this additional component.

All assigned priors were very weak priors, meaning that high variance and correlation were not excluded *a priori*. PC priors for the marginal variances of the G(M)RF components were parameterised such that the probability for these parameters to be greater than 10 was 0.01. PC priors for the range parameters were parameterised such that the probability for these parameters to be lower than half the average distance between the two furthest points of metropolitan France on the latitude and longitude axes (i.e., 545 km), was 0.99. The relative weights between the structured and unstructured heterogeneity of BYM2 components received a PC prior parameterised such that the probability to be lower than 0.5 was 0.5. The global intercept, common to all data, received a univariate Gaussian prior with a variance equal to 10 (i.e., a precision equal to 0.1). The prior for other additional intercepts was specified such that half the probability mass was assigned to values greater than 1. Finally, all latent parameters of the exchangeable correlation structure were parameterised using the general Fisher's z-transformation. The corresponding latent parameters were assigned univariate Gaussian distribution as prior with variance set to 5, resulting in a U-shaped prior allowing us to put sufficient probability mass on extreme values and hence not excluding *a priori* extreme correlation [19].

We discriminated between the six competing spatial structures through the use of leave-one-group-out-cross-validation approach, following [25, 26]. We first computed the posterior predictive density of each observed data point, conditioning on the non-observation of a group of data, further named leave-one-group-out-cross-validation (LOGO-CV). The group of data used to compute this quantity, comprising 32 observations, was selected automatically using the posterior correlation matrix of the model [25, 26]. We further computed the logarithmic score associated with these LOGO-CV, defined as the negative of the average logarithmic LOGO-CV score, and selected the model with the lowest value [26, 27].

We estimated the model using the `inlabru` package [28, 29] (development version as of 29/01/2025, test version 2.12.0.9002), a wrapper around the `R-INLA` package (development version as of 29/01/2025, test version 25.01.23, Rocky Linux-8.10 (Green Obsidian) build) [30, 31], in R version 4.4.0 [32]. All quantities were computed using 3,000 draws from the full joint posterior distribution, except for Marginal Difference in Expected Prevalence (see Supplementary subsection S5.4) for which we used only 1,550 draws due to computational constraints.

The sensitivity analyses explored the effect on the selected model of assigning to correlation parameters

a prior with a variance of 1.25 and 2.5, instead of 5. Such a change had the effect of decreasing the weights assigned to extreme values of correlation [19]. We also reported the results of the non-selected models.

S5.2 Mathematical details of the statistical model

Notations and references

- Let B^{-} denote the generalised inverse of the matrix B.
- Let $\mathcal{N}\left(\mu, \sigma^2\right)$ be the Gaussian distribution with expectation μ and variance σ^2.
- Let $\tau = \sigma^{-2}$ be the precision parameter.
- Let $\mathrm{Cov}(X, Y)$ be the covariance and $\mathrm{Corr}\,(X, Y)$ be the correlation between the two random variables X and Y.
- Let $\mathcal{PC}_X$ denote the appropriate Penalising Complexity (PC) prior for the random variable X (see [22–24]).
- The PC prior for the precision parameter $\tau = \sigma^{-2}$ of a GMRF corresponds to a type-2 Gumbel distribution. It corresponds to an exponential distribution for the standard deviation. Considering the Wasserstein distance within the PC framework introduced in [22, 23], instead of the Kullback–Leibler Divergence (KLD), leads to the same prior [33] [1].
- Let $d(z, z')$ be the Euclidean distance for $(z, z') \in \mathbb{R}^d \times \mathbb{R}^d,\ d \geq 1$.
- A Gaussian random field $u : \mathbb{R}^d \times \mathbb{R}^d \to \mathbb{R}^d$ is defined through the following system:

$$\begin{cases} \mathbb{E}\left(x(z)\right) = m(z) \\ \mathrm{Cov}\left(x(z), x(z')\right) = k\left(z, z'\right) \\ \left(x(z_i)\right)_{i \in \{1,\dots,n\}} \sim \mathcal{N}\left(\left(m(z_i)\right)_{i \in \{1,\dots,N\}}, \left(k(z_i, z_j)\right)_{(i,j) \in \{1,\dots,N\}^2}\right) \end{cases}$$

  for all finite sets of locations $(z_1, \dots, z_N)$. Here, $k\,(.,.)$ is the covariance function (yielding a positive semi-definite correlation matrix), which is a positive definite function and $m\,(.)$, the mean function (see Chapter 4 in reference [7]).

  If x is a GRF, we will be using the following notation: $x(z) \sim \mathcal{GP}\left(m\left(z\right), k\left(z, z'\right)\right)$.

- Various choices are available for the covariance kernel $k(.,.)$. In spatial statistics, the most widely used choice is the Matérn covariance function, which has dominated the field for more than half a century [34, 35].
- Let $C_{\sigma,\nu,\kappa}(z, z')$ be the Matérn covariance function between any two locations $(z, z') \in \mathbb{R}^d \times \mathbb{R}^d$. In our use case, $d = 2$. The Matérn covariance function is parameterised through three parameters: the marginal variance σ^2, the scaling parameter $\kappa > 0$, and the smoothness parameter $\nu > 0$.

$$C_{\sigma,\nu,\kappa}(z, z') = \sigma^2 \frac{1}{2^{\nu-1}\Gamma(\nu)} \left(\kappa d(z, z')\right)^{\nu} K_{\nu}\left(\kappa d(z, z')\right)$$

  where Γ is the gamma function[2], K_ν is the modified Bessel function of the second kind.

  Following Lindgren et al. [8], one may use the empirically derived definition $\rho = \frac{\sqrt{8\nu}}{\kappa}$, giving the range parameter ρ the following natural interpretation: it is the distance at which the covariance function becomes about 0.1 [8].

  The smoothing parameter ν controls the mean-square differentiability of the underlying process. This parameter is usually poorly identifiable and Lindgren et al. [8] define it as $\nu = \alpha - \frac{d}{2} > 0$, with $\alpha \in (0, 2]$ a fixed parameter. We used $\alpha = 2$, which is the usual default value.

[1] The related R-INLA webpage for the PC prior is https://inla.r-inla-download.org/r-inla.org/doc/prior/pc.prec.pdf.
[2] $\forall z \in \mathbb{C},\ Re(z) > 0, \Gamma(z) = \int_0^{+\infty} t^{z-1}\,\mathrm{e}^{-t}\,\mathrm{d}t$, where $Re(z)$ denotes the real part of z.

With such a parameterisation, the covariance function is stationary, meaning that it only depends on the relative position of the two locations, and isotropic, meaning that the covariance between two points depends only on the Euclidean distance between the locations.

Additional details are available in [8, 9].

- Lindgren et al. [8] present a computationally efficient way to compute Matérn models (i.e., GRF with Matérn covariance function). This approach is routinely available in `R-INLA` and was used to handle GRF model components. The mesh we used for carrying out this approximation had 18,648 nodes. This mesh is represented in the Supplementary Figure S8.

- The Matérn covariance function presented above is stationary and isotropic. In real applications, physical barriers or holes can lead to a violation of this assumption [15]. To handle such cases, Bakka et al. [15] introduced a non-stationary GRF accounting for physical barriers using the efficient computation approach introduced by Lindgren et al. [8].

  To handle physical barriers or holes in the spatial domain of interest, the Barrier model introduces a partition of the domain into two disjoint subspaces. The first of these subspaces corresponds to the normal area (denoted as Ω_n) while the second part corresponds to physical barriers (denoted as Ω_b). Each partition receives its own Matérn covariance function. They both share the same marginal standard deviation but each has its own range parameter.

$$\begin{cases} \forall(z,z') \in \Omega_n : C_{\sigma,\nu,\rho}(z,z') = \sigma^2 \frac{1}{2^{\nu-1}\Gamma(\nu)} \left(\frac{\sqrt{8\nu}}{\rho} d(z,z')\right)^{\nu} K_\nu \left(\frac{\sqrt{8\nu}}{\rho} d(z,z')\right) \\ \forall(z,z') \in \Omega_b : C_{\sigma,\nu,\rho_b}(z,z') = \sigma^2 \frac{1}{2^{\nu-1}\Gamma(\nu)} \left(\frac{\sqrt{8\nu}}{\rho_b} d(z,z')\right)^{\nu} K_\nu \left(\frac{\sqrt{8\nu}}{\rho_b} d(z,z')\right) \end{cases}$$

  To keep the computational cost close to that of the stationary case, the range applied to the barrier domain, ρ_b, is specified as a fraction of that of the normal domain, ρ: $\rho_b = k \times \rho$. We used the default value for k: $k = 0.1$.

  We considered the ocean, seas, and neighbouring countries as natural barriers.

- The PC prior for the parameters of the Matérn covariance function is derived in [24]. Additional details can be found in the documentation of the `inla.spde2.pcmatern()` function in `R-INLA` [30, 31], available in https://inla.r-inla-download.org/r-inla.org/doc/prior/pc.matern.pdf. The PC prior for the barrier model is available in [15].

- The `R-INLA` webpage, which describes the SPDE representation of the GP process with a stationary Matérn covariance function, is available here: https://inla.r-inla-download.org/r-inla.org/doc/prior/pc.matern.pdf

- The Barrier SPDE model [15] is implemented in the INLAspacetime package, see https://github.com/eliaskrainski/INLAspacetime

- Let I_S be the identity matrix with S rows and columns.

- Let R be a spatially structured matrix such that R_{ij}, the element of row i and column j, is defined as $R_{ij} = \begin{cases} n_i \text{ if } i = j \\ -1 \text{ if } i \sim j \\ 0 \textit{ else} \end{cases}$, with n_i the number of neighbouring areas for the spatial unit i and $\sim$ denoting the adjacency (neighbouring) predicate.

- The Matérn and Barrier model considers space in its native continuous form. Because strata are associated with postcodes, we had to assign arbitrary specific coordinates (i.e., those of the centroid) for using these approaches. A more suitable approach not assigning these arbitrary coordinates is to consider that the layout of cities in space forms an irregular lattice and then

rely on approaches developed specifically to deal with such data, such as the BYM2 model, introduced in [11]. The BYM2 model is the current workhorse in areal spatial statistics, notably because it responds to criticisms directed towards previously developed priors [10–13].

Let $\alpha = (\alpha_1, \ldots, \alpha_S)$ denote a vector of parameters such that $\forall i \in \{1, \ldots S\}$, $\alpha_i = \frac{1}{\sqrt{\tau}}\left(\sqrt{1-\phi}v_i + \phi u_i\right)$ with $\tau = \sigma^{-2}$ the marginal precision parameter, $\phi \in [0,1]$ the relative weight of the structured heterogeneity, $v \sim \mathcal{N}(0, I_S)$, and $u \sim \mathcal{N}(0, R_*^-)$. The spatial structure matrix R_* denotes the scaled counterpart of the matrix R, meaning that the average variance (the diagonal of the generalised inverse) is equal to 1, see section 3.2. of [11].

The covariance matrix of α is given by $\mathrm{Var}(\alpha \mid \tau, \phi) = \tau^{-1}\left((1-\phi) I + \phi R_*^-\right)$.

For identifiability, a sum-to-zero constraint is imposed on the components of the scaled iCAR/BYM model associated with each connected subgraph of the matrix R [11].

- The BYM2 model, like other spatially structured GMRFs, relies on a neighbourhood matrix encoding the conditional dependencies between the data.

  Two distance-based neighbourhood matrices were considered, using the centroid of each postcode. The first of these distances was the Delaunay Triangulation approach, which is a geometric-based approach connecting areas into triangles such that the minimum angle of all triangles is maximised [16]. The second one consisted of the thinning of the Delaunay Triangulation structure through the sphere of influence approach. Starting from the Delaunay Triangulation neighbourhood structure, two areas were linked to each other if their respective nearest-neighbour circles from the nearest neighbour, defined as the largest circle centred at the centroid of postcode p that contains no other points than this centroid, overlapped.

  A third and a fourth matrix were defined based on the first-order and second-order Queen contiguity approach, respectively.

  We used the `spdep` package to define spatial neighbourhood [36–38].

- The PC prior for the BYM2 model is provided in [11] and is implemented in the `R-INLA` package, see https://inla.r-inla-download.org/r-inla.org/doc/latent/bym2.pdf

- Let $\beta = (\beta_1, \ldots, \beta_n)$. Let β' be the transpose of β. The RW(2) prior has an improper (rank deficiency equal to $n-2$) prior density given by:

$$\pi_\beta(\beta \mid \tau) \propto \tau^{\frac{n-2}{2}} \exp\left(-\frac{1}{2}\beta' Q \beta\right), \; Q = \tau R_{\mathrm{RW}(2)}$$

  With $R_{\mathrm{RW}(2)}$ the structure matrix defined as:

$$R_{\mathrm{RW}(2)} = \begin{pmatrix} 1 & -2 & 1 & 0 & 0 & \ldots & 0 & 0 \\ -2 & 5 & -4 & 1 & 0 & \ldots & 0 & 0 \\ 1 & -4 & 6 & -4 & 1 & \ldots & 0 & 0 \\ 0 & 1 & -4 & 6 & -4 & \ddots & 0 & 0 \\ \vdots & \vdots & \ddots & \ddots & \ddots & \ddots & \vdots & \vdots \\ 0 & 0 & \ldots & 1 & -4 & 6 & -4 & 1 \\ 0 & 0 & \ldots & 0 & 1 & -4 & 5 & -2 \\ 0 & 0 & \ldots & 0 & 0 & 1 & -2 & 1 \end{pmatrix}$$

In practice, sum-to-zero constraints are applied to make the joint density proper and obtain a finite marginal standard deviation, as described in reference [39]. In addition, the precision

matrix for this iGMRF is scaled for reasons explained in [11, 39].

More details are provided in references [10, 14, 39].

- The R-INLA webpage, which describes the RW(2) prior, is available here: https://inla.r-inla-download.org/r-inla.org/doc/latent/rw2.pdf
- The uniform exchangeable correlation structure through Gaussian Kronecker product Markov random field is introduced in [19].

Fit on HPC

Model fit, computation of GCPO, and draws from the joint posterior were performed within the same R-session for models 1 and 2 and models 3 to 6. The Barrier model (Model 2) used results from the Stationary Matérn model (model 1) as starting values for the optimisation process. Similarly, models 3, 4, and 6 used the results from model 5 (BYM2 with Queen-contiguity structure) as starting values for the optimisation process. Once all fits, GCPOs, and draws were obtained, two new R sessions were launched. The first session performed the post-fit analyses for non-selected models, fitted the two additional sensitivity analyses, and ran the post-analysis R-function on the selected model, which took almost one week to complete because of all the quantities to compute and the large number of draws considered.

HPC logs can be found on the GitHub page of the paper: https://github.com/osupplisson/hpv_prevalence/tree/main/log_hpc.

Full joint model

Let s denote a stratum. Let $\mathcal{S}$ be the set of all strata.

A stratum s is defined by a 5-tuple $s = (a_s, z_s, t_s, g_s, c_s)$ where a_s denotes the age, z_s the postcode, t_s denotes the week-year of screening, g_s the genotype, and c_s denotes the screening pathway.

The postcode is a five-digit number, age a_s is an integer belonging to the set $\{30, \ldots, 66\}$. The week-year ranges from the week-year of 2020-08-17 to the week-year 2023-11-27, g_s is either 'HPV16/18' or 'Other genotypes', and c_s is either 'Organised screening' or 'Opportunistic screening'.

Depending on the model, this 5-tuple was mapped to a numeric 5-tuple used as inputs of the model components.

- For space: the postcode was mapped to the centroid (longitude,latitude) of the corresponding spatial area for GRF components and to a unique spatial index for GMRF components;
- For age: the age was mapped to an age-specific index starting at 1 for females aged 30 and ending at 36 for females aged 66;
- For time: the week-year was mapped to a time-specific index starting at 1 for the first week-year and ending at 174;
- For genotypes: 'HPV16/18' was assigned index 1 and 'Other genotypes' was assigned index 2;
- For screening pathways: 'Organised screening' was assigned index 1 and 'Opportunistic screening' was assigned index 2;

Using these notations, the full hierarchical model is given by the set of specifications in the system below. For clarity, the symbol indicating conditional dependencies has been omitted from the system. In short, the observation level is conditional on the latent field, and the latent field is itself conditional on the hyperparameter level. Within the observation level, y_s is conditional on (N_s, p_s), and p_s is conditional on μ_s, which is conditional on the latent field.

$$
\begin{cases}
\text{Observation level}
\begin{cases}
y_s \sim \mathcal{B}in\left(N_s, p_s\right) \\
\begin{cases}
p_s = \frac{1}{1+\exp(-\mu_s)} \\
\\
\mu_s = \psi_0 + \mathbb{1}_{g_s=2}\psi_1 + v_{\text{SPACE}}(z_s, g_s) + \gamma_{\text{AGE}}(a_s, g_s) + \beta_{\text{TIME}}(t_s, g_s) + \\
\mathbb{1}_{c_s=2}\left[\psi_2 + \mathbb{1}_{g_s=2}\psi_3 + v'_{\text{SPACE}}(z_s, g_s) + \gamma'_{\text{AGE}}(a_s, g_s) + \beta'_{\text{TIME}}(t_s, g_s)\right] \text{ Model 1 and 2} \\
\\
\mu_s = \psi_0 + \mathbb{1}_{g_s=2}\psi_1 + \alpha_{\text{SPACE}}(z_s, g_s) + \gamma_{\text{AGE}}(a_s, g_s) + \beta_{\text{TIME}}(t_s, g_s) + \\
\mathbb{1}_{c_s=2}\left[\psi_2 + \mathbb{1}_{g_s=2}\psi_3 + \alpha'_{\text{SPACE}}(z_s, g_s) + \gamma'_{\text{AGE}}(a_s, g_s) + \beta'_{\text{TIME}}(t_s, g_s)\right] \text{ Model 3 to 6}
\end{cases}
\end{cases} \\
\text{Parameters}
\begin{cases}
\psi_0 \sim \mathcal{N}(0, 10) \\
\psi_{j\in\{1,2,3\}} \sim \mathcal{N}\left(0, \sigma^2_{\psi_{j\in\{1,2,3\}}}\right) \\
\text{Hyperparameters for } \psi_{j\in\{1,2,3\}} \begin{cases} \sigma_{\psi_{j\in\{1,2,3\}}} \sim \mathcal{PC}_\sigma(1, 0.5) \end{cases} \\
\textbf{RW(2) for age and exchangeable correlation structure between genotypes} \\
\text{for } f \in \{\gamma, \gamma'\},\ (g_s, g'_s) \in \{1,2\}^2,\ a_s \in \{1, \ldots, 36\}: \\
\text{Latent field} \begin{cases} f_{\text{AGE}}(a_s, g_s) - 2f_{\text{AGE}}(a_s+1, g_s) + f_{\text{AGE}}(a_s+2, g_s) \sim \mathcal{N}\left(0, \sigma^2\right) \\ \text{Corr}(f_{\text{AGE}}(a_s, 1), f_{\text{AGE}}(a_s, 2)) = \eta_{f_{\text{AGE}}} \end{cases} \\
\text{Hyperparameters} \begin{cases} \eta_{f_{\text{AGE}}} = \frac{\exp(\eta^*_{f_{\text{AGE}}})-1}{\exp(\eta^*_{f_{\text{AGE}}})+2-1} \\ \eta^*_{f_{\text{AGE}}} \sim \mathcal{N}(0, 5) \\ \sigma_{f_{\text{AGE}}} \sim \mathcal{PC}_\sigma\left(\lambda_{\sigma_{f_{\text{AGE}}}}\right) \\ \lambda_{\sigma_{f_{\text{AGE}}}} = -\frac{-\log\left(p_{\sigma_{f_{\text{AGE}}}}\right)}{\sigma_0},\ \Pr(\sigma > \sigma_0) = p_{\sigma_{f_{\text{AGE}}}} \\ \left(\sigma_0, p_{\sigma_{f_{\text{AGE}}}}\right) = (10, 0.01) \end{cases} \\
\textbf{RW(2) for time and exchangeable correlation structure between genotypes} \\
\text{for } f \in \{\beta, \beta'\},\ (g_s, g'_s) \in \{1,2\}^2,\ t_s \in \{1, \ldots, 174\}: \\
\text{Latent field} \begin{cases} f_{\text{TIME}}(t_s, g_s) - 2f_{\text{TIME}}(t_s+1, g_s) + f_{\text{TIME}}(t_s+2, g_s) \sim \mathcal{N}\left(0, \sigma^2\right) \\ \text{Corr}(f_{\text{TIME}}(t_s, 1), f_{\text{TIME}}(t_s, 2)) = \eta_{f_{\text{TIME}}} \end{cases} \\
\text{Hyperparameters} \begin{cases} \eta_{f_{\text{TIME}}} = \frac{\exp(\eta^*_{f_{\text{TIME}}})-1}{\exp(\eta^*_{f_{\text{TIME}}})+2-1} \\ \eta^*_{f_{\text{TIME}}} \sim \mathcal{N}(0, 5) \\ \sigma_{f_{\text{TIME}}} \sim \mathcal{PC}_\sigma\left(\lambda_{\sigma_{f_{\text{TIME}}}}\right) \\ \lambda_{\sigma_{f_{\text{TIME}}}} = -\frac{-\log\left(p_{\sigma_{f_{\text{TIME}}}}\right)}{\sigma_0},\ \Pr(\sigma > \sigma_0) = p_{\sigma_{f_{\text{TIME}}}} \\ \left(\sigma_0, p_{\sigma_{f_{\text{TIME}}}}\right) = (10, 0.01) \end{cases} \\
\text{...see next page}
\end{cases}
\end{cases}
\tag{1.1}
$$

$$
\begin{cases}
\text{Parameters}
\begin{cases}
\textit{...next} \\
\textbf{Model 1, spatial GRF: GRF with stationary Matérn covariance for space} \\
\textbf{and exchangeable correlation structure between genotypes} \\
\text{for } f \in \{v, v'\},\ (g_s, g_s') \in \{1,2\}^2,\ \forall (z_s, z_s') \in \Omega \times \Omega : \\
\text{Latent field}
\begin{cases}
f_{\text{SPACE}}(z_s, g_s) \sim \mathcal{GP}\left(0, C_{\sigma_{f_{\text{SPACE}}}, \nu_{f_{\text{SPACE}}}, \rho_{f_{\text{SPACE}}}}(z_s, z_s')\right) \\
\text{Corr}(f_{\text{SPACE}}(z_s, 1), f_{\text{SPACE}}(z_s, 2)) = \eta_{f_{\text{SPACE}}}
\end{cases} \\
\text{Hyperparameters}
\begin{cases}
\eta_{f_{\text{SPACE}}} = \frac{\exp(\eta^*_{f_{\text{SPACE}}}) - 1}{\exp(\eta^*_{f_{\text{SPACE}}}) + 2 - 1} \\
\eta^*_{f_{\text{SPACE}}} \sim \mathcal{N}(0, 5) \\
\left(\rho_{f_{\text{SPACE}}}, \sigma_{f_{\text{SPACE}}}\right) \sim \mathcal{PC}_{(\rho,\sigma)}\left(\lambda_{\rho_{f_{\text{SPACE}}}}, \lambda_{\sigma_{f_{\text{SPACE}}}}\right) \\
\lambda_{\sigma_{f_{\text{SPACE}}}} = \text{arg}_{\in \mathbb{R}^+} : \Pr_{\lambda_{\sigma_{f_{\text{SPACE}}}}}\left(\sigma_{f_{\text{SPACE}}} \geq \sigma_0\right) = p_{\sigma_{f_{\text{SPACE}}}} \\
\lambda_{\rho_{f_{\text{SPACE}}}} = \text{arg}_{\in \mathbb{R}^+} : \Pr_{\lambda_{\rho_{f_{\text{SPACE}}}}}\left(\rho_{f_{\text{SPACE}}} \leq \rho_0\right) = p_{\rho_{f_{\text{SPACE}}}} \\
\left(\sigma_0, p_{\sigma_{f_{\text{SPACE}}}}\right) = (10, 0.01) \\
\left(\rho_0, p_{\rho_{f_{\text{SPACE}}}}\right) = (545, 0.99) \\
\alpha_{f_{\text{SPACE}}} = 2
\end{cases} \\
\\
\textbf{Model 2, spatial GRF: GRF with non-stationary Matérn covariance (Barrier model) for space} \\
\textbf{and exchangeable correlation structure between genotypes} \\
\Omega = \text{Spatial domain, definition domain for the GRF as given by the mesh} \\
\Omega_b = \text{Northern and Mediterranean seas, the Atlantic Ocean, the English Channel...} \\
\text{..., and foreign neighbouring countries (Spain, Italy...)} \\
\Omega_n = \text{Mainland France and Corsica} \\
\Omega = \Omega_n \cup \Omega_b,\ \Omega_n \cap \Omega_b = \varnothing \\
\text{for } f \in \{v, v'\},\ (g_s, g_s') \in \{1,2\}^2 : \\
\text{Latent field}
\begin{cases}
(z_s, z_s') \in \Omega_n^2 : f_{\text{SPACE}}(z_s, g_s) \sim \mathcal{GP}\left(0, C_{\sigma_{f_{\text{SPACE}}}, \nu_{f_{\text{SPACE}}}, \rho_{f_{\text{SPACE}}}}(z_s, z_s')\right) \\
(z_s, z_s') \in \Omega_b^2 : f_{\text{SPACE}}(z_s, g_s) \sim \mathcal{GP}\left(0, C_{\sigma_{f_{\text{SPACE}}}, \nu_{f_{\text{SPACE}}}, \rho_{b, f_{\text{SPACE}}}}(z_s, z_s')\right) \\
\text{Corr}(f_{\text{SPACE}}(z_s, 1), f_{\text{SPACE}}(z_s, 2)) = \eta_{f_{\text{SPACE}}}
\end{cases} \\
\text{Hyperparameters}
\begin{cases}
\eta_{f_{\text{SPACE}}} = \frac{\exp(\eta^*_{f_{\text{SPACE}}}) - 1}{\exp(\eta^*_{f_{\text{SPACE}}}) + 2 - 1} \\
\eta^*_{f_{\text{SPACE}}} \sim \mathcal{N}(0, 5) \\
\left(\rho_{f_{\text{SPACE}}}, \sigma_{f_{\text{SPACE}}}\right) \sim \mathcal{PC}_{(\rho,\sigma)}\left(\lambda_{\rho_{f_{\text{SPACE}}}}, \lambda_{\sigma_{f_{\text{SPACE}}}}\right) \\
\lambda_{\sigma_{f_{\text{SPACE}}}} = \text{arg}_{\in \mathbb{R}^+} : \Pr\left(\sigma_{f_{\text{SPACE}}} \geq \sigma_0\right) = p_{\sigma_{f_{\text{SPACE}}}} \\
\lambda_{\rho_{f_{\text{SPACE}}}} = \text{arg}_{\in \mathbb{R}^+} : \Pr_{\lambda_{\rho_{f_{\text{SPACE}}}}}\left(\rho_{f_{\text{SPACE}}} \leq \rho_0\right) = p_{\rho_{f_{\text{SPACE}}}} \\
\left(\sigma_0, p_{\sigma_{f_{\text{SPACE}}}}\right) = (10, 0.01) \\
\left(\rho_0, p_{\rho_{f_{\text{SPACE}}}}\right) = (545, 0.99) \\
\alpha_{f_{\text{SPACE}}} = 2
\end{cases} \\
\\
\text{...see next page}
\end{cases}
\end{cases}
\tag{1.2}
$$

$$
\left\{
\text{Parameters}
\begin{cases}
\textit{...next} \\
\textbf{Models 3 to 6, spatial GMRF: GMRF with BYM2 structure for space} \\
\textbf{and exchangeable correlation structure between genotypes} \\
\text{for } f \in \{\alpha, \alpha'\},\ (g_s, g_s') \in \{1,2\}^2,\ \forall z_s \in \text{Set of postcodes :} \\
\text{Latent field} \begin{cases} f_{SPACE}(g_s) \sim BYM2\left(\sigma_{\text{SPACE}}, \phi_{\text{SPACE}}, R\right) \\ \text{Corr}(f_{SPACE}(z_s,1), f_{SPACE}(z_s,2)) = \eta_{f_{\text{SPACE}}} \end{cases} \\
\text{Hyperparameters} \begin{cases}
\eta_{f_{\text{SPACE}}} = \frac{\exp(\eta^*_{f_{\text{SPACE}}})-1}{\exp(\eta^*_{f_{\text{SPACE}}})+2-1} \\
\eta^*_{f_{\text{SPACE}}} \sim \mathcal{N}(0,5) \\
\phi_{f_{SPACE}} \sim \mathcal{PC}_\phi\left(\lambda_{f_{SPACE}}\right) \\
\lambda_{f_{SPACE}} = \arg_{\in \mathbb{R}^+} : \Pr_{\lambda_{\sigma_{f_{\phi_{\text{SPACE}}}}}}\left(\phi_{f_{\text{SPACE}}} \leq \phi_0\right) = p_{\phi_{f_{\text{SPACE}}}} \\
\sigma_{f_{SPACE}} \sim \mathcal{PC}_\sigma\left(\lambda_{\sigma_{f_{SPACE}}}\right) \\
\lambda_{\sigma_{f_{SPACE}}} = -\frac{-\log\left(p_{\sigma_{f_{SPACE}}}\right)}{\sigma_0},\ \Pr\left(\sigma > \sigma_0\right) = p_{\sigma_{f_{SPACE}}} \\
\left(\sigma_0, p_{\sigma_{f_{SPACE}}}\right) = (10, 0.01) \\
\left(\phi_0, p_{\phi_{f_{SPACE}}}\right) = (0.5, 0.5) \\
R : \begin{cases} \text{Delaunay Triangulation (model 3)} \\ \text{SOI subgraph of the Delaunay Triangulation (model 4)} \\ \text{1st-order Queen contiguity (model 5)} \\ \text{2nd-order Queen contiguity (model 6)} \end{cases}
\end{cases}
\end{cases}
\right.
\tag{1.3}
$$

- We discriminated between competing models using the logarithmic score, computed using the leave-one-group-out conditional predictive ordinate (LOO-CPO), also known as leave-one-group-out cross-validation (LOGO-CV). Left-out groups comprised 32 observations which were automatically selected using the posterior correlation matrix of the model [26].
- Denoting π the probability density function and $y_{-\mathcal{G}_s}$ all observed data leaving out the group of data $\mathcal{G}_s$, defined using the posterior correlation matrix between data points [26]:

$$\begin{cases} \text{LOGO-CV}_s = p(y_s \mid y_{-\mathcal{G}_s}) \\ \text{log-score} = -\frac{1}{\text{card}(\mathcal{S})} \left(\sum_{s \in \mathcal{S}} \log \left(\text{LOGO-CV}_s \right) \right) \end{cases}$$

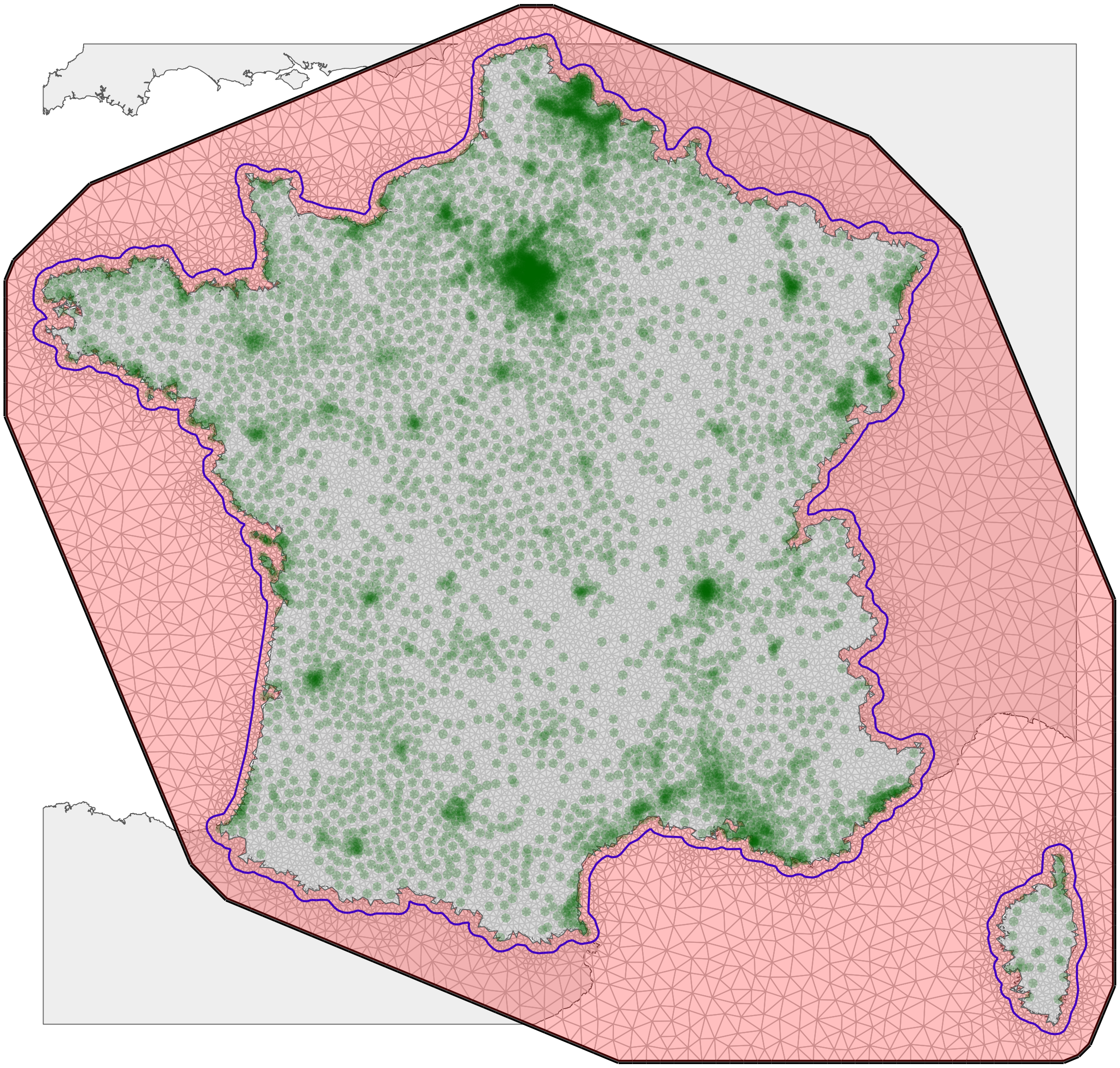

Red shaded area indicates the barrier accounted for by the Barrier model.

Figure S8 Spatial location of all data points (green dots) and mesh (grey edges) used for the finite-elements decomposition approximation of the Gaussian random fields. The blue line delineates the areas in which the density of the mesh could change. It was defined to be finer within metropolitan France than outside, where no data could be observed.

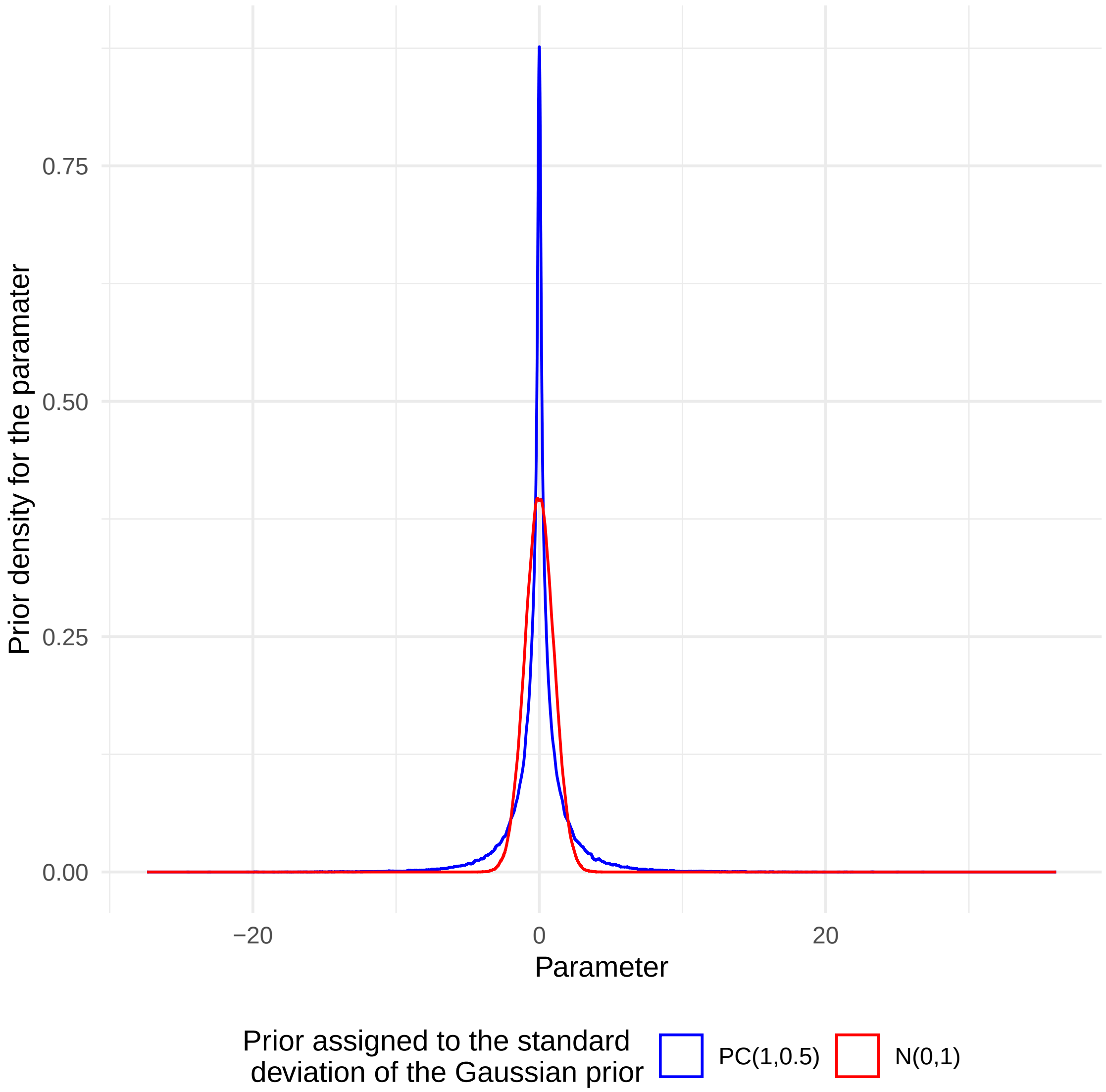

Figure S9 Prior density for a Gaussian prior distribution with PC(1,0.5) hyperprior assigned to the precision parameter (blue curve) compared with a standard Gaussian distribution (red curve) .

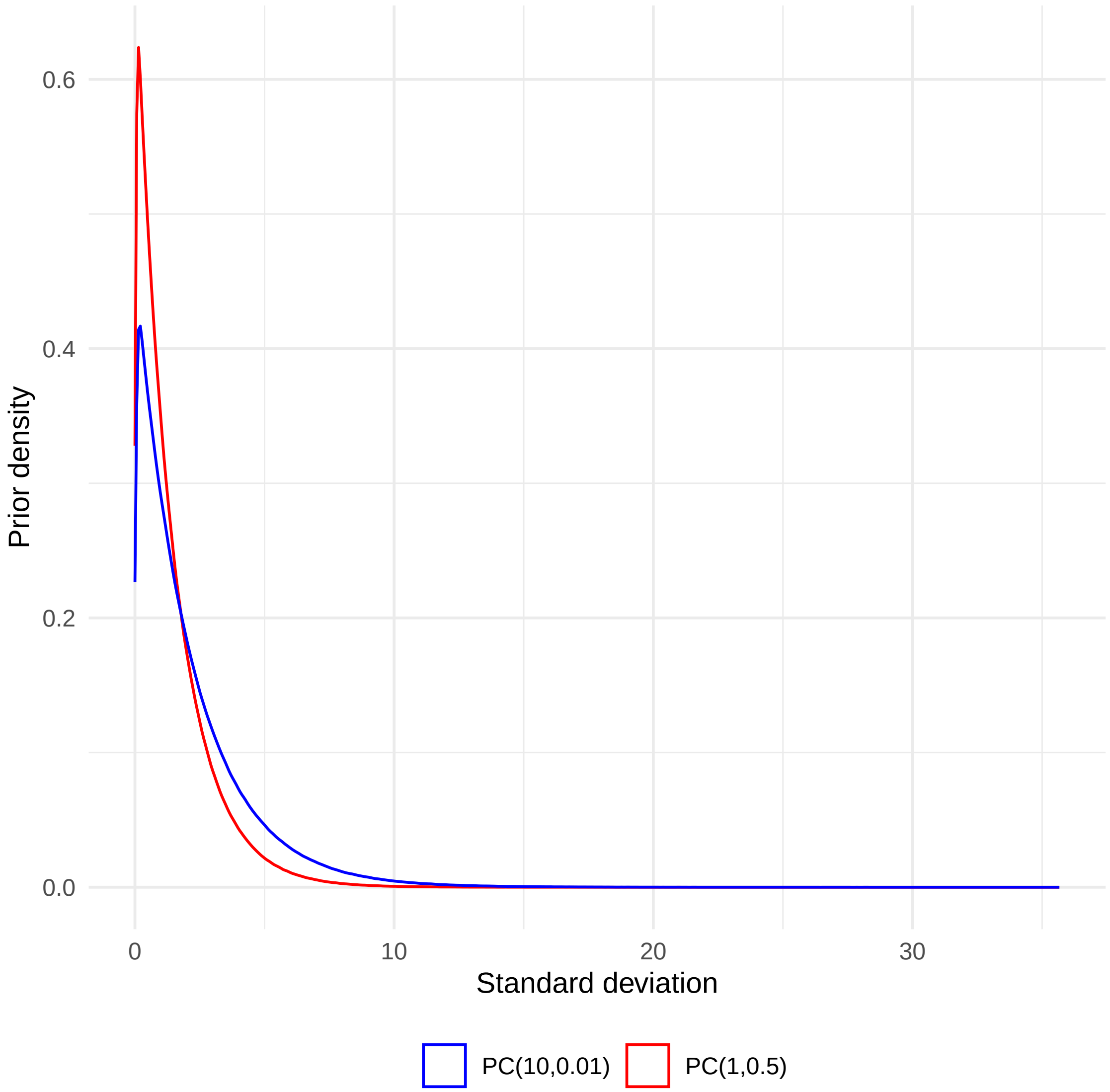

Figure S10 PC prior for the standard deviation of GMRFs.

S5.3 Details about the sensitivity analyses

Two sensitivity analyses were explored:

1. Reporting of the non-selected competing models;
2. Change in the prior for the correlation parameters: variance of 1.25 and 2.5, instead of 5. These changes are illustrated in the Supplementary Figures S11.

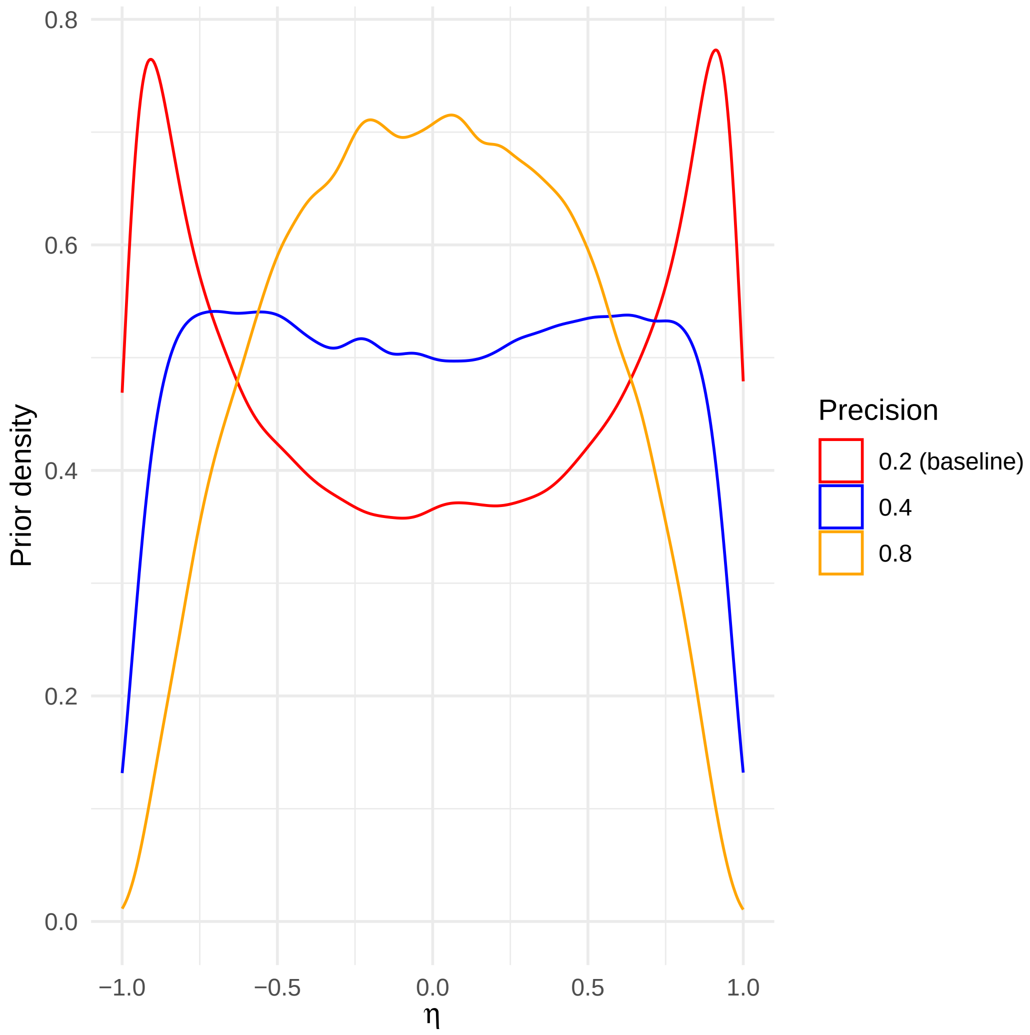

Figure S11 Precision for the prior distribution of the latent correlation parameters.

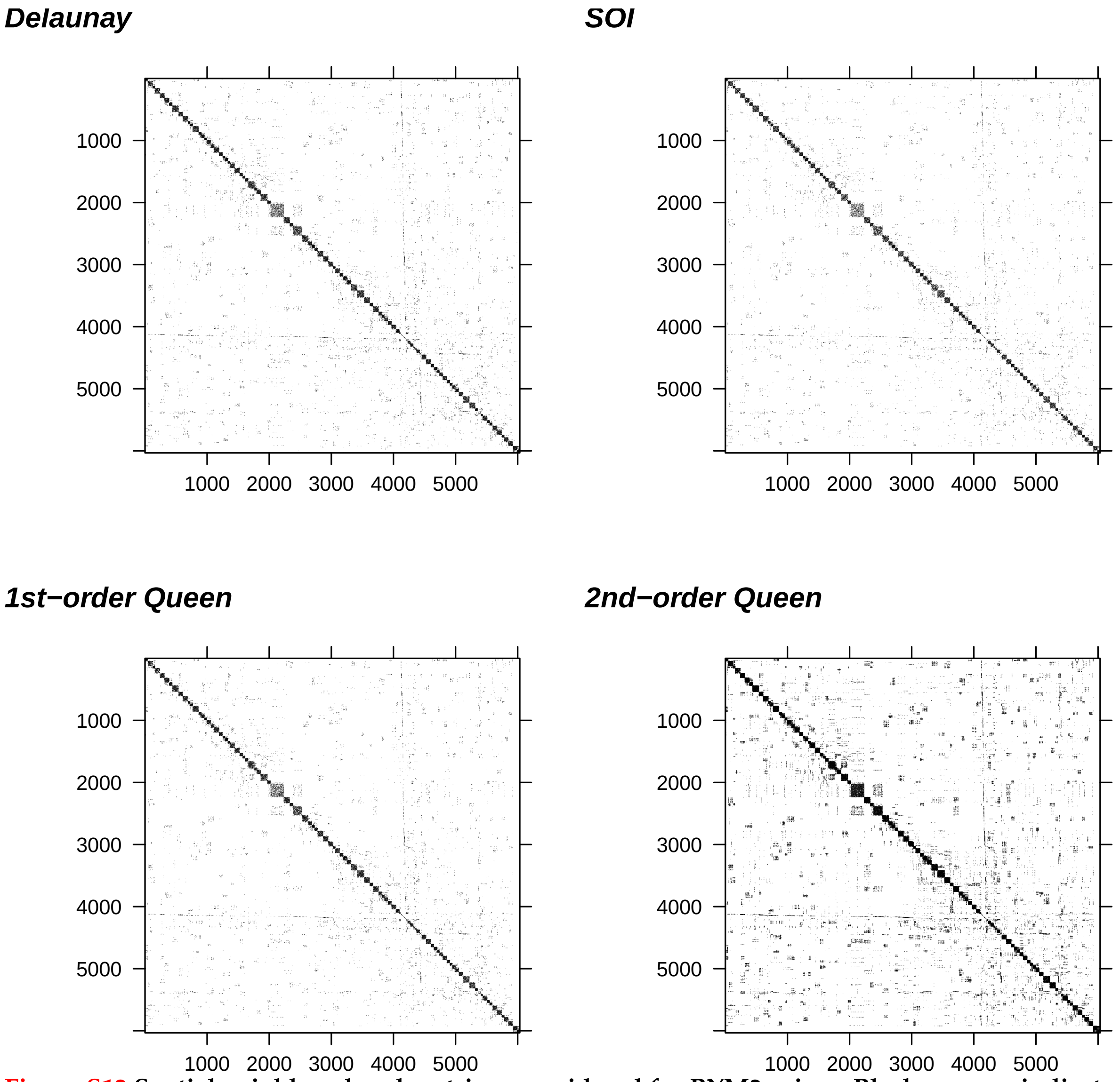

Figure S12 **Spatial neighbourhood matrices considered for BYM2 priors. Black squares indicate that the two areas are neighbours.**

S5.4 Details about the Marginal Difference in Expected Prevalence (MDEP)

To compute the MDEP, we considered each unique 4-tuple (a_s, z_s, t_s, g_s) and computed the corresponding number of observed tests (i.e., we summed the number of tests across the two screening pathways).

For each of these 4-tuples we then made 1,550 draws from the joint posterior distribution to obtain the expected proportion of positive tests. We did so considering, first, the model specific to the organised screening pathway and, second, the model specific to the opportunistic screening pathway. We then computed the expected number of positive tests under each screening pathway. We finally took the difference between the expected number of positive tests under opportunistic and organised screening and then divided this difference by the total number of tests, yielding the Marginal Difference in Expected Prevalence.

In mathematical form:

- Step 1. Compute the observed number of tests, integrating out the screening pathway: $N_{a_s,z_s,t_s,g_s} = N_{a_s,z_s,t_s,g_s,1} + N_{a_s,z_s,t_s,g_s,2}$
- Step 2. Draws from the full joint posterior distribution $\hat{p}_{a_s,z_s,t_s,g_s,1}$ and $\hat{p}_{a_s,z_s,t_s,g_s,2}$;
- Step 3. For each draw, compute the expected number of positive tests under each screening pathway: $\hat{p}_{a_s,z_s,t_s,g_s,1} \times N_{a_s,z_s,t_s,g_s}$ and $\hat{p}_{a_s,z_s,t_s,g_s,2} \times N_{a_s,z_s,t_s,g_s}$
- Step 4A. For each draw, compute the marginal prevalence integrating out various dimensions. To do so, sum over all focal dimensions the expected number of positive tests. Then, divide this sum by the observed number of tests. For example, for a given screening pathway c_s, integrating out all dimensions except the genotype one: $\frac{\sum_{a_s,z_s,t_s} \hat{p}_{a_s,z_s,t_s,g_s,c_s} \times N_{a_s,z_s,t_s,g_s}}{\sum_{a_s,z_s,t_s} N_{a_s,z_s,t_s,g_s}}$
- Step 4B. For each draw, compute the difference between the marginal expected prevalence under opportunistic and organised screening.

S6 Descriptive reporting for the analytical sample

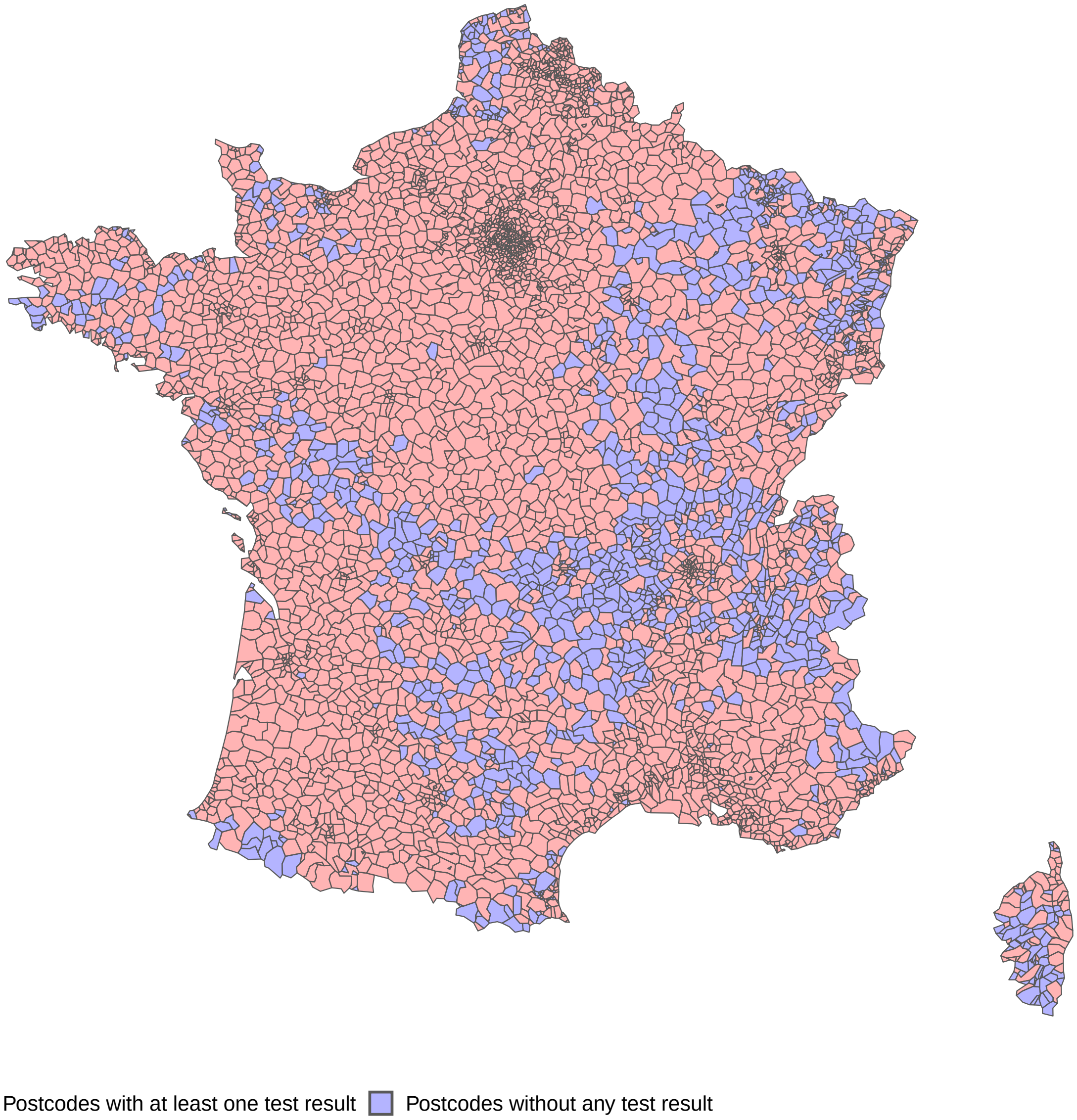

Figure S13 Postcodes with at least one test result observed in the analytical sample.

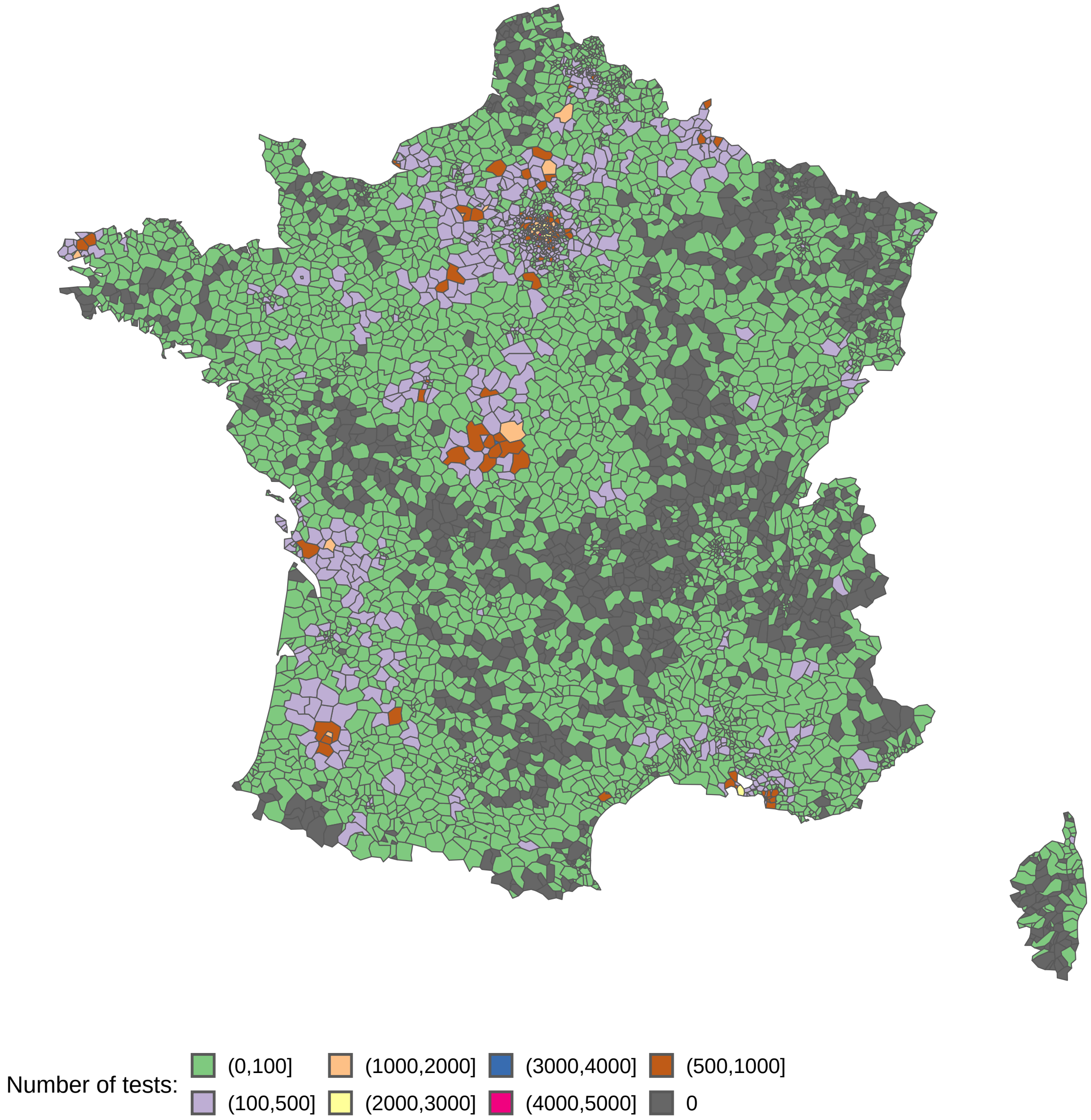

Figure S14 **Postcodes stratified by the number of HR HPV tests in the analytical sample.**

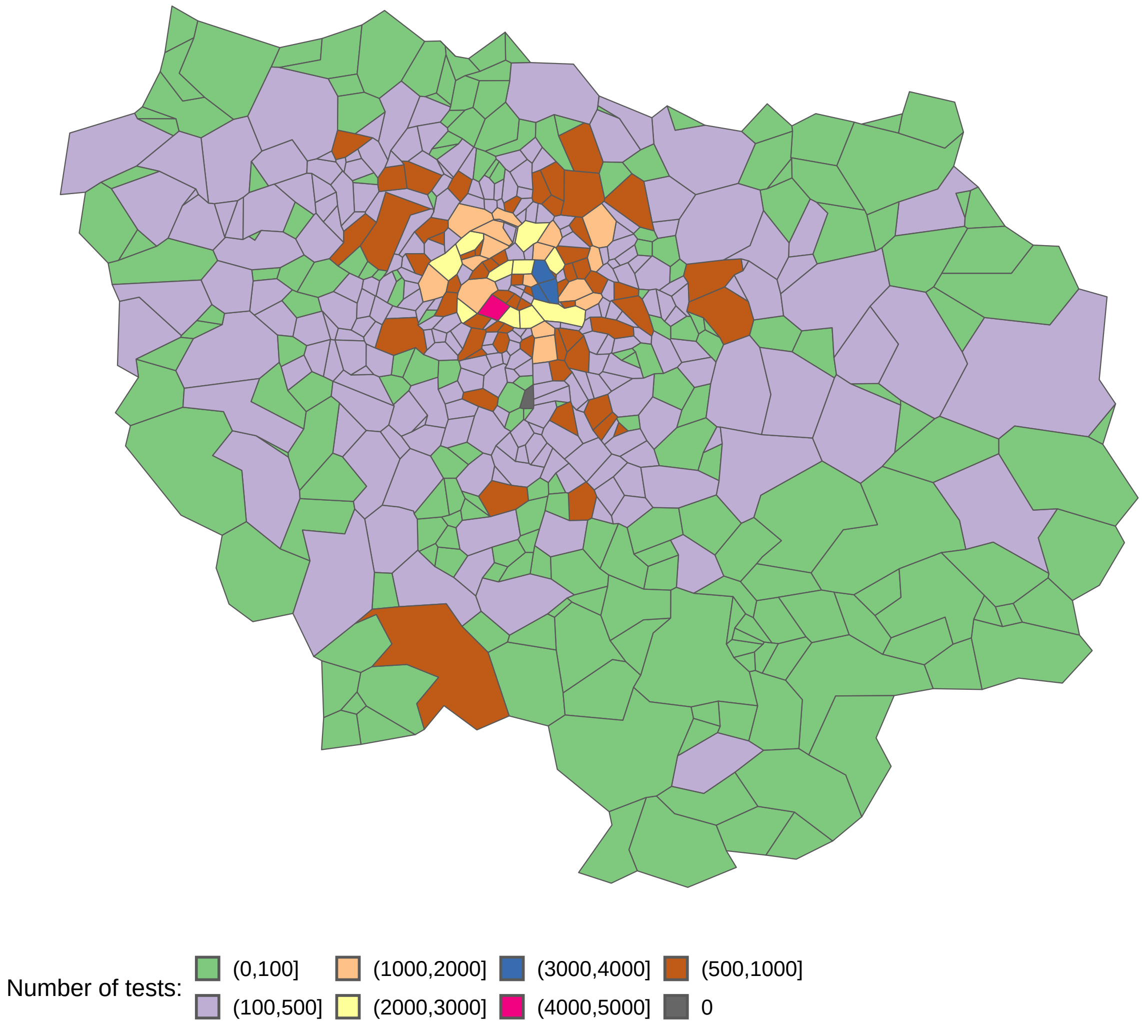

Figure S15 Postcodes stratified by the number of HR HPV tests in the analytical sample, zoom on the Paris region.

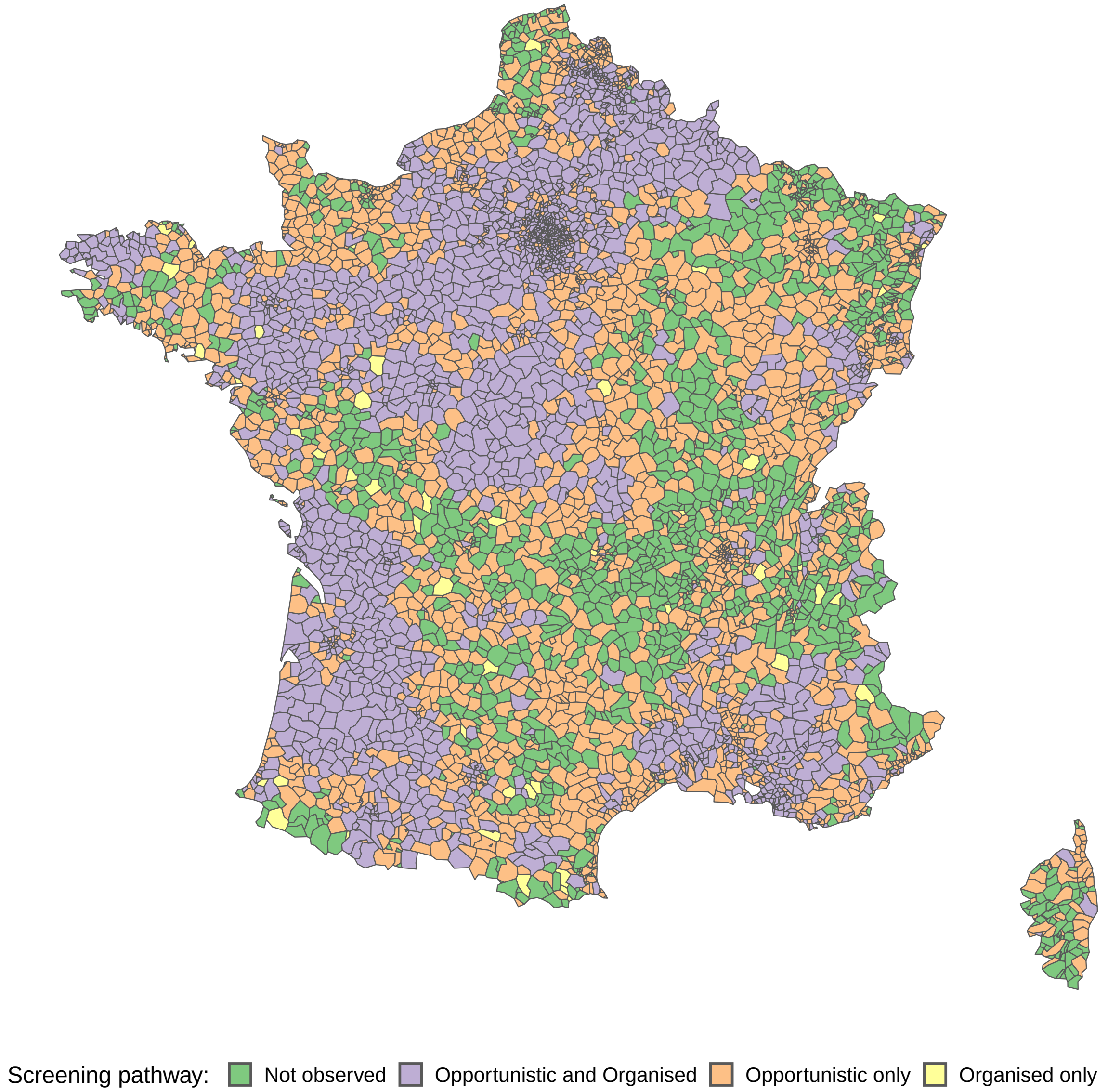

Figure S16 Postcodes only observed through opportunistic screening, organised screening, both opportunistic and organised screening, and not observed in the analytical sample.

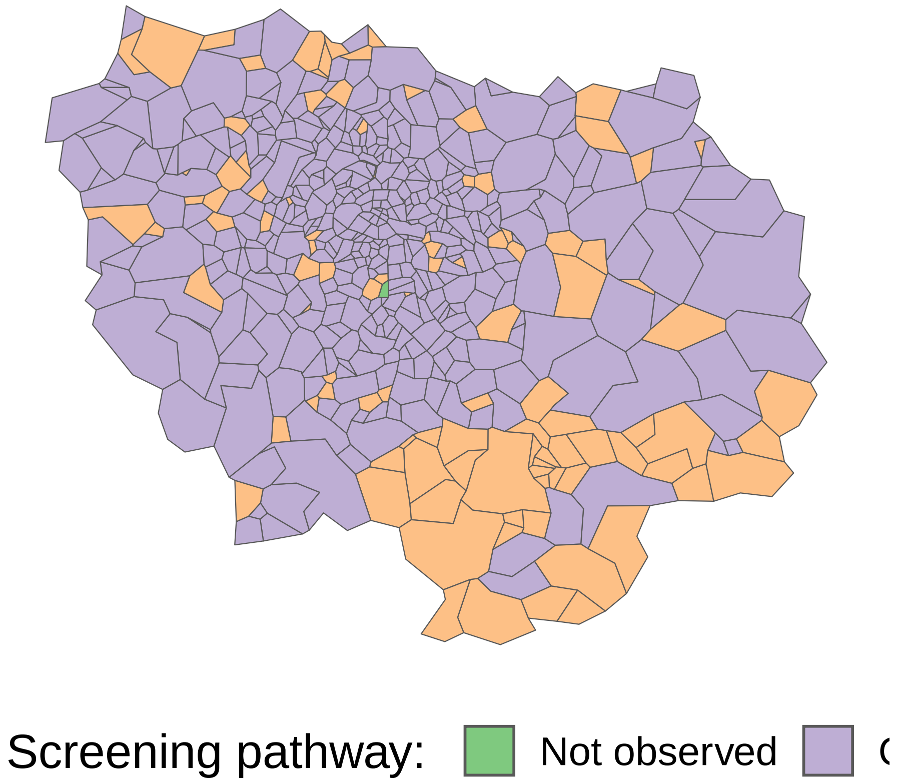

Figure S17 Postcodes only observed through opportunistic screening, organised screening, both opportunistic and organised screening, and not observed in the analytical sample, zoom on Paris region.

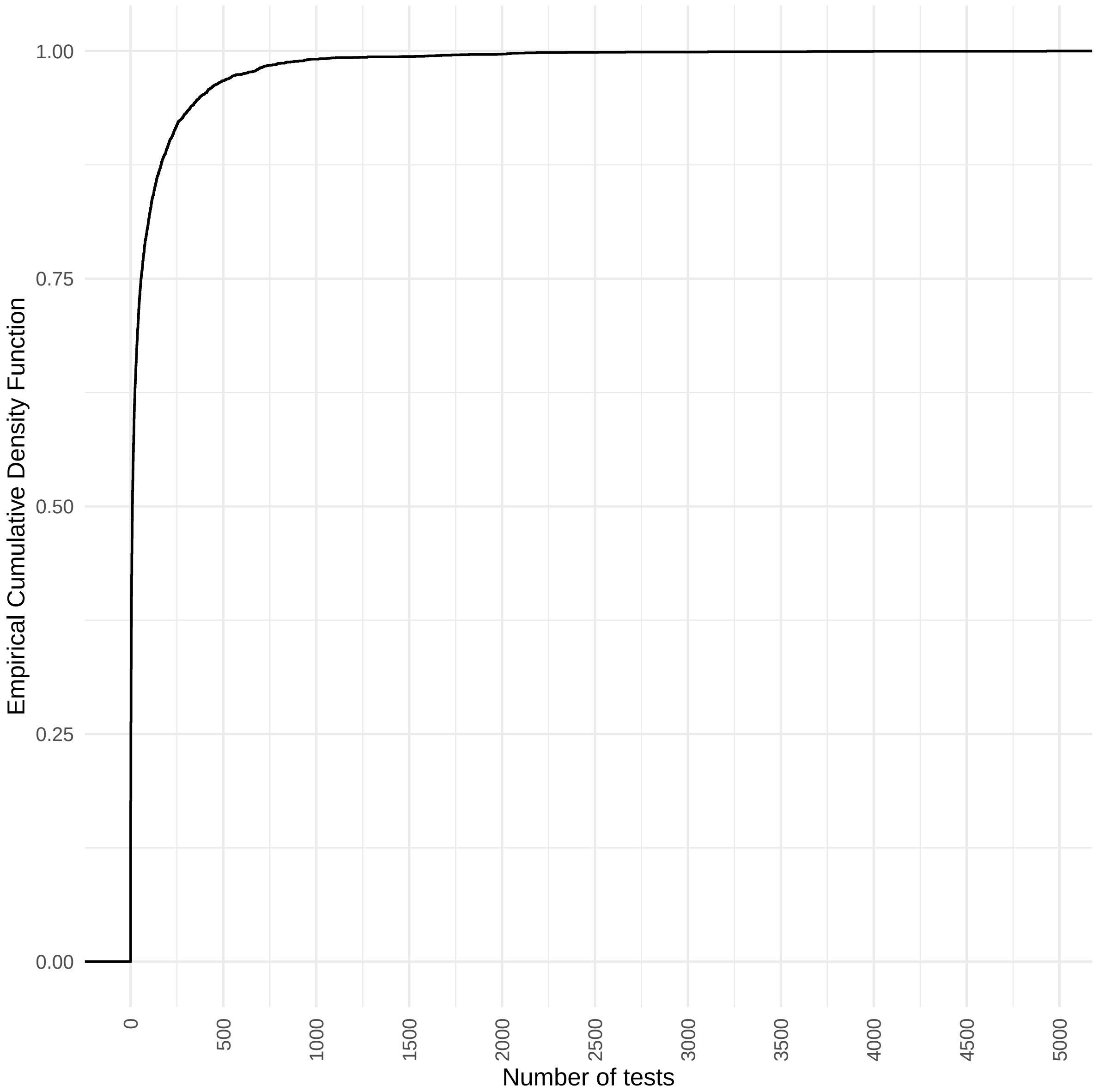

Figure S18 Empirical cumulative distribution function of the number of tests within postcodes in the analytical sample.

S7 Performance of the selected model

S7.1 Model selection and raw reporting of the selected model

Table S5 Logarithmic-score based on LOGO-CV. The model with the minimum score was selected.

Model	Type	Structure	Log-score
Model 5	BYM2	1st-order Queen	0.2351
Model 4	BYM2	SOI	0.2351
Model 3	BYM2	Delaunay	0.2351
Model 6	BYM2	2nd-order Queen	0.2351
Model 1	Matérn	Stationary	0.2352
Model 2	Matérn	Non-stationary (Barrier model)	0.2352

Table S6 Raw summary provided by `R-INLA` of the posterior distribution for the latent parameters.

	Description of the posterior distribution					
			Quantile			
Parameters	Average	SD	0.025	0.5	0.975	Mode
ψ_0	-3.48	0.05	-3.58	-3.48	-3.38	-3.48
ψ_1	0.88	0.05	0.76	0.88	0.98	0.90
ψ_2	0.23	0.05	0.13	0.23	0.32	0.23
ψ_3	0.04	0.06	-0.07	0.03	0.15	0.02

Table S7 **Raw summary provided by `R-INLA` of the posterior distribution for the hyperparameters.**

	Description of the posterior distribution					
			Quantile			
Parameters	Average	SD	0.025	0.5	0.975	Mode
σ^{-2} for ψ_1	1.63	3.08	0.04	0.71	8.82	0.08
σ^{-2} for ψ_2	20.50	104.01	0.03	3.06	146.30	0.01
σ^{-2} for ψ_3	3,782.89	192,792.03	0.04	23.92	13,055.10	0.00
σ^{-2} for $\gamma_{AGE}(age_s, g_s)$	75.32	49.27	19.78	62.96	204.37	44.24
η for $\gamma_{AGE}(age_s, g_s)$	-0.21	0.41	-0.86	-0.26	0.63	-0.47
σ^{-2} for $\gamma'_{AGE}(age_s, g_s)$	4,445.98	21,985.49	33.97	874.46	30,422.39	59.18
η for $\gamma'_{AGE}(age_s, g_s)$	0.12	0.67	-0.97	0.22	0.99	0.99
σ^{-2} for $\alpha_{SPACE}(z_s, g_s)$	15.44	2.44	11.07	15.29	20.67	15.06
ϕ for $\alpha_{SPACE}(z_s, g_s)$	0.95	0.05	0.82	0.96	0.99	0.98
η for $\alpha_{SPACE}(z_s, g_s)$	0.95	0.03	0.89	0.96	0.99	0.97
σ^{-2} for $\alpha'_{SPACE}(z_s, g_s)$	82.27	46.95	27.33	70.98	205.00	53.72
ϕ for $\alpha'_{SPACE}(z_s, g_s)$	0.80	0.16	0.38	0.84	0.98	0.95
η for $\alpha'_{SPACE}(z_s, g_s)$	0.91	0.10	0.62	0.94	0.99	0.98
σ^{-2} for $\beta_{TIME}(t_s, g_s)$	1,018.77	4,944.59	5.73	197.67	6,991.05	7.90
η for $\beta_{TIME}(t_s, g_s)$	0.13	0.62	-0.94	0.21	0.98	0.97
σ^{-2} for $\beta'_{TIME}(t_s, g_s)$	323.38	814.88	7.28	118.44	1,929.37	15.94
η for $\beta'_{TIME}(t_s, g_s)$	-0.04	0.63	-0.96	-0.06	0.96	-0.95

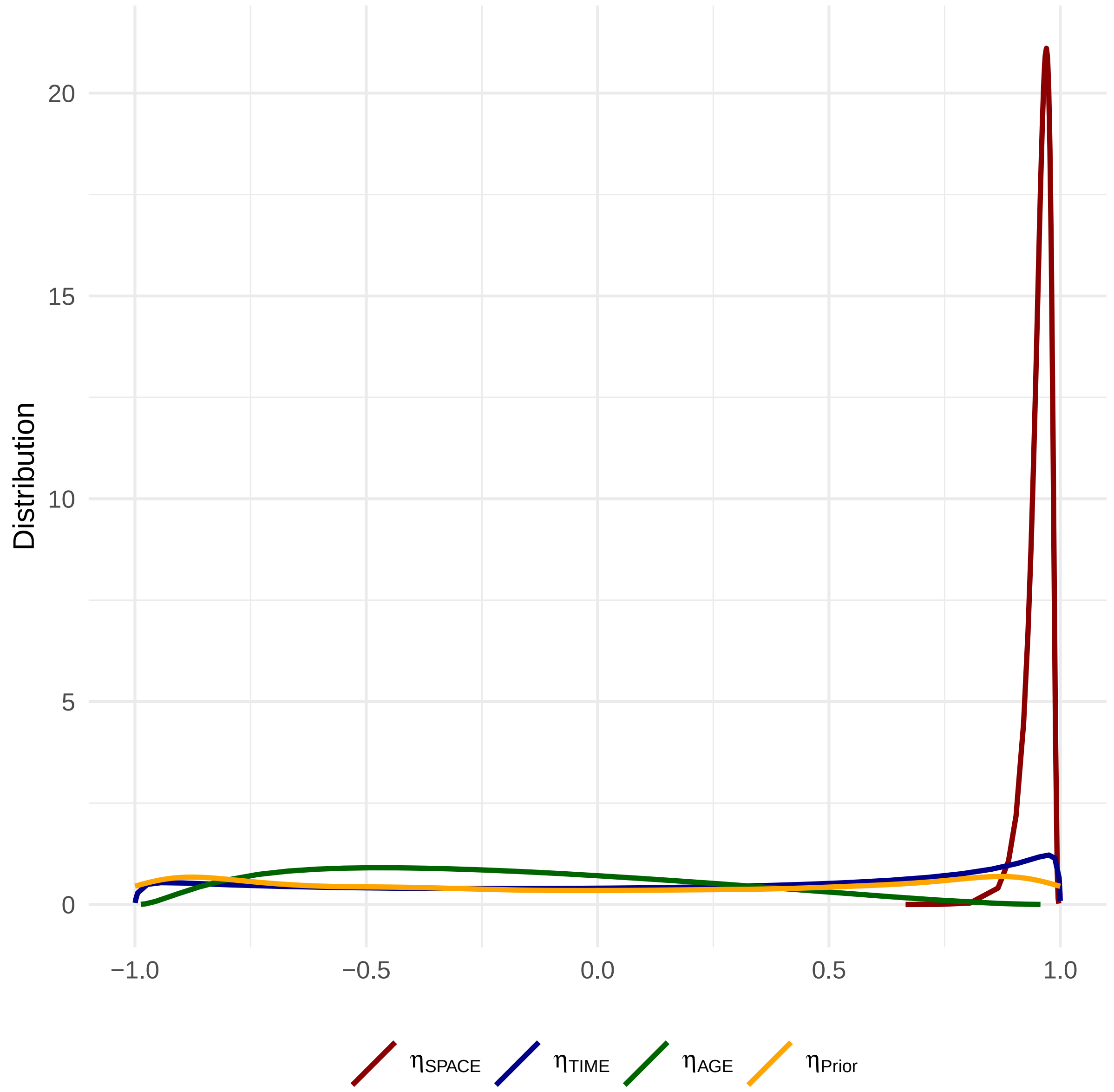

Figure S19 Posterior and prior distribution for the correlations between the two groups of genotypes for components specific to organised screening.

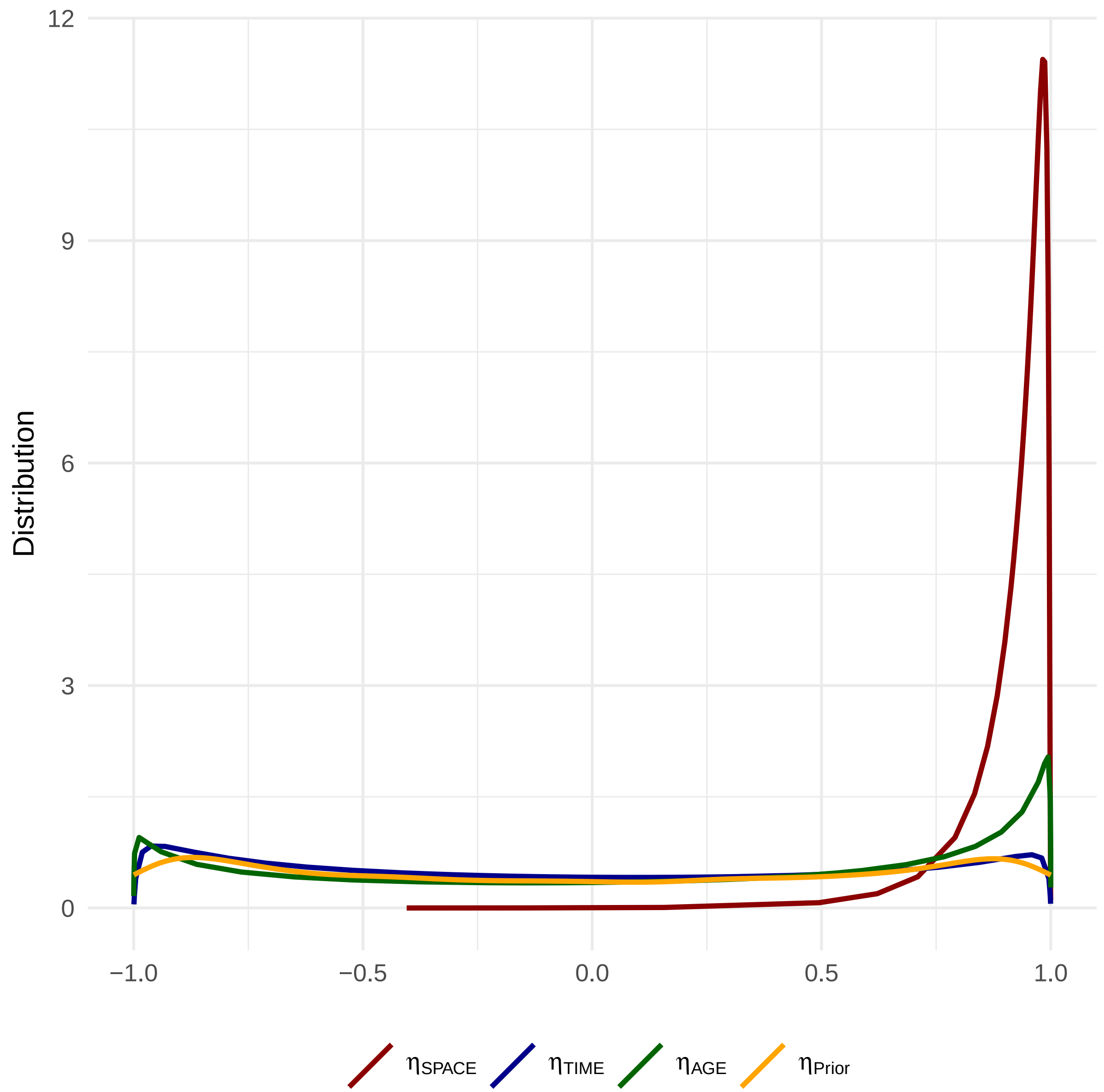

Figure S20 **Posterior and prior distribution for the correlations between the two groups of genotypes for components specific to opportunistic screening.**

S7.2 Posterior predictive check from the selected model

Table S8 Observed HR HPV cervical infection prevalence, posterior predictive HR HPV cervical infection prevalence, and posterior expected HR HPV cervical infection prevalence, stratified by various dimensions.

							Posterior distribution							
							Predictive prevalence				Expected prevalence			
									ETI95%				ETI95%	
Region	Genotypes	Screening pathway	Year	Age	Number of tests	Observed	Average	Median	LB	UB	Average	Median	LB	UB
	HPV16/18				362,963	3.70	3.72	3.73	3.64	3.81	3.72	3.72	3.66	3.78
	Other genotypes				362,963	9.23	9.27	9.27	9.14	9.41	9.27	9.27	9.18	9.37
	HPV16/18		2020		71,081	3.12	3.19	3.19	3.02	3.38	3.19	3.19	3.07	3.32
	HPV16/18		2021		159,182	3.65	3.64	3.64	3.51	3.77	3.64	3.64	3.55	3.73
	HPV16/18		2022		72,265	3.89	3.93	3.93	3.75	4.12	3.93	3.93	3.80	4.06
	HPV16/18		2023		60,435	4.32	4.33	4.33	4.11	4.55	4.33	4.33	4.18	4.49
	Other genotypes		2020		71,081	9.36	9.44	9.44	9.15	9.74	9.44	9.44	9.24	9.65
	Other genotypes		2021		159,182	8.75	8.77	8.77	8.58	8.95	8.77	8.77	8.64	8.91
	Other genotypes		2022		72,265	9.08	9.12	9.12	8.84	9.41	9.12	9.12	8.93	9.32
	Other genotypes		2023		60,435	10.49	10.57	10.57	10.24	10.92	10.58	10.58	10.35	10.82
	HPV16/18			[30,39]			4.54	4.55	4.39	4.70	4.54	4.55	4.44	4.65
	HPV16/18			(39,49]	104,989	3.68	3.71	3.70	3.55	3.86	3.70	3.70	3.60	3.82
	HPV16/18			(49,59]	83,824	2.91	2.95	2.95	2.80	3.12	2.95	2.95	2.85	3.06
	HPV16/18			(59,66]	42,356	2.77	2.75	2.74	2.54	2.96	2.75	2.75	2.60	2.89
	Other genotypes			[30,39]			12.16	12.15	11.91	12.40	12.16	12.16	11.98	12.33
	Other genotypes			(39,49]	104,989	8.42	8.49	8.49	8.26	8.72	8.49	8.49	8.32	8.65
	Other genotypes			(49,59]	83,824	7.14	7.18	7.17	6.94	7.42	7.18	7.18	7.02	7.35
	Other genotypes			(59,66]	42,356	6.36	6.38	6.38	6.05	6.71	6.38	6.38	6.16	6.61
Auvergne-Rhône-Alpes	HPV16/18				4,671	3.81	4.01	4.00	3.38	4.71	4.02	4.01	3.69	4.35
Bourgogne-Franche-Comté	HPV16/18				4,035	3.64	3.67	3.67	3.02	4.36	3.66	3.66	3.33	4.03
Bretagne	HPV16/18				11,062	3.88	3.87	3.87	3.42	4.36	3.87	3.87	3.58	4.19
Centre-Val de Loire	HPV16/18				32,749	3.70	3.59	3.59	3.34	3.86	3.59	3.59	3.44	3.76
Corse	HPV16/18				430	3.49	3.43	3.49	1.63	5.35	3.40	3.38	2.71	4.25
Grand Est	HPV16/18				11,886	3.58	3.60	3.59	3.18	4.05	3.59	3.59	3.35	3.85
Hauts-de-France	HPV16/18				31,938	3.72	3.74	3.73	3.48	4.02	3.74	3.74	3.57	3.91
Normandie	HPV16/18				19,963	4.32	4.15	4.15	3.79	4.52	4.15	4.15	3.92	4.38
Nouvelle-Aquitaine	HPV16/18				30,721	3.76	3.81	3.81	3.54	4.10	3.82	3.82	3.64	4.00
Occitanie	HPV16/18				12,524	4.07	4.09	4.08	3.66	4.51	4.09	4.09	3.84	4.34
Pays de la Loire	HPV16/18				6,705	2.98	3.14	3.13	2.67	3.64	3.14	3.14	2.89	3.40
Provence-Alpes-Côte d'Azur	HPV16/18				27,889	4.11	4.12	4.12	3.81	4.44	4.12	4.12	3.92	4.34
Île-de-France	HPV16/18				168,390	3.55	3.61	3.61	3.48	3.73	3.60	3.60	3.52	3.69
Auvergne-Rhône-Alpes	Other genotypes				4,671	9.98	9.90	9.89	8.82	10.98	9.90	9.89	9.24	10.59
Bourgogne-Franche-Comté	Other genotypes				4,035	9.59	9.00	9.00	7.88	10.14	9.00	9.00	8.35	9.73
Bretagne	Other genotypes				11,062	8.82	8.83	8.81	8.14	9.56	8.82	8.82	8.35	9.31
Centre-Val de Loire	Other genotypes				32,749	8.18	8.34	8.34	7.94	8.73	8.34	8.34	8.07	8.61
Corse	Other genotypes				430	10.93	10.13	10.00	6.98	13.72	10.11	10.07	8.35	12.22

Table S8 Observed HR HPV cervical infection prevalence, posterior predictive HR HPV cervical infection prevalence, and posterior expected HR HPV cervical infection prevalence, stratified by various dimensions. ***(continued)***

							Posterior distribution							
							Predictive prevalence				Expected prevalence			
									ETI95%				ETI95%	
Region	Genotypes	Screening pathway	Year	Age	Number of tests	Observed	Average	Median	LB	UB	Average	Median	LB	UB
Grand Est	Other genotypes				11,886	8.51	8.61	8.61	7.93	9.30	8.61	8.60	8.18	9.08
Hauts-de-France	Other genotypes				31,938	8.80	8.91	8.91	8.49	9.33	8.91	8.92	8.63	9.20
Normandie	Other genotypes				19,963	8.97	9.22	9.22	8.66	9.76	9.22	9.22	8.86	9.59
Nouvelle-Aquitaine	Other genotypes				30,721	9.22	9.20	9.20	8.77	9.65	9.20	9.20	8.92	9.51
Occitanie	Other genotypes				12,524	9.85	9.86	9.86	9.17	10.54	9.86	9.86	9.42	10.33
Pays de la Loire	Other genotypes				6,705	7.05	7.36	7.35	6.58	8.16	7.36	7.35	6.90	7.86
Provence-Alpes-Côte d'Azur	Other genotypes				27,889	9.82	9.88	9.87	9.41	10.36	9.88	9.88	9.55	10.21
Île-de-France	Other genotypes				168,390	9.53	9.53	9.53	9.34	9.73	9.53	9.53	9.40	9.68
	HPV16/18	Opportunistic			343,042	3.75	3.77	3.77	3.68	3.86	3.77	3.77	3.71	3.83
	HPV16/18	Organised			19,921	2.89	2.90	2.91	2.58	3.22	2.91	2.91	2.69	3.14
	Other genotypes	Opportunistic			343,042	9.36	9.41	9.41	9.27	9.55	9.41	9.41	9.31	9.50
	Other genotypes	Organised			19,921	6.89	6.93	6.92	6.46	7.39	6.94	6.94	6.59	7.28
	HPV16/18		2020	[30,39]			3.86	3.86	3.59	4.15	3.86	3.86	3.70	4.03
	HPV16/18		2020	(39,49]	21,620	2.86	3.17	3.17	2.90	3.46	3.17	3.17	3.03	3.32
	HPV16/18		2020	(49,59]	16,498	2.36	2.53	2.53	2.27	2.81	2.53	2.53	2.41	2.66
	HPV16/18		2020	(59,66]	7,086	2.27	2.35	2.34	1.98	2.75	2.35	2.35	2.20	2.50
	HPV16/18		2021	[30,39]			4.45	4.45	4.23	4.67	4.45	4.45	4.32	4.57
	HPV16/18		2021	(39,49]	46,281	3.54	3.64	3.64	3.44	3.85	3.64	3.64	3.52	3.77
	HPV16/18		2021	(49,59]	36,867	2.82	2.90	2.90	2.70	3.12	2.91	2.90	2.79	3.02
	HPV16/18		2021	(59,66]	19,728	2.69	2.70	2.70	2.44	2.99	2.70	2.70	2.55	2.86
	HPV16/18		2022	[30,39]			4.79	4.79	4.48	5.09	4.79	4.79	4.61	4.97
	HPV16/18		2022	(39,49]	20,354	4.08	3.91	3.91	3.59	4.24	3.91	3.91	3.75	4.07
	HPV16/18		2022	(49,59]	16,678	2.99	3.10	3.10	2.80	3.39	3.10	3.09	2.96	3.24
	HPV16/18		2022	(59,66]	8,295	2.83	2.86	2.86	2.47	3.25	2.86	2.85	2.68	3.03
	HPV16/18		2023	[30,39]			5.27	5.27	4.93	5.64	5.28	5.28	5.08	5.48
	HPV16/18		2023	(39,49]	16,734	4.63	4.32	4.32	3.98	4.68	4.32	4.32	4.14	4.52
	HPV16/18		2023	(49,59]	13,781	3.74	3.43	3.43	3.09	3.77	3.43	3.43	3.26	3.60
	HPV16/18		2023	(59,66]	7,247	3.38	3.13	3.12	2.69	3.59	3.13	3.13	2.93	3.34
	Other genotypes		2020	[30,39]			12.29	12.29	11.81	12.81	12.30	12.30	12.01	12.59
	Other genotypes		2020	(39,49]	21,620	8.41	8.64	8.64	8.19	9.08	8.64	8.64	8.41	8.87
	Other genotypes		2020	(49,59]	16,498	7.18	7.30	7.29	6.86	7.77	7.30	7.29	7.08	7.53
	Other genotypes		2020	(59,66]	7,086	5.91	6.48	6.48	5.86	7.11	6.47	6.47	6.21	6.74
	Other genotypes		2021	[30,39]			11.53	11.53	11.19	11.88	11.53	11.53	11.32	11.75
	Other genotypes		2021	(39,49]	46,281	8.01	8.09	8.09	7.80	8.40	8.09	8.09	7.91	8.27
	Other genotypes		2021	(49,59]	36,867	6.69	6.83	6.83	6.52	7.14	6.83	6.83	6.66	7.01
	Other genotypes		2021	(59,66]	19,728	6.03	6.08	6.08	5.68	6.48	6.08	6.08	5.86	6.31
	Other genotypes		2022	[30,39]			11.92	11.92	11.44	12.39	11.91	11.91	11.64	12.19
	Other genotypes		2022	(39,49]	20,354	8.24	8.30	8.29	7.86	8.74	8.30	8.30	8.08	8.53
	Other genotypes		2022	(49,59]	16,678	7.25	7.02	7.02	6.60	7.49	7.03	7.03	6.82	7.24

Table S8 Observed HR HPV cervical infection prevalence, posterior predictive HR HPV cervical infection prevalence, and posterior expected HR HPV cervical infection prevalence, stratified by various dimensions. ***(continued)***

							Posterior distribution							
							Predictive prevalence				Expected prevalence			
									ETI95%				ETI95%	
Region	Genotypes	Screening pathway	Year	Age	Number of tests	Observed	Average	Median	LB	UB	Average	Median	LB	UB
	Other genotypes		2022	(59,66]	8,295	6.04	6.29	6.29	5.74	6.91	6.29	6.29	6.03	6.55
	Other genotypes		2023	[30,39]			13.84	13.84	13.30	14.39	13.84	13.84	13.51	14.17
	Other genotypes		2023	(39,49]	16,734	9.75	9.62	9.62	9.11	10.14	9.62	9.62	9.35	9.89
	Other genotypes		2023	(49,59]	13,781	8.20	8.15	8.14	7.64	8.69	8.15	8.15	7.91	8.42
	Other genotypes		2023	(59,66]	7,247	8.06	7.20	7.19	6.55	7.85	7.20	7.20	6.91	7.50
Auvergne-Rhône-Alpes	HPV16/18		2020		988	3.44	3.46	3.44	2.33	4.76	3.48	3.47	3.14	3.83
Bourgogne-Franche-Comté	HPV16/18		2020		537	2.98	3.08	2.98	1.68	4.66	3.05	3.04	2.73	3.39
Bretagne	HPV16/18		2020		559	3.76	3.24	3.22	1.79	4.83	3.23	3.23	2.88	3.62
Centre-Val de Loire	HPV16/18		2020		3,670	2.64	3.07	3.08	2.51	3.68	3.08	3.07	2.89	3.27
Corse	HPV16/18		2020		377	3.45	3.36	3.18	1.59	5.57	3.32	3.30	2.55	4.28
Grand Est	HPV16/18		2020		882	3.17	2.77	2.72	1.70	3.97	2.77	2.77	2.48	3.08
Hauts-de-France	HPV16/18		2020		2,840	3.49	3.01	2.99	2.36	3.70	3.02	3.01	2.83	3.21
Normandie	HPV16/18		2020		2,313	3.67	3.30	3.29	2.55	4.11	3.30	3.30	3.06	3.54
Nouvelle-Aquitaine	HPV16/18		2020		1,689	3.37	3.16	3.14	2.31	4.09	3.16	3.16	2.95	3.38
Occitanie	HPV16/18		2020		2,179	4.22	3.91	3.90	3.03	4.82	3.91	3.91	3.55	4.31
Pays de la Loire	HPV16/18		2020		1,161	1.72	2.65	2.67	1.72	3.62	2.67	2.66	2.40	2.96
Provence-Alpes-Côte d'Azur	HPV16/18		2020		2,131	3.10	3.24	3.24	2.49	4.08	3.24	3.23	3.00	3.49
Île-de-France	HPV16/18		2020		51,755	3.07	3.19	3.18	3.00	3.39	3.19	3.19	3.06	3.32
Auvergne-Rhône-Alpes	HPV16/18		2021		3,147	3.37	4.03	4.04	3.30	4.80	4.04	4.03	3.70	4.38
Bourgogne-Franche-Comté	HPV16/18		2021		1,537	3.97	3.58	3.58	2.67	4.62	3.58	3.58	3.24	3.95
Bretagne	HPV16/18		2021		2,374	3.45	3.60	3.62	2.82	4.42	3.60	3.59	3.27	3.95
Centre-Val de Loire	HPV16/18		2021		15,029	3.35	3.47	3.47	3.14	3.81	3.47	3.47	3.31	3.66
Corse	HPV16/18		2021		35	2.86	3.73	2.86	0.00	11.43	3.77	3.76	3.27	4.35
Grand Est	HPV16/18		2021		3,923	3.14	3.35	3.34	2.75	3.98	3.34	3.33	3.10	3.58
Hauts-de-France	HPV16/18		2021		10,748	3.67	3.55	3.54	3.16	3.94	3.55	3.55	3.37	3.73
Normandie	HPV16/18		2021		8,079	4.30	3.93	3.94	3.44	4.43	3.93	3.93	3.69	4.17
Nouvelle-Aquitaine	HPV16/18		2021		8,917	3.51	3.54	3.54	3.12	3.99	3.54	3.54	3.36	3.74
Occitanie	HPV16/18		2021		5,839	4.01	4.06	4.06	3.49	4.66	4.07	4.07	3.81	4.34
Pays de la Loire	HPV16/18		2021		3,081	3.21	3.06	3.05	2.43	3.77	3.06	3.06	2.78	3.34
Provence-Alpes-Côte d'Azur	HPV16/18		2021		11,313	3.81	3.88	3.88	3.47	4.31	3.88	3.88	3.67	4.11
Île-de-France	HPV16/18		2021		85,160	3.65	3.62	3.62	3.46	3.79	3.62	3.62	3.52	3.73
Auvergne-Rhône-Alpes	HPV16/18		2022		295	8.47	4.63	4.41	2.37	7.12	4.62	4.61	4.13	5.18
Bourgogne-Franche-Comté	HPV16/18		2022		1,220	3.36	3.90	3.85	2.79	5.16	3.89	3.89	3.51	4.33
Bretagne	HPV16/18		2022		3,082	3.50	3.89	3.89	3.18	4.64	3.89	3.89	3.58	4.23
Centre-Val de Loire	HPV16/18		2022		7,977	4.25	3.63	3.64	3.18	4.10	3.64	3.64	3.45	3.85
Corse	HPV16/18		2022		13	0.00	4.44	0.00	0.00	15.38	4.33	4.31	3.69	5.09
Grand Est	HPV16/18		2022		3,704	3.75	3.66	3.64	3.00	4.37	3.66	3.66	3.37	3.95
Hauts-de-France	HPV16/18		2022		9,943	3.53	3.77	3.76	3.34	4.20	3.77	3.77	3.57	3.98
Normandie	HPV16/18		2022		5,912	4.35	4.39	4.38	3.82	4.99	4.38	4.38	4.11	4.66

Table S8 Observed HR HPV cervical infection prevalence, posterior predictive HR HPV cervical infection prevalence, and posterior expected HR HPV cervical infection prevalence, stratified by various dimensions. ***(continued)***

							Posterior distribution							
							Predictive prevalence				Expected prevalence			
									ETI95%				ETI95%	
Region	Genotypes	Screening pathway	Year	Age	Number of tests	Observed	Average	Median	LB	UB	Average	Median	LB	UB
Nouvelle-Aquitaine	HPV16/18		2022		10,965	3.80	3.79	3.78	3.39	4.22	3.79	3.79	3.58	3.99
Occitanie	HPV16/18		2022		3,131	3.93	4.08	4.09	3.35	4.85	4.08	4.08	3.79	4.37
Pays de la Loire	HPV16/18		2022		962	2.49	3.40	3.33	2.29	4.57	3.37	3.36	3.08	3.71
Provence-Alpes-Côte d'Azur	HPV16/18		2022		8,238	4.44	4.24	4.24	3.74	4.76	4.24	4.24	4.00	4.49
Île-de-France	HPV16/18		2022		16,823	3.67	3.99	3.99	3.64	4.32	3.99	3.99	3.81	4.17
Auvergne-Rhône-Alpes	HPV16/18		2023		241	5.39	5.21	4.98	2.49	8.30	5.23	5.21	4.50	6.07
Bourgogne-Franche-Comté	HPV16/18		2023		741	3.91	3.92	3.91	2.43	5.40	3.91	3.90	3.45	4.42
Bretagne	HPV16/18		2023		5,047	4.32	4.06	4.06	3.45	4.72	4.06	4.05	3.73	4.41
Centre-Val de Loire	HPV16/18		2023		6,073	4.46	4.13	4.12	3.59	4.69	4.13	4.13	3.90	4.37
Corse	HPV16/18		2023		5	20.00	3.77	0.00	0.00	20.00	4.08	4.02	3.08	5.35
Grand Est	HPV16/18		2023		3,377	4.03	4.03	4.03	3.32	4.77	4.03	4.03	3.72	4.35
Hauts-de-France	HPV16/18		2023		8,407	4.09	4.19	4.19	3.71	4.67	4.19	4.19	3.96	4.43
Normandie	HPV16/18		2023		3,659	4.73	4.80	4.78	4.04	5.60	4.81	4.81	4.49	5.13
Nouvelle-Aquitaine	HPV16/18		2023		9,150	4.02	4.23	4.23	3.77	4.71	4.23	4.23	4.00	4.47
Occitanie	HPV16/18		2023		1,375	4.44	4.48	4.51	3.27	5.75	4.49	4.49	4.12	4.88
Pays de la Loire	HPV16/18		2023		1,501	3.80	3.51	3.46	2.60	4.53	3.52	3.52	3.19	3.89
Provence-Alpes-Côte d'Azur	HPV16/18		2023		6,207	4.58	4.70	4.70	4.14	5.28	4.70	4.70	4.42	4.99
Île-de-France	HPV16/18		2023		14,652	4.49	4.53	4.52	4.13	4.93	4.52	4.53	4.32	4.74
Auvergne-Rhône-Alpes	Other genotypes		2020		988	9.11	10.01	10.02	8.00	12.15	10.01	10.00	9.25	10.80
Bourgogne-Franche-Comté	Other genotypes		2020		537	9.50	8.73	8.75	6.33	11.17	8.74	8.73	8.04	9.50
Bretagne	Other genotypes		2020		559	8.77	8.59	8.59	6.26	11.09	8.61	8.61	7.87	9.40
Centre-Val de Loire	Other genotypes		2020		3,670	7.08	8.40	8.39	7.41	9.37	8.39	8.39	8.03	8.76
Corse	Other genotypes		2020		377	10.61	10.26	10.08	6.90	14.06	10.24	10.19	8.28	12.60
Grand Est	Other genotypes		2020		882	7.82	8.00	7.94	6.24	9.98	8.01	8.00	7.32	8.75
Hauts-de-France	Other genotypes		2020		2,840	8.06	8.56	8.56	7.46	9.65	8.55	8.55	8.17	8.94
Normandie	Other genotypes		2020		2,313	9.21	8.80	8.78	7.57	10.07	8.80	8.80	8.35	9.27
Nouvelle-Aquitaine	Other genotypes		2020		1,689	8.94	9.15	9.12	7.76	10.60	9.16	9.16	8.77	9.57
Occitanie	Other genotypes		2020		2,179	9.91	10.92	10.92	9.45	12.44	10.92	10.91	10.13	11.72
Pays de la Loire	Other genotypes		2020		1,161	6.63	7.25	7.24	5.68	8.96	7.26	7.25	6.65	7.92
Provence-Alpes-Côte d'Azur	Other genotypes		2020		2,131	9.62	9.41	9.39	8.12	10.75	9.41	9.41	8.91	9.92
Île-de-France	Other genotypes		2020		51,755	9.67	9.62	9.61	9.29	9.96	9.62	9.61	9.41	9.84
Auvergne-Rhône-Alpes	Other genotypes		2021		3,147	9.12	9.56	9.53	8.33	10.80	9.56	9.55	8.91	10.26
Bourgogne-Franche-Comté	Other genotypes		2021		1,537	9.24	8.56	8.52	6.96	10.21	8.55	8.55	7.91	9.25
Bretagne	Other genotypes		2021		2,374	7.03	7.97	7.96	6.74	9.22	7.94	7.94	7.40	8.49
Centre-Val de Loire	Other genotypes		2021		15,029	7.58	7.85	7.84	7.36	8.35	7.85	7.84	7.57	8.13
Corse	Other genotypes		2021		35	8.57	9.00	8.57	0.00	20.00	9.07	9.05	7.95	10.37
Grand Est	Other genotypes		2021		3,923	6.96	7.79	7.77	6.91	8.74	7.79	7.78	7.37	8.24
Hauts-de-France	Other genotypes		2021		10,748	7.75	8.27	8.27	7.68	8.87	8.27	8.27	7.99	8.58
Normandie	Other genotypes		2021		8,079	8.39	8.58	8.59	7.88	9.28	8.58	8.58	8.22	8.95

Table S8 **Observed HR HPV cervical infection prevalence, posterior predictive HR HPV cervical infection prevalence, and posterior expected HR HPV cervical infection prevalence, stratified by various dimensions.** ***(continued)***

							Posterior distribution							
							Predictive prevalence				Expected prevalence			
									ETI95%				ETI95%	
Region	Genotypes	Screening pathway	Year	Age	Number of tests	Observed	Average	Median	LB	UB	Average	Median	LB	UB
Nouvelle-Aquitaine	Other genotypes		2021		8,917	8.32	8.39	8.39	7.74	9.05	8.39	8.39	8.09	8.72
Occitanie	Other genotypes		2021		5,839	9.71	9.46	9.45	8.58	10.34	9.47	9.46	9.00	9.95
Pays de la Loire	Other genotypes		2021		3,081	6.04	6.87	6.88	5.87	7.89	6.87	6.87	6.39	7.40
Provence-Alpes-Côte d'Azur	Other genotypes		2021		11,313	9.15	9.14	9.13	8.53	9.79	9.13	9.13	8.79	9.49
Île-de-France	Other genotypes		2021		85,160	9.25	9.06	9.06	8.82	9.31	9.06	9.06	8.90	9.23
Auvergne-Rhône-Alpes	Other genotypes		2022		295	18.31	10.72	10.51	7.12	14.58	10.76	10.75	9.75	11.78
Bourgogne-Franche-Comté	Other genotypes		2022		1,220	9.18	9.19	9.18	7.46	11.07	9.20	9.19	8.48	9.98
Bretagne	Other genotypes		2022		3,082	8.70	8.58	8.60	7.50	9.73	8.58	8.58	8.06	9.09
Centre-Val de Loire	Other genotypes		2022		7,977	8.65	8.16	8.16	7.47	8.83	8.15	8.15	7.83	8.47
Corse	Other genotypes		2022		13	23.08	9.62	7.69	0.00	30.77	9.43	9.40	8.13	10.83
Grand Est	Other genotypes		2022		3,704	9.10	8.53	8.50	7.51	9.58	8.53	8.52	8.06	9.04
Hauts-de-France	Other genotypes		2022		9,943	8.90	8.71	8.72	8.09	9.34	8.71	8.71	8.40	9.03
Normandie	Other genotypes		2022		5,912	9.08	9.38	9.37	8.51	10.28	9.37	9.37	8.95	9.78
Nouvelle-Aquitaine	Other genotypes		2022		10,965	8.75	8.88	8.88	8.27	9.51	8.89	8.88	8.58	9.21
Occitanie	Other genotypes		2022		3,131	9.55	9.40	9.39	8.27	10.57	9.39	9.39	8.89	9.94
Pays de la Loire	Other genotypes		2022		962	8.42	7.60	7.59	6.03	9.36	7.60	7.59	7.02	8.22
Provence-Alpes-Côte d'Azur	Other genotypes		2022		8,238	9.48	9.86	9.86	9.12	10.56	9.87	9.86	9.49	10.25
Île-de-France	Other genotypes		2022		16,823	9.27	9.76	9.76	9.23	10.29	9.76	9.76	9.49	10.03
Auvergne-Rhône-Alpes	Other genotypes		2023		241	14.52	12.80	12.86	8.30	17.43	12.77	12.77	11.18	14.45
Bourgogne-Franche-Comté	Other genotypes		2023		741	11.07	9.81	9.85	7.56	12.15	9.81	9.80	8.91	10.79
Bretagne	Other genotypes		2023		5,047	9.75	9.41	9.41	8.46	10.44	9.41	9.40	8.89	9.97
Centre-Val de Loire	Other genotypes		2023		6,073	9.70	9.76	9.76	8.94	10.60	9.78	9.78	9.41	10.18
Corse	Other genotypes		2023		5	20.00	8.98	0.00	0.00	40.00	9.04	8.94	6.97	11.58
Grand Est	Other genotypes		2023		3,377	9.86	9.81	9.83	8.68	10.96	9.80	9.80	9.25	10.40
Hauts-de-France	Other genotypes		2023		8,407	10.29	10.09	10.09	9.36	10.87	10.09	10.09	9.71	10.49
Normandie	Other genotypes		2023		3,659	9.92	10.64	10.63	9.54	11.78	10.65	10.65	10.16	11.15
Nouvelle-Aquitaine	Other genotypes		2023		9,150	10.73	10.39	10.37	9.68	11.13	10.38	10.38	10.02	10.77
Occitanie	Other genotypes		2023		1,375	11.05	10.92	10.91	9.16	12.73	10.92	10.91	10.24	11.67
Pays de la Loire	Other genotypes		2023		1,501	8.59	8.28	8.26	6.80	9.93	8.29	8.28	7.65	8.99
Provence-Alpes-Côte d'Azur	Other genotypes		2023		6,207	11.58	11.40	11.41	10.47	12.32	11.41	11.41	10.96	11.87
Île-de-France	Other genotypes		2023		14,652	10.92	11.72	11.72	11.12	12.37	11.73	11.72	11.40	12.07
	HPV16/18	Opportunistic	2020		71,043	3.11	3.19	3.19	3.02	3.38	3.19	3.19	3.07	3.32
	HPV16/18	Organised	2020		38	7.89	3.08	2.63	0.00	10.53	3.12	3.09	2.42	3.98
	HPV16/18	Opportunistic	2021		154,586	3.67	3.66	3.66	3.53	3.79	3.66	3.66	3.57	3.75
	HPV16/18	Organised	2021		4,596	2.87	2.91	2.92	2.31	3.59	2.91	2.90	2.51	3.36
	HPV16/18	Opportunistic	2022		64,519	4.02	4.05	4.05	3.85	4.26	4.05	4.05	3.91	4.19
	HPV16/18	Organised	2022		7,746	2.80	2.89	2.88	2.41	3.37	2.90	2.89	2.62	3.18
	HPV16/18	Opportunistic	2023		52,894	4.52	4.53	4.53	4.29	4.78	4.53	4.54	4.37	4.71
	HPV16/18	Organised	2023		7,541	2.96	2.92	2.90	2.43	3.47	2.92	2.92	2.58	3.27

Table S8 Observed HR HPV cervical infection prevalence, posterior predictive HR HPV cervical infection prevalence, and posterior expected HR HPV cervical infection prevalence, stratified by various dimensions. ***(continued)***

							Posterior distribution							
							Predictive prevalence				Expected prevalence			
									ETI95%				ETI95%	
Region	Genotypes	Screening pathway	Year	Age	Number of tests	Observed	Average	Median	LB	UB	Average	Median	LB	UB
	Other genotypes	Opportunistic	2020		71,043	9.37	9.44	9.44	9.15	9.74	9.44	9.44	9.24	9.66
	Other genotypes	Organised	2020		38	5.26	6.87	5.26	0.00	15.79	6.96	6.93	5.75	8.33
	Other genotypes	Opportunistic	2021		154,586	8.83	8.84	8.84	8.65	9.03	8.84	8.84	8.70	8.98
	Other genotypes	Organised	2021		4,596	6.24	6.42	6.40	5.53	7.38	6.42	6.42	5.82	7.07
	Other genotypes	Opportunistic	2022		64,519	9.31	9.38	9.38	9.07	9.69	9.38	9.37	9.17	9.58
	Other genotypes	Organised	2022		7,746	7.18	7.01	7.00	6.29	7.73	7.01	7.00	6.55	7.48
	Other genotypes	Opportunistic	2023		52,894	10.99	11.06	11.06	10.69	11.43	11.06	11.07	10.81	11.33
	Other genotypes	Organised	2023		7,541	6.99	7.15	7.15	6.42	7.93	7.17	7.17	6.65	7.72
Auvergne-Rhône-Alpes	HPV16/18			[30,39]			4.82	4.80	3.78	5.93	4.82	4.82	4.41	5.24
Bourgogne-Franche-Comté	HPV16/18			[30,39]			4.42	4.40	3.38	5.55	4.42	4.41	3.98	4.88
Bretagne	HPV16/18			[30,39]			4.79	4.80	4.07	5.58	4.80	4.79	4.42	5.20
Centre-Val de Loire	HPV16/18			[30,39]			4.48	4.48	4.02	4.95	4.48	4.48	4.28	4.71
Corse	HPV16/18			[30,39]			4.32	4.55	0.91	9.09	4.32	4.30	3.48	5.38
Grand Est	HPV16/18			[30,39]			4.43	4.44	3.72	5.14	4.42	4.42	4.12	4.76
Hauts-de-France	HPV16/18			[30,39]			4.40	4.40	4.01	4.82	4.40	4.40	4.18	4.62
Normandie	HPV16/18			[30,39]			5.08	5.07	4.50	5.67	5.08	5.08	4.78	5.38
Nouvelle-Aquitaine	HPV16/18			[30,39]			4.72	4.72	4.25	5.20	4.72	4.72	4.48	4.96
Occitanie	HPV16/18			[30,39]			5.04	5.04	4.30	5.81	5.04	5.04	4.72	5.36
Pays de la Loire	HPV16/18			[30,39]			3.82	3.82	3.00	4.68	3.81	3.81	3.50	4.14
Provence-Alpes-Côte d'Azur	HPV16/18			[30,39]			5.02	5.02	4.53	5.51	5.02	5.02	4.76	5.30
Île-de-France	HPV16/18			[30,39]			4.39	4.39	4.18	4.60	4.39	4.39	4.26	4.53
Auvergne-Rhône-Alpes	HPV16/18			(39,49]	1,258	3.66	3.95	3.97	2.86	5.09	3.96	3.96	3.63	4.31
Bourgogne-Franche-Comté	HPV16/18			(39,49]	1,096	3.19	3.64	3.65	2.55	4.93	3.62	3.62	3.27	4.02
Bretagne	HPV16/18			(39,49]	2,970	4.18	3.86	3.84	3.13	4.68	3.85	3.85	3.55	4.19
Centre-Val de Loire	HPV16/18			(39,49]	9,293	3.94	3.66	3.66	3.24	4.10	3.66	3.66	3.48	3.86
Corse	HPV16/18			(39,49]	129	4.65	3.61	3.10	0.78	6.98	3.55	3.53	2.76	4.53
Grand Est	HPV16/18			(39,49]	3,360	3.96	3.66	3.66	2.98	4.38	3.66	3.66	3.39	3.95
Hauts-de-France	HPV16/18			(39,49]	8,705	4.24	3.66	3.66	3.24	4.10	3.66	3.66	3.47	3.86
Normandie	HPV16/18			(39,49]	5,956	4.30	4.17	4.16	3.61	4.79	4.16	4.16	3.91	4.42
Nouvelle-Aquitaine	HPV16/18			(39,49]	8,585	3.84	3.88	3.88	3.41	4.36	3.88	3.88	3.67	4.09
Occitanie	HPV16/18			(39,49]	3,661	3.91	4.16	4.15	3.47	4.89	4.18	4.18	3.90	4.45
Pays de la Loire	HPV16/18			(39,49]	1,993	2.96	3.16	3.16	2.36	4.01	3.17	3.16	2.91	3.45
Provence-Alpes-Côte d'Azur	HPV16/18			(39,49]	7,655	4.22	4.08	4.08	3.59	4.56	4.08	4.07	3.85	4.31
Île-de-France	HPV16/18			(39,49]	50,328	3.32	3.56	3.56	3.35	3.77	3.56	3.55	3.44	3.68
Auvergne-Rhône-Alpes	HPV16/18			(49,59]	1,035	3.09	3.16	3.19	2.13	4.35	3.19	3.19	2.91	3.47
Bourgogne-Franche-Comté	HPV16/18			(49,59]	826	2.42	2.95	2.91	1.82	4.24	2.94	2.93	2.67	3.25
Bretagne	HPV16/18			(49,59]	2,517	2.86	3.04	3.02	2.34	3.77	3.04	3.04	2.78	3.31
Centre-Val de Loire	HPV16/18			(49,59]	8,682	2.59	2.92	2.91	2.56	3.32	2.92	2.92	2.77	3.09
Corse	HPV16/18			(49,59]	123	2.44	2.83	2.44	0.00	6.50	2.79	2.77	2.18	3.53

Table S8 Observed HR HPV cervical infection prevalence, posterior predictive HR HPV cervical infection prevalence, and posterior expected HR HPV cervical infection prevalence, stratified by various dimensions. ***(continued)***

							Posterior distribution							
							Predictive prevalence				Expected prevalence			
									ETI95%				ETI95%	
Region	Genotypes	Screening pathway	Year	Age	Number of tests	Observed	Average	Median	LB	UB	Average	Median	LB	UB
Grand Est	HPV16/18			(49,59]	3,050	2.82	2.94	2.92	2.33	3.57	2.93	2.93	2.70	3.16
Hauts-de-France	HPV16/18			(49,59]	6,400	3.23	2.91	2.91	2.48	3.36	2.91	2.91	2.75	3.08
Normandie	HPV16/18			(49,59]	4,427	3.50	3.27	3.25	2.71	3.86	3.27	3.26	3.06	3.48
Nouvelle-Aquitaine	HPV16/18			(49,59]	7,434	3.12	3.05	3.04	2.65	3.47	3.05	3.05	2.89	3.24
Occitanie	HPV16/18			(49,59]	3,221	3.51	3.35	3.35	2.73	4.04	3.36	3.36	3.13	3.60
Pays de la Loire	HPV16/18			(49,59]	1,688	2.31	2.58	2.55	1.84	3.44	2.58	2.58	2.36	2.82
Provence-Alpes-Côte d'Azur	HPV16/18			(49,59]	6,437	3.08	3.24	3.23	2.77	3.76	3.25	3.24	3.05	3.45
Île-de-France	HPV16/18			(49,59]	37,984	2.79	2.84	2.83	2.64	3.05	2.84	2.84	2.72	2.95
Auvergne-Rhône-Alpes	HPV16/18			(59,66]	524	1.34	2.96	2.86	1.53	4.58	2.96	2.95	2.68	3.25
Bourgogne-Franche-Comté	HPV16/18			(59,66]	546	3.85	2.67	2.56	1.47	4.03	2.69	2.68	2.41	2.98
Bretagne	HPV16/18			(59,66]	1,451	2.83	2.72	2.69	1.93	3.59	2.72	2.72	2.46	3.01
Centre-Val de Loire	HPV16/18			(59,66]	4,471	2.75	2.68	2.68	2.17	3.20	2.68	2.68	2.51	2.88
Corse	HPV16/18			(59,66]	68	1.47	2.72	2.94	0.00	7.35	2.72	2.69	2.20	3.37
Grand Est	HPV16/18			(59,66]	1,602	2.31	2.71	2.68	1.87	3.56	2.71	2.71	2.49	2.95
Hauts-de-France	HPV16/18			(59,66]	3,095	2.42	2.71	2.71	2.13	3.33	2.72	2.72	2.54	2.91
Normandie	HPV16/18			(59,66]	2,387	3.02	2.97	2.97	2.30	3.69	2.96	2.96	2.74	3.20
Nouvelle-Aquitaine	HPV16/18			(59,66]	4,321	3.03	2.83	2.82	2.31	3.40	2.83	2.83	2.64	3.04
Occitanie	HPV16/18			(59,66]	1,714	3.33	3.11	3.09	2.28	4.03	3.11	3.11	2.88	3.36
Pays de la Loire	HPV16/18			(59,66]	888	2.48	2.52	2.48	1.58	3.60	2.51	2.50	2.27	2.77
Provence-Alpes-Côte d'Azur	HPV16/18			(59,66]	3,201	2.94	3.02	3.00	2.41	3.66	3.02	3.01	2.80	3.24
Île-de-France	HPV16/18			(59,66]	18,088	2.71	2.65	2.65	2.39	2.92	2.64	2.64	2.50	2.80
Auvergne-Rhône-Alpes	Other genotypes			[30,39]			12.73	12.73	11.11	14.46	12.73	12.72	11.88	13.60
Bourgogne-Franche-Comté	Other genotypes			[30,39]			11.66	11.61	9.83	13.53	11.67	11.67	10.82	12.65
Bretagne	Other genotypes			[30,39]			11.65	11.64	10.50	12.88	11.63	11.63	10.99	12.31
Centre-Val de Loire	Other genotypes			[30,39]			11.26	11.26	10.54	11.95	11.26	11.26	10.88	11.64
Corse	Other genotypes			[30,39]			13.54	13.64	7.27	20.00	13.59	13.53	11.37	16.20
Grand Est	Other genotypes			[30,39]			11.38	11.38	10.22	12.52	11.38	11.37	10.80	12.01
Hauts-de-France	Other genotypes			[30,39]			11.20	11.20	10.56	11.84	11.20	11.20	10.84	11.58
Normandie	Other genotypes			[30,39]			12.09	12.08	11.23	13.00	12.08	12.08	11.59	12.58
Nouvelle-Aquitaine	Other genotypes			[30,39]			12.23	12.22	11.47	12.99	12.23	12.22	11.83	12.64
Occitanie	Other genotypes			[30,39]			13.04	13.03	11.84	14.23	13.04	13.03	12.44	13.68
Pays de la Loire	Other genotypes			[30,39]			9.59	9.60	8.19	11.05	9.61	9.60	8.98	10.26
Provence-Alpes-Côte d'Azur	Other genotypes			[30,39]			12.87	12.86	12.11	13.66	12.87	12.88	12.44	13.31
Île-de-France	Other genotypes			[30,39]			12.50	12.50	12.16	12.85	12.50	12.50	12.29	12.73
Auvergne-Rhône-Alpes	Other genotypes			(39,49]	1,258	8.43	8.89	8.90	7.23	10.65	8.89	8.88	8.28	9.56
Bourgogne-Franche-Comté	Other genotypes			(39,49]	1,096	8.67	8.14	8.12	6.39	9.95	8.12	8.11	7.49	8.79
Bretagne	Other genotypes			(39,49]	2,970	8.96	8.05	8.01	7.00	9.19	8.05	8.05	7.60	8.53
Centre-Val de Loire	Other genotypes			(39,49]	9,293	7.95	7.85	7.86	7.23	8.47	7.85	7.85	7.57	8.14
Corse	Other genotypes			(39,49]	129	14.73	10.15	10.08	5.43	16.28	10.10	10.05	8.19	12.40

Table S8 Observed HR HPV cervical infection prevalence, posterior predictive HR HPV cervical infection prevalence, and posterior expected HR HPV cervical infection prevalence, stratified by various dimensions. ***(continued)***

							Posterior distribution							
							Predictive prevalence				Expected prevalence			
									ETI95%				ETI95%	
Region	Genotypes	Screening pathway	Year	Age	Number of tests	Observed	Average	Median	LB	UB	Average	Median	LB	UB
Grand Est	Other genotypes			(39,49]	3,360	8.96	8.10	8.10	7.14	9.17	8.11	8.11	7.69	8.60
Hauts-de-France	Other genotypes			(39,49]	8,705	8.65	7.92	7.93	7.27	8.57	7.92	7.92	7.63	8.21
Normandie	Other genotypes			(39,49]	5,956	8.28	8.52	8.51	7.72	9.35	8.52	8.52	8.15	8.90
Nouvelle-Aquitaine	Other genotypes			(39,49]	8,585	8.69	8.60	8.60	7.97	9.27	8.61	8.60	8.31	8.92
Occitanie	Other genotypes			(39,49]	3,661	9.01	9.35	9.34	8.36	10.41	9.35	9.35	8.90	9.84
Pays de la Loire	Other genotypes			(39,49]	1,993	6.67	6.92	6.92	5.72	8.13	6.91	6.91	6.45	7.41
Provence-Alpes-Côte d'Azur	Other genotypes			(39,49]	7,655	8.83	8.96	8.95	8.27	9.68	8.96	8.96	8.62	9.31
Île-de-France	Other genotypes			(39,49]	50,328	8.31	8.65	8.65	8.35	8.96	8.65	8.65	8.47	8.84
Auvergne-Rhône-Alpes	Other genotypes			(49,59]	1,035	7.63	7.62	7.63	5.89	9.37	7.63	7.61	7.09	8.20
Bourgogne-Franche-Comté	Other genotypes			(49,59]	826	7.38	6.96	6.90	5.21	8.72	6.96	6.96	6.43	7.52
Bretagne	Other genotypes			(49,59]	2,517	6.87	6.79	6.79	5.72	7.87	6.78	6.78	6.39	7.19
Centre-Val de Loire	Other genotypes			(49,59]	8,682	6.15	6.66	6.66	6.07	7.24	6.66	6.66	6.40	6.92
Corse	Other genotypes			(49,59]	123	5.69	8.46	8.13	3.25	13.82	8.40	8.35	6.81	10.29
Grand Est	Other genotypes			(49,59]	3,050	6.00	6.90	6.89	5.93	7.87	6.90	6.90	6.53	7.32
Hauts-de-France	Other genotypes			(49,59]	6,400	7.58	6.71	6.70	6.05	7.39	6.72	6.72	6.47	6.99
Normandie	Other genotypes			(49,59]	4,427	6.44	7.11	7.12	6.28	7.95	7.11	7.11	6.80	7.44
Nouvelle-Aquitaine	Other genotypes			(49,59]	7,434	7.37	7.24	7.24	6.63	7.90	7.24	7.24	6.97	7.52
Occitanie	Other genotypes			(49,59]	3,221	8.20	7.99	7.98	7.02	9.03	7.99	7.99	7.60	8.41
Pays de la Loire	Other genotypes			(49,59]	1,688	6.10	5.97	5.98	4.80	7.23	5.98	5.97	5.58	6.43
Provence-Alpes-Côte d'Azur	Other genotypes			(49,59]	6,437	8.16	7.57	7.57	6.87	8.28	7.58	7.58	7.27	7.89
Île-de-France	Other genotypes			(49,59]	37,984	7.22	7.32	7.32	7.01	7.65	7.32	7.32	7.14	7.52
Auvergne-Rhône-Alpes	Other genotypes			(59,66]	524	6.11	6.77	6.68	4.58	8.97	6.80	6.79	6.29	7.34
Bourgogne-Franche-Comté	Other genotypes			(59,66]	546	8.06	6.19	6.23	4.21	8.42	6.21	6.20	5.71	6.76
Bretagne	Other genotypes			(59,66]	1,451	5.86	5.93	5.93	4.69	7.31	5.94	5.94	5.53	6.36
Centre-Val de Loire	Other genotypes			(59,66]	4,471	6.08	5.90	5.90	5.14	6.64	5.90	5.90	5.61	6.17
Corse	Other genotypes			(59,66]	68	2.94	7.56	7.35	1.47	14.71	7.59	7.55	6.32	9.10
Grand Est	Other genotypes			(59,66]	1,602	4.62	6.21	6.18	4.99	7.55	6.19	6.18	5.81	6.57
Hauts-de-France	Other genotypes			(59,66]	3,095	6.20	6.07	6.04	5.23	6.98	6.07	6.07	5.78	6.36
Normandie	Other genotypes			(59,66]	2,387	5.70	6.24	6.24	5.24	7.25	6.25	6.24	5.91	6.58
Nouvelle-Aquitaine	Other genotypes			(59,66]	4,321	6.23	6.51	6.50	5.72	7.31	6.51	6.51	6.21	6.84
Occitanie	Other genotypes			(59,66]	1,714	7.29	7.16	7.18	5.89	8.40	7.18	7.17	6.78	7.60
Pays de la Loire	Other genotypes			(59,66]	888	4.73	5.58	5.52	4.05	7.21	5.60	5.59	5.17	6.09
Provence-Alpes-Côte d'Azur	Other genotypes			(59,66]	3,201	7.19	6.78	6.78	5.87	7.75	6.78	6.78	6.44	7.12
Île-de-France	Other genotypes			(59,66]	18,088	6.58	6.47	6.47	6.04	6.91	6.47	6.47	6.23	6.71
	HPV16/18	Opportunistic		[30,39]			4.58	4.58	4.42	4.74	4.58	4.58	4.47	4.69
	HPV16/18	Organised		[30,39]			3.65	3.66	3.02	4.33	3.66	3.65	3.22	4.12
	HPV16/18	Opportunistic		(39,49]	100,422	3.71	3.74	3.74	3.58	3.90	3.74	3.74	3.63	3.85
	HPV16/18	Organised		(39,49]	4,567	2.93	3.01	3.00	2.45	3.59	3.01	3.00	2.71	3.31
	HPV16/18	Opportunistic		(49,59]	78,627	2.95	2.98	2.98	2.82	3.16	2.98	2.98	2.88	3.09

Table S8 **Observed HR HPV cervical infection prevalence, posterior predictive HR HPV cervical infection prevalence, and posterior expected HR HPV cervical infection prevalence, stratified by various dimensions.** ***(continued)***

Region	Genotypes	Screening pathway	Year	Age	Number of tests	Observed	Posterior distribution							
							Predictive prevalence				Expected prevalence			
									ETI95%				ETI95%	
							Average	Median	LB	UB	Average	Median	LB	UB
	HPV16/18	Organised		(49,59]	5,197	2.42	2.49	2.48	2.02	3.00	2.50	2.50	2.25	2.77
	HPV16/18	Opportunistic		(59,66]	37,369	2.80	2.78	2.78	2.57	3.02	2.78	2.78	2.63	2.94
	HPV16/18	Organised		(59,66]	4,987	2.53	2.46	2.47	1.93	3.03	2.47	2.46	2.14	2.84
	Other genotypes	Opportunistic		[30,39]			12.28	12.28	12.03	12.54	12.28	12.29	12.10	12.46
	Other genotypes	Organised		[30,39]			9.02	8.99	7.99	10.14	9.04	9.03	8.35	9.76
	Other genotypes	Opportunistic		(39,49]	100,422	8.49	8.57	8.57	8.34	8.81	8.57	8.57	8.40	8.73
	Other genotypes	Organised		(39,49]	4,567	6.74	6.66	6.66	5.82	7.55	6.68	6.68	6.21	7.17
	Other genotypes	Opportunistic		(49,59]	78,627	7.22	7.25	7.24	7.01	7.50	7.25	7.25	7.08	7.42
	Other genotypes	Organised		(49,59]	5,197	6.02	6.13	6.12	5.39	6.89	6.13	6.13	5.71	6.58
	Other genotypes	Opportunistic		(59,66]	37,369	6.43	6.45	6.45	6.10	6.80	6.45	6.45	6.22	6.70
	Other genotypes	Organised		(59,66]	4,987	5.82	5.83	5.82	5.01	6.68	5.83	5.82	5.30	6.40
Auvergne-Rhône-Alpes	HPV16/18	Opportunistic			4,422	3.78	4.03	4.03	3.37	4.75	4.04	4.04	3.71	4.38
Auvergne-Rhône-Alpes	HPV16/18	Organised			249	4.42	3.60	3.61	1.61	6.02	3.61	3.59	3.10	4.19
Bourgogne-Franche-Comté	HPV16/18	Opportunistic			3,773	3.74	3.73	3.74	3.05	4.45	3.73	3.72	3.38	4.11
Bourgogne-Franche-Comté	HPV16/18	Organised			262	2.29	2.77	2.67	1.15	4.96	2.77	2.76	2.30	3.30
Bretagne	HPV16/18	Opportunistic			8,765	4.26	4.22	4.21	3.70	4.76	4.22	4.21	3.89	4.57
Bretagne	HPV16/18	Organised			2,297	2.44	2.55	2.53	1.83	3.40	2.56	2.55	2.16	3.04
Centre-Val de Loire	HPV16/18	Opportunistic			30,545	3.73	3.65	3.65	3.39	3.92	3.65	3.64	3.49	3.83
Centre-Val de Loire	HPV16/18	Organised			2,204	3.27	2.79	2.77	2.09	3.58	2.80	2.80	2.53	3.11
Corse	HPV16/18	Opportunistic			427	3.51	3.43	3.28	1.64	5.39	3.40	3.38	2.70	4.26
Corse	HPV16/18	Organised			3	0.00	2.90	0.00	0.00	33.33	3.04	3.01	2.38	3.84
Grand Est	HPV16/18	Opportunistic			10,708	3.70	3.70	3.69	3.26	4.15	3.69	3.68	3.44	3.96
Grand Est	HPV16/18	Organised			1,178	2.55	2.71	2.72	1.70	3.74	2.71	2.70	2.34	3.11
Hauts-de-France	HPV16/18	Opportunistic			29,988	3.80	3.80	3.79	3.52	4.09	3.80	3.80	3.62	3.98
Hauts-de-France	HPV16/18	Organised			1,950	2.46	2.82	2.82	2.05	3.64	2.83	2.82	2.51	3.17
Normandie	HPV16/18	Opportunistic			19,589	4.36	4.18	4.18	3.81	4.55	4.18	4.17	3.94	4.41
Normandie	HPV16/18	Organised			374	2.14	2.83	2.67	1.34	4.81	2.82	2.82	2.46	3.20
Nouvelle-Aquitaine	HPV16/18	Opportunistic			26,985	3.90	3.94	3.94	3.64	4.25	3.94	3.94	3.75	4.13
Nouvelle-Aquitaine	HPV16/18	Organised			3,736	2.76	2.88	2.86	2.27	3.51	2.89	2.88	2.59	3.22
Occitanie	HPV16/18	Opportunistic			11,511	4.20	4.15	4.14	3.71	4.60	4.15	4.15	3.90	4.41
Occitanie	HPV16/18	Organised			1,013	2.57	3.38	3.36	2.27	4.64	3.39	3.38	3.00	3.82
Pays de la Loire	HPV16/18	Opportunistic			5,691	3.09	3.25	3.25	2.72	3.80	3.24	3.24	2.98	3.52
Pays de la Loire	HPV16/18	Organised			1,014	2.37	2.55	2.56	1.58	3.65	2.55	2.54	2.22	2.89
Provence-Alpes-Côte d'Azur	HPV16/18	Opportunistic			26,765	4.09	4.14	4.14	3.82	4.46	4.14	4.13	3.93	4.36
Provence-Alpes-Côte d'Azur	HPV16/18	Organised			1,124	4.63	3.76	3.74	2.58	5.07	3.75	3.74	3.22	4.37
Île-de-France	HPV16/18	Opportunistic			163,873	3.56	3.62	3.62	3.50	3.75	3.62	3.62	3.53	3.71
Île-de-France	HPV16/18	Organised			4,517	3.08	2.98	2.99	2.44	3.59	2.98	2.97	2.68	3.32
Auvergne-Rhône-Alpes	Other genotypes	Opportunistic			4,422	9.93	10.00	10.00	8.89	11.13	10.00	9.98	9.33	10.71
Auvergne-Rhône-Alpes	Other genotypes	Organised			249	10.84	8.07	8.03	4.82	11.65	8.14	8.13	7.13	9.15

Table S8 Observed HR HPV cervical infection prevalence, posterior predictive HR HPV cervical infection prevalence, and posterior expected HR HPV cervical infection prevalence, stratified by various dimensions. ***(continued)***

							Posterior distribution							
							Predictive prevalence				Expected prevalence			
									ETI95%				ETI95%	
Region	Genotypes	Screening pathway	Year	Age	Number of tests	Observed	Average	Median	LB	UB	Average	Median	LB	UB
Bourgogne-Franche-Comté	Other genotypes	Opportunistic			3,773	9.78	9.16	9.14	8.03	10.36	9.16	9.15	8.48	9.90
Bourgogne-Franche-Comté	Other genotypes	Organised			262	6.87	6.76	6.87	3.82	9.92	6.81	6.79	5.84	7.92
Bretagne	Other genotypes	Opportunistic			8,765	9.82	9.57	9.57	8.73	10.41	9.57	9.57	9.00	10.16
Bretagne	Other genotypes	Organised			2,297	5.01	5.98	5.96	4.75	7.27	5.98	5.96	5.21	6.86
Centre-Val de Loire	Other genotypes	Opportunistic			30,545	8.30	8.48	8.47	8.08	8.89	8.48	8.48	8.20	8.76
Centre-Val de Loire	Other genotypes	Organised			2,204	6.44	6.39	6.35	5.31	7.53	6.39	6.38	5.90	6.92
Corse	Other genotypes	Opportunistic			427	11.01	10.15	10.07	7.03	13.82	10.13	10.09	8.37	12.27
Corse	Other genotypes	Organised			3	0.00	6.46	0.00	0.00	33.33	6.67	6.63	5.35	8.25
Grand Est	Other genotypes	Opportunistic			10,708	8.78	8.84	8.84	8.13	9.55	8.84	8.84	8.39	9.34
Grand Est	Other genotypes	Organised			1,178	6.11	6.47	6.45	5.01	8.06	6.48	6.47	5.76	7.24
Hauts-de-France	Other genotypes	Opportunistic			29,988	8.97	9.06	9.06	8.62	9.50	9.06	9.07	8.77	9.36
Hauts-de-France	Other genotypes	Organised			1,950	6.26	6.61	6.56	5.38	7.90	6.61	6.60	6.03	7.23
Normandie	Other genotypes	Opportunistic			19,589	9.03	9.27	9.27	8.71	9.83	9.27	9.27	8.91	9.64
Normandie	Other genotypes	Organised			374	5.88	6.51	6.42	4.01	9.09	6.49	6.49	5.83	7.20
Nouvelle-Aquitaine	Other genotypes	Opportunistic			26,985	9.48	9.49	9.49	9.02	9.98	9.49	9.49	9.20	9.82
Nouvelle-Aquitaine	Other genotypes	Organised			3,736	7.39	7.10	7.09	6.18	8.11	7.11	7.11	6.60	7.69
Occitanie	Other genotypes	Opportunistic			11,511	9.99	10.03	10.03	9.32	10.74	10.03	10.03	9.58	10.52
Occitanie	Other genotypes	Organised			1,013	8.29	7.88	7.90	6.12	9.77	7.90	7.88	7.16	8.70
Pays de la Loire	Other genotypes	Opportunistic			5,691	7.43	7.63	7.63	6.78	8.52	7.63	7.62	7.14	8.18
Pays de la Loire	Other genotypes	Organised			1,014	4.93	5.84	5.82	4.34	7.50	5.84	5.83	5.24	6.49
Provence-Alpes-Côte d'Azur	Other genotypes	Opportunistic			26,765	9.84	9.90	9.90	9.42	10.40	9.90	9.91	9.57	10.24
Provence-Alpes-Côte d'Azur	Other genotypes	Organised			1,124	9.34	9.19	9.16	7.21	11.30	9.23	9.20	8.18	10.50
Île-de-France	Other genotypes	Opportunistic			163,873	9.58	9.60	9.60	9.40	9.80	9.60	9.60	9.46	9.75
Île-de-France	Other genotypes	Organised			4,517	7.50	7.23	7.22	6.31	8.19	7.24	7.23	6.72	7.82
Auvergne-Rhône-Alpes	HPV16/18		2020	[30,39]			4.14	4.10	2.41	6.27	4.14	4.13	3.73	4.58
Bourgogne-Franche-Comté	HPV16/18		2020	[30,39]			3.69	3.86	1.45	6.76	3.66	3.66	3.26	4.11
Bretagne	HPV16/18		2020	[30,39]			3.89	3.93	1.75	6.55	3.88	3.87	3.46	4.33
Centre-Val de Loire	HPV16/18		2020	[30,39]			3.79	3.77	2.68	4.94	3.79	3.79	3.55	4.05
Corse	HPV16/18		2020	[30,39]			4.22	4.35	1.09	8.70	4.22	4.20	3.24	5.42
Grand Est	HPV16/18		2020	[30,39]			3.62	3.40	1.28	6.38	3.58	3.57	3.21	3.96
Hauts-de-France	HPV16/18		2020	[30,39]			3.58	3.56	2.54	4.75	3.59	3.58	3.34	3.83
Normandie	HPV16/18		2020	[30,39]			4.05	4.09	2.81	5.50	4.06	4.06	3.75	4.37
Nouvelle-Aquitaine	HPV16/18		2020	[30,39]			3.82	3.80	2.38	5.39	3.84	3.84	3.57	4.10
Occitanie	HPV16/18		2020	[30,39]			4.76	4.68	3.16	6.46	4.77	4.76	4.31	5.26
Pays de la Loire	HPV16/18		2020	[30,39]			3.19	3.24	1.62	5.14	3.23	3.22	2.89	3.61
Provence-Alpes-Côte d'Azur	HPV16/18		2020	[30,39]			3.96	3.91	2.65	5.42	3.96	3.96	3.67	4.28
Île-de-France	HPV16/18		2020	[30,39]			3.84	3.84	3.55	4.17	3.85	3.85	3.68	4.02
Auvergne-Rhône-Alpes	HPV16/18		2020	(39,49]	262	0.76	3.37	3.44	1.52	5.73	3.38	3.37	3.04	3.74
Bourgogne-Franche-Comté	HPV16/18		2020	(39,49]	144	2.08	3.02	2.78	0.69	6.25	2.99	2.99	2.64	3.36

Table S8 Observed HR HPV cervical infection prevalence, posterior predictive HR HPV cervical infection prevalence, and posterior expected HR HPV cervical infection prevalence, stratified by various dimensions. ***(continued)***

							Posterior distribution							
							Predictive prevalence				Expected prevalence			
									ETI95%				ETI95%	
Region	Genotypes	Screening pathway	Year	Age	Number of tests	Observed	Average	Median	LB	UB	Average	Median	LB	UB
Bretagne	HPV16/18		2020	(39,49]	158	5.06	3.20	3.16	0.63	6.33	3.20	3.19	2.82	3.62
Centre-Val de Loire	HPV16/18		2020	(39,49]	1,001	2.50	3.12	3.10	2.10	4.30	3.13	3.14	2.92	3.36
Corse	HPV16/18		2020	(39,49]	118	3.39	3.56	3.39	0.85	7.63	3.50	3.47	2.66	4.54
Grand Est	HPV16/18		2020	(39,49]	241	2.90	2.90	2.90	0.83	5.39	2.89	2.88	2.57	3.23
Hauts-de-France	HPV16/18		2020	(39,49]	759	3.82	2.97	2.90	1.84	4.22	2.99	2.99	2.78	3.19
Normandie	HPV16/18		2020	(39,49]	675	3.56	3.26	3.26	1.93	4.74	3.25	3.24	2.99	3.50
Nouvelle-Aquitaine	HPV16/18		2020	(39,49]	424	2.83	3.20	3.07	1.65	4.95	3.20	3.20	2.96	3.43
Occitanie	HPV16/18		2020	(39,49]	628	3.66	4.05	3.98	2.55	5.73	4.05	4.05	3.64	4.49
Pays de la Loire	HPV16/18		2020	(39,49]	370	1.89	2.67	2.70	1.08	4.59	2.66	2.66	2.38	2.98
Provence-Alpes-Côte d'Azur	HPV16/18		2020	(39,49]	620	3.71	3.19	3.23	1.94	4.68	3.18	3.18	2.92	3.45
Île-de-France	HPV16/18		2020	(39,49]	16,220	2.78	3.15	3.15	2.85	3.48	3.15	3.16	3.01	3.30
Auvergne-Rhône-Alpes	HPV16/18		2020	(49,59]	215	4.65	2.68	2.79	0.93	5.12	2.74	2.73	2.45	3.04
Bourgogne-Franche-Comté	HPV16/18		2020	(49,59]	98	1.02	2.55	2.04	0.00	6.12	2.48	2.48	2.22	2.78
Bretagne	HPV16/18		2020	(49,59]	107	1.87	2.51	2.80	0.00	5.61	2.51	2.50	2.20	2.86
Centre-Val de Loire	HPV16/18		2020	(49,59]	973	1.64	2.52	2.47	1.64	3.60	2.52	2.52	2.35	2.70
Corse	HPV16/18		2020	(49,59]	111	2.70	2.81	2.70	0.00	6.31	2.76	2.74	2.12	3.56
Grand Est	HPV16/18		2020	(49,59]	279	3.23	2.25	2.15	0.72	4.30	2.29	2.29	2.02	2.58
Hauts-de-France	HPV16/18		2020	(49,59]	626	2.72	2.34	2.24	1.28	3.67	2.35	2.35	2.18	2.52
Normandie	HPV16/18		2020	(49,59]	521	3.07	2.58	2.50	1.34	4.03	2.58	2.58	2.37	2.80
Nouvelle-Aquitaine	HPV16/18		2020	(49,59]	367	3.00	2.52	2.45	1.09	4.09	2.53	2.53	2.34	2.73
Occitanie	HPV16/18		2020	(49,59]	536	2.99	3.11	2.99	1.68	4.66	3.13	3.12	2.83	3.44
Pays de la Loire	HPV16/18		2020	(49,59]	279	0.72	2.17	2.15	0.72	3.94	2.19	2.19	1.96	2.45
Provence-Alpes-Côte d'Azur	HPV16/18		2020	(49,59]	509	1.96	2.51	2.55	1.18	3.93	2.52	2.52	2.32	2.74
Île-de-France	HPV16/18		2020	(49,59]	11,877	2.32	2.52	2.52	2.21	2.84	2.52	2.51	2.40	2.64
Auvergne-Rhône-Alpes	HPV16/18		2020	(59,66]	96	3.12	2.46	2.08	0.00	6.25	2.52	2.52	2.24	2.84
Bourgogne-Franche-Comté	HPV16/18		2020	(59,66]	88	4.55	2.34	2.27	0.00	5.68	2.31	2.30	2.04	2.60
Bretagne	HPV16/18		2020	(59,66]	65	1.54	2.24	1.54	0.00	6.15	2.24	2.23	1.92	2.59
Centre-Val de Loire	HPV16/18		2020	(59,66]	501	1.60	2.34	2.20	1.20	3.79	2.33	2.33	2.15	2.53
Corse	HPV16/18		2020	(59,66]	56	1.79	2.63	1.79	0.00	7.14	2.60	2.57	2.03	3.31
Grand Est	HPV16/18		2020	(59,66]	127	3.15	2.11	1.97	0.00	4.72	2.10	2.10	1.84	2.40
Hauts-de-France	HPV16/18		2020	(59,66]	276	2.54	2.15	2.17	0.72	3.99	2.17	2.17	1.99	2.36
Normandie	HPV16/18		2020	(59,66]	262	3.82	2.34	2.29	0.76	4.20	2.35	2.34	2.13	2.58
Nouvelle-Aquitaine	HPV16/18		2020	(59,66]	267	2.25	2.41	2.25	0.75	4.49	2.40	2.39	2.18	2.62
Occitanie	HPV16/18		2020	(59,66]	288	4.51	2.92	2.78	1.04	5.21	2.93	2.92	2.63	3.25
Pays de la Loire	HPV16/18		2020	(59,66]	142	0.70	2.15	2.11	0.00	4.93	2.15	2.15	1.91	2.43
Provence-Alpes-Côte d'Azur	HPV16/18		2020	(59,66]	209	1.44	2.39	2.39	0.48	4.78	2.40	2.40	2.18	2.62
Île-de-France	HPV16/18		2020	(59,66]	4,709	2.12	2.33	2.34	1.89	2.80	2.33	2.33	2.18	2.48
Auvergne-Rhône-Alpes	HPV16/18		2021	[30,39]			4.89	4.88	3.66	6.18	4.87	4.87	4.46	5.31
Bourgogne-Franche-Comté	HPV16/18		2021	[30,39]			4.31	4.27	2.73	5.97	4.32	4.31	3.89	4.79

Table S8 Observed HR HPV cervical infection prevalence, posterior predictive HR HPV cervical infection prevalence, and posterior expected HR HPV cervical infection prevalence, stratified by various dimensions. ***(continued)***

							Posterior distribution							
							Predictive prevalence				Expected prevalence			
									ETI95%				ETI95%	
Region	Genotypes	Screening pathway	Year	Age	Number of tests	Observed	Average	Median	LB	UB	Average	Median	LB	UB
Bretagne	HPV16/18		2021	[30,39]			4.43	4.36	3.05	5.88	4.41	4.40	4.00	4.85
Centre-Val de Loire	HPV16/18		2021	[30,39]			4.35	4.35	3.72	4.98	4.36	4.36	4.15	4.60
Corse	HPV16/18		2021	[30,39]			4.70	0.00	0.00	15.38	4.73	4.70	3.85	5.80
Grand Est	HPV16/18		2021	[30,39]			4.20	4.23	3.00	5.38	4.18	4.18	3.87	4.51
Hauts-de-France	HPV16/18		2021	[30,39]			4.17	4.17	3.56	4.82	4.17	4.17	3.95	4.39
Normandie	HPV16/18		2021	[30,39]			4.82	4.79	3.98	5.67	4.81	4.80	4.50	5.11
Nouvelle-Aquitaine	HPV16/18		2021	[30,39]			4.41	4.43	3.65	5.20	4.40	4.40	4.16	4.66
Occitanie	HPV16/18		2021	[30,39]			5.01	5.01	3.99	6.09	5.01	5.01	4.66	5.36
Pays de la Loire	HPV16/18		2021	[30,39]			3.72	3.69	2.49	4.99	3.70	3.69	3.35	4.06
Provence-Alpes-Côte d'Azur	HPV16/18		2021	[30,39]			4.74	4.74	4.08	5.48	4.74	4.74	4.48	5.02
Île-de-France	HPV16/18		2021	[30,39]			4.42	4.41	4.14	4.70	4.42	4.42	4.27	4.57
Auvergne-Rhône-Alpes	HPV16/18		2021	(39,49]	852	3.76	3.98	3.99	2.70	5.40	3.98	3.98	3.64	4.35
Bourgogne-Franche-Comté	HPV16/18		2021	(39,49]	392	3.06	3.58	3.57	1.79	5.61	3.58	3.57	3.21	3.96
Bretagne	HPV16/18		2021	(39,49]	604	3.31	3.58	3.48	2.15	5.14	3.59	3.58	3.26	3.98
Centre-Val de Loire	HPV16/18		2021	(39,49]	4,339	3.13	3.58	3.57	3.02	4.17	3.57	3.57	3.38	3.78
Corse	HPV16/18		2021	(39,49]	5	20.00	3.87	0.00	0.00	20.00	3.96	3.95	3.14	4.93
Grand Est	HPV16/18		2021	(39,49]	1,119	3.31	3.44	3.40	2.32	4.56	3.44	3.44	3.17	3.72
Hauts-de-France	HPV16/18		2021	(39,49]	3,026	3.80	3.47	3.47	2.84	4.13	3.46	3.46	3.27	3.65
Normandie	HPV16/18		2021	(39,49]	2,315	4.92	3.92	3.89	3.11	4.84	3.92	3.92	3.66	4.18
Nouvelle-Aquitaine	HPV16/18		2021	(39,49]	2,362	3.64	3.59	3.60	2.88	4.36	3.60	3.60	3.38	3.82
Occitanie	HPV16/18		2021	(39,49]	1,748	4.12	4.09	4.06	3.15	5.09	4.10	4.11	3.82	4.39
Pays de la Loire	HPV16/18		2021	(39,49]	919	2.61	3.08	3.05	1.96	4.35	3.09	3.09	2.81	3.39
Provence-Alpes-Côte d'Azur	HPV16/18		2021	(39,49]	3,177	3.71	3.86	3.84	3.12	4.56	3.85	3.85	3.62	4.10
Île-de-France	HPV16/18		2021	(39,49]	25,423	3.43	3.62	3.62	3.36	3.87	3.62	3.62	3.48	3.75
Auvergne-Rhône-Alpes	HPV16/18		2021	(49,59]	704	2.13	3.17	3.12	1.99	4.55	3.19	3.19	2.91	3.50
Bourgogne-Franche-Comté	HPV16/18		2021	(49,59]	330	3.33	2.90	2.73	1.21	4.85	2.90	2.90	2.62	3.23
Bretagne	HPV16/18		2021	(49,59]	592	2.03	2.79	2.70	1.52	4.22	2.78	2.78	2.51	3.09
Centre-Val de Loire	HPV16/18		2021	(49,59]	4,068	2.21	2.84	2.83	2.31	3.39	2.84	2.84	2.68	3.01
Corse	HPV16/18		2021	(49,59]	10	0.00	2.90	0.00	0.00	20.00	2.95	2.94	2.54	3.41
Grand Est	HPV16/18		2021	(49,59]	1,062	2.64	2.79	2.82	1.79	3.86	2.77	2.76	2.55	3.00
Hauts-de-France	HPV16/18		2021	(49,59]	2,111	3.17	2.76	2.75	2.08	3.51	2.77	2.76	2.60	2.93
Normandie	HPV16/18		2021	(49,59]	1,834	2.89	3.09	3.05	2.29	3.98	3.09	3.09	2.87	3.31
Nouvelle-Aquitaine	HPV16/18		2021	(49,59]	2,165	2.86	2.86	2.86	2.17	3.60	2.86	2.86	2.69	3.03
Occitanie	HPV16/18		2021	(49,59]	1,467	3.89	3.34	3.34	2.45	4.36	3.35	3.35	3.11	3.60
Pays de la Loire	HPV16/18		2021	(49,59]	781	3.07	2.50	2.43	1.41	3.71	2.49	2.49	2.26	2.74
Provence-Alpes-Côte d'Azur	HPV16/18		2021	(49,59]	2,584	2.90	3.05	3.06	2.36	3.79	3.06	3.05	2.86	3.26
Île-de-France	HPV16/18		2021	(49,59]	19,159	2.84	2.88	2.89	2.62	3.16	2.88	2.89	2.75	3.01
Auvergne-Rhône-Alpes	HPV16/18		2021	(59,66]	361	0.83	2.95	2.77	1.39	4.99	2.94	2.93	2.66	3.24
Bourgogne-Franche-Comté	HPV16/18		2021	(59,66]	229	4.80	2.66	2.62	0.87	4.80	2.68	2.68	2.41	2.99

Table S8 **Observed HR HPV cervical infection prevalence, posterior predictive HR HPV cervical infection prevalence, and posterior expected HR HPV cervical infection prevalence, stratified by various dimensions.** ***(continued)***

							Posterior distribution							
							Predictive prevalence				Expected prevalence			
									ETI95%				ETI95%	
Region	Genotypes	Screening pathway	Year	Age	Number of tests	Observed	Average	Median	LB	UB	Average	Median	LB	UB
Bretagne	HPV16/18		2021	(59,66]	260	2.31	2.60	2.69	0.77	4.62	2.61	2.60	2.34	2.91
Centre-Val de Loire	HPV16/18		2021	(59,66]	2,161	2.59	2.63	2.64	1.94	3.33	2.64	2.64	2.46	2.84
Corse	HPV16/18		2021	(59,66]	7	0.00	3.00	0.00	0.00	14.29	3.03	3.01	2.44	3.74
Grand Est	HPV16/18		2021	(59,66]	608	2.14	2.57	2.47	1.32	3.95	2.57	2.57	2.35	2.80
Hauts-de-France	HPV16/18		2021	(59,66]	980	2.24	2.56	2.55	1.63	3.57	2.56	2.55	2.37	2.75
Normandie	HPV16/18		2021	(59,66]	968	2.79	2.86	2.79	1.86	4.03	2.85	2.85	2.62	3.10
Nouvelle-Aquitaine	HPV16/18		2021	(59,66]	1,407	2.91	2.69	2.70	1.85	3.62	2.69	2.69	2.50	2.89
Occitanie	HPV16/18		2021	(59,66]	768	2.86	3.10	3.12	1.95	4.30	3.11	3.10	2.86	3.37
Pays de la Loire	HPV16/18		2021	(59,66]	378	2.65	2.44	2.38	1.06	4.23	2.44	2.43	2.19	2.71
Provence-Alpes-Côte d'Azur	HPV16/18		2021	(59,66]	1,336	2.84	2.83	2.84	2.02	3.74	2.83	2.83	2.62	3.06
Île-de-France	HPV16/18		2021	(59,66]	10,265	2.75	2.68	2.67	2.33	3.05	2.68	2.67	2.52	2.84
Auvergne-Rhône-Alpes	HPV16/18		2022	[30,39]			5.44	5.04	1.68	10.08	5.48	5.47	4.93	6.09
Bourgogne-Franche-Comté	HPV16/18		2022	[30,39]			4.63	4.52	2.95	6.48	4.62	4.61	4.15	5.14
Bretagne	HPV16/18		2022	[30,39]			4.81	4.80	3.60	6.17	4.82	4.82	4.41	5.27
Centre-Val de Loire	HPV16/18		2022	[30,39]			4.54	4.53	3.72	5.42	4.54	4.54	4.29	4.82
Corse	HPV16/18		2022	[30,39]			5.11	0.00	0.00	25.00	5.19	5.15	4.27	6.30
Grand Est	HPV16/18		2022	[30,39]			4.36	4.32	3.22	5.56	4.36	4.35	4.01	4.72
Hauts-de-France	HPV16/18		2022	[30,39]			4.43	4.41	3.77	5.09	4.43	4.43	4.19	4.68
Normandie	HPV16/18		2022	[30,39]			5.36	5.33	4.40	6.39	5.35	5.35	5.02	5.72
Nouvelle-Aquitaine	HPV16/18		2022	[30,39]			4.72	4.72	4.00	5.47	4.72	4.72	4.44	4.98
Occitanie	HPV16/18		2022	[30,39]			5.08	5.10	3.64	6.56	5.07	5.07	4.69	5.46
Pays de la Loire	HPV16/18		2022	[30,39]			4.15	4.09	2.20	6.60	4.11	4.11	3.75	4.52
Provence-Alpes-Côte d'Azur	HPV16/18		2022	[30,39]			5.13	5.13	4.31	5.95	5.12	5.12	4.82	5.43
Île-de-France	HPV16/18		2022	[30,39]			4.88	4.89	4.31	5.47	4.88	4.88	4.65	5.11
Auvergne-Rhône-Alpes	HPV16/18		2022	(39,49]	79	11.39	4.54	3.80	1.27	10.13	4.55	4.54	3.97	5.21
Bourgogne-Franche-Comté	HPV16/18		2022	(39,49]	334	3.59	3.83	3.89	1.80	5.99	3.82	3.81	3.40	4.26
Bretagne	HPV16/18		2022	(39,49]	836	4.43	3.83	3.83	2.51	5.26	3.83	3.82	3.50	4.18
Centre-Val de Loire	HPV16/18		2022	(39,49]	2,327	4.86	3.70	3.70	2.92	4.51	3.71	3.71	3.50	3.94
Corse	HPV16/18		2022	(39,49]	5	0.00	4.42	0.00	0.00	20.00	4.12	4.08	3.27	5.10
Grand Est	HPV16/18		2022	(39,49]	1,056	4.36	3.64	3.60	2.56	4.83	3.63	3.63	3.33	3.95
Hauts-de-France	HPV16/18		2022	(39,49]	2,668	4.20	3.66	3.67	2.92	4.42	3.67	3.67	3.45	3.88
Normandie	HPV16/18		2022	(39,49]	1,836	3.81	4.39	4.36	3.43	5.39	4.38	4.38	4.08	4.67
Nouvelle-Aquitaine	HPV16/18		2022	(39,49]	3,149	4.16	3.87	3.87	3.18	4.57	3.86	3.86	3.63	4.10
Occitanie	HPV16/18		2022	(39,49]	881	3.41	4.15	4.09	2.84	5.56	4.16	4.16	3.84	4.49
Pays de la Loire	HPV16/18		2022	(39,49]	274	2.19	3.41	3.28	1.46	5.84	3.37	3.37	3.06	3.72
Provence-Alpes-Côte d'Azur	HPV16/18		2022	(39,49]	2,168	4.20	4.19	4.20	3.32	5.07	4.19	4.20	3.93	4.47
Île-de-France	HPV16/18		2022	(39,49]	4,741	3.67	3.91	3.92	3.35	4.51	3.91	3.90	3.71	4.11
Auvergne-Rhône-Alpes	HPV16/18		2022	(49,59]	62	6.45	3.89	3.23	0.00	9.68	3.78	3.77	3.27	4.37
Bourgogne-Franche-Comté	HPV16/18		2022	(49,59]	240	2.50	3.11	2.92	1.25	5.42	3.09	3.09	2.78	3.46

Table S8 **Observed HR HPV cervical infection prevalence, posterior predictive HR HPV cervical infection prevalence, and posterior expected HR HPV cervical infection prevalence, stratified by various dimensions.** ***(continued)***

							Posterior distribution							
							Predictive prevalence				Expected prevalence			
									ETI95%				ETI95%	
Region	Genotypes	Screening pathway	Year	Age	Number of tests	Observed	Average	Median	LB	UB	Average	Median	LB	UB
Bretagne	HPV16/18		2022	(49,59]	679	2.36	3.06	3.09	1.77	4.42	3.06	3.06	2.78	3.35
Centre-Val de Loire	HPV16/18		2022	(49,59]	2,116	3.54	2.96	2.93	2.22	3.73	2.97	2.97	2.79	3.17
Corse	HPV16/18		2022	(49,59]	2	0.00	3.60	0.00	0.00	50.00	3.54	3.49	2.51	4.83
Grand Est	HPV16/18		2022	(49,59]	887	2.25	3.02	3.04	1.92	4.17	3.02	3.01	2.75	3.28
Hauts-de-France	HPV16/18		2022	(49,59]	1,992	3.01	2.93	2.91	2.21	3.71	2.93	2.93	2.75	3.12
Normandie	HPV16/18		2022	(49,59]	1,212	4.21	3.41	3.38	2.39	4.54	3.42	3.42	3.19	3.67
Nouvelle-Aquitaine	HPV16/18		2022	(49,59]	2,734	2.89	3.02	3.00	2.38	3.69	3.02	3.02	2.84	3.21
Occitanie	HPV16/18		2022	(49,59]	851	2.94	3.37	3.41	2.23	4.70	3.37	3.37	3.11	3.63
Pays de la Loire	HPV16/18		2022	(49,59]	225	1.33	2.76	2.67	0.89	4.89	2.75	2.75	2.49	3.05
Provence-Alpes-Côte d'Azur	HPV16/18		2022	(49,59]	1,876	3.14	3.32	3.30	2.51	4.21	3.32	3.32	3.10	3.54
Île-de-France	HPV16/18		2022	(49,59]	3,802	2.66	3.07	3.05	2.52	3.63	3.06	3.06	2.89	3.23
Auvergne-Rhône-Alpes	HPV16/18		2022	(59,66]	35	2.86	3.44	2.86	0.00	11.43	3.36	3.35	2.93	3.86
Bourgogne-Franche-Comté	HPV16/18		2022	(59,66]	137	2.19	2.77	2.92	0.73	5.84	2.79	2.78	2.47	3.13
Bretagne	HPV16/18		2022	(59,66]	401	2.99	2.76	2.74	1.25	4.49	2.75	2.75	2.49	3.04
Centre-Val de Loire	HPV16/18		2022	(59,66]	1,060	3.21	2.71	2.74	1.70	3.77	2.71	2.71	2.51	2.93
Corse	HPV16/18		2022	(59,66]	2	0.00	3.98	0.00	0.00	50.00	3.96	3.89	2.74	5.63
Grand Est	HPV16/18		2022	(59,66]	394	2.54	2.77	2.79	1.27	4.57	2.76	2.75	2.51	3.02
Hauts-de-France	HPV16/18		2022	(59,66]	883	2.49	2.71	2.72	1.70	3.85	2.72	2.72	2.53	2.93
Normandie	HPV16/18		2022	(59,66]	705	2.84	3.09	3.12	1.84	4.40	3.07	3.07	2.83	3.33
Nouvelle-Aquitaine	HPV16/18		2022	(59,66]	1,480	2.91	2.79	2.77	1.96	3.72	2.80	2.79	2.58	3.03
Occitanie	HPV16/18		2022	(59,66]	438	3.42	3.11	2.97	1.60	4.79	3.11	3.11	2.84	3.39
Pays de la Loire	HPV16/18		2022	(59,66]	145	2.07	2.74	2.76	0.69	5.52	2.68	2.68	2.39	3.01
Provence-Alpes-Côte d'Azur	HPV16/18		2022	(59,66]	902	3.10	3.06	2.99	2.00	4.21	3.06	3.06	2.82	3.31
Île-de-France	HPV16/18		2022	(59,66]	1,713	2.57	2.86	2.86	2.10	3.68	2.85	2.85	2.66	3.05
Auvergne-Rhône-Alpes	HPV16/18		2023	[30,39]			6.30	6.67	2.22	12.22	6.29	6.27	5.41	7.25
Bourgogne-Franche-Comté	HPV16/18		2023	[30,39]			4.84	4.91	2.64	7.55	4.83	4.82	4.25	5.46
Bretagne	HPV16/18		2023	[30,39]			5.09	5.08	4.03	6.24	5.09	5.09	4.67	5.54
Centre-Val de Loire	HPV16/18		2023	[30,39]			5.05	5.02	4.10	6.03	5.04	5.04	4.74	5.35
Corse	HPV16/18		2023	[30,39]			5.13	0.00	0.00	100.00	5.09	4.93	2.81	8.48
Grand Est	HPV16/18		2023	[30,39]			4.91	4.92	3.69	6.24	4.91	4.91	4.52	5.33
Hauts-de-France	HPV16/18		2023	[30,39]			4.95	4.93	4.20	5.70	4.95	4.94	4.66	5.24
Normandie	HPV16/18		2023	[30,39]			5.94	5.92	4.60	7.40	5.96	5.96	5.54	6.38
Nouvelle-Aquitaine	HPV16/18		2023	[30,39]			5.20	5.18	4.39	6.03	5.20	5.20	4.90	5.51
Occitanie	HPV16/18		2023	[30,39]			5.61	5.47	3.39	8.07	5.62	5.62	5.15	6.11
Pays de la Loire	HPV16/18		2023	[30,39]			4.34	4.27	2.47	6.29	4.34	4.33	3.92	4.80
Provence-Alpes-Côte d'Azur	HPV16/18		2023	[30,39]			5.74	5.73	4.75	6.80	5.75	5.75	5.41	6.12
Île-de-France	HPV16/18		2023	[30,39]			5.45	5.44	4.84	6.09	5.45	5.45	5.18	5.72
Auvergne-Rhône-Alpes	HPV16/18		2023	(39,49]	65	4.62	5.15	4.62	0.00	10.77	5.19	5.18	4.40	6.11
Bourgogne-Franche-Comté	HPV16/18		2023	(39,49]	226	3.54	3.88	3.98	1.33	6.64	3.82	3.81	3.36	4.36

Table S8 **Observed HR HPV cervical infection prevalence, posterior predictive HR HPV cervical infection prevalence, and posterior expected HR HPV cervical infection prevalence, stratified by various dimensions.** ***(continued)***

							Posterior distribution							
							Predictive prevalence				Expected prevalence			
									ETI95%				ETI95%	
Region	Genotypes	Screening pathway	Year	Age	Number of tests	Observed	Average	Median	LB	UB	Average	Median	LB	UB
Bretagne	HPV16/18		2023	(39,49]	1,372	4.30	4.07	4.08	3.06	5.25	4.06	4.05	3.72	4.44
Centre-Val de Loire	HPV16/18		2023	(39,49]	1,626	5.66	4.16	4.12	3.20	5.23	4.17	4.17	3.92	4.44
Corse	HPV16/18		2023	(39,49]	1	100.00	4.50	0.00	0.00	100.00	5.06	4.84	2.69	8.51
Grand Est	HPV16/18		2023	(39,49]	944	4.56	4.15	4.13	2.86	5.51	4.15	4.14	3.81	4.50
Hauts-de-France	HPV16/18		2023	(39,49]	2,252	5.02	4.17	4.17	3.33	5.02	4.16	4.16	3.91	4.42
Normandie	HPV16/18		2023	(39,49]	1,130	4.25	4.86	4.87	3.63	6.19	4.86	4.86	4.52	5.21
Nouvelle-Aquitaine	HPV16/18		2023	(39,49]	2,650	3.81	4.25	4.23	3.47	5.06	4.25	4.25	4.01	4.53
Occitanie	HPV16/18		2023	(39,49]	404	4.46	4.70	4.70	2.72	6.93	4.71	4.71	4.28	5.16
Pays de la Loire	HPV16/18		2023	(39,49]	430	5.12	3.62	3.49	2.09	5.58	3.62	3.62	3.27	4.01
Provence-Alpes-Côte d'Azur	HPV16/18		2023	(39,49]	1,690	5.38	4.68	4.67	3.73	5.80	4.67	4.66	4.37	4.99
Île-de-France	HPV16/18		2023	(39,49]	3,944	4.46	4.40	4.41	3.73	5.07	4.39	4.39	4.16	4.62
Auvergne-Rhône-Alpes	HPV16/18		2023	(49,59]	54	5.56	4.17	3.70	0.00	11.11	4.23	4.22	3.56	5.04
Bourgogne-Franche-Comté	HPV16/18		2023	(49,59]	158	1.27	3.07	3.16	0.63	5.70	3.06	3.05	2.69	3.47
Bretagne	HPV16/18		2023	(49,59]	1,139	3.69	3.21	3.16	2.19	4.30	3.21	3.20	2.92	3.52
Centre-Val de Loire	HPV16/18		2023	(49,59]	1,525	2.89	3.34	3.34	2.43	4.26	3.34	3.34	3.12	3.56
Grand Est	HPV16/18		2023	(49,59]	822	3.53	3.27	3.28	2.07	4.62	3.26	3.25	2.98	3.55
Hauts-de-France	HPV16/18		2023	(49,59]	1,671	3.77	3.28	3.23	2.45	4.19	3.29	3.28	3.09	3.51
Normandie	HPV16/18		2023	(49,59]	860	4.07	3.87	3.84	2.67	5.23	3.85	3.84	3.56	4.13
Nouvelle-Aquitaine	HPV16/18		2023	(49,59]	2,168	3.69	3.38	3.37	2.63	4.15	3.38	3.38	3.18	3.60
Occitanie	HPV16/18		2023	(49,59]	367	4.09	3.71	3.81	1.91	5.72	3.72	3.72	3.38	4.09
Pays de la Loire	HPV16/18		2023	(49,59]	403	2.48	2.91	2.73	1.49	4.71	2.93	2.93	2.64	3.26
Provence-Alpes-Côte d'Azur	HPV16/18		2023	(49,59]	1,468	3.68	3.74	3.75	2.79	4.77	3.73	3.73	3.48	3.99
Île-de-France	HPV16/18		2023	(49,59]	3,146	4.39	3.47	3.46	2.83	4.16	3.47	3.47	3.28	3.68
Auvergne-Rhône-Alpes	HPV16/18		2023	(59,66]	32	0.00	4.07	3.12	0.00	12.50	3.99	3.98	3.36	4.72
Bourgogne-Franche-Comté	HPV16/18		2023	(59,66]	92	3.26	2.85	2.17	0.00	6.52	2.89	2.89	2.51	3.32
Bretagne	HPV16/18		2023	(59,66]	725	3.03	2.79	2.76	1.66	4.14	2.79	2.78	2.48	3.12
Centre-Val de Loire	HPV16/18		2023	(59,66]	749	3.34	3.02	2.94	1.87	4.41	3.01	3.00	2.79	3.25
Corse	HPV16/18		2023	(59,66]	3	0.00	3.07	0.00	0.00	33.33	3.41	3.35	2.42	4.69
Grand Est	HPV16/18		2023	(59,66]	473	2.11	3.00	2.96	1.48	4.65	3.01	3.01	2.74	3.31
Hauts-de-France	HPV16/18		2023	(59,66]	956	2.51	3.04	3.03	1.99	4.18	3.04	3.03	2.81	3.28
Normandie	HPV16/18		2023	(59,66]	452	3.32	3.38	3.32	1.77	5.31	3.39	3.38	3.11	3.69
Nouvelle-Aquitaine	HPV16/18		2023	(59,66]	1,167	3.51	3.13	3.08	2.14	4.20	3.13	3.13	2.90	3.41
Occitanie	HPV16/18		2023	(59,66]	220	3.18	3.40	3.18	1.36	5.91	3.40	3.40	3.06	3.77
Pays de la Loire	HPV16/18		2023	(59,66]	223	3.59	2.74	2.69	0.90	4.93	2.74	2.73	2.43	3.08
Provence-Alpes-Côte d'Azur	HPV16/18		2023	(59,66]	754	3.32	3.47	3.45	2.25	4.78	3.47	3.47	3.20	3.76
Île-de-France	HPV16/18		2023	(59,66]	1,401	4.64	3.21	3.21	2.28	4.21	3.21	3.21	2.99	3.43
Auvergne-Rhône-Alpes	Other genotypes		2020	[30,39]			12.65	12.53	9.40	15.90	12.64	12.63	11.67	13.64
Bourgogne-Franche-Comté	Other genotypes		2020	[30,39]			11.21	11.11	6.76	15.94	11.23	11.22	10.27	12.29
Bretagne	Other genotypes		2020	[30,39]			10.92	10.92	6.99	14.86	10.91	10.91	10.02	11.85

Table S8 Observed HR HPV cervical infection prevalence, posterior predictive HR HPV cervical infection prevalence, and posterior expected HR HPV cervical infection prevalence, stratified by various dimensions. ***(continued)***

							Posterior distribution							
							Predictive prevalence				Expected prevalence			
									ETI95%				ETI95%	
Region	Genotypes	Screening pathway	Year	Age	Number of tests	Observed	Average	Median	LB	UB	Average	Median	LB	UB
Centre-Val de Loire	Other genotypes		2020	[30,39]			11.25	11.21	9.37	13.14	11.25	11.25	10.77	11.77
Corse	Other genotypes		2020	[30,39]			13.82	14.13	6.52	21.74	13.89	13.83	11.30	16.88
Grand Est	Other genotypes		2020	[30,39]			11.13	11.06	7.23	15.32	11.17	11.16	10.28	12.16
Hauts-de-France	Other genotypes		2020	[30,39]			10.94	10.94	8.99	12.81	10.91	10.91	10.43	11.42
Normandie	Other genotypes		2020	[30,39]			11.63	11.58	9.47	13.80	11.62	11.62	11.01	12.24
Nouvelle-Aquitaine	Other genotypes		2020	[30,39]			12.00	11.89	9.51	14.74	12.01	12.01	11.47	12.56
Occitanie	Other genotypes		2020	[30,39]			14.12	14.03	11.42	16.78	14.16	14.15	13.15	15.22
Pays de la Loire	Other genotypes		2020	[30,39]			9.43	9.46	6.49	12.43	9.46	9.44	8.62	10.35
Provence-Alpes-Côte d'Azur	Other genotypes		2020	[30,39]			12.42	12.36	10.21	14.88	12.43	12.44	11.78	13.08
Île-de-France	Other genotypes		2020	[30,39]			12.49	12.49	11.95	13.06	12.50	12.49	12.20	12.80
Auvergne-Rhône-Alpes	Other genotypes		2020	(39,49]	262	7.25	8.93	8.78	5.34	12.60	8.91	8.91	8.20	9.66
Bourgogne-Franche-Comté	Other genotypes		2020	(39,49]	144	6.94	7.95	7.64	3.47	12.50	7.93	7.92	7.25	8.68
Bretagne	Other genotypes		2020	(39,49]	158	10.76	7.80	7.59	3.80	12.03	7.86	7.86	7.10	8.71
Centre-Val de Loire	Other genotypes		2020	(39,49]	1,001	7.59	7.91	7.89	6.29	9.69	7.89	7.89	7.54	8.27
Corse	Other genotypes		2020	(39,49]	118	15.25	10.29	10.17	5.08	16.10	10.22	10.17	8.16	12.70
Grand Est	Other genotypes		2020	(39,49]	241	8.71	7.85	7.88	4.56	11.21	7.84	7.83	7.16	8.61
Hauts-de-France	Other genotypes		2020	(39,49]	759	7.25	7.66	7.64	5.67	9.62	7.66	7.65	7.29	8.05
Normandie	Other genotypes		2020	(39,49]	675	8.44	8.00	8.00	5.93	10.22	7.99	7.99	7.55	8.46
Nouvelle-Aquitaine	Other genotypes		2020	(39,49]	424	7.78	8.33	8.25	5.90	11.08	8.37	8.36	7.97	8.78
Occitanie	Other genotypes		2020	(39,49]	628	8.60	10.56	10.51	8.12	13.22	10.54	10.54	9.69	11.39
Pays de la Loire	Other genotypes		2020	(39,49]	370	7.03	6.86	6.76	4.32	9.46	6.84	6.84	6.22	7.50
Provence-Alpes-Côte d'Azur	Other genotypes		2020	(39,49]	620	9.52	8.43	8.39	6.29	10.81	8.44	8.43	7.94	8.96
Île-de-France	Other genotypes		2020	(39,49]	16,220	8.47	8.75	8.75	8.26	9.24	8.74	8.74	8.51	8.99
Auvergne-Rhône-Alpes	Other genotypes		2020	(49,59]	215	6.51	7.63	7.44	4.19	11.16	7.68	7.68	7.05	8.36
Bourgogne-Franche-Comté	Other genotypes		2020	(49,59]	98	6.12	6.87	7.14	2.04	12.24	6.92	6.90	6.33	7.57
Bretagne	Other genotypes		2020	(49,59]	107	6.54	6.61	6.54	2.80	12.15	6.58	6.56	5.91	7.32
Centre-Val de Loire	Other genotypes		2020	(49,59]	973	5.76	6.63	6.63	5.14	8.32	6.64	6.64	6.33	6.98
Corse	Other genotypes		2020	(49,59]	111	6.31	8.62	8.11	3.60	14.41	8.55	8.51	6.84	10.62
Grand Est	Other genotypes		2020	(49,59]	279	5.73	6.52	6.45	3.58	9.68	6.51	6.50	5.86	7.24
Hauts-de-France	Other genotypes		2020	(49,59]	626	7.03	6.44	6.39	4.63	8.47	6.45	6.45	6.11	6.80
Normandie	Other genotypes		2020	(49,59]	521	6.91	6.68	6.72	4.61	9.02	6.69	6.69	6.32	7.09
Nouvelle-Aquitaine	Other genotypes		2020	(49,59]	367	8.45	7.10	7.08	4.63	9.82	7.12	7.12	6.75	7.52
Occitanie	Other genotypes		2020	(49,59]	536	9.89	8.67	8.58	6.34	11.19	8.65	8.65	8.01	9.33
Pays de la Loire	Other genotypes		2020	(49,59]	279	5.73	5.80	5.73	3.23	8.96	5.82	5.81	5.33	6.37
Provence-Alpes-Côte d'Azur	Other genotypes		2020	(49,59]	509	8.64	7.13	7.07	5.11	9.43	7.12	7.12	6.69	7.55
Île-de-France	Other genotypes		2020	(49,59]	11,877	7.20	7.42	7.42	6.92	7.96	7.42	7.42	7.19	7.66
Auvergne-Rhône-Alpes	Other genotypes		2020	(59,66]	96	10.42	6.84	6.25	2.08	12.50	6.87	6.86	6.26	7.53
Bourgogne-Franche-Comté	Other genotypes		2020	(59,66]	88	7.95	6.23	5.68	2.27	11.36	6.23	6.22	5.65	6.84
Bretagne	Other genotypes		2020	(59,66]	65	4.62	5.57	6.15	0.00	12.31	5.67	5.64	5.01	6.42

Table S8 Observed HR HPV cervical infection prevalence, posterior predictive HR HPV cervical infection prevalence, and posterior expected HR HPV cervical infection prevalence, stratified by various dimensions. ***(continued)***

							Posterior distribution							
							Predictive prevalence				Expected prevalence			
									ETI95%				ETI95%	
Region	Genotypes	Screening pathway	Year	Age	Number of tests	Observed	Average	Median	LB	UB	Average	Median	LB	UB
Centre-Val de Loire	Other genotypes		2020	(59,66]	501	3.99	5.98	5.99	3.99	8.18	5.94	5.94	5.60	6.29
Corse	Other genotypes		2020	(59,66]	56	1.79	7.61	7.14	1.79	16.07	7.65	7.59	6.23	9.39
Grand Est	Other genotypes		2020	(59,66]	127	3.94	5.74	5.51	1.57	10.24	5.76	5.75	5.14	6.46
Hauts-de-France	Other genotypes		2020	(59,66]	276	6.16	5.72	5.80	3.26	8.70	5.69	5.69	5.34	6.04
Normandie	Other genotypes		2020	(59,66]	262	4.58	5.81	5.73	3.05	8.78	5.88	5.87	5.48	6.29
Nouvelle-Aquitaine	Other genotypes		2020	(59,66]	267	7.12	6.49	6.37	3.75	9.74	6.48	6.48	6.09	6.90
Occitanie	Other genotypes		2020	(59,66]	288	6.25	7.80	7.64	4.86	10.77	7.78	7.78	7.17	8.43
Pays de la Loire	Other genotypes		2020	(59,66]	142	3.52	5.43	5.63	2.11	9.86	5.44	5.43	4.93	6.03
Provence-Alpes-Côte d'Azur	Other genotypes		2020	(59,66]	209	5.26	6.39	6.22	3.35	9.58	6.38	6.38	5.98	6.79
Île-de-France	Other genotypes		2020	(59,66]	4,709	6.18	6.58	6.58	5.82	7.35	6.57	6.57	6.30	6.86
Auvergne-Rhône-Alpes	Other genotypes		2021	[30,39]			12.41	12.36	10.41	14.47	12.42	12.40	11.57	13.30
Bourgogne-Franche-Comté	Other genotypes		2021	[30,39]			11.13	11.09	8.53	13.99	11.14	11.13	10.28	12.07
Bretagne	Other genotypes		2021	[30,39]			10.45	10.46	8.28	12.64	10.42	10.41	9.72	11.15
Centre-Val de Loire	Other genotypes		2021	[30,39]			10.64	10.63	9.68	11.63	10.63	10.63	10.24	11.03
Corse	Other genotypes		2021	[30,39]			12.13	7.69	0.00	30.77	12.30	12.23	10.20	14.78
Grand Est	Other genotypes		2021	[30,39]			10.56	10.58	8.73	12.43	10.57	10.57	9.99	11.21
Hauts-de-France	Other genotypes		2021	[30,39]			10.38	10.39	9.48	11.36	10.39	10.39	10.02	10.79
Normandie	Other genotypes		2021	[30,39]			11.24	11.21	10.03	12.42	11.23	11.23	10.73	11.74
Nouvelle-Aquitaine	Other genotypes		2021	[30,39]			11.28	11.26	10.06	12.54	11.27	11.26	10.84	11.73
Occitanie	Other genotypes		2021	[30,39]			12.50	12.50	10.88	14.12	12.51	12.50	11.89	13.15
Pays de la Loire	Other genotypes		2021	[30,39]			8.94	8.97	7.08	10.87	8.97	8.96	8.30	9.70
Provence-Alpes-Côte d'Azur	Other genotypes		2021	[30,39]			11.96	11.95	10.91	13.05	11.95	11.96	11.51	12.43
Île-de-France	Other genotypes		2021	[30,39]			11.90	11.89	11.44	12.35	11.90	11.90	11.66	12.15
Auvergne-Rhône-Alpes	Other genotypes		2021	(39,49]	852	8.10	8.59	8.57	6.69	10.56	8.60	8.59	7.95	9.27
Bourgogne-Franche-Comté	Other genotypes		2021	(39,49]	392	9.44	7.81	7.91	5.10	10.71	7.78	7.78	7.14	8.46
Bretagne	Other genotypes		2021	(39,49]	604	8.61	7.26	7.28	5.13	9.44	7.24	7.24	6.70	7.79
Centre-Val de Loire	Other genotypes		2021	(39,49]	4,339	7.12	7.50	7.49	6.64	8.37	7.50	7.50	7.20	7.80
Corse	Other genotypes		2021	(39,49]	5	0.00	8.41	0.00	0.00	40.00	8.56	8.53	6.83	10.46
Grand Est	Other genotypes		2021	(39,49]	1,119	7.06	7.48	7.42	5.81	9.12	7.48	7.48	7.04	7.96
Hauts-de-France	Other genotypes		2021	(39,49]	3,026	7.60	7.34	7.34	6.38	8.36	7.34	7.34	7.05	7.63
Normandie	Other genotypes		2021	(39,49]	2,315	7.95	7.89	7.90	6.78	9.07	7.90	7.89	7.54	8.27
Nouvelle-Aquitaine	Other genotypes		2021	(39,49]	2,362	7.96	7.85	7.83	6.73	9.06	7.86	7.86	7.54	8.19
Occitanie	Other genotypes		2021	(39,49]	1,748	10.07	8.88	8.87	7.49	10.35	8.87	8.87	8.40	9.36
Pays de la Loire	Other genotypes		2021	(39,49]	919	6.42	6.45	6.42	4.79	8.05	6.43	6.43	5.96	6.95
Provence-Alpes-Côte d'Azur	Other genotypes		2021	(39,49]	3,177	8.31	8.34	8.34	7.40	9.38	8.33	8.33	7.97	8.71
Île-de-France	Other genotypes		2021	(39,49]	25,423	8.11	8.34	8.33	7.96	8.72	8.33	8.34	8.13	8.54
Auvergne-Rhône-Alpes	Other genotypes		2021	(49,59]	704	6.96	7.34	7.24	5.39	9.38	7.33	7.32	6.78	7.90
Bourgogne-Franche-Comté	Other genotypes		2021	(49,59]	330	6.67	6.69	6.67	3.94	9.39	6.68	6.67	6.13	7.25
Bretagne	Other genotypes		2021	(49,59]	592	3.89	5.96	5.91	4.05	8.11	5.94	5.94	5.50	6.40

Table S8 Observed HR HPV cervical infection prevalence, posterior predictive HR HPV cervical infection prevalence, and posterior expected HR HPV cervical infection prevalence, stratified by various dimensions. ***(continued)***

							Posterior distribution							
							Predictive prevalence				Expected prevalence			
									ETI95%				ETI95%	
Region	Genotypes	Screening pathway	Year	Age	Number of tests	Observed	Average	Median	LB	UB	Average	Median	LB	UB
Centre-Val de Loire	Other genotypes		2021	(49,59]	4,068	5.92	6.33	6.34	5.53	7.15	6.33	6.33	6.07	6.59
Corse	Other genotypes		2021	(49,59]	10	0.00	6.75	10.00	0.00	30.00	6.83	6.81	5.96	7.77
Grand Est	Other genotypes		2021	(49,59]	1,062	4.90	6.35	6.31	4.90	7.91	6.35	6.34	5.97	6.75
Hauts-de-France	Other genotypes		2021	(49,59]	2,111	6.44	6.22	6.21	5.21	7.30	6.23	6.23	5.98	6.51
Normandie	Other genotypes		2021	(49,59]	1,834	5.34	6.60	6.60	5.45	7.74	6.60	6.60	6.28	6.92
Nouvelle-Aquitaine	Other genotypes		2021	(49,59]	2,165	6.47	6.59	6.61	5.54	7.71	6.59	6.59	6.32	6.88
Occitanie	Other genotypes		2021	(49,59]	1,467	7.70	7.68	7.63	6.34	9.20	7.69	7.68	7.26	8.12
Pays de la Loire	Other genotypes		2021	(49,59]	781	5.51	5.52	5.51	3.97	7.30	5.52	5.51	5.12	5.98
Provence-Alpes-Côte d'Azur	Other genotypes		2021	(49,59]	2,584	7.39	7.00	7.00	6.00	8.09	7.01	7.00	6.70	7.34
Île-de-France	Other genotypes		2021	(49,59]	19,159	7.08	7.05	7.05	6.64	7.47	7.05	7.05	6.86	7.26
Auvergne-Rhône-Alpes	Other genotypes		2021	(59,66]	361	5.54	6.46	6.37	4.16	9.14	6.49	6.48	5.98	7.03
Bourgogne-Franche-Comté	Other genotypes		2021	(59,66]	229	7.86	5.95	5.68	3.06	9.17	5.97	5.97	5.46	6.51
Bretagne	Other genotypes		2021	(59,66]	260	4.23	5.37	5.38	2.69	8.46	5.37	5.36	4.92	5.82
Centre-Val de Loire	Other genotypes		2021	(59,66]	2,161	6.06	5.64	5.65	4.63	6.71	5.65	5.65	5.36	5.94
Corse	Other genotypes		2021	(59,66]	7	0.00	6.80	0.00	0.00	28.57	6.66	6.61	5.48	8.06
Grand Est	Other genotypes		2021	(59,66]	608	3.29	5.69	5.76	3.78	7.73	5.69	5.69	5.32	6.08
Hauts-de-France	Other genotypes		2021	(59,66]	980	4.18	5.56	5.51	4.18	7.04	5.55	5.55	5.27	5.84
Normandie	Other genotypes		2021	(59,66]	968	5.79	5.87	5.89	4.34	7.44	5.88	5.88	5.54	6.22
Nouvelle-Aquitaine	Other genotypes		2021	(59,66]	1,407	4.83	5.95	5.90	4.76	7.25	5.97	5.97	5.67	6.29
Occitanie	Other genotypes		2021	(59,66]	768	7.94	6.84	6.90	4.95	8.72	6.88	6.88	6.45	7.32
Pays de la Loire	Other genotypes		2021	(59,66]	378	3.17	5.19	5.03	2.91	7.67	5.19	5.18	4.77	5.67
Provence-Alpes-Côte d'Azur	Other genotypes		2021	(59,66]	1,336	7.11	6.26	6.29	4.94	7.56	6.24	6.24	5.91	6.58
Île-de-France	Other genotypes		2021	(59,66]	10,265	6.39	6.24	6.23	5.74	6.78	6.24	6.24	5.99	6.49
Auvergne-Rhône-Alpes	Other genotypes		2022	[30,39]			13.45	13.45	7.56	20.17	13.49	13.47	12.36	14.62
Bourgogne-Franche-Comté	Other genotypes		2022	[30,39]			11.72	11.79	8.84	14.73	11.74	11.73	10.82	12.77
Bretagne	Other genotypes		2022	[30,39]			11.32	11.32	9.43	13.29	11.29	11.29	10.60	11.99
Centre-Val de Loire	Other genotypes		2022	[30,39]			11.00	10.99	9.74	12.29	10.97	10.97	10.53	11.43
Corse	Other genotypes		2022	[30,39]			12.17	0.00	0.00	50.00	11.67	11.61	9.76	13.89
Grand Est	Other genotypes		2022	[30,39]			10.88	10.90	9.22	12.66	10.85	10.85	10.26	11.50
Hauts-de-France	Other genotypes		2022	[30,39]			10.90	10.91	9.91	11.86	10.89	10.89	10.48	11.30
Normandie	Other genotypes		2022	[30,39]			12.29	12.27	10.84	13.80	12.28	12.27	11.72	12.83
Nouvelle-Aquitaine	Other genotypes		2022	[30,39]			11.80	11.80	10.72	12.97	11.81	11.80	11.38	12.26
Occitanie	Other genotypes		2022	[30,39]			12.51	12.49	10.41	14.78	12.50	12.49	11.81	13.21
Pays de la Loire	Other genotypes		2022	[30,39]			9.92	9.75	6.60	13.21	9.91	9.90	9.16	10.72
Provence-Alpes-Côte d'Azur	Other genotypes		2022	[30,39]			12.70	12.73	11.42	13.88	12.71	12.70	12.21	13.22
Île-de-France	Other genotypes		2022	[30,39]			12.81	12.80	11.94	13.69	12.81	12.81	12.44	13.18
Auvergne-Rhône-Alpes	Other genotypes		2022	(39,49]	79	16.46	9.63	8.86	3.80	16.46	9.71	9.69	8.61	10.85
Bourgogne-Franche-Comté	Other genotypes		2022	(39,49]	334	8.68	8.17	8.08	5.09	11.38	8.17	8.16	7.48	8.90
Bretagne	Other genotypes		2022	(39,49]	836	7.30	7.73	7.66	5.86	9.69	7.73	7.73	7.23	8.22

Table S8 **Observed HR HPV cervical infection prevalence, posterior predictive HR HPV cervical infection prevalence, and posterior expected HR HPV cervical infection prevalence, stratified by various dimensions.** ***(continued)***

							Posterior distribution							
							Predictive prevalence				Expected prevalence			
									ETI95%				ETI95%	
Region	Genotypes	Screening pathway	Year	Age	Number of tests	Observed	Average	Median	LB	UB	Average	Median	LB	UB
Centre-Val de Loire	Other genotypes		2022	(39,49]	2,327	8.12	7.67	7.69	6.53	8.85	7.68	7.67	7.34	8.02
Corse	Other genotypes		2022	(39,49]	5	20.00	8.65	0.00	0.00	40.00	8.54	8.48	6.93	10.44
Grand Est	Other genotypes		2022	(39,49]	1,056	9.85	7.76	7.77	6.16	9.47	7.78	7.77	7.32	8.29
Hauts-de-France	Other genotypes		2022	(39,49]	2,668	8.43	7.67	7.68	6.60	8.73	7.66	7.66	7.35	7.99
Normandie	Other genotypes		2022	(39,49]	1,836	8.33	8.62	8.61	7.30	10.02	8.61	8.61	8.19	9.03
Nouvelle-Aquitaine	Other genotypes		2022	(39,49]	3,149	7.94	8.35	8.35	7.34	9.37	8.36	8.36	8.04	8.71
Occitanie	Other genotypes		2022	(39,49]	881	8.17	8.88	8.85	6.92	10.78	8.90	8.90	8.38	9.46
Pays de la Loire	Other genotypes		2022	(39,49]	274	6.20	7.01	6.93	4.01	10.22	7.04	7.03	6.47	7.65
Provence-Alpes-Côte d'Azur	Other genotypes		2022	(39,49]	2,168	8.30	8.91	8.90	7.70	10.15	8.92	8.92	8.55	9.30
Île-de-France	Other genotypes		2022	(39,49]	4,741	8.08	8.68	8.67	7.85	9.53	8.69	8.68	8.41	8.98
Auvergne-Rhône-Alpes	Other genotypes		2022	(49,59]	62	17.74	8.71	8.06	1.61	16.13	8.68	8.66	7.62	9.81
Bourgogne-Franche-Comté	Other genotypes		2022	(49,59]	240	7.92	6.98	7.08	3.75	10.42	6.99	6.98	6.40	7.62
Bretagne	Other genotypes		2022	(49,59]	679	6.77	6.61	6.63	4.71	8.69	6.63	6.62	6.18	7.06
Centre-Val de Loire	Other genotypes		2022	(49,59]	2,116	6.66	6.54	6.52	5.53	7.66	6.54	6.54	6.23	6.84
Corse	Other genotypes		2022	(49,59]	2	0.00	8.30	0.00	0.00	50.00	7.65	7.56	5.63	10.09
Grand Est	Other genotypes		2022	(49,59]	887	5.86	6.86	6.88	5.30	8.57	6.89	6.88	6.46	7.35
Hauts-de-France	Other genotypes		2022	(49,59]	1,992	8.08	6.53	6.53	5.47	7.68	6.54	6.54	6.26	6.85
Normandie	Other genotypes		2022	(49,59]	1,212	6.77	7.15	7.10	5.69	8.66	7.16	7.16	6.80	7.53
Nouvelle-Aquitaine	Other genotypes		2022	(49,59]	2,734	7.06	7.03	7.02	6.07	8.05	7.03	7.02	6.73	7.33
Occitanie	Other genotypes		2022	(49,59]	851	7.76	7.68	7.64	5.88	9.52	7.69	7.68	7.24	8.18
Pays de la Loire	Other genotypes		2022	(49,59]	225	6.67	6.19	6.22	3.11	9.33	6.16	6.16	5.66	6.73
Provence-Alpes-Côte d'Azur	Other genotypes		2022	(49,59]	1,876	7.36	7.49	7.52	6.34	8.69	7.51	7.51	7.16	7.85
Île-de-France	Other genotypes		2022	(49,59]	3,802	7.50	7.27	7.29	6.44	8.15	7.27	7.27	7.01	7.53
Auvergne-Rhône-Alpes	Other genotypes		2022	(59,66]	35	2.86	7.44	5.71	0.00	17.14	7.53	7.52	6.66	8.47
Bourgogne-Franche-Comté	Other genotypes		2022	(59,66]	137	5.84	6.09	5.84	2.19	10.22	6.14	6.14	5.59	6.74
Bretagne	Other genotypes		2022	(59,66]	401	6.23	5.76	5.74	3.49	8.23	5.78	5.78	5.34	6.23
Centre-Val de Loire	Other genotypes		2022	(59,66]	1,060	6.98	5.80	5.75	4.43	7.26	5.81	5.81	5.49	6.13
Corse	Other genotypes		2022	(59,66]	2	50.00	8.23	0.00	0.00	50.00	8.93	8.79	6.33	12.22
Grand Est	Other genotypes		2022	(59,66]	394	5.58	6.19	6.09	3.81	8.63	6.15	6.15	5.75	6.60
Hauts-de-France	Other genotypes		2022	(59,66]	883	6.00	5.91	5.89	4.42	7.59	5.91	5.91	5.60	6.23
Normandie	Other genotypes		2022	(59,66]	705	4.68	6.29	6.24	4.54	8.09	6.26	6.26	5.90	6.63
Nouvelle-Aquitaine	Other genotypes		2022	(59,66]	1,480	6.42	6.34	6.35	5.07	7.64	6.34	6.34	6.00	6.70
Occitanie	Other genotypes		2022	(59,66]	438	5.94	6.94	6.85	4.57	9.59	6.90	6.90	6.46	7.40
Pays de la Loire	Other genotypes		2022	(59,66]	145	5.52	5.79	5.52	2.07	9.67	5.80	5.79	5.25	6.41
Provence-Alpes-Côte d'Azur	Other genotypes		2022	(59,66]	902	6.21	6.70	6.65	4.99	8.43	6.69	6.69	6.34	7.06
Île-de-France	Other genotypes		2022	(59,66]	1,713	5.78	6.56	6.54	5.43	7.82	6.55	6.55	6.25	6.85
Auvergne-Rhône-Alpes	Other genotypes		2023	[30,39]			16.36	16.67	8.89	24.44	16.36	16.36	14.50	18.35
Bourgogne-Franche-Comté	Other genotypes		2023	[30,39]			13.04	12.83	8.68	17.74	13.08	13.07	11.89	14.33
Bretagne	Other genotypes		2023	[30,39]			12.56	12.53	10.88	14.30	12.56	12.55	11.85	13.34

Table S8 Observed HR HPV cervical infection prevalence, posterior predictive HR HPV cervical infection prevalence, and posterior expected HR HPV cervical infection prevalence, stratified by various dimensions. ***(continued)***

							Posterior distribution							
							Predictive prevalence				Expected prevalence			
									ETI95%				ETI95%	
Region	Genotypes	Screening pathway	Year	Age	Number of tests	Observed	Average	Median	LB	UB	Average	Median	LB	UB
Centre-Val de Loire	Other genotypes		2023	[30,39]			12.84	12.84	11.41	14.36	12.87	12.86	12.34	13.43
Corse	Other genotypes		2023	[30,39]			11.07	0.00	0.00	100.00	10.92	10.55	6.24	17.33
Grand Est	Other genotypes		2023	[30,39]			12.87	12.83	10.81	15.03	12.87	12.86	12.13	13.65
Hauts-de-France	Other genotypes		2023	[30,39]			12.75	12.73	11.59	13.97	12.76	12.76	12.27	13.25
Normandie	Other genotypes		2023	[30,39]			14.13	14.13	12.08	16.19	14.14	14.14	13.49	14.82
Nouvelle-Aquitaine	Other genotypes		2023	[30,39]			13.65	13.65	12.35	14.98	13.64	13.64	13.15	14.17
Occitanie	Other genotypes		2023	[30,39]			14.86	14.84	11.45	18.75	14.83	14.82	13.94	15.85
Pays de la Loire	Other genotypes		2023	[30,39]			10.94	11.01	8.09	14.16	10.97	10.95	10.10	11.89
Provence-Alpes-Côte d'Azur	Other genotypes		2023	[30,39]			14.95	14.95	13.42	16.51	14.95	14.94	14.35	15.56
Île-de-France	Other genotypes		2023	[30,39]			15.13	15.11	14.15	16.15	15.13	15.13	14.69	15.58
Auvergne-Rhône-Alpes	Other genotypes		2023	(39,49]	65	7.69	11.80	12.31	4.62	20.00	11.71	11.69	10.09	13.39
Bourgogne-Franche-Comté	Other genotypes		2023	(39,49]	226	8.41	8.79	8.85	5.31	12.83	8.74	8.74	7.86	9.71
Bretagne	Other genotypes		2023	(39,49]	1,372	9.91	8.61	8.60	7.07	10.20	8.63	8.63	8.12	9.18
Centre-Val de Loire	Other genotypes		2023	(39,49]	1,626	10.15	9.01	8.98	7.63	10.52	9.01	9.02	8.63	9.42
Corse	Other genotypes		2023	(39,49]	1	0.00	9.77	0.00	0.00	100.00	11.01	10.74	6.05	17.73
Grand Est	Other genotypes		2023	(39,49]	944	10.28	9.30	9.32	7.42	11.33	9.31	9.31	8.75	9.90
Hauts-de-France	Other genotypes		2023	(39,49]	2,252	10.79	9.10	9.10	7.86	10.30	9.10	9.10	8.72	9.49
Normandie	Other genotypes		2023	(39,49]	1,130	8.76	9.96	10.00	8.14	11.77	9.99	9.98	9.48	10.50
Nouvelle-Aquitaine	Other genotypes		2023	(39,49]	2,650	10.38	9.61	9.62	8.45	10.83	9.61	9.60	9.23	10.03
Occitanie	Other genotypes		2023	(39,49]	404	6.93	10.58	10.64	7.67	13.61	10.59	10.58	9.85	11.40
Pays de la Loire	Other genotypes		2023	(39,49]	430	7.21	7.93	7.91	5.35	10.70	7.91	7.90	7.27	8.62
Provence-Alpes-Côte d'Azur	Other genotypes		2023	(39,49]	1,690	10.24	10.37	10.36	8.87	11.89	10.37	10.37	9.91	10.84
Île-de-France	Other genotypes		2023	(39,49]	3,944	9.15	10.28	10.27	9.28	11.28	10.28	10.28	9.94	10.64
Auvergne-Rhône-Alpes	Other genotypes		2023	(49,59]	54	9.26	10.06	9.26	3.66	18.52	10.13	10.11	8.68	11.73
Bourgogne-Franche-Comté	Other genotypes		2023	(49,59]	158	8.86	7.56	7.59	3.80	12.03	7.53	7.52	6.78	8.36
Bretagne	Other genotypes		2023	(49,59]	1,139	8.52	7.34	7.33	5.79	8.96	7.33	7.33	6.88	7.83
Centre-Val de Loire	Other genotypes		2023	(49,59]	1,525	6.30	7.69	7.67	6.30	9.11	7.71	7.71	7.37	8.07
Grand Est	Other genotypes		2023	(49,59]	822	7.66	7.78	7.79	5.96	9.61	7.77	7.76	7.29	8.29
Hauts-de-France	Other genotypes		2023	(49,59]	1,671	8.62	7.66	7.66	6.34	9.04	7.66	7.66	7.33	8.01
Normandie	Other genotypes		2023	(49,59]	860	8.02	8.39	8.37	6.51	10.35	8.40	8.40	7.96	8.86
Nouvelle-Aquitaine	Other genotypes		2023	(49,59]	2,168	8.49	8.19	8.16	7.06	9.46	8.18	8.18	7.84	8.54
Occitanie	Other genotypes		2023	(49,59]	367	8.72	8.97	8.99	6.27	11.99	8.95	8.94	8.32	9.65
Pays de la Loire	Other genotypes		2023	(49,59]	403	7.20	6.84	6.70	4.47	9.68	6.87	6.86	6.29	7.51
Provence-Alpes-Côte d'Azur	Other genotypes		2023	(49,59]	1,468	10.35	8.84	8.86	7.43	10.35	8.86	8.85	8.44	9.29
Île-de-France	Other genotypes		2023	(49,59]	3,146	7.79	8.66	8.65	7.63	9.73	8.66	8.65	8.35	8.99
Auvergne-Rhône-Alpes	Other genotypes		2023	(59,66]	32	3.12	9.42	9.38	0.00	21.88	9.31	9.30	8.03	10.71
Bourgogne-Franche-Comté	Other genotypes		2023	(59,66]	92	11.96	6.91	6.52	2.17	11.96	6.90	6.89	6.16	7.71
Bretagne	Other genotypes		2023	(59,66]	725	6.34	6.26	6.21	4.41	8.14	6.26	6.26	5.76	6.79
Centre-Val de Loire	Other genotypes		2023	(59,66]	749	6.28	6.72	6.68	4.94	8.68	6.71	6.71	6.34	7.08

Table S8 **Observed HR HPV cervical infection prevalence, posterior predictive HR HPV cervical infection prevalence, and posterior expected HR HPV cervical infection prevalence, stratified by various dimensions.** ***(continued)***

							Posterior distribution							
							Predictive prevalence				Expected prevalence			
									ETI95%				ETI95%	
Region	Genotypes	Screening pathway	Year	Age	Number of tests	Observed	Average	Median	LB	UB	Average	Median	LB	UB
Corse	Other genotypes		2023	(59,66]	3	0.00	8.02	0.00	0.00	33.33	7.75	7.67	5.63	10.49
Grand Est	Other genotypes		2023	(59,66]	473	5.71	7.01	6.98	4.65	9.51	6.96	6.96	6.48	7.47
Hauts-de-France	Other genotypes		2023	(59,66]	956	8.47	6.84	6.80	5.23	8.58	6.86	6.85	6.49	7.21
Normandie	Other genotypes		2023	(59,66]	452	7.74	7.23	7.30	4.87	9.73	7.23	7.22	6.81	7.68
Nouvelle-Aquitaine	Other genotypes		2023	(59,66]	1,167	7.46	7.39	7.37	5.91	9.00	7.39	7.39	7.01	7.80
Occitanie	Other genotypes		2023	(59,66]	220	9.09	7.91	7.73	4.55	11.82	7.97	7.97	7.38	8.63
Pays de la Loire	Other genotypes		2023	(59,66]	223	7.62	6.21	6.28	3.14	9.42	6.25	6.24	5.67	6.86
Provence-Alpes-Côte d'Azur	Other genotypes		2023	(59,66]	754	9.02	7.90	7.82	5.97	9.95	7.93	7.93	7.51	8.36
Île-de-France	Other genotypes		2023	(59,66]	1,401	10.28	7.71	7.71	6.35	9.14	7.71	7.71	7.37	8.08
	HPV16/18	Opportunistic	2020	[30,39]			3.86	3.86	3.59	4.15	3.86	3.86	3.70	4.03
	HPV16/18	Organised	2020	[30,39]			3.49	0.00	0.00	16.67	3.73	3.69	2.76	4.92
	HPV16/18	Opportunistic	2020	(39,49]	21,616	2.86	3.17	3.17	2.91	3.46	3.17	3.17	3.03	3.32
	HPV16/18	Organised	2020	(39,49]	4	0.00	2.73	0.00	0.00	25.00	2.84	2.82	2.07	3.77
	HPV16/18	Opportunistic	2020	(49,59]	16,489	2.36	2.53	2.53	2.27	2.81	2.53	2.53	2.41	2.66
	HPV16/18	Organised	2020	(49,59]	9	0.00	3.21	0.00	0.00	22.22	3.07	3.03	2.32	4.00
	HPV16/18	Opportunistic	2020	(59,66]	7,073	2.23	2.35	2.35	1.98	2.74	2.35	2.35	2.20	2.50
	HPV16/18	Organised	2020	(59,66]	13	23.08	2.71	0.00	0.00	15.38	2.67	2.65	2.03	3.49
	HPV16/18	Opportunistic	2021	[30,39]			4.46	4.46	4.24	4.68	4.46	4.46	4.33	4.59
	HPV16/18	Organised	2021	[30,39]			3.80	3.81	2.47	5.25	3.78	3.77	3.15	4.52
	HPV16/18	Opportunistic	2021	(39,49]	45,649	3.56	3.65	3.65	3.44	3.86	3.65	3.65	3.53	3.78
	HPV16/18	Organised	2021	(39,49]	632	1.90	3.13	3.16	1.74	4.75	3.15	3.13	2.67	3.67
	HPV16/18	Opportunistic	2021	(49,59]	35,896	2.83	2.91	2.91	2.71	3.13	2.91	2.91	2.80	3.03
	HPV16/18	Organised	2021	(49,59]	971	2.47	2.55	2.57	1.54	3.60	2.57	2.56	2.21	2.97
	HPV16/18	Opportunistic	2021	(59,66]	17,707	2.71	2.71	2.71	2.43	3.00	2.71	2.71	2.56	2.87
	HPV16/18	Organised	2021	(59,66]	2,021	2.57	2.59	2.57	1.78	3.46	2.58	2.57	2.14	3.11
	HPV16/18	Opportunistic	2022	[30,39]			4.89	4.89	4.56	5.21	4.88	4.88	4.70	5.07
	HPV16/18	Organised	2022	[30,39]			3.62	3.60	2.74	4.61	3.63	3.62	3.18	4.14
	HPV16/18	Opportunistic	2022	(39,49]	18,412	4.16	4.01	4.01	3.67	4.34	4.01	4.01	3.84	4.18
	HPV16/18	Organised	2022	(39,49]	1,942	3.35	2.96	2.94	2.16	3.81	2.96	2.96	2.61	3.31
	HPV16/18	Opportunistic	2022	(49,59]	14,453	3.11	3.19	3.19	2.86	3.51	3.19	3.19	3.04	3.34
	HPV16/18	Organised	2022	(49,59]	2,225	2.25	2.49	2.47	1.80	3.19	2.49	2.48	2.20	2.79
	HPV16/18	Opportunistic	2022	(59,66]	6,798	2.96	2.96	2.96	2.53	3.41	2.96	2.96	2.77	3.15
	HPV16/18	Organised	2022	(59,66]	1,497	2.27	2.38	2.34	1.54	3.27	2.39	2.39	2.04	2.79
	HPV16/18	Opportunistic	2023	[30,39]			5.44	5.45	5.07	5.82	5.45	5.44	5.23	5.66
	HPV16/18	Organised	2023	[30,39]			3.62	3.61	2.71	4.56	3.62	3.62	3.12	4.15
	HPV16/18	Opportunistic	2023	(39,49]	14,745	4.87	4.50	4.50	4.12	4.89	4.49	4.49	4.30	4.69
	HPV16/18	Organised	2023	(39,49]	1,989	2.87	3.01	3.02	2.26	3.92	3.01	3.01	2.64	3.42
	HPV16/18	Opportunistic	2023	(49,59]	11,789	3.93	3.59	3.59	3.22	3.97	3.59	3.59	3.42	3.77
	HPV16/18	Organised	2023	(49,59]	1,992	2.61	2.47	2.46	1.71	3.26	2.48	2.48	2.15	2.86

Table S8 **Observed HR HPV cervical infection prevalence, posterior predictive HR HPV cervical infection prevalence, and posterior expected HR HPV cervical infection prevalence, stratified by various dimensions.** ***(continued)***

							Posterior distribution							
							Predictive prevalence				Expected prevalence			
									ETI95%				ETI95%	
Region	Genotypes	Screening pathway	Year	Age	Number of tests	Observed	Average	Median	LB	UB	Average	Median	LB	UB
	HPV16/18	Opportunistic	2023	(59,66]	5,791	3.59	3.32	3.32	2.81	3.85	3.32	3.32	3.11	3.54
	HPV16/18	Organised	2023	(59,66]	1,456	2.54	2.38	2.34	1.58	3.37	2.38	2.37	1.96	2.83
	Other genotypes	Opportunistic	2020	[30,39]			12.30	12.29	11.81	12.81	12.30	12.30	12.01	12.59
	Other genotypes	Organised	2020	[30,39]			8.88	8.33	0.00	25.00	9.08	9.05	7.36	11.12
	Other genotypes	Opportunistic	2020	(39,49]	21,616	8.42	8.64	8.64	8.19	9.07	8.64	8.64	8.41	8.87
	Other genotypes	Organised	2020	(39,49]	4	0.00	5.48	0.00	0.00	25.00	5.60	5.56	4.35	7.06
	Other genotypes	Opportunistic	2020	(49,59]	16,489	7.18	7.30	7.29	6.86	7.77	7.30	7.29	7.08	7.53
	Other genotypes	Organised	2020	(49,59]	9	11.11	6.39	0.00	0.00	22.22	6.52	6.48	5.25	8.00
	Other genotypes	Opportunistic	2020	(59,66]	7,073	5.91	6.48	6.48	5.85	7.11	6.47	6.47	6.21	6.74
	Other genotypes	Organised	2020	(59,66]	13	7.69	5.78	7.69	0.00	23.08	5.72	5.69	4.59	7.02
	Other genotypes	Opportunistic	2021	[30,39]			11.58	11.57	11.24	11.93	11.58	11.58	11.37	11.80
	Other genotypes	Organised	2021	[30,39]			8.77	8.74	6.79	10.91	8.79	8.76	7.80	9.90
	Other genotypes	Opportunistic	2021	(39,49]	45,649	8.07	8.12	8.12	7.83	8.43	8.12	8.12	7.94	8.30
	Other genotypes	Organised	2021	(39,49]	632	3.80	6.23	6.17	4.27	8.23	6.24	6.23	5.52	6.97
	Other genotypes	Opportunistic	2021	(49,59]	35,896	6.71	6.86	6.86	6.54	7.18	6.86	6.86	6.69	7.05
	Other genotypes	Organised	2021	(49,59]	971	5.77	5.70	5.66	4.22	7.31	5.71	5.71	5.13	6.31
	Other genotypes	Opportunistic	2021	(59,66]	17,707	6.05	6.12	6.12	5.72	6.55	6.13	6.13	5.89	6.37
	Other genotypes	Organised	2021	(59,66]	2,021	5.79	5.69	5.69	4.50	6.98	5.69	5.67	5.00	6.45
	Other genotypes	Opportunistic	2022	[30,39]			12.15	12.15	11.64	12.67	12.15	12.15	11.86	12.44
	Other genotypes	Organised	2022	[30,39]			9.07	9.08	7.64	10.57	9.07	9.06	8.30	9.91
	Other genotypes	Opportunistic	2022	(39,49]	18,412	8.37	8.47	8.47	8.01	8.94	8.48	8.48	8.25	8.71
	Other genotypes	Organised	2022	(39,49]	1,942	7.00	6.61	6.59	5.41	7.88	6.63	6.62	6.09	7.18
	Other genotypes	Opportunistic	2022	(49,59]	14,453	7.35	7.16	7.15	6.69	7.65	7.16	7.16	6.94	7.38
	Other genotypes	Organised	2022	(49,59]	2,225	6.56	6.17	6.20	5.08	7.33	6.17	6.17	5.66	6.70
	Other genotypes	Opportunistic	2022	(59,66]	6,798	6.18	6.38	6.37	5.77	7.05	6.38	6.38	6.11	6.65
	Other genotypes	Organised	2022	(59,66]	1,497	5.41	5.90	5.88	4.54	7.35	5.88	5.87	5.28	6.57
	Other genotypes	Opportunistic	2023	[30,39]			14.32	14.32	13.73	14.91	14.32	14.33	13.99	14.67
	Other genotypes	Organised	2023	[30,39]			9.08	9.08	7.65	10.65	9.12	9.11	8.29	9.99
	Other genotypes	Opportunistic	2023	(39,49]	14,745	10.06	9.99	9.99	9.45	10.55	9.99	9.99	9.70	10.29
	Other genotypes	Organised	2023	(39,49]	1,989	7.44	6.86	6.84	5.63	8.14	6.88	6.87	6.28	7.53
	Other genotypes	Opportunistic	2023	(49,59]	11,789	8.65	8.46	8.46	7.89	9.03	8.46	8.46	8.21	8.75
	Other genotypes	Organised	2023	(49,59]	1,992	5.52	6.29	6.28	5.12	7.53	6.28	6.28	5.72	6.87
	Other genotypes	Opportunistic	2023	(59,66]	5,791	8.51	7.51	7.51	6.77	8.24	7.51	7.51	7.20	7.83
	Other genotypes	Organised	2023	(59,66]	1,456	6.25	5.94	5.98	4.60	7.35	5.98	5.97	5.30	6.70
Auvergne-Rhône-Alpes	HPV16/18	Opportunistic	2020		988	3.44	3.46	3.44	2.33	4.76	3.48	3.47	3.14	3.83
Bourgogne-Franche-Comté	HPV16/18	Opportunistic	2020		537	2.98	3.08	2.98	1.68	4.66	3.05	3.04	2.73	3.39
Bretagne	HPV16/18	Opportunistic	2020		559	3.76	3.24	3.22	1.79	4.83	3.23	3.23	2.88	3.62
Centre-Val de Loire	HPV16/18	Opportunistic	2020		3,667	2.62	3.07	3.08	2.51	3.68	3.08	3.07	2.89	3.27
Centre-Val de Loire	HPV16/18	Organised	2020		3	33.33	3.13	0.00	0.00	33.33	2.98	2.93	2.15	4.04

Table S8 **Observed HR HPV cervical infection prevalence, posterior predictive HR HPV cervical infection prevalence, and posterior expected HR HPV cervical infection prevalence, stratified by various dimensions.** ***(continued)***

							Posterior distribution							
							Predictive prevalence				Expected prevalence			
									ETI95%				ETI95%	
Region	Genotypes	Screening pathway	Year	Age	Number of tests	Observed	Average	Median	LB	UB	Average	Median	LB	UB
Corse	HPV16/18	Opportunistic	2020		377	3.45	3.36	3.18	1.59	5.57	3.32	3.30	2.55	4.28
Grand Est	HPV16/18	Opportunistic	2020		882	3.17	2.77	2.72	1.70	3.97	2.77	2.77	2.48	3.08
Hauts-de-France	HPV16/18	Opportunistic	2020		2,833	3.46	3.01	3.00	2.36	3.71	3.02	3.01	2.83	3.22
Hauts-de-France	HPV16/18	Organised	2020		7	14.29	2.78	0.00	0.00	14.29	2.84	2.81	2.13	3.74
Normandie	HPV16/18	Opportunistic	2020		2,313	3.67	3.30	3.29	2.55	4.11	3.30	3.30	3.06	3.54
Nouvelle-Aquitaine	HPV16/18	Opportunistic	2020		1,688	3.38	3.16	3.14	2.31	4.09	3.16	3.16	2.95	3.38
Nouvelle-Aquitaine	HPV16/18	Organised	2020		1	0.00	2.33	0.00	0.00	0.00	2.52	2.43	1.46	4.06
Occitanie	HPV16/18	Opportunistic	2020		2,175	4.23	3.91	3.91	3.03	4.83	3.92	3.91	3.56	4.31
Occitanie	HPV16/18	Organised	2020		4	0.00	3.35	0.00	0.00	25.00	3.19	3.13	2.18	4.54
Pays de la Loire	HPV16/18	Opportunistic	2020		1,156	1.73	2.65	2.68	1.73	3.63	2.67	2.66	2.40	2.96
Pays de la Loire	HPV16/18	Organised	2020		5	0.00	2.57	0.00	0.00	20.00	2.80	2.75	2.00	3.77
Provence-Alpes-Côte d'Azur	HPV16/18	Opportunistic	2020		2,129	3.05	3.24	3.24	2.49	4.04	3.24	3.23	3.00	3.49
Provence-Alpes-Côte d'Azur	HPV16/18	Organised	2020		2	50.00	2.80	0.00	0.00	50.00	2.71	2.66	1.88	3.84
Île-de-France	HPV16/18	Opportunistic	2020		51,739	3.07	3.19	3.18	3.00	3.39	3.19	3.19	3.06	3.32
Île-de-France	HPV16/18	Organised	2020		16	0.00	3.37	0.00	0.00	12.50	3.44	3.41	2.64	4.44
Auvergne-Rhône-Alpes	HPV16/18	Opportunistic	2021		2,995	3.47	4.06	4.04	3.31	4.84	4.06	4.06	3.72	4.42
Auvergne-Rhône-Alpes	HPV16/18	Organised	2021		152	1.32	3.56	3.29	0.66	6.58	3.54	3.52	2.93	4.23
Bourgogne-Franche-Comté	HPV16/18	Opportunistic	2021		1,510	3.91	3.59	3.58	2.65	4.64	3.60	3.59	3.26	3.97
Bourgogne-Franche-Comté	HPV16/18	Organised	2021		27	7.41	2.58	3.70	0.00	11.11	2.64	2.63	2.15	3.22
Bretagne	HPV16/18	Opportunistic	2021		2,260	3.41	3.66	3.67	2.83	4.51	3.66	3.65	3.32	4.02
Bretagne	HPV16/18	Organised	2021		114	4.39	2.46	2.63	0.00	5.26	2.47	2.45	1.94	3.11
Centre-Val de Loire	HPV16/18	Opportunistic	2021		14,308	3.39	3.50	3.49	3.15	3.86	3.50	3.50	3.33	3.68
Centre-Val de Loire	HPV16/18	Organised	2021		721	2.64	2.91	2.91	1.66	4.30	2.92	2.91	2.51	3.39
Corse	HPV16/18	Opportunistic	2021		34	2.94	3.77	2.94	0.00	11.76	3.80	3.79	3.29	4.39
Corse	HPV16/18	Organised	2021		1	0.00	2.37	0.00	0.00	0.00	2.84	2.77	1.84	4.21
Grand Est	HPV16/18	Opportunistic	2021		3,787	3.22	3.38	3.38	2.77	4.01	3.37	3.37	3.13	3.62
Grand Est	HPV16/18	Organised	2021		136	0.74	2.41	2.21	0.00	5.15	2.43	2.43	2.01	2.92
Hauts-de-France	HPV16/18	Opportunistic	2021		10,358	3.72	3.57	3.57	3.18	3.97	3.57	3.57	3.39	3.75
Hauts-de-France	HPV16/18	Organised	2021		390	2.31	2.98	2.82	1.28	4.87	3.01	3.00	2.56	3.51
Normandie	HPV16/18	Opportunistic	2021		8,045	4.29	3.94	3.93	3.46	4.44	3.93	3.93	3.69	4.17
Normandie	HPV16/18	Organised	2021		34	5.88	3.03	2.94	0.00	8.82	2.90	2.89	2.40	3.48
Nouvelle-Aquitaine	HPV16/18	Opportunistic	2021		8,526	3.48	3.58	3.58	3.16	4.02	3.58	3.59	3.39	3.78
Nouvelle-Aquitaine	HPV16/18	Organised	2021		391	4.09	2.66	2.56	1.28	4.35	2.65	2.64	2.27	3.09
Occitanie	HPV16/18	Opportunistic	2021		5,534	4.03	4.09	4.08	3.51	4.70	4.09	4.09	3.83	4.37
Occitanie	HPV16/18	Organised	2021		305	3.61	3.67	3.61	1.64	6.23	3.66	3.66	3.06	4.36
Pays de la Loire	HPV16/18	Opportunistic	2021		2,811	3.34	3.11	3.09	2.45	3.84	3.10	3.10	2.82	3.39
Pays de la Loire	HPV16/18	Organised	2021		270	1.85	2.57	2.59	0.74	4.81	2.58	2.56	2.12	3.12
Provence-Alpes-Côte d'Azur	HPV16/18	Opportunistic	2021		11,257	3.82	3.89	3.88	3.46	4.32	3.88	3.88	3.67	4.11
Provence-Alpes-Côte d'Azur	HPV16/18	Organised	2021		56	1.79	3.49	3.57	0.00	8.93	3.50	3.49	2.91	4.23

Table S8 Observed HR HPV cervical infection prevalence, posterior predictive HR HPV cervical infection prevalence, and posterior expected HR HPV cervical infection prevalence, stratified by various dimensions. ***(continued)***

							Posterior distribution							
							Predictive prevalence				Expected prevalence			
									ETI95%				ETI95%	
Region	Genotypes	Screening pathway	Year	Age	Number of tests	Observed	Average	Median	LB	UB	Average	Median	LB	UB
Île-de-France	HPV16/18	Opportunistic	2021		83,161	3.67	3.64	3.64	3.48	3.80	3.64	3.64	3.53	3.75
Île-de-France	HPV16/18	Organised	2021		1,999	2.95	2.87	2.85	2.05	3.80	2.87	2.85	2.41	3.38
Auvergne-Rhône-Alpes	HPV16/18	Opportunistic	2022		247	7.29	4.85	4.86	2.43	7.69	4.83	4.82	4.33	5.42
Auvergne-Rhône-Alpes	HPV16/18	Organised	2022		48	14.58	3.51	4.17	0.00	8.33	3.54	3.52	2.92	4.27
Bourgogne-Franche-Comté	HPV16/18	Opportunistic	2022		1,175	3.40	3.94	3.91	2.81	5.19	3.93	3.92	3.53	4.37
Bourgogne-Franche-Comté	HPV16/18	Organised	2022		45	2.22	2.94	2.22	0.00	8.89	3.03	3.01	2.50	3.65
Bretagne	HPV16/18	Opportunistic	2022		2,496	3.85	4.22	4.21	3.41	5.09	4.22	4.21	3.87	4.61
Bretagne	HPV16/18	Organised	2022		586	2.05	2.49	2.39	1.19	3.92	2.50	2.49	2.06	3.00
Centre-Val de Loire	HPV16/18	Opportunistic	2022		7,059	4.32	3.75	3.75	3.27	4.25	3.76	3.76	3.55	3.98
Centre-Val de Loire	HPV16/18	Organised	2022		918	3.70	2.70	2.72	1.63	3.92	2.72	2.71	2.41	3.04
Corse	HPV16/18	Opportunistic	2022		11	0.00	4.67	0.00	0.00	18.18	4.55	4.52	3.86	5.40
Corse	HPV16/18	Organised	2022		2	0.00	3.17	0.00	0.00	50.00	3.15	3.11	2.28	4.23
Grand Est	HPV16/18	Opportunistic	2022		3,082	3.89	3.83	3.83	3.11	4.61	3.83	3.83	3.52	4.15
Grand Est	HPV16/18	Organised	2022		622	3.05	2.82	2.73	1.61	4.34	2.81	2.81	2.40	3.26
Hauts-de-France	HPV16/18	Opportunistic	2022		8,863	3.69	3.89	3.88	3.43	4.34	3.89	3.89	3.68	4.10
Hauts-de-France	HPV16/18	Organised	2022		1,080	2.22	2.80	2.78	1.85	3.89	2.81	2.81	2.47	3.18
Normandie	HPV16/18	Opportunistic	2022		5,759	4.43	4.43	4.43	3.85	5.05	4.43	4.43	4.15	4.71
Normandie	HPV16/18	Organised	2022		153	1.31	2.67	2.61	0.65	5.23	2.69	2.68	2.30	3.10
Nouvelle-Aquitaine	HPV16/18	Opportunistic	2022		9,232	4.09	3.98	3.98	3.54	4.46	3.98	3.98	3.75	4.20
Nouvelle-Aquitaine	HPV16/18	Organised	2022		1,733	2.25	2.79	2.77	1.96	3.64	2.79	2.79	2.47	3.14
Occitanie	HPV16/18	Opportunistic	2022		2,624	4.27	4.23	4.23	3.43	5.11	4.23	4.23	3.92	4.54
Occitanie	HPV16/18	Organised	2022		507	2.17	3.28	3.16	1.78	4.93	3.30	3.29	2.87	3.80
Pays de la Loire	HPV16/18	Opportunistic	2022		721	2.91	3.70	3.61	2.36	5.27	3.66	3.66	3.33	4.03
Pays de la Loire	HPV16/18	Organised	2022		241	1.24	2.51	2.49	0.83	4.56	2.49	2.49	2.13	2.88
Provence-Alpes-Côte d'Azur	HPV16/18	Opportunistic	2022		7,734	4.38	4.27	4.27	3.75	4.80	4.27	4.26	4.02	4.52
Provence-Alpes-Côte d'Azur	HPV16/18	Organised	2022		504	5.36	3.85	3.77	2.18	5.75	3.86	3.84	3.27	4.55
Île-de-France	HPV16/18	Opportunistic	2022		15,516	3.74	4.08	4.08	3.71	4.43	4.07	4.07	3.89	4.26
Île-de-France	HPV16/18	Organised	2022		1,307	2.91	2.98	2.98	1.99	3.98	2.98	2.97	2.63	3.38
Auvergne-Rhône-Alpes	HPV16/18	Opportunistic	2023		192	5.73	5.57	5.21	2.60	9.38	5.56	5.54	4.78	6.47
Auvergne-Rhône-Alpes	HPV16/18	Organised	2023		49	4.08	3.81	4.08	0.00	10.20	3.90	3.87	3.11	4.82
Bourgogne-Franche-Comté	HPV16/18	Opportunistic	2023		551	4.72	4.32	4.36	2.54	6.17	4.31	4.31	3.81	4.87
Bourgogne-Franche-Comté	HPV16/18	Organised	2023		190	1.58	2.76	2.63	0.53	5.26	2.72	2.71	2.20	3.33
Bretagne	HPV16/18	Opportunistic	2023		3,450	5.19	4.74	4.72	3.97	5.57	4.74	4.73	4.35	5.16
Bretagne	HPV16/18	Organised	2023		1,597	2.44	2.58	2.57	1.69	3.57	2.59	2.57	2.15	3.11
Centre-Val de Loire	HPV16/18	Opportunistic	2023		5,511	4.59	4.27	4.26	3.70	4.86	4.27	4.27	4.03	4.52
Centre-Val de Loire	HPV16/18	Organised	2023		562	3.20	2.79	2.85	1.42	4.27	2.79	2.78	2.42	3.21
Corse	HPV16/18	Opportunistic	2023		5	20.00	3.77	0.00	0.00	20.00	4.08	4.02	3.08	5.35
Grand Est	HPV16/18	Opportunistic	2023		2,957	4.26	4.23	4.23	3.45	5.07	4.23	4.22	3.90	4.58
Grand Est	HPV16/18	Organised	2023		420	2.38	2.63	2.62	1.19	4.29	2.64	2.63	2.22	3.13

Table S8 Observed HR HPV cervical infection prevalence, posterior predictive HR HPV cervical infection prevalence, and posterior expected HR HPV cervical infection prevalence, stratified by various dimensions. ***(continued)***

							Posterior distribution							
							Predictive prevalence				Expected prevalence			
									ETI95%				ETI95%	
Region	Genotypes	Screening pathway	Year	Age	Number of tests	Observed	Average	Median	LB	UB	Average	Median	LB	UB
Hauts-de-France	HPV16/18	Opportunistic	2023		7,934	4.16	4.28	4.27	3.77	4.79	4.28	4.27	4.04	4.52
Hauts-de-France	HPV16/18	Organised	2023		473	2.96	2.73	2.75	1.27	4.23	2.72	2.71	2.34	3.16
Normandie	HPV16/18	Opportunistic	2023		3,472	4.87	4.90	4.90	4.12	5.73	4.91	4.91	4.58	5.25
Normandie	HPV16/18	Organised	2023		187	2.14	2.91	2.67	0.53	5.88	2.91	2.91	2.49	3.39
Nouvelle-Aquitaine	HPV16/18	Opportunistic	2023		7,539	4.24	4.48	4.48	3.97	5.03	4.49	4.49	4.23	4.75
Nouvelle-Aquitaine	HPV16/18	Organised	2023		1,611	2.98	3.03	3.04	2.17	3.97	3.05	3.04	2.65	3.48
Occitanie	HPV16/18	Opportunistic	2023		1,178	4.84	4.70	4.67	3.40	6.03	4.71	4.70	4.31	5.12
Occitanie	HPV16/18	Organised	2023		197	2.03	3.20	3.05	1.02	6.09	3.21	3.20	2.71	3.76
Pays de la Loire	HPV16/18	Opportunistic	2023		1,003	4.09	3.98	3.99	2.79	5.28	4.00	3.99	3.63	4.42
Pays de la Loire	HPV16/18	Organised	2023		498	3.21	2.55	2.41	1.20	4.22	2.55	2.55	2.16	3.00
Provence-Alpes-Côte d'Azur	HPV16/18	Opportunistic	2023		5,645	4.62	4.80	4.80	4.20	5.46	4.80	4.80	4.52	5.11
Provence-Alpes-Côte d'Azur	HPV16/18	Organised	2023		562	4.09	3.70	3.74	2.14	5.52	3.69	3.67	3.09	4.38
Île-de-France	HPV16/18	Opportunistic	2023		13,457	4.58	4.65	4.64	4.23	5.08	4.64	4.64	4.43	4.87
Île-de-France	HPV16/18	Organised	2023		1,195	3.51	3.17	3.18	2.09	4.27	3.17	3.15	2.74	3.67
Auvergne-Rhône-Alpes	Other genotypes	Opportunistic	2020		988	9.11	10.01	10.02	8.00	12.15	10.01	10.00	9.25	10.80
Bourgogne-Franche-Comté	Other genotypes	Opportunistic	2020		537	9.50	8.73	8.75	6.33	11.17	8.74	8.73	8.04	9.50
Bretagne	Other genotypes	Opportunistic	2020		559	8.77	8.59	8.59	6.26	11.09	8.61	8.61	7.87	9.40
Centre-Val de Loire	Other genotypes	Opportunistic	2020		3,667	7.06	8.40	8.40	7.42	9.38	8.39	8.39	8.03	8.76
Centre-Val de Loire	Other genotypes	Organised	2020		3	33.33	6.02	0.00	0.00	33.33	6.03	5.96	4.51	7.84
Corse	Other genotypes	Opportunistic	2020		377	10.61	10.26	10.08	6.90	14.06	10.24	10.19	8.28	12.60
Grand Est	Other genotypes	Opportunistic	2020		882	7.82	8.00	7.94	6.24	9.98	8.01	8.00	7.32	8.75
Hauts-de-France	Other genotypes	Opportunistic	2020		2,833	8.08	8.57	8.58	7.48	9.67	8.56	8.55	8.18	8.95
Hauts-de-France	Other genotypes	Organised	2020		7	0.00	6.22	0.00	0.00	28.57	6.12	6.09	4.88	7.58
Normandie	Other genotypes	Opportunistic	2020		2,313	9.21	8.80	8.78	7.57	10.07	8.80	8.80	8.35	9.27
Nouvelle-Aquitaine	Other genotypes	Opportunistic	2020		1,688	8.95	9.15	9.12	7.76	10.60	9.16	9.16	8.77	9.57
Nouvelle-Aquitaine	Other genotypes	Organised	2020		1	0.00	6.10	0.00	0.00	100.00	5.80	5.65	3.58	8.90
Occitanie	Other genotypes	Opportunistic	2020		2,175	9.89	10.93	10.94	9.47	12.46	10.92	10.92	10.13	11.73
Occitanie	Other genotypes	Organised	2020		4	25.00	6.53	0.00	0.00	25.00	6.83	6.76	4.88	9.26
Pays de la Loire	Other genotypes	Opportunistic	2020		1,156	6.66	7.26	7.27	5.62	8.91	7.27	7.25	6.65	7.92
Pays de la Loire	Other genotypes	Organised	2020		5	0.00	5.35	0.00	0.00	40.00	5.50	5.44	4.24	7.05
Provence-Alpes-Côte d'Azur	Other genotypes	Opportunistic	2020		2,129	9.63	9.41	9.39	8.13	10.76	9.41	9.41	8.92	9.92
Provence-Alpes-Côte d'Azur	Other genotypes	Organised	2020		2	0.00	5.45	0.00	0.00	50.00	5.75	5.66	4.15	7.76
Île-de-France	Other genotypes	Opportunistic	2020		51,739	9.68	9.62	9.61	9.29	9.96	9.62	9.61	9.41	9.84
Île-de-France	Other genotypes	Organised	2020		16	0.00	8.10	6.25	0.00	25.00	8.22	8.18	6.74	9.90
Auvergne-Rhône-Alpes	Other genotypes	Opportunistic	2021		2,995	9.12	9.67	9.65	8.41	10.98	9.67	9.66	9.00	10.38
Auvergne-Rhône-Alpes	Other genotypes	Organised	2021		152	9.21	7.43	7.24	3.29	11.84	7.52	7.51	6.44	8.59
Bourgogne-Franche-Comté	Other genotypes	Opportunistic	2021		1,510	9.21	8.61	8.61	7.02	10.26	8.60	8.60	7.95	9.31
Bourgogne-Franche-Comté	Other genotypes	Organised	2021		27	11.11	5.68	3.70	0.00	14.81	5.89	5.87	4.97	6.92
Bretagne	Other genotypes	Opportunistic	2021		2,260	7.26	8.10	8.10	6.81	9.42	8.08	8.08	7.51	8.66

Table S8 Observed HR HPV cervical infection prevalence, posterior predictive HR HPV cervical infection prevalence, and posterior expected HR HPV cervical infection prevalence, stratified by various dimensions. ***(continued)***

							Posterior distribution							
							Predictive prevalence				Expected prevalence			
									ETI95%				ETI95%	
Region	Genotypes	Screening pathway	Year	Age	Number of tests	Observed	Average	Median	LB	UB	Average	Median	LB	UB
Bretagne	Other genotypes	Organised	2021		114	2.63	5.24	5.26	1.75	9.65	5.23	5.21	4.33	6.25
Centre-Val de Loire	Other genotypes	Opportunistic	2021		14,308	7.68	7.93	7.92	7.43	8.46	7.93	7.93	7.64	8.22
Centre-Val de Loire	Other genotypes	Organised	2021		721	5.55	6.24	6.24	4.58	8.18	6.22	6.22	5.59	6.91
Corse	Other genotypes	Opportunistic	2021		34	8.82	9.07	8.82	0.00	20.59	9.16	9.13	8.01	10.50
Corse	Other genotypes	Organised	2021		1	0.00	6.40	0.00	0.00	100.00	6.17	6.05	4.07	8.97
Grand Est	Other genotypes	Opportunistic	2021		3,787	7.10	7.87	7.84	6.97	8.82	7.88	7.87	7.45	8.34
Grand Est	Other genotypes	Organised	2021		136	2.94	5.35	5.15	2.21	9.56	5.36	5.35	4.58	6.16
Hauts-de-France	Other genotypes	Opportunistic	2021		10,358	7.81	8.34	8.33	7.74	8.96	8.34	8.34	8.05	8.65
Hauts-de-France	Other genotypes	Organised	2021		390	6.15	6.45	6.41	3.85	8.97	6.43	6.42	5.71	7.19
Normandie	Other genotypes	Opportunistic	2021		8,045	8.39	8.60	8.60	7.89	9.30	8.59	8.59	8.23	8.96
Normandie	Other genotypes	Organised	2021		34	8.82	5.93	5.88	0.00	14.71	6.00	5.99	5.18	6.85
Nouvelle-Aquitaine	Other genotypes	Opportunistic	2021		8,526	8.40	8.50	8.49	7.83	9.17	8.50	8.50	8.18	8.83
Nouvelle-Aquitaine	Other genotypes	Organised	2021		391	6.65	6.11	6.14	3.83	8.70	6.14	6.13	5.45	6.84
Occitanie	Other genotypes	Opportunistic	2021		5,534	9.78	9.56	9.56	8.64	10.46	9.56	9.56	9.09	10.05
Occitanie	Other genotypes	Organised	2021		305	8.52	7.70	7.54	4.92	10.82	7.75	7.73	6.75	8.84
Pays de la Loire	Other genotypes	Opportunistic	2021		2,811	6.19	7.03	7.04	5.98	8.11	7.03	7.02	6.53	7.58
Pays de la Loire	Other genotypes	Organised	2021		270	4.44	5.24	5.19	2.59	8.15	5.25	5.24	4.49	6.07
Provence-Alpes-Côte d'Azur	Other genotypes	Opportunistic	2021		11,257	9.16	9.14	9.14	8.55	9.79	9.14	9.14	8.80	9.50
Provence-Alpes-Côte d'Azur	Other genotypes	Organised	2021		56	7.14	7.97	7.14	1.79	16.07	7.94	7.90	6.85	9.19
Île-de-France	Other genotypes	Opportunistic	2021		83,161	9.32	9.12	9.12	8.87	9.38	9.12	9.12	8.96	9.30
Île-de-France	Other genotypes	Organised	2021		1,999	6.40	6.53	6.50	5.25	7.90	6.54	6.52	5.81	7.35
Auvergne-Rhône-Alpes	Other genotypes	Opportunistic	2022		247	19.43	11.17	10.93	7.29	15.38	11.22	11.21	10.20	12.28
Auvergne-Rhône-Alpes	Other genotypes	Organised	2022		48	12.50	8.40	8.33	2.08	16.67	8.38	8.36	7.12	9.76
Bourgogne-Franche-Comté	Other genotypes	Opportunistic	2022		1,175	9.36	9.24	9.19	7.49	11.15	9.25	9.24	8.51	10.03
Bourgogne-Franche-Comté	Other genotypes	Organised	2022		45	4.44	7.83	6.67	2.17	15.56	7.89	7.88	6.67	9.21
Bretagne	Other genotypes	Opportunistic	2022		2,496	9.70	9.24	9.25	7.93	10.58	9.23	9.23	8.62	9.85
Bretagne	Other genotypes	Organised	2022		586	4.44	5.79	5.80	3.92	8.02	5.81	5.77	4.95	6.78
Centre-Val de Loire	Other genotypes	Opportunistic	2022		7,059	8.85	8.39	8.40	7.65	9.12	8.39	8.39	8.05	8.72
Centre-Val de Loire	Other genotypes	Organised	2022		918	7.08	6.33	6.32	4.68	8.06	6.32	6.32	5.77	6.91
Corse	Other genotypes	Opportunistic	2022		11	27.27	10.19	9.09	0.00	27.27	9.88	9.85	8.51	11.43
Corse	Other genotypes	Organised	2022		2	0.00	6.48	0.00	0.00	50.00	6.92	6.82	5.15	9.08
Grand Est	Other genotypes	Opportunistic	2022		3,082	9.34	8.90	8.89	7.75	10.06	8.90	8.89	8.39	9.45
Grand Est	Other genotypes	Organised	2022		622	7.88	6.69	6.59	4.82	8.84	6.70	6.69	5.89	7.54
Hauts-de-France	Other genotypes	Opportunistic	2022		8,863	9.20	8.97	8.97	8.29	9.65	8.97	8.96	8.64	9.30
Hauts-de-France	Other genotypes	Organised	2022		1,080	6.48	6.63	6.57	5.09	8.24	6.62	6.61	6.00	7.30
Normandie	Other genotypes	Opportunistic	2022		5,759	9.20	9.46	9.45	8.56	10.38	9.45	9.45	9.03	9.87
Normandie	Other genotypes	Organised	2022		153	4.58	6.32	5.88	2.61	10.46	6.26	6.25	5.55	7.03
Nouvelle-Aquitaine	Other genotypes	Opportunistic	2022		9,232	8.99	9.25	9.25	8.57	9.92	9.26	9.26	8.92	9.62
Nouvelle-Aquitaine	Other genotypes	Organised	2022		1,733	7.44	6.93	6.92	5.71	8.31	6.92	6.91	6.35	7.54

Table S8 **Observed HR HPV cervical infection prevalence, posterior predictive HR HPV cervical infection prevalence, and posterior expected HR HPV cervical infection prevalence, stratified by various dimensions.** ***(continued)***

							Posterior distribution							
							Predictive prevalence				Expected prevalence			
									ETI95%				ETI95%	
Region	Genotypes	Screening pathway	Year	Age	Number of tests	Observed	Average	Median	LB	UB	Average	Median	LB	UB
Occitanie	Other genotypes	Opportunistic	2022		2,624	9.83	9.67	9.64	8.38	10.94	9.67	9.66	9.12	10.24
Occitanie	Other genotypes	Organised	2022		507	8.09	8.00	7.89	5.52	10.45	7.99	7.97	7.17	8.91
Pays de la Loire	Other genotypes	Opportunistic	2022		721	9.71	8.18	8.18	6.24	10.26	8.18	8.18	7.56	8.87
Pays de la Loire	Other genotypes	Organised	2022		241	4.56	5.87	5.81	2.90	9.13	5.85	5.83	5.15	6.60
Provence-Alpes-Côte d'Azur	Other genotypes	Opportunistic	2022		7,734	9.53	9.89	9.88	9.13	10.62	9.89	9.89	9.51	10.30
Provence-Alpes-Côte d'Azur	Other genotypes	Organised	2022		504	8.73	9.45	9.33	6.75	12.50	9.51	9.45	8.36	10.90
Île-de-France	Other genotypes	Opportunistic	2022		15,516	9.36	9.95	9.95	9.40	10.49	9.95	9.95	9.67	10.24
Île-de-France	Other genotypes	Organised	2022		1,307	8.11	7.49	7.50	5.97	9.10	7.50	7.50	6.87	8.20
Auvergne-Rhône-Alpes	Other genotypes	Opportunistic	2023		192	14.58	13.58	13.54	8.33	18.75	13.52	13.51	11.81	15.33
Auvergne-Rhône-Alpes	Other genotypes	Organised	2023		49	14.29	9.71	10.20	2.04	18.37	9.84	9.81	8.15	11.79
Bourgogne-Franche-Comté	Other genotypes	Opportunistic	2023		551	12.52	10.90	10.89	8.34	13.79	10.88	10.87	9.86	11.95
Bourgogne-Franche-Comté	Other genotypes	Organised	2023		190	6.84	6.66	6.84	3.16	10.53	6.68	6.66	5.60	7.91
Bretagne	Other genotypes	Opportunistic	2023		3,450	11.77	10.93	10.93	9.71	12.23	10.94	10.93	10.25	11.65
Bretagne	Other genotypes	Organised	2023		1,597	5.39	6.11	6.07	4.76	7.58	6.10	6.08	5.30	7.02
Centre-Val de Loire	Other genotypes	Opportunistic	2023		5,511	10.03	10.08	10.07	9.22	10.98	10.09	10.09	9.69	10.52
Centre-Val de Loire	Other genotypes	Organised	2023		562	6.41	6.67	6.58	4.63	8.90	6.71	6.70	6.06	7.41
Corse	Other genotypes	Opportunistic	2023		5	20.00	8.98	0.00	0.00	40.00	9.04	8.94	6.97	11.58
Grand Est	Other genotypes	Opportunistic	2023		2,957	10.62	10.28	10.28	9.03	11.53	10.27	10.27	9.67	10.90
Grand Est	Other genotypes	Organised	2023		420	4.52	6.49	6.43	4.28	9.05	6.53	6.51	5.68	7.44
Hauts-de-France	Other genotypes	Opportunistic	2023		7,934	10.55	10.29	10.28	9.53	11.10	10.29	10.30	9.89	10.70
Hauts-de-France	Other genotypes	Organised	2023		473	5.92	6.68	6.55	4.44	9.30	6.73	6.72	5.99	7.54
Normandie	Other genotypes	Opportunistic	2023		3,472	10.11	10.85	10.83	9.71	12.04	10.86	10.86	10.36	11.37
Normandie	Other genotypes	Organised	2023		187	6.42	6.77	6.42	3.21	10.70	6.77	6.77	6.02	7.58
Nouvelle-Aquitaine	Other genotypes	Opportunistic	2023		7,539	11.42	11.00	10.98	10.20	11.83	10.99	10.98	10.58	11.43
Nouvelle-Aquitaine	Other genotypes	Organised	2023		1,611	7.51	7.53	7.51	6.08	9.00	7.55	7.54	6.88	8.28
Occitanie	Other genotypes	Opportunistic	2023		1,178	11.54	11.43	11.38	9.51	13.33	11.42	11.40	10.70	12.22
Occitanie	Other genotypes	Organised	2023		197	8.12	7.87	7.61	4.57	12.18	7.92	7.91	6.98	8.96
Pays de la Loire	Other genotypes	Opportunistic	2023		1,003	10.17	9.33	9.27	7.48	11.37	9.35	9.33	8.62	10.17
Pays de la Loire	Other genotypes	Organised	2023		498	5.42	6.15	6.02	4.02	8.43	6.16	6.14	5.43	6.98
Provence-Alpes-Côte d'Azur	Other genotypes	Opportunistic	2023		5,645	11.73	11.63	11.64	10.68	12.60	11.64	11.64	11.16	12.12
Provence-Alpes-Côte d'Azur	Other genotypes	Organised	2023		562	10.14	9.08	9.07	6.41	11.92	9.13	9.10	7.97	10.52
Île-de-France	Other genotypes	Opportunistic	2023		13,457	11.11	12.05	12.05	11.41	12.72	12.05	12.04	11.70	12.41
Île-de-France	Other genotypes	Organised	2023		1,195	8.79	8.11	8.12	6.44	9.87	8.12	8.11	7.36	9.01
Auvergne-Rhône-Alpes	HPV16/18	Opportunistic		[30,39]			4.84	4.87	3.80	5.93	4.83	4.83	4.42	5.26
Auvergne-Rhône-Alpes	HPV16/18	Organised		[30,39]			4.42	4.55	0.00	10.61	4.40	4.37	3.67	5.25
Bourgogne-Franche-Comté	HPV16/18	Opportunistic		[30,39]			4.47	4.47	3.40	5.61	4.46	4.45	4.03	4.93
Bourgogne-Franche-Comté	HPV16/18	Organised		[30,39]			3.44	2.90	0.00	8.70	3.45	3.43	2.82	4.17
Bretagne	HPV16/18	Opportunistic		[30,39]			5.10	5.09	4.28	5.95	5.11	5.10	4.69	5.55
Bretagne	HPV16/18	Organised		[30,39]			3.20	3.17	1.81	4.68	3.20	3.17	2.61	3.89

Table S8 Observed HR HPV cervical infection prevalence, posterior predictive HR HPV cervical infection prevalence, and posterior expected HR HPV cervical infection prevalence, stratified by various dimensions. ***(continued)***

							Posterior distribution							
							Predictive prevalence				Expected prevalence			
									ETI95%				ETI95%	
Region	Genotypes	Screening pathway	Year	Age	Number of tests	Observed	Average	Median	LB	UB	Average	Median	LB	UB
Centre-Val de Loire	HPV16/18	Opportunistic		[30,39]			4.54	4.54	4.06	5.02	4.54	4.54	4.32	4.77
Centre-Val de Loire	HPV16/18	Organised		[30,39]			3.52	3.39	1.96	5.18	3.52	3.51	3.03	4.05
Corse	HPV16/18	Opportunistic		[30,39]			4.32	4.55	0.91	9.09	4.32	4.30	3.48	5.38
Grand Est	HPV16/18	Opportunistic		[30,39]			4.53	4.54	3.78	5.30	4.53	4.52	4.21	4.88
Grand Est	HPV16/18	Organised		[30,39]			3.29	3.35	1.52	5.49	3.29	3.28	2.79	3.86
Hauts-de-France	HPV16/18	Opportunistic		[30,39]			4.45	4.45	4.04	4.88	4.45	4.45	4.22	4.67
Hauts-de-France	HPV16/18	Organised		[30,39]			3.42	3.48	2.05	4.90	3.44	3.43	2.94	3.96
Normandie	HPV16/18	Opportunistic		[30,39]			5.09	5.08	4.52	5.70	5.09	5.09	4.79	5.39
Normandie	HPV16/18	Organised		[30,39]			3.61	2.99	0.00	8.96	3.67	3.67	3.13	4.25
Nouvelle-Aquitaine	HPV16/18	Opportunistic		[30,39]			4.80	4.81	4.31	5.31	4.80	4.80	4.54	5.05
Nouvelle-Aquitaine	HPV16/18	Organised		[30,39]			3.68	3.60	2.26	5.19	3.71	3.70	3.19	4.29
Occitanie	HPV16/18	Opportunistic		[30,39]			5.09	5.08	4.30	5.89	5.09	5.09	4.76	5.42
Occitanie	HPV16/18	Organised		[30,39]			4.19	3.95	1.75	7.02	4.19	4.18	3.57	4.87
Pays de la Loire	HPV16/18	Opportunistic		[30,39]			3.92	3.90	3.04	4.86	3.91	3.91	3.58	4.25
Pays de la Loire	HPV16/18	Organised		[30,39]			3.10	3.05	1.15	5.34	3.11	3.10	2.62	3.65
Provence-Alpes-Côte d'Azur	HPV16/18	Opportunistic		[30,39]			5.05	5.04	4.55	5.54	5.05	5.04	4.78	5.33
Provence-Alpes-Côte d'Azur	HPV16/18	Organised		[30,39]			4.47	4.51	2.58	6.65	4.45	4.44	3.72	5.33
Île-de-France	HPV16/18	Opportunistic		[30,39]			4.40	4.40	4.19	4.61	4.40	4.40	4.27	4.54
Île-de-France	HPV16/18	Organised		[30,39]			3.87	3.81	2.69	5.20	3.86	3.85	3.32	4.46
Auvergne-Rhône-Alpes	HPV16/18	Opportunistic		(39,49]	1,178	3.57	3.97	3.99	2.89	5.18	3.97	3.97	3.64	4.33
Auvergne-Rhône-Alpes	HPV16/18	Organised		(39,49]	80	5.00	3.68	3.75	0.00	8.75	3.73	3.71	3.15	4.39
Bourgogne-Franche-Comté	HPV16/18	Opportunistic		(39,49]	1,030	3.30	3.69	3.69	2.52	4.95	3.67	3.67	3.31	4.07
Bourgogne-Franche-Comté	HPV16/18	Organised		(39,49]	66	1.52	2.86	3.03	0.00	7.58	2.84	2.83	2.30	3.48
Bretagne	HPV16/18	Opportunistic		(39,49]	2,375	4.38	4.17	4.17	3.37	5.05	4.17	4.16	3.84	4.54
Bretagne	HPV16/18	Organised		(39,49]	595	3.36	2.60	2.52	1.34	4.04	2.61	2.59	2.16	3.14
Centre-Val de Loire	HPV16/18	Opportunistic		(39,49]	8,774	3.99	3.70	3.70	3.28	4.16	3.71	3.70	3.52	3.91
Centre-Val de Loire	HPV16/18	Organised		(39,49]	519	3.08	2.92	2.89	1.54	4.43	2.93	2.92	2.57	3.30
Corse	HPV16/18	Opportunistic		(39,49]	127	4.72	3.62	3.15	0.79	7.09	3.56	3.53	2.76	4.55
Corse	HPV16/18	Organised		(39,49]	2	0.00	3.17	0.00	0.00	50.00	3.15	3.11	2.28	4.23
Grand Est	HPV16/18	Opportunistic		(39,49]	3,035	4.09	3.76	3.76	3.03	4.51	3.76	3.75	3.48	4.05
Grand Est	HPV16/18	Organised		(39,49]	325	2.77	2.78	2.77	1.23	4.92	2.76	2.75	2.35	3.20
Hauts-de-France	HPV16/18	Opportunistic		(39,49]	8,207	4.37	3.71	3.72	3.29	4.17	3.71	3.71	3.52	3.91
Hauts-de-France	HPV16/18	Organised		(39,49]	498	2.01	2.86	2.81	1.41	4.42	2.85	2.85	2.48	3.25
Normandie	HPV16/18	Opportunistic		(39,49]	5,887	4.33	4.18	4.16	3.60	4.79	4.18	4.18	3.92	4.43
Normandie	HPV16/18	Organised		(39,49]	69	1.45	3.16	2.90	0.00	7.25	3.13	3.12	2.70	3.60
Nouvelle-Aquitaine	HPV16/18	Opportunistic		(39,49]	7,804	3.86	3.96	3.96	3.47	4.45	3.96	3.95	3.74	4.18
Nouvelle-Aquitaine	HPV16/18	Organised		(39,49]	781	3.71	3.09	3.07	1.92	4.35	3.09	3.08	2.71	3.51
Occitanie	HPV16/18	Opportunistic		(39,49]	3,365	4.04	4.22	4.22	3.51	4.99	4.24	4.23	3.96	4.52
Occitanie	HPV16/18	Organised		(39,49]	296	2.36	3.48	3.38	1.35	5.74	3.50	3.49	3.01	4.01

Table S8 Observed HR HPV cervical infection prevalence, posterior predictive HR HPV cervical infection prevalence, and posterior expected HR HPV cervical infection prevalence, stratified by various dimensions. ***(continued)***

							Posterior distribution							
							Predictive prevalence				Expected prevalence			
									ETI95%				ETI95%	
Region	Genotypes	Screening pathway	Year	Age	Number of tests	Observed	Average	Median	LB	UB	Average	Median	LB	UB
Pays de la Loire	HPV16/18	Opportunistic		(39,49]	1,705	2.93	3.26	3.23	2.40	4.16	3.26	3.26	2.99	3.56
Pays de la Loire	HPV16/18	Organised		(39,49]	288	3.12	2.58	2.43	1.04	4.51	2.59	2.59	2.23	3.00
Provence-Alpes-Côte d'Azur	HPV16/18	Opportunistic		(39,49]	7,397	4.24	4.09	4.10	3.58	4.58	4.09	4.08	3.85	4.32
Provence-Alpes-Côte d'Azur	HPV16/18	Organised		(39,49]	258	3.49	3.70	3.49	1.55	6.20	3.70	3.69	3.14	4.37
Île-de-France	HPV16/18	Opportunistic		(39,49]	49,538	3.34	3.56	3.56	3.36	3.77	3.56	3.56	3.44	3.69
Île-de-France	HPV16/18	Organised		(39,49]	790	2.41	3.16	3.16	1.90	4.43	3.15	3.15	2.76	3.59
Auvergne-Rhône-Alpes	HPV16/18	Opportunistic		(49,59]	975	3.18	3.17	3.18	2.05	4.41	3.20	3.19	2.91	3.50
Auvergne-Rhône-Alpes	HPV16/18	Organised		(49,59]	60	1.67	3.01	3.33	0.00	8.33	3.06	3.05	2.57	3.62
Bourgogne-Franche-Comté	HPV16/18	Opportunistic		(49,59]	770	2.60	2.99	2.99	1.82	4.29	2.98	2.98	2.70	3.30
Bourgogne-Franche-Comté	HPV16/18	Organised		(49,59]	56	0.00	2.41	1.79	0.00	7.14	2.35	2.34	1.86	2.93
Bretagne	HPV16/18	Opportunistic		(49,59]	1,948	3.08	3.30	3.29	2.52	4.16	3.30	3.29	3.01	3.60
Bretagne	HPV16/18	Organised		(49,59]	569	2.11	2.16	2.11	1.05	3.51	2.16	2.15	1.78	2.61
Centre-Val de Loire	HPV16/18	Opportunistic		(49,59]	8,009	2.58	2.96	2.96	2.56	3.38	2.96	2.96	2.80	3.14
Centre-Val de Loire	HPV16/18	Organised		(49,59]	673	2.67	2.44	2.38	1.34	3.71	2.44	2.43	2.14	2.77
Corse	HPV16/18	Opportunistic		(49,59]	122	2.46	2.84	2.46	0.00	6.56	2.79	2.77	2.18	3.54
Corse	HPV16/18	Organised		(49,59]	1	0.00	2.37	0.00	0.00	0.00	2.84	2.77	1.84	4.21
Grand Est	HPV16/18	Opportunistic		(49,59]	2,742	2.88	3.01	3.03	2.33	3.72	3.00	2.99	2.76	3.23
Grand Est	HPV16/18	Organised		(49,59]	308	2.27	2.30	2.27	0.65	4.22	2.33	2.32	1.98	2.71
Hauts-de-France	HPV16/18	Opportunistic		(49,59]	5,919	3.26	2.95	2.94	2.52	3.43	2.96	2.96	2.80	3.13
Hauts-de-France	HPV16/18	Organised		(49,59]	481	2.91	2.35	2.29	1.04	3.74	2.36	2.35	2.06	2.70
Normandie	HPV16/18	Opportunistic		(49,59]	4,371	3.52	3.28	3.27	2.72	3.87	3.27	3.27	3.07	3.49
Normandie	HPV16/18	Organised		(49,59]	56	1.79	2.64	1.79	0.00	7.14	2.56	2.55	2.20	2.97
Nouvelle-Aquitaine	HPV16/18	Opportunistic		(49,59]	6,164	3.28	3.16	3.15	2.71	3.62	3.16	3.16	2.98	3.34
Nouvelle-Aquitaine	HPV16/18	Organised		(49,59]	1,270	2.36	2.55	2.52	1.73	3.46	2.56	2.55	2.25	2.90
Occitanie	HPV16/18	Opportunistic		(49,59]	2,922	3.70	3.39	3.39	2.74	4.11	3.40	3.39	3.16	3.64
Occitanie	HPV16/18	Organised		(49,59]	299	1.67	3.01	3.01	1.34	5.02	3.01	3.01	2.62	3.46
Pays de la Loire	HPV16/18	Opportunistic		(49,59]	1,413	2.34	2.65	2.62	1.84	3.54	2.65	2.65	2.42	2.90
Pays de la Loire	HPV16/18	Organised		(49,59]	275	2.18	2.23	2.18	0.73	4.00	2.23	2.22	1.91	2.57
Provence-Alpes-Côte d'Azur	HPV16/18	Opportunistic		(49,59]	6,214	3.07	3.25	3.25	2.77	3.77	3.25	3.25	3.06	3.46
Provence-Alpes-Côte d'Azur	HPV16/18	Organised		(49,59]	223	3.14	3.04	3.14	0.90	5.38	3.05	3.03	2.56	3.61
Île-de-France	HPV16/18	Opportunistic		(49,59]	37,058	2.79	2.85	2.84	2.64	3.07	2.84	2.84	2.73	2.96
Île-de-France	HPV16/18	Organised		(49,59]	926	2.70	2.55	2.48	1.51	3.67	2.56	2.55	2.24	2.92
Auvergne-Rhône-Alpes	HPV16/18	Opportunistic		(59,66]	481	1.46	2.96	2.91	1.46	4.57	2.96	2.95	2.67	3.26
Auvergne-Rhône-Alpes	HPV16/18	Organised		(59,66]	43	0.00	3.02	2.33	0.00	9.30	2.96	2.94	2.42	3.55
Bourgogne-Franche-Comté	HPV16/18	Opportunistic		(59,66]	475	3.79	2.72	2.74	1.47	4.21	2.73	2.73	2.45	3.04
Bourgogne-Franche-Comté	HPV16/18	Organised		(59,66]	71	4.23	2.32	1.41	0.00	7.04	2.37	2.36	1.94	2.89
Bretagne	HPV16/18	Opportunistic		(59,66]	981	3.47	3.03	3.06	2.04	4.18	3.03	3.02	2.73	3.33
Bretagne	HPV16/18	Organised		(59,66]	470	1.49	2.08	2.13	0.85	3.62	2.09	2.08	1.66	2.60
Centre-Val de Loire	HPV16/18	Opportunistic		(59,66]	4,019	2.74	2.73	2.71	2.21	3.26	2.73	2.72	2.54	2.93

Table S8 Observed HR HPV cervical infection prevalence, posterior predictive HR HPV cervical infection prevalence, and posterior expected HR HPV cervical infection prevalence, stratified by various dimensions. ***(continued)***

							Posterior distribution							
							Predictive prevalence				Expected prevalence			
									ETI95%				ETI95%	
Region	Genotypes	Screening pathway	Year	Age	Number of tests	Observed	Average	Median	LB	UB	Average	Median	LB	UB
Centre-Val de Loire	HPV16/18	Organised		(59,66]	452	2.88	2.29	2.21	1.11	3.76	2.31	2.30	1.96	2.71
Corse	HPV16/18	Opportunistic		(59,66]	68	1.47	2.72	2.94	0.00	7.35	2.72	2.69	2.20	3.37
Grand Est	HPV16/18	Opportunistic		(59,66]	1,385	2.45	2.77	2.74	1.95	3.68	2.78	2.77	2.54	3.03
Grand Est	HPV16/18	Organised		(59,66]	217	1.38	2.28	2.30	0.46	4.61	2.29	2.28	1.87	2.76
Hauts-de-France	HPV16/18	Opportunistic		(59,66]	2,757	2.32	2.76	2.76	2.14	3.41	2.76	2.76	2.57	2.97
Hauts-de-France	HPV16/18	Organised		(59,66]	338	3.25	2.31	2.37	0.89	4.14	2.33	2.32	1.96	2.75
Normandie	HPV16/18	Opportunistic		(59,66]	2,205	2.99	3.01	2.99	2.31	3.72	3.00	3.00	2.77	3.25
Normandie	HPV16/18	Organised		(59,66]	182	3.30	2.47	2.20	0.55	4.95	2.47	2.45	2.05	2.95
Nouvelle-Aquitaine	HPV16/18	Opportunistic		(59,66]	3,387	3.16	2.91	2.89	2.33	3.54	2.92	2.92	2.72	3.13
Nouvelle-Aquitaine	HPV16/18	Organised		(59,66]	934	2.57	2.50	2.46	1.39	3.64	2.50	2.49	2.13	2.93
Occitanie	HPV16/18	Opportunistic		(59,66]	1,524	3.54	3.14	3.15	2.29	4.13	3.15	3.14	2.90	3.41
Occitanie	HPV16/18	Organised		(59,66]	190	1.58	2.85	2.63	0.53	5.26	2.87	2.85	2.41	3.41
Pays de la Loire	HPV16/18	Opportunistic		(59,66]	699	2.72	2.61	2.58	1.43	3.86	2.60	2.60	2.34	2.88
Pays de la Loire	HPV16/18	Organised		(59,66]	189	1.59	2.19	2.12	0.53	4.76	2.17	2.16	1.80	2.58
Provence-Alpes-Côte d'Azur	HPV16/18	Opportunistic		(59,66]	3,024	2.84	3.02	3.01	2.38	3.70	3.03	3.02	2.81	3.26
Provence-Alpes-Côte d'Azur	HPV16/18	Organised		(59,66]	177	4.52	2.89	2.82	0.56	5.65	2.87	2.86	2.37	3.49
Île-de-France	HPV16/18	Opportunistic		(59,66]	16,364	2.73	2.65	2.65	2.37	2.94	2.65	2.65	2.50	2.81
Île-de-France	HPV16/18	Organised		(59,66]	1,724	2.61	2.58	2.55	1.74	3.54	2.58	2.57	2.15	3.08
Auvergne-Rhône-Alpes	Other genotypes	Opportunistic		[30,39]			12.81	12.81	11.13	14.60	12.81	12.80	11.95	13.71
Auvergne-Rhône-Alpes	Other genotypes	Organised		[30,39]			10.41	10.61	3.03	18.18	10.40	10.41	9.00	11.86
Bourgogne-Franche-Comté	Other genotypes	Opportunistic		[30,39]			11.79	11.75	9.88	13.75	11.81	11.80	10.93	12.80
Bourgogne-Franche-Comté	Other genotypes	Organised		[30,39]			8.66	8.70	2.90	15.94	8.79	8.75	7.43	10.32
Bretagne	Other genotypes	Opportunistic		[30,39]			12.41	12.42	11.10	13.78	12.39	12.39	11.65	13.16
Bretagne	Other genotypes	Organised		[30,39]			7.68	7.54	5.58	10.11	7.67	7.63	6.59	8.93
Centre-Val de Loire	Other genotypes	Opportunistic		[30,39]			11.44	11.43	10.71	12.14	11.43	11.43	11.05	11.83
Centre-Val de Loire	Other genotypes	Organised		[30,39]			8.16	8.21	5.89	10.54	8.16	8.15	7.40	9.02
Corse	Other genotypes	Opportunistic		[30,39]			13.54	13.64	7.27	20.00	13.59	13.53	11.37	16.20
Grand Est	Other genotypes	Opportunistic		[30,39]			11.69	11.68	10.43	12.92	11.69	11.68	11.08	12.33
Grand Est	Other genotypes	Organised		[30,39]			8.04	7.93	5.18	11.28	8.04	8.03	7.01	9.09
Hauts-de-France	Other genotypes	Opportunistic		[30,39]			11.35	11.35	10.70	12.00	11.35	11.35	10.97	11.74
Hauts-de-France	Other genotypes	Organised		[30,39]			8.15	8.06	5.85	10.74	8.15	8.14	7.29	9.05
Normandie	Other genotypes	Opportunistic		[30,39]			12.12	12.12	11.25	13.04	12.11	12.11	11.62	12.61
Normandie	Other genotypes	Organised		[30,39]			8.52	8.96	2.99	14.93	8.55	8.54	7.57	9.59
Nouvelle-Aquitaine	Other genotypes	Opportunistic		[30,39]			12.45	12.44	11.67	13.26	12.44	12.43	12.02	12.87
Nouvelle-Aquitaine	Other genotypes	Organised		[30,39]			9.45	9.45	7.19	11.72	9.48	9.46	8.56	10.44
Occitanie	Other genotypes	Opportunistic		[30,39]			13.22	13.22	11.92	14.46	13.22	13.21	12.62	13.88
Occitanie	Other genotypes	Organised		[30,39]			10.13	10.09	6.14	14.47	10.11	10.08	9.04	11.31
Pays de la Loire	Other genotypes	Opportunistic		[30,39]			9.91	9.93	8.43	11.47	9.93	9.91	9.27	10.63
Pays de la Loire	Other genotypes	Organised		[30,39]			7.27	7.25	4.20	10.69	7.34	7.33	6.47	8.26

Table S8 Observed HR HPV cervical infection prevalence, posterior predictive HR HPV cervical infection prevalence, and posterior expected HR HPV cervical infection prevalence, stratified by various dimensions. ***(continued)***

							Posterior distribution							
							Predictive prevalence				Expected prevalence			
									ETI95%				ETI95%	
Region	Genotypes	Screening pathway	Year	Age	Number of tests	Observed	Average	Median	LB	UB	Average	Median	LB	UB
Provence-Alpes-Côte d'Azur	Other genotypes	Opportunistic		[30,39]			12.95	12.94	12.17	13.77	12.94	12.94	12.50	13.40
Provence-Alpes-Côte d'Azur	Other genotypes	Organised		[30,39]			11.25	11.16	8.15	14.59	11.31	11.26	9.90	12.99
Île-de-France	Other genotypes	Opportunistic		[30,39]			12.54	12.54	12.21	12.90	12.54	12.55	12.33	12.77
Île-de-France	Other genotypes	Organised		[30,39]			9.98	9.94	7.99	12.07	10.01	9.99	9.03	11.05
Auvergne-Rhône-Alpes	Other genotypes	Opportunistic		(39,49]	1,178	8.32	8.97	8.91	7.22	10.78	8.97	8.95	8.33	9.64
Auvergne-Rhône-Alpes	Other genotypes	Organised		(39,49]	80	10.00	7.71	7.50	2.50	13.75	7.79	7.79	6.73	8.89
Bourgogne-Franche-Comté	Other genotypes	Opportunistic		(39,49]	1,030	8.93	8.24	8.25	6.50	10.10	8.22	8.21	7.58	8.91
Bourgogne-Franche-Comté	Other genotypes	Organised		(39,49]	66	4.55	6.58	6.06	1.52	13.64	6.59	6.56	5.49	7.84
Bretagne	Other genotypes	Opportunistic		(39,49]	2,375	9.81	8.66	8.67	7.45	9.98	8.66	8.65	8.12	9.22
Bretagne	Other genotypes	Organised		(39,49]	595	5.55	5.60	5.55	3.70	7.56	5.64	5.62	4.87	6.47
Centre-Val de Loire	Other genotypes	Opportunistic		(39,49]	8,774	8.09	7.95	7.94	7.31	8.58	7.95	7.95	7.66	8.24
Centre-Val de Loire	Other genotypes	Organised		(39,49]	519	5.59	6.20	6.17	4.05	8.48	6.21	6.21	5.64	6.80
Corse	Other genotypes	Opportunistic		(39,49]	127	14.96	10.21	10.24	4.72	15.75	10.15	10.10	8.20	12.51
Corse	Other genotypes	Organised		(39,49]	2	0.00	6.48	0.00	0.00	50.00	6.92	6.82	5.15	9.08
Grand Est	Other genotypes	Opportunistic		(39,49]	3,035	9.13	8.31	8.30	7.25	9.42	8.32	8.31	7.87	8.82
Grand Est	Other genotypes	Organised		(39,49]	325	7.38	6.13	6.15	3.69	8.93	6.18	6.17	5.42	7.00
Hauts-de-France	Other genotypes	Opportunistic		(39,49]	8,207	8.87	8.03	8.03	7.35	8.69	8.03	8.03	7.73	8.32
Hauts-de-France	Other genotypes	Organised		(39,49]	498	5.02	6.19	6.22	4.02	8.43	6.17	6.16	5.54	6.80
Normandie	Other genotypes	Opportunistic		(39,49]	5,887	8.26	8.54	8.53	7.73	9.38	8.55	8.54	8.17	8.92
Normandie	Other genotypes	Organised		(39,49]	69	10.14	6.64	5.80	1.45	13.04	6.72	6.72	5.96	7.52
Nouvelle-Aquitaine	Other genotypes	Opportunistic		(39,49]	7,804	8.73	8.75	8.75	8.05	9.46	8.76	8.75	8.44	9.09
Nouvelle-Aquitaine	Other genotypes	Organised		(39,49]	781	8.32	7.14	7.17	5.25	9.09	7.14	7.12	6.48	7.85
Occitanie	Other genotypes	Opportunistic		(39,49]	3,365	9.27	9.52	9.51	8.47	10.64	9.51	9.51	9.04	10.00
Occitanie	Other genotypes	Organised		(39,49]	296	6.08	7.49	7.43	4.73	10.81	7.55	7.54	6.73	8.47
Pays de la Loire	Other genotypes	Opportunistic		(39,49]	1,705	7.10	7.15	7.16	5.87	8.50	7.15	7.14	6.66	7.68
Pays de la Loire	Other genotypes	Organised		(39,49]	288	4.17	5.56	5.56	3.12	8.33	5.52	5.51	4.88	6.19
Provence-Alpes-Côte d'Azur	Other genotypes	Opportunistic		(39,49]	7,397	8.85	8.98	8.98	8.27	9.71	8.98	8.98	8.64	9.33
Provence-Alpes-Côte d'Azur	Other genotypes	Organised		(39,49]	258	8.14	8.30	8.14	4.65	12.02	8.34	8.30	7.29	9.62
Île-de-France	Other genotypes	Opportunistic		(39,49]	49,538	8.31	8.68	8.68	8.37	8.99	8.67	8.67	8.49	8.86
Île-de-France	Other genotypes	Organised		(39,49]	790	7.97	7.27	7.22	5.44	9.37	7.30	7.29	6.65	8.04
Auvergne-Rhône-Alpes	Other genotypes	Opportunistic		(49,59]	975	7.49	7.67	7.69	5.95	9.54	7.66	7.65	7.11	8.24
Auvergne-Rhône-Alpes	Other genotypes	Organised		(49,59]	60	10.00	6.94	6.67	1.67	15.00	7.10	7.09	6.13	8.16
Bourgogne-Franche-Comté	Other genotypes	Opportunistic		(49,59]	770	7.40	7.03	7.01	5.19	8.96	7.03	7.02	6.49	7.60
Bourgogne-Franche-Comté	Other genotypes	Organised		(49,59]	56	7.14	6.05	5.36	0.00	12.50	6.02	5.99	4.98	7.23
Bretagne	Other genotypes	Opportunistic		(49,59]	1,948	7.55	7.25	7.24	6.06	8.52	7.25	7.25	6.77	7.73
Bretagne	Other genotypes	Organised		(49,59]	569	4.57	5.21	5.27	3.34	7.38	5.20	5.17	4.48	6.03
Centre-Val de Loire	Other genotypes	Opportunistic		(49,59]	8,009	6.21	6.74	6.74	6.12	7.34	6.74	6.74	6.48	7.00
Centre-Val de Loire	Other genotypes	Organised		(49,59]	673	5.50	5.69	5.65	3.86	7.58	5.70	5.69	5.19	6.26
Corse	Other genotypes	Opportunistic		(49,59]	122	5.74	8.48	8.20	3.28	13.93	8.42	8.37	6.82	10.33

Table S8 **Observed HR HPV cervical infection prevalence, posterior predictive HR HPV cervical infection prevalence, and posterior expected HR HPV cervical infection prevalence, stratified by various dimensions.** ***(continued)***

							Posterior distribution							
							Predictive prevalence				Expected prevalence			
									ETI95%				ETI95%	
Region	Genotypes	Screening pathway	Year	Age	Number of tests	Observed	Average	Median	LB	UB	Average	Median	LB	UB
Corse	Other genotypes	Organised		(49,59]	1	0.00	6.40	0.00	0.00	100.00	6.17	6.05	4.07	8.97
Grand Est	Other genotypes	Opportunistic		(49,59]	2,742	6.24	7.03	7.04	6.02	8.02	7.03	7.03	6.64	7.48
Grand Est	Other genotypes	Organised		(49,59]	308	3.90	5.76	5.84	3.25	8.44	5.74	5.73	5.05	6.48
Hauts-de-France	Other genotypes	Opportunistic		(49,59]	5,919	7.72	6.80	6.79	6.10	7.48	6.81	6.81	6.55	7.08
Hauts-de-France	Other genotypes	Organised		(49,59]	481	5.82	5.69	5.61	3.74	7.90	5.71	5.70	5.14	6.34
Normandie	Other genotypes	Opportunistic		(49,59]	4,371	6.47	7.12	7.12	6.29	7.96	7.13	7.13	6.81	7.46
Normandie	Other genotypes	Organised		(49,59]	56	3.57	6.13	5.36	0.00	12.50	6.10	6.10	5.40	6.85
Nouvelle-Aquitaine	Other genotypes	Opportunistic		(49,59]	6,164	7.54	7.41	7.40	6.73	8.10	7.41	7.41	7.13	7.72
Nouvelle-Aquitaine	Other genotypes	Organised		(49,59]	1,270	6.54	6.42	6.38	5.04	7.87	6.40	6.39	5.85	7.02
Occitanie	Other genotypes	Opportunistic		(49,59]	2,922	8.35	8.08	8.08	7.05	9.17	8.07	8.07	7.67	8.50
Occitanie	Other genotypes	Organised		(49,59]	299	6.69	7.14	7.02	4.35	10.37	7.19	7.17	6.42	8.03
Pays de la Loire	Other genotypes	Opportunistic		(49,59]	1,413	6.09	6.11	6.09	4.81	7.43	6.12	6.11	5.70	6.59
Pays de la Loire	Other genotypes	Organised		(49,59]	275	6.18	5.25	5.09	2.55	8.00	5.25	5.24	4.66	5.90
Provence-Alpes-Côte d'Azur	Other genotypes	Opportunistic		(49,59]	6,214	8.18	7.57	7.56	6.86	8.29	7.58	7.58	7.27	7.90
Provence-Alpes-Côte d'Azur	Other genotypes	Organised		(49,59]	223	7.62	7.61	7.62	4.04	11.21	7.63	7.59	6.61	8.87
Île-de-France	Other genotypes	Opportunistic		(49,59]	37,058	7.23	7.34	7.34	7.03	7.67	7.34	7.34	7.16	7.54
Île-de-France	Other genotypes	Organised		(49,59]	926	6.59	6.52	6.48	4.86	8.21	6.51	6.49	5.91	7.15
Auvergne-Rhône-Alpes	Other genotypes	Opportunistic		(59,66]	481	6.44	6.78	6.65	4.57	9.15	6.81	6.79	6.28	7.36
Auvergne-Rhône-Alpes	Other genotypes	Organised		(59,66]	43	2.33	6.72	6.98	0.00	13.95	6.76	6.75	5.77	7.84
Bourgogne-Franche-Comté	Other genotypes	Opportunistic		(59,66]	475	8.00	6.27	6.32	4.00	8.63	6.28	6.28	5.76	6.85
Bourgogne-Franche-Comté	Other genotypes	Organised		(59,66]	71	8.45	5.64	5.63	1.41	11.27	5.72	5.71	4.83	6.68
Bretagne	Other genotypes	Opportunistic		(59,66]	981	6.83	6.39	6.32	4.79	8.05	6.40	6.40	5.93	6.87
Bretagne	Other genotypes	Organised		(59,66]	470	3.83	4.99	4.89	2.98	7.23	4.99	4.98	4.21	5.88
Centre-Val de Loire	Other genotypes	Opportunistic		(59,66]	4,019	6.10	5.95	5.95	5.18	6.77	5.95	5.95	5.65	6.25
Centre-Val de Loire	Other genotypes	Organised		(59,66]	452	5.97	5.43	5.31	3.32	7.74	5.41	5.39	4.82	6.10
Corse	Other genotypes	Opportunistic		(59,66]	68	2.94	7.56	7.35	1.47	14.71	7.59	7.55	6.32	9.10
Grand Est	Other genotypes	Opportunistic		(59,66]	1,385	4.69	6.30	6.28	4.98	7.65	6.28	6.27	5.88	6.69
Grand Est	Other genotypes	Organised		(59,66]	217	4.15	5.60	5.53	2.76	8.76	5.62	5.61	4.83	6.45
Hauts-de-France	Other genotypes	Opportunistic		(59,66]	2,757	6.13	6.13	6.09	5.22	7.07	6.12	6.12	5.82	6.43
Hauts-de-France	Other genotypes	Organised		(59,66]	338	6.80	5.63	5.62	3.25	8.28	5.64	5.63	4.95	6.38
Normandie	Other genotypes	Opportunistic		(59,66]	2,205	5.80	6.28	6.26	5.26	7.35	6.28	6.28	5.95	6.63
Normandie	Other genotypes	Organised		(59,66]	182	4.40	5.83	5.49	2.75	9.34	5.77	5.75	5.01	6.62
Nouvelle-Aquitaine	Other genotypes	Opportunistic		(59,66]	3,387	6.17	6.61	6.61	5.73	7.56	6.61	6.61	6.29	6.95
Nouvelle-Aquitaine	Other genotypes	Organised		(59,66]	934	6.42	6.12	6.10	4.50	7.82	6.15	6.14	5.48	6.90
Occitanie	Other genotypes	Opportunistic		(59,66]	1,524	7.15	7.19	7.15	5.84	8.53	7.21	7.21	6.79	7.65
Occitanie	Other genotypes	Organised		(59,66]	190	8.42	6.93	6.84	3.68	10.54	6.92	6.90	6.08	7.89
Pays de la Loire	Other genotypes	Opportunistic		(59,66]	699	5.29	5.71	5.72	4.01	7.58	5.73	5.72	5.27	6.25
Pays de la Loire	Other genotypes	Organised		(59,66]	189	2.65	5.12	5.29	2.12	8.47	5.10	5.09	4.42	5.87
Provence-Alpes-Côte d'Azur	Other genotypes	Opportunistic		(59,66]	3,024	7.21	6.76	6.75	5.82	7.77	6.76	6.76	6.42	7.10

Table S8 Observed HR HPV cervical infection prevalence, posterior predictive HR HPV cervical infection prevalence, and posterior expected HR HPV cervical infection prevalence, stratified by various dimensions. ***(continued)***

							Posterior distribution							
							Predictive prevalence				Expected prevalence			
									ETI95%				ETI95%	
Region	Genotypes	Screening pathway	Year	Age	Number of tests	Observed	Average	Median	LB	UB	Average	Median	LB	UB
Provence-Alpes-Côte d'Azur	Other genotypes	Organised		(59,66]	177	6.78	7.02	6.78	3.39	11.30	7.07	7.04	6.09	8.26
Île-de-France	Other genotypes	Opportunistic		(59,66]	16,364	6.63	6.54	6.54	6.09	6.98	6.53	6.53	6.29	6.79
Île-de-France	Other genotypes	Organised		(59,66]	1,724	6.09	5.88	5.86	4.58	7.31	5.88	5.86	5.19	6.63
Auvergne-Rhône-Alpes	HPV16/18	Opportunistic	2020	[30,39]			4.14	4.10	2.41	6.27	4.14	4.13	3.73	4.58
Bourgogne-Franche-Comté	HPV16/18	Opportunistic	2020	[30,39]			3.69	3.86	1.45	6.76	3.66	3.66	3.26	4.11
Bretagne	HPV16/18	Opportunistic	2020	[30,39]			3.89	3.93	1.75	6.55	3.88	3.87	3.46	4.33
Centre-Val de Loire	HPV16/18	Opportunistic	2020	[30,39]			3.79	3.77	2.68	4.94	3.79	3.79	3.55	4.05
Corse	HPV16/18	Opportunistic	2020	[30,39]			4.22	4.35	1.09	8.70	4.22	4.20	3.24	5.42
Grand Est	HPV16/18	Opportunistic	2020	[30,39]			3.62	3.40	1.28	6.38	3.58	3.57	3.21	3.96
Hauts-de-France	HPV16/18	Opportunistic	2020	[30,39]			3.58	3.57	2.55	4.75	3.59	3.58	3.34	3.83
Hauts-de-France	HPV16/18	Organised	2020	[30,39]			3.37	0.00	0.00	100.00	3.78	3.70	2.44	5.48
Normandie	HPV16/18	Opportunistic	2020	[30,39]			4.05	4.09	2.81	5.50	4.06	4.06	3.75	4.37
Nouvelle-Aquitaine	HPV16/18	Opportunistic	2020	[30,39]			3.82	3.80	2.38	5.39	3.84	3.84	3.57	4.10
Occitanie	HPV16/18	Opportunistic	2020	[30,39]			4.76	4.68	3.16	6.46	4.77	4.76	4.31	5.26
Pays de la Loire	HPV16/18	Opportunistic	2020	[30,39]			3.20	3.00	1.36	5.18	3.23	3.23	2.89	3.61
Pays de la Loire	HPV16/18	Organised	2020	[30,39]			2.99	0.00	0.00	33.33	3.11	3.06	2.15	4.31
Provence-Alpes-Côte d'Azur	HPV16/18	Opportunistic	2020	[30,39]			3.96	3.91	2.65	5.42	3.96	3.96	3.67	4.28
Île-de-France	HPV16/18	Opportunistic	2020	[30,39]			3.84	3.84	3.55	4.17	3.85	3.85	3.68	4.02
Île-de-France	HPV16/18	Organised	2020	[30,39]			3.70	0.00	0.00	25.00	3.96	3.91	2.90	5.25
Auvergne-Rhône-Alpes	HPV16/18	Opportunistic	2020	(39,49]	262	0.76	3.37	3.44	1.52	5.73	3.38	3.37	3.04	3.74
Bourgogne-Franche-Comté	HPV16/18	Opportunistic	2020	(39,49]	144	2.08	3.02	2.78	0.69	6.25	2.99	2.99	2.64	3.36
Bretagne	HPV16/18	Opportunistic	2020	(39,49]	158	5.06	3.20	3.16	0.63	6.33	3.20	3.19	2.82	3.62
Centre-Val de Loire	HPV16/18	Opportunistic	2020	(39,49]	1,001	2.50	3.12	3.10	2.10	4.30	3.13	3.14	2.92	3.36
Corse	HPV16/18	Opportunistic	2020	(39,49]	118	3.39	3.56	3.39	0.85	7.63	3.50	3.47	2.66	4.54
Grand Est	HPV16/18	Opportunistic	2020	(39,49]	241	2.90	2.90	2.90	0.83	5.39	2.89	2.88	2.57	3.23
Hauts-de-France	HPV16/18	Opportunistic	2020	(39,49]	757	3.83	2.97	2.91	1.85	4.23	2.99	2.99	2.78	3.19
Hauts-de-France	HPV16/18	Organised	2020	(39,49]	2	0.00	2.83	0.00	0.00	50.00	2.89	2.85	2.01	3.99
Normandie	HPV16/18	Opportunistic	2020	(39,49]	675	3.56	3.26	3.26	1.93	4.74	3.25	3.24	2.99	3.50
Nouvelle-Aquitaine	HPV16/18	Opportunistic	2020	(39,49]	424	2.83	3.20	3.07	1.65	4.95	3.20	3.20	2.96	3.43
Occitanie	HPV16/18	Opportunistic	2020	(39,49]	628	3.66	4.05	3.98	2.55	5.73	4.05	4.05	3.64	4.49
Pays de la Loire	HPV16/18	Opportunistic	2020	(39,49]	369	1.90	2.67	2.71	1.08	4.61	2.67	2.66	2.38	2.98
Pays de la Loire	HPV16/18	Organised	2020	(39,49]	1	0.00	1.70	0.00	0.00	0.00	2.49	2.43	1.62	3.67
Provence-Alpes-Côte d'Azur	HPV16/18	Opportunistic	2020	(39,49]	620	3.71	3.19	3.23	1.94	4.68	3.18	3.18	2.92	3.45
Île-de-France	HPV16/18	Opportunistic	2020	(39,49]	16,219	2.78	3.15	3.15	2.85	3.48	3.15	3.16	3.01	3.30
Île-de-France	HPV16/18	Organised	2020	(39,49]	1	0.00	3.57	0.00	0.00	100.00	3.11	3.07	2.21	4.24
Auvergne-Rhône-Alpes	HPV16/18	Opportunistic	2020	(49,59]	215	4.65	2.68	2.79	0.93	5.12	2.74	2.73	2.45	3.04
Bourgogne-Franche-Comté	HPV16/18	Opportunistic	2020	(49,59]	98	1.02	2.55	2.04	0.00	6.12	2.48	2.48	2.22	2.78
Bretagne	HPV16/18	Opportunistic	2020	(49,59]	107	1.87	2.51	2.80	0.00	5.61	2.51	2.50	2.20	2.86
Centre-Val de Loire	HPV16/18	Opportunistic	2020	(49,59]	971	1.65	2.52	2.47	1.54	3.60	2.52	2.52	2.35	2.70

Table S8 Observed HR HPV cervical infection prevalence, posterior predictive HR HPV cervical infection prevalence, and posterior expected HR HPV cervical infection prevalence, stratified by various dimensions. *(continued)*

							Posterior distribution							
							Predictive prevalence				Expected prevalence			
									ETI95%				ETI95%	
Region	Genotypes	Screening pathway	Year	Age	Number of tests	Observed	Average	Median	LB	UB	Average	Median	LB	UB
Centre-Val de Loire	HPV16/18	Organised	2020	(49,59]	2	0.00	2.83	0.00	0.00	50.00	2.76	2.72	1.96	3.76
Corse	HPV16/18	Opportunistic	2020	(49,59]	111	2.70	2.81	2.70	0.00	6.31	2.76	2.74	2.12	3.56
Grand Est	HPV16/18	Opportunistic	2020	(49,59]	279	3.23	2.25	2.15	0.72	4.30	2.29	2.29	2.02	2.58
Hauts-de-France	HPV16/18	Opportunistic	2020	(49,59]	626	2.72	2.34	2.24	1.28	3.67	2.35	2.35	2.18	2.52
Normandie	HPV16/18	Opportunistic	2020	(49,59]	521	3.07	2.58	2.50	1.34	4.03	2.58	2.58	2.37	2.80
Nouvelle-Aquitaine	HPV16/18	Opportunistic	2020	(49,59]	367	3.00	2.52	2.45	1.09	4.09	2.53	2.53	2.34	2.73
Occitanie	HPV16/18	Opportunistic	2020	(49,59]	533	3.00	3.11	3.00	1.69	4.69	3.13	3.12	2.83	3.44
Occitanie	HPV16/18	Organised	2020	(49,59]	3	0.00	3.39	0.00	0.00	33.33	3.24	3.17	2.20	4.63
Pays de la Loire	HPV16/18	Opportunistic	2020	(49,59]	278	0.72	2.17	2.16	0.72	3.96	2.19	2.19	1.96	2.45
Pays de la Loire	HPV16/18	Organised	2020	(49,59]	1	0.00	2.20	0.00	0.00	0.00	2.15	2.10	1.43	3.15
Provence-Alpes-Côte d'Azur	HPV16/18	Opportunistic	2020	(49,59]	509	1.96	2.51	2.55	1.18	3.93	2.52	2.52	2.32	2.74
Île-de-France	HPV16/18	Opportunistic	2020	(49,59]	11,874	2.32	2.52	2.52	2.21	2.84	2.52	2.51	2.40	2.64
Île-de-France	HPV16/18	Organised	2020	(49,59]	3	0.00	3.63	0.00	0.00	33.33	3.40	3.36	2.58	4.40
Auvergne-Rhône-Alpes	HPV16/18	Opportunistic	2020	(59,66]	96	3.12	2.46	2.08	0.00	6.25	2.52	2.52	2.24	2.84
Bourgogne-Franche-Comté	HPV16/18	Opportunistic	2020	(59,66]	88	4.55	2.34	2.27	0.00	5.68	2.31	2.30	2.04	2.60
Bretagne	HPV16/18	Opportunistic	2020	(59,66]	65	1.54	2.24	1.54	0.00	6.15	2.24	2.23	1.92	2.59
Centre-Val de Loire	HPV16/18	Opportunistic	2020	(59,66]	500	1.40	2.33	2.20	1.20	3.80	2.33	2.33	2.15	2.52
Centre-Val de Loire	HPV16/18	Organised	2020	(59,66]	1	100.00	3.73	0.00	0.00	100.00	3.41	3.34	2.19	5.11
Corse	HPV16/18	Opportunistic	2020	(59,66]	56	1.79	2.63	1.79	0.00	7.14	2.60	2.57	2.03	3.31
Grand Est	HPV16/18	Opportunistic	2020	(59,66]	127	3.15	2.11	1.97	0.00	4.72	2.10	2.10	1.84	2.40
Hauts-de-France	HPV16/18	Opportunistic	2020	(59,66]	272	2.21	2.14	2.21	0.74	4.04	2.16	2.16	1.98	2.35
Hauts-de-France	HPV16/18	Organised	2020	(59,66]	4	25.00	2.61	0.00	0.00	25.00	2.58	2.54	1.89	3.46
Normandie	HPV16/18	Opportunistic	2020	(59,66]	262	3.82	2.34	2.29	0.76	4.20	2.35	2.34	2.13	2.58
Nouvelle-Aquitaine	HPV16/18	Opportunistic	2020	(59,66]	266	2.26	2.41	2.26	0.75	4.51	2.40	2.39	2.18	2.62
Nouvelle-Aquitaine	HPV16/18	Organised	2020	(59,66]	1	0.00	2.33	0.00	0.00	0.00	2.52	2.43	1.46	4.06
Occitanie	HPV16/18	Opportunistic	2020	(59,66]	287	4.53	2.92	2.79	1.05	4.89	2.93	2.92	2.63	3.25
Occitanie	HPV16/18	Organised	2020	(59,66]	1	0.00	3.23	0.00	0.00	100.00	3.05	2.98	2.01	4.39
Pays de la Loire	HPV16/18	Opportunistic	2020	(59,66]	142	0.70	2.15	2.11	0.00	4.93	2.15	2.15	1.91	2.43
Provence-Alpes-Côte d'Azur	HPV16/18	Opportunistic	2020	(59,66]	207	0.97	2.38	2.42	0.48	4.83	2.40	2.39	2.18	2.62
Provence-Alpes-Côte d'Azur	HPV16/18	Organised	2020	(59,66]	2	50.00	2.80	0.00	0.00	50.00	2.71	2.66	1.88	3.84
Île-de-France	HPV16/18	Opportunistic	2020	(59,66]	4,705	2.13	2.33	2.34	1.89	2.81	2.33	2.33	2.18	2.48
Île-de-France	HPV16/18	Organised	2020	(59,66]	4	0.00	2.47	0.00	0.00	25.00	2.50	2.47	1.87	3.32
Auvergne-Rhône-Alpes	HPV16/18	Opportunistic	2021	[30,39]			4.90	4.94	3.68	6.19	4.89	4.88	4.47	5.33
Auvergne-Rhône-Alpes	HPV16/18	Organised	2021	[30,39]			4.58	2.86	0.00	11.43	4.46	4.43	3.59	5.52
Bourgogne-Franche-Comté	HPV16/18	Opportunistic	2021	[30,39]			4.32	4.29	2.74	6.00	4.32	4.31	3.89	4.79
Bourgogne-Franche-Comté	HPV16/18	Organised	2021	[30,39]			3.82	0.00	0.00	33.33	3.94	3.89	2.95	5.21
Bretagne	HPV16/18	Opportunistic	2021	[30,39]			4.49	4.45	3.08	5.93	4.48	4.47	4.05	4.93
Bretagne	HPV16/18	Organised	2021	[30,39]			3.00	2.44	0.00	9.76	3.01	2.98	2.29	3.86
Centre-Val de Loire	HPV16/18	Opportunistic	2021	[30,39]			4.39	4.38	3.75	5.04	4.40	4.40	4.17	4.63

Table S8 **Observed HR HPV cervical infection prevalence, posterior predictive HR HPV cervical infection prevalence, and posterior expected HR HPV cervical infection prevalence, stratified by various dimensions.** ***(continued)***

							Posterior distribution							
							Predictive prevalence				Expected prevalence			
									ETI95%				ETI95%	
Region	Genotypes	Screening pathway	Year	Age	Number of tests	Observed	Average	Median	LB	UB	Average	Median	LB	UB
Centre-Val de Loire	HPV16/18	Organised	2021	[30,39]			3.68	3.70	1.39	6.48	3.66	3.64	3.03	4.36
Corse	HPV16/18	Opportunistic	2021	[30,39]			4.70	0.00	0.00	15.38	4.73	4.70	3.85	5.80
Grand Est	HPV16/18	Opportunistic	2021	[30,39]			4.20	4.16	3.01	5.40	4.18	4.18	3.88	4.51
Grand Est	HPV16/18	Organised	2021	[30,39]			3.63	0.00	0.00	20.00	3.37	3.33	2.56	4.36
Hauts-de-France	HPV16/18	Opportunistic	2021	[30,39]			4.19	4.18	3.58	4.85	4.19	4.18	3.96	4.41
Hauts-de-France	HPV16/18	Organised	2021	[30,39]			3.58	3.70	0.74	7.41	3.62	3.61	3.00	4.34
Normandie	HPV16/18	Opportunistic	2021	[30,39]			4.82	4.80	3.99	5.68	4.81	4.81	4.50	5.12
Normandie	HPV16/18	Organised	2021	[30,39]			4.03	0.00	0.00	16.67	3.84	3.81	2.96	4.88
Nouvelle-Aquitaine	HPV16/18	Opportunistic	2021	[30,39]			4.42	4.41	3.66	5.22	4.41	4.41	4.16	4.67
Nouvelle-Aquitaine	HPV16/18	Organised	2021	[30,39]			3.55	3.03	0.00	12.12	3.62	3.60	2.87	4.55
Occitanie	HPV16/18	Opportunistic	2021	[30,39]			5.02	4.99	3.99	6.15	5.02	5.02	4.68	5.38
Occitanie	HPV16/18	Organised	2021	[30,39]			4.64	3.85	0.00	11.54	4.58	4.56	3.77	5.53
Pays de la Loire	HPV16/18	Opportunistic	2021	[30,39]			3.76	3.69	2.53	5.06	3.73	3.73	3.37	4.11
Pays de la Loire	HPV16/18	Organised	2021	[30,39]			3.15	3.64	0.00	9.09	3.14	3.12	2.48	3.94
Provence-Alpes-Côte d'Azur	HPV16/18	Opportunistic	2021	[30,39]			4.75	4.74	4.08	5.48	4.74	4.74	4.48	5.02
Provence-Alpes-Côte d'Azur	HPV16/18	Organised	2021	[30,39]			4.35	5.00	0.00	15.00	4.41	4.39	3.53	5.49
Île-de-France	HPV16/18	Opportunistic	2021	[30,39]			4.42	4.42	4.14	4.71	4.42	4.43	4.27	4.57
Île-de-France	HPV16/18	Organised	2021	[30,39]			3.93	3.77	1.89	6.20	3.90	3.88	3.16	4.76
Auvergne-Rhône-Alpes	HPV16/18	Opportunistic	2021	(39,49]	804	3.98	4.00	3.98	2.61	5.35	4.00	4.00	3.65	4.38
Auvergne-Rhône-Alpes	HPV16/18	Organised	2021	(39,49]	48	0.00	3.69	4.17	0.00	10.42	3.66	3.64	2.96	4.45
Bourgogne-Franche-Comté	HPV16/18	Opportunistic	2021	(39,49]	391	3.07	3.58	3.58	1.79	5.63	3.58	3.57	3.22	3.97
Bourgogne-Franche-Comté	HPV16/18	Organised	2021	(39,49]	1	0.00	2.33	0.00	0.00	0.00	2.94	2.89	2.06	4.06
Bretagne	HPV16/18	Opportunistic	2021	(39,49]	588	3.06	3.61	3.57	2.21	5.27	3.62	3.62	3.29	4.02
Bretagne	HPV16/18	Organised	2021	(39,49]	16	12.50	2.35	0.00	0.00	12.50	2.40	2.37	1.84	3.06
Centre-Val de Loire	HPV16/18	Opportunistic	2021	(39,49]	4,201	3.19	3.60	3.59	3.02	4.19	3.59	3.59	3.39	3.80
Centre-Val de Loire	HPV16/18	Organised	2021	(39,49]	138	1.45	3.01	2.90	0.72	6.52	3.05	3.03	2.56	3.61
Corse	HPV16/18	Opportunistic	2021	(39,49]	5	20.00	3.87	0.00	0.00	20.00	3.96	3.95	3.14	4.93
Grand Est	HPV16/18	Opportunistic	2021	(39,49]	1,093	3.39	3.45	3.48	2.38	4.57	3.46	3.46	3.19	3.74
Grand Est	HPV16/18	Organised	2021	(39,49]	26	0.00	2.79	3.85	0.00	11.54	2.70	2.69	2.21	3.24
Hauts-de-France	HPV16/18	Opportunistic	2021	(39,49]	2,916	3.88	3.49	3.46	2.81	4.15	3.48	3.48	3.29	3.68
Hauts-de-France	HPV16/18	Organised	2021	(39,49]	110	1.82	2.99	2.73	0.00	6.36	2.99	2.98	2.48	3.55
Normandie	HPV16/18	Opportunistic	2021	(39,49]	2,310	4.94	3.92	3.90	3.12	4.81	3.92	3.92	3.66	4.18
Normandie	HPV16/18	Organised	2021	(39,49]	5	0.00	3.55	0.00	0.00	20.00	3.40	3.38	2.63	4.31
Nouvelle-Aquitaine	HPV16/18	Opportunistic	2021	(39,49]	2,359	3.65	3.59	3.60	2.88	4.37	3.60	3.60	3.38	3.82
Nouvelle-Aquitaine	HPV16/18	Organised	2021	(39,49]	3	0.00	3.20	0.00	0.00	33.33	3.20	3.17	2.43	4.10
Occitanie	HPV16/18	Opportunistic	2021	(39,49]	1,638	4.15	4.12	4.09	3.11	5.19	4.13	4.13	3.85	4.43
Occitanie	HPV16/18	Organised	2021	(39,49]	110	3.64	3.68	3.64	0.91	7.27	3.70	3.69	3.02	4.45
Pays de la Loire	HPV16/18	Opportunistic	2021	(39,49]	834	2.76	3.13	3.12	1.92	4.44	3.14	3.13	2.85	3.44
Pays de la Loire	HPV16/18	Organised	2021	(39,49]	85	1.18	2.59	2.35	0.00	5.88	2.68	2.67	2.15	3.28

Table S8 Observed HR HPV cervical infection prevalence, posterior predictive HR HPV cervical infection prevalence, and posterior expected HR HPV cervical infection prevalence, stratified by various dimensions. ***(continued)***

							Posterior distribution							
							Predictive prevalence				Expected prevalence			
									ETI95%				ETI95%	
Region	Genotypes	Screening pathway	Year	Age	Number of tests	Observed	Average	Median	LB	UB	Average	Median	LB	UB
Provence-Alpes-Côte d'Azur	HPV16/18	Opportunistic	2021	(39,49]	3,170	3.72	3.86	3.85	3.12	4.57	3.85	3.85	3.62	4.10
Provence-Alpes-Côte d'Azur	HPV16/18	Organised	2021	(39,49]	7	0.00	3.79	0.00	0.00	28.57	3.73	3.70	3.00	4.56
Île-de-France	HPV16/18	Opportunistic	2021	(39,49]	25,340	3.44	3.62	3.62	3.36	3.88	3.62	3.62	3.48	3.76
Île-de-France	HPV16/18	Organised	2021	(39,49]	83	1.20	3.19	2.41	0.00	7.23	3.18	3.17	2.69	3.73
Auvergne-Rhône-Alpes	HPV16/18	Opportunistic	2021	(49,59]	666	2.25	3.18	3.15	1.95	4.65	3.20	3.20	2.91	3.52
Auvergne-Rhône-Alpes	HPV16/18	Organised	2021	(49,59]	38	0.00	2.96	2.63	0.00	7.89	3.03	3.02	2.46	3.71
Bourgogne-Franche-Comté	HPV16/18	Opportunistic	2021	(49,59]	329	3.34	2.90	2.74	1.22	4.86	2.90	2.90	2.62	3.23
Bourgogne-Franche-Comté	HPV16/18	Organised	2021	(49,59]	1	0.00	2.77	0.00	0.00	100.00	2.46	2.42	1.73	3.35
Bretagne	HPV16/18	Opportunistic	2021	(49,59]	556	2.16	2.85	2.88	1.44	4.32	2.83	2.83	2.55	3.14
Bretagne	HPV16/18	Organised	2021	(49,59]	36	0.00	1.99	2.78	0.00	8.33	2.04	2.03	1.59	2.61
Centre-Val de Loire	HPV16/18	Opportunistic	2021	(49,59]	3,844	2.21	2.86	2.86	2.32	3.43	2.86	2.86	2.70	3.04
Centre-Val de Loire	HPV16/18	Organised	2021	(49,59]	224	2.23	2.48	2.23	0.45	4.91	2.48	2.47	2.11	2.92
Corse	HPV16/18	Opportunistic	2021	(49,59]	9	0.00	2.96	0.00	0.00	11.11	2.97	2.95	2.53	3.44
Corse	HPV16/18	Organised	2021	(49,59]	1	0.00	2.37	0.00	0.00	0.00	2.84	2.77	1.84	4.21
Grand Est	HPV16/18	Opportunistic	2021	(49,59]	1,003	2.79	2.82	2.79	1.79	3.89	2.79	2.79	2.57	3.02
Grand Est	HPV16/18	Organised	2021	(49,59]	59	0.00	2.27	1.69	0.00	6.78	2.34	2.33	1.91	2.85
Hauts-de-France	HPV16/18	Opportunistic	2021	(49,59]	2,015	3.18	2.77	2.78	2.08	3.52	2.78	2.78	2.61	2.95
Hauts-de-France	HPV16/18	Organised	2021	(49,59]	96	3.12	2.40	2.08	0.00	6.25	2.44	2.43	2.07	2.85
Normandie	HPV16/18	Opportunistic	2021	(49,59]	1,829	2.90	3.09	3.06	2.30	3.94	3.09	3.09	2.87	3.31
Normandie	HPV16/18	Organised	2021	(49,59]	5	0.00	2.74	0.00	0.00	20.00	2.63	2.62	2.03	3.34
Nouvelle-Aquitaine	HPV16/18	Opportunistic	2021	(49,59]	1,995	2.86	2.89	2.86	2.16	3.66	2.88	2.88	2.71	3.07
Nouvelle-Aquitaine	HPV16/18	Organised	2021	(49,59]	170	2.94	2.53	2.35	0.59	5.29	2.54	2.53	2.16	2.97
Occitanie	HPV16/18	Opportunistic	2021	(49,59]	1,372	3.94	3.34	3.35	2.41	4.37	3.35	3.35	3.10	3.61
Occitanie	HPV16/18	Organised	2021	(49,59]	95	3.16	3.35	3.16	0.00	7.37	3.35	3.33	2.73	4.05
Pays de la Loire	HPV16/18	Opportunistic	2021	(49,59]	703	3.13	2.52	2.56	1.42	3.84	2.52	2.51	2.27	2.77
Pays de la Loire	HPV16/18	Organised	2021	(49,59]	78	2.56	2.33	2.56	0.00	6.41	2.29	2.27	1.86	2.79
Provence-Alpes-Côte d'Azur	HPV16/18	Opportunistic	2021	(49,59]	2,577	2.91	3.05	3.03	2.37	3.76	3.06	3.05	2.86	3.27
Provence-Alpes-Côte d'Azur	HPV16/18	Organised	2021	(49,59]	7	0.00	3.00	0.00	0.00	14.29	2.98	2.95	2.35	3.75
Île-de-France	HPV16/18	Opportunistic	2021	(49,59]	18,998	2.84	2.89	2.89	2.63	3.16	2.89	2.89	2.76	3.01
Île-de-France	HPV16/18	Organised	2021	(49,59]	161	3.73	2.51	2.48	0.62	4.97	2.53	2.52	2.15	2.96
Auvergne-Rhône-Alpes	HPV16/18	Opportunistic	2021	(59,66]	330	0.91	2.95	3.03	1.21	4.85	2.95	2.94	2.66	3.25
Auvergne-Rhône-Alpes	HPV16/18	Organised	2021	(59,66]	31	0.00	2.96	3.23	0.00	9.68	2.92	2.90	2.34	3.59
Bourgogne-Franche-Comté	HPV16/18	Opportunistic	2021	(59,66]	207	4.83	2.69	2.42	0.97	5.31	2.71	2.70	2.42	3.01
Bourgogne-Franche-Comté	HPV16/18	Organised	2021	(59,66]	22	4.55	2.42	0.00	0.00	9.09	2.46	2.45	1.96	3.06
Bretagne	HPV16/18	Opportunistic	2021	(59,66]	239	1.67	2.63	2.51	0.84	5.02	2.65	2.64	2.36	2.96
Bretagne	HPV16/18	Organised	2021	(59,66]	21	9.52	2.29	0.00	0.00	9.52	2.17	2.14	1.64	2.85
Centre-Val de Loire	HPV16/18	Opportunistic	2021	(59,66]	2,018	2.63	2.65	2.63	1.93	3.42	2.66	2.65	2.47	2.86
Centre-Val de Loire	HPV16/18	Organised	2021	(59,66]	143	2.10	2.35	2.10	0.00	4.90	2.38	2.36	1.98	2.82
Corse	HPV16/18	Opportunistic	2021	(59,66]	7	0.00	3.00	0.00	0.00	14.29	3.03	3.01	2.44	3.74

Table S8 Observed HR HPV cervical infection prevalence, posterior predictive HR HPV cervical infection prevalence, and posterior expected HR HPV cervical infection prevalence, stratified by various dimensions. ***(continued)***

							Posterior distribution							
							Predictive prevalence				Expected prevalence			
									ETI95%				ETI95%	
Region	Genotypes	Screening pathway	Year	Age	Number of tests	Observed	Average	Median	LB	UB	Average	Median	LB	UB
Grand Est	HPV16/18	Opportunistic	2021	(59,66]	562	2.14	2.60	2.49	1.25	3.91	2.59	2.59	2.37	2.83
Grand Est	HPV16/18	Organised	2021	(59,66]	46	2.17	2.24	2.17	0.00	6.52	2.29	2.27	1.82	2.85
Hauts-de-France	HPV16/18	Opportunistic	2021	(59,66]	931	2.26	2.56	2.58	1.61	3.65	2.56	2.56	2.37	2.76
Hauts-de-France	HPV16/18	Organised	2021	(59,66]	49	2.04	2.43	2.04	0.00	8.16	2.49	2.47	2.03	3.03
Normandie	HPV16/18	Opportunistic	2021	(59,66]	950	2.63	2.86	2.84	1.89	4.00	2.86	2.85	2.62	3.11
Normandie	HPV16/18	Organised	2021	(59,66]	18	11.11	2.63	0.00	0.00	11.11	2.51	2.50	2.04	3.08
Nouvelle-Aquitaine	HPV16/18	Opportunistic	2021	(59,66]	1,222	2.70	2.70	2.70	1.80	3.68	2.71	2.71	2.51	2.92
Nouvelle-Aquitaine	HPV16/18	Organised	2021	(59,66]	185	4.32	2.61	2.70	0.54	5.41	2.58	2.57	2.14	3.06
Occitanie	HPV16/18	Opportunistic	2021	(59,66]	720	3.06	3.09	3.06	1.94	4.44	3.10	3.09	2.85	3.36
Occitanie	HPV16/18	Organised	2021	(59,66]	48	0.00	3.21	2.08	0.00	8.33	3.22	3.20	2.59	3.93
Pays de la Loire	HPV16/18	Opportunistic	2021	(59,66]	326	3.07	2.47	2.45	0.92	4.29	2.46	2.46	2.20	2.74
Pays de la Loire	HPV16/18	Organised	2021	(59,66]	52	0.00	2.28	1.92	0.00	7.69	2.27	2.25	1.80	2.83
Provence-Alpes-Côte d'Azur	HPV16/18	Opportunistic	2021	(59,66]	1,314	2.82	2.83	2.82	1.98	3.73	2.83	2.83	2.62	3.06
Provence-Alpes-Côte d'Azur	HPV16/18	Organised	2021	(59,66]	22	4.55	2.78	0.00	0.00	9.09	2.78	2.75	2.19	3.52
Île-de-France	HPV16/18	Opportunistic	2021	(59,66]	8,881	2.80	2.69	2.69	2.32	3.07	2.69	2.69	2.53	2.86
Île-de-France	HPV16/18	Organised	2021	(59,66]	1,384	2.38	2.61	2.60	1.66	3.61	2.61	2.59	2.12	3.17
Auvergne-Rhône-Alpes	HPV16/18	Opportunistic	2022	[30,39]			5.58	5.61	1.87	10.28	5.62	5.60	5.03	6.25
Auvergne-Rhône-Alpes	HPV16/18	Organised	2022	[30,39]			4.14	0.00	0.00	16.67	4.24	4.22	3.45	5.19
Bourgogne-Franche-Comté	HPV16/18	Opportunistic	2022	[30,39]			4.69	4.55	2.90	6.63	4.68	4.67	4.19	5.22
Bourgogne-Franche-Comté	HPV16/18	Organised	2022	[30,39]			3.35	3.85	0.00	11.54	3.48	3.46	2.78	4.31
Bretagne	HPV16/18	Opportunistic	2022	[30,39]			5.07	5.05	3.77	6.54	5.08	5.08	4.64	5.58
Bretagne	HPV16/18	Organised	2022	[30,39]			3.12	3.18	0.64	6.37	3.15	3.13	2.52	3.91
Centre-Val de Loire	HPV16/18	Opportunistic	2022	[30,39]			4.65	4.63	3.74	5.59	4.65	4.64	4.38	4.94
Centre-Val de Loire	HPV16/18	Organised	2022	[30,39]			3.38	3.43	0.98	5.88	3.41	3.40	2.91	3.95
Corse	HPV16/18	Opportunistic	2022	[30,39]			5.11	0.00	0.00	25.00	5.19	5.15	4.27	6.30
Grand Est	HPV16/18	Opportunistic	2022	[30,39]			4.56	4.54	3.32	5.85	4.57	4.56	4.20	4.95
Grand Est	HPV16/18	Organised	2022	[30,39]			3.30	3.15	0.90	5.86	3.30	3.29	2.77	3.92
Hauts-de-France	HPV16/18	Opportunistic	2022	[30,39]			4.52	4.50	3.83	5.24	4.52	4.52	4.26	4.78
Hauts-de-France	HPV16/18	Organised	2022	[30,39]			3.40	3.35	1.68	5.59	3.41	3.40	2.90	3.98
Normandie	HPV16/18	Opportunistic	2022	[30,39]			5.38	5.38	4.40	6.41	5.37	5.37	5.03	5.74
Normandie	HPV16/18	Organised	2022	[30,39]			3.43	4.55	0.00	13.64	3.54	3.52	2.91	4.29
Nouvelle-Aquitaine	HPV16/18	Opportunistic	2022	[30,39]			4.82	4.80	4.04	5.59	4.82	4.81	4.52	5.10
Nouvelle-Aquitaine	HPV16/18	Organised	2022	[30,39]			3.65	3.53	1.60	5.77	3.66	3.65	3.12	4.26
Occitanie	HPV16/18	Opportunistic	2022	[30,39]			5.26	5.25	3.79	6.96	5.24	5.24	4.86	5.66
Occitanie	HPV16/18	Organised	2022	[30,39]			4.04	4.23	1.41	7.75	4.07	4.06	3.42	4.78
Pays de la Loire	HPV16/18	Opportunistic	2022	[30,39]			4.43	4.31	1.96	7.06	4.39	4.38	3.98	4.83
Pays de la Loire	HPV16/18	Organised	2022	[30,39]			2.98	3.17	0.00	7.94	3.00	2.99	2.50	3.59
Provence-Alpes-Côte d'Azur	HPV16/18	Opportunistic	2022	[30,39]			5.18	5.18	4.36	6.06	5.17	5.17	4.86	5.49
Provence-Alpes-Côte d'Azur	HPV16/18	Organised	2022	[30,39]			4.44	4.15	2.07	7.47	4.44	4.41	3.69	5.33

Table S8 Observed HR HPV cervical infection prevalence, posterior predictive HR HPV cervical infection prevalence, and posterior expected HR HPV cervical infection prevalence, stratified by various dimensions. ***(continued)***

							Posterior distribution							
							Predictive prevalence				Expected prevalence			
									ETI95%				ETI95%	
Region	Genotypes	Screening pathway	Year	Age	Number of tests	Observed	Average	Median	LB	UB	Average	Median	LB	UB
Île-de-France	HPV16/18	Opportunistic	2022	[30,39]			4.94	4.93	4.37	5.54	4.94	4.94	4.70	5.18
Île-de-France	HPV16/18	Organised	2022	[30,39]			3.79	3.72	1.86	6.19	3.79	3.77	3.24	4.40
Auvergne-Rhône-Alpes	HPV16/18	Opportunistic	2022	(39,49]	64	9.38	4.78	4.69	0.00	10.94	4.78	4.77	4.16	5.51
Auvergne-Rhône-Alpes	HPV16/18	Organised	2022	(39,49]	15	20.00	3.48	0.00	0.00	13.33	3.59	3.56	2.93	4.37
Bourgogne-Franche-Comté	HPV16/18	Opportunistic	2022	(39,49]	333	3.60	3.83	3.90	1.80	6.01	3.82	3.81	3.40	4.26
Bourgogne-Franche-Comté	HPV16/18	Organised	2022	(39,49]	1	0.00	3.07	0.00	0.00	100.00	2.85	2.81	2.02	3.94
Bretagne	HPV16/18	Opportunistic	2022	(39,49]	663	4.37	4.16	4.07	2.71	5.88	4.16	4.15	3.79	4.56
Bretagne	HPV16/18	Organised	2022	(39,49]	173	4.62	2.56	2.31	0.58	5.20	2.55	2.54	2.09	3.11
Centre-Val de Loire	HPV16/18	Opportunistic	2022	(39,49]	2,079	4.95	3.80	3.80	2.98	4.67	3.82	3.81	3.58	4.05
Centre-Val de Loire	HPV16/18	Organised	2022	(39,49]	248	4.03	2.82	2.82	0.81	5.24	2.83	2.82	2.45	3.22
Corse	HPV16/18	Opportunistic	2022	(39,49]	3	0.00	5.26	0.00	0.00	33.33	4.76	4.72	3.68	6.06
Corse	HPV16/18	Organised	2022	(39,49]	2	0.00	3.17	0.00	0.00	50.00	3.15	3.11	2.28	4.23
Grand Est	HPV16/18	Opportunistic	2022	(39,49]	858	4.55	3.84	3.85	2.56	5.24	3.84	3.83	3.52	4.19
Grand Est	HPV16/18	Organised	2022	(39,49]	198	3.54	2.78	2.53	0.51	5.56	2.74	2.73	2.29	3.20
Hauts-de-France	HPV16/18	Opportunistic	2022	(39,49]	2,382	4.49	3.76	3.78	2.98	4.58	3.77	3.77	3.54	4.00
Hauts-de-France	HPV16/18	Organised	2022	(39,49]	286	1.75	2.80	2.80	1.05	4.90	2.81	2.80	2.42	3.23
Normandie	HPV16/18	Opportunistic	2022	(39,49]	1,814	3.80	4.40	4.41	3.42	5.40	4.39	4.39	4.10	4.69
Normandie	HPV16/18	Organised	2022	(39,49]	22	4.55	3.04	4.55	0.00	13.64	3.05	3.05	2.59	3.56
Nouvelle-Aquitaine	HPV16/18	Opportunistic	2022	(39,49]	2,812	4.30	3.97	3.98	3.24	4.77	3.97	3.97	3.72	4.22
Nouvelle-Aquitaine	HPV16/18	Organised	2022	(39,49]	337	2.97	2.99	2.97	1.19	5.04	3.00	2.99	2.61	3.41
Occitanie	HPV16/18	Opportunistic	2022	(39,49]	746	3.62	4.30	4.29	2.82	5.76	4.31	4.30	3.98	4.66
Occitanie	HPV16/18	Organised	2022	(39,49]	135	2.22	3.32	2.96	0.74	6.67	3.34	3.33	2.83	3.91
Pays de la Loire	HPV16/18	Opportunistic	2022	(39,49]	203	1.48	3.68	3.45	1.48	6.40	3.66	3.65	3.31	4.04
Pays de la Loire	HPV16/18	Organised	2022	(39,49]	71	4.23	2.62	2.82	0.00	7.04	2.55	2.55	2.15	3.00
Provence-Alpes-Côte d'Azur	HPV16/18	Opportunistic	2022	(39,49]	2,056	4.23	4.21	4.18	3.36	5.16	4.22	4.22	3.96	4.50
Provence-Alpes-Côte d'Azur	HPV16/18	Organised	2022	(39,49]	112	3.57	3.72	3.57	0.89	7.14	3.74	3.72	3.13	4.44
Île-de-France	HPV16/18	Opportunistic	2022	(39,49]	4,399	3.71	3.98	3.98	3.39	4.59	3.97	3.97	3.77	4.18
Île-de-France	HPV16/18	Organised	2022	(39,49]	342	3.22	3.12	2.92	1.46	5.26	3.12	3.12	2.70	3.58
Auvergne-Rhône-Alpes	HPV16/18	Opportunistic	2022	(49,59]	49	6.12	4.10	4.08	0.00	10.20	3.97	3.95	3.43	4.59
Auvergne-Rhône-Alpes	HPV16/18	Organised	2022	(49,59]	13	7.69	3.12	0.00	0.00	15.38	3.10	3.07	2.41	3.90
Bourgogne-Franche-Comté	HPV16/18	Opportunistic	2022	(49,59]	239	2.51	3.11	2.93	1.26	5.44	3.10	3.09	2.78	3.47
Bourgogne-Franche-Comté	HPV16/18	Organised	2022	(49,59]	1	0.00	2.57	0.00	0.00	100.00	2.47	2.43	1.71	3.46
Bretagne	HPV16/18	Opportunistic	2022	(49,59]	535	2.62	3.32	3.36	1.87	4.86	3.31	3.31	3.00	3.65
Bretagne	HPV16/18	Organised	2022	(49,59]	144	1.39	2.11	2.08	0.00	4.86	2.11	2.10	1.71	2.56
Centre-Val de Loire	HPV16/18	Opportunistic	2022	(49,59]	1,838	3.54	3.04	3.05	2.29	3.86	3.05	3.05	2.86	3.27
Centre-Val de Loire	HPV16/18	Organised	2022	(49,59]	278	3.60	2.40	2.16	0.72	4.32	2.40	2.40	2.08	2.76
Corse	HPV16/18	Opportunistic	2022	(49,59]	2	0.00	3.60	0.00	0.00	50.00	3.54	3.49	2.51	4.83
Grand Est	HPV16/18	Opportunistic	2022	(49,59]	767	2.35	3.13	3.13	1.96	4.43	3.12	3.11	2.84	3.40
Grand Est	HPV16/18	Organised	2022	(49,59]	120	1.67	2.36	2.50	0.00	5.00	2.38	2.37	2.00	2.79

Table S8 **Observed HR HPV cervical infection prevalence, posterior predictive HR HPV cervical infection prevalence, and posterior expected HR HPV cervical infection prevalence, stratified by various dimensions.** ***(continued)***

							Posterior distribution							
							Predictive prevalence				Expected prevalence			
									ETI95%				ETI95%	
Region	Genotypes	Screening pathway	Year	Age	Number of tests	Observed	Average	Median	LB	UB	Average	Median	LB	UB
Hauts-de-France	HPV16/18	Opportunistic	2022	(49,59]	1,725	3.01	3.02	3.01	2.20	3.88	3.02	3.02	2.83	3.22
Hauts-de-France	HPV16/18	Organised	2022	(49,59]	267	3.00	2.35	2.25	0.75	4.12	2.34	2.34	2.02	2.70
Normandie	HPV16/18	Opportunistic	2022	(49,59]	1,196	4.26	3.42	3.43	2.42	4.52	3.43	3.43	3.20	3.68
Normandie	HPV16/18	Organised	2022	(49,59]	16	0.00	2.55	0.00	0.00	12.50	2.48	2.47	2.08	2.94
Nouvelle-Aquitaine	HPV16/18	Opportunistic	2022	(49,59]	2,090	3.11	3.18	3.16	2.44	3.97	3.18	3.18	2.99	3.39
Nouvelle-Aquitaine	HPV16/18	Organised	2022	(49,59]	644	2.17	2.52	2.48	1.40	3.88	2.51	2.51	2.18	2.87
Occitanie	HPV16/18	Opportunistic	2022	(49,59]	710	3.38	3.47	3.38	2.11	4.93	3.47	3.47	3.19	3.75
Occitanie	HPV16/18	Organised	2022	(49,59]	141	0.71	2.86	2.84	0.71	5.67	2.86	2.85	2.43	3.35
Pays de la Loire	HPV16/18	Opportunistic	2022	(49,59]	158	1.90	3.02	3.16	0.63	5.70	3.01	3.01	2.71	3.33
Pays de la Loire	HPV16/18	Organised	2022	(49,59]	67	0.00	2.12	1.49	0.00	5.97	2.15	2.15	1.82	2.52
Provence-Alpes-Côte d'Azur	HPV16/18	Opportunistic	2022	(49,59]	1,791	3.13	3.33	3.29	2.46	4.24	3.33	3.33	3.11	3.56
Provence-Alpes-Côte d'Azur	HPV16/18	Organised	2022	(49,59]	85	3.53	3.14	2.35	0.00	7.06	3.11	3.10	2.59	3.73
Île-de-France	HPV16/18	Opportunistic	2022	(49,59]	3,353	2.74	3.14	3.13	2.56	3.76	3.13	3.13	2.96	3.31
Île-de-France	HPV16/18	Organised	2022	(49,59]	449	2.00	2.53	2.45	1.11	4.23	2.53	2.53	2.20	2.93
Auvergne-Rhône-Alpes	HPV16/18	Opportunistic	2022	(59,66]	27	3.70	3.50	3.70	0.00	11.11	3.43	3.42	3.00	3.91
Auvergne-Rhône-Alpes	HPV16/18	Organised	2022	(59,66]	8	0.00	3.25	0.00	0.00	25.00	3.13	3.11	2.37	4.08
Bourgogne-Franche-Comté	HPV16/18	Opportunistic	2022	(59,66]	120	1.67	2.83	2.50	0.00	5.83	2.85	2.84	2.52	3.21
Bourgogne-Franche-Comté	HPV16/18	Organised	2022	(59,66]	17	5.88	2.33	0.00	0.00	11.76	2.39	2.37	1.89	2.99
Bretagne	HPV16/18	Opportunistic	2022	(59,66]	289	4.15	3.05	3.11	1.38	5.19	3.04	3.04	2.73	3.38
Bretagne	HPV16/18	Organised	2022	(59,66]	112	0.00	2.00	1.79	0.00	5.36	2.01	2.00	1.59	2.51
Centre-Val de Loire	HPV16/18	Opportunistic	2022	(59,66]	872	3.21	2.81	2.75	1.72	4.01	2.81	2.81	2.59	3.04
Centre-Val de Loire	HPV16/18	Organised	2022	(59,66]	188	3.19	2.25	2.13	0.53	4.79	2.28	2.27	1.91	2.70
Corse	HPV16/18	Opportunistic	2022	(59,66]	2	0.00	3.98	0.00	0.00	50.00	3.96	3.89	2.74	5.63
Grand Est	HPV16/18	Opportunistic	2022	(59,66]	312	2.56	2.89	2.88	1.28	4.81	2.87	2.87	2.61	3.16
Grand Est	HPV16/18	Organised	2022	(59,66]	82	2.44	2.32	2.44	0.00	6.10	2.32	2.31	1.89	2.81
Hauts-de-France	HPV16/18	Opportunistic	2022	(59,66]	714	2.38	2.81	2.80	1.68	4.06	2.82	2.82	2.61	3.04
Hauts-de-France	HPV16/18	Organised	2022	(59,66]	169	2.96	2.27	2.37	0.59	4.73	2.29	2.28	1.90	2.72
Normandie	HPV16/18	Opportunistic	2022	(59,66]	612	3.10	3.19	3.10	1.80	4.58	3.17	3.16	2.91	3.44
Normandie	HPV16/18	Organised	2022	(59,66]	93	1.08	2.43	2.15	0.00	6.45	2.44	2.42	2.00	2.96
Nouvelle-Aquitaine	HPV16/18	Opportunistic	2022	(59,66]	1,040	3.37	2.95	2.88	2.02	4.04	2.95	2.95	2.73	3.18
Nouvelle-Aquitaine	HPV16/18	Organised	2022	(59,66]	440	1.82	2.42	2.27	1.14	4.09	2.43	2.43	2.04	2.88
Occitanie	HPV16/18	Opportunistic	2022	(59,66]	349	3.72	3.22	3.15	1.43	5.16	3.21	3.21	2.92	3.51
Occitanie	HPV16/18	Organised	2022	(59,66]	89	2.25	2.69	2.25	0.00	6.74	2.72	2.70	2.26	3.26
Pays de la Loire	HPV16/18	Opportunistic	2022	(59,66]	105	2.86	2.94	2.86	0.00	6.67	2.89	2.88	2.55	3.26
Pays de la Loire	HPV16/18	Organised	2022	(59,66]	40	0.00	2.22	2.50	0.00	7.50	2.15	2.14	1.75	2.62
Provence-Alpes-Côte d'Azur	HPV16/18	Opportunistic	2022	(59,66]	836	2.99	3.07	2.99	1.91	4.31	3.08	3.07	2.83	3.33
Provence-Alpes-Côte d'Azur	HPV16/18	Organised	2022	(59,66]	66	4.55	2.88	3.03	0.00	7.58	2.88	2.87	2.35	3.55
Île-de-France	HPV16/18	Opportunistic	2022	(59,66]	1,520	2.50	2.91	2.89	2.11	3.82	2.91	2.90	2.71	3.12
Île-de-France	HPV16/18	Organised	2022	(59,66]	193	3.11	2.41	2.07	0.52	4.66	2.43	2.41	2.04	2.89

Table S8 Observed HR HPV cervical infection prevalence, posterior predictive HR HPV cervical infection prevalence, and posterior expected HR HPV cervical infection prevalence, stratified by various dimensions. ***(continued)***

							Posterior distribution							
							Predictive prevalence				Expected prevalence			
									ETI95%				ETI95%	
Region	Genotypes	Screening pathway	Year	Age	Number of tests	Observed	Average	Median	LB	UB	Average	Median	LB	UB
Auvergne-Rhône-Alpes	HPV16/18	Opportunistic	2023	[30,39]			6.83	7.04	1.41	14.08	6.79	6.77	5.83	7.88
Auvergne-Rhône-Alpes	HPV16/18	Organised	2023	[30,39]			4.31	5.26	0.00	15.79	4.38	4.34	3.45	5.49
Bourgogne-Franche-Comté	HPV16/18	Opportunistic	2023	[30,39]			5.08	4.89	2.22	8.00	5.09	5.08	4.48	5.76
Bourgogne-Franche-Comté	HPV16/18	Organised	2023	[30,39]			3.47	2.50	0.00	10.00	3.39	3.37	2.70	4.21
Bretagne	HPV16/18	Opportunistic	2023	[30,39]			5.72	5.72	4.38	7.06	5.74	5.74	5.26	6.25
Bretagne	HPV16/18	Organised	2023	[30,39]			3.24	3.23	1.72	5.16	3.23	3.21	2.63	3.96
Centre-Val de Loire	HPV16/18	Opportunistic	2023	[30,39]			5.16	5.12	4.18	6.20	5.15	5.15	4.83	5.47
Centre-Val de Loire	HPV16/18	Organised	2023	[30,39]			3.46	3.57	0.71	7.14	3.47	3.45	2.92	4.06
Corse	HPV16/18	Opportunistic	2023	[30,39]			5.13	0.00	0.00	100.00	5.09	4.93	2.81	8.48
Grand Est	HPV16/18	Opportunistic	2023	[30,39]			5.07	5.01	3.76	6.46	5.07	5.06	4.66	5.51
Grand Est	HPV16/18	Organised	2023	[30,39]			3.27	2.97	0.00	6.93	3.27	3.26	2.70	3.91
Hauts-de-France	HPV16/18	Opportunistic	2023	[30,39]			5.02	5.02	4.22	5.81	5.01	5.01	4.73	5.31
Hauts-de-France	HPV16/18	Organised	2023	[30,39]			3.33	2.88	0.72	6.47	3.32	3.31	2.76	3.93
Normandie	HPV16/18	Opportunistic	2023	[30,39]			6.02	6.03	4.58	7.47	6.03	6.03	5.61	6.46
Normandie	HPV16/18	Organised	2023	[30,39]			3.64	2.56	0.00	10.26	3.73	3.71	3.15	4.39
Nouvelle-Aquitaine	HPV16/18	Opportunistic	2023	[30,39]			5.42	5.40	4.53	6.34	5.42	5.41	5.08	5.75
Nouvelle-Aquitaine	HPV16/18	Organised	2023	[30,39]			3.72	3.69	1.97	5.67	3.76	3.75	3.19	4.41
Occitanie	HPV16/18	Opportunistic	2023	[30,39]			5.75	5.71	3.43	8.29	5.77	5.77	5.29	6.28
Occitanie	HPV16/18	Organised	2023	[30,39]			4.15	2.94	0.00	11.76	4.12	4.11	3.43	4.87
Pays de la Loire	HPV16/18	Opportunistic	2023	[30,39]			4.89	4.93	2.63	7.57	4.89	4.88	4.41	5.42
Pays de la Loire	HPV16/18	Organised	2023	[30,39]			3.14	2.84	0.71	6.38	3.15	3.14	2.58	3.77
Provence-Alpes-Côte d'Azur	HPV16/18	Opportunistic	2023	[30,39]			5.86	5.84	4.83	6.99	5.88	5.88	5.52	6.26
Provence-Alpes-Côte d'Azur	HPV16/18	Organised	2023	[30,39]			4.51	4.39	1.95	7.80	4.47	4.46	3.67	5.43
Île-de-France	HPV16/18	Opportunistic	2023	[30,39]			5.55	5.55	4.93	6.20	5.55	5.55	5.28	5.83
Île-de-France	HPV16/18	Organised	2023	[30,39]			3.87	3.73	1.87	6.13	3.88	3.87	3.25	4.60
Auvergne-Rhône-Alpes	HPV16/18	Opportunistic	2023	(39,49]	48	4.17	5.62	6.25	0.00	12.50	5.61	5.59	4.77	6.61
Auvergne-Rhône-Alpes	HPV16/18	Organised	2023	(39,49]	17	5.88	3.84	0.00	0.00	17.65	4.02	3.99	3.05	5.16
Bourgogne-Franche-Comté	HPV16/18	Opportunistic	2023	(39,49]	162	4.32	4.28	4.32	1.23	7.41	4.21	4.20	3.69	4.80
Bourgogne-Franche-Comté	HPV16/18	Organised	2023	(39,49]	64	1.56	2.86	3.12	0.00	7.81	2.84	2.82	2.29	3.49
Bretagne	HPV16/18	Opportunistic	2023	(39,49]	966	5.07	4.68	4.66	3.42	6.11	4.66	4.65	4.26	5.11
Bretagne	HPV16/18	Organised	2023	(39,49]	406	2.46	2.62	2.46	1.23	4.43	2.64	2.62	2.16	3.20
Centre-Val de Loire	HPV16/18	Opportunistic	2023	(39,49]	1,493	5.89	4.26	4.22	3.28	5.43	4.28	4.27	4.02	4.56
Centre-Val de Loire	HPV16/18	Organised	2023	(39,49]	133	3.01	3.00	3.01	0.75	6.02	2.98	2.98	2.57	3.47
Corse	HPV16/18	Opportunistic	2023	(39,49]	1	100.00	4.50	0.00	0.00	100.00	5.06	4.84	2.69	8.51
Grand Est	HPV16/18	Opportunistic	2023	(39,49]	843	4.86	4.31	4.27	2.97	5.81	4.31	4.30	3.95	4.69
Grand Est	HPV16/18	Organised	2023	(39,49]	101	1.98	2.78	2.97	0.00	6.93	2.81	2.80	2.33	3.35
Hauts-de-France	HPV16/18	Opportunistic	2023	(39,49]	2,152	5.11	4.23	4.23	3.39	5.11	4.22	4.22	3.97	4.49
Hauts-de-France	HPV16/18	Organised	2023	(39,49]	100	3.00	2.88	3.00	0.00	7.00	2.83	2.82	2.40	3.32
Normandie	HPV16/18	Opportunistic	2023	(39,49]	1,088	4.41	4.92	4.87	3.68	6.34	4.93	4.93	4.58	5.28

Table S8 Observed HR HPV cervical infection prevalence, posterior predictive HR HPV cervical infection prevalence, and posterior expected HR HPV cervical infection prevalence, stratified by various dimensions. ***(continued)***

							Posterior distribution							
							Predictive prevalence				Expected prevalence			
									ETI95%				ETI95%	
Region	Genotypes	Screening pathway	Year	Age	Number of tests	Observed	Average	Median	LB	UB	Average	Median	LB	UB
Normandie	HPV16/18	Organised	2023	(39,49]	42	0.00	3.18	2.38	0.00	9.52	3.13	3.12	2.65	3.67
Nouvelle-Aquitaine	HPV16/18	Opportunistic	2023	(39,49]	2,209	3.71	4.47	4.44	3.58	5.39	4.47	4.47	4.20	4.76
Nouvelle-Aquitaine	HPV16/18	Organised	2023	(39,49]	441	4.31	3.16	3.17	1.59	4.99	3.17	3.15	2.72	3.67
Occitanie	HPV16/18	Opportunistic	2023	(39,49]	353	5.10	4.88	4.82	2.83	7.37	4.89	4.88	4.44	5.36
Occitanie	HPV16/18	Organised	2023	(39,49]	51	0.00	3.48	3.92	0.00	9.80	3.49	3.48	2.92	4.10
Pays de la Loire	HPV16/18	Opportunistic	2023	(39,49]	299	5.69	4.08	4.01	2.01	6.35	4.09	4.08	3.69	4.55
Pays de la Loire	HPV16/18	Organised	2023	(39,49]	131	3.82	2.56	2.29	0.00	5.34	2.56	2.55	2.13	3.04
Provence-Alpes-Côte d'Azur	HPV16/18	Opportunistic	2023	(39,49]	1,551	5.54	4.77	4.77	3.74	5.93	4.76	4.76	4.45	5.09
Provence-Alpes-Côte d'Azur	HPV16/18	Organised	2023	(39,49]	139	3.60	3.68	3.60	0.72	7.19	3.67	3.65	3.07	4.39
Île-de-France	HPV16/18	Opportunistic	2023	(39,49]	3,580	4.72	4.52	4.53	3.83	5.22	4.51	4.51	4.28	4.75
Île-de-France	HPV16/18	Organised	2023	(39,49]	364	1.92	3.18	3.02	1.37	5.22	3.18	3.16	2.72	3.71
Auvergne-Rhône-Alpes	HPV16/18	Opportunistic	2023	(49,59]	45	6.67	4.39	4.44	0.00	11.11	4.46	4.44	3.73	5.32
Auvergne-Rhône-Alpes	HPV16/18	Organised	2023	(49,59]	9	0.00	3.05	0.00	0.00	11.11	3.10	3.06	2.37	4.03
Bourgogne-Franche-Comté	HPV16/18	Opportunistic	2023	(49,59]	104	1.92	3.42	2.88	0.00	7.69	3.43	3.42	3.02	3.89
Bourgogne-Franche-Comté	HPV16/18	Organised	2023	(49,59]	54	0.00	2.40	1.85	0.00	7.41	2.35	2.34	1.85	2.94
Bretagne	HPV16/18	Opportunistic	2023	(49,59]	750	4.27	3.74	3.73	2.40	5.20	3.74	3.73	3.40	4.11
Bretagne	HPV16/18	Organised	2023	(49,59]	389	2.57	2.19	2.06	0.77	3.86	2.19	2.18	1.78	2.67
Centre-Val de Loire	HPV16/18	Opportunistic	2023	(49,59]	1,356	3.02	3.46	3.47	2.51	4.42	3.45	3.45	3.22	3.68
Centre-Val de Loire	HPV16/18	Organised	2023	(49,59]	169	1.78	2.43	2.37	0.59	5.33	2.45	2.44	2.07	2.88
Grand Est	HPV16/18	Opportunistic	2023	(49,59]	693	3.46	3.46	3.46	2.16	4.91	3.44	3.43	3.13	3.76
Grand Est	HPV16/18	Organised	2023	(49,59]	129	3.88	2.25	2.33	0.00	5.43	2.28	2.26	1.86	2.75
Hauts-de-France	HPV16/18	Opportunistic	2023	(49,59]	1,553	3.86	3.36	3.35	2.45	4.31	3.36	3.36	3.15	3.59
Hauts-de-France	HPV16/18	Organised	2023	(49,59]	118	2.54	2.32	2.54	0.00	5.08	2.34	2.33	1.96	2.80
Normandie	HPV16/18	Opportunistic	2023	(49,59]	825	4.12	3.92	3.88	2.67	5.33	3.90	3.89	3.61	4.19
Normandie	HPV16/18	Organised	2023	(49,59]	35	2.86	2.67	2.86	0.00	8.57	2.59	2.58	2.18	3.05
Nouvelle-Aquitaine	HPV16/18	Opportunistic	2023	(49,59]	1,712	4.03	3.58	3.56	2.69	4.50	3.58	3.58	3.36	3.82
Nouvelle-Aquitaine	HPV16/18	Organised	2023	(49,59]	456	2.41	2.60	2.63	1.32	4.17	2.63	2.63	2.25	3.06
Occitanie	HPV16/18	Opportunistic	2023	(49,59]	307	4.56	3.90	3.91	1.95	6.19	3.90	3.89	3.53	4.29
Occitanie	HPV16/18	Organised	2023	(49,59]	60	1.67	2.78	3.33	0.00	6.67	2.83	2.82	2.35	3.38
Pays de la Loire	HPV16/18	Opportunistic	2023	(49,59]	274	2.19	3.23	3.28	1.46	5.47	3.26	3.26	2.93	3.63
Pays de la Loire	HPV16/18	Organised	2023	(49,59]	129	3.10	2.22	2.33	0.00	5.43	2.23	2.22	1.85	2.65
Provence-Alpes-Côte d'Azur	HPV16/18	Opportunistic	2023	(49,59]	1,337	3.74	3.81	3.81	2.77	4.86	3.80	3.80	3.54	4.08
Provence-Alpes-Côte d'Azur	HPV16/18	Organised	2023	(49,59]	131	3.05	2.97	3.05	0.76	6.11	3.01	2.99	2.46	3.65
Île-de-France	HPV16/18	Opportunistic	2023	(49,59]	2,833	4.52	3.57	3.57	2.89	4.27	3.57	3.57	3.37	3.79
Île-de-France	HPV16/18	Organised	2023	(49,59]	313	3.19	2.60	2.56	0.96	4.47	2.60	2.58	2.20	3.06
Auvergne-Rhône-Alpes	HPV16/18	Opportunistic	2023	(59,66]	28	0.00	4.22	3.57	0.00	14.29	4.15	4.13	3.47	4.93
Auvergne-Rhône-Alpes	HPV16/18	Organised	2023	(59,66]	4	0.00	3.01	0.00	0.00	25.00	2.90	2.87	2.15	3.77
Bourgogne-Franche-Comté	HPV16/18	Opportunistic	2023	(59,66]	60	3.33	3.18	3.33	0.00	8.33	3.21	3.20	2.79	3.68
Bourgogne-Franche-Comté	HPV16/18	Organised	2023	(59,66]	32	3.12	2.25	3.12	0.00	9.38	2.30	2.28	1.77	2.95

Table S8 Observed HR HPV cervical infection prevalence, posterior predictive HR HPV cervical infection prevalence, and posterior expected HR HPV cervical infection prevalence, stratified by various dimensions. ***(continued)***

							Posterior distribution							
							Predictive prevalence				Expected prevalence			
									ETI95%				ETI95%	
Region	Genotypes	Screening pathway	Year	Age	Number of tests	Observed	Average	Median	LB	UB	Average	Median	LB	UB
Bretagne	HPV16/18	Opportunistic	2023	(59,66]	388	4.38	3.40	3.35	1.80	5.15	3.38	3.38	3.04	3.75
Bretagne	HPV16/18	Organised	2023	(59,66]	337	1.48	2.09	2.08	0.59	3.86	2.11	2.10	1.66	2.67
Centre-Val de Loire	HPV16/18	Opportunistic	2023	(59,66]	629	3.50	3.17	3.18	1.91	4.61	3.15	3.15	2.91	3.41
Centre-Val de Loire	HPV16/18	Organised	2023	(59,66]	120	2.50	2.28	2.50	0.00	5.00	2.27	2.26	1.85	2.76
Corse	HPV16/18	Opportunistic	2023	(59,66]	3	0.00	3.07	0.00	0.00	33.33	3.41	3.35	2.42	4.69
Grand Est	HPV16/18	Opportunistic	2023	(59,66]	384	2.60	3.16	3.12	1.56	4.95	3.19	3.19	2.90	3.51
Grand Est	HPV16/18	Organised	2023	(59,66]	89	0.00	2.27	2.25	0.00	5.62	2.25	2.24	1.77	2.81
Hauts-de-France	HPV16/18	Opportunistic	2023	(59,66]	840	2.38	3.15	3.10	2.02	4.40	3.14	3.14	2.89	3.39
Hauts-de-France	HPV16/18	Organised	2023	(59,66]	116	3.45	2.31	1.72	0.00	5.17	2.31	2.30	1.87	2.82
Normandie	HPV16/18	Opportunistic	2023	(59,66]	381	3.15	3.55	3.41	1.84	5.51	3.55	3.55	3.26	3.87
Normandie	HPV16/18	Organised	2023	(59,66]	71	4.23	2.47	2.82	0.00	7.04	2.50	2.49	2.02	3.05
Nouvelle-Aquitaine	HPV16/18	Opportunistic	2023	(59,66]	859	3.84	3.33	3.26	2.10	4.54	3.34	3.34	3.09	3.63
Nouvelle-Aquitaine	HPV16/18	Organised	2023	(59,66]	308	2.60	2.57	2.60	0.97	4.55	2.55	2.53	2.08	3.08
Occitanie	HPV16/18	Opportunistic	2023	(59,66]	168	3.57	3.59	3.57	1.19	6.55	3.59	3.58	3.23	3.97
Occitanie	HPV16/18	Organised	2023	(59,66]	52	1.92	2.78	1.92	0.00	7.69	2.79	2.77	2.23	3.48
Pays de la Loire	HPV16/18	Opportunistic	2023	(59,66]	126	3.97	3.21	3.17	0.79	6.35	3.22	3.21	2.85	3.61
Pays de la Loire	HPV16/18	Organised	2023	(59,66]	97	3.09	2.13	2.06	0.00	5.15	2.12	2.11	1.70	2.59
Provence-Alpes-Côte d'Azur	HPV16/18	Opportunistic	2023	(59,66]	667	3.30	3.54	3.45	2.25	5.10	3.55	3.55	3.26	3.85
Provence-Alpes-Côte d'Azur	HPV16/18	Organised	2023	(59,66]	87	3.45	2.93	2.30	0.00	6.90	2.89	2.88	2.31	3.60
Île-de-France	HPV16/18	Opportunistic	2023	(59,66]	1,258	4.69	3.29	3.26	2.31	4.37	3.29	3.28	3.07	3.53
Île-de-France	HPV16/18	Organised	2023	(59,66]	143	4.20	2.53	2.10	0.68	5.59	2.51	2.49	2.03	3.06
Auvergne-Rhône-Alpes	Other genotypes	Opportunistic	2020	[30,39]			12.65	12.53	9.40	15.90	12.64	12.63	11.67	13.64
Bourgogne-Franche-Comté	Other genotypes	Opportunistic	2020	[30,39]			11.21	11.11	6.76	15.94	11.23	11.22	10.27	12.29
Bretagne	Other genotypes	Opportunistic	2020	[30,39]			10.92	10.92	6.99	14.86	10.91	10.91	10.02	11.85
Centre-Val de Loire	Other genotypes	Opportunistic	2020	[30,39]			11.25	11.21	9.37	13.14	11.25	11.25	10.77	11.77
Corse	Other genotypes	Opportunistic	2020	[30,39]			13.82	14.13	6.52	21.74	13.89	13.83	11.30	16.88
Grand Est	Other genotypes	Opportunistic	2020	[30,39]			11.13	11.06	7.23	15.32	11.17	11.16	10.28	12.16
Hauts-de-France	Other genotypes	Opportunistic	2020	[30,39]			10.94	10.95	9.00	12.82	10.91	10.91	10.43	11.42
Hauts-de-France	Other genotypes	Organised	2020	[30,39]			9.17	0.00	0.00	100.00	9.64	9.53	6.86	13.08
Normandie	Other genotypes	Opportunistic	2020	[30,39]			11.63	11.58	9.47	13.80	11.62	11.62	11.01	12.24
Nouvelle-Aquitaine	Other genotypes	Opportunistic	2020	[30,39]			12.00	11.89	9.51	14.74	12.01	12.01	11.47	12.56
Occitanie	Other genotypes	Opportunistic	2020	[30,39]			14.12	14.03	11.42	16.78	14.16	14.15	13.15	15.22
Pays de la Loire	Other genotypes	Opportunistic	2020	[30,39]			9.46	9.54	6.54	12.53	9.49	9.47	8.64	10.38
Pays de la Loire	Other genotypes	Organised	2020	[30,39]			6.02	0.00	0.00	33.33	6.13	6.07	4.61	8.08
Provence-Alpes-Côte d'Azur	Other genotypes	Opportunistic	2020	[30,39]			12.42	12.36	10.21	14.88	12.43	12.44	11.78	13.08
Île-de-France	Other genotypes	Opportunistic	2020	[30,39]			12.49	12.49	11.95	13.06	12.50	12.49	12.20	12.80
Île-de-France	Other genotypes	Organised	2020	[30,39]			9.92	12.50	0.00	37.50	10.12	10.09	8.11	12.47
Auvergne-Rhône-Alpes	Other genotypes	Opportunistic	2020	(39,49]	262	7.25	8.93	8.78	5.34	12.60	8.91	8.91	8.20	9.66
Bourgogne-Franche-Comté	Other genotypes	Opportunistic	2020	(39,49]	144	6.94	7.95	7.64	3.47	12.50	7.93	7.92	7.25	8.68

Table S8 Observed HR HPV cervical infection prevalence, posterior predictive HR HPV cervical infection prevalence, and posterior expected HR HPV cervical infection prevalence, stratified by various dimensions. ***(continued)***

							Posterior distribution							
							Predictive prevalence				Expected prevalence			
									ETI95%				ETI95%	
Region	Genotypes	Screening pathway	Year	Age	Number of tests	Observed	Average	Median	LB	UB	Average	Median	LB	UB
Bretagne	Other genotypes	Opportunistic	2020	(39,49]	158	10.76	7.80	7.59	3.80	12.03	7.86	7.86	7.10	8.71
Centre-Val de Loire	Other genotypes	Opportunistic	2020	(39,49]	1,001	7.59	7.91	7.89	6.29	9.69	7.89	7.89	7.54	8.27
Corse	Other genotypes	Opportunistic	2020	(39,49]	118	15.25	10.29	10.17	5.08	16.10	10.22	10.17	8.16	12.70
Grand Est	Other genotypes	Opportunistic	2020	(39,49]	241	8.71	7.85	7.88	4.56	11.21	7.84	7.83	7.16	8.61
Hauts-de-France	Other genotypes	Opportunistic	2020	(39,49]	757	7.27	7.66	7.66	5.68	9.51	7.66	7.66	7.30	8.05
Hauts-de-France	Other genotypes	Organised	2020	(39,49]	2	0.00	5.80	0.00	0.00	50.00	5.73	5.67	4.20	7.58
Normandie	Other genotypes	Opportunistic	2020	(39,49]	675	8.44	8.00	8.00	5.93	10.22	7.99	7.99	7.55	8.46
Nouvelle-Aquitaine	Other genotypes	Opportunistic	2020	(39,49]	424	7.78	8.33	8.25	5.90	11.08	8.37	8.36	7.97	8.78
Occitanie	Other genotypes	Opportunistic	2020	(39,49]	628	8.60	10.56	10.51	8.12	13.22	10.54	10.54	9.69	11.39
Pays de la Loire	Other genotypes	Opportunistic	2020	(39,49]	369	7.05	6.86	6.78	4.34	9.49	6.85	6.84	6.23	7.51
Pays de la Loire	Other genotypes	Organised	2020	(39,49]	1	0.00	4.23	0.00	0.00	100.00	4.61	4.54	3.14	6.47
Provence-Alpes-Côte d'Azur	Other genotypes	Opportunistic	2020	(39,49]	620	9.52	8.43	8.39	6.29	10.81	8.44	8.43	7.94	8.96
Île-de-France	Other genotypes	Opportunistic	2020	(39,49]	16,219	8.47	8.75	8.75	8.26	9.24	8.75	8.74	8.51	8.99
Île-de-France	Other genotypes	Organised	2020	(39,49]	1	0.00	6.10	0.00	0.00	100.00	6.33	6.29	4.81	8.22
Auvergne-Rhône-Alpes	Other genotypes	Opportunistic	2020	(49,59]	215	6.51	7.63	7.44	4.19	11.16	7.68	7.68	7.05	8.36
Bourgogne-Franche-Comté	Other genotypes	Opportunistic	2020	(49,59]	98	6.12	6.87	7.14	2.04	12.24	6.92	6.90	6.33	7.57
Bretagne	Other genotypes	Opportunistic	2020	(49,59]	107	6.54	6.61	6.54	2.80	12.15	6.58	6.56	5.91	7.32
Centre-Val de Loire	Other genotypes	Opportunistic	2020	(49,59]	971	5.77	6.64	6.59	5.15	8.34	6.65	6.64	6.33	6.98
Centre-Val de Loire	Other genotypes	Organised	2020	(49,59]	2	0.00	5.60	0.00	0.00	50.00	5.65	5.58	4.23	7.34
Corse	Other genotypes	Opportunistic	2020	(49,59]	111	6.31	8.62	8.11	3.60	14.41	8.55	8.51	6.84	10.62
Grand Est	Other genotypes	Opportunistic	2020	(49,59]	279	5.73	6.52	6.45	3.58	9.68	6.51	6.50	5.86	7.24
Hauts-de-France	Other genotypes	Opportunistic	2020	(49,59]	626	7.03	6.44	6.39	4.63	8.47	6.45	6.45	6.11	6.80
Normandie	Other genotypes	Opportunistic	2020	(49,59]	521	6.91	6.68	6.72	4.61	9.02	6.69	6.69	6.32	7.09
Nouvelle-Aquitaine	Other genotypes	Opportunistic	2020	(49,59]	367	8.45	7.10	7.08	4.63	9.82	7.12	7.12	6.75	7.52
Occitanie	Other genotypes	Opportunistic	2020	(49,59]	533	9.76	8.69	8.63	6.19	11.26	8.66	8.66	8.01	9.34
Occitanie	Other genotypes	Organised	2020	(49,59]	3	33.33	6.54	0.00	0.00	33.33	6.84	6.77	4.87	9.33
Pays de la Loire	Other genotypes	Opportunistic	2020	(49,59]	278	5.76	5.80	5.76	3.24	8.63	5.83	5.82	5.33	6.37
Pays de la Loire	Other genotypes	Organised	2020	(49,59]	1	0.00	4.43	0.00	0.00	100.00	4.49	4.41	3.13	6.28
Provence-Alpes-Côte d'Azur	Other genotypes	Opportunistic	2020	(49,59]	509	8.64	7.13	7.07	5.11	9.43	7.12	7.12	6.69	7.55
Île-de-France	Other genotypes	Opportunistic	2020	(49,59]	11,874	7.20	7.42	7.42	6.91	7.96	7.42	7.42	7.19	7.66
Île-de-France	Other genotypes	Organised	2020	(49,59]	3	0.00	7.42	0.00	0.00	33.33	7.46	7.43	5.98	9.16
Auvergne-Rhône-Alpes	Other genotypes	Opportunistic	2020	(59,66]	96	10.42	6.84	6.25	2.08	12.50	6.87	6.86	6.26	7.53
Bourgogne-Franche-Comté	Other genotypes	Opportunistic	2020	(59,66]	88	7.95	6.23	5.68	2.27	11.36	6.23	6.22	5.65	6.84
Bretagne	Other genotypes	Opportunistic	2020	(59,66]	65	4.62	5.57	6.15	0.00	12.31	5.67	5.64	5.01	6.42
Centre-Val de Loire	Other genotypes	Opportunistic	2020	(59,66]	500	3.80	5.98	6.00	4.00	8.20	5.94	5.94	5.60	6.29
Centre-Val de Loire	Other genotypes	Organised	2020	(59,66]	1	100.00	6.87	0.00	0.00	100.00	6.79	6.68	4.51	9.64
Corse	Other genotypes	Opportunistic	2020	(59,66]	56	1.79	7.61	7.14	1.79	16.07	7.65	7.59	6.23	9.39
Grand Est	Other genotypes	Opportunistic	2020	(59,66]	127	3.94	5.74	5.51	1.57	10.24	5.76	5.75	5.14	6.46
Hauts-de-France	Other genotypes	Opportunistic	2020	(59,66]	272	6.25	5.72	5.51	3.31	8.46	5.70	5.69	5.34	6.05

Table S8 Observed HR HPV cervical infection prevalence, posterior predictive HR HPV cervical infection prevalence, and posterior expected HR HPV cervical infection prevalence, stratified by various dimensions. ***(continued)***

							Posterior distribution							
							Predictive prevalence				Expected prevalence			
									ETI95%				ETI95%	
Region	Genotypes	Screening pathway	Year	Age	Number of tests	Observed	Average	Median	LB	UB	Average	Median	LB	UB
Hauts-de-France	Other genotypes	Organised	2020	(59,66]	4	0.00	5.70	0.00	0.00	25.00	5.44	5.41	4.20	6.86
Normandie	Other genotypes	Opportunistic	2020	(59,66]	262	4.58	5.81	5.73	3.05	8.78	5.88	5.87	5.48	6.29
Nouvelle-Aquitaine	Other genotypes	Opportunistic	2020	(59,66]	266	7.14	6.49	6.39	3.76	9.77	6.48	6.48	6.09	6.90
Nouvelle-Aquitaine	Other genotypes	Organised	2020	(59,66]	1	0.00	6.10	0.00	0.00	100.00	5.80	5.65	3.58	8.90
Occitanie	Other genotypes	Opportunistic	2020	(59,66]	287	6.27	7.80	7.67	4.88	10.80	7.79	7.78	7.17	8.43
Occitanie	Other genotypes	Organised	2020	(59,66]	1	0.00	6.50	0.00	0.00	100.00	6.79	6.69	4.78	9.28
Pays de la Loire	Other genotypes	Opportunistic	2020	(59,66]	142	3.52	5.43	5.63	2.11	9.86	5.44	5.43	4.93	6.03
Provence-Alpes-Côte d'Azur	Other genotypes	Opportunistic	2020	(59,66]	207	5.31	6.40	6.28	3.38	9.66	6.39	6.39	5.98	6.79
Provence-Alpes-Côte d'Azur	Other genotypes	Organised	2020	(59,66]	2	0.00	5.45	0.00	0.00	50.00	5.75	5.66	4.15	7.76
Île-de-France	Other genotypes	Opportunistic	2020	(59,66]	4,705	6.18	6.58	6.57	5.82	7.35	6.57	6.57	6.30	6.86
Île-de-France	Other genotypes	Organised	2020	(59,66]	4	0.00	5.49	0.00	0.00	25.00	5.44	5.41	4.28	6.81
Auvergne-Rhône-Alpes	Other genotypes	Opportunistic	2021	[30,39]			12.49	12.47	10.46	14.56	12.49	12.48	11.62	13.40
Auvergne-Rhône-Alpes	Other genotypes	Organised	2021	[30,39]			9.79	8.57	0.00	20.00	9.79	9.76	8.28	11.37
Bourgogne-Franche-Comté	Other genotypes	Opportunistic	2021	[30,39]			11.14	11.15	8.58	13.89	11.15	11.14	10.29	12.09
Bourgogne-Franche-Comté	Other genotypes	Organised	2021	[30,39]			8.57	0.00	0.00	33.33	8.89	8.81	7.00	11.23
Bretagne	Other genotypes	Opportunistic	2021	[30,39]			10.63	10.60	8.44	12.88	10.60	10.59	9.88	11.36
Bretagne	Other genotypes	Organised	2021	[30,39]			6.63	7.32	0.00	14.63	6.66	6.60	5.40	8.12
Centre-Val de Loire	Other genotypes	Opportunistic	2021	[30,39]			10.78	10.77	9.78	11.85	10.77	10.77	10.38	11.18
Centre-Val de Loire	Other genotypes	Organised	2021	[30,39]			7.99	7.87	4.63	12.04	7.97	7.96	7.03	9.03
Corse	Other genotypes	Opportunistic	2021	[30,39]			12.13	7.69	0.00	30.77	12.30	12.23	10.20	14.78
Grand Est	Other genotypes	Opportunistic	2021	[30,39]			10.57	10.54	8.77	12.40	10.58	10.58	10.00	11.22
Grand Est	Other genotypes	Organised	2021	[30,39]			8.59	0.00	0.00	40.00	8.37	8.33	6.78	10.24
Hauts-de-France	Other genotypes	Opportunistic	2021	[30,39]			10.46	10.45	9.54	11.43	10.46	10.46	10.08	10.87
Hauts-de-France	Other genotypes	Organised	2021	[30,39]			7.92	8.15	3.70	12.59	7.92	7.91	6.95	9.00
Normandie	Other genotypes	Opportunistic	2021	[30,39]			11.24	11.23	10.05	12.45	11.23	11.24	10.74	11.74
Normandie	Other genotypes	Organised	2021	[30,39]			7.52	0.00	0.00	33.33	7.84	7.80	6.39	9.51
Nouvelle-Aquitaine	Other genotypes	Opportunistic	2021	[30,39]			11.30	11.29	10.10	12.54	11.28	11.28	10.85	11.74
Nouvelle-Aquitaine	Other genotypes	Organised	2021	[30,39]			9.94	9.09	0.00	21.21	10.12	10.09	8.63	11.80
Occitanie	Other genotypes	Opportunistic	2021	[30,39]			12.57	12.58	10.92	14.25	12.58	12.57	11.95	13.23
Occitanie	Other genotypes	Organised	2021	[30,39]			10.14	9.62	1.92	19.23	10.12	10.09	8.77	11.62
Pays de la Loire	Other genotypes	Opportunistic	2021	[30,39]			9.10	9.07	7.07	11.18	9.11	9.11	8.42	9.86
Pays de la Loire	Other genotypes	Organised	2021	[30,39]			6.29	5.45	0.00	12.73	6.47	6.46	5.43	7.61
Provence-Alpes-Côte d'Azur	Other genotypes	Opportunistic	2021	[30,39]			11.96	11.94	10.92	13.06	11.96	11.96	11.51	12.44
Provence-Alpes-Côte d'Azur	Other genotypes	Organised	2021	[30,39]			10.74	10.00	0.00	25.00	10.62	10.59	8.96	12.51
Île-de-France	Other genotypes	Opportunistic	2021	[30,39]			11.92	11.92	11.47	12.38	11.93	11.93	11.69	12.18
Île-de-France	Other genotypes	Organised	2021	[30,39]			9.68	9.70	6.47	13.21	9.69	9.66	8.42	11.08
Auvergne-Rhône-Alpes	Other genotypes	Opportunistic	2021	(39,49]	804	7.96	8.68	8.71	6.59	10.82	8.68	8.67	8.03	9.36
Auvergne-Rhône-Alpes	Other genotypes	Organised	2021	(39,49]	48	10.42	7.19	6.25	0.00	14.58	7.23	7.22	6.09	8.43
Bourgogne-Franche-Comté	Other genotypes	Opportunistic	2021	(39,49]	391	9.46	7.82	7.67	5.12	10.74	7.79	7.78	7.15	8.47

Table S8 Observed HR HPV cervical infection prevalence, posterior predictive HR HPV cervical infection prevalence, and posterior expected HR HPV cervical infection prevalence, stratified by various dimensions. ***(continued)***

							Posterior distribution							
							Predictive prevalence				Expected prevalence			
									ETI95%				ETI95%	
Region	Genotypes	Screening pathway	Year	Age	Number of tests	Observed	Average	Median	LB	UB	Average	Median	LB	UB
Bourgogne-Franche-Comté	Other genotypes	Organised	2021	(39,49]	1	0.00	4.93	0.00	0.00	100.00	5.90	5.83	4.33	7.84
Bretagne	Other genotypes	Opportunistic	2021	(39,49]	588	8.84	7.34	7.31	5.27	9.52	7.31	7.31	6.76	7.87
Bretagne	Other genotypes	Organised	2021	(39,49]	16	0.00	4.55	6.25	0.00	18.75	4.60	4.58	3.68	5.59
Centre-Val de Loire	Other genotypes	Opportunistic	2021	(39,49]	4,201	7.28	7.55	7.55	6.69	8.43	7.55	7.55	7.25	7.85
Centre-Val de Loire	Other genotypes	Organised	2021	(39,49]	138	2.17	5.97	5.80	2.17	10.14	5.96	5.96	5.22	6.75
Corse	Other genotypes	Opportunistic	2021	(39,49]	5	0.00	8.41	0.00	0.00	40.00	8.56	8.53	6.83	10.46
Grand Est	Other genotypes	Opportunistic	2021	(39,49]	1,093	7.14	7.52	7.50	5.86	9.24	7.52	7.52	7.08	8.00
Grand Est	Other genotypes	Organised	2021	(39,49]	26	3.85	5.61	3.85	0.00	15.38	5.64	5.63	4.78	6.58
Hauts-de-France	Other genotypes	Opportunistic	2021	(39,49]	2,916	7.82	7.39	7.37	6.41	8.44	7.39	7.39	7.10	7.69
Hauts-de-France	Other genotypes	Organised	2021	(39,49]	110	1.82	5.97	5.45	1.82	10.91	5.93	5.92	5.17	6.77
Normandie	Other genotypes	Opportunistic	2021	(39,49]	2,310	7.92	7.90	7.88	6.80	9.09	7.90	7.90	7.54	8.27
Normandie	Other genotypes	Organised	2021	(39,49]	5	20.00	6.05	0.00	0.00	40.00	6.23	6.21	5.07	7.53
Nouvelle-Aquitaine	Other genotypes	Opportunistic	2021	(39,49]	2,359	7.97	7.85	7.84	6.74	9.03	7.86	7.86	7.55	8.19
Nouvelle-Aquitaine	Other genotypes	Organised	2021	(39,49]	3	0.00	5.99	0.00	0.00	33.33	6.19	6.14	4.93	7.68
Occitanie	Other genotypes	Opportunistic	2021	(39,49]	1,638	10.32	8.98	8.97	7.51	10.50	8.97	8.97	8.49	9.47
Occitanie	Other genotypes	Organised	2021	(39,49]	110	6.36	7.34	7.27	2.73	12.73	7.37	7.35	6.34	8.52
Pays de la Loire	Other genotypes	Opportunistic	2021	(39,49]	834	6.71	6.58	6.59	4.92	8.39	6.57	6.57	6.08	7.10
Pays de la Loire	Other genotypes	Organised	2021	(39,49]	85	3.53	5.15	4.71	1.18	10.59	5.09	5.07	4.30	5.96
Provence-Alpes-Côte d'Azur	Other genotypes	Opportunistic	2021	(39,49]	3,170	8.30	8.34	8.36	7.38	9.37	8.34	8.33	7.97	8.71
Provence-Alpes-Côte d'Azur	Other genotypes	Organised	2021	(39,49]	7	14.29	7.61	0.00	0.00	28.57	7.49	7.46	6.26	8.95
Île-de-France	Other genotypes	Opportunistic	2021	(39,49]	25,340	8.13	8.34	8.34	7.96	8.73	8.34	8.34	8.14	8.55
Île-de-France	Other genotypes	Organised	2021	(39,49]	83	1.20	6.53	6.02	1.20	12.05	6.62	6.61	5.83	7.44
Auvergne-Rhône-Alpes	Other genotypes	Opportunistic	2021	(49,59]	666	6.91	7.39	7.36	5.26	9.46	7.36	7.35	6.81	7.94
Auvergne-Rhône-Alpes	Other genotypes	Organised	2021	(49,59]	38	7.89	6.42	5.26	0.00	15.79	6.68	6.67	5.62	7.83
Bourgogne-Franche-Comté	Other genotypes	Opportunistic	2021	(49,59]	329	6.69	6.70	6.69	3.95	9.42	6.68	6.67	6.14	7.25
Bourgogne-Franche-Comté	Other genotypes	Organised	2021	(49,59]	1	0.00	4.77	0.00	0.00	100.00	5.37	5.31	3.99	7.04
Bretagne	Other genotypes	Opportunistic	2021	(49,59]	556	3.96	6.07	6.12	3.96	8.09	6.05	6.05	5.58	6.52
Bretagne	Other genotypes	Organised	2021	(49,59]	36	2.78	4.37	2.78	0.00	11.11	4.34	4.32	3.54	5.23
Centre-Val de Loire	Other genotypes	Opportunistic	2021	(49,59]	3,844	5.93	6.39	6.37	5.59	7.21	6.38	6.39	6.11	6.66
Centre-Val de Loire	Other genotypes	Organised	2021	(49,59]	224	5.80	5.41	5.36	2.68	8.49	5.38	5.38	4.77	6.03
Corse	Other genotypes	Opportunistic	2021	(49,59]	9	0.00	6.79	0.00	0.00	22.22	6.90	6.89	5.96	7.93
Corse	Other genotypes	Organised	2021	(49,59]	1	0.00	6.40	0.00	0.00	100.00	6.17	6.05	4.07	8.97
Grand Est	Other genotypes	Opportunistic	2021	(49,59]	1,003	5.08	6.42	6.38	4.99	7.98	6.42	6.41	6.03	6.82
Grand Est	Other genotypes	Organised	2021	(49,59]	59	1.69	5.23	5.08	0.00	11.86	5.21	5.21	4.40	6.07
Hauts-de-France	Other genotypes	Opportunistic	2021	(49,59]	2,015	6.50	6.25	6.25	5.26	7.39	6.27	6.27	6.01	6.55
Hauts-de-France	Other genotypes	Organised	2021	(49,59]	96	5.21	5.42	5.21	1.04	10.42	5.43	5.43	4.80	6.12
Normandie	Other genotypes	Opportunistic	2021	(49,59]	1,829	5.36	6.61	6.56	5.47	7.76	6.60	6.60	6.29	6.93
Normandie	Other genotypes	Organised	2021	(49,59]	5	0.00	5.33	0.00	0.00	20.50	5.46	5.44	4.45	6.62
Nouvelle-Aquitaine	Other genotypes	Opportunistic	2021	(49,59]	1,995	6.47	6.65	6.62	5.56	7.82	6.65	6.65	6.38	6.95

Table S8 Observed HR HPV cervical infection prevalence, posterior predictive HR HPV cervical infection prevalence, and posterior expected HR HPV cervical infection prevalence, stratified by various dimensions. ***(continued)***

							Posterior distribution							
							Predictive prevalence				Expected prevalence			
									ETI95%				ETI95%	
Region	Genotypes	Screening pathway	Year	Age	Number of tests	Observed	Average	Median	LB	UB	Average	Median	LB	UB
Nouvelle-Aquitaine	Other genotypes	Organised	2021	(49,59]	170	6.47	5.88	5.88	2.35	10.00	5.84	5.82	5.16	6.56
Occitanie	Other genotypes	Opportunistic	2021	(49,59]	1,372	7.94	7.72	7.65	6.34	9.26	7.72	7.71	7.29	8.16
Occitanie	Other genotypes	Organised	2021	(49,59]	95	4.21	7.17	7.37	2.11	12.63	7.28	7.25	6.20	8.45
Pays de la Loire	Other genotypes	Opportunistic	2021	(49,59]	703	5.41	5.60	5.55	3.98	7.40	5.60	5.58	5.17	6.07
Pays de la Loire	Other genotypes	Organised	2021	(49,59]	78	6.41	4.85	5.13	0.00	10.26	4.84	4.83	4.11	5.63
Provence-Alpes-Côte d'Azur	Other genotypes	Opportunistic	2021	(49,59]	2,577	7.37	7.00	7.02	5.98	8.07	7.01	7.00	6.70	7.34
Provence-Alpes-Côte d'Azur	Other genotypes	Organised	2021	(49,59]	7	14.29	6.50	0.00	0.00	28.57	6.68	6.64	5.46	8.13
Île-de-France	Other genotypes	Opportunistic	2021	(49,59]	18,998	7.08	7.06	7.06	6.67	7.48	7.06	7.06	6.87	7.27
Île-de-France	Other genotypes	Organised	2021	(49,59]	161	7.45	5.90	5.59	2.48	9.94	5.93	5.92	5.30	6.66
Auvergne-Rhône-Alpes	Other genotypes	Opportunistic	2021	(59,66]	330	5.76	6.47	6.36	3.94	9.09	6.50	6.49	5.98	7.04
Auvergne-Rhône-Alpes	Other genotypes	Organised	2021	(59,66]	31	3.23	6.38	6.45	0.00	16.13	6.42	6.41	5.43	7.53
Bourgogne-Franche-Comté	Other genotypes	Opportunistic	2021	(59,66]	207	7.25	6.01	5.80	2.90	9.66	6.02	6.02	5.49	6.57
Bourgogne-Franche-Comté	Other genotypes	Organised	2021	(59,66]	22	13.64	5.36	4.55	0.00	18.18	5.51	5.49	4.60	6.55
Bretagne	Other genotypes	Opportunistic	2021	(59,66]	239	4.18	5.45	5.44	2.93	8.79	5.44	5.44	4.99	5.92
Bretagne	Other genotypes	Organised	2021	(59,66]	21	4.76	4.51	4.76	0.00	14.29	4.48	4.44	3.59	5.56
Centre-Val de Loire	Other genotypes	Opportunistic	2021	(59,66]	2,018	6.19	5.67	5.65	4.66	6.79	5.68	5.68	5.38	5.99
Centre-Val de Loire	Other genotypes	Organised	2021	(59,66]	143	4.20	5.19	4.90	2.10	9.09	5.16	5.14	4.51	5.88
Corse	Other genotypes	Opportunistic	2021	(59,66]	7	0.00	6.80	0.00	0.00	28.57	6.66	6.61	5.48	8.06
Grand Est	Other genotypes	Opportunistic	2021	(59,66]	562	3.38	5.75	5.69	3.91	7.83	5.75	5.74	5.37	6.14
Grand Est	Other genotypes	Organised	2021	(59,66]	46	2.17	5.02	4.35	0.00	13.04	5.05	5.05	4.21	5.93
Hauts-de-France	Other genotypes	Opportunistic	2021	(59,66]	931	4.08	5.57	5.59	4.19	7.09	5.56	5.56	5.27	5.86
Hauts-de-France	Other genotypes	Organised	2021	(59,66]	49	6.12	5.48	6.12	0.00	12.24	5.39	5.38	4.63	6.19
Normandie	Other genotypes	Opportunistic	2021	(59,66]	950	5.68	5.88	5.89	4.32	7.47	5.88	5.89	5.55	6.23
Normandie	Other genotypes	Organised	2021	(59,66]	18	11.11	5.54	5.56	0.00	16.67	5.47	5.46	4.64	6.39
Nouvelle-Aquitaine	Other genotypes	Opportunistic	2021	(59,66]	1,222	4.58	6.00	5.97	4.66	7.36	6.01	6.01	5.70	6.34
Nouvelle-Aquitaine	Other genotypes	Organised	2021	(59,66]	185	6.49	5.65	5.41	2.70	9.19	5.72	5.71	4.96	6.49
Occitanie	Other genotypes	Opportunistic	2021	(59,66]	720	7.64	6.83	6.81	5.00	8.75	6.87	6.87	6.44	7.32
Occitanie	Other genotypes	Organised	2021	(59,66]	48	12.50	6.93	6.25	0.00	14.58	6.99	6.95	5.97	8.17
Pays de la Loire	Other genotypes	Opportunistic	2021	(59,66]	326	3.37	5.25	5.21	2.76	7.98	5.25	5.24	4.81	5.73
Pays de la Loire	Other genotypes	Organised	2021	(59,66]	52	1.92	4.85	3.85	0.00	11.54	4.81	4.80	4.02	5.72
Provence-Alpes-Côte d'Azur	Other genotypes	Opportunistic	2021	(59,66]	1,314	7.15	6.26	6.24	4.95	7.53	6.25	6.25	5.91	6.59
Provence-Alpes-Côte d'Azur	Other genotypes	Organised	2021	(59,66]	22	4.55	6.03	4.55	0.00	18.18	6.03	6.00	4.99	7.20
Île-de-France	Other genotypes	Opportunistic	2021	(59,66]	8,881	6.49	6.32	6.32	5.78	6.88	6.31	6.31	6.07	6.57
Île-de-France	Other genotypes	Organised	2021	(59,66]	1,384	5.78	5.77	5.78	4.34	7.37	5.76	5.74	5.01	6.60
Auvergne-Rhône-Alpes	Other genotypes	Opportunistic	2022	[30,39]			13.83	14.02	7.48	20.56	13.89	13.88	12.74	15.08
Auvergne-Rhône-Alpes	Other genotypes	Organised	2022	[30,39]			10.02	8.33	0.00	25.00	9.90	9.88	8.34	11.61
Bourgogne-Franche-Comté	Other genotypes	Opportunistic	2022	[30,39]			11.85	11.80	8.90	15.11	11.86	11.85	10.91	12.89
Bourgogne-Franche-Comté	Other genotypes	Organised	2022	[30,39]			9.33	7.69	0.00	23.08	9.41	9.36	7.81	11.20
Bretagne	Other genotypes	Opportunistic	2022	[30,39]			11.88	11.89	9.81	14.07	11.85	11.85	11.07	12.64

Table S8 **Observed HR HPV cervical infection prevalence, posterior predictive HR HPV cervical infection prevalence, and posterior expected HR HPV cervical infection prevalence, stratified by various dimensions.** ***(continued)***

							Posterior distribution							
							Predictive prevalence				Expected prevalence			
									ETI95%				ETI95%	
Region	Genotypes	Screening pathway	Year	Age	Number of tests	Observed	Average	Median	LB	UB	Average	Median	LB	UB
Bretagne	Other genotypes	Organised	2022	[30,39]			7.72	7.64	3.82	12.12	7.71	7.67	6.49	9.15
Centre-Val de Loire	Other genotypes	Opportunistic	2022	[30,39]			11.25	11.23	9.91	12.64	11.23	11.23	10.77	11.70
Centre-Val de Loire	Other genotypes	Organised	2022	[30,39]			8.16	8.33	4.41	12.25	8.12	8.10	7.29	9.04
Corse	Other genotypes	Opportunistic	2022	[30,39]			12.17	0.00	0.00	50.00	11.67	11.61	9.76	13.89
Grand Est	Other genotypes	Opportunistic	2022	[30,39]			11.44	11.44	9.52	13.45	11.42	11.41	10.77	12.12
Grand Est	Other genotypes	Organised	2022	[30,39]			7.98	8.11	4.50	11.71	7.97	7.95	6.90	9.05
Hauts-de-France	Other genotypes	Opportunistic	2022	[30,39]			11.14	11.16	10.07	12.20	11.13	11.13	10.71	11.56
Hauts-de-France	Other genotypes	Organised	2022	[30,39]			8.14	8.10	5.31	11.17	8.15	8.13	7.23	9.10
Normandie	Other genotypes	Opportunistic	2022	[30,39]			12.33	12.31	10.86	13.85	12.31	12.31	11.75	12.87
Normandie	Other genotypes	Organised	2022	[30,39]			8.72	9.09	0.00	22.73	8.83	8.79	7.59	10.21
Nouvelle-Aquitaine	Other genotypes	Opportunistic	2022	[30,39]			12.03	12.01	10.82	13.25	12.04	12.03	11.60	12.51
Nouvelle-Aquitaine	Other genotypes	Organised	2022	[30,39]			9.45	9.29	6.09	12.82	9.44	9.42	8.48	10.48
Occitanie	Other genotypes	Opportunistic	2022	[30,39]			12.92	12.82	10.62	15.38	12.91	12.90	12.19	13.66
Occitanie	Other genotypes	Organised	2022	[30,39]			10.15	9.86	5.63	15.49	10.11	10.09	8.95	11.39
Pays de la Loire	Other genotypes	Opportunistic	2022	[30,39]			10.59	10.59	7.06	14.51	10.60	10.58	9.77	11.47
Pays de la Loire	Other genotypes	Organised	2022	[30,39]			7.20	6.35	1.59	14.29	7.15	7.13	6.18	8.17
Provence-Alpes-Côte d'Azur	Other genotypes	Opportunistic	2022	[30,39]			12.81	12.82	11.50	14.09	12.81	12.81	12.31	13.35
Provence-Alpes-Côte d'Azur	Other genotypes	Organised	2022	[30,39]			11.27	11.20	7.47	15.77	11.33	11.27	9.88	13.08
Île-de-France	Other genotypes	Opportunistic	2022	[30,39]			12.96	12.96	12.08	13.85	12.96	12.96	12.57	13.34
Île-de-France	Other genotypes	Organised	2022	[30,39]			9.93	9.91	6.50	13.62	9.95	9.94	8.92	11.04
Auvergne-Rhône-Alpes	Other genotypes	Opportunistic	2022	(39,49]	64	17.19	10.04	9.38	3.12	18.75	10.11	10.09	8.95	11.36
Auvergne-Rhône-Alpes	Other genotypes	Organised	2022	(39,49]	15	13.33	7.92	6.67	0.00	26.67	8.01	7.98	6.71	9.44
Bourgogne-Franche-Comté	Other genotypes	Opportunistic	2022	(39,49]	333	8.71	8.18	8.11	5.11	11.41	8.18	8.17	7.49	8.90
Bourgogne-Franche-Comté	Other genotypes	Organised	2022	(39,49]	1	0.00	6.63	0.00	0.00	100.00	6.45	6.38	4.66	8.65
Bretagne	Other genotypes	Opportunistic	2022	(39,49]	663	7.69	8.33	8.30	6.18	10.56	8.33	8.32	7.75	8.92
Bretagne	Other genotypes	Organised	2022	(39,49]	173	5.78	5.40	5.20	2.31	9.25	5.44	5.43	4.62	6.37
Centre-Val de Loire	Other genotypes	Opportunistic	2022	(39,49]	2,079	8.37	7.86	7.84	6.69	9.09	7.86	7.86	7.51	8.22
Centre-Val de Loire	Other genotypes	Organised	2022	(39,49]	248	6.05	6.14	6.05	3.23	9.27	6.14	6.13	5.52	6.78
Corse	Other genotypes	Opportunistic	2022	(39,49]	3	33.33	10.10	0.00	0.00	66.67	9.62	9.54	7.61	11.95
Corse	Other genotypes	Organised	2022	(39,49]	2	0.00	6.48	0.00	0.00	50.00	6.92	6.82	5.15	9.08
Grand Est	Other genotypes	Opportunistic	2022	(39,49]	858	10.14	8.15	8.16	6.29	10.14	8.17	8.16	7.67	8.72
Grand Est	Other genotypes	Organised	2022	(39,49]	198	8.59	6.06	6.06	3.03	9.60	6.12	6.11	5.29	6.98
Hauts-de-France	Other genotypes	Opportunistic	2022	(39,49]	2,382	8.73	7.85	7.85	6.72	9.03	7.84	7.84	7.51	8.18
Hauts-de-France	Other genotypes	Organised	2022	(39,49]	286	5.94	6.21	6.29	3.50	9.44	6.18	6.17	5.53	6.86
Normandie	Other genotypes	Opportunistic	2022	(39,49]	1,814	8.27	8.64	8.65	7.28	10.03	8.64	8.63	8.21	9.05
Normandie	Other genotypes	Organised	2022	(39,49]	22	13.64	6.52	4.55	0.00	18.18	6.58	6.57	5.79	7.45
Nouvelle-Aquitaine	Other genotypes	Opportunistic	2022	(39,49]	2,812	8.04	8.52	8.50	7.43	9.64	8.54	8.53	8.19	8.90
Nouvelle-Aquitaine	Other genotypes	Organised	2022	(39,49]	337	7.12	6.90	6.82	4.15	9.79	6.89	6.88	6.24	7.63
Occitanie	Other genotypes	Opportunistic	2022	(39,49]	746	8.58	9.14	9.12	7.10	11.26	9.15	9.14	8.60	9.73

Table S8 **Observed HR HPV cervical infection prevalence, posterior predictive HR HPV cervical infection prevalence, and posterior expected HR HPV cervical infection prevalence, stratified by various dimensions.** ***(continued)***

							Posterior distribution							
							Predictive prevalence				Expected prevalence			
									ETI95%				ETI95%	
Region	Genotypes	Screening pathway	Year	Age	Number of tests	Observed	Average	Median	LB	UB	Average	Median	LB	UB
Occitanie	Other genotypes	Organised	2022	(39,49]	135	5.93	7.43	7.41	3.70	11.85	7.50	7.48	6.62	8.47
Pays de la Loire	Other genotypes	Opportunistic	2022	(39,49]	203	7.88	7.51	7.39	3.94	11.33	7.53	7.52	6.91	8.20
Pays de la Loire	Other genotypes	Organised	2022	(39,49]	71	1.41	5.59	5.63	1.41	11.27	5.63	5.61	4.89	6.40
Provence-Alpes-Côte d'Azur	Other genotypes	Opportunistic	2022	(39,49]	2,056	8.46	8.94	8.95	7.68	10.21	8.95	8.95	8.58	9.35
Provence-Alpes-Côte d'Azur	Other genotypes	Organised	2022	(39,49]	112	5.36	8.29	8.04	3.57	14.29	8.37	8.33	7.27	9.71
Île-de-France	Other genotypes	Opportunistic	2022	(39,49]	4,399	7.96	8.80	8.80	7.91	9.68	8.80	8.80	8.51	9.10
Île-de-France	Other genotypes	Organised	2022	(39,49]	342	9.65	7.20	7.02	4.39	10.23	7.23	7.21	6.53	8.00
Auvergne-Rhône-Alpes	Other genotypes	Opportunistic	2022	(49,59]	49	20.41	8.94	8.16	2.04	18.37	8.92	8.90	7.86	10.07
Auvergne-Rhône-Alpes	Other genotypes	Organised	2022	(49,59]	13	7.69	7.86	7.69	0.00	23.08	7.79	7.75	6.31	9.49
Bourgogne-Franche-Comté	Other genotypes	Opportunistic	2022	(49,59]	239	7.53	6.98	7.11	3.77	10.46	6.99	6.98	6.41	7.63
Bourgogne-Franche-Comté	Other genotypes	Organised	2022	(49,59]	1	100.00	6.30	0.00	0.00	100.00	6.41	6.32	4.58	8.74
Bretagne	Other genotypes	Opportunistic	2022	(49,59]	535	7.85	7.04	7.10	4.86	9.35	7.06	7.05	6.54	7.59
Bretagne	Other genotypes	Organised	2022	(49,59]	144	2.78	5.02	4.86	2.08	9.03	5.04	5.01	4.26	5.95
Centre-Val de Loire	Other genotypes	Opportunistic	2022	(49,59]	1,838	6.58	6.66	6.64	5.55	7.83	6.66	6.66	6.34	6.98
Centre-Val de Loire	Other genotypes	Organised	2022	(49,59]	278	7.19	5.73	5.76	3.24	8.63	5.75	5.75	5.18	6.37
Corse	Other genotypes	Opportunistic	2022	(49,59]	2	0.00	8.30	0.00	0.00	50.00	7.65	7.56	5.63	10.09
Grand Est	Other genotypes	Opportunistic	2022	(49,59]	767	6.13	7.00	7.04	5.22	8.87	7.04	7.03	6.57	7.53
Grand Est	Other genotypes	Organised	2022	(49,59]	120	4.17	5.94	5.83	1.67	10.02	5.92	5.91	5.18	6.72
Hauts-de-France	Other genotypes	Opportunistic	2022	(49,59]	1,725	8.17	6.66	6.67	5.51	7.94	6.67	6.67	6.38	6.98
Hauts-de-France	Other genotypes	Organised	2022	(49,59]	267	7.49	5.71	5.62	3.00	8.99	5.72	5.71	5.11	6.40
Normandie	Other genotypes	Opportunistic	2022	(49,59]	1,196	6.77	7.16	7.11	5.69	8.70	7.18	7.18	6.82	7.55
Normandie	Other genotypes	Organised	2022	(49,59]	16	6.25	6.12	6.25	0.00	18.75	5.98	5.98	5.18	6.82
Nouvelle-Aquitaine	Other genotypes	Opportunistic	2022	(49,59]	2,090	7.32	7.25	7.22	6.17	8.47	7.25	7.25	6.93	7.59
Nouvelle-Aquitaine	Other genotypes	Organised	2022	(49,59]	644	6.21	6.31	6.21	4.35	8.39	6.30	6.29	5.71	6.96
Occitanie	Other genotypes	Opportunistic	2022	(49,59]	710	7.75	7.80	7.75	5.77	9.86	7.81	7.80	7.34	8.33
Occitanie	Other genotypes	Organised	2022	(49,59]	141	7.80	7.08	7.09	2.84	11.35	7.09	7.06	6.26	8.02
Pays de la Loire	Other genotypes	Opportunistic	2022	(49,59]	158	7.59	6.57	6.33	2.53	10.76	6.55	6.55	6.00	7.17
Pays de la Loire	Other genotypes	Organised	2022	(49,59]	67	4.48	5.29	4.48	0.00	10.49	5.24	5.24	4.58	5.99
Provence-Alpes-Côte d'Azur	Other genotypes	Opportunistic	2022	(49,59]	1,791	7.31	7.48	7.48	6.25	8.77	7.50	7.50	7.15	7.85
Provence-Alpes-Côte d'Azur	Other genotypes	Organised	2022	(49,59]	85	8.24	7.62	7.06	2.35	14.12	7.70	7.66	6.65	9.03
Île-de-France	Other genotypes	Opportunistic	2022	(49,59]	3,353	7.52	7.38	7.37	6.47	8.35	7.38	7.38	7.10	7.65
Île-de-France	Other genotypes	Organised	2022	(49,59]	449	7.35	6.50	6.46	4.23	8.91	6.49	6.48	5.83	7.19
Auvergne-Rhône-Alpes	Other genotypes	Opportunistic	2022	(59,66]	27	3.70	7.35	7.41	0.00	18.52	7.46	7.46	6.65	8.34
Auvergne-Rhône-Alpes	Other genotypes	Organised	2022	(59,66]	8	0.00	7.75	0.00	0.00	25.00	7.74	7.69	6.09	9.70
Bourgogne-Franche-Comté	Other genotypes	Opportunistic	2022	(59,66]	120	5.83	6.15	5.83	2.50	10.83	6.19	6.19	5.62	6.83
Bourgogne-Franche-Comté	Other genotypes	Organised	2022	(59,66]	17	5.88	5.69	5.88	0.00	17.65	5.75	5.73	4.76	6.86
Bretagne	Other genotypes	Opportunistic	2022	(59,66]	289	7.96	6.18	6.23	3.46	9.00	6.21	6.20	5.71	6.71
Bretagne	Other genotypes	Organised	2022	(59,66]	112	1.79	4.67	4.46	0.89	8.93	4.69	4.67	3.89	5.64
Centre-Val de Loire	Other genotypes	Opportunistic	2022	(59,66]	872	6.88	5.88	5.85	4.36	7.46	5.88	5.88	5.56	6.22

Table S8 **Observed HR HPV cervical infection prevalence, posterior predictive HR HPV cervical infection prevalence, and posterior expected HR HPV cervical infection prevalence, stratified by various dimensions.** ***(continued)***

							Posterior distribution							
							Predictive prevalence				Expected prevalence			
									ETI95%				ETI95%	
Region	Genotypes	Screening pathway	Year	Age	Number of tests	Observed	Average	Median	LB	UB	Average	Median	LB	UB
Centre-Val de Loire	Other genotypes	Organised	2022	(59,66]	188	7.45	5.46	5.32	2.66	9.04	5.45	5.43	4.80	6.20
Corse	Other genotypes	Opportunistic	2022	(59,66]	2	50.00	8.23	0.00	0.00	50.00	8.93	8.79	6.33	12.22
Grand Est	Other genotypes	Opportunistic	2022	(59,66]	312	5.77	6.29	6.09	3.85	8.97	6.25	6.25	5.81	6.71
Grand Est	Other genotypes	Organised	2022	(59,66]	82	4.88	5.83	6.10	1.22	10.98	5.80	5.78	4.95	6.73
Hauts-de-France	Other genotypes	Opportunistic	2022	(59,66]	714	6.16	5.99	6.02	4.34	7.84	5.99	5.99	5.68	6.32
Hauts-de-France	Other genotypes	Organised	2022	(59,66]	169	5.33	5.59	5.33	2.37	9.47	5.58	5.56	4.85	6.38
Normandie	Other genotypes	Opportunistic	2022	(59,66]	612	5.07	6.37	6.37	4.41	8.33	6.35	6.35	5.98	6.74
Normandie	Other genotypes	Organised	2022	(59,66]	93	2.15	5.73	5.38	1.08	10.75	5.62	5.60	4.83	6.52
Nouvelle-Aquitaine	Other genotypes	Opportunistic	2022	(59,66]	1,040	6.06	6.45	6.44	5.00	7.98	6.45	6.45	6.10	6.80
Nouvelle-Aquitaine	Other genotypes	Organised	2022	(59,66]	440	7.27	6.07	6.14	3.86	8.64	6.08	6.06	5.36	6.89
Occitanie	Other genotypes	Opportunistic	2022	(59,66]	349	6.02	6.96	6.88	4.58	9.74	6.94	6.93	6.47	7.46
Occitanie	Other genotypes	Organised	2022	(59,66]	89	5.62	6.87	6.74	2.25	12.36	6.77	6.75	5.90	7.79
Pays de la Loire	Other genotypes	Opportunistic	2022	(59,66]	105	7.62	6.00	5.71	1.90	10.48	6.03	6.02	5.44	6.66
Pays de la Loire	Other genotypes	Organised	2022	(59,66]	40	0.00	5.22	5.00	0.00	12.50	5.21	5.20	4.42	6.07
Provence-Alpes-Côte d'Azur	Other genotypes	Opportunistic	2022	(59,66]	836	6.22	6.66	6.70	4.90	8.49	6.66	6.66	6.29	7.03
Provence-Alpes-Côte d'Azur	Other genotypes	Organised	2022	(59,66]	66	6.06	7.15	7.58	1.52	13.64	7.13	7.08	6.03	8.37
Île-de-France	Other genotypes	Opportunistic	2022	(59,66]	1,520	5.99	6.60	6.58	5.39	7.89	6.59	6.58	6.28	6.90
Île-de-France	Other genotypes	Organised	2022	(59,66]	193	4.15	6.24	6.22	3.11	9.84	6.23	6.21	5.52	7.04
Auvergne-Rhône-Alpes	Other genotypes	Opportunistic	2023	[30,39]			17.58	16.90	8.45	26.76	17.57	17.57	15.51	19.69
Auvergne-Rhône-Alpes	Other genotypes	Organised	2023	[30,39]			11.80	10.53	0.00	26.32	11.85	11.84	9.79	14.15
Bourgogne-Franche-Comté	Other genotypes	Opportunistic	2023	[30,39]			13.90	13.78	9.33	19.11	13.92	13.91	12.63	15.28
Bourgogne-Franche-Comté	Other genotypes	Organised	2023	[30,39]			8.24	7.50	0.00	17.50	8.37	8.34	6.99	10.03
Bretagne	Other genotypes	Opportunistic	2023	[30,39]			14.22	14.19	12.11	16.34	14.22	14.21	13.35	15.13
Bretagne	Other genotypes	Organised	2023	[30,39]			7.76	7.74	5.16	10.54	7.75	7.71	6.66	9.01
Centre-Val de Loire	Other genotypes	Opportunistic	2023	[30,39]			13.14	13.13	11.61	14.71	13.16	13.16	12.63	13.74
Centre-Val de Loire	Other genotypes	Organised	2023	[30,39]			8.43	8.57	3.57	13.57	8.53	8.50	7.59	9.57
Corse	Other genotypes	Opportunistic	2023	[30,39]			11.07	0.00	0.00	100.00	10.92	10.55	6.24	17.33
Grand Est	Other genotypes	Opportunistic	2023	[30,39]			13.33	13.31	11.09	15.62	13.32	13.31	12.55	14.13
Grand Est	Other genotypes	Organised	2023	[30,39]			8.14	7.92	2.97	13.86	8.19	8.19	7.08	9.41
Hauts-de-France	Other genotypes	Opportunistic	2023	[30,39]			12.93	12.92	11.71	14.19	12.94	12.94	12.44	13.44
Hauts-de-France	Other genotypes	Organised	2023	[30,39]			8.37	7.91	4.32	13.67	8.38	8.37	7.35	9.51
Normandie	Other genotypes	Opportunistic	2023	[30,39]			14.31	14.26	12.22	16.47	14.32	14.33	13.66	15.01
Normandie	Other genotypes	Organised	2023	[30,39]			8.55	7.69	0.00	17.95	8.50	8.50	7.45	9.61
Nouvelle-Aquitaine	Other genotypes	Opportunistic	2023	[30,39]			14.27	14.28	12.87	15.69	14.26	14.26	13.72	14.83
Nouvelle-Aquitaine	Other genotypes	Organised	2023	[30,39]			9.40	9.36	6.65	12.56	9.46	9.43	8.47	10.50
Occitanie	Other genotypes	Opportunistic	2023	[30,39]			15.33	15.14	11.71	19.43	15.30	15.27	14.36	16.34
Occitanie	Other genotypes	Organised	2023	[30,39]			10.06	8.82	0.00	20.59	10.07	10.05	8.81	11.50
Pays de la Loire	Other genotypes	Opportunistic	2023	[30,39]			12.44	12.50	8.88	16.45	12.44	12.42	11.44	13.52
Pays de la Loire	Other genotypes	Organised	2023	[30,39]			7.72	7.80	3.55	12.77	7.79	7.78	6.76	8.93

Table S8 **Observed HR HPV cervical infection prevalence, posterior predictive HR HPV cervical infection prevalence, and posterior expected HR HPV cervical infection prevalence, stratified by various dimensions.** ***(continued)***

							Posterior distribution							
							Predictive prevalence				Expected prevalence			
									ETI95%				ETI95%	
Region	Genotypes	Screening pathway	Year	Age	Number of tests	Observed	Average	Median	LB	UB	Average	Median	LB	UB
Provence-Alpes-Côte d'Azur	Other genotypes	Opportunistic	2023	[30,39]			15.31	15.31	13.68	16.99	15.31	15.31	14.68	15.93
Provence-Alpes-Côte d'Azur	Other genotypes	Organised	2023	[30,39]			11.28	11.22	6.83	16.10	11.36	11.31	9.81	13.12
Île-de-France	Other genotypes	Opportunistic	2023	[30,39]			15.44	15.43	14.43	16.45	15.44	15.44	14.99	15.90
Île-de-France	Other genotypes	Organised	2023	[30,39]			10.34	10.40	7.20	13.87	10.37	10.34	9.27	11.64
Auvergne-Rhône-Alpes	Other genotypes	Opportunistic	2023	(39,49]	48	8.33	12.81	12.50	4.17	22.92	12.60	12.58	10.91	14.43
Auvergne-Rhône-Alpes	Other genotypes	Organised	2023	(39,49]	17	5.88	8.97	5.88	0.00	23.53	9.20	9.14	7.30	11.48
Bourgogne-Franche-Comté	Other genotypes	Opportunistic	2023	(39,49]	162	9.88	9.65	9.26	5.56	14.81	9.59	9.58	8.60	10.61
Bourgogne-Franche-Comté	Other genotypes	Organised	2023	(39,49]	64	4.69	6.61	6.25	1.56	14.06	6.60	6.57	5.50	7.87
Bretagne	Other genotypes	Opportunistic	2023	(39,49]	966	11.70	9.82	9.83	7.97	11.80	9.84	9.83	9.19	10.51
Bretagne	Other genotypes	Organised	2023	(39,49]	406	5.67	5.73	5.67	3.45	8.37	5.76	5.75	4.95	6.63
Centre-Val de Loire	Other genotypes	Opportunistic	2023	(39,49]	1,493	10.31	9.23	9.24	7.70	10.79	9.23	9.23	8.82	9.64
Centre-Val de Loire	Other genotypes	Organised	2023	(39,49]	133	8.27	6.56	6.77	2.26	11.28	6.62	6.61	5.88	7.40
Corse	Other genotypes	Opportunistic	2023	(39,49]	1	0.00	9.77	0.00	0.00	100.00	11.01	10.74	6.05	17.73
Grand Est	Other genotypes	Opportunistic	2023	(39,49]	843	10.79	9.65	9.61	7.59	11.86	9.65	9.65	9.07	10.28
Grand Est	Other genotypes	Organised	2023	(39,49]	101	5.94	6.40	5.94	1.98	11.88	6.46	6.44	5.59	7.42
Hauts-de-France	Other genotypes	Opportunistic	2023	(39,49]	2,152	11.01	9.22	9.20	7.95	10.50	9.23	9.23	8.83	9.62
Hauts-de-France	Other genotypes	Organised	2023	(39,49]	100	6.00	6.39	6.00	2.00	11.03	6.40	6.39	5.62	7.27
Normandie	Other genotypes	Opportunistic	2023	(39,49]	1,088	8.82	10.08	10.11	8.27	11.95	10.11	10.10	9.58	10.63
Normandie	Other genotypes	Organised	2023	(39,49]	42	7.14	6.78	7.14	0.00	14.29	6.84	6.84	5.98	7.78
Nouvelle-Aquitaine	Other genotypes	Opportunistic	2023	(39,49]	2,209	10.59	10.07	10.05	8.78	11.41	10.06	10.06	9.65	10.51
Nouvelle-Aquitaine	Other genotypes	Organised	2023	(39,49]	441	9.30	7.34	7.26	4.76	10.20	7.33	7.31	6.58	8.17
Occitanie	Other genotypes	Opportunistic	2023	(39,49]	353	7.08	10.96	11.05	7.93	14.45	10.96	10.94	10.18	11.82
Occitanie	Other genotypes	Organised	2023	(39,49]	51	5.88	7.97	7.84	1.96	15.69	8.05	8.05	6.98	9.20
Pays de la Loire	Other genotypes	Opportunistic	2023	(39,49]	299	7.69	8.86	8.70	5.69	12.37	8.86	8.85	8.14	9.68
Pays de la Loire	Other genotypes	Organised	2023	(39,49]	131	6.11	5.82	5.34	2.29	9.92	5.74	5.72	4.98	6.57
Provence-Alpes-Côte d'Azur	Other genotypes	Opportunistic	2023	(39,49]	1,551	10.25	10.55	10.51	8.96	12.19	10.55	10.55	10.08	11.04
Provence-Alpes-Côte d'Azur	Other genotypes	Organised	2023	(39,49]	139	10.07	8.34	7.91	3.60	13.67	8.37	8.33	7.22	9.76
Île-de-France	Other genotypes	Opportunistic	2023	(39,49]	3,580	9.27	10.56	10.56	9.47	11.65	10.56	10.56	10.20	10.93
Île-de-France	Other genotypes	Organised	2023	(39,49]	364	7.97	7.51	7.42	4.67	10.44	7.53	7.50	6.73	8.45
Auvergne-Rhône-Alpes	Other genotypes	Opportunistic	2023	(49,59]	45	6.67	10.51	11.11	2.22	20.00	10.57	10.56	8.98	12.29
Auvergne-Rhône-Alpes	Other genotypes	Organised	2023	(49,59]	9	22.22	7.79	11.11	0.00	33.33	7.88	7.84	6.29	9.71
Bourgogne-Franche-Comté	Other genotypes	Opportunistic	2023	(49,59]	104	10.58	8.34	7.69	3.85	13.46	8.31	8.30	7.52	9.16
Bourgogne-Franche-Comté	Other genotypes	Organised	2023	(49,59]	54	5.56	6.07	5.56	0.00	12.96	6.03	5.99	4.96	7.27
Bretagne	Other genotypes	Opportunistic	2023	(49,59]	750	10.13	8.37	8.40	6.40	10.53	8.37	8.37	7.80	8.96
Bretagne	Other genotypes	Organised	2023	(49,59]	389	5.40	5.36	5.40	3.08	7.97	5.33	5.31	4.59	6.17
Centre-Val de Loire	Other genotypes	Opportunistic	2023	(49,59]	1,356	6.78	7.90	7.89	6.42	9.37	7.92	7.91	7.56	8.30
Centre-Val de Loire	Other genotypes	Organised	2023	(49,59]	169	2.37	6.00	5.92	2.37	10.06	6.03	6.03	5.37	6.76
Grand Est	Other genotypes	Opportunistic	2023	(49,59]	693	8.23	8.14	8.08	6.20	10.25	8.14	8.13	7.62	8.69
Grand Est	Other genotypes	Organised	2023	(49,59]	129	4.65	5.84	5.43	2.33	10.08	5.81	5.80	5.01	6.69

Table S8 Observed HR HPV cervical infection prevalence, posterior predictive HR HPV cervical infection prevalence, and posterior expected HR HPV cervical infection prevalence, stratified by various dimensions. ***(continued)***

							Posterior distribution							
							Predictive prevalence				Expected prevalence			
									ETI95%				ETI95%	
Region	Genotypes	Screening pathway	Year	Age	Number of tests	Observed	Average	Median	LB	UB	Average	Median	LB	UB
Hauts-de-France	Other genotypes	Opportunistic	2023	(49,59]	1,553	9.08	7.80	7.79	6.44	9.21	7.80	7.79	7.45	8.16
Hauts-de-France	Other genotypes	Organised	2023	(49,59]	118	2.54	5.86	5.93	1.69	11.02	5.92	5.91	5.19	6.71
Normandie	Other genotypes	Opportunistic	2023	(49,59]	825	8.24	8.48	8.48	6.55	10.43	8.50	8.49	8.05	8.95
Normandie	Other genotypes	Organised	2023	(49,59]	35	2.86	6.25	5.71	0.00	14.29	6.24	6.24	5.45	7.07
Nouvelle-Aquitaine	Other genotypes	Opportunistic	2023	(49,59]	1,712	8.88	8.57	8.59	7.24	9.93	8.56	8.56	8.20	8.95
Nouvelle-Aquitaine	Other genotypes	Organised	2023	(49,59]	456	7.02	6.77	6.80	4.60	9.21	6.75	6.74	6.07	7.50
Occitanie	Other genotypes	Opportunistic	2023	(49,59]	307	9.12	9.31	9.12	6.19	12.70	9.28	9.26	8.61	10.02
Occitanie	Other genotypes	Organised	2023	(49,59]	60	6.67	7.24	6.67	1.67	15.00	7.28	7.27	6.33	8.35
Pays de la Loire	Other genotypes	Opportunistic	2023	(49,59]	274	7.30	7.49	7.30	4.38	10.95	7.51	7.50	6.88	8.23
Pays de la Loire	Other genotypes	Organised	2023	(49,59]	129	6.98	5.47	5.43	2.33	10.08	5.50	5.49	4.78	6.31
Provence-Alpes-Côte d'Azur	Other genotypes	Opportunistic	2023	(49,59]	1,337	10.70	8.96	8.98	7.40	10.55	8.98	8.98	8.55	9.42
Provence-Alpes-Côte d'Azur	Other genotypes	Organised	2023	(49,59]	131	6.87	7.67	7.63	3.05	12.21	7.63	7.59	6.53	9.01
Île-de-France	Other genotypes	Opportunistic	2023	(49,59]	2,833	8.08	8.86	8.86	7.80	9.99	8.86	8.86	8.54	9.20
Île-de-France	Other genotypes	Organised	2023	(49,59]	313	5.11	6.85	6.71	4.15	9.90	6.83	6.81	6.07	7.65
Auvergne-Rhône-Alpes	Other genotypes	Opportunistic	2023	(59,66]	28	3.57	9.72	10.71	0.00	21.43	9.58	9.57	8.18	11.10
Auvergne-Rhône-Alpes	Other genotypes	Organised	2023	(59,66]	4	0.00	7.30	0.00	0.00	50.00	7.45	7.39	5.90	9.28
Bourgogne-Franche-Comté	Other genotypes	Opportunistic	2023	(59,66]	60	15.00	7.50	6.67	1.67	15.00	7.46	7.45	6.68	8.31
Bourgogne-Franche-Comté	Other genotypes	Organised	2023	(59,66]	32	6.25	5.80	6.25	0.00	15.62	5.85	5.83	4.76	7.08
Bretagne	Other genotypes	Opportunistic	2023	(59,66]	388	7.99	7.25	7.22	4.64	9.79	7.25	7.25	6.72	7.82
Bretagne	Other genotypes	Organised	2023	(59,66]	337	4.45	5.13	5.04	2.67	7.72	5.12	5.10	4.30	6.07
Centre-Val de Loire	Other genotypes	Opportunistic	2023	(59,66]	629	6.52	6.91	6.84	4.93	9.06	6.91	6.91	6.52	7.29
Centre-Val de Loire	Other genotypes	Organised	2023	(59,66]	120	5.00	5.67	5.83	1.67	10.00	5.64	5.63	4.88	6.48
Corse	Other genotypes	Opportunistic	2023	(59,66]	3	0.00	8.02	0.00	0.00	33.33	7.75	7.67	5.63	10.49
Grand Est	Other genotypes	Opportunistic	2023	(59,66]	384	5.99	7.31	7.29	4.69	10.16	7.24	7.24	6.73	7.77
Grand Est	Other genotypes	Organised	2023	(59,66]	89	4.49	5.68	5.62	1.12	11.24	5.74	5.73	4.81	6.75
Hauts-de-France	Other genotypes	Opportunistic	2023	(59,66]	840	8.33	6.99	6.90	5.36	8.81	6.99	6.99	6.61	7.38
Hauts-de-France	Other genotypes	Organised	2023	(59,66]	116	9.48	5.74	6.03	1.72	10.34	5.85	5.83	5.04	6.78
Normandie	Other genotypes	Opportunistic	2023	(59,66]	381	8.14	7.45	7.35	4.99	10.24	7.45	7.44	7.01	7.92
Normandie	Other genotypes	Organised	2023	(59,66]	71	5.63	6.04	5.63	1.41	12.68	6.04	6.02	5.17	7.00
Nouvelle-Aquitaine	Other genotypes	Opportunistic	2023	(59,66]	859	8.27	7.72	7.68	5.94	9.55	7.70	7.70	7.29	8.13
Nouvelle-Aquitaine	Other genotypes	Organised	2023	(59,66]	308	5.19	6.48	6.49	3.90	9.74	6.53	6.52	5.73	7.43
Occitanie	Other genotypes	Opportunistic	2023	(59,66]	168	8.93	8.18	8.33	4.17	12.50	8.23	8.22	7.61	8.94
Occitanie	Other genotypes	Organised	2023	(59,66]	52	9.62	7.04	5.77	1.92	15.38	7.13	7.10	6.03	8.36
Pays de la Loire	Other genotypes	Opportunistic	2023	(59,66]	126	10.32	6.98	7.14	3.17	11.13	7.05	7.04	6.37	7.79
Pays de la Loire	Other genotypes	Organised	2023	(59,66]	97	4.12	5.22	5.15	1.03	9.30	5.22	5.20	4.44	6.10
Provence-Alpes-Côte d'Azur	Other genotypes	Opportunistic	2023	(59,66]	667	9.15	8.00	7.95	6.00	10.19	8.01	8.01	7.57	8.46
Provence-Alpes-Côte d'Azur	Other genotypes	Organised	2023	(59,66]	87	8.05	7.21	6.90	2.30	12.64	7.32	7.28	6.21	8.65
Île-de-France	Other genotypes	Opportunistic	2023	(59,66]	1,258	10.10	7.84	7.79	6.44	9.38	7.84	7.84	7.48	8.22

Île-de-France	Other genotypes	Organised	2023	(59,66]	143	11.89	6.54	6.29	2.80	10.49	6.58	6.56	5.74	7.53

S8 Systematic difference in expected HR HPV cervical infection prevalence between opportunistic and organised screening, among females aged 30, at the end of November 2023

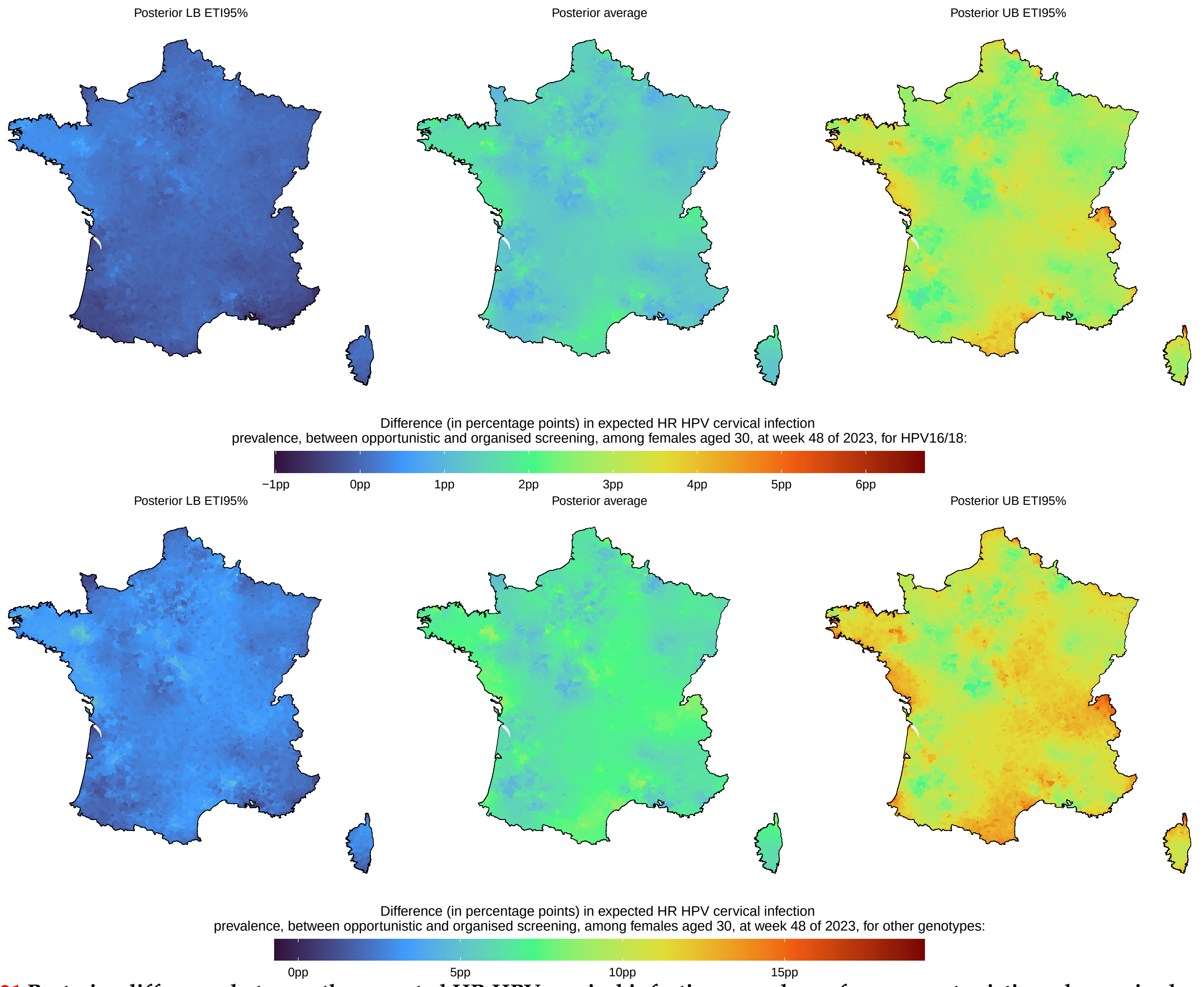

Figure S21 Posterior difference between the expected HR HPV cervical infection prevalence from opportunistic and organised screening, among females aged 30, at week 48 of 2023.

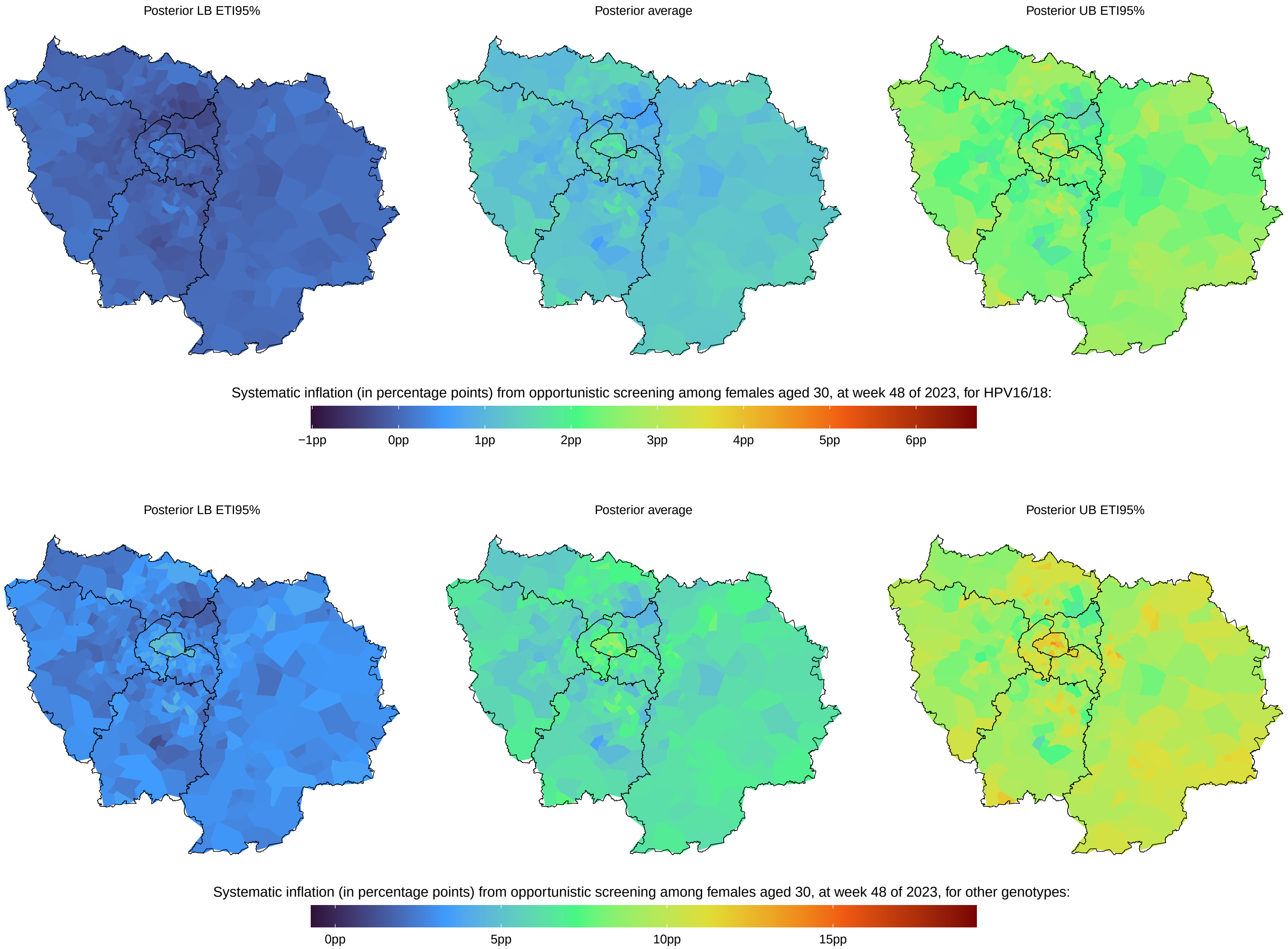

Figure S22 Posterior difference between the expected HR HPV between screening pathways, among females aged 30, at week 48 of 2023, in Paris region.

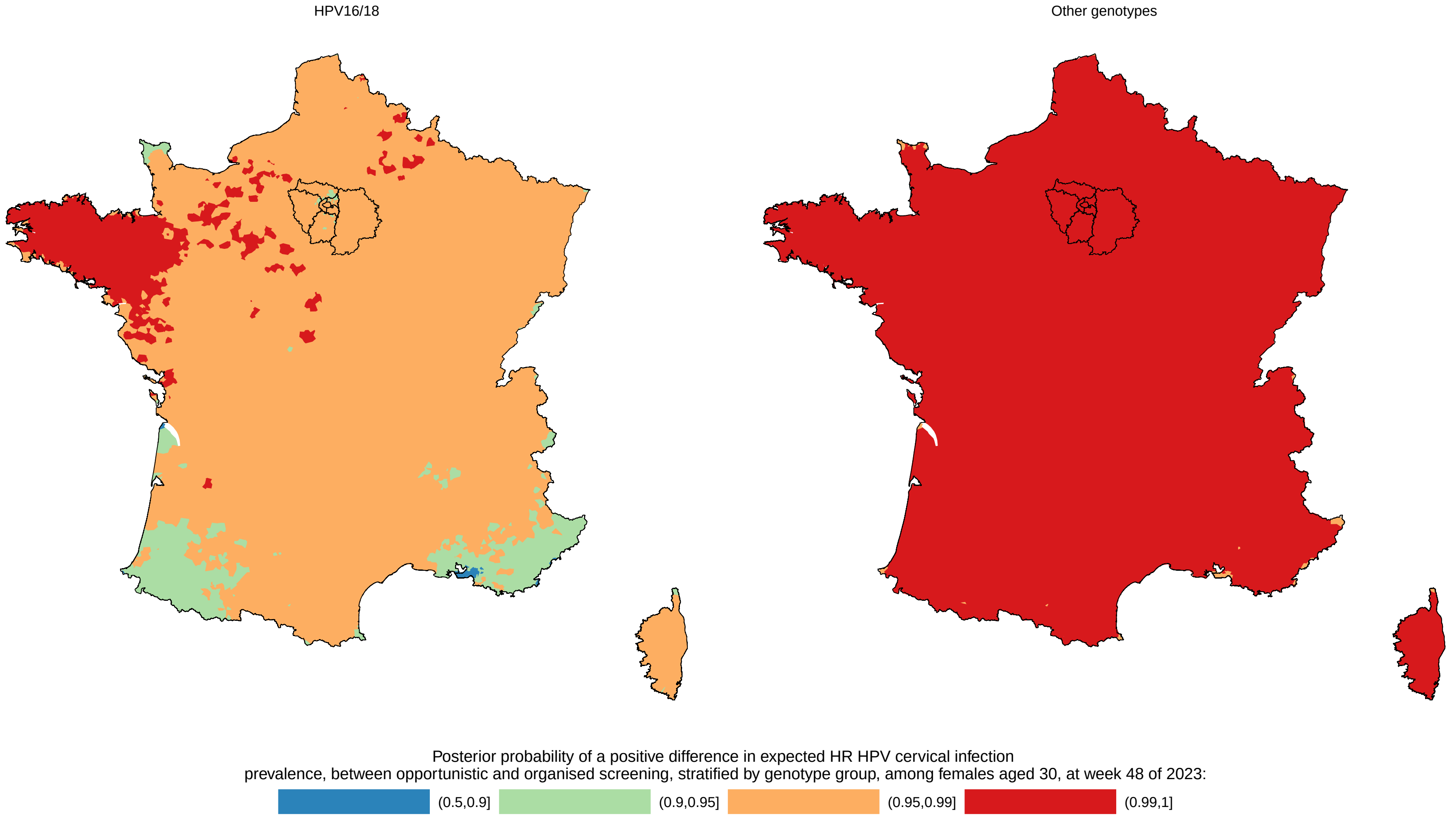

Figure S23 Posterior probability of a positive difference in expected HR HPV between opportunistic and organised screening, stratified by genotype group, among females aged 30, at week 48 of 2023.

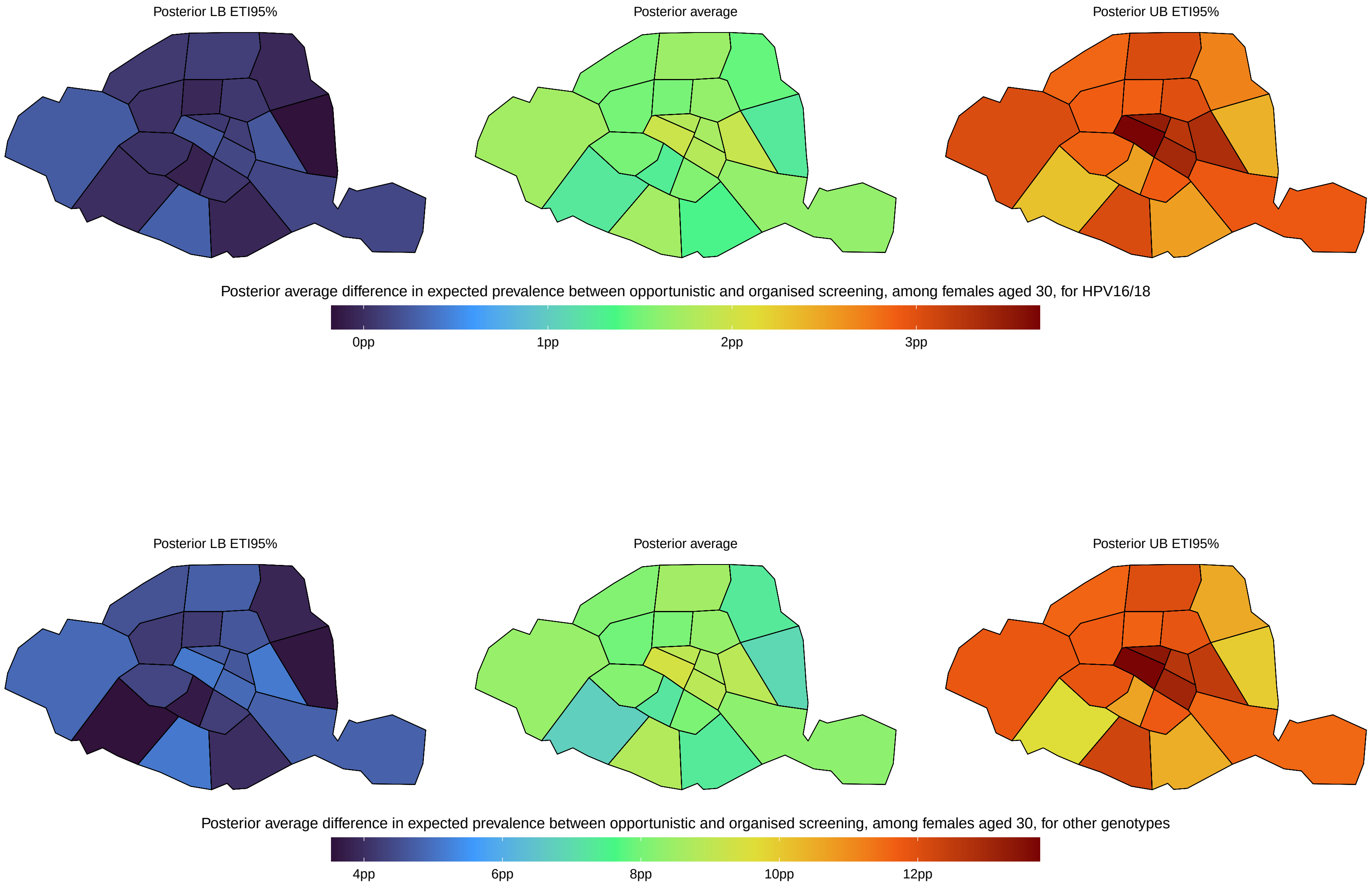

Figure S24 Posterior systematic inflation in HR HPV cervical infection prevalence under opportunistic screening, compared with organised screening, in each of the Paris arrondissements, among females aged 30, at week 48 of 2023.

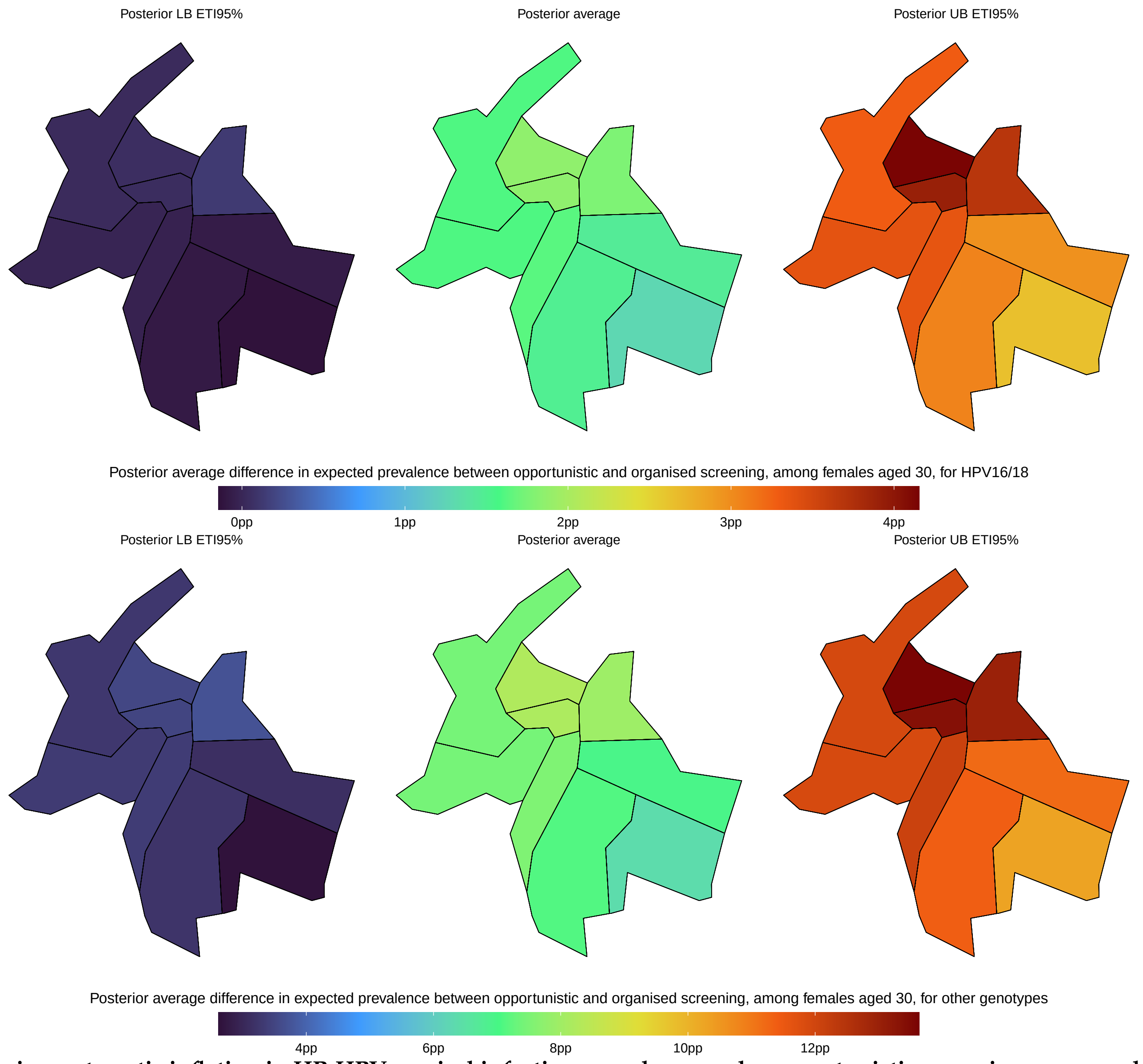

Figure S25 Posterior systematic inflation in HR HPV cervical infection prevalence under opportunistic screening, compared with organised screening, in each of the Lyon arrondissements, among females aged 30, at week 48 of 2023.

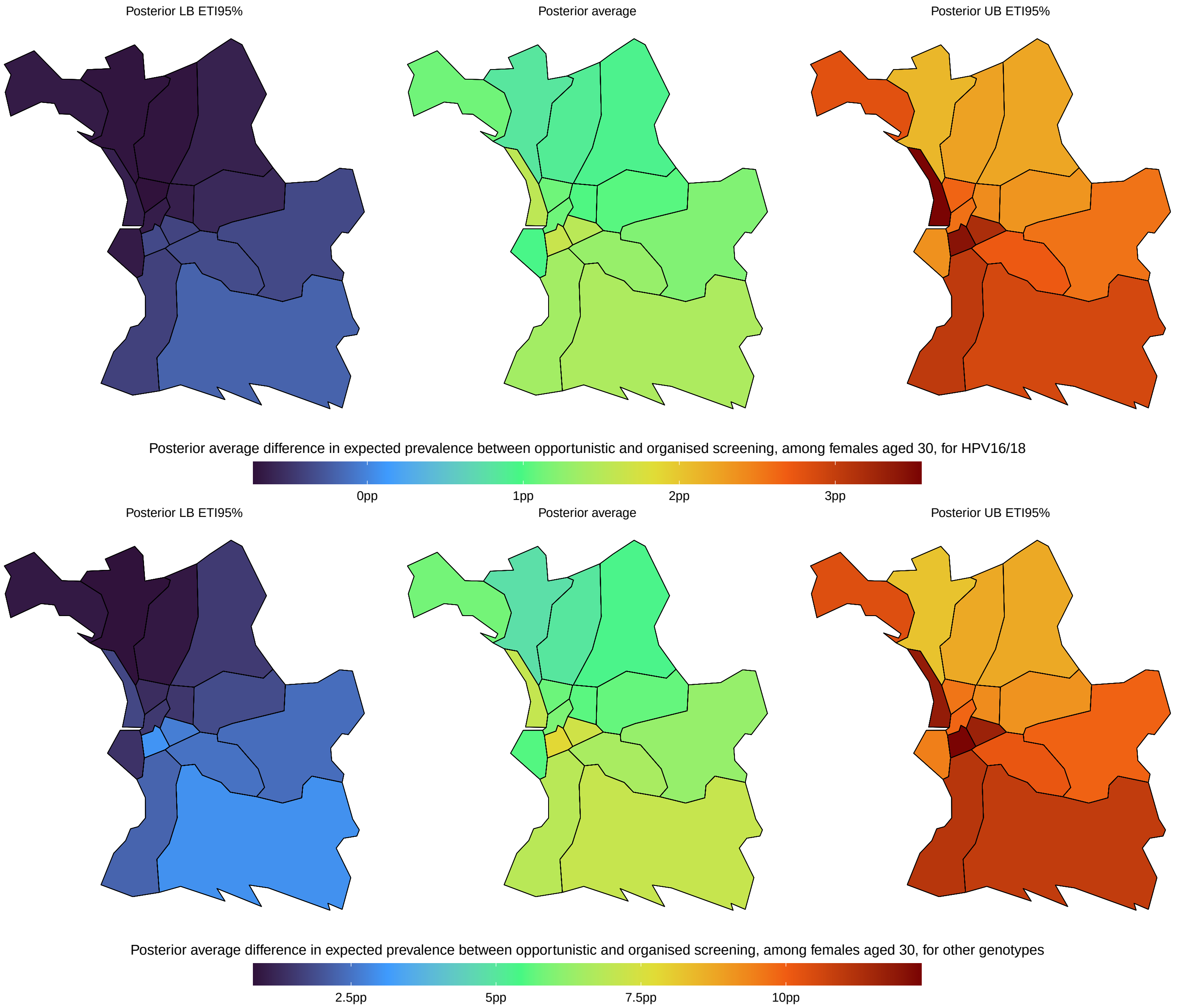

Figure S26 **Posterior systematic inflation in HR HPV cervical infection prevalence under opportunistic screening, compared with organised screening, in each of the Marseille arrondissements, among females aged 30, at week 48 of 2023.**

S9 Expected HR HPV cervical infection prevalence, among females aged 30, at week 48 of 2023, under organised screening

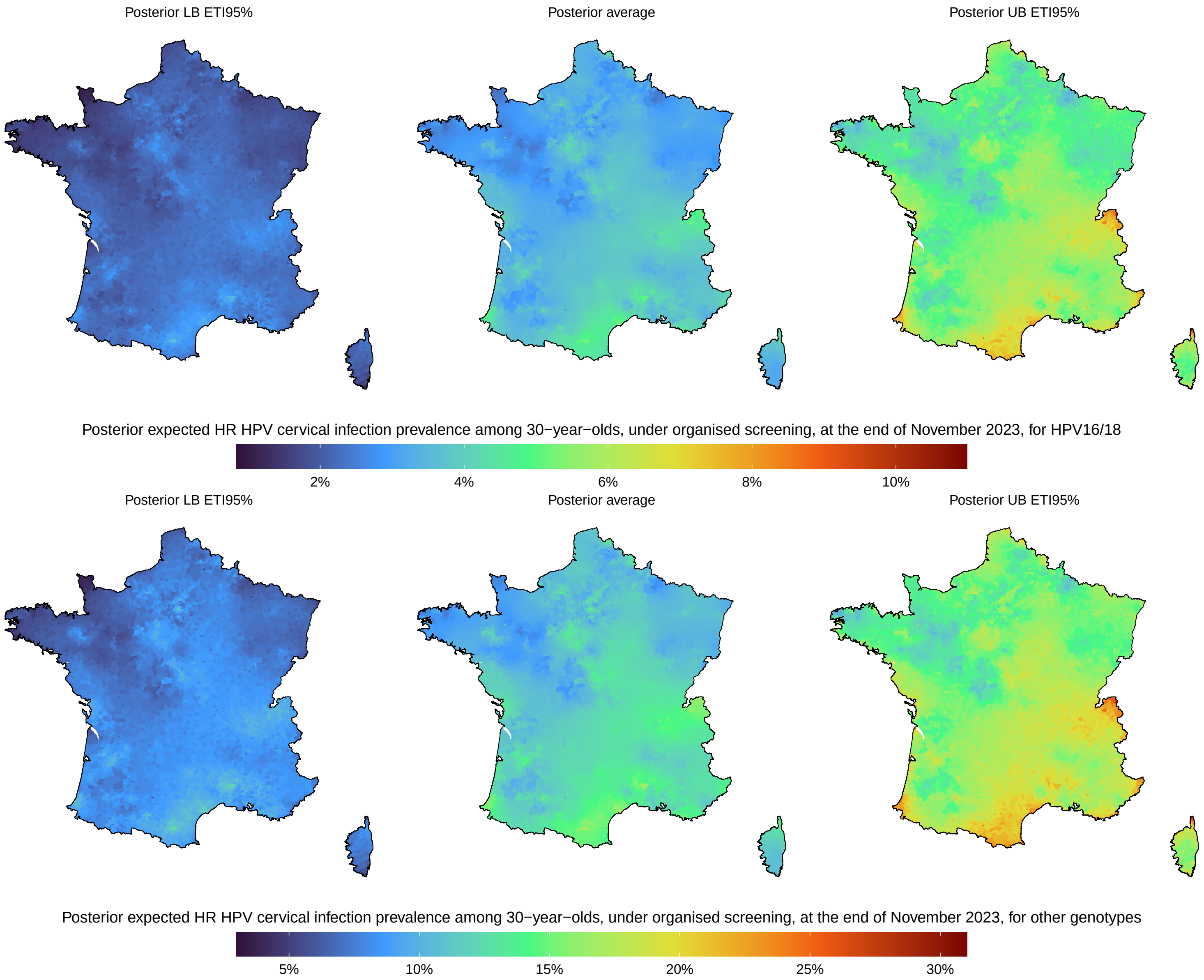

Figure S27 Posterior HR HPV cervical infection prevalence in metropolitan France, among females aged 30, at week 48 of 2023, correcting for the systematic inflation associated with opportunistic screening, stratified by genotype group.

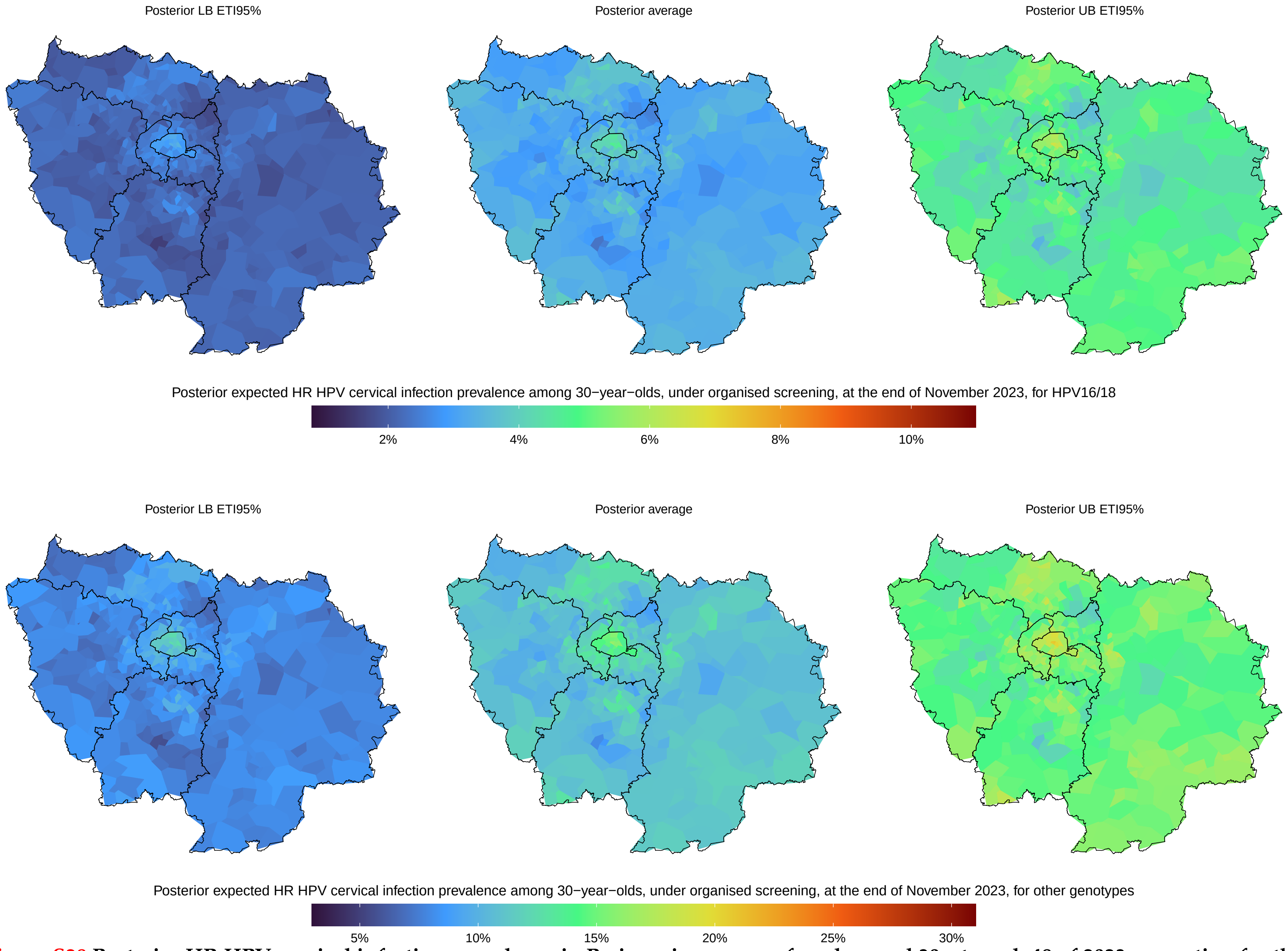

Figure S28 Posterior HR HPV cervical infection prevalence in Paris region, among females aged 30, at week 48 of 2023, correcting for the systematic inflation associated with opportunistic screening, stratified by genotype group.

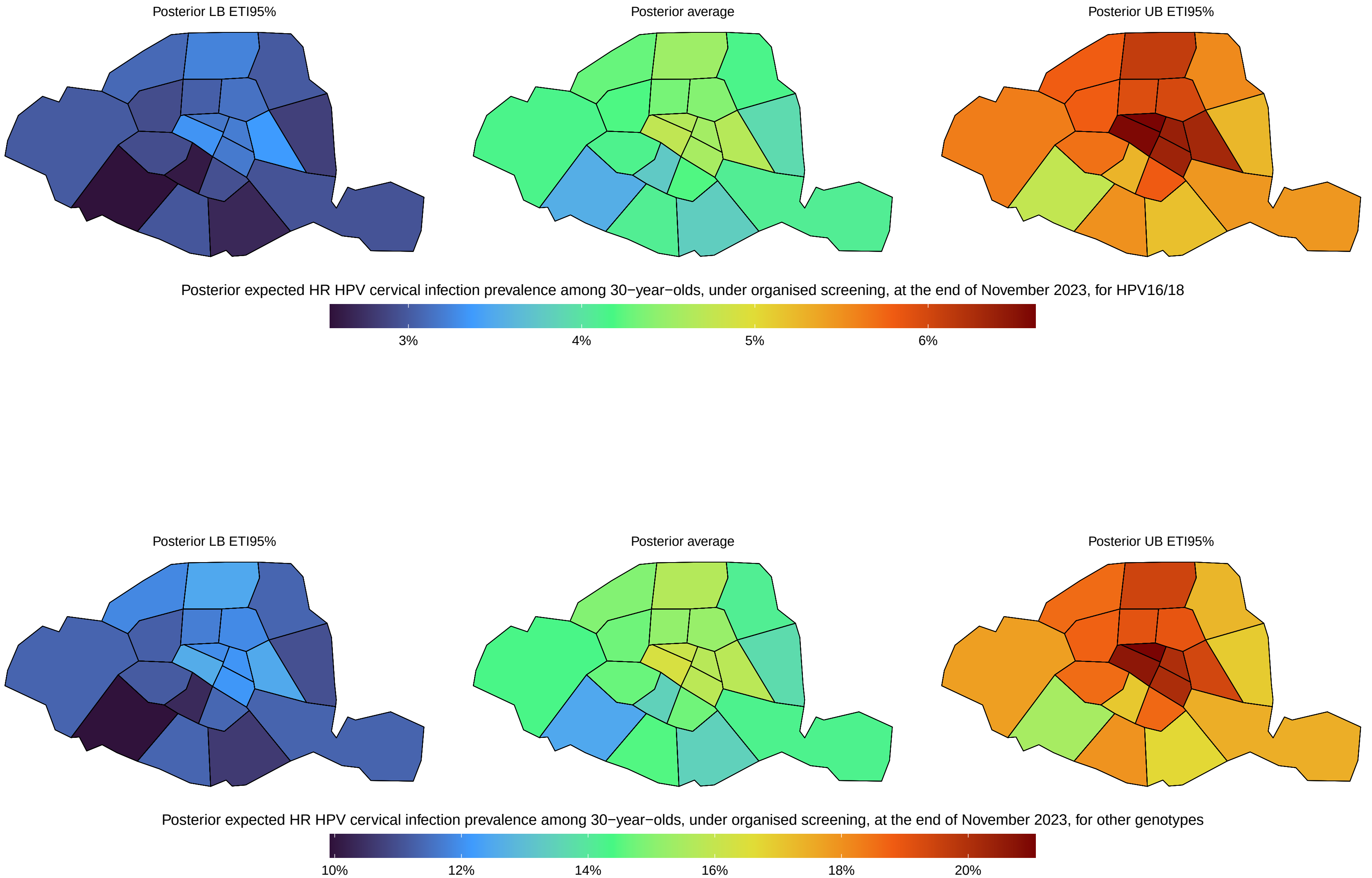

Figure S29 **Posterior HR HPV cervical infection prevalence in each of the Paris arrondissements, among females aged 30, at week 48 of 2023, correcting for the systematic inflation associated with opportunistic screening, stratified by genotype group.**

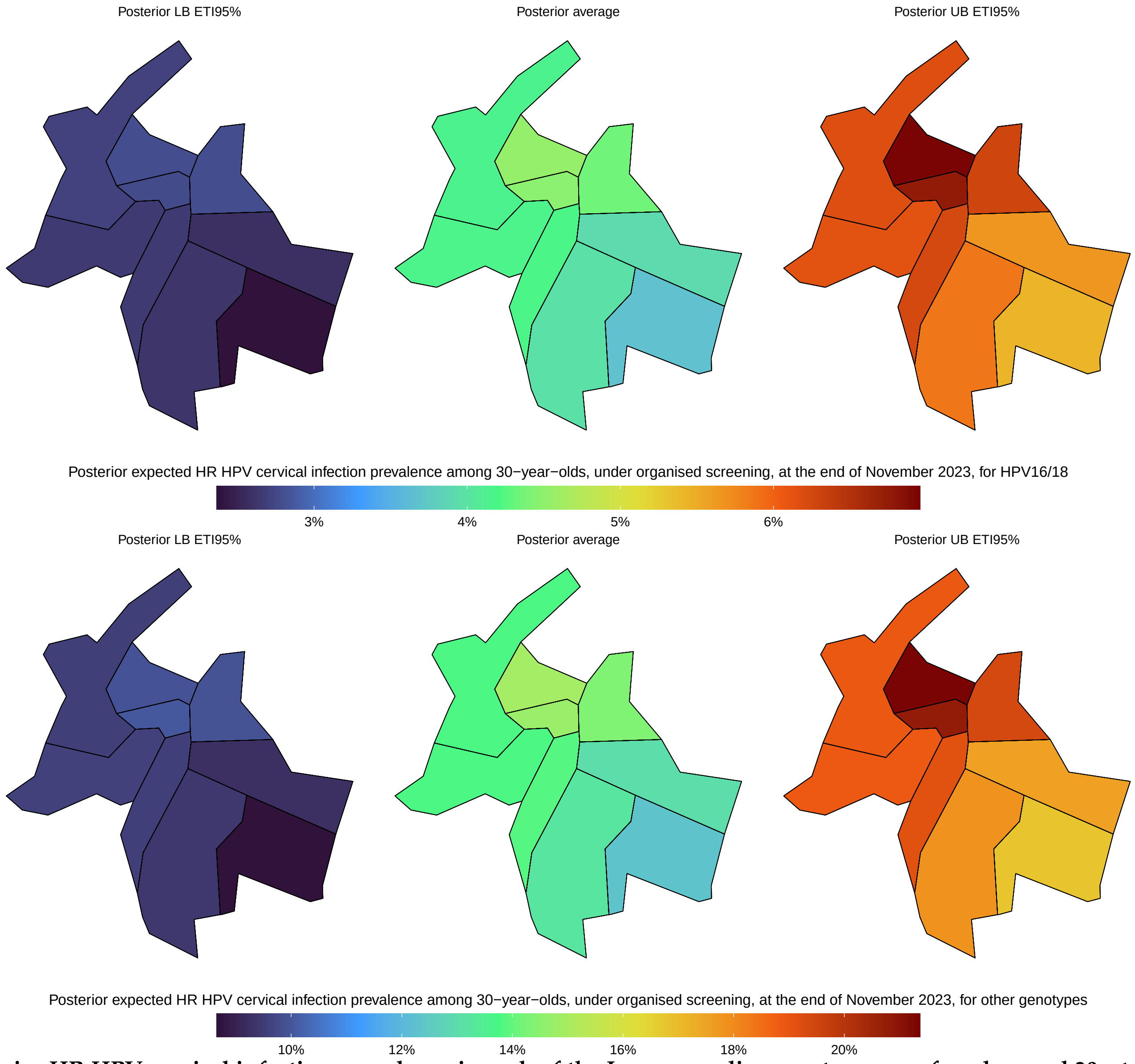

Figure S30 Posterior HR HPV cervical infection prevalence in each of the Lyon arrondissements, among females aged 30, at week 48 of 2023, correcting for the systematic inflation associated with opportunistic screening, stratified by genotype group.

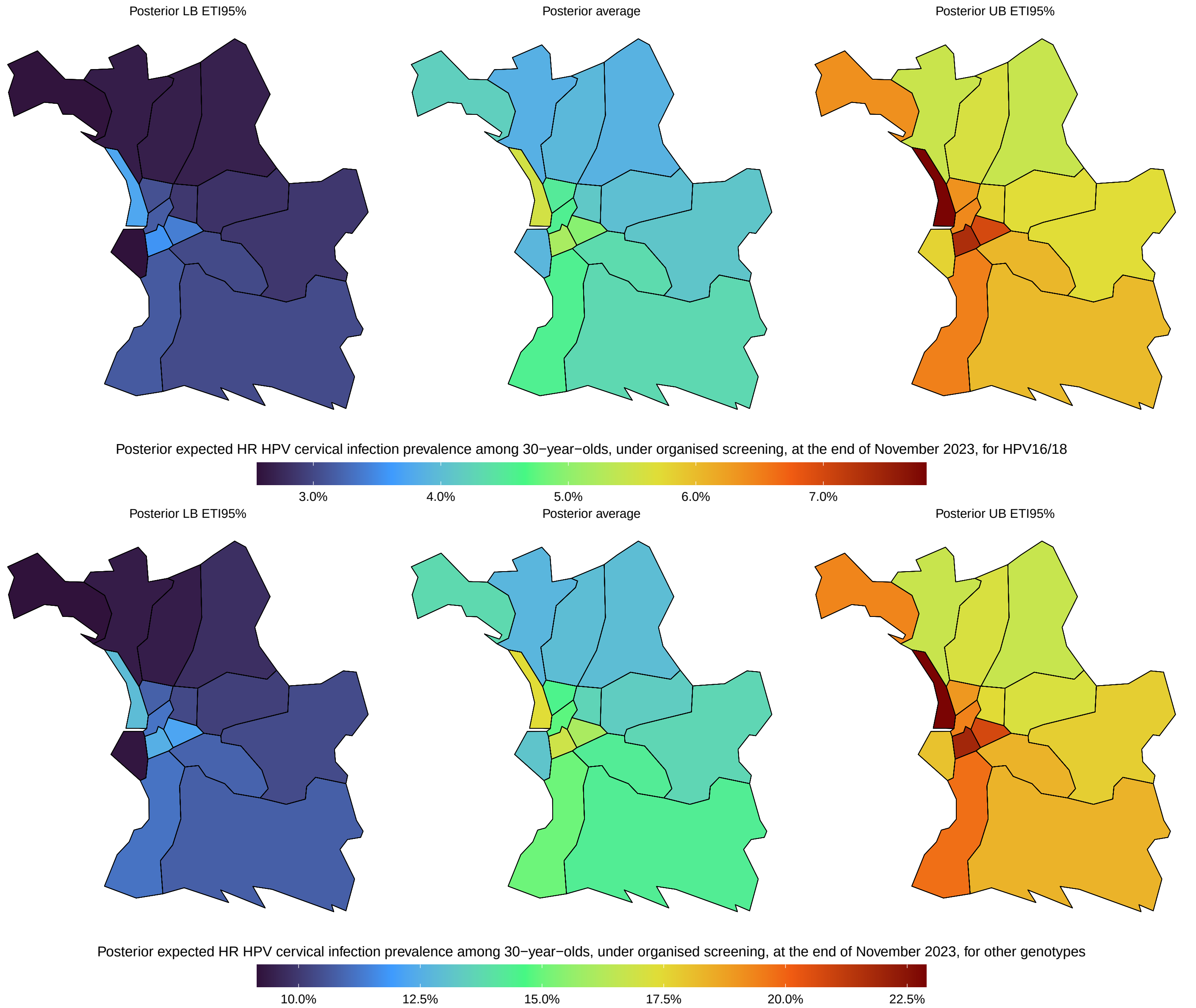

Figure S31 Posterior HR HPV cervical infection prevalence in each of the Marseille arrondissements, among females aged 30, at week 48 of 2023, correcting for the systematic inflation associated with opportunistic screening, stratified by genotype group.

S10 Maps of the posterior average expected infection prevalence stratified by age, at week 48 of 2023, under organised screening

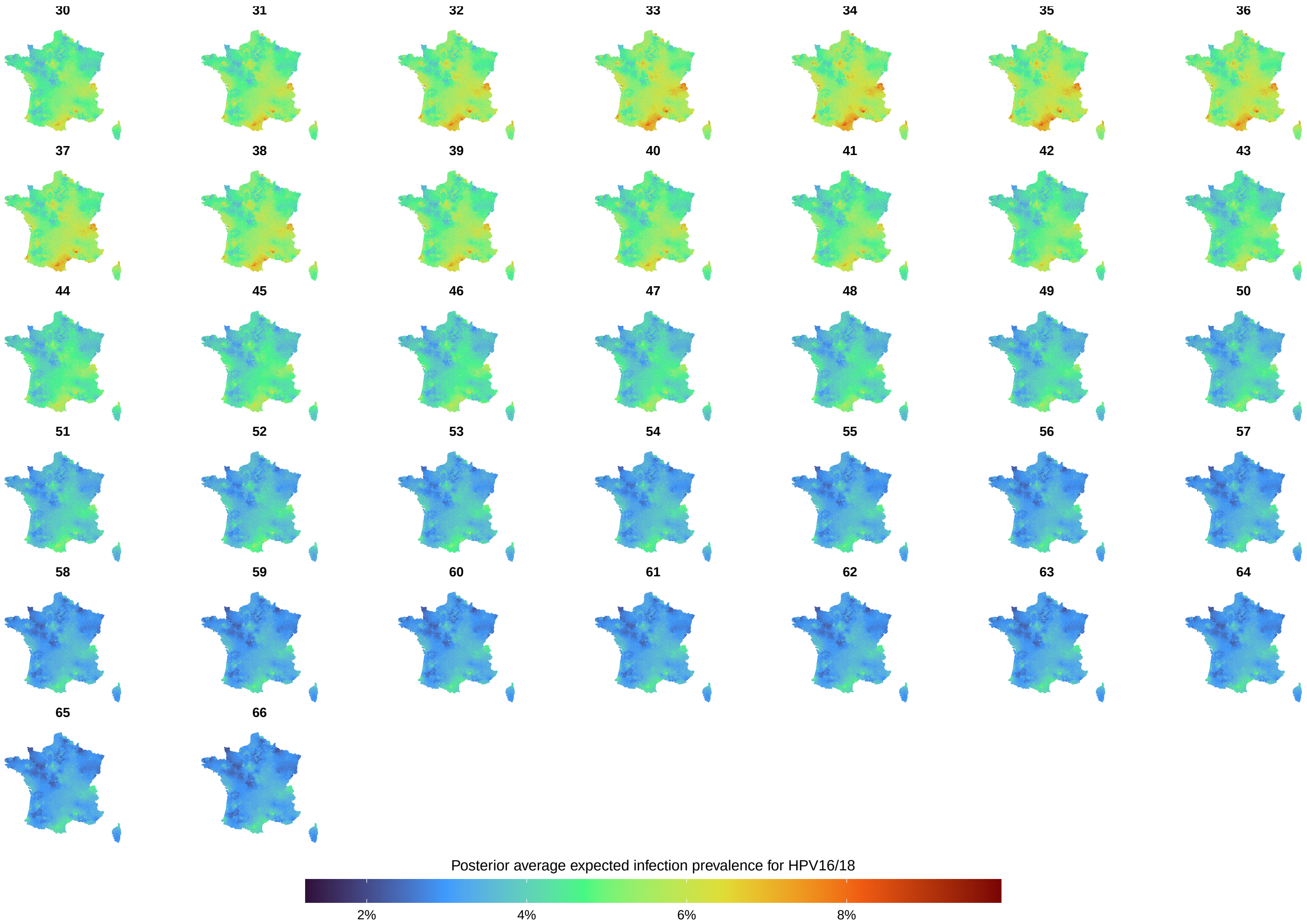

Figure S32 **Posterior average expected infection prevalence for HPV16/18 under organised screening, across metropolitan France, stratified by age.**

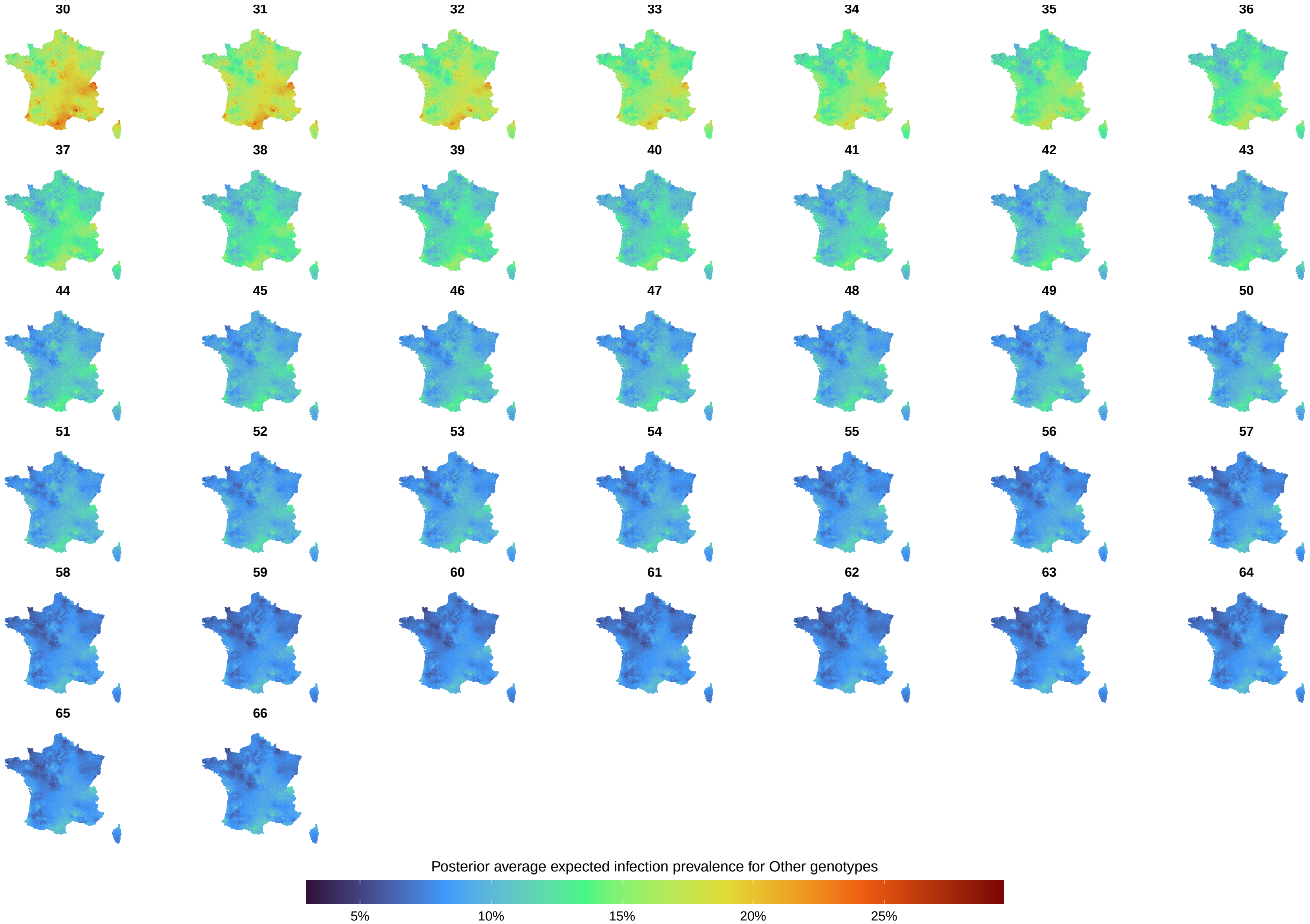

Figure S33 Posterior average expected infection prevalence for Other genotypes, under organised screening, across metropolitan France, stratified by age.

S11 Figure 3A and B and 4A and B in table format

Table S9 Posterior expected HR HPV cervical infection prevalence (in %) in major French cities, stratified by type of test, city, and age. Table counterpart to Figure 3A.

				Posterior distribution							
					Percentiles						
Virus	Test	City	Age	Average	0.01	0.025	0.1	0.5	0.9	0.975	0.99
HPV16/18	Organised	Nice	30	3.82	1.77	2.01	2.49	3.66	5.35	6.57	7.23
HPV16/18	Organised	Nice	31	4.01	1.88	2.12	2.63	3.84	5.60	6.84	7.50
HPV16/18	Organised	Nice	32	4.18	1.99	2.23	2.75	4.01	5.80	7.05	7.82
HPV16/18	Organised	Nice	33	4.31	2.06	2.32	2.83	4.13	5.97	7.24	8.00
HPV16/18	Organised	Nice	34	4.35	2.10	2.35	2.87	4.17	6.04	7.33	8.10
HPV16/18	Organised	Nice	35	4.31	2.10	2.33	2.86	4.13	5.98	7.24	8.04
HPV16/18	Organised	Nice	36	4.23	2.06	2.29	2.81	4.05	5.86	7.09	7.77
HPV16/18	Organised	Nice	37	4.12	2.03	2.22	2.74	3.94	5.74	6.88	7.61
HPV16/18	Organised	Nice	38	4.01	2.00	2.15	2.66	3.84	5.59	6.71	7.38
HPV16/18	Organised	Nice	39	3.91	1.96	2.11	2.59	3.75	5.46	6.52	7.27
HPV16/18	Organised	Nice	40	3.81	1.89	2.06	2.53	3.65	5.31	6.38	7.12
HPV16/18	Organised	Nice	41	3.72	1.84	2.00	2.47	3.56	5.19	6.20	6.93
HPV16/18	Organised	Nice	42	3.62	1.80	1.96	2.39	3.46	5.03	6.04	6.75
HPV16/18	Organised	Nice	43	3.52	1.75	1.92	2.33	3.38	4.89	5.87	6.54
HPV16/18	Organised	Nice	44	3.45	1.72	1.88	2.27	3.30	4.79	5.77	6.39
HPV16/18	Organised	Nice	45	3.38	1.69	1.84	2.24	3.25	4.71	5.68	6.27
HPV16/18	Organised	Nice	46	3.31	1.64	1.79	2.18	3.17	4.62	5.58	6.14
HPV16/18	Organised	Nice	47	3.24	1.59	1.75	2.14	3.12	4.50	5.45	6.06
HPV16/18	Organised	Nice	48	3.18	1.56	1.72	2.10	3.05	4.43	5.37	5.99
HPV16/18	Organised	Nice	49	3.12	1.53	1.68	2.06	2.99	4.33	5.27	5.89
HPV16/18	Organised	Nice	50	3.06	1.49	1.65	2.02	2.93	4.26	5.18	5.74
HPV16/18	Organised	Nice	51	3.00	1.47	1.63	1.98	2.88	4.19	5.09	5.67
HPV16/18	Organised	Nice	52	2.95	1.44	1.61	1.95	2.83	4.10	5.01	5.58
HPV16/18	Organised	Nice	53	2.89	1.39	1.58	1.91	2.77	4.03	4.91	5.47
HPV16/18	Organised	Nice	54	2.83	1.36	1.53	1.86	2.71	3.96	4.84	5.33
HPV16/18	Organised	Nice	55	2.78	1.33	1.51	1.83	2.66	3.91	4.76	5.28
HPV16/18	Organised	Nice	56	2.75	1.31	1.49	1.79	2.63	3.87	4.65	5.23

Table S9 Posterior expected HR HPV cervical infection prevalence (in %) in major French cities, stratified by type of test, city, and age. Table counterpart to Figure 3A. *(continued)*

				Posterior distribution							
					Percentiles						
Virus	Test	City	Age	Average	0.01	0.025	0.1	0.5	0.9	0.975	0.99
HPV16/18	Organised	Nice	57	2.73	1.29	1.47	1.78	2.61	3.84	4.62	5.20
HPV16/18	Organised	Nice	58	2.72	1.30	1.47	1.77	2.62	3.84	4.65	5.23
HPV16/18	Organised	Nice	59	2.73	1.28	1.46	1.76	2.62	3.85	4.64	5.24
HPV16/18	Organised	Nice	60	2.74	1.30	1.46	1.77	2.63	3.87	4.68	5.27
HPV16/18	Organised	Nice	61	2.76	1.31	1.47	1.78	2.64	3.90	4.72	5.33
HPV16/18	Organised	Nice	62	2.77	1.29	1.47	1.78	2.66	3.92	4.74	5.36
HPV16/18	Organised	Nice	63	2.78	1.30	1.47	1.78	2.66	3.95	4.77	5.35
HPV16/18	Organised	Nice	64	2.78	1.29	1.45	1.77	2.66	3.93	4.81	5.27
HPV16/18	Organised	Nice	65	2.77	1.28	1.45	1.75	2.65	3.90	4.84	5.29
HPV16/18	Organised	Nice	66	2.75	1.27	1.43	1.73	2.63	3.91	4.89	5.32
HPV16/18	Organised	Marseille	30	4.56	2.93	3.15	3.54	4.48	5.66	6.42	6.84
HPV16/18	Organised	Marseille	31	4.79	3.13	3.36	3.75	4.72	5.91	6.66	7.16
HPV16/18	Organised	Marseille	32	4.99	3.27	3.51	3.94	4.93	6.12	6.85	7.29
HPV16/18	Organised	Marseille	33	5.14	3.42	3.66	4.08	5.09	6.33	7.04	7.50
HPV16/18	Organised	Marseille	34	5.20	3.48	3.70	4.14	5.14	6.38	7.10	7.53
HPV16/18	Organised	Marseille	35	5.15	3.49	3.69	4.10	5.09	6.31	7.04	7.43
HPV16/18	Organised	Marseille	36	5.05	3.42	3.63	4.03	5.00	6.18	6.90	7.31
HPV16/18	Organised	Marseille	37	4.92	3.36	3.55	3.94	4.87	6.00	6.72	7.06
HPV16/18	Organised	Marseille	38	4.80	3.27	3.45	3.83	4.74	5.85	6.54	6.91
HPV16/18	Organised	Marseille	39	4.68	3.22	3.39	3.74	4.62	5.71	6.40	6.74
HPV16/18	Organised	Marseille	40	4.56	3.14	3.31	3.65	4.50	5.57	6.22	6.61
HPV16/18	Organised	Marseille	41	4.45	3.05	3.22	3.55	4.39	5.42	6.07	6.40
HPV16/18	Organised	Marseille	42	4.33	2.97	3.13	3.45	4.26	5.28	5.88	6.28
HPV16/18	Organised	Marseille	43	4.22	2.87	3.04	3.37	4.16	5.15	5.77	6.14
HPV16/18	Organised	Marseille	44	4.12	2.81	2.97	3.29	4.06	5.02	5.68	6.02
HPV16/18	Organised	Marseille	45	4.04	2.74	2.92	3.22	3.98	4.94	5.57	5.91
HPV16/18	Organised	Marseille	46	3.96	2.68	2.85	3.15	3.90	4.84	5.44	5.79
HPV16/18	Organised	Marseille	47	3.88	2.62	2.79	3.08	3.82	4.74	5.34	5.66
HPV16/18	Organised	Marseille	48	3.80	2.56	2.75	3.02	3.74	4.65	5.23	5.56

Table S9 Posterior expected HR HPV cervical infection prevalence (in %) in major French cities, stratified by type of test, city, and age. Table counterpart to Figure 3A. *(continued)*

				Posterior distribution							
					Percentiles						
Virus	Test	City	Age	Average	0.01	0.025	0.1	0.5	0.9	0.975	0.99
HPV16/18	Organised	Marseille	49	3.73	2.52	2.69	2.96	3.67	4.57	5.12	5.43
HPV16/18	Organised	Marseille	50	3.66	2.47	2.63	2.91	3.61	4.47	5.05	5.36
HPV16/18	Organised	Marseille	51	3.59	2.42	2.58	2.85	3.54	4.40	4.93	5.25
HPV16/18	Organised	Marseille	52	3.53	2.37	2.53	2.80	3.48	4.33	4.84	5.21
HPV16/18	Organised	Marseille	53	3.46	2.32	2.47	2.74	3.41	4.25	4.75	5.08
HPV16/18	Organised	Marseille	54	3.39	2.27	2.42	2.67	3.34	4.15	4.65	4.98
HPV16/18	Organised	Marseille	55	3.33	2.23	2.38	2.63	3.28	4.08	4.60	4.87
HPV16/18	Organised	Marseille	56	3.29	2.18	2.35	2.60	3.24	4.03	4.53	4.77
HPV16/18	Organised	Marseille	57	3.27	2.17	2.33	2.57	3.22	4.02	4.48	4.75
HPV16/18	Organised	Marseille	58	3.26	2.16	2.32	2.57	3.21	4.02	4.47	4.71
HPV16/18	Organised	Marseille	59	3.27	2.15	2.32	2.57	3.22	4.02	4.48	4.75
HPV16/18	Organised	Marseille	60	3.28	2.18	2.33	2.58	3.23	4.06	4.50	4.80
HPV16/18	Organised	Marseille	61	3.30	2.20	2.34	2.59	3.26	4.09	4.52	4.84
HPV16/18	Organised	Marseille	62	3.32	2.19	2.32	2.59	3.27	4.13	4.56	4.92
HPV16/18	Organised	Marseille	63	3.32	2.19	2.31	2.58	3.27	4.13	4.58	4.90
HPV16/18	Organised	Marseille	64	3.32	2.16	2.29	2.57	3.27	4.14	4.63	4.93
HPV16/18	Organised	Marseille	65	3.31	2.13	2.25	2.53	3.26	4.16	4.66	4.96
HPV16/18	Organised	Marseille	66	3.29	2.06	2.20	2.49	3.24	4.16	4.74	5.04
HPV16/18	Organised	Toulouse	30	3.81	2.15	2.31	2.70	3.72	5.06	5.97	6.59
HPV16/18	Organised	Toulouse	31	4.00	2.29	2.45	2.86	3.91	5.27	6.19	6.82
HPV16/18	Organised	Toulouse	32	4.17	2.39	2.57	3.00	4.08	5.49	6.41	7.05
HPV16/18	Organised	Toulouse	33	4.30	2.45	2.66	3.10	4.23	5.64	6.57	7.18
HPV16/18	Organised	Toulouse	34	4.35	2.51	2.70	3.13	4.27	5.68	6.56	7.21
HPV16/18	Organised	Toulouse	35	4.31	2.49	2.69	3.11	4.23	5.62	6.47	7.13
HPV16/18	Organised	Toulouse	36	4.22	2.43	2.65	3.03	4.14	5.51	6.36	7.05
HPV16/18	Organised	Toulouse	37	4.12	2.36	2.58	2.96	4.04	5.36	6.24	6.89
HPV16/18	Organised	Toulouse	38	4.01	2.32	2.51	2.90	3.93	5.24	6.09	6.69
HPV16/18	Organised	Toulouse	39	3.91	2.27	2.43	2.83	3.83	5.09	5.88	6.51
HPV16/18	Organised	Toulouse	40	3.81	2.21	2.37	2.75	3.72	4.95	5.70	6.28

Table S9 **Posterior expected HR HPV cervical infection prevalence (in %) in major French cities, stratified by type of test, city, and age. Table counterpart to Figure 3A.** *(continued)*

					Posterior distribution						
					Percentiles						
Virus	Test	City	Age	Average	0.01	0.025	0.1	0.5	0.9	0.975	0.99
HPV16/18	Organised	Toulouse	41	3.71	2.15	2.30	2.68	3.63	4.84	5.59	6.09
HPV16/18	Organised	Toulouse	42	3.61	2.09	2.23	2.61	3.53	4.71	5.42	5.91
HPV16/18	Organised	Toulouse	43	3.52	2.03	2.18	2.53	3.44	4.59	5.29	5.76
HPV16/18	Organised	Toulouse	44	3.44	1.98	2.14	2.47	3.37	4.49	5.19	5.61
HPV16/18	Organised	Toulouse	45	3.38	1.96	2.10	2.43	3.30	4.40	5.09	5.47
HPV16/18	Organised	Toulouse	46	3.30	1.91	2.04	2.38	3.23	4.32	4.99	5.35
HPV16/18	Organised	Toulouse	47	3.23	1.85	2.00	2.33	3.17	4.23	4.90	5.24
HPV16/18	Organised	Toulouse	48	3.17	1.82	1.96	2.27	3.10	4.14	4.80	5.15
HPV16/18	Organised	Toulouse	49	3.11	1.79	1.93	2.23	3.04	4.06	4.71	5.03
HPV16/18	Organised	Toulouse	50	3.05	1.75	1.89	2.18	2.99	3.99	4.63	4.96
HPV16/18	Organised	Toulouse	51	3.00	1.72	1.85	2.13	2.94	3.93	4.52	4.89
HPV16/18	Organised	Toulouse	52	2.94	1.70	1.82	2.09	2.89	3.86	4.44	4.85
HPV16/18	Organised	Toulouse	53	2.88	1.66	1.78	2.05	2.82	3.79	4.38	4.76
HPV16/18	Organised	Toulouse	54	2.83	1.62	1.74	2.01	2.77	3.70	4.31	4.68
HPV16/18	Organised	Toulouse	55	2.78	1.59	1.72	1.98	2.72	3.63	4.24	4.59
HPV16/18	Organised	Toulouse	56	2.74	1.57	1.70	1.95	2.68	3.59	4.20	4.56
HPV16/18	Organised	Toulouse	57	2.72	1.55	1.69	1.93	2.67	3.57	4.16	4.52
HPV16/18	Organised	Toulouse	58	2.72	1.54	1.66	1.93	2.65	3.58	4.12	4.50
HPV16/18	Organised	Toulouse	59	2.72	1.53	1.66	1.93	2.65	3.58	4.17	4.48
HPV16/18	Organised	Toulouse	60	2.74	1.54	1.67	1.93	2.67	3.61	4.19	4.51
HPV16/18	Organised	Toulouse	61	2.75	1.57	1.66	1.95	2.69	3.63	4.22	4.56
HPV16/18	Organised	Toulouse	62	2.77	1.57	1.67	1.95	2.70	3.64	4.25	4.62
HPV16/18	Organised	Toulouse	63	2.77	1.57	1.66	1.96	2.69	3.66	4.29	4.70
HPV16/18	Organised	Toulouse	64	2.77	1.54	1.65	1.94	2.69	3.65	4.30	4.74
HPV16/18	Organised	Toulouse	65	2.76	1.52	1.63	1.92	2.67	3.65	4.32	4.81
HPV16/18	Organised	Toulouse	66	2.75	1.46	1.60	1.89	2.65	3.66	4.39	4.86
HPV16/18	Organised	Bordeaux	30	4.08	2.62	2.75	3.14	4.02	5.10	5.80	6.15
HPV16/18	Organised	Bordeaux	31	4.29	2.80	2.90	3.31	4.22	5.32	6.02	6.39
HPV16/18	Organised	Bordeaux	32	4.47	2.91	3.06	3.47	4.41	5.55	6.22	6.58

Table S9 **Posterior expected HR HPV cervical infection prevalence (in %) in major French cities, stratified by type of test, city, and age. Table counterpart to Figure 3A.** ***(continued)***

Virus	Test	City	Age	Posterior distribution							
					Percentiles						
				Average	0.01	0.025	0.1	0.5	0.9	0.975	0.99
HPV16/18	Organised	Bordeaux	33	4.61	3.02	3.18	3.60	4.55	5.71	6.37	6.74
HPV16/18	Organised	Bordeaux	34	4.66	3.05	3.23	3.65	4.60	5.75	6.42	6.74
HPV16/18	Organised	Bordeaux	35	4.61	3.04	3.20	3.62	4.56	5.68	6.35	6.67
HPV16/18	Organised	Bordeaux	36	4.52	2.97	3.17	3.56	4.47	5.55	6.22	6.55
HPV16/18	Organised	Bordeaux	37	4.41	2.89	3.11	3.47	4.35	5.41	6.06	6.39
HPV16/18	Organised	Bordeaux	38	4.30	2.81	3.02	3.38	4.24	5.28	5.91	6.22
HPV16/18	Organised	Bordeaux	39	4.19	2.74	2.96	3.30	4.13	5.15	5.77	6.06
HPV16/18	Organised	Bordeaux	40	4.08	2.67	2.89	3.22	4.03	5.01	5.61	5.96
HPV16/18	Organised	Bordeaux	41	3.98	2.61	2.81	3.14	3.92	4.89	5.50	5.83
HPV16/18	Organised	Bordeaux	42	3.87	2.55	2.74	3.05	3.82	4.77	5.35	5.65
HPV16/18	Organised	Bordeaux	43	3.77	2.49	2.66	2.97	3.72	4.64	5.19	5.53
HPV16/18	Organised	Bordeaux	44	3.69	2.42	2.61	2.90	3.64	4.54	5.06	5.42
HPV16/18	Organised	Bordeaux	45	3.62	2.37	2.54	2.84	3.57	4.45	4.95	5.32
HPV16/18	Organised	Bordeaux	46	3.54	2.32	2.48	2.79	3.50	4.38	4.86	5.20
HPV16/18	Organised	Bordeaux	47	3.47	2.27	2.44	2.73	3.42	4.28	4.76	5.12
HPV16/18	Organised	Bordeaux	48	3.40	2.22	2.40	2.68	3.35	4.17	4.67	5.01
HPV16/18	Organised	Bordeaux	49	3.34	2.18	2.35	2.63	3.28	4.10	4.59	4.92
HPV16/18	Organised	Bordeaux	50	3.27	2.14	2.30	2.57	3.23	4.02	4.51	4.85
HPV16/18	Organised	Bordeaux	51	3.21	2.10	2.25	2.53	3.17	3.95	4.45	4.77
HPV16/18	Organised	Bordeaux	52	3.16	2.06	2.21	2.48	3.12	3.89	4.37	4.65
HPV16/18	Organised	Bordeaux	53	3.09	2.03	2.17	2.42	3.05	3.81	4.30	4.58
HPV16/18	Organised	Bordeaux	54	3.03	1.98	2.11	2.38	2.99	3.74	4.22	4.49
HPV16/18	Organised	Bordeaux	55	2.98	1.97	2.07	2.33	2.94	3.69	4.16	4.41
HPV16/18	Organised	Bordeaux	56	2.94	1.93	2.04	2.31	2.90	3.64	4.11	4.35
HPV16/18	Organised	Bordeaux	57	2.92	1.89	2.01	2.29	2.88	3.60	4.10	4.35
HPV16/18	Organised	Bordeaux	58	2.91	1.88	2.01	2.27	2.87	3.59	4.09	4.36
HPV16/18	Organised	Bordeaux	59	2.92	1.88	2.01	2.27	2.88	3.60	4.12	4.41
HPV16/18	Organised	Bordeaux	60	2.94	1.88	2.02	2.29	2.89	3.63	4.16	4.45
HPV16/18	Organised	Bordeaux	61	2.95	1.90	2.02	2.30	2.91	3.66	4.21	4.49

Table S9 Posterior expected HR HPV cervical infection prevalence (in %) in major French cities, stratified by type of test, city, and age. Table counterpart to Figure 3A. *(continued)*

				Posterior distribution							
					Percentiles						
Virus	Test	City	Age	Average	0.01	0.025	0.1	0.5	0.9	0.975	0.99
HPV16/18	Organised	Bordeaux	62	2.97	1.90	2.02	2.30	2.92	3.68	4.20	4.53
HPV16/18	Organised	Bordeaux	63	2.97	1.90	2.00	2.29	2.92	3.69	4.23	4.55
HPV16/18	Organised	Bordeaux	64	2.97	1.89	1.98	2.27	2.91	3.73	4.27	4.55
HPV16/18	Organised	Bordeaux	65	2.96	1.84	1.97	2.23	2.91	3.74	4.28	4.63
HPV16/18	Organised	Bordeaux	66	2.94	1.80	1.93	2.20	2.89	3.75	4.33	4.64
HPV16/18	Organised	Montpellier	30	4.41	2.59	2.77	3.23	4.30	5.73	6.79	7.29
HPV16/18	Organised	Montpellier	31	4.63	2.77	2.93	3.41	4.51	5.99	7.02	7.55
HPV16/18	Organised	Montpellier	32	4.82	2.89	3.09	3.57	4.73	6.20	7.23	7.82
HPV16/18	Organised	Montpellier	33	4.97	2.99	3.19	3.69	4.88	6.34	7.41	8.00
HPV16/18	Organised	Montpellier	34	5.03	3.04	3.24	3.73	4.94	6.42	7.48	8.04
HPV16/18	Organised	Montpellier	35	4.98	3.03	3.22	3.71	4.89	6.36	7.41	7.93
HPV16/18	Organised	Montpellier	36	4.88	2.96	3.16	3.64	4.79	6.23	7.25	7.75
HPV16/18	Organised	Montpellier	37	4.76	2.91	3.09	3.56	4.67	6.07	7.02	7.55
HPV16/18	Organised	Montpellier	38	4.64	2.84	3.02	3.47	4.56	5.90	6.88	7.40
HPV16/18	Organised	Montpellier	39	4.52	2.77	2.94	3.38	4.44	5.75	6.67	7.17
HPV16/18	Organised	Montpellier	40	4.41	2.69	2.86	3.30	4.32	5.61	6.51	7.01
HPV16/18	Organised	Montpellier	41	4.30	2.62	2.79	3.22	4.21	5.47	6.33	6.85
HPV16/18	Organised	Montpellier	42	4.18	2.54	2.71	3.12	4.09	5.33	6.17	6.72
HPV16/18	Organised	Montpellier	43	4.08	2.49	2.64	3.04	3.98	5.22	6.02	6.48
HPV16/18	Organised	Montpellier	44	3.99	2.42	2.57	2.95	3.90	5.10	5.86	6.38
HPV16/18	Organised	Montpellier	45	3.91	2.37	2.50	2.90	3.81	5.01	5.76	6.25
HPV16/18	Organised	Montpellier	46	3.83	2.31	2.45	2.83	3.73	4.90	5.65	6.15
HPV16/18	Organised	Montpellier	47	3.75	2.25	2.41	2.78	3.66	4.81	5.55	6.03
HPV16/18	Organised	Montpellier	48	3.67	2.20	2.37	2.72	3.59	4.70	5.44	6.00
HPV16/18	Organised	Montpellier	49	3.61	2.17	2.33	2.67	3.52	4.62	5.34	5.84
HPV16/18	Organised	Montpellier	50	3.54	2.13	2.29	2.62	3.46	4.55	5.23	5.70
HPV16/18	Organised	Montpellier	51	3.47	2.07	2.23	2.57	3.39	4.48	5.16	5.63
HPV16/18	Organised	Montpellier	52	3.41	2.03	2.18	2.52	3.33	4.41	5.07	5.55
HPV16/18	Organised	Montpellier	53	3.34	2.00	2.15	2.48	3.26	4.32	4.98	5.42

Table S9 Posterior expected HR HPV cervical infection prevalence (in %) in major French cities, stratified by type of test, city, and age. Table counterpart to Figure 3A. *(continued)*

				Posterior distribution							
					Percentiles						
Virus	Test	City	Age	Average	0.01	0.025	0.1	0.5	0.9	0.975	0.99
HPV16/18	Organised	Montpellier	54	3.28	1.95	2.09	2.43	3.20	4.22	4.88	5.32
HPV16/18	Organised	Montpellier	55	3.22	1.90	2.05	2.38	3.14	4.15	4.82	5.25
HPV16/18	Organised	Montpellier	56	3.18	1.86	2.03	2.34	3.11	4.11	4.77	5.21
HPV16/18	Organised	Montpellier	57	3.16	1.83	2.00	2.34	3.09	4.08	4.74	5.15
HPV16/18	Organised	Montpellier	58	3.15	1.84	2.00	2.32	3.08	4.07	4.73	5.17
HPV16/18	Organised	Montpellier	59	3.16	1.85	1.99	2.32	3.08	4.09	4.77	5.17
HPV16/18	Organised	Montpellier	60	3.17	1.85	2.01	2.33	3.10	4.12	4.81	5.17
HPV16/18	Organised	Montpellier	61	3.19	1.88	2.01	2.34	3.12	4.14	4.84	5.21
HPV16/18	Organised	Montpellier	62	3.21	1.88	1.99	2.36	3.13	4.17	4.86	5.25
HPV16/18	Organised	Montpellier	63	3.21	1.85	1.99	2.36	3.13	4.18	4.88	5.27
HPV16/18	Organised	Montpellier	64	3.21	1.83	1.98	2.35	3.12	4.21	4.88	5.31
HPV16/18	Organised	Montpellier	65	3.20	1.81	1.94	2.31	3.11	4.23	4.91	5.31
HPV16/18	Organised	Montpellier	66	3.18	1.77	1.91	2.29	3.09	4.23	4.93	5.32
HPV16/18	Organised	Rennes	30	3.93	2.55	2.71	3.04	3.88	4.89	5.56	5.83
HPV16/18	Organised	Rennes	31	4.12	2.71	2.86	3.22	4.08	5.11	5.74	6.03
HPV16/18	Organised	Rennes	32	4.30	2.85	3.00	3.37	4.25	5.30	5.92	6.20
HPV16/18	Organised	Rennes	33	4.43	2.94	3.11	3.51	4.39	5.48	6.04	6.38
HPV16/18	Organised	Rennes	34	4.48	3.00	3.16	3.55	4.44	5.52	6.09	6.42
HPV16/18	Organised	Rennes	35	4.44	2.98	3.14	3.52	4.39	5.44	6.04	6.31
HPV16/18	Organised	Rennes	36	4.35	2.94	3.09	3.46	4.30	5.32	5.90	6.17
HPV16/18	Organised	Rennes	37	4.24	2.87	3.03	3.37	4.19	5.18	5.76	6.05
HPV16/18	Organised	Rennes	38	4.14	2.80	2.96	3.29	4.08	5.05	5.61	5.91
HPV16/18	Organised	Rennes	39	4.03	2.75	2.89	3.20	3.98	4.92	5.48	5.78
HPV16/18	Organised	Rennes	40	3.93	2.67	2.82	3.12	3.88	4.80	5.36	5.69
HPV16/18	Organised	Rennes	41	3.83	2.62	2.74	3.05	3.78	4.67	5.25	5.55
HPV16/18	Organised	Rennes	42	3.72	2.53	2.67	2.97	3.68	4.56	5.13	5.41
HPV16/18	Organised	Rennes	43	3.63	2.47	2.60	2.89	3.58	4.44	5.00	5.29
HPV16/18	Organised	Rennes	44	3.55	2.42	2.53	2.82	3.50	4.34	4.87	5.21
HPV16/18	Organised	Rennes	45	3.48	2.36	2.48	2.76	3.43	4.25	4.78	5.11

Table S9 Posterior expected HR HPV cervical infection prevalence (in %) in major French cities, stratified by type of test, city, and age. Table counterpart to Figure 3A. *(continued)*

				Posterior distribution							
					Percentiles						
Virus	Test	City	Age	Average	0.01	0.025	0.1	0.5	0.9	0.975	0.99
HPV16/18	Organised	Rennes	46	3.41	2.31	2.44	2.71	3.36	4.16	4.68	4.99
HPV16/18	Organised	Rennes	47	3.34	2.26	2.39	2.65	3.29	4.08	4.58	4.85
HPV16/18	Organised	Rennes	48	3.27	2.21	2.34	2.59	3.23	4.01	4.49	4.78
HPV16/18	Organised	Rennes	49	3.21	2.16	2.30	2.54	3.17	3.93	4.42	4.70
HPV16/18	Organised	Rennes	50	3.15	2.13	2.25	2.49	3.10	3.86	4.36	4.64
HPV16/18	Organised	Rennes	51	3.09	2.07	2.21	2.44	3.05	3.80	4.26	4.53
HPV16/18	Organised	Rennes	52	3.04	2.01	2.16	2.39	2.99	3.73	4.20	4.43
HPV16/18	Organised	Rennes	53	2.98	1.97	2.11	2.34	2.93	3.66	4.12	4.35
HPV16/18	Organised	Rennes	54	2.91	1.94	2.06	2.28	2.87	3.58	4.02	4.30
HPV16/18	Organised	Rennes	55	2.86	1.91	2.03	2.25	2.82	3.52	3.95	4.26
HPV16/18	Organised	Rennes	56	2.83	1.90	2.00	2.22	2.79	3.50	3.90	4.21
HPV16/18	Organised	Rennes	57	2.81	1.87	1.98	2.20	2.78	3.48	3.89	4.18
HPV16/18	Organised	Rennes	58	2.80	1.85	1.96	2.19	2.77	3.46	3.89	4.17
HPV16/18	Organised	Rennes	59	2.81	1.85	1.96	2.18	2.78	3.48	3.92	4.17
HPV16/18	Organised	Rennes	60	2.82	1.85	1.97	2.19	2.79	3.50	3.92	4.18
HPV16/18	Organised	Rennes	61	2.84	1.86	1.97	2.21	2.81	3.52	3.96	4.21
HPV16/18	Organised	Rennes	62	2.85	1.86	1.96	2.22	2.82	3.54	3.99	4.24
HPV16/18	Organised	Rennes	63	2.86	1.85	1.95	2.21	2.82	3.56	4.00	4.27
HPV16/18	Organised	Rennes	64	2.86	1.82	1.95	2.20	2.82	3.57	4.03	4.27
HPV16/18	Organised	Rennes	65	2.85	1.78	1.92	2.18	2.81	3.58	4.03	4.29
HPV16/18	Organised	Rennes	66	2.83	1.74	1.89	2.14	2.79	3.60	4.07	4.32
HPV16/18	Organised	Nantes	30	3.62	2.01	2.16	2.56	3.52	4.79	5.60	6.19
HPV16/18	Organised	Nantes	31	3.80	2.14	2.29	2.70	3.70	5.01	5.83	6.37
HPV16/18	Organised	Nantes	32	3.96	2.23	2.41	2.83	3.85	5.20	6.06	6.60
HPV16/18	Organised	Nantes	33	4.08	2.32	2.50	2.94	3.97	5.34	6.25	6.77
HPV16/18	Organised	Nantes	34	4.13	2.35	2.54	2.99	4.03	5.42	6.24	6.80
HPV16/18	Organised	Nantes	35	4.09	2.32	2.51	2.96	3.98	5.38	6.21	6.69
HPV16/18	Organised	Nantes	36	4.01	2.29	2.49	2.91	3.90	5.29	6.11	6.55
HPV16/18	Organised	Nantes	37	3.91	2.23	2.43	2.84	3.80	5.14	5.94	6.39

Table S9 Posterior expected HR HPV cervical infection prevalence (in %) in major French cities, stratified by type of test, city, and age. Table counterpart to Figure 3A. ***(continued)***

				Posterior distribution							
					Percentiles						
Virus	Test	City	Age	Average	0.01	0.025	0.1	0.5	0.9	0.975	0.99
HPV16/18	Organised	Nantes	38	3.81	2.18	2.38	2.77	3.71	5.02	5.80	6.17
HPV16/18	Organised	Nantes	39	3.71	2.14	2.32	2.71	3.62	4.90	5.59	6.01
HPV16/18	Organised	Nantes	40	3.61	2.07	2.26	2.63	3.52	4.78	5.42	5.85
HPV16/18	Organised	Nantes	41	3.52	2.01	2.19	2.57	3.43	4.66	5.26	5.65
HPV16/18	Organised	Nantes	42	3.43	1.96	2.11	2.49	3.33	4.53	5.14	5.48
HPV16/18	Organised	Nantes	43	3.34	1.91	2.07	2.43	3.25	4.41	5.00	5.34
HPV16/18	Organised	Nantes	44	3.26	1.86	2.03	2.37	3.18	4.30	4.90	5.26
HPV16/18	Organised	Nantes	45	3.20	1.83	1.99	2.33	3.12	4.22	4.84	5.19
HPV16/18	Organised	Nantes	46	3.13	1.80	1.96	2.28	3.06	4.12	4.70	5.08
HPV16/18	Organised	Nantes	47	3.07	1.74	1.92	2.24	2.99	4.04	4.62	5.01
HPV16/18	Organised	Nantes	48	3.01	1.70	1.88	2.19	2.93	3.95	4.54	4.94
HPV16/18	Organised	Nantes	49	2.95	1.67	1.85	2.15	2.87	3.88	4.45	4.86
HPV16/18	Organised	Nantes	50	2.89	1.64	1.81	2.11	2.81	3.81	4.36	4.81
HPV16/18	Organised	Nantes	51	2.84	1.60	1.78	2.07	2.76	3.73	4.29	4.71
HPV16/18	Organised	Nantes	52	2.79	1.57	1.75	2.04	2.71	3.68	4.23	4.59
HPV16/18	Organised	Nantes	53	2.73	1.54	1.71	1.99	2.66	3.58	4.14	4.50
HPV16/18	Organised	Nantes	54	2.68	1.52	1.67	1.95	2.60	3.51	4.08	4.41
HPV16/18	Organised	Nantes	55	2.63	1.49	1.64	1.91	2.56	3.46	4.00	4.36
HPV16/18	Organised	Nantes	56	2.60	1.47	1.61	1.89	2.53	3.42	3.97	4.33
HPV16/18	Organised	Nantes	57	2.58	1.46	1.59	1.87	2.51	3.40	3.93	4.29
HPV16/18	Organised	Nantes	58	2.58	1.44	1.59	1.86	2.51	3.40	3.95	4.25
HPV16/18	Organised	Nantes	59	2.58	1.45	1.58	1.87	2.51	3.42	3.97	4.24
HPV16/18	Organised	Nantes	60	2.59	1.45	1.58	1.88	2.53	3.43	3.99	4.29
HPV16/18	Organised	Nantes	61	2.61	1.46	1.58	1.89	2.54	3.45	4.02	4.33
HPV16/18	Organised	Nantes	62	2.62	1.46	1.59	1.89	2.55	3.47	4.05	4.35
HPV16/18	Organised	Nantes	63	2.63	1.47	1.59	1.89	2.55	3.47	4.05	4.41
HPV16/18	Organised	Nantes	64	2.62	1.44	1.58	1.87	2.54	3.48	4.07	4.42
HPV16/18	Organised	Nantes	65	2.62	1.42	1.55	1.86	2.53	3.48	4.12	4.53
HPV16/18	Organised	Nantes	66	2.60	1.41	1.52	1.82	2.52	3.50	4.17	4.60

Table S9 **Posterior expected HR HPV cervical infection prevalence (in %) in major French cities, stratified by type of test, city, and age. Table counterpart to Figure 3A.** ***(continued)***

				Posterior distribution							
					Percentiles						
Virus	Test	City	Age	Average	0.01	0.025	0.1	0.5	0.9	0.975	0.99
HPV16/18	Organised	Lille	30	3.95	2.31	2.50	2.92	3.86	5.10	5.92	6.39
HPV16/18	Organised	Lille	31	4.14	2.47	2.65	3.08	4.06	5.32	6.15	6.61
HPV16/18	Organised	Lille	32	4.32	2.57	2.80	3.22	4.23	5.55	6.38	6.80
HPV16/18	Organised	Lille	33	4.45	2.65	2.91	3.33	4.36	5.70	6.58	6.99
HPV16/18	Organised	Lille	34	4.50	2.67	2.95	3.36	4.41	5.76	6.57	7.03
HPV16/18	Organised	Lille	35	4.46	2.68	2.92	3.34	4.37	5.72	6.50	6.91
HPV16/18	Organised	Lille	36	4.37	2.64	2.89	3.29	4.28	5.59	6.37	6.78
HPV16/18	Organised	Lille	37	4.26	2.57	2.83	3.21	4.18	5.44	6.17	6.62
HPV16/18	Organised	Lille	38	4.15	2.53	2.74	3.13	4.07	5.30	6.01	6.48
HPV16/18	Organised	Lille	39	4.05	2.47	2.68	3.05	3.97	5.16	5.84	6.31
HPV16/18	Organised	Lille	40	3.95	2.39	2.61	2.98	3.86	5.03	5.70	6.13
HPV16/18	Organised	Lille	41	3.85	2.34	2.54	2.90	3.77	4.90	5.60	6.06
HPV16/18	Organised	Lille	42	3.74	2.28	2.47	2.81	3.66	4.77	5.42	5.88
HPV16/18	Organised	Lille	43	3.65	2.22	2.41	2.74	3.57	4.66	5.30	5.77
HPV16/18	Organised	Lille	44	3.57	2.18	2.35	2.67	3.49	4.55	5.17	5.65
HPV16/18	Organised	Lille	45	3.50	2.14	2.30	2.63	3.43	4.46	5.07	5.53
HPV16/18	Organised	Lille	46	3.42	2.08	2.26	2.58	3.36	4.38	4.95	5.39
HPV16/18	Organised	Lille	47	3.35	2.02	2.19	2.52	3.29	4.29	4.84	5.32
HPV16/18	Organised	Lille	48	3.29	1.97	2.14	2.47	3.22	4.22	4.76	5.20
HPV16/18	Organised	Lille	49	3.22	1.95	2.10	2.43	3.16	4.13	4.67	5.10
HPV16/18	Organised	Lille	50	3.16	1.91	2.05	2.38	3.09	4.06	4.59	5.02
HPV16/18	Organised	Lille	51	3.11	1.86	2.00	2.33	3.04	3.99	4.50	4.88
HPV16/18	Organised	Lille	52	3.05	1.84	1.96	2.29	2.98	3.91	4.42	4.79
HPV16/18	Organised	Lille	53	2.99	1.80	1.93	2.23	2.92	3.84	4.34	4.67
HPV16/18	Organised	Lille	54	2.93	1.77	1.89	2.18	2.86	3.76	4.26	4.60
HPV16/18	Organised	Lille	55	2.88	1.74	1.86	2.14	2.81	3.70	4.20	4.53
HPV16/18	Organised	Lille	56	2.84	1.69	1.85	2.11	2.78	3.65	4.17	4.49
HPV16/18	Organised	Lille	57	2.82	1.66	1.83	2.10	2.76	3.62	4.16	4.44
HPV16/18	Organised	Lille	58	2.82	1.65	1.82	2.09	2.76	3.63	4.13	4.46

Table S9 Posterior expected HR HPV cervical infection prevalence (in %) in major French cities, stratified by type of test, city, and age. Table counterpart to Figure 3A. ***(continued)***

				Posterior distribution							
					Percentiles						
Virus	Test	City	Age	Average	0.01	0.025	0.1	0.5	0.9	0.975	0.99
HPV16/18	Organised	Lille	59	2.82	1.64	1.82	2.09	2.77	3.64	4.14	4.48
HPV16/18	Organised	Lille	60	2.84	1.64	1.81	2.10	2.78	3.66	4.14	4.51
HPV16/18	Organised	Lille	61	2.86	1.64	1.81	2.11	2.80	3.70	4.17	4.56
HPV16/18	Organised	Lille	62	2.87	1.67	1.81	2.12	2.81	3.71	4.22	4.59
HPV16/18	Organised	Lille	63	2.87	1.66	1.81	2.12	2.81	3.73	4.26	4.65
HPV16/18	Organised	Lille	64	2.87	1.62	1.80	2.10	2.80	3.74	4.29	4.68
HPV16/18	Organised	Lille	65	2.86	1.61	1.76	2.09	2.79	3.74	4.30	4.70
HPV16/18	Organised	Lille	66	2.85	1.60	1.72	2.05	2.77	3.76	4.36	4.74
HPV16/18	Organised	Strasbourg	30	3.23	1.71	1.88	2.26	3.13	4.35	5.24	5.76
HPV16/18	Organised	Strasbourg	31	3.39	1.82	1.99	2.38	3.27	4.56	5.46	5.99
HPV16/18	Organised	Strasbourg	32	3.54	1.90	2.08	2.49	3.42	4.72	5.71	6.24
HPV16/18	Organised	Strasbourg	33	3.65	2.00	2.15	2.56	3.54	4.88	5.81	6.45
HPV16/18	Organised	Strasbourg	34	3.69	2.02	2.18	2.60	3.57	4.93	5.85	6.48
HPV16/18	Organised	Strasbourg	35	3.66	2.02	2.17	2.58	3.53	4.86	5.79	6.46
HPV16/18	Organised	Strasbourg	36	3.58	1.97	2.13	2.53	3.47	4.77	5.64	6.33
HPV16/18	Organised	Strasbourg	37	3.49	1.92	2.09	2.47	3.38	4.65	5.49	6.15
HPV16/18	Organised	Strasbourg	38	3.40	1.87	2.04	2.41	3.29	4.53	5.35	6.01
HPV16/18	Organised	Strasbourg	39	3.32	1.82	1.98	2.34	3.20	4.41	5.22	5.89
HPV16/18	Organised	Strasbourg	40	3.23	1.77	1.93	2.29	3.12	4.31	5.09	5.75
HPV16/18	Organised	Strasbourg	41	3.15	1.72	1.87	2.23	3.04	4.21	4.97	5.63
HPV16/18	Organised	Strasbourg	42	3.06	1.69	1.82	2.16	2.96	4.09	4.85	5.42
HPV16/18	Organised	Strasbourg	43	2.98	1.65	1.78	2.10	2.88	3.98	4.72	5.33
HPV16/18	Organised	Strasbourg	44	2.92	1.61	1.74	2.06	2.82	3.89	4.62	5.20
HPV16/18	Organised	Strasbourg	45	2.86	1.57	1.70	2.03	2.77	3.81	4.55	5.07
HPV16/18	Organised	Strasbourg	46	2.80	1.54	1.67	1.98	2.71	3.74	4.45	4.96
HPV16/18	Organised	Strasbourg	47	2.74	1.50	1.64	1.94	2.65	3.67	4.34	4.86
HPV16/18	Organised	Strasbourg	48	2.69	1.47	1.61	1.89	2.60	3.61	4.27	4.76
HPV16/18	Organised	Strasbourg	49	2.64	1.44	1.58	1.85	2.55	3.54	4.23	4.67
HPV16/18	Organised	Strasbourg	50	2.59	1.40	1.55	1.82	2.50	3.46	4.15	4.60

Table S9 **Posterior expected HR HPV cervical infection prevalence (in %) in major French cities, stratified by type of test, city, and age. Table counterpart to Figure 3A.** ***(continued)***

				Posterior distribution								
					Percentiles							
Virus	Test	City	Age	Average	0.01	0.025	0.1	0.5	0.9	0.975	0.99
HPV16/18	Organised	Strasbourg	51	2.54	1.38	1.52	1.78	2.46	3.41	4.11	4.53
HPV16/18	Organised	Strasbourg	52	2.49	1.34	1.49	1.75	2.42	3.34	4.05	4.48
HPV16/18	Organised	Strasbourg	53	2.44	1.31	1.46	1.71	2.37	3.28	3.97	4.33
HPV16/18	Organised	Strasbourg	54	2.39	1.29	1.43	1.68	2.32	3.21	3.89	4.21
HPV16/18	Organised	Strasbourg	55	2.35	1.26	1.40	1.65	2.28	3.15	3.82	4.11
HPV16/18	Organised	Strasbourg	56	2.32	1.26	1.38	1.62	2.25	3.12	3.75	4.06
HPV16/18	Organised	Strasbourg	57	2.31	1.25	1.36	1.61	2.23	3.11	3.72	4.06
HPV16/18	Organised	Strasbourg	58	2.30	1.25	1.35	1.61	2.22	3.10	3.72	4.05
HPV16/18	Organised	Strasbourg	59	2.31	1.25	1.35	1.61	2.23	3.11	3.75	4.05
HPV16/18	Organised	Strasbourg	60	2.32	1.26	1.36	1.62	2.25	3.14	3.75	4.07
HPV16/18	Organised	Strasbourg	61	2.33	1.27	1.37	1.63	2.26	3.15	3.78	4.13
HPV16/18	Organised	Strasbourg	62	2.34	1.27	1.37	1.63	2.27	3.18	3.81	4.19
HPV16/18	Organised	Strasbourg	63	2.35	1.26	1.37	1.62	2.27	3.20	3.83	4.19
HPV16/18	Organised	Strasbourg	64	2.34	1.24	1.36	1.61	2.26	3.21	3.84	4.28
HPV16/18	Organised	Strasbourg	65	2.34	1.22	1.35	1.59	2.25	3.21	3.85	4.30
HPV16/18	Organised	Strasbourg	66	2.33	1.18	1.31	1.57	2.23	3.20	3.86	4.30
HPV16/18	Organised	Lyon	30	4.47	2.60	2.77	3.24	4.34	5.86	6.76	7.32
HPV16/18	Organised	Lyon	31	4.69	2.73	2.96	3.43	4.56	6.11	7.05	7.65
HPV16/18	Organised	Lyon	32	4.89	2.87	3.10	3.58	4.76	6.36	7.28	7.90
HPV16/18	Organised	Lyon	33	5.04	2.95	3.22	3.72	4.91	6.56	7.46	8.05
HPV16/18	Organised	Lyon	34	5.10	2.99	3.26	3.76	4.97	6.63	7.54	8.06
HPV16/18	Organised	Lyon	35	5.05	2.96	3.24	3.73	4.91	6.56	7.47	7.97
HPV16/18	Organised	Lyon	36	4.95	2.94	3.18	3.66	4.83	6.44	7.34	7.79
HPV16/18	Organised	Lyon	37	4.83	2.84	3.11	3.57	4.72	6.27	7.19	7.60
HPV16/18	Organised	Lyon	38	4.71	2.76	3.04	3.48	4.59	6.10	7.01	7.40
HPV16/18	Organised	Lyon	39	4.59	2.72	2.97	3.39	4.48	5.94	6.84	7.17
HPV16/18	Organised	Lyon	40	4.47	2.65	2.88	3.31	4.36	5.78	6.66	7.04
HPV16/18	Organised	Lyon	41	4.36	2.58	2.80	3.24	4.25	5.63	6.47	6.86
HPV16/18	Organised	Lyon	42	4.24	2.53	2.72	3.15	4.14	5.47	6.28	6.70

Table S9 Posterior expected HR HPV cervical infection prevalence (in %) in major French cities, stratified by type of test, city, and age. Table counterpart to Figure 3A. *(continued)*

				Posterior distribution							
					Percentiles						
Virus	Test	City	Age	Average	0.01	0.025	0.1	0.5	0.9	0.975	0.99
HPV16/18	Organised	Lyon	43	4.13	2.45	2.66	3.06	4.04	5.33	6.11	6.55
HPV16/18	Organised	Lyon	44	4.04	2.38	2.59	2.99	3.95	5.20	5.96	6.42
HPV16/18	Organised	Lyon	45	3.97	2.35	2.56	2.93	3.87	5.11	5.86	6.30
HPV16/18	Organised	Lyon	46	3.88	2.30	2.50	2.87	3.79	5.01	5.77	6.15
HPV16/18	Organised	Lyon	47	3.80	2.26	2.44	2.80	3.71	4.91	5.68	6.07
HPV16/18	Organised	Lyon	48	3.73	2.22	2.40	2.74	3.64	4.81	5.59	5.96
HPV16/18	Organised	Lyon	49	3.66	2.20	2.35	2.69	3.57	4.73	5.50	5.85
HPV16/18	Organised	Lyon	50	3.59	2.14	2.29	2.64	3.50	4.65	5.42	5.77
HPV16/18	Organised	Lyon	51	3.52	2.10	2.25	2.59	3.44	4.55	5.31	5.71
HPV16/18	Organised	Lyon	52	3.46	2.05	2.22	2.54	3.38	4.49	5.23	5.59
HPV16/18	Organised	Lyon	53	3.39	2.02	2.17	2.49	3.31	4.40	5.13	5.48
HPV16/18	Organised	Lyon	54	3.32	1.97	2.12	2.43	3.23	4.31	5.01	5.37
HPV16/18	Organised	Lyon	55	3.27	1.94	2.08	2.40	3.18	4.23	4.93	5.31
HPV16/18	Organised	Lyon	56	3.23	1.90	2.04	2.36	3.14	4.18	4.87	5.24
HPV16/18	Organised	Lyon	57	3.20	1.88	2.03	2.34	3.12	4.15	4.84	5.21
HPV16/18	Organised	Lyon	58	3.20	1.87	2.03	2.32	3.11	4.15	4.81	5.20
HPV16/18	Organised	Lyon	59	3.20	1.86	2.02	2.33	3.12	4.17	4.83	5.23
HPV16/18	Organised	Lyon	60	3.22	1.86	2.02	2.34	3.14	4.22	4.85	5.27
HPV16/18	Organised	Lyon	61	3.24	1.87	2.03	2.34	3.15	4.26	4.90	5.31
HPV16/18	Organised	Lyon	62	3.26	1.86	2.03	2.34	3.17	4.26	4.90	5.37
HPV16/18	Organised	Lyon	63	3.26	1.87	2.03	2.34	3.17	4.28	4.95	5.40
HPV16/18	Organised	Lyon	64	3.26	1.88	2.01	2.32	3.15	4.30	4.98	5.44
HPV16/18	Organised	Lyon	65	3.25	1.87	1.97	2.30	3.15	4.33	5.00	5.51
HPV16/18	Organised	Lyon	66	3.23	1.81	1.94	2.27	3.13	4.35	5.05	5.52
HPV16/18	Organised	Paris	30	4.74	3.11	3.33	3.75	4.66	5.79	6.59	6.95
HPV16/18	Organised	Paris	31	4.97	3.31	3.55	3.96	4.91	6.04	6.81	7.22
HPV16/18	Organised	Paris	32	5.18	3.50	3.72	4.15	5.13	6.29	7.08	7.52
HPV16/18	Organised	Paris	33	5.34	3.67	3.84	4.29	5.29	6.45	7.20	7.67
HPV16/18	Organised	Paris	34	5.40	3.73	3.89	4.34	5.35	6.51	7.21	7.70

Table S9 **Posterior expected HR HPV cervical infection prevalence (in %) in major French cities, stratified by type of test, city, and age. Table counterpart to Figure 3A.** ***(continued)***

				Posterior distribution							
					Percentiles						
Virus	Test	City	Age	Average	0.01	0.025	0.1	0.5	0.9	0.975	0.99
HPV16/18	Organised	Paris	35	5.35	3.71	3.88	4.29	5.30	6.43	7.11	7.62
HPV16/18	Organised	Paris	36	5.24	3.63	3.83	4.22	5.20	6.30	6.93	7.45
HPV16/18	Organised	Paris	37	5.11	3.56	3.76	4.13	5.07	6.15	6.75	7.23
HPV16/18	Organised	Paris	38	4.98	3.51	3.68	4.03	4.94	6.00	6.59	7.05
HPV16/18	Organised	Paris	39	4.86	3.43	3.59	3.94	4.81	5.85	6.44	6.86
HPV16/18	Organised	Paris	40	4.73	3.31	3.50	3.84	4.68	5.70	6.26	6.71
HPV16/18	Organised	Paris	41	4.62	3.22	3.40	3.75	4.56	5.57	6.13	6.55
HPV16/18	Organised	Paris	42	4.49	3.13	3.30	3.65	4.44	5.42	5.95	6.38
HPV16/18	Organised	Paris	43	4.38	3.05	3.22	3.56	4.32	5.27	5.81	6.23
HPV16/18	Organised	Paris	44	4.28	2.98	3.16	3.47	4.22	5.15	5.72	6.07
HPV16/18	Organised	Paris	45	4.20	2.93	3.09	3.41	4.14	5.06	5.59	5.98
HPV16/18	Organised	Paris	46	4.11	2.87	3.03	3.33	4.06	4.95	5.47	5.86
HPV16/18	Organised	Paris	47	4.03	2.80	2.96	3.26	3.98	4.85	5.36	5.73
HPV16/18	Organised	Paris	48	3.95	2.73	2.89	3.20	3.90	4.76	5.29	5.64
HPV16/18	Organised	Paris	49	3.87	2.68	2.83	3.14	3.83	4.66	5.21	5.52
HPV16/18	Organised	Paris	50	3.80	2.63	2.76	3.07	3.75	4.58	5.11	5.43
HPV16/18	Organised	Paris	51	3.73	2.58	2.71	3.01	3.68	4.50	5.02	5.33
HPV16/18	Organised	Paris	52	3.67	2.51	2.67	2.96	3.62	4.43	4.94	5.25
HPV16/18	Organised	Paris	53	3.59	2.46	2.63	2.90	3.54	4.34	4.85	5.16
HPV16/18	Organised	Paris	54	3.52	2.40	2.57	2.84	3.47	4.25	4.74	5.05
HPV16/18	Organised	Paris	55	3.46	2.36	2.50	2.78	3.41	4.18	4.66	4.97
HPV16/18	Organised	Paris	56	3.42	2.31	2.47	2.75	3.38	4.13	4.63	4.90
HPV16/18	Organised	Paris	57	3.39	2.28	2.43	2.72	3.35	4.11	4.61	4.89
HPV16/18	Organised	Paris	58	3.39	2.25	2.42	2.71	3.34	4.12	4.61	4.88
HPV16/18	Organised	Paris	59	3.39	2.25	2.42	2.71	3.35	4.14	4.61	4.88
HPV16/18	Organised	Paris	60	3.41	2.29	2.42	2.72	3.36	4.17	4.65	4.91
HPV16/18	Organised	Paris	61	3.43	2.28	2.43	2.74	3.38	4.21	4.72	4.97
HPV16/18	Organised	Paris	62	3.45	2.31	2.43	2.74	3.39	4.23	4.72	5.01
HPV16/18	Organised	Paris	63	3.45	2.30	2.42	2.73	3.40	4.24	4.73	5.07

Table S9 Posterior expected HR HPV cervical infection prevalence (in %) in major French cities, stratified by type of test, city, and age. Table counterpart to Figure 3A. ***(continued)***

				Posterior distribution							
					Percentiles						
Virus	Test	City	Age	Average	0.01	0.025	0.1	0.5	0.9	0.975	0.99
HPV16/18	Organised	Paris	64	3.45	2.29	2.42	2.71	3.40	4.26	4.77	5.13
HPV16/18	Organised	Paris	65	3.44	2.24	2.38	2.69	3.38	4.27	4.83	5.16
HPV16/18	Organised	Paris	66	3.42	2.16	2.32	2.63	3.37	4.28	4.91	5.23
HPV16/18	Opportunistic	Nice	30	5.00	2.46	2.75	3.37	4.83	6.88	8.38	9.11
HPV16/18	Opportunistic	Nice	31	5.29	2.62	2.94	3.56	5.12	7.27	8.77	9.74
HPV16/18	Opportunistic	Nice	32	5.55	2.74	3.08	3.73	5.37	7.61	9.18	10.23
HPV16/18	Opportunistic	Nice	33	5.75	2.87	3.18	3.85	5.55	7.88	9.57	10.61
HPV16/18	Opportunistic	Nice	34	5.83	2.90	3.26	3.92	5.63	8.00	9.68	10.68
HPV16/18	Opportunistic	Nice	35	5.78	2.89	3.23	3.89	5.58	7.95	9.59	10.60
HPV16/18	Opportunistic	Nice	36	5.67	2.81	3.15	3.81	5.48	7.76	9.40	10.38
HPV16/18	Opportunistic	Nice	37	5.53	2.77	3.07	3.72	5.34	7.57	9.18	10.14
HPV16/18	Opportunistic	Nice	38	5.38	2.69	2.99	3.62	5.21	7.38	8.95	9.92
HPV16/18	Opportunistic	Nice	39	5.24	2.62	2.90	3.54	5.06	7.21	8.70	9.61
HPV16/18	Opportunistic	Nice	40	5.10	2.53	2.83	3.43	4.91	7.04	8.44	9.36
HPV16/18	Opportunistic	Nice	41	4.96	2.46	2.76	3.33	4.79	6.85	8.25	9.14
HPV16/18	Opportunistic	Nice	42	4.81	2.38	2.68	3.23	4.65	6.63	8.03	8.91
HPV16/18	Opportunistic	Nice	43	4.68	2.30	2.60	3.14	4.51	6.45	7.82	8.64
HPV16/18	Opportunistic	Nice	44	4.56	2.25	2.53	3.06	4.40	6.28	7.59	8.40
HPV16/18	Opportunistic	Nice	45	4.47	2.20	2.45	3.00	4.30	6.14	7.42	8.23
HPV16/18	Opportunistic	Nice	46	4.36	2.13	2.39	2.92	4.20	5.98	7.27	8.02
HPV16/18	Opportunistic	Nice	47	4.26	2.09	2.34	2.84	4.11	5.87	7.12	7.84
HPV16/18	Opportunistic	Nice	48	4.16	2.06	2.29	2.78	4.01	5.74	6.95	7.71
HPV16/18	Opportunistic	Nice	49	4.07	2.02	2.22	2.71	3.92	5.62	6.81	7.50
HPV16/18	Opportunistic	Nice	50	3.98	1.97	2.17	2.65	3.84	5.49	6.66	7.29
HPV16/18	Opportunistic	Nice	51	3.89	1.94	2.12	2.60	3.76	5.35	6.52	7.13
HPV16/18	Opportunistic	Nice	52	3.81	1.90	2.09	2.53	3.67	5.25	6.41	7.00
HPV16/18	Opportunistic	Nice	53	3.71	1.84	2.03	2.48	3.57	5.13	6.26	6.85
HPV16/18	Opportunistic	Nice	54	3.62	1.77	1.97	2.40	3.49	4.99	6.12	6.69
HPV16/18	Opportunistic	Nice	55	3.54	1.74	1.93	2.35	3.41	4.88	6.00	6.59

Table S9 **Posterior expected HR HPV cervical infection prevalence (in %) in major French cities, stratified by type of test, city, and age. Table counterpart to Figure 3A.** ***(continued)***

				Posterior distribution							
					Percentiles						
Virus	Test	City	Age	Average	0.01	0.025	0.1	0.5	0.9	0.975	0.99
HPV16/18	Opportunistic	Nice	56	3.48	1.71	1.91	2.32	3.36	4.80	5.89	6.48
HPV16/18	Opportunistic	Nice	57	3.43	1.69	1.88	2.29	3.32	4.75	5.80	6.37
HPV16/18	Opportunistic	Nice	58	3.41	1.67	1.87	2.27	3.28	4.71	5.73	6.34
HPV16/18	Opportunistic	Nice	59	3.40	1.67	1.85	2.25	3.27	4.71	5.68	6.32
HPV16/18	Opportunistic	Nice	60	3.40	1.67	1.85	2.25	3.28	4.71	5.71	6.36
HPV16/18	Opportunistic	Nice	61	3.40	1.69	1.86	2.26	3.28	4.73	5.74	6.38
HPV16/18	Opportunistic	Nice	62	3.40	1.69	1.85	2.25	3.27	4.71	5.69	6.33
HPV16/18	Opportunistic	Nice	63	3.38	1.68	1.84	2.25	3.25	4.67	5.68	6.27
HPV16/18	Opportunistic	Nice	64	3.36	1.67	1.83	2.23	3.23	4.64	5.65	6.25
HPV16/18	Opportunistic	Nice	65	3.33	1.64	1.82	2.20	3.20	4.58	5.62	6.18
HPV16/18	Opportunistic	Nice	66	3.29	1.59	1.79	2.15	3.16	4.54	5.61	6.23
HPV16/18	Opportunistic	Marseille	30	5.67	4.23	4.45	4.85	5.64	6.52	7.08	7.41
HPV16/18	Opportunistic	Marseille	31	5.99	4.52	4.73	5.14	5.96	6.88	7.46	7.76
HPV16/18	Opportunistic	Marseille	32	6.29	4.75	4.95	5.39	6.25	7.22	7.79	8.10
HPV16/18	Opportunistic	Marseille	33	6.51	4.91	5.13	5.58	6.48	7.46	8.03	8.34
HPV16/18	Opportunistic	Marseille	34	6.60	4.97	5.20	5.65	6.58	7.55	8.15	8.48
HPV16/18	Opportunistic	Marseille	35	6.55	4.95	5.13	5.61	6.51	7.50	8.09	8.45
HPV16/18	Opportunistic	Marseille	36	6.42	4.88	5.03	5.51	6.38	7.39	7.96	8.26
HPV16/18	Opportunistic	Marseille	37	6.26	4.72	4.94	5.35	6.22	7.17	7.79	8.04
HPV16/18	Opportunistic	Marseille	38	6.10	4.59	4.83	5.21	6.05	7.00	7.59	7.85
HPV16/18	Opportunistic	Marseille	39	5.93	4.47	4.69	5.08	5.90	6.82	7.38	7.66
HPV16/18	Opportunistic	Marseille	40	5.77	4.33	4.54	4.95	5.73	6.66	7.17	7.50
HPV16/18	Opportunistic	Marseille	41	5.62	4.19	4.42	4.81	5.58	6.48	6.97	7.31
HPV16/18	Opportunistic	Marseille	42	5.45	4.08	4.29	4.68	5.41	6.30	6.75	7.11
HPV16/18	Opportunistic	Marseille	43	5.30	3.96	4.17	4.54	5.27	6.12	6.57	6.87
HPV16/18	Opportunistic	Marseille	44	5.17	3.87	4.06	4.43	5.14	5.97	6.43	6.70
HPV16/18	Opportunistic	Marseille	45	5.06	3.78	3.98	4.32	5.03	5.85	6.28	6.54
HPV16/18	Opportunistic	Marseille	46	4.94	3.68	3.88	4.22	4.91	5.70	6.14	6.41
HPV16/18	Opportunistic	Marseille	47	4.82	3.59	3.80	4.12	4.79	5.56	6.01	6.24

Table S9 Posterior expected HR HPV cervical infection prevalence (in %) in major French cities, stratified by type of test, city, and age. Table counterpart to Figure 3A. *(continued)*

				Posterior distribution							
					Percentiles						
Virus	Test	City	Age	Average	0.01	0.025	0.1	0.5	0.9	0.975	0.99
HPV16/18	Opportunistic	Marseille	48	4.72	3.52	3.72	4.03	4.68	5.43	5.88	6.10
HPV16/18	Opportunistic	Marseille	49	4.61	3.45	3.64	3.94	4.58	5.32	5.75	5.96
HPV16/18	Opportunistic	Marseille	50	4.51	3.40	3.57	3.84	4.48	5.20	5.60	5.83
HPV16/18	Opportunistic	Marseille	51	4.41	3.33	3.49	3.76	4.38	5.09	5.48	5.72
HPV16/18	Opportunistic	Marseille	52	4.32	3.24	3.41	3.68	4.29	4.98	5.38	5.60
HPV16/18	Opportunistic	Marseille	53	4.21	3.14	3.33	3.59	4.19	4.86	5.26	5.48
HPV16/18	Opportunistic	Marseille	54	4.10	3.05	3.22	3.50	4.08	4.73	5.10	5.40
HPV16/18	Opportunistic	Marseille	55	4.01	2.98	3.15	3.42	3.98	4.63	5.01	5.31
HPV16/18	Opportunistic	Marseille	56	3.94	2.95	3.08	3.36	3.92	4.54	4.92	5.19
HPV16/18	Opportunistic	Marseille	57	3.89	2.89	3.04	3.32	3.87	4.49	4.87	5.08
HPV16/18	Opportunistic	Marseille	58	3.86	2.86	3.02	3.30	3.84	4.45	4.84	5.00
HPV16/18	Opportunistic	Marseille	59	3.85	2.85	3.00	3.28	3.83	4.45	4.80	5.00
HPV16/18	Opportunistic	Marseille	60	3.85	2.86	3.00	3.28	3.83	4.45	4.81	5.03
HPV16/18	Opportunistic	Marseille	61	3.86	2.86	3.01	3.29	3.84	4.47	4.84	5.05
HPV16/18	Opportunistic	Marseille	62	3.85	2.86	3.01	3.28	3.83	4.45	4.84	5.08
HPV16/18	Opportunistic	Marseille	63	3.84	2.84	2.99	3.26	3.80	4.44	4.83	5.04
HPV16/18	Opportunistic	Marseille	64	3.81	2.82	2.95	3.23	3.78	4.42	4.81	5.02
HPV16/18	Opportunistic	Marseille	65	3.78	2.76	2.90	3.19	3.74	4.39	4.82	5.02
HPV16/18	Opportunistic	Marseille	66	3.73	2.67	2.82	3.11	3.70	4.38	4.84	5.04
HPV16/18	Opportunistic	Toulouse	30	5.19	3.06	3.23	3.80	5.08	6.73	7.75	8.60
HPV16/18	Opportunistic	Toulouse	31	5.49	3.23	3.45	4.00	5.36	7.10	8.21	9.00
HPV16/18	Opportunistic	Toulouse	32	5.76	3.37	3.66	4.22	5.63	7.46	8.54	9.37
HPV16/18	Opportunistic	Toulouse	33	5.96	3.51	3.80	4.39	5.83	7.70	8.88	9.69
HPV16/18	Opportunistic	Toulouse	34	6.05	3.55	3.86	4.45	5.92	7.80	8.99	9.78
HPV16/18	Opportunistic	Toulouse	35	6.00	3.51	3.81	4.40	5.88	7.75	8.90	9.74
HPV16/18	Opportunistic	Toulouse	36	5.88	3.46	3.72	4.32	5.76	7.60	8.71	9.64
HPV16/18	Opportunistic	Toulouse	37	5.73	3.38	3.60	4.18	5.59	7.39	8.53	9.39
HPV16/18	Opportunistic	Toulouse	38	5.58	3.28	3.53	4.06	5.45	7.21	8.31	9.14
HPV16/18	Opportunistic	Toulouse	39	5.43	3.20	3.44	3.95	5.30	7.03	8.07	8.88

Table S9 **Posterior expected HR HPV cervical infection prevalence (in %) in major French cities, stratified by type of test, city, and age. Table counterpart to Figure 3A.** ***(continued)***

				Posterior distribution							
					Percentiles						
Virus	Test	City	Age	Average	0.01	0.025	0.1	0.5	0.9	0.975	0.99
HPV16/18	Opportunistic	Toulouse	40	5.28	3.11	3.35	3.84	5.15	6.84	7.82	8.54
HPV16/18	Opportunistic	Toulouse	41	5.14	3.00	3.25	3.73	5.02	6.65	7.66	8.34
HPV16/18	Opportunistic	Toulouse	42	4.99	2.91	3.15	3.63	4.87	6.46	7.47	8.09
HPV16/18	Opportunistic	Toulouse	43	4.85	2.83	3.06	3.54	4.73	6.28	7.26	7.92
HPV16/18	Opportunistic	Toulouse	44	4.73	2.74	2.97	3.45	4.63	6.13	7.08	7.74
HPV16/18	Opportunistic	Toulouse	45	4.63	2.67	2.91	3.37	4.54	6.01	6.93	7.58
HPV16/18	Opportunistic	Toulouse	46	4.52	2.61	2.85	3.29	4.43	5.87	6.75	7.36
HPV16/18	Opportunistic	Toulouse	47	4.41	2.54	2.78	3.22	4.33	5.74	6.60	7.17
HPV16/18	Opportunistic	Toulouse	48	4.31	2.48	2.72	3.15	4.22	5.62	6.48	7.05
HPV16/18	Opportunistic	Toulouse	49	4.22	2.42	2.66	3.07	4.12	5.47	6.34	6.93
HPV16/18	Opportunistic	Toulouse	50	4.12	2.35	2.60	2.99	4.02	5.33	6.21	6.78
HPV16/18	Opportunistic	Toulouse	51	4.04	2.33	2.54	2.93	3.94	5.22	6.07	6.63
HPV16/18	Opportunistic	Toulouse	52	3.95	2.25	2.48	2.86	3.85	5.11	5.95	6.47
HPV16/18	Opportunistic	Toulouse	53	3.85	2.21	2.41	2.79	3.76	4.99	5.82	6.34
HPV16/18	Opportunistic	Toulouse	54	3.75	2.16	2.35	2.72	3.66	4.90	5.65	6.20
HPV16/18	Opportunistic	Toulouse	55	3.67	2.12	2.28	2.66	3.58	4.78	5.51	6.04
HPV16/18	Opportunistic	Toulouse	56	3.61	2.08	2.24	2.61	3.52	4.69	5.44	5.95
HPV16/18	Opportunistic	Toulouse	57	3.56	2.06	2.23	2.60	3.48	4.65	5.36	5.88
HPV16/18	Opportunistic	Toulouse	58	3.53	2.03	2.21	2.57	3.45	4.62	5.32	5.79
HPV16/18	Opportunistic	Toulouse	59	3.52	2.02	2.20	2.56	3.43	4.58	5.33	5.77
HPV16/18	Opportunistic	Toulouse	60	3.52	2.03	2.19	2.56	3.44	4.58	5.35	5.75
HPV16/18	Opportunistic	Toulouse	61	3.53	2.03	2.20	2.56	3.44	4.59	5.34	5.75
HPV16/18	Opportunistic	Toulouse	62	3.52	2.04	2.19	2.55	3.45	4.58	5.31	5.75
HPV16/18	Opportunistic	Toulouse	63	3.51	2.02	2.18	2.53	3.43	4.55	5.28	5.76
HPV16/18	Opportunistic	Toulouse	64	3.48	2.01	2.18	2.50	3.40	4.53	5.23	5.74
HPV16/18	Opportunistic	Toulouse	65	3.45	1.97	2.15	2.47	3.37	4.48	5.25	5.70
HPV16/18	Opportunistic	Toulouse	66	3.41	1.93	2.09	2.44	3.33	4.47	5.21	5.77
HPV16/18	Opportunistic	Bordeaux	30	5.78	4.23	4.39	4.80	5.72	6.83	7.40	7.91
HPV16/18	Opportunistic	Bordeaux	31	6.11	4.50	4.69	5.10	6.06	7.22	7.82	8.24

Table S9 **Posterior expected HR HPV cervical infection prevalence (in %) in major French cities, stratified by type of test, city, and age. Table counterpart to Figure 3A.** ***(continued)***

				Posterior distribution							
					Percentiles						
Virus	Test	City	Age	Average	0.01	0.025	0.1	0.5	0.9	0.975	0.99
HPV16/18	Opportunistic	Bordeaux	32	6.40	4.71	4.91	5.36	6.36	7.57	8.21	8.65
HPV16/18	Opportunistic	Bordeaux	33	6.63	4.88	5.09	5.55	6.58	7.83	8.52	8.91
HPV16/18	Opportunistic	Bordeaux	34	6.73	4.98	5.17	5.62	6.68	7.93	8.65	9.06
HPV16/18	Opportunistic	Bordeaux	35	6.67	4.94	5.12	5.57	6.62	7.86	8.59	8.98
HPV16/18	Opportunistic	Bordeaux	36	6.54	4.82	5.04	5.45	6.49	7.70	8.43	8.86
HPV16/18	Opportunistic	Bordeaux	37	6.38	4.69	4.90	5.31	6.32	7.52	8.20	8.62
HPV16/18	Opportunistic	Bordeaux	38	6.21	4.58	4.77	5.17	6.15	7.33	8.00	8.39
HPV16/18	Opportunistic	Bordeaux	39	6.05	4.43	4.64	5.04	6.00	7.14	7.81	8.17
HPV16/18	Opportunistic	Bordeaux	40	5.88	4.29	4.51	4.89	5.83	6.96	7.60	7.97
HPV16/18	Opportunistic	Bordeaux	41	5.72	4.19	4.38	4.76	5.66	6.76	7.37	7.78
HPV16/18	Opportunistic	Bordeaux	42	5.56	4.07	4.25	4.63	5.50	6.55	7.17	7.53
HPV16/18	Opportunistic	Bordeaux	43	5.40	3.97	4.13	4.50	5.35	6.37	6.97	7.30
HPV16/18	Opportunistic	Bordeaux	44	5.27	3.88	4.03	4.40	5.22	6.22	6.78	7.11
HPV16/18	Opportunistic	Bordeaux	45	5.16	3.78	3.93	4.30	5.11	6.11	6.65	6.93
HPV16/18	Opportunistic	Bordeaux	46	5.04	3.70	3.84	4.19	4.98	5.97	6.51	6.74
HPV16/18	Opportunistic	Bordeaux	47	4.92	3.59	3.74	4.10	4.86	5.82	6.34	6.61
HPV16/18	Opportunistic	Bordeaux	48	4.81	3.50	3.65	4.00	4.75	5.69	6.21	6.52
HPV16/18	Opportunistic	Bordeaux	49	4.70	3.42	3.58	3.91	4.65	5.56	6.07	6.36
HPV16/18	Opportunistic	Bordeaux	50	4.59	3.36	3.50	3.81	4.54	5.45	5.93	6.25
HPV16/18	Opportunistic	Bordeaux	51	4.50	3.27	3.44	3.73	4.46	5.34	5.82	6.11
HPV16/18	Opportunistic	Bordeaux	52	4.40	3.20	3.36	3.65	4.36	5.22	5.70	5.96
HPV16/18	Opportunistic	Bordeaux	53	4.29	3.10	3.26	3.56	4.26	5.08	5.57	5.83
HPV16/18	Opportunistic	Bordeaux	54	4.18	3.02	3.17	3.47	4.15	4.95	5.41	5.69
HPV16/18	Opportunistic	Bordeaux	55	4.09	2.96	3.09	3.40	4.05	4.85	5.31	5.58
HPV16/18	Opportunistic	Bordeaux	56	4.02	2.90	3.04	3.33	3.97	4.77	5.23	5.47
HPV16/18	Opportunistic	Bordeaux	57	3.97	2.88	3.01	3.29	3.92	4.71	5.16	5.42
HPV16/18	Opportunistic	Bordeaux	58	3.94	2.84	2.98	3.27	3.89	4.68	5.12	5.39
HPV16/18	Opportunistic	Bordeaux	59	3.92	2.82	2.98	3.25	3.88	4.68	5.10	5.36
HPV16/18	Opportunistic	Bordeaux	60	3.93	2.85	2.98	3.25	3.88	4.68	5.10	5.38

Table S9 **Posterior expected HR HPV cervical infection prevalence (in %) in major French cities, stratified by type of test, city, and age. Table counterpart to Figure 3A.** ***(continued)***

				Posterior distribution							
					Percentiles						
Virus	Test	City	Age	Average	0.01	0.025	0.1	0.5	0.9	0.975	0.99
HPV16/18	Opportunistic	Bordeaux	61	3.93	2.84	2.97	3.25	3.89	4.67	5.10	5.38
HPV16/18	Opportunistic	Bordeaux	62	3.93	2.85	2.97	3.25	3.88	4.66	5.10	5.35
HPV16/18	Opportunistic	Bordeaux	63	3.91	2.83	2.96	3.22	3.87	4.65	5.08	5.31
HPV16/18	Opportunistic	Bordeaux	64	3.88	2.79	2.92	3.20	3.84	4.62	5.05	5.28
HPV16/18	Opportunistic	Bordeaux	65	3.85	2.75	2.88	3.16	3.81	4.60	5.05	5.25
HPV16/18	Opportunistic	Bordeaux	66	3.80	2.65	2.82	3.09	3.76	4.56	5.04	5.29
HPV16/18	Opportunistic	Montpellier	30	6.11	3.86	4.09	4.62	5.98	7.73	8.74	9.44
HPV16/18	Opportunistic	Montpellier	31	6.46	4.09	4.31	4.92	6.33	8.17	9.19	10.00
HPV16/18	Opportunistic	Montpellier	32	6.77	4.28	4.56	5.16	6.63	8.55	9.67	10.36
HPV16/18	Opportunistic	Montpellier	33	7.02	4.41	4.71	5.36	6.87	8.82	10.08	10.69
HPV16/18	Opportunistic	Montpellier	34	7.11	4.46	4.79	5.44	6.98	8.95	10.23	10.85
HPV16/18	Opportunistic	Montpellier	35	7.06	4.43	4.76	5.40	6.92	8.88	10.09	10.74
HPV16/18	Opportunistic	Montpellier	36	6.92	4.35	4.65	5.30	6.80	8.71	9.87	10.58
HPV16/18	Opportunistic	Montpellier	37	6.74	4.25	4.51	5.15	6.63	8.45	9.61	10.32
HPV16/18	Opportunistic	Montpellier	38	6.57	4.17	4.40	5.01	6.46	8.26	9.40	10.18
HPV16/18	Opportunistic	Montpellier	39	6.40	4.03	4.27	4.88	6.28	8.06	9.17	10.06
HPV16/18	Opportunistic	Montpellier	40	6.22	3.91	4.16	4.73	6.10	7.86	8.98	9.78
HPV16/18	Opportunistic	Montpellier	41	6.06	3.81	4.02	4.59	5.95	7.65	8.77	9.60
HPV16/18	Opportunistic	Montpellier	42	5.88	3.68	3.92	4.46	5.77	7.43	8.48	9.26
HPV16/18	Opportunistic	Montpellier	43	5.72	3.59	3.80	4.34	5.61	7.23	8.24	8.95
HPV16/18	Opportunistic	Montpellier	44	5.58	3.49	3.72	4.22	5.47	7.05	8.06	8.68
HPV16/18	Opportunistic	Montpellier	45	5.46	3.42	3.65	4.14	5.35	6.91	7.89	8.42
HPV16/18	Opportunistic	Montpellier	46	5.33	3.36	3.56	4.05	5.23	6.72	7.70	8.26
HPV16/18	Opportunistic	Montpellier	47	5.21	3.24	3.48	3.95	5.10	6.56	7.52	8.07
HPV16/18	Opportunistic	Montpellier	48	5.09	3.17	3.40	3.85	4.99	6.41	7.40	7.91
HPV16/18	Opportunistic	Montpellier	49	4.98	3.11	3.33	3.76	4.89	6.27	7.24	7.73
HPV16/18	Opportunistic	Montpellier	50	4.87	3.04	3.25	3.69	4.78	6.17	7.07	7.52
HPV16/18	Opportunistic	Montpellier	51	4.76	2.96	3.19	3.61	4.67	6.05	6.90	7.36
HPV16/18	Opportunistic	Montpellier	52	4.66	2.91	3.12	3.53	4.56	5.93	6.77	7.21

Table S9 Posterior expected HR HPV cervical infection prevalence (in %) in major French cities, stratified by type of test, city, and age. Table counterpart to Figure 3A. ***(continued)***

				Posterior distribution							
					Percentiles						
Virus	Test	City	Age	Average	0.01	0.025	0.1	0.5	0.9	0.975	0.99
HPV16/18	Opportunistic	Montpellier	53	4.55	2.83	3.04	3.45	4.44	5.78	6.59	7.01
HPV16/18	Opportunistic	Montpellier	54	4.43	2.75	2.95	3.36	4.33	5.64	6.42	6.86
HPV16/18	Opportunistic	Montpellier	55	4.34	2.67	2.88	3.28	4.24	5.52	6.26	6.74
HPV16/18	Opportunistic	Montpellier	56	4.26	2.64	2.83	3.22	4.17	5.42	6.15	6.67
HPV16/18	Opportunistic	Montpellier	57	4.21	2.59	2.80	3.18	4.12	5.36	6.08	6.59
HPV16/18	Opportunistic	Montpellier	58	4.17	2.60	2.77	3.14	4.09	5.30	6.07	6.56
HPV16/18	Opportunistic	Montpellier	59	4.16	2.59	2.76	3.13	4.08	5.27	6.04	6.55
HPV16/18	Opportunistic	Montpellier	60	4.16	2.61	2.77	3.15	4.08	5.27	6.06	6.52
HPV16/18	Opportunistic	Montpellier	61	4.17	2.61	2.77	3.14	4.07	5.28	6.07	6.58
HPV16/18	Opportunistic	Montpellier	62	4.16	2.60	2.77	3.15	4.08	5.27	6.05	6.59
HPV16/18	Opportunistic	Montpellier	63	4.15	2.59	2.75	3.13	4.06	5.25	6.01	6.61
HPV16/18	Opportunistic	Montpellier	64	4.12	2.54	2.71	3.11	4.04	5.23	5.99	6.58
HPV16/18	Opportunistic	Montpellier	65	4.08	2.55	2.67	3.06	3.99	5.20	5.93	6.49
HPV16/18	Opportunistic	Montpellier	66	4.03	2.45	2.61	2.99	3.94	5.15	5.92	6.44
HPV16/18	Opportunistic	Rennes	30	6.43	4.84	5.07	5.48	6.38	7.43	8.10	8.46
HPV16/18	Opportunistic	Rennes	31	6.79	5.17	5.36	5.81	6.75	7.82	8.53	8.88
HPV16/18	Opportunistic	Rennes	32	7.12	5.44	5.62	6.10	7.08	8.20	8.93	9.29
HPV16/18	Opportunistic	Rennes	33	7.38	5.63	5.82	6.31	7.35	8.48	9.24	9.59
HPV16/18	Opportunistic	Rennes	34	7.48	5.71	5.90	6.40	7.44	8.60	9.37	9.77
HPV16/18	Opportunistic	Rennes	35	7.42	5.67	5.85	6.36	7.38	8.52	9.28	9.67
HPV16/18	Opportunistic	Rennes	36	7.28	5.55	5.76	6.23	7.24	8.36	9.12	9.56
HPV16/18	Opportunistic	Rennes	37	7.09	5.40	5.61	6.07	7.06	8.15	8.87	9.29
HPV16/18	Opportunistic	Rennes	38	6.91	5.28	5.46	5.91	6.86	7.96	8.66	9.04
HPV16/18	Opportunistic	Rennes	39	6.73	5.11	5.32	5.74	6.68	7.75	8.47	8.80
HPV16/18	Opportunistic	Rennes	40	6.55	4.97	5.17	5.59	6.49	7.54	8.23	8.57
HPV16/18	Opportunistic	Rennes	41	6.37	4.83	5.03	5.43	6.33	7.35	8.01	8.35
HPV16/18	Opportunistic	Rennes	42	6.19	4.68	4.88	5.28	6.14	7.14	7.77	8.10
HPV16/18	Opportunistic	Rennes	43	6.02	4.56	4.74	5.14	5.98	6.94	7.54	7.86
HPV16/18	Opportunistic	Rennes	44	5.87	4.43	4.61	5.02	5.83	6.77	7.37	7.69

Table S9 Posterior expected HR HPV cervical infection prevalence (in %) in major French cities, stratified by type of test, city, and age. Table counterpart to Figure 3A. ***(continued)***

				Posterior distribution							
					Percentiles						
Virus	Test	City	Age	Average	0.01	0.025	0.1	0.5	0.9	0.975	0.99
HPV16/18	Opportunistic	Rennes	45	5.75	4.34	4.50	4.91	5.72	6.61	7.21	7.53
HPV16/18	Opportunistic	Rennes	46	5.61	4.23	4.40	4.80	5.58	6.47	7.05	7.40
HPV16/18	Opportunistic	Rennes	47	5.48	4.13	4.30	4.69	5.44	6.33	6.91	7.22
HPV16/18	Opportunistic	Rennes	48	5.36	4.06	4.20	4.57	5.32	6.20	6.74	7.06
HPV16/18	Opportunistic	Rennes	49	5.24	3.99	4.11	4.48	5.20	6.05	6.62	6.91
HPV16/18	Opportunistic	Rennes	50	5.12	3.87	4.02	4.38	5.09	5.91	6.47	6.78
HPV16/18	Opportunistic	Rennes	51	5.01	3.79	3.93	4.28	4.98	5.78	6.33	6.61
HPV16/18	Opportunistic	Rennes	52	4.91	3.70	3.85	4.18	4.88	5.67	6.19	6.45
HPV16/18	Opportunistic	Rennes	53	4.79	3.60	3.75	4.07	4.75	5.54	6.02	6.33
HPV16/18	Opportunistic	Rennes	54	4.67	3.52	3.66	3.96	4.63	5.40	5.89	6.14
HPV16/18	Opportunistic	Rennes	55	4.56	3.43	3.58	3.89	4.53	5.29	5.76	6.03
HPV16/18	Opportunistic	Rennes	56	4.48	3.39	3.52	3.82	4.45	5.19	5.65	5.90
HPV16/18	Opportunistic	Rennes	57	4.43	3.34	3.47	3.77	4.39	5.13	5.60	5.84
HPV16/18	Opportunistic	Rennes	58	4.39	3.30	3.43	3.74	4.36	5.10	5.56	5.76
HPV16/18	Opportunistic	Rennes	59	4.38	3.29	3.43	3.72	4.34	5.09	5.54	5.80
HPV16/18	Opportunistic	Rennes	60	4.38	3.30	3.44	3.73	4.34	5.09	5.56	5.77
HPV16/18	Opportunistic	Rennes	61	4.39	3.30	3.46	3.73	4.35	5.09	5.57	5.75
HPV16/18	Opportunistic	Rennes	62	4.38	3.29	3.43	3.72	4.35	5.07	5.55	5.77
HPV16/18	Opportunistic	Rennes	63	4.36	3.27	3.41	3.71	4.33	5.06	5.51	5.79
HPV16/18	Opportunistic	Rennes	64	4.33	3.23	3.36	3.68	4.30	5.03	5.48	5.79
HPV16/18	Opportunistic	Rennes	65	4.29	3.19	3.31	3.63	4.26	5.01	5.46	5.75
HPV16/18	Opportunistic	Rennes	66	4.24	3.08	3.22	3.55	4.20	4.98	5.45	5.76
HPV16/18	Opportunistic	Nantes	30	5.47	3.31	3.52	4.04	5.34	7.10	8.08	8.70
HPV16/18	Opportunistic	Nantes	31	5.78	3.47	3.77	4.30	5.66	7.46	8.53	9.25
HPV16/18	Opportunistic	Nantes	32	6.06	3.64	3.99	4.51	5.94	7.81	8.94	9.68
HPV16/18	Opportunistic	Nantes	33	6.28	3.82	4.13	4.67	6.13	8.06	9.24	9.95
HPV16/18	Opportunistic	Nantes	34	6.37	3.87	4.17	4.72	6.21	8.19	9.38	10.05
HPV16/18	Opportunistic	Nantes	35	6.32	3.85	4.14	4.69	6.17	8.13	9.32	9.98
HPV16/18	Opportunistic	Nantes	36	6.19	3.79	4.04	4.60	6.05	7.97	9.13	9.86

Table S9 **Posterior expected HR HPV cervical infection prevalence (in %) in major French cities, stratified by type of test, city, and age. Table counterpart to Figure 3A.** ***(continued)***

				Posterior distribution							
					Percentiles						
Virus	Test	City	Age	Average	0.01	0.025	0.1	0.5	0.9	0.975	0.99
HPV16/18	Opportunistic	Nantes	37	6.03	3.72	3.95	4.48	5.90	7.77	8.89	9.55
HPV16/18	Opportunistic	Nantes	38	5.88	3.60	3.85	4.36	5.74	7.59	8.69	9.36
HPV16/18	Opportunistic	Nantes	39	5.72	3.48	3.72	4.25	5.58	7.39	8.47	9.16
HPV16/18	Opportunistic	Nantes	40	5.57	3.39	3.61	4.13	5.43	7.20	8.23	8.79
HPV16/18	Opportunistic	Nantes	41	5.42	3.28	3.52	4.01	5.28	6.99	8.02	8.60
HPV16/18	Opportunistic	Nantes	42	5.26	3.18	3.44	3.90	5.13	6.80	7.79	8.31
HPV16/18	Opportunistic	Nantes	43	5.11	3.11	3.32	3.78	5.00	6.60	7.57	8.16
HPV16/18	Opportunistic	Nantes	44	4.99	3.02	3.25	3.69	4.87	6.45	7.40	7.93
HPV16/18	Opportunistic	Nantes	45	4.88	2.95	3.17	3.61	4.76	6.33	7.23	7.78
HPV16/18	Opportunistic	Nantes	46	4.76	2.88	3.10	3.53	4.65	6.20	7.07	7.63
HPV16/18	Opportunistic	Nantes	47	4.65	2.80	3.02	3.45	4.54	6.06	6.91	7.50
HPV16/18	Opportunistic	Nantes	48	4.55	2.73	2.94	3.36	4.43	5.91	6.75	7.26
HPV16/18	Opportunistic	Nantes	49	4.45	2.69	2.88	3.29	4.33	5.76	6.60	7.12
HPV16/18	Opportunistic	Nantes	50	4.35	2.62	2.81	3.22	4.24	5.64	6.46	6.97
HPV16/18	Opportunistic	Nantes	51	4.25	2.57	2.75	3.14	4.15	5.54	6.32	6.83
HPV16/18	Opportunistic	Nantes	52	4.16	2.51	2.70	3.08	4.06	5.41	6.16	6.70
HPV16/18	Opportunistic	Nantes	53	4.06	2.44	2.62	2.99	3.96	5.27	6.03	6.56
HPV16/18	Opportunistic	Nantes	54	3.96	2.37	2.54	2.92	3.86	5.14	5.87	6.37
HPV16/18	Opportunistic	Nantes	55	3.87	2.32	2.48	2.85	3.77	5.02	5.73	6.22
HPV16/18	Opportunistic	Nantes	56	3.80	2.26	2.44	2.80	3.71	4.93	5.64	6.13
HPV16/18	Opportunistic	Nantes	57	3.76	2.23	2.41	2.76	3.66	4.88	5.60	6.09
HPV16/18	Opportunistic	Nantes	58	3.73	2.22	2.39	2.74	3.62	4.83	5.55	6.11
HPV16/18	Opportunistic	Nantes	59	3.71	2.20	2.38	2.73	3.62	4.82	5.56	6.08
HPV16/18	Opportunistic	Nantes	60	3.71	2.22	2.39	2.73	3.63	4.82	5.55	6.05
HPV16/18	Opportunistic	Nantes	61	3.72	2.23	2.40	2.73	3.63	4.84	5.58	6.05
HPV16/18	Opportunistic	Nantes	62	3.72	2.23	2.39	2.73	3.63	4.81	5.55	6.01
HPV16/18	Opportunistic	Nantes	63	3.70	2.20	2.39	2.72	3.62	4.78	5.53	5.98
HPV16/18	Opportunistic	Nantes	64	3.67	2.20	2.36	2.69	3.59	4.75	5.53	5.96
HPV16/18	Opportunistic	Nantes	65	3.64	2.18	2.33	2.66	3.55	4.74	5.52	5.94

Table S9 **Posterior expected HR HPV cervical infection prevalence (in %) in major French cities, stratified by type of test, city, and age. Table counterpart to Figure 3A.** ***(continued)***

				Posterior distribution							
					Percentiles						
Virus	Test	City	Age	Average	0.01	0.025	0.1	0.5	0.9	0.975	0.99
HPV16/18	Opportunistic	Nantes	66	3.60	2.14	2.27	2.62	3.51	4.67	5.46	5.90
HPV16/18	Opportunistic	Lille	30	5.83	3.73	4.01	4.54	5.74	7.25	8.11	8.62
HPV16/18	Opportunistic	Lille	31	6.17	3.94	4.25	4.82	6.08	7.62	8.57	9.04
HPV16/18	Opportunistic	Lille	32	6.47	4.14	4.44	5.06	6.38	8.00	8.96	9.50
HPV16/18	Opportunistic	Lille	33	6.70	4.28	4.61	5.24	6.59	8.30	9.25	9.80
HPV16/18	Opportunistic	Lille	34	6.79	4.35	4.68	5.32	6.68	8.41	9.42	9.97
HPV16/18	Opportunistic	Lille	35	6.74	4.28	4.66	5.28	6.64	8.34	9.38	9.90
HPV16/18	Opportunistic	Lille	36	6.61	4.22	4.57	5.18	6.50	8.17	9.20	9.71
HPV16/18	Opportunistic	Lille	37	6.44	4.11	4.42	5.03	6.34	7.94	8.97	9.52
HPV16/18	Opportunistic	Lille	38	6.27	4.04	4.34	4.91	6.17	7.75	8.72	9.28
HPV16/18	Opportunistic	Lille	39	6.11	3.93	4.22	4.77	6.00	7.55	8.49	9.01
HPV16/18	Opportunistic	Lille	40	5.94	3.83	4.12	4.64	5.85	7.36	8.28	8.77
HPV16/18	Opportunistic	Lille	41	5.78	3.68	3.99	4.51	5.70	7.19	8.04	8.55
HPV16/18	Opportunistic	Lille	42	5.61	3.56	3.86	4.37	5.53	6.98	7.81	8.34
HPV16/18	Opportunistic	Lille	43	5.46	3.44	3.74	4.25	5.37	6.78	7.64	8.15
HPV16/18	Opportunistic	Lille	44	5.32	3.36	3.64	4.14	5.24	6.62	7.48	7.92
HPV16/18	Opportunistic	Lille	45	5.21	3.29	3.56	4.06	5.12	6.47	7.31	7.72
HPV16/18	Opportunistic	Lille	46	5.09	3.22	3.49	3.97	5.00	6.32	7.15	7.54
HPV16/18	Opportunistic	Lille	47	4.97	3.17	3.41	3.88	4.89	6.16	6.96	7.43
HPV16/18	Opportunistic	Lille	48	4.86	3.05	3.32	3.80	4.78	6.02	6.78	7.22
HPV16/18	Opportunistic	Lille	49	4.75	3.00	3.26	3.72	4.68	5.91	6.64	7.05
HPV16/18	Opportunistic	Lille	50	4.64	2.95	3.17	3.63	4.58	5.77	6.48	6.92
HPV16/18	Opportunistic	Lille	51	4.54	2.89	3.11	3.54	4.47	5.65	6.37	6.80
HPV16/18	Opportunistic	Lille	52	4.45	2.82	3.05	3.46	4.37	5.51	6.21	6.63
HPV16/18	Opportunistic	Lille	53	4.34	2.73	2.97	3.37	4.27	5.38	6.08	6.48
HPV16/18	Opportunistic	Lille	54	4.23	2.67	2.90	3.29	4.16	5.27	5.92	6.29
HPV16/18	Opportunistic	Lille	55	4.13	2.59	2.84	3.21	4.06	5.14	5.81	6.16
HPV16/18	Opportunistic	Lille	56	4.06	2.55	2.79	3.16	3.99	5.07	5.73	6.08
HPV16/18	Opportunistic	Lille	57	4.01	2.51	2.77	3.11	3.93	5.01	5.66	5.96

Table S9 Posterior expected HR HPV cervical infection prevalence (in %) in major French cities, stratified by type of test, city, and age. Table counterpart to Figure 3A. ***(continued)***

					Posterior distribution							
					Percentiles							
Virus	Test	City	Age	Average	0.01	0.025	0.1	0.5	0.9	0.975	0.99	
HPV16/18	Opportunistic	Lille	58	3.98	2.50	2.75	3.09	3.90	4.97	5.61	5.89	
HPV16/18	Opportunistic	Lille	59	3.97	2.47	2.74	3.07	3.90	4.94	5.59	5.88	
HPV16/18	Opportunistic	Lille	60	3.97	2.50	2.74	3.08	3.90	4.95	5.61	5.91	
HPV16/18	Opportunistic	Lille	61	3.97	2.52	2.73	3.07	3.91	4.96	5.60	5.93	
HPV16/18	Opportunistic	Lille	62	3.97	2.52	2.72	3.07	3.91	4.95	5.60	5.89	
HPV16/18	Opportunistic	Lille	63	3.95	2.51	2.72	3.06	3.89	4.93	5.55	5.89	
HPV16/18	Opportunistic	Lille	64	3.92	2.49	2.69	3.03	3.86	4.91	5.52	5.94	
HPV16/18	Opportunistic	Lille	65	3.89	2.46	2.65	3.00	3.82	4.87	5.48	5.95	
HPV16/18	Opportunistic	Lille	66	3.84	2.38	2.59	2.94	3.76	4.84	5.50	5.91	
HPV16/18	Opportunistic	Strasbourg	30	4.66	2.62	2.85	3.35	4.53	6.14	7.25	7.86	
HPV16/18	Opportunistic	Strasbourg	31	4.93	2.75	3.04	3.54	4.80	6.49	7.62	8.37	
HPV16/18	Opportunistic	Strasbourg	32	5.17	2.90	3.18	3.71	5.05	6.80	7.98	8.76	
HPV16/18	Opportunistic	Strasbourg	33	5.36	2.98	3.29	3.84	5.22	7.05	8.28	9.01	
HPV16/18	Opportunistic	Strasbourg	34	5.44	3.04	3.34	3.88	5.29	7.14	8.37	9.20	
HPV16/18	Opportunistic	Strasbourg	35	5.39	3.03	3.33	3.85	5.24	7.09	8.33	9.14	
HPV16/18	Opportunistic	Strasbourg	36	5.29	2.97	3.26	3.78	5.14	6.93	8.18	8.96	
HPV16/18	Opportunistic	Strasbourg	37	5.15	2.87	3.17	3.69	5.00	6.74	7.98	8.81	
HPV16/18	Opportunistic	Strasbourg	38	5.01	2.78	3.08	3.59	4.87	6.55	7.79	8.59	
HPV16/18	Opportunistic	Strasbourg	39	4.88	2.70	2.99	3.50	4.74	6.40	7.58	8.26	
HPV16/18	Opportunistic	Strasbourg	40	4.75	2.64	2.90	3.40	4.62	6.22	7.33	8.00	
HPV16/18	Opportunistic	Strasbourg	41	4.62	2.56	2.83	3.30	4.49	6.06	7.18	7.76	
HPV16/18	Opportunistic	Strasbourg	42	4.48	2.49	2.74	3.20	4.36	5.90	6.98	7.52	
HPV16/18	Opportunistic	Strasbourg	43	4.36	2.40	2.66	3.10	4.24	5.74	6.78	7.36	
HPV16/18	Opportunistic	Strasbourg	44	4.25	2.35	2.60	3.02	4.13	5.58	6.57	7.23	
HPV16/18	Opportunistic	Strasbourg	45	4.16	2.29	2.55	2.97	4.03	5.47	6.50	7.05	
HPV16/18	Opportunistic	Strasbourg	46	4.06	2.25	2.47	2.90	3.94	5.32	6.32	6.93	
HPV16/18	Opportunistic	Strasbourg	47	3.96	2.20	2.41	2.82	3.84	5.18	6.20	6.82	
HPV16/18	Opportunistic	Strasbourg	48	3.87	2.13	2.36	2.75	3.75	5.06	6.06	6.63	
HPV16/18	Opportunistic	Strasbourg	49	3.78	2.08	2.30	2.69	3.67	4.96	5.91	6.45	

Table S9 **Posterior expected HR HPV cervical infection prevalence (in %) in major French cities, stratified by type of test, city, and age. Table counterpart to Figure 3A.** ***(continued)***

				Posterior distribution							
					Percentiles						
Virus	Test	City	Age	Average	0.01	0.025	0.1	0.5	0.9	0.975	0.99
HPV16/18	Opportunistic	Strasbourg	50	3.70	2.05	2.25	2.63	3.58	4.86	5.79	6.30
HPV16/18	Opportunistic	Strasbourg	51	3.62	2.00	2.21	2.57	3.51	4.76	5.65	6.12
HPV16/18	Opportunistic	Strasbourg	52	3.54	1.95	2.15	2.51	3.43	4.67	5.54	6.10
HPV16/18	Opportunistic	Strasbourg	53	3.46	1.90	2.10	2.45	3.35	4.54	5.43	5.97
HPV16/18	Opportunistic	Strasbourg	54	3.37	1.84	2.06	2.38	3.26	4.43	5.26	5.84
HPV16/18	Opportunistic	Strasbourg	55	3.29	1.79	2.00	2.34	3.19	4.34	5.15	5.70
HPV16/18	Opportunistic	Strasbourg	56	3.23	1.75	1.97	2.30	3.14	4.25	5.04	5.54
HPV16/18	Opportunistic	Strasbourg	57	3.19	1.74	1.94	2.27	3.10	4.21	5.00	5.47
HPV16/18	Opportunistic	Strasbourg	58	3.17	1.73	1.93	2.25	3.08	4.18	4.96	5.42
HPV16/18	Opportunistic	Strasbourg	59	3.16	1.71	1.92	2.24	3.06	4.17	4.97	5.42
HPV16/18	Opportunistic	Strasbourg	60	3.16	1.74	1.93	2.24	3.06	4.16	4.98	5.42
HPV16/18	Opportunistic	Strasbourg	61	3.16	1.74	1.93	2.26	3.07	4.17	4.97	5.36
HPV16/18	Opportunistic	Strasbourg	62	3.16	1.75	1.92	2.25	3.06	4.17	4.98	5.46
HPV16/18	Opportunistic	Strasbourg	63	3.14	1.75	1.90	2.25	3.04	4.15	4.94	5.39
HPV16/18	Opportunistic	Strasbourg	64	3.12	1.72	1.88	2.23	3.02	4.14	4.91	5.37
HPV16/18	Opportunistic	Strasbourg	65	3.09	1.70	1.85	2.20	3.00	4.12	4.86	5.33
HPV16/18	Opportunistic	Strasbourg	66	3.06	1.66	1.80	2.16	2.97	4.10	4.84	5.36
HPV16/18	Opportunistic	Lyon	30	6.32	3.82	4.13	4.75	6.20	8.07	9.39	9.89
HPV16/18	Opportunistic	Lyon	31	6.68	4.05	4.36	5.02	6.55	8.52	9.81	10.54
HPV16/18	Opportunistic	Lyon	32	7.01	4.25	4.62	5.29	6.85	8.93	10.24	10.94
HPV16/18	Opportunistic	Lyon	33	7.25	4.40	4.77	5.47	7.08	9.24	10.56	11.42
HPV16/18	Opportunistic	Lyon	34	7.35	4.49	4.85	5.54	7.19	9.39	10.69	11.55
HPV16/18	Opportunistic	Lyon	35	7.30	4.44	4.77	5.50	7.12	9.31	10.62	11.46
HPV16/18	Opportunistic	Lyon	36	7.16	4.33	4.68	5.39	7.00	9.13	10.47	11.23
HPV16/18	Opportunistic	Lyon	37	6.98	4.23	4.57	5.24	6.82	8.92	10.20	10.91
HPV16/18	Opportunistic	Lyon	38	6.80	4.13	4.45	5.10	6.65	8.72	9.95	10.64
HPV16/18	Opportunistic	Lyon	39	6.62	4.03	4.33	4.96	6.47	8.48	9.71	10.38
HPV16/18	Opportunistic	Lyon	40	6.44	3.90	4.22	4.84	6.29	8.24	9.47	10.12
HPV16/18	Opportunistic	Lyon	41	6.27	3.79	4.08	4.70	6.13	8.02	9.19	9.86

Table S9 Posterior expected HR HPV cervical infection prevalence (in %) in major French cities, stratified by type of test, city, and age. Table counterpart to Figure 3A. *(continued)*

				Posterior distribution							
					Percentiles						
Virus	Test	City	Age	Average	0.01	0.025	0.1	0.5	0.9	0.975	0.99
HPV16/18	Opportunistic	Lyon	42	6.09	3.68	3.98	4.57	5.95	7.79	8.96	9.56
HPV16/18	Opportunistic	Lyon	43	5.92	3.57	3.86	4.44	5.78	7.59	8.72	9.32
HPV16/18	Opportunistic	Lyon	44	5.77	3.48	3.78	4.34	5.63	7.37	8.48	9.15
HPV16/18	Opportunistic	Lyon	45	5.65	3.41	3.69	4.24	5.51	7.22	8.30	9.00
HPV16/18	Opportunistic	Lyon	46	5.52	3.31	3.60	4.14	5.38	7.07	8.12	8.72
HPV16/18	Opportunistic	Lyon	47	5.39	3.24	3.51	4.04	5.27	6.93	7.94	8.48
HPV16/18	Opportunistic	Lyon	48	5.27	3.15	3.43	3.93	5.15	6.75	7.81	8.30
HPV16/18	Opportunistic	Lyon	49	5.15	3.12	3.35	3.84	5.03	6.61	7.62	8.10
HPV16/18	Opportunistic	Lyon	50	5.04	3.04	3.29	3.76	4.92	6.47	7.43	7.91
HPV16/18	Opportunistic	Lyon	51	4.93	2.97	3.22	3.67	4.82	6.34	7.28	7.75
HPV16/18	Opportunistic	Lyon	52	4.83	2.91	3.15	3.61	4.72	6.21	7.12	7.59
HPV16/18	Opportunistic	Lyon	53	4.71	2.83	3.06	3.53	4.60	6.05	6.94	7.43
HPV16/18	Opportunistic	Lyon	54	4.59	2.78	2.98	3.43	4.48	5.89	6.79	7.24
HPV16/18	Opportunistic	Lyon	55	4.49	2.71	2.91	3.36	4.38	5.75	6.68	7.11
HPV16/18	Opportunistic	Lyon	56	4.41	2.67	2.86	3.29	4.31	5.66	6.57	7.02
HPV16/18	Opportunistic	Lyon	57	4.36	2.63	2.82	3.25	4.24	5.59	6.49	6.91
HPV16/18	Opportunistic	Lyon	58	4.32	2.60	2.79	3.22	4.21	5.56	6.48	6.84
HPV16/18	Opportunistic	Lyon	59	4.31	2.57	2.78	3.22	4.21	5.56	6.41	6.86
HPV16/18	Opportunistic	Lyon	60	4.31	2.57	2.77	3.21	4.21	5.54	6.41	6.86
HPV16/18	Opportunistic	Lyon	61	4.32	2.58	2.78	3.22	4.22	5.56	6.45	6.88
HPV16/18	Opportunistic	Lyon	62	4.31	2.58	2.79	3.21	4.22	5.54	6.46	6.87
HPV16/18	Opportunistic	Lyon	63	4.29	2.57	2.77	3.18	4.20	5.53	6.43	6.87
HPV16/18	Opportunistic	Lyon	64	4.26	2.55	2.77	3.16	4.16	5.48	6.40	6.78
HPV16/18	Opportunistic	Lyon	65	4.22	2.50	2.73	3.13	4.11	5.47	6.38	6.74
HPV16/18	Opportunistic	Lyon	66	4.18	2.42	2.65	3.06	4.05	5.43	6.35	6.71
HPV16/18	Opportunistic	Paris	30	6.70	5.01	5.23	5.69	6.65	7.79	8.41	8.71
HPV16/18	Opportunistic	Paris	31	7.08	5.29	5.59	6.04	7.02	8.17	8.84	9.12
HPV16/18	Opportunistic	Paris	32	7.42	5.61	5.87	6.35	7.37	8.56	9.25	9.53
HPV16/18	Opportunistic	Paris	33	7.69	5.77	6.07	6.58	7.63	8.85	9.58	9.87

Table S9 Posterior expected HR HPV cervical infection prevalence (in %) in major French cities, stratified by type of test, city, and age. Table counterpart to Figure 3A. ***(continued)***

				Posterior distribution							
					Percentiles						
Virus	Test	City	Age	Average	0.01	0.025	0.1	0.5	0.9	0.975	0.99
HPV16/18	Opportunistic	Paris	34	7.79	5.91	6.17	6.67	7.73	8.99	9.71	10.01
HPV16/18	Opportunistic	Paris	35	7.73	5.84	6.11	6.60	7.68	8.93	9.64	9.92
HPV16/18	Opportunistic	Paris	36	7.58	5.70	6.03	6.48	7.53	8.73	9.50	9.75
HPV16/18	Opportunistic	Paris	37	7.39	5.53	5.86	6.31	7.34	8.52	9.24	9.56
HPV16/18	Opportunistic	Paris	38	7.20	5.38	5.70	6.16	7.15	8.31	9.00	9.30
HPV16/18	Opportunistic	Paris	39	7.01	5.23	5.52	6.00	6.97	8.10	8.78	9.06
HPV16/18	Opportunistic	Paris	40	6.82	5.12	5.38	5.83	6.78	7.87	8.54	8.86
HPV16/18	Opportunistic	Paris	41	6.64	4.98	5.23	5.66	6.60	7.68	8.29	8.62
HPV16/18	Opportunistic	Paris	42	6.45	4.84	5.08	5.49	6.41	7.47	8.05	8.38
HPV16/18	Opportunistic	Paris	43	6.27	4.71	4.92	5.35	6.24	7.26	7.83	8.14
HPV16/18	Opportunistic	Paris	44	6.12	4.60	4.80	5.21	6.08	7.09	7.66	7.88
HPV16/18	Opportunistic	Paris	45	5.99	4.49	4.70	5.10	5.95	6.96	7.51	7.73
HPV16/18	Opportunistic	Paris	46	5.85	4.38	4.58	4.98	5.81	6.78	7.34	7.61
HPV16/18	Opportunistic	Paris	47	5.71	4.28	4.47	4.87	5.67	6.62	7.17	7.40
HPV16/18	Opportunistic	Paris	48	5.59	4.21	4.36	4.76	5.55	6.47	7.01	7.25
HPV16/18	Opportunistic	Paris	49	5.46	4.09	4.26	4.65	5.42	6.34	6.85	7.09
HPV16/18	Opportunistic	Paris	50	5.34	3.99	4.18	4.55	5.31	6.21	6.68	6.93
HPV16/18	Opportunistic	Paris	51	5.23	3.89	4.09	4.46	5.19	6.07	6.55	6.77
HPV16/18	Opportunistic	Paris	52	5.12	3.82	4.00	4.36	5.08	5.94	6.43	6.66
HPV16/18	Opportunistic	Paris	53	4.99	3.74	3.90	4.25	4.96	5.79	6.29	6.49
HPV16/18	Opportunistic	Paris	54	4.87	3.65	3.79	4.14	4.84	5.63	6.14	6.34
HPV16/18	Opportunistic	Paris	55	4.76	3.57	3.71	4.04	4.73	5.51	5.99	6.21
HPV16/18	Opportunistic	Paris	56	4.68	3.49	3.63	3.96	4.65	5.42	5.89	6.08
HPV16/18	Opportunistic	Paris	57	4.62	3.44	3.59	3.91	4.60	5.37	5.83	6.01
HPV16/18	Opportunistic	Paris	58	4.58	3.43	3.56	3.88	4.56	5.33	5.79	5.96
HPV16/18	Opportunistic	Paris	59	4.57	3.39	3.55	3.86	4.55	5.32	5.78	5.97
HPV16/18	Opportunistic	Paris	60	4.57	3.38	3.56	3.86	4.54	5.32	5.78	5.97
HPV16/18	Opportunistic	Paris	61	4.58	3.41	3.57	3.88	4.55	5.33	5.80	6.01
HPV16/18	Opportunistic	Paris	62	4.57	3.40	3.57	3.88	4.54	5.32	5.79	6.00

Table S9 Posterior expected HR HPV cervical infection prevalence (in %) in major French cities, stratified by type of test, city, and age. Table counterpart to Figure 3A. ***(continued)***

				Posterior distribution							
					Percentiles						
Virus	Test	City	Age	Average	0.01	0.025	0.1	0.5	0.9	0.975	0.99
HPV16/18	Opportunistic	Paris	63	4.55	3.38	3.55	3.86	4.52	5.30	5.77	5.96
HPV16/18	Opportunistic	Paris	64	4.52	3.35	3.51	3.81	4.49	5.27	5.75	5.91
HPV16/18	Opportunistic	Paris	65	4.48	3.28	3.44	3.76	4.44	5.26	5.70	5.96
HPV16/18	Opportunistic	Paris	66	4.43	3.18	3.35	3.69	4.39	5.23	5.70	5.94
Other genotypes	Organised	Nice	30	12.88	6.80	7.47	8.98	12.49	17.12	20.26	22.18
Other genotypes	Organised	Nice	31	12.26	6.51	7.16	8.52	11.90	16.33	19.41	21.17
Other genotypes	Organised	Nice	32	11.61	6.23	6.78	8.06	11.28	15.51	18.40	20.01
Other genotypes	Organised	Nice	33	10.96	5.84	6.36	7.61	10.69	14.65	17.26	18.88
Other genotypes	Organised	Nice	34	10.42	5.53	6.01	7.20	10.16	13.91	16.36	18.04
Other genotypes	Organised	Nice	35	9.98	5.28	5.79	6.93	9.70	13.36	15.81	17.30
Other genotypes	Organised	Nice	36	9.60	5.07	5.56	6.68	9.33	12.86	15.23	16.72
Other genotypes	Organised	Nice	37	9.29	4.91	5.36	6.42	9.01	12.46	14.81	16.18
Other genotypes	Organised	Nice	38	9.01	4.74	5.19	6.23	8.73	12.06	14.40	15.72
Other genotypes	Organised	Nice	39	8.76	4.63	5.05	6.06	8.48	11.70	14.02	15.29
Other genotypes	Organised	Nice	40	8.50	4.47	4.91	5.90	8.24	11.40	13.68	14.89
Other genotypes	Organised	Nice	41	8.28	4.37	4.74	5.73	8.03	11.10	13.36	14.53
Other genotypes	Organised	Nice	42	8.10	4.25	4.63	5.58	7.84	10.88	13.08	14.20
Other genotypes	Organised	Nice	43	7.95	4.18	4.57	5.48	7.69	10.68	12.91	13.86
Other genotypes	Organised	Nice	44	7.84	4.11	4.51	5.39	7.58	10.53	12.72	13.69
Other genotypes	Organised	Nice	45	7.74	4.08	4.42	5.33	7.49	10.40	12.53	13.47
Other genotypes	Organised	Nice	46	7.67	4.04	4.38	5.29	7.41	10.30	12.41	13.39
Other genotypes	Organised	Nice	47	7.62	4.00	4.32	5.25	7.36	10.20	12.30	13.27
Other genotypes	Organised	Nice	48	7.59	3.98	4.31	5.21	7.35	10.19	12.25	13.28
Other genotypes	Organised	Nice	49	7.57	3.97	4.34	5.20	7.31	10.18	12.31	13.36
Other genotypes	Organised	Nice	50	7.55	3.99	4.33	5.18	7.29	10.15	12.25	13.12
Other genotypes	Organised	Nice	51	7.53	3.95	4.30	5.17	7.26	10.12	12.27	13.13
Other genotypes	Organised	Nice	52	7.48	3.92	4.27	5.14	7.21	10.03	12.15	13.07
Other genotypes	Organised	Nice	53	7.41	3.89	4.23	5.10	7.16	9.95	12.03	12.87
Other genotypes	Organised	Nice	54	7.30	3.80	4.15	5.02	7.05	9.83	11.79	12.73

Table S9 **Posterior expected HR HPV cervical infection prevalence (in %) in major French cities, stratified by type of test, city, and age. Table counterpart to Figure 3A.** ***(continued)***

				Posterior distribution							
					Percentiles						
Virus	Test	City	Age	Average	0.01	0.025	0.1	0.5	0.9	0.975	0.99
Other genotypes	Organised	Nice	55	7.21	3.76	4.10	4.95	6.96	9.71	11.62	12.64
Other genotypes	Organised	Nice	56	7.12	3.72	4.07	4.89	6.89	9.62	11.46	12.51
Other genotypes	Organised	Nice	57	7.03	3.67	4.01	4.82	6.79	9.49	11.31	12.42
Other genotypes	Organised	Nice	58	6.95	3.60	3.99	4.75	6.71	9.40	11.14	12.23
Other genotypes	Organised	Nice	59	6.89	3.53	3.96	4.72	6.65	9.36	11.08	12.21
Other genotypes	Organised	Nice	60	6.87	3.52	3.95	4.70	6.62	9.30	11.03	12.22
Other genotypes	Organised	Nice	61	6.88	3.56	3.94	4.71	6.63	9.31	11.06	12.19
Other genotypes	Organised	Nice	62	6.92	3.58	3.96	4.74	6.70	9.36	11.12	12.29
Other genotypes	Organised	Nice	63	7.01	3.63	4.02	4.78	6.79	9.50	11.20	12.59
Other genotypes	Organised	Nice	64	7.15	3.68	4.04	4.87	6.91	9.69	11.45	12.89
Other genotypes	Organised	Nice	65	7.32	3.74	4.12	4.98	7.07	9.98	11.82	13.18
Other genotypes	Organised	Nice	66	7.53	3.83	4.20	5.11	7.26	10.35	12.27	13.65
Other genotypes	Organised	Marseille	30	14.84	10.58	11.17	12.20	14.72	17.67	19.42	20.45
Other genotypes	Organised	Marseille	31	14.14	10.16	10.68	11.65	14.01	16.82	18.52	19.44
Other genotypes	Organised	Marseille	32	13.41	9.67	10.13	11.03	13.27	15.97	17.55	18.36
Other genotypes	Organised	Marseille	33	12.67	9.10	9.54	10.40	12.52	15.10	16.58	17.33
Other genotypes	Organised	Marseille	34	12.04	8.58	9.08	9.89	11.91	14.33	15.81	16.50
Other genotypes	Organised	Marseille	35	11.55	8.21	8.67	9.47	11.43	13.76	15.19	15.83
Other genotypes	Organised	Marseille	36	11.11	7.90	8.32	9.12	11.00	13.31	14.65	15.23
Other genotypes	Organised	Marseille	37	10.76	7.64	8.03	8.81	10.65	12.88	14.23	14.75
Other genotypes	Organised	Marseille	38	10.45	7.40	7.79	8.54	10.33	12.48	13.84	14.36
Other genotypes	Organised	Marseille	39	10.15	7.22	7.56	8.30	10.06	12.16	13.42	13.93
Other genotypes	Organised	Marseille	40	9.86	6.99	7.38	8.06	9.77	11.80	13.02	13.58
Other genotypes	Organised	Marseille	41	9.60	6.80	7.16	7.83	9.52	11.51	12.67	13.24
Other genotypes	Organised	Marseille	42	9.40	6.62	6.96	7.66	9.30	11.28	12.43	13.05
Other genotypes	Organised	Marseille	43	9.23	6.48	6.81	7.52	9.14	11.07	12.25	12.78
Other genotypes	Organised	Marseille	44	9.10	6.39	6.75	7.41	9.00	10.94	12.05	12.57
Other genotypes	Organised	Marseille	45	8.99	6.31	6.65	7.31	8.89	10.83	11.90	12.50
Other genotypes	Organised	Marseille	46	8.90	6.24	6.57	7.25	8.81	10.75	11.85	12.36

Table S9 Posterior expected HR HPV cervical infection prevalence (in %) in major French cities, stratified by type of test, city, and age. Table counterpart to Figure 3A. *(continued)*

Virus	Test	City	Age	Posterior distribution							
					Percentiles						
				Average	0.01	0.025	0.1	0.5	0.9	0.975	0.99
Other genotypes	Organised	Marseille	47	8.85	6.20	6.53	7.21	8.77	10.68	11.80	12.28
Other genotypes	Organised	Marseille	48	8.82	6.20	6.50	7.18	8.73	10.65	11.75	12.26
Other genotypes	Organised	Marseille	49	8.80	6.18	6.46	7.17	8.72	10.62	11.67	12.19
Other genotypes	Organised	Marseille	50	8.78	6.16	6.45	7.14	8.71	10.60	11.71	12.19
Other genotypes	Organised	Marseille	51	8.75	6.12	6.43	7.09	8.67	10.57	11.69	12.16
Other genotypes	Organised	Marseille	52	8.68	6.07	6.40	7.06	8.62	10.51	11.64	12.13
Other genotypes	Organised	Marseille	53	8.61	6.06	6.36	6.98	8.53	10.43	11.54	12.02
Other genotypes	Organised	Marseille	54	8.48	5.99	6.28	6.88	8.40	10.28	11.34	11.87
Other genotypes	Organised	Marseille	55	8.37	5.93	6.17	6.78	8.29	10.15	11.19	11.75
Other genotypes	Organised	Marseille	56	8.28	5.86	6.11	6.70	8.19	10.01	11.10	11.57
Other genotypes	Organised	Marseille	57	8.16	5.81	6.03	6.62	8.07	9.87	10.95	11.41
Other genotypes	Organised	Marseille	58	8.07	5.73	5.95	6.55	7.98	9.75	10.81	11.30
Other genotypes	Organised	Marseille	59	8.01	5.67	5.88	6.49	7.92	9.65	10.73	11.22
Other genotypes	Organised	Marseille	60	7.98	5.65	5.90	6.47	7.88	9.60	10.71	11.19
Other genotypes	Organised	Marseille	61	7.99	5.62	5.91	6.47	7.88	9.61	10.73	11.22
Other genotypes	Organised	Marseille	62	8.04	5.61	5.92	6.51	7.94	9.70	10.80	11.33
Other genotypes	Organised	Marseille	63	8.15	5.66	5.98	6.58	8.04	9.84	10.94	11.56
Other genotypes	Organised	Marseille	64	8.30	5.70	6.08	6.68	8.18	10.04	11.24	11.84
Other genotypes	Organised	Marseille	65	8.50	5.79	6.15	6.81	8.40	10.36	11.58	12.21
Other genotypes	Organised	Marseille	66	8.74	5.84	6.25	6.92	8.63	10.71	12.01	12.67
Other genotypes	Organised	Toulouse	30	12.80	7.80	8.35	9.55	12.57	16.27	18.42	19.55
Other genotypes	Organised	Toulouse	31	12.19	7.51	7.98	9.06	11.96	15.47	17.59	18.68
Other genotypes	Organised	Toulouse	32	11.54	7.07	7.55	8.57	11.32	14.70	16.72	17.91
Other genotypes	Organised	Toulouse	33	10.89	6.70	7.11	8.08	10.69	13.90	15.68	16.98
Other genotypes	Organised	Toulouse	34	10.35	6.35	6.73	7.68	10.15	13.22	14.93	16.14
Other genotypes	Organised	Toulouse	35	9.91	6.10	6.39	7.37	9.72	12.65	14.33	15.51
Other genotypes	Organised	Toulouse	36	9.53	5.88	6.14	7.08	9.34	12.18	13.84	14.82
Other genotypes	Organised	Toulouse	37	9.23	5.67	5.97	6.84	9.05	11.80	13.34	14.34
Other genotypes	Organised	Toulouse	38	8.95	5.51	5.80	6.64	8.77	11.45	12.93	13.99

Table S9 Posterior expected HR HPV cervical infection prevalence (in %) in major French cities, stratified by type of test, city, and age. Table counterpart to Figure 3A. ***(continued)***

					Posterior distribution							
					Percentiles							
Virus	Test	City	Age	Average	0.01	0.025	0.1	0.5	0.9	0.975	0.99
Other genotypes	Organised	Toulouse	39	8.70	5.31	5.60	6.45	8.54	11.12	12.60	13.52
Other genotypes	Organised	Toulouse	40	8.44	5.14	5.40	6.26	8.28	10.76	12.24	13.16
Other genotypes	Organised	Toulouse	41	8.22	5.01	5.26	6.10	8.05	10.47	11.97	12.84
Other genotypes	Organised	Toulouse	42	8.04	4.85	5.16	5.97	7.88	10.25	11.71	12.68
Other genotypes	Organised	Toulouse	43	7.90	4.77	5.08	5.88	7.75	10.10	11.55	12.47
Other genotypes	Organised	Toulouse	44	7.78	4.67	5.01	5.82	7.64	9.94	11.41	12.31
Other genotypes	Organised	Toulouse	45	7.68	4.63	4.94	5.72	7.55	9.81	11.32	12.12
Other genotypes	Organised	Toulouse	46	7.61	4.59	4.89	5.66	7.48	9.74	11.28	12.06
Other genotypes	Organised	Toulouse	47	7.56	4.55	4.87	5.61	7.41	9.69	11.21	11.97
Other genotypes	Organised	Toulouse	48	7.54	4.54	4.85	5.59	7.39	9.67	11.17	11.89
Other genotypes	Organised	Toulouse	49	7.52	4.54	4.84	5.57	7.37	9.67	11.08	11.80
Other genotypes	Organised	Toulouse	50	7.50	4.53	4.83	5.55	7.38	9.66	11.06	11.80
Other genotypes	Organised	Toulouse	51	7.48	4.51	4.81	5.53	7.34	9.61	11.03	11.75
Other genotypes	Organised	Toulouse	52	7.42	4.46	4.75	5.49	7.28	9.53	10.95	11.69
Other genotypes	Organised	Toulouse	53	7.35	4.42	4.71	5.44	7.22	9.45	10.83	11.61
Other genotypes	Organised	Toulouse	54	7.25	4.36	4.65	5.36	7.09	9.34	10.69	11.46
Other genotypes	Organised	Toulouse	55	7.15	4.27	4.58	5.29	7.01	9.19	10.56	11.38
Other genotypes	Organised	Toulouse	56	7.07	4.22	4.53	5.22	6.93	9.07	10.49	11.24
Other genotypes	Organised	Toulouse	57	6.97	4.17	4.46	5.14	6.83	8.93	10.33	11.13
Other genotypes	Organised	Toulouse	58	6.90	4.10	4.42	5.09	6.75	8.84	10.18	10.97
Other genotypes	Organised	Toulouse	59	6.84	4.08	4.38	5.03	6.70	8.78	10.08	10.86
Other genotypes	Organised	Toulouse	60	6.82	4.06	4.38	5.02	6.67	8.73	10.04	10.83
Other genotypes	Organised	Toulouse	61	6.82	4.08	4.37	5.02	6.67	8.77	10.10	10.83
Other genotypes	Organised	Toulouse	62	6.87	4.08	4.39	5.04	6.71	8.88	10.22	10.93
Other genotypes	Organised	Toulouse	63	6.96	4.13	4.45	5.10	6.79	9.02	10.39	11.09
Other genotypes	Organised	Toulouse	64	7.10	4.16	4.50	5.18	6.91	9.20	10.55	11.31
Other genotypes	Organised	Toulouse	65	7.27	4.22	4.57	5.28	7.09	9.41	10.83	11.63
Other genotypes	Organised	Toulouse	66	7.47	4.29	4.63	5.43	7.32	9.74	11.19	12.01
Other genotypes	Organised	Bordeaux	30	13.60	9.57	10.13	11.16	13.45	16.18	17.85	18.72

Table S9 **Posterior expected HR HPV cervical infection prevalence (in %) in major French cities, stratified by type of test, city, and age. Table counterpart to Figure 3A.** ***(continued)***

				Posterior distribution							
					Percentiles						
Virus	Test	City	Age	Average	0.01	0.025	0.1	0.5	0.9	0.975	0.99
Other genotypes	Organised	Bordeaux	31	12.95	9.18	9.64	10.67	12.82	15.41	16.98	17.73
Other genotypes	Organised	Bordeaux	32	12.26	8.78	9.18	10.12	12.16	14.59	16.01	16.79
Other genotypes	Organised	Bordeaux	33	11.58	8.28	8.65	9.54	11.47	13.79	15.16	15.90
Other genotypes	Organised	Bordeaux	34	11.00	7.84	8.23	9.05	10.89	13.12	14.41	15.12
Other genotypes	Organised	Bordeaux	35	10.54	7.47	7.88	8.68	10.42	12.60	13.85	14.53
Other genotypes	Organised	Bordeaux	36	10.14	7.14	7.56	8.35	10.03	12.11	13.27	13.97
Other genotypes	Organised	Bordeaux	37	9.82	6.89	7.32	8.09	9.71	11.74	12.84	13.58
Other genotypes	Organised	Bordeaux	38	9.52	6.72	7.11	7.83	9.42	11.39	12.50	13.17
Other genotypes	Organised	Bordeaux	39	9.25	6.49	6.92	7.60	9.14	11.05	12.15	12.77
Other genotypes	Organised	Bordeaux	40	8.98	6.31	6.73	7.37	8.89	10.75	11.83	12.45
Other genotypes	Organised	Bordeaux	41	8.74	6.15	6.57	7.17	8.64	10.48	11.55	12.11
Other genotypes	Organised	Bordeaux	42	8.56	6.01	6.41	7.01	8.46	10.26	11.36	11.76
Other genotypes	Organised	Bordeaux	43	8.41	5.89	6.27	6.89	8.31	10.06	11.15	11.66
Other genotypes	Organised	Bordeaux	44	8.28	5.79	6.18	6.78	8.19	9.92	11.00	11.51
Other genotypes	Organised	Bordeaux	45	8.18	5.76	6.09	6.70	8.07	9.80	10.87	11.34
Other genotypes	Organised	Bordeaux	46	8.10	5.69	6.05	6.63	8.00	9.72	10.72	11.16
Other genotypes	Organised	Bordeaux	47	8.05	5.66	5.98	6.60	7.94	9.66	10.64	11.12
Other genotypes	Organised	Bordeaux	48	8.02	5.63	5.95	6.56	7.90	9.64	10.61	11.12
Other genotypes	Organised	Bordeaux	49	8.00	5.58	5.96	6.56	7.89	9.60	10.57	11.09
Other genotypes	Organised	Bordeaux	50	7.99	5.58	5.94	6.53	7.88	9.56	10.53	10.99
Other genotypes	Organised	Bordeaux	51	7.96	5.55	5.91	6.51	7.88	9.52	10.53	11.01
Other genotypes	Organised	Bordeaux	52	7.90	5.53	5.86	6.46	7.82	9.48	10.47	10.98
Other genotypes	Organised	Bordeaux	53	7.83	5.48	5.79	6.40	7.76	9.40	10.32	10.89
Other genotypes	Organised	Bordeaux	54	7.72	5.39	5.69	6.32	7.66	9.26	10.16	10.81
Other genotypes	Organised	Bordeaux	55	7.62	5.36	5.61	6.24	7.54	9.16	10.07	10.71
Other genotypes	Organised	Bordeaux	56	7.53	5.28	5.53	6.16	7.46	9.06	10.00	10.51
Other genotypes	Organised	Bordeaux	57	7.42	5.24	5.47	6.08	7.35	8.92	9.84	10.35
Other genotypes	Organised	Bordeaux	58	7.34	5.20	5.40	6.01	7.26	8.83	9.67	10.30
Other genotypes	Organised	Bordeaux	59	7.28	5.15	5.34	5.97	7.20	8.75	9.60	10.13

Table S9 Posterior expected HR HPV cervical infection prevalence (in %) in major French cities, stratified by type of test, city, and age. Table counterpart to Figure 3A. *(continued)*

				Posterior distribution							
					Percentiles						
Virus	Test	City	Age	Average	0.01	0.025	0.1	0.5	0.9	0.975	0.99
Other genotypes	Organised	Bordeaux	60	7.26	5.13	5.35	5.95	7.17	8.73	9.54	10.13
Other genotypes	Organised	Bordeaux	61	7.26	5.13	5.34	5.92	7.19	8.73	9.55	10.14
Other genotypes	Organised	Bordeaux	62	7.31	5.15	5.37	5.94	7.25	8.78	9.70	10.19
Other genotypes	Organised	Bordeaux	63	7.41	5.20	5.41	6.02	7.34	8.90	9.88	10.36
Other genotypes	Organised	Bordeaux	64	7.55	5.26	5.49	6.11	7.47	9.07	10.10	10.64
Other genotypes	Organised	Bordeaux	65	7.74	5.36	5.56	6.23	7.65	9.34	10.41	11.03
Other genotypes	Organised	Bordeaux	66	7.95	5.41	5.64	6.33	7.84	9.69	10.88	11.46
Other genotypes	Organised	Montpellier	30	14.60	9.46	10.05	11.35	14.41	18.13	20.60	21.91
Other genotypes	Organised	Montpellier	31	13.91	9.11	9.59	10.84	13.74	17.23	19.60	20.85
Other genotypes	Organised	Montpellier	32	13.19	8.62	9.06	10.25	13.04	16.32	18.60	19.74
Other genotypes	Organised	Montpellier	33	12.46	8.13	8.59	9.69	12.32	15.49	17.54	18.78
Other genotypes	Organised	Montpellier	34	11.85	7.71	8.15	9.20	11.71	14.70	16.73	18.04
Other genotypes	Organised	Montpellier	35	11.36	7.36	7.79	8.81	11.23	14.12	16.04	17.29
Other genotypes	Organised	Montpellier	36	10.93	7.10	7.48	8.45	10.79	13.57	15.47	16.57
Other genotypes	Organised	Montpellier	37	10.58	6.85	7.25	8.17	10.44	13.16	14.96	16.01
Other genotypes	Organised	Montpellier	38	10.27	6.59	7.04	7.92	10.14	12.75	14.51	15.56
Other genotypes	Organised	Montpellier	39	9.99	6.39	6.83	7.69	9.84	12.43	14.09	15.22
Other genotypes	Organised	Montpellier	40	9.69	6.16	6.62	7.47	9.56	12.06	13.72	14.79
Other genotypes	Organised	Montpellier	41	9.44	6.00	6.47	7.26	9.31	11.75	13.37	14.30
Other genotypes	Organised	Montpellier	42	9.24	5.86	6.32	7.10	9.12	11.50	13.08	14.01
Other genotypes	Organised	Montpellier	43	9.08	5.74	6.20	6.97	8.95	11.30	12.91	13.77
Other genotypes	Organised	Montpellier	44	8.94	5.65	6.11	6.85	8.83	11.15	12.69	13.65
Other genotypes	Organised	Montpellier	45	8.83	5.59	6.01	6.78	8.72	11.01	12.61	13.57
Other genotypes	Organised	Montpellier	46	8.75	5.57	5.94	6.72	8.64	10.90	12.51	13.38
Other genotypes	Organised	Montpellier	47	8.70	5.54	5.91	6.68	8.58	10.82	12.43	13.23
Other genotypes	Organised	Montpellier	48	8.67	5.47	5.90	6.65	8.55	10.79	12.39	13.34
Other genotypes	Organised	Montpellier	49	8.65	5.46	5.87	6.63	8.53	10.77	12.38	13.22
Other genotypes	Organised	Montpellier	50	8.63	5.43	5.86	6.60	8.51	10.73	12.39	13.18
Other genotypes	Organised	Montpellier	51	8.60	5.41	5.83	6.58	8.48	10.73	12.32	13.06

Table S9 **Posterior expected HR HPV cervical infection prevalence (in %) in major French cities, stratified by type of test, city, and age. Table counterpart to Figure 3A.** ***(continued)***

				Posterior distribution							
					Percentiles						
Virus	Test	City	Age	Average	0.01	0.025	0.1	0.5	0.9	0.975	0.99
Other genotypes	Organised	Montpellier	52	8.54	5.36	5.79	6.52	8.42	10.64	12.21	13.10
Other genotypes	Organised	Montpellier	53	8.46	5.37	5.77	6.45	8.35	10.55	12.06	12.87
Other genotypes	Organised	Montpellier	54	8.34	5.35	5.69	6.36	8.22	10.42	11.95	12.78
Other genotypes	Organised	Montpellier	55	8.23	5.25	5.58	6.27	8.11	10.26	11.79	12.59
Other genotypes	Organised	Montpellier	56	8.14	5.19	5.53	6.20	8.02	10.13	11.61	12.34
Other genotypes	Organised	Montpellier	57	8.03	5.11	5.43	6.14	7.92	10.01	11.49	12.20
Other genotypes	Organised	Montpellier	58	7.94	5.07	5.37	6.07	7.83	9.91	11.36	12.13
Other genotypes	Organised	Montpellier	59	7.88	5.03	5.33	6.02	7.77	9.83	11.25	12.04
Other genotypes	Organised	Montpellier	60	7.85	4.97	5.28	5.98	7.74	9.83	11.24	11.92
Other genotypes	Organised	Montpellier	61	7.86	4.97	5.28	5.99	7.74	9.84	11.19	11.88
Other genotypes	Organised	Montpellier	62	7.91	4.99	5.30	6.01	7.78	9.92	11.24	11.93
Other genotypes	Organised	Montpellier	63	8.01	5.05	5.35	6.10	7.88	10.06	11.37	12.18
Other genotypes	Organised	Montpellier	64	8.16	5.17	5.42	6.21	8.03	10.28	11.64	12.49
Other genotypes	Organised	Montpellier	65	8.36	5.23	5.52	6.32	8.22	10.56	12.02	12.99
Other genotypes	Organised	Montpellier	66	8.59	5.26	5.64	6.47	8.46	10.92	12.42	13.52
Other genotypes	Organised	Rennes	30	12.75	9.04	9.47	10.57	12.66	15.11	16.70	17.41
Other genotypes	Organised	Rennes	31	12.14	8.62	9.05	10.07	12.03	14.36	15.84	16.44
Other genotypes	Organised	Rennes	32	11.49	8.17	8.61	9.52	11.38	13.57	14.88	15.54
Other genotypes	Organised	Rennes	33	10.84	7.73	8.12	9.01	10.75	12.84	14.06	14.68
Other genotypes	Organised	Rennes	34	10.30	7.36	7.74	8.54	10.22	12.20	13.39	13.89
Other genotypes	Organised	Rennes	35	9.86	7.04	7.35	8.16	9.80	11.67	12.77	13.36
Other genotypes	Organised	Rennes	36	9.48	6.81	7.06	7.83	9.42	11.24	12.28	12.92
Other genotypes	Organised	Rennes	37	9.18	6.56	6.87	7.59	9.12	10.88	11.89	12.51
Other genotypes	Organised	Rennes	38	8.90	6.31	6.62	7.38	8.82	10.53	11.57	12.16
Other genotypes	Organised	Rennes	39	8.65	6.16	6.46	7.16	8.58	10.27	11.26	11.74
Other genotypes	Organised	Rennes	40	8.39	6.00	6.29	6.93	8.33	9.96	10.93	11.37
Other genotypes	Organised	Rennes	41	8.17	5.84	6.11	6.73	8.12	9.69	10.67	11.10
Other genotypes	Organised	Rennes	42	7.99	5.71	5.99	6.58	7.94	9.48	10.44	10.88
Other genotypes	Organised	Rennes	43	7.85	5.60	5.88	6.47	7.80	9.32	10.24	10.68

Table S9 Posterior expected HR HPV cervical infection prevalence (in %) in major French cities, stratified by type of test, city, and age. Table counterpart to Figure 3A. ***(continued)***

				Posterior distribution							
					Percentiles						
Virus	Test	City	Age	Average	0.01	0.025	0.1	0.5	0.9	0.975	0.99
Other genotypes	Organised	Rennes	44	7.73	5.51	5.78	6.36	7.68	9.20	10.07	10.49
Other genotypes	Organised	Rennes	45	7.64	5.44	5.70	6.29	7.58	9.08	9.97	10.31
Other genotypes	Organised	Rennes	46	7.57	5.40	5.66	6.22	7.51	9.01	9.87	10.27
Other genotypes	Organised	Rennes	47	7.52	5.37	5.62	6.18	7.45	8.93	9.80	10.20
Other genotypes	Organised	Rennes	48	7.49	5.36	5.60	6.17	7.43	8.90	9.76	10.18
Other genotypes	Organised	Rennes	49	7.47	5.34	5.61	6.14	7.41	8.88	9.74	10.17
Other genotypes	Organised	Rennes	50	7.46	5.33	5.60	6.12	7.41	8.85	9.70	10.14
Other genotypes	Organised	Rennes	51	7.43	5.27	5.60	6.11	7.38	8.83	9.67	10.08
Other genotypes	Organised	Rennes	52	7.38	5.25	5.55	6.04	7.34	8.78	9.60	9.99
Other genotypes	Organised	Rennes	53	7.31	5.19	5.49	6.00	7.26	8.70	9.53	9.93
Other genotypes	Organised	Rennes	54	7.20	5.14	5.40	5.90	7.14	8.57	9.40	9.81
Other genotypes	Organised	Rennes	55	7.11	5.07	5.34	5.82	7.06	8.47	9.27	9.68
Other genotypes	Organised	Rennes	56	7.03	5.02	5.27	5.75	6.96	8.36	9.19	9.57
Other genotypes	Organised	Rennes	57	6.93	4.95	5.19	5.67	6.86	8.26	9.08	9.43
Other genotypes	Organised	Rennes	58	6.85	4.89	5.13	5.62	6.79	8.17	8.97	9.31
Other genotypes	Organised	Rennes	59	6.80	4.83	5.10	5.57	6.74	8.10	8.90	9.23
Other genotypes	Organised	Rennes	60	6.78	4.82	5.06	5.55	6.71	8.07	8.85	9.25
Other genotypes	Organised	Rennes	61	6.78	4.82	5.07	5.56	6.72	8.08	8.84	9.29
Other genotypes	Organised	Rennes	62	6.83	4.82	5.07	5.60	6.77	8.13	8.91	9.36
Other genotypes	Organised	Rennes	63	6.92	4.86	5.11	5.65	6.85	8.26	9.07	9.56
Other genotypes	Organised	Rennes	64	7.05	4.94	5.17	5.75	6.97	8.44	9.28	9.83
Other genotypes	Organised	Rennes	65	7.22	4.96	5.25	5.85	7.14	8.71	9.57	10.08
Other genotypes	Organised	Rennes	66	7.43	5.02	5.32	5.93	7.35	9.00	9.99	10.57
Other genotypes	Organised	Nantes	30	11.81	7.26	7.76	8.85	11.59	15.07	17.09	18.07
Other genotypes	Organised	Nantes	31	11.23	6.92	7.41	8.45	11.04	14.36	16.26	17.11
Other genotypes	Organised	Nantes	32	10.63	6.61	7.01	7.98	10.43	13.59	15.47	16.15
Other genotypes	Organised	Nantes	33	10.03	6.21	6.61	7.53	9.82	12.82	14.64	15.21
Other genotypes	Organised	Nantes	34	9.52	5.84	6.26	7.14	9.32	12.18	13.90	14.45
Other genotypes	Organised	Nantes	35	9.12	5.58	6.03	6.85	8.92	11.67	13.26	13.89

Table S9 **Posterior expected HR HPV cervical infection prevalence (in %) in major French cities, stratified by type of test, city, and age. Table counterpart to Figure 3A.** ***(continued)***

				Posterior distribution							
					Percentiles						
Virus	Test	City	Age	Average	0.01	0.025	0.1	0.5	0.9	0.975	0.99
Other genotypes	Organised	Nantes	36	8.76	5.31	5.79	6.56	8.59	11.26	12.78	13.40
Other genotypes	Organised	Nantes	37	8.48	5.12	5.61	6.34	8.32	10.91	12.38	13.05
Other genotypes	Organised	Nantes	38	8.23	4.95	5.44	6.15	8.08	10.58	12.01	12.71
Other genotypes	Organised	Nantes	39	7.99	4.81	5.27	5.96	7.84	10.27	11.65	12.31
Other genotypes	Organised	Nantes	40	7.75	4.67	5.09	5.77	7.61	9.99	11.30	11.90
Other genotypes	Organised	Nantes	41	7.54	4.53	4.94	5.61	7.40	9.74	10.97	11.74
Other genotypes	Organised	Nantes	42	7.38	4.41	4.83	5.48	7.24	9.53	10.75	11.45
Other genotypes	Organised	Nantes	43	7.25	4.34	4.72	5.39	7.11	9.36	10.57	11.25
Other genotypes	Organised	Nantes	44	7.14	4.28	4.64	5.29	6.99	9.24	10.41	11.15
Other genotypes	Organised	Nantes	45	7.05	4.28	4.58	5.22	6.90	9.09	10.33	11.03
Other genotypes	Organised	Nantes	46	6.99	4.22	4.53	5.18	6.83	9.03	10.29	10.89
Other genotypes	Organised	Nantes	47	6.94	4.16	4.49	5.13	6.79	8.94	10.19	10.83
Other genotypes	Organised	Nantes	48	6.92	4.15	4.49	5.12	6.79	8.91	10.13	10.84
Other genotypes	Organised	Nantes	49	6.90	4.11	4.47	5.13	6.76	8.91	10.08	10.74
Other genotypes	Organised	Nantes	50	6.88	4.08	4.47	5.11	6.73	8.90	10.07	10.70
Other genotypes	Organised	Nantes	51	6.86	4.08	4.44	5.09	6.71	8.88	10.03	10.74
Other genotypes	Organised	Nantes	52	6.81	4.06	4.43	5.06	6.66	8.78	9.99	10.68
Other genotypes	Organised	Nantes	53	6.75	4.03	4.39	5.01	6.60	8.71	9.92	10.58
Other genotypes	Organised	Nantes	54	6.65	3.98	4.33	4.95	6.50	8.61	9.79	10.37
Other genotypes	Organised	Nantes	55	6.56	3.90	4.26	4.86	6.42	8.48	9.62	10.15
Other genotypes	Organised	Nantes	56	6.48	3.84	4.21	4.80	6.33	8.39	9.53	10.05
Other genotypes	Organised	Nantes	57	6.40	3.80	4.13	4.73	6.24	8.29	9.42	9.99
Other genotypes	Organised	Nantes	58	6.32	3.79	4.08	4.69	6.17	8.19	9.31	9.86
Other genotypes	Organised	Nantes	59	6.27	3.73	4.06	4.66	6.12	8.11	9.21	9.73
Other genotypes	Organised	Nantes	60	6.25	3.71	4.02	4.66	6.10	8.06	9.18	9.76
Other genotypes	Organised	Nantes	61	6.26	3.73	4.04	4.66	6.10	8.07	9.21	9.79
Other genotypes	Organised	Nantes	62	6.30	3.74	4.06	4.70	6.15	8.13	9.28	9.88
Other genotypes	Organised	Nantes	63	6.38	3.80	4.08	4.76	6.23	8.27	9.43	10.03
Other genotypes	Organised	Nantes	64	6.51	3.88	4.15	4.83	6.35	8.45	9.64	10.22

Table S9 Posterior expected HR HPV cervical infection prevalence (in %) in major French cities, stratified by type of test, city, and age. Table counterpart to Figure 3A. *(continued)*

				Posterior distribution							
					Percentiles						
Virus	Test	City	Age	Average	0.01	0.025	0.1	0.5	0.9	0.975	0.99
Other genotypes	Organised	Nantes	65	6.67	3.95	4.23	4.92	6.51	8.66	10.00	10.61
Other genotypes	Organised	Nantes	66	6.86	4.02	4.31	5.04	6.68	8.93	10.39	11.03
Other genotypes	Organised	Lille	30	12.70	8.12	8.74	9.88	12.51	15.74	17.93	18.81
Other genotypes	Organised	Lille	31	12.09	7.76	8.29	9.44	11.90	14.97	16.96	17.92
Other genotypes	Organised	Lille	32	11.45	7.36	7.89	8.94	11.25	14.17	16.02	17.10
Other genotypes	Organised	Lille	33	10.80	6.90	7.43	8.43	10.62	13.42	15.19	16.18
Other genotypes	Organised	Lille	34	10.26	6.49	7.07	8.02	10.09	12.76	14.45	15.42
Other genotypes	Organised	Lille	35	9.83	6.21	6.78	7.69	9.65	12.22	13.85	14.82
Other genotypes	Organised	Lille	36	9.45	5.98	6.52	7.37	9.27	11.77	13.30	14.25
Other genotypes	Organised	Lille	37	9.15	5.76	6.28	7.14	8.98	11.37	12.89	13.85
Other genotypes	Organised	Lille	38	8.87	5.61	6.11	6.92	8.71	11.03	12.51	13.48
Other genotypes	Organised	Lille	39	8.62	5.44	5.93	6.73	8.46	10.72	12.15	13.02
Other genotypes	Organised	Lille	40	8.37	5.29	5.73	6.53	8.21	10.43	11.79	12.63
Other genotypes	Organised	Lille	41	8.14	5.15	5.54	6.35	8.00	10.16	11.43	12.28
Other genotypes	Organised	Lille	42	7.97	5.04	5.43	6.22	7.82	9.99	11.17	11.98
Other genotypes	Organised	Lille	43	7.83	4.91	5.30	6.11	7.69	9.82	11.00	11.84
Other genotypes	Organised	Lille	44	7.71	4.83	5.21	6.01	7.58	9.66	10.84	11.69
Other genotypes	Organised	Lille	45	7.61	4.78	5.14	5.93	7.46	9.53	10.70	11.52
Other genotypes	Organised	Lille	46	7.54	4.74	5.08	5.88	7.40	9.44	10.61	11.41
Other genotypes	Organised	Lille	47	7.49	4.73	5.07	5.83	7.34	9.36	10.55	11.32
Other genotypes	Organised	Lille	48	7.47	4.71	5.09	5.79	7.33	9.32	10.53	11.27
Other genotypes	Organised	Lille	49	7.45	4.69	5.06	5.80	7.31	9.30	10.49	11.20
Other genotypes	Organised	Lille	50	7.43	4.70	5.01	5.79	7.29	9.29	10.51	11.19
Other genotypes	Organised	Lille	51	7.41	4.63	5.00	5.77	7.28	9.24	10.43	11.13
Other genotypes	Organised	Lille	52	7.35	4.59	4.96	5.73	7.22	9.20	10.37	11.09
Other genotypes	Organised	Lille	53	7.29	4.53	4.90	5.66	7.15	9.10	10.24	10.96
Other genotypes	Organised	Lille	54	7.18	4.44	4.82	5.57	7.04	9.00	10.12	10.84
Other genotypes	Organised	Lille	55	7.09	4.41	4.78	5.49	6.95	8.87	9.98	10.68
Other genotypes	Organised	Lille	56	7.00	4.36	4.74	5.42	6.87	8.78	9.89	10.54

Table S9 **Posterior expected HR HPV cervical infection prevalence (in %) in major French cities, stratified by type of test, city, and age. Table counterpart to Figure 3A.** ***(continued)***

					Posterior distribution							
						Percentiles						
Virus	Test	City	Age	Average	0.01	0.025	0.1	0.5	0.9	0.975	0.99
Other genotypes	Organised	Lille	57	6.91	4.32	4.67	5.33	6.78	8.67	9.73	10.44
Other genotypes	Organised	Lille	58	6.83	4.25	4.61	5.28	6.70	8.57	9.61	10.22
Other genotypes	Organised	Lille	59	6.78	4.23	4.60	5.23	6.65	8.49	9.59	10.19
Other genotypes	Organised	Lille	60	6.75	4.22	4.57	5.22	6.62	8.46	9.57	10.15
Other genotypes	Organised	Lille	61	6.76	4.24	4.59	5.23	6.63	8.48	9.59	10.20
Other genotypes	Organised	Lille	62	6.80	4.26	4.59	5.25	6.66	8.52	9.63	10.32
Other genotypes	Organised	Lille	63	6.90	4.30	4.66	5.29	6.77	8.66	9.78	10.42
Other genotypes	Organised	Lille	64	7.03	4.37	4.75	5.40	6.92	8.86	9.98	10.69
Other genotypes	Organised	Lille	65	7.20	4.45	4.82	5.52	7.08	9.12	10.27	10.99
Other genotypes	Organised	Lille	66	7.40	4.56	4.88	5.60	7.25	9.45	10.68	11.44
Other genotypes	Organised	Strasbourg	30	11.01	6.37	6.89	8.09	10.76	14.28	16.54	17.85
Other genotypes	Organised	Strasbourg	31	10.48	6.04	6.56	7.68	10.23	13.54	15.75	17.04
Other genotypes	Organised	Strasbourg	32	9.91	5.74	6.17	7.27	9.67	12.85	14.93	16.15
Other genotypes	Organised	Strasbourg	33	9.34	5.42	5.80	6.86	9.12	12.12	14.12	15.26
Other genotypes	Organised	Strasbourg	34	8.87	5.04	5.48	6.49	8.64	11.50	13.42	14.54
Other genotypes	Organised	Strasbourg	35	8.49	4.80	5.26	6.20	8.27	11.00	12.86	13.98
Other genotypes	Organised	Strasbourg	36	8.16	4.61	5.01	5.96	7.94	10.59	12.42	13.34
Other genotypes	Organised	Strasbourg	37	7.89	4.47	4.88	5.75	7.70	10.24	12.04	12.88
Other genotypes	Organised	Strasbourg	38	7.66	4.36	4.74	5.56	7.46	9.96	11.70	12.61
Other genotypes	Organised	Strasbourg	39	7.43	4.21	4.60	5.39	7.22	9.69	11.38	12.24
Other genotypes	Organised	Strasbourg	40	7.21	4.09	4.45	5.22	7.01	9.40	11.02	11.88
Other genotypes	Organised	Strasbourg	41	7.02	4.01	4.34	5.08	6.81	9.14	10.70	11.59
Other genotypes	Organised	Strasbourg	42	6.86	3.92	4.23	4.97	6.67	8.96	10.53	11.38
Other genotypes	Organised	Strasbourg	43	6.74	3.87	4.16	4.88	6.55	8.81	10.33	11.28
Other genotypes	Organised	Strasbourg	44	6.64	3.80	4.10	4.80	6.45	8.69	10.13	11.06
Other genotypes	Organised	Strasbourg	45	6.56	3.75	4.05	4.74	6.38	8.60	10.03	10.89
Other genotypes	Organised	Strasbourg	46	6.50	3.71	4.00	4.72	6.31	8.53	9.94	10.76
Other genotypes	Organised	Strasbourg	47	6.45	3.69	3.97	4.69	6.28	8.45	9.88	10.79
Other genotypes	Organised	Strasbourg	48	6.43	3.69	3.97	4.66	6.25	8.43	9.87	10.79

Table S9 **Posterior expected HR HPV cervical infection prevalence (in %) in major French cities, stratified by type of test, city, and age. Table counterpart to Figure 3A.** ***(continued)***

				Posterior distribution							
					Percentiles						
Virus	Test	City	Age	Average	0.01	0.025	0.1	0.5	0.9	0.975	0.99
Other genotypes	Organised	Strasbourg	49	6.41	3.65	3.97	4.66	6.23	8.39	9.82	10.73
Other genotypes	Organised	Strasbourg	50	6.40	3.62	3.96	4.66	6.21	8.38	9.83	10.73
Other genotypes	Organised	Strasbourg	51	6.38	3.63	3.93	4.64	6.19	8.34	9.78	10.68
Other genotypes	Organised	Strasbourg	52	6.33	3.62	3.89	4.60	6.15	8.31	9.70	10.61
Other genotypes	Organised	Strasbourg	53	6.27	3.55	3.87	4.56	6.09	8.23	9.57	10.50
Other genotypes	Organised	Strasbourg	54	6.18	3.50	3.80	4.48	6.00	8.10	9.40	10.29
Other genotypes	Organised	Strasbourg	55	6.10	3.43	3.73	4.43	5.92	8.03	9.28	10.18
Other genotypes	Organised	Strasbourg	56	6.02	3.42	3.68	4.38	5.85	7.93	9.12	10.07
Other genotypes	Organised	Strasbourg	57	5.94	3.33	3.63	4.32	5.78	7.81	9.06	9.96
Other genotypes	Organised	Strasbourg	58	5.87	3.30	3.59	4.28	5.72	7.70	8.95	9.77
Other genotypes	Organised	Strasbourg	59	5.83	3.27	3.56	4.24	5.67	7.59	8.87	9.74
Other genotypes	Organised	Strasbourg	60	5.81	3.29	3.55	4.23	5.65	7.60	8.91	9.62
Other genotypes	Organised	Strasbourg	61	5.81	3.28	3.55	4.22	5.66	7.60	8.90	9.67
Other genotypes	Organised	Strasbourg	62	5.85	3.29	3.55	4.24	5.70	7.68	8.96	9.75
Other genotypes	Organised	Strasbourg	63	5.93	3.30	3.60	4.30	5.77	7.78	9.01	9.88
Other genotypes	Organised	Strasbourg	64	6.05	3.35	3.67	4.35	5.87	7.94	9.17	10.03
Other genotypes	Organised	Strasbourg	65	6.20	3.41	3.75	4.45	6.02	8.14	9.41	10.26
Other genotypes	Organised	Strasbourg	66	6.37	3.49	3.79	4.53	6.18	8.39	9.80	10.70
Other genotypes	Organised	Lyon	30	14.72	9.29	10.02	11.30	14.50	18.33	20.78	21.98
Other genotypes	Organised	Lyon	31	14.03	8.86	9.58	10.77	13.82	17.44	19.80	20.91
Other genotypes	Organised	Lyon	32	13.30	8.46	9.05	10.17	13.11	16.53	18.89	19.81
Other genotypes	Organised	Lyon	33	12.57	7.97	8.53	9.62	12.39	15.66	17.91	18.75
Other genotypes	Organised	Lyon	34	11.95	7.60	8.08	9.12	11.78	14.92	17.06	17.98
Other genotypes	Organised	Lyon	35	11.45	7.27	7.71	8.74	11.28	14.28	16.40	17.18
Other genotypes	Organised	Lyon	36	11.02	6.96	7.43	8.41	10.85	13.74	15.80	16.58
Other genotypes	Organised	Lyon	37	10.68	6.72	7.22	8.15	10.53	13.28	15.29	16.18
Other genotypes	Organised	Lyon	38	10.36	6.50	6.97	7.92	10.21	12.88	14.93	15.72
Other genotypes	Organised	Lyon	39	10.07	6.33	6.79	7.68	9.93	12.52	14.50	15.26
Other genotypes	Organised	Lyon	40	9.78	6.14	6.56	7.44	9.63	12.19	14.13	14.75

Table S9 **Posterior expected HR HPV cervical infection prevalence (in %) in major French cities, stratified by type of test, city, and age. Table counterpart to Figure 3A.** *(continued)*

				Posterior distribution							
					Percentiles						
Virus	Test	City	Age	Average	0.01	0.025	0.1	0.5	0.9	0.975	0.99
Other genotypes	Organised	Lyon	41	9.52	5.93	6.37	7.23	9.39	11.89	13.73	14.38
Other genotypes	Organised	Lyon	42	9.32	5.80	6.24	7.08	9.19	11.63	13.52	14.10
Other genotypes	Organised	Lyon	43	9.16	5.69	6.11	6.97	9.03	11.45	13.26	13.93
Other genotypes	Organised	Lyon	44	9.02	5.61	6.00	6.86	8.90	11.27	13.06	13.81
Other genotypes	Organised	Lyon	45	8.91	5.53	5.91	6.76	8.76	11.17	12.88	13.73
Other genotypes	Organised	Lyon	46	8.83	5.44	5.85	6.68	8.69	11.06	12.73	13.56
Other genotypes	Organised	Lyon	47	8.77	5.45	5.81	6.65	8.65	10.99	12.70	13.47
Other genotypes	Organised	Lyon	48	8.74	5.44	5.82	6.62	8.63	10.94	12.66	13.41
Other genotypes	Organised	Lyon	49	8.72	5.41	5.81	6.62	8.60	10.92	12.61	13.39
Other genotypes	Organised	Lyon	50	8.70	5.40	5.81	6.61	8.58	10.90	12.60	13.36
Other genotypes	Organised	Lyon	51	8.68	5.37	5.80	6.58	8.56	10.90	12.57	13.32
Other genotypes	Organised	Lyon	52	8.61	5.34	5.74	6.53	8.50	10.82	12.45	13.27
Other genotypes	Organised	Lyon	53	8.54	5.31	5.66	6.46	8.42	10.69	12.33	13.19
Other genotypes	Organised	Lyon	54	8.42	5.23	5.57	6.37	8.31	10.58	12.24	13.00
Other genotypes	Organised	Lyon	55	8.31	5.14	5.52	6.29	8.19	10.43	12.04	13.00
Other genotypes	Organised	Lyon	56	8.21	5.07	5.44	6.21	8.10	10.31	11.97	12.93
Other genotypes	Organised	Lyon	57	8.10	5.00	5.37	6.12	8.00	10.18	11.80	12.69
Other genotypes	Organised	Lyon	58	8.01	4.92	5.32	6.05	7.90	10.08	11.67	12.53
Other genotypes	Organised	Lyon	59	7.95	4.88	5.28	6.00	7.83	10.03	11.58	12.45
Other genotypes	Organised	Lyon	60	7.92	4.89	5.26	5.99	7.81	10.01	11.55	12.41
Other genotypes	Organised	Lyon	61	7.93	4.88	5.24	5.97	7.79	10.00	11.59	12.46
Other genotypes	Organised	Lyon	62	7.98	4.90	5.26	6.00	7.86	10.09	11.66	12.58
Other genotypes	Organised	Lyon	63	8.09	4.99	5.31	6.06	7.95	10.23	11.76	12.76
Other genotypes	Organised	Lyon	64	8.24	5.01	5.40	6.16	8.10	10.46	12.01	12.98
Other genotypes	Organised	Lyon	65	8.44	5.06	5.51	6.32	8.29	10.76	12.36	13.29
Other genotypes	Organised	Lyon	66	8.67	5.13	5.63	6.46	8.53	11.14	12.76	13.73
Other genotypes	Organised	Paris	30	16.37	11.88	12.57	13.76	16.26	19.16	20.68	21.56
Other genotypes	Organised	Paris	31	15.61	11.43	12.02	13.16	15.52	18.28	19.71	20.59
Other genotypes	Organised	Paris	32	14.81	10.82	11.39	12.49	14.72	17.32	18.67	19.58

Table S9 Posterior expected HR HPV cervical infection prevalence (in %) in major French cities, stratified by type of test, city, and age. Table counterpart to Figure 3A. *(continued)*

				Posterior distribution							
					Percentiles						
Virus	Test	City	Age	Average	0.01	0.025	0.1	0.5	0.9	0.975	0.99
Other genotypes	Organised	Paris	33	14.01	10.19	10.76	11.78	13.91	16.35	17.69	18.39
Other genotypes	Organised	Paris	34	13.33	9.74	10.26	11.23	13.24	15.58	16.86	17.45
Other genotypes	Organised	Paris	35	12.79	9.30	9.87	10.75	12.71	14.94	16.24	16.73
Other genotypes	Organised	Paris	36	12.31	8.98	9.48	10.34	12.24	14.38	15.67	16.16
Other genotypes	Organised	Paris	37	11.93	8.73	9.18	10.01	11.87	13.91	15.16	15.69
Other genotypes	Organised	Paris	38	11.58	8.49	8.92	9.71	11.52	13.54	14.73	15.15
Other genotypes	Organised	Paris	39	11.26	8.27	8.68	9.43	11.20	13.16	14.40	14.81
Other genotypes	Organised	Paris	40	10.94	7.96	8.38	9.16	10.87	12.78	14.02	14.46
Other genotypes	Organised	Paris	41	10.65	7.78	8.14	8.89	10.58	12.48	13.61	14.16
Other genotypes	Organised	Paris	42	10.43	7.61	7.94	8.72	10.36	12.24	13.35	13.88
Other genotypes	Organised	Paris	43	10.25	7.49	7.82	8.57	10.19	12.06	13.13	13.69
Other genotypes	Organised	Paris	44	10.10	7.40	7.68	8.44	10.03	11.89	12.99	13.52
Other genotypes	Organised	Paris	45	9.98	7.30	7.56	8.30	9.91	11.73	12.86	13.39
Other genotypes	Organised	Paris	46	9.89	7.23	7.52	8.22	9.83	11.60	12.74	13.28
Other genotypes	Organised	Paris	47	9.82	7.18	7.47	8.18	9.77	11.52	12.61	13.21
Other genotypes	Organised	Paris	48	9.79	7.14	7.44	8.14	9.74	11.48	12.58	13.15
Other genotypes	Organised	Paris	49	9.77	7.12	7.43	8.14	9.71	11.45	12.57	13.21
Other genotypes	Organised	Paris	50	9.75	7.13	7.40	8.12	9.68	11.45	12.52	13.06
Other genotypes	Organised	Paris	51	9.72	7.08	7.41	8.09	9.65	11.40	12.48	13.00
Other genotypes	Organised	Paris	52	9.65	7.03	7.36	8.01	9.58	11.33	12.35	12.97
Other genotypes	Organised	Paris	53	9.56	6.98	7.27	7.93	9.49	11.21	12.26	12.86
Other genotypes	Organised	Paris	54	9.43	6.86	7.15	7.82	9.36	11.05	12.09	12.61
Other genotypes	Organised	Paris	55	9.30	6.74	7.04	7.73	9.23	10.92	11.89	12.42
Other genotypes	Organised	Paris	56	9.20	6.67	6.98	7.64	9.13	10.76	11.79	12.23
Other genotypes	Organised	Paris	57	9.07	6.58	6.88	7.55	9.01	10.63	11.64	12.06
Other genotypes	Organised	Paris	58	8.97	6.50	6.85	7.47	8.91	10.54	11.53	12.00
Other genotypes	Organised	Paris	59	8.90	6.47	6.77	7.41	8.85	10.44	11.43	11.98
Other genotypes	Organised	Paris	60	8.87	6.46	6.75	7.37	8.83	10.39	11.36	11.94
Other genotypes	Organised	Paris	61	8.88	6.45	6.76	7.37	8.85	10.42	11.35	11.91

Table S9 **Posterior expected HR HPV cervical infection prevalence (in %) in major French cities, stratified by type of test, city, and age. Table counterpart to Figure 3A.** ***(continued)***

					Posterior distribution							
					Percentiles							
Virus	Test	City	Age	Average	0.01	0.025	0.1	0.5	0.9	0.975	0.99
Other genotypes	Organised	Paris	62	8.94	6.44	6.78	7.44	8.90	10.50	11.48	12.11
Other genotypes	Organised	Paris	63	9.06	6.49	6.85	7.52	9.01	10.64	11.65	12.25
Other genotypes	Organised	Paris	64	9.23	6.57	6.93	7.63	9.17	10.85	11.93	12.50
Other genotypes	Organised	Paris	65	9.45	6.65	7.03	7.76	9.37	11.15	12.33	12.88
Other genotypes	Organised	Paris	66	9.71	6.70	7.14	7.88	9.62	11.55	12.84	13.60
Other genotypes	Opportunistic	Nice	30	19.10	10.41	11.48	13.74	18.77	24.92	28.53	30.86
Other genotypes	Opportunistic	Nice	31	18.21	9.87	10.90	13.04	17.91	23.87	27.22	29.58
Other genotypes	Opportunistic	Nice	32	17.26	9.33	10.32	12.29	16.95	22.75	25.98	28.08
Other genotypes	Opportunistic	Nice	33	16.29	8.77	9.66	11.56	16.01	21.49	24.76	26.67
Other genotypes	Opportunistic	Nice	34	15.45	8.28	9.07	10.92	15.16	20.45	23.57	25.63
Other genotypes	Opportunistic	Nice	35	14.77	7.88	8.65	10.41	14.47	19.58	22.59	24.68
Other genotypes	Opportunistic	Nice	36	14.15	7.48	8.24	9.98	13.88	18.73	21.76	23.82
Other genotypes	Opportunistic	Nice	37	13.65	7.25	7.94	9.59	13.34	18.15	21.05	23.02
Other genotypes	Opportunistic	Nice	38	13.18	7.01	7.59	9.24	12.88	17.57	20.38	22.24
Other genotypes	Opportunistic	Nice	39	12.75	6.71	7.37	8.92	12.45	17.00	19.81	21.53
Other genotypes	Opportunistic	Nice	40	12.30	6.46	7.11	8.60	12.00	16.45	19.15	20.97
Other genotypes	Opportunistic	Nice	41	11.91	6.18	6.88	8.30	11.61	15.91	18.53	20.14
Other genotypes	Opportunistic	Nice	42	11.59	5.98	6.66	8.08	11.32	15.49	18.06	19.83
Other genotypes	Opportunistic	Nice	43	11.31	5.83	6.51	7.88	11.05	15.13	17.61	19.58
Other genotypes	Opportunistic	Nice	44	11.08	5.71	6.36	7.72	10.80	14.86	17.31	19.01
Other genotypes	Opportunistic	Nice	45	10.87	5.58	6.25	7.54	10.61	14.62	17.00	18.59
Other genotypes	Opportunistic	Nice	46	10.70	5.47	6.13	7.45	10.46	14.39	16.76	18.35
Other genotypes	Opportunistic	Nice	47	10.56	5.42	6.05	7.34	10.31	14.26	16.54	18.18
Other genotypes	Opportunistic	Nice	48	10.45	5.37	5.95	7.26	10.19	14.11	16.35	17.99
Other genotypes	Opportunistic	Nice	49	10.35	5.29	5.89	7.19	10.08	14.00	16.15	18.02
Other genotypes	Opportunistic	Nice	50	10.25	5.29	5.85	7.09	9.99	13.89	16.05	17.76
Other genotypes	Opportunistic	Nice	51	10.14	5.26	5.76	7.01	9.88	13.72	15.83	17.63
Other genotypes	Opportunistic	Nice	52	9.98	5.15	5.71	6.89	9.73	13.47	15.66	17.42
Other genotypes	Opportunistic	Nice	53	9.79	5.05	5.55	6.75	9.55	13.22	15.47	17.16

Table S9 **Posterior expected HR HPV cervical infection prevalence (in %) in major French cities, stratified by type of test, city, and age. Table counterpart to Figure 3A.** ***(continued)***

				Posterior distribution							
					Percentiles						
Virus	Test	City	Age	Average	0.01	0.025	0.1	0.5	0.9	0.975	0.99
Other genotypes	Opportunistic	Nice	54	9.56	4.91	5.42	6.58	9.30	12.95	15.07	16.78
Other genotypes	Opportunistic	Nice	55	9.33	4.82	5.28	6.42	9.08	12.61	14.72	16.41
Other genotypes	Opportunistic	Nice	56	9.12	4.67	5.19	6.31	8.88	12.31	14.37	16.09
Other genotypes	Opportunistic	Nice	57	8.89	4.52	5.05	6.14	8.66	11.97	14.03	15.74
Other genotypes	Opportunistic	Nice	58	8.70	4.40	4.93	6.01	8.47	11.71	13.76	15.46
Other genotypes	Opportunistic	Nice	59	8.54	4.33	4.81	5.88	8.30	11.56	13.51	15.10
Other genotypes	Opportunistic	Nice	60	8.44	4.27	4.74	5.82	8.20	11.40	13.34	14.90
Other genotypes	Opportunistic	Nice	61	8.37	4.27	4.71	5.77	8.16	11.31	13.24	14.79
Other genotypes	Opportunistic	Nice	62	8.36	4.30	4.70	5.75	8.16	11.30	13.25	14.76
Other genotypes	Opportunistic	Nice	63	8.41	4.33	4.79	5.76	8.21	11.37	13.32	14.85
Other genotypes	Opportunistic	Nice	64	8.51	4.35	4.86	5.85	8.29	11.49	13.41	15.00
Other genotypes	Opportunistic	Nice	65	8.66	4.37	4.93	5.94	8.44	11.72	13.64	15.16
Other genotypes	Opportunistic	Nice	66	8.84	4.43	5.08	6.06	8.57	11.99	13.96	15.54
Other genotypes	Opportunistic	Marseille	30	20.76	16.67	17.20	18.39	20.75	23.18	24.49	25.42
Other genotypes	Opportunistic	Marseille	31	19.81	15.84	16.40	17.56	19.79	22.11	23.37	24.25
Other genotypes	Opportunistic	Marseille	32	18.78	15.06	15.53	16.67	18.74	20.99	22.22	23.06
Other genotypes	Opportunistic	Marseille	33	17.74	14.09	14.70	15.72	17.69	19.84	21.05	21.84
Other genotypes	Opportunistic	Marseille	34	16.83	13.33	13.94	14.89	16.79	18.86	20.05	20.70
Other genotypes	Opportunistic	Marseille	35	16.09	12.75	13.27	14.21	16.04	18.06	19.23	19.78
Other genotypes	Opportunistic	Marseille	36	15.42	12.21	12.64	13.58	15.40	17.30	18.40	19.05
Other genotypes	Opportunistic	Marseille	37	14.88	11.78	12.14	13.09	14.85	16.73	17.74	18.41
Other genotypes	Opportunistic	Marseille	38	14.37	11.39	11.72	12.63	14.36	16.15	17.17	17.83
Other genotypes	Opportunistic	Marseille	39	13.90	11.02	11.34	12.24	13.89	15.61	16.63	17.23
Other genotypes	Opportunistic	Marseille	40	13.42	10.55	10.93	11.78	13.40	15.11	16.09	16.61
Other genotypes	Opportunistic	Marseille	41	12.99	10.23	10.58	11.39	12.97	14.65	15.58	16.07
Other genotypes	Opportunistic	Marseille	42	12.64	9.90	10.32	11.10	12.61	14.27	15.14	15.62
Other genotypes	Opportunistic	Marseille	43	12.35	9.65	10.02	10.83	12.31	13.92	14.84	15.33
Other genotypes	Opportunistic	Marseille	44	12.09	9.51	9.82	10.60	12.05	13.62	14.56	15.03
Other genotypes	Opportunistic	Marseille	45	11.87	9.27	9.65	10.40	11.84	13.40	14.30	14.78

Table S9 Posterior expected HR HPV cervical infection prevalence (in %) in major French cities, stratified by type of test, city, and age. Table counterpart to Figure 3A. *(continued)*

				Posterior distribution							
					Percentiles						
Virus	Test	City	Age	Average	0.01	0.025	0.1	0.5	0.9	0.975	0.99
Other genotypes	Opportunistic	Marseille	46	11.68	9.11	9.51	10.23	11.65	13.22	14.14	14.55
Other genotypes	Opportunistic	Marseille	47	11.53	9.00	9.41	10.09	11.49	13.02	13.91	14.35
Other genotypes	Opportunistic	Marseille	48	11.41	8.90	9.27	9.99	11.36	12.90	13.79	14.19
Other genotypes	Opportunistic	Marseille	49	11.30	8.85	9.18	9.89	11.27	12.76	13.63	14.07
Other genotypes	Opportunistic	Marseille	50	11.19	8.78	9.05	9.80	11.17	12.64	13.48	14.00
Other genotypes	Opportunistic	Marseille	51	11.07	8.66	8.96	9.67	11.04	12.54	13.37	13.79
Other genotypes	Opportunistic	Marseille	52	10.89	8.58	8.79	9.53	10.88	12.30	13.15	13.58
Other genotypes	Opportunistic	Marseille	53	10.69	8.39	8.67	9.35	10.66	12.07	12.95	13.44
Other genotypes	Opportunistic	Marseille	54	10.43	8.17	8.50	9.12	10.41	11.80	12.62	13.18
Other genotypes	Opportunistic	Marseille	55	10.19	7.92	8.28	8.92	10.17	11.55	12.30	12.78
Other genotypes	Opportunistic	Marseille	56	9.96	7.74	8.07	8.70	9.94	11.29	12.10	12.53
Other genotypes	Opportunistic	Marseille	57	9.71	7.50	7.91	8.48	9.69	11.03	11.82	12.24
Other genotypes	Opportunistic	Marseille	58	9.50	7.37	7.70	8.31	9.47	10.78	11.56	11.97
Other genotypes	Opportunistic	Marseille	59	9.34	7.28	7.56	8.13	9.30	10.59	11.38	11.75
Other genotypes	Opportunistic	Marseille	60	9.22	7.19	7.47	8.01	9.20	10.45	11.20	11.63
Other genotypes	Opportunistic	Marseille	61	9.15	7.11	7.43	7.95	9.13	10.38	11.11	11.56
Other genotypes	Opportunistic	Marseille	62	9.14	7.09	7.43	7.96	9.11	10.37	11.05	11.58
Other genotypes	Opportunistic	Marseille	63	9.19	7.14	7.43	7.98	9.16	10.44	11.11	11.58
Other genotypes	Opportunistic	Marseille	64	9.30	7.23	7.53	8.08	9.27	10.57	11.26	11.70
Other genotypes	Opportunistic	Marseille	65	9.46	7.30	7.62	8.18	9.42	10.79	11.51	11.97
Other genotypes	Opportunistic	Marseille	66	9.65	7.38	7.70	8.31	9.59	11.09	11.86	12.43
Other genotypes	Opportunistic	Toulouse	30	19.46	12.21	13.05	15.14	19.27	24.23	26.94	28.53
Other genotypes	Opportunistic	Toulouse	31	18.56	11.71	12.40	14.42	18.35	23.16	25.83	27.31
Other genotypes	Opportunistic	Toulouse	32	17.58	11.07	11.67	13.61	17.41	21.95	24.58	25.95
Other genotypes	Opportunistic	Toulouse	33	16.59	10.36	10.93	12.85	16.40	20.78	23.22	24.60
Other genotypes	Opportunistic	Toulouse	34	15.74	9.75	10.36	12.14	15.54	19.78	22.10	23.41
Other genotypes	Opportunistic	Toulouse	35	15.04	9.35	9.85	11.57	14.87	18.90	21.17	22.48
Other genotypes	Opportunistic	Toulouse	36	14.41	8.85	9.41	11.04	14.22	18.16	20.40	21.72
Other genotypes	Opportunistic	Toulouse	37	13.90	8.52	9.04	10.63	13.69	17.50	19.82	21.03

Table S9 Posterior expected HR HPV cervical infection prevalence (in %) in major French cities, stratified by type of test, city, and age. Table counterpart to Figure 3A. ***(continued)***

				Posterior distribution							
					Percentiles						
Virus	Test	City	Age	Average	0.01	0.025	0.1	0.5	0.9	0.975	0.99
Other genotypes	Opportunistic	Toulouse	38	13.43	8.16	8.73	10.28	13.25	16.97	19.09	20.35
Other genotypes	Opportunistic	Toulouse	39	12.98	7.91	8.45	9.92	12.80	16.39	18.49	19.86
Other genotypes	Opportunistic	Toulouse	40	12.53	7.66	8.06	9.57	12.35	15.81	17.93	19.21
Other genotypes	Opportunistic	Toulouse	41	12.13	7.41	7.81	9.26	11.94	15.35	17.32	18.55
Other genotypes	Opportunistic	Toulouse	42	11.80	7.18	7.61	8.99	11.62	14.95	16.87	18.04
Other genotypes	Opportunistic	Toulouse	43	11.52	7.06	7.46	8.77	11.34	14.63	16.51	17.69
Other genotypes	Opportunistic	Toulouse	44	11.28	6.84	7.27	8.62	11.10	14.31	16.13	17.30
Other genotypes	Opportunistic	Toulouse	45	11.07	6.70	7.12	8.44	10.88	14.03	15.88	17.02
Other genotypes	Opportunistic	Toulouse	46	10.90	6.54	7.00	8.30	10.72	13.84	15.64	16.80
Other genotypes	Opportunistic	Toulouse	47	10.75	6.45	6.93	8.20	10.55	13.67	15.40	16.59
Other genotypes	Opportunistic	Toulouse	48	10.64	6.42	6.85	8.11	10.45	13.56	15.34	16.48
Other genotypes	Opportunistic	Toulouse	49	10.54	6.35	6.80	8.00	10.35	13.45	15.12	16.34
Other genotypes	Opportunistic	Toulouse	50	10.44	6.25	6.72	7.93	10.25	13.29	15.09	16.25
Other genotypes	Opportunistic	Toulouse	51	10.32	6.20	6.61	7.82	10.14	13.15	14.91	15.96
Other genotypes	Opportunistic	Toulouse	52	10.16	6.10	6.50	7.69	9.98	12.93	14.68	15.82
Other genotypes	Opportunistic	Toulouse	53	9.97	6.01	6.41	7.55	9.79	12.65	14.40	15.48
Other genotypes	Opportunistic	Toulouse	54	9.73	5.84	6.23	7.37	9.56	12.38	14.02	15.04
Other genotypes	Opportunistic	Toulouse	55	9.50	5.67	6.07	7.19	9.34	12.12	13.81	14.74
Other genotypes	Opportunistic	Toulouse	56	9.28	5.55	5.96	7.01	9.11	11.86	13.52	14.43
Other genotypes	Opportunistic	Toulouse	57	9.05	5.45	5.81	6.83	8.89	11.57	13.13	14.16
Other genotypes	Opportunistic	Toulouse	58	8.86	5.30	5.69	6.69	8.70	11.31	12.91	13.92
Other genotypes	Opportunistic	Toulouse	59	8.70	5.20	5.55	6.57	8.57	11.12	12.71	13.63
Other genotypes	Opportunistic	Toulouse	60	8.59	5.12	5.48	6.47	8.45	10.99	12.53	13.39
Other genotypes	Opportunistic	Toulouse	61	8.53	5.10	5.42	6.43	8.38	10.91	12.44	13.33
Other genotypes	Opportunistic	Toulouse	62	8.52	5.06	5.40	6.43	8.37	10.89	12.46	13.28
Other genotypes	Opportunistic	Toulouse	63	8.57	5.07	5.45	6.44	8.41	10.97	12.52	13.39
Other genotypes	Opportunistic	Toulouse	64	8.67	5.17	5.51	6.53	8.52	11.09	12.64	13.56
Other genotypes	Opportunistic	Toulouse	65	8.82	5.26	5.56	6.63	8.65	11.30	12.85	13.77
Other genotypes	Opportunistic	Toulouse	66	9.00	5.30	5.68	6.71	8.82	11.56	13.19	14.17

Table S9 Posterior expected HR HPV cervical infection prevalence (in %) in major French cities, stratified by type of test, city, and age. Table counterpart to Figure 3A. ***(continued)***

				Posterior distribution							
					Percentiles						
Virus	Test	City	Age	Average	0.01	0.025	0.1	0.5	0.9	0.975	0.99
Other genotypes	Opportunistic	Bordeaux	30	21.29	16.78	17.43	18.56	21.13	24.21	25.99	27.04
Other genotypes	Opportunistic	Bordeaux	31	20.32	15.98	16.63	17.70	20.21	23.12	24.86	25.89
Other genotypes	Opportunistic	Bordeaux	32	19.27	15.14	15.73	16.74	19.13	21.91	23.61	24.64
Other genotypes	Opportunistic	Bordeaux	33	18.20	14.28	14.79	15.78	18.10	20.77	22.37	23.44
Other genotypes	Opportunistic	Bordeaux	34	17.28	13.51	14.06	14.98	17.16	19.77	21.35	22.31
Other genotypes	Opportunistic	Bordeaux	35	16.52	12.91	13.43	14.30	16.40	18.93	20.46	21.44
Other genotypes	Opportunistic	Bordeaux	36	15.84	12.34	12.80	13.67	15.71	18.16	19.65	20.60
Other genotypes	Opportunistic	Bordeaux	37	15.28	11.90	12.31	13.20	15.16	17.53	18.97	19.85
Other genotypes	Opportunistic	Bordeaux	38	14.77	11.48	11.91	12.74	14.65	16.97	18.33	19.31
Other genotypes	Opportunistic	Bordeaux	39	14.29	11.10	11.48	12.31	14.17	16.42	17.77	18.58
Other genotypes	Opportunistic	Bordeaux	40	13.79	10.63	11.05	11.86	13.67	15.85	17.17	17.98
Other genotypes	Opportunistic	Bordeaux	41	13.36	10.23	10.70	11.47	13.25	15.35	16.61	17.33
Other genotypes	Opportunistic	Bordeaux	42	13.00	9.99	10.42	11.15	12.89	14.95	16.17	16.98
Other genotypes	Opportunistic	Bordeaux	43	12.70	9.78	10.16	10.88	12.59	14.61	15.76	16.54
Other genotypes	Opportunistic	Bordeaux	44	12.43	9.56	9.96	10.67	12.32	14.31	15.48	16.20
Other genotypes	Opportunistic	Bordeaux	45	12.20	9.41	9.75	10.48	12.12	14.07	15.15	15.86
Other genotypes	Opportunistic	Bordeaux	46	12.02	9.24	9.64	10.31	11.93	13.89	14.95	15.75
Other genotypes	Opportunistic	Bordeaux	47	11.86	9.11	9.47	10.16	11.76	13.69	14.81	15.57
Other genotypes	Opportunistic	Bordeaux	48	11.73	8.97	9.37	10.08	11.63	13.55	14.66	15.40
Other genotypes	Opportunistic	Bordeaux	49	11.62	8.90	9.27	9.97	11.52	13.40	14.57	15.23
Other genotypes	Opportunistic	Bordeaux	50	11.51	8.81	9.17	9.87	11.41	13.27	14.38	15.12
Other genotypes	Opportunistic	Bordeaux	51	11.39	8.70	9.10	9.76	11.29	13.13	14.25	14.91
Other genotypes	Opportunistic	Bordeaux	52	11.21	8.59	8.94	9.60	11.10	12.93	14.02	14.70
Other genotypes	Opportunistic	Bordeaux	53	11.00	8.42	8.77	9.43	10.90	12.69	13.82	14.43
Other genotypes	Opportunistic	Bordeaux	54	10.74	8.23	8.57	9.20	10.64	12.41	13.46	14.07
Other genotypes	Opportunistic	Bordeaux	55	10.48	8.02	8.33	8.97	10.38	12.13	13.17	13.73
Other genotypes	Opportunistic	Bordeaux	56	10.25	7.82	8.19	8.77	10.15	11.86	12.88	13.45
Other genotypes	Opportunistic	Bordeaux	57	10.00	7.66	7.97	8.55	9.91	11.55	12.59	13.14
Other genotypes	Opportunistic	Bordeaux	58	9.78	7.53	7.80	8.36	9.68	11.31	12.34	12.89

Table S9 **Posterior expected HR HPV cervical infection prevalence (in %) in major French cities, stratified by type of test, city, and age. Table counterpart to Figure 3A.** ***(continued)***

				Posterior distribution							
					Percentiles						
Virus	Test	City	Age	Average	0.01	0.025	0.1	0.5	0.9	0.975	0.99
Other genotypes	Opportunistic	Bordeaux	59	9.61	7.38	7.65	8.21	9.53	11.10	12.15	12.59
Other genotypes	Opportunistic	Bordeaux	60	9.49	7.25	7.57	8.09	9.41	10.97	12.00	12.52
Other genotypes	Opportunistic	Bordeaux	61	9.42	7.22	7.51	8.02	9.33	10.89	11.87	12.46
Other genotypes	Opportunistic	Bordeaux	62	9.41	7.18	7.48	8.01	9.32	10.91	11.91	12.45
Other genotypes	Opportunistic	Bordeaux	63	9.46	7.22	7.52	8.04	9.37	11.00	11.93	12.52
Other genotypes	Opportunistic	Bordeaux	64	9.57	7.29	7.58	8.14	9.49	11.14	12.00	12.67
Other genotypes	Opportunistic	Bordeaux	65	9.73	7.34	7.68	8.25	9.65	11.34	12.29	12.90
Other genotypes	Opportunistic	Bordeaux	66	9.94	7.34	7.74	8.38	9.84	11.64	12.65	13.22
Other genotypes	Opportunistic	Montpellier	30	22.37	15.23	16.18	18.00	22.14	26.98	30.09	31.68
Other genotypes	Opportunistic	Montpellier	31	21.37	14.42	15.39	17.15	21.16	25.81	28.62	30.34
Other genotypes	Opportunistic	Montpellier	32	20.28	13.69	14.53	16.24	20.10	24.50	27.37	28.84
Other genotypes	Opportunistic	Montpellier	33	19.18	12.84	13.65	15.34	18.96	23.27	26.03	27.39
Other genotypes	Opportunistic	Montpellier	34	18.22	12.16	12.92	14.51	18.00	22.18	24.86	26.06
Other genotypes	Opportunistic	Montpellier	35	17.43	11.69	12.34	13.84	17.22	21.24	23.81	25.23
Other genotypes	Opportunistic	Montpellier	36	16.72	11.23	11.82	13.24	16.50	20.43	22.82	24.27
Other genotypes	Opportunistic	Montpellier	37	16.14	10.67	11.40	12.76	15.93	19.73	22.16	23.41
Other genotypes	Opportunistic	Montpellier	38	15.61	10.30	11.03	12.32	15.40	19.11	21.43	22.85
Other genotypes	Opportunistic	Montpellier	39	15.10	9.93	10.64	11.87	14.92	18.48	20.75	22.14
Other genotypes	Opportunistic	Montpellier	40	14.59	9.60	10.24	11.47	14.38	17.90	20.02	21.33
Other genotypes	Opportunistic	Montpellier	41	14.13	9.25	9.94	11.12	13.95	17.32	19.49	20.84
Other genotypes	Opportunistic	Montpellier	42	13.75	9.03	9.63	10.82	13.58	16.90	19.05	20.37
Other genotypes	Opportunistic	Montpellier	43	13.44	8.78	9.38	10.56	13.25	16.52	18.65	19.78
Other genotypes	Opportunistic	Montpellier	44	13.16	8.56	9.20	10.33	12.98	16.18	18.24	19.44
Other genotypes	Opportunistic	Montpellier	45	12.92	8.39	9.04	10.15	12.74	15.91	17.95	19.12
Other genotypes	Opportunistic	Montpellier	46	12.73	8.31	8.90	9.98	12.51	15.71	17.75	18.77
Other genotypes	Opportunistic	Montpellier	47	12.56	8.22	8.75	9.87	12.36	15.47	17.48	18.55
Other genotypes	Opportunistic	Montpellier	48	12.43	8.16	8.64	9.77	12.22	15.31	17.31	18.25
Other genotypes	Opportunistic	Montpellier	49	12.32	8.12	8.57	9.67	12.11	15.18	17.21	18.05
Other genotypes	Opportunistic	Montpellier	50	12.20	8.02	8.48	9.56	12.00	15.07	17.02	17.98

Table S9 Posterior expected HR HPV cervical infection prevalence (in %) in major French cities, stratified by type of test, city, and age. Table counterpart to Figure 3A. ***(continued)***

				Posterior distribution							
					Percentiles						
Virus	Test	City	Age	Average	0.01	0.025	0.1	0.5	0.9	0.975	0.99
Other genotypes	Opportunistic	Montpellier	51	12.07	7.92	8.37	9.43	11.88	14.89	16.82	17.86
Other genotypes	Opportunistic	Montpellier	52	11.88	7.69	8.24	9.27	11.68	14.67	16.54	17.64
Other genotypes	Opportunistic	Montpellier	53	11.67	7.57	8.09	9.10	11.49	14.42	16.25	17.32
Other genotypes	Opportunistic	Montpellier	54	11.38	7.39	7.88	8.90	11.21	14.11	15.86	17.07
Other genotypes	Opportunistic	Montpellier	55	11.12	7.22	7.70	8.70	10.96	13.79	15.57	16.61
Other genotypes	Opportunistic	Montpellier	56	10.87	7.10	7.53	8.47	10.70	13.47	15.24	16.27
Other genotypes	Opportunistic	Montpellier	57	10.61	6.89	7.37	8.25	10.45	13.11	14.87	15.77
Other genotypes	Opportunistic	Montpellier	58	10.38	6.75	7.22	8.07	10.23	12.85	14.55	15.52
Other genotypes	Opportunistic	Montpellier	59	10.20	6.57	7.06	7.90	10.04	12.64	14.35	15.24
Other genotypes	Opportunistic	Montpellier	60	10.08	6.53	6.97	7.81	9.93	12.47	14.16	15.07
Other genotypes	Opportunistic	Montpellier	61	10.00	6.49	6.90	7.75	9.87	12.35	14.10	14.90
Other genotypes	Opportunistic	Montpellier	62	9.99	6.47	6.87	7.75	9.85	12.35	14.03	14.92
Other genotypes	Opportunistic	Montpellier	63	10.04	6.49	6.91	7.79	9.90	12.43	14.15	15.04
Other genotypes	Opportunistic	Montpellier	64	10.16	6.53	7.05	7.88	10.03	12.61	14.31	15.24
Other genotypes	Opportunistic	Montpellier	65	10.33	6.49	7.16	7.99	10.20	12.84	14.53	15.52
Other genotypes	Opportunistic	Montpellier	66	10.54	6.69	7.26	8.15	10.42	13.13	14.87	15.98
Other genotypes	Opportunistic	Rennes	30	22.70	18.13	18.94	20.17	22.62	25.32	26.92	27.64
Other genotypes	Opportunistic	Rennes	31	21.68	17.40	18.02	19.27	21.59	24.19	25.69	26.39
Other genotypes	Opportunistic	Rennes	32	20.58	16.51	17.11	18.26	20.51	22.99	24.44	25.20
Other genotypes	Opportunistic	Rennes	33	19.46	15.55	16.12	17.24	19.36	21.73	23.09	23.90
Other genotypes	Opportunistic	Rennes	34	18.49	14.69	15.24	16.38	18.42	20.68	21.96	22.73
Other genotypes	Opportunistic	Rennes	35	17.69	14.03	14.59	15.64	17.62	19.83	21.08	21.77
Other genotypes	Opportunistic	Rennes	36	16.97	13.42	13.98	14.99	16.91	19.04	20.24	20.99
Other genotypes	Opportunistic	Rennes	37	16.38	12.90	13.51	14.47	16.29	18.38	19.65	20.33
Other genotypes	Opportunistic	Rennes	38	15.83	12.55	13.02	13.97	15.75	17.79	18.94	19.59
Other genotypes	Opportunistic	Rennes	39	15.32	12.14	12.62	13.51	15.25	17.23	18.39	18.99
Other genotypes	Opportunistic	Rennes	40	14.80	11.67	12.19	13.03	14.73	16.66	17.78	18.30
Other genotypes	Opportunistic	Rennes	41	14.34	11.30	11.78	12.59	14.26	16.14	17.28	17.75
Other genotypes	Opportunistic	Rennes	42	13.95	11.02	11.48	12.24	13.87	15.71	16.82	17.29

Table S9 Posterior expected HR HPV cervical infection prevalence (in %) in major French cities, stratified by type of test, city, and age. Table counterpart to Figure 3A. ***(continued)***

				Posterior distribution							
					Percentiles						
Virus	Test	City	Age	Average	0.01	0.025	0.1	0.5	0.9	0.975	0.99
Other genotypes	Opportunistic	Rennes	43	13.63	10.72	11.21	11.97	13.57	15.37	16.42	16.91
Other genotypes	Opportunistic	Rennes	44	13.35	10.51	10.93	11.71	13.29	15.08	16.08	16.60
Other genotypes	Opportunistic	Rennes	45	13.11	10.30	10.71	11.50	13.06	14.79	15.77	16.29
Other genotypes	Opportunistic	Rennes	46	12.91	10.17	10.51	11.32	12.86	14.56	15.52	16.05
Other genotypes	Opportunistic	Rennes	47	12.74	10.04	10.33	11.17	12.68	14.38	15.38	15.90
Other genotypes	Opportunistic	Rennes	48	12.61	9.87	10.24	11.06	12.55	14.22	15.25	15.73
Other genotypes	Opportunistic	Rennes	49	12.49	9.77	10.14	10.97	12.45	14.10	15.09	15.66
Other genotypes	Opportunistic	Rennes	50	12.37	9.72	10.03	10.86	12.32	13.96	14.94	15.47
Other genotypes	Opportunistic	Rennes	51	12.24	9.62	9.95	10.74	12.19	13.84	14.81	15.33
Other genotypes	Opportunistic	Rennes	52	12.05	9.51	9.78	10.57	12.00	13.63	14.59	15.07
Other genotypes	Opportunistic	Rennes	53	11.83	9.28	9.63	10.36	11.79	13.39	14.33	14.79
Other genotypes	Opportunistic	Rennes	54	11.55	9.05	9.39	10.10	11.49	13.10	13.97	14.50
Other genotypes	Opportunistic	Rennes	55	11.28	8.79	9.17	9.86	11.21	12.79	13.64	14.12
Other genotypes	Opportunistic	Rennes	56	11.03	8.63	8.93	9.63	10.98	12.50	13.39	13.79
Other genotypes	Opportunistic	Rennes	57	10.76	8.37	8.70	9.39	10.71	12.21	13.06	13.47
Other genotypes	Opportunistic	Rennes	58	10.53	8.18	8.51	9.20	10.48	11.96	12.79	13.23
Other genotypes	Opportunistic	Rennes	59	10.35	8.04	8.37	9.03	10.30	11.72	12.57	12.95
Other genotypes	Opportunistic	Rennes	60	10.22	7.90	8.28	8.91	10.17	11.57	12.42	12.82
Other genotypes	Opportunistic	Rennes	61	10.14	7.87	8.23	8.85	10.09	11.48	12.36	12.74
Other genotypes	Opportunistic	Rennes	62	10.13	7.86	8.17	8.81	10.07	11.49	12.31	12.74
Other genotypes	Opportunistic	Rennes	63	10.19	7.86	8.23	8.87	10.12	11.57	12.35	12.87
Other genotypes	Opportunistic	Rennes	64	10.30	8.03	8.33	8.97	10.24	11.72	12.49	13.00
Other genotypes	Opportunistic	Rennes	65	10.48	8.15	8.46	9.11	10.42	11.92	12.74	13.23
Other genotypes	Opportunistic	Rennes	66	10.69	8.24	8.58	9.23	10.62	12.22	13.08	13.53
Other genotypes	Opportunistic	Nantes	30	19.73	12.65	13.61	15.49	19.39	24.48	27.08	28.51
Other genotypes	Opportunistic	Nantes	31	18.82	12.09	12.94	14.72	18.49	23.44	25.94	27.46
Other genotypes	Opportunistic	Nantes	32	17.84	11.36	12.26	13.90	17.52	22.28	24.62	26.15
Other genotypes	Opportunistic	Nantes	33	16.84	10.75	11.49	13.09	16.53	21.06	23.44	25.00
Other genotypes	Opportunistic	Nantes	34	15.97	10.17	10.86	12.37	15.68	19.96	22.34	23.81

Table S9 **Posterior expected HR HPV cervical infection prevalence (in %) in major French cities, stratified by type of test, city, and age. Table counterpart to Figure 3A.** *(continued)*

				Posterior distribution							
					Percentiles						
Virus	Test	City	Age	Average	0.01	0.025	0.1	0.5	0.9	0.975	0.99
Other genotypes	Opportunistic	Nantes	35	15.27	9.69	10.37	11.80	14.98	19.10	21.39	22.90
Other genotypes	Opportunistic	Nantes	36	14.63	9.25	9.91	11.29	14.36	18.29	20.56	21.86
Other genotypes	Opportunistic	Nantes	37	14.11	8.87	9.49	10.89	13.84	17.75	19.90	21.24
Other genotypes	Opportunistic	Nantes	38	13.63	8.53	9.17	10.51	13.37	17.14	19.33	20.55
Other genotypes	Opportunistic	Nantes	39	13.18	8.24	8.86	10.14	12.92	16.61	18.72	19.89
Other genotypes	Opportunistic	Nantes	40	12.72	7.95	8.56	9.77	12.46	16.02	18.09	19.36
Other genotypes	Opportunistic	Nantes	41	12.32	7.69	8.23	9.44	12.07	15.53	17.53	18.75
Other genotypes	Opportunistic	Nantes	42	11.98	7.45	7.97	9.18	11.73	15.16	17.03	18.26
Other genotypes	Opportunistic	Nantes	43	11.70	7.26	7.81	8.97	11.44	14.84	16.64	17.85
Other genotypes	Opportunistic	Nantes	44	11.46	7.14	7.60	8.76	11.22	14.54	16.39	17.46
Other genotypes	Opportunistic	Nantes	45	11.25	6.99	7.46	8.57	10.99	14.28	16.01	17.16
Other genotypes	Opportunistic	Nantes	46	11.07	6.85	7.34	8.44	10.82	14.07	15.82	16.88
Other genotypes	Opportunistic	Nantes	47	10.92	6.77	7.24	8.31	10.68	13.90	15.61	16.74
Other genotypes	Opportunistic	Nantes	48	10.81	6.65	7.17	8.22	10.55	13.75	15.49	16.55
Other genotypes	Opportunistic	Nantes	49	10.71	6.61	7.10	8.16	10.45	13.63	15.35	16.42
Other genotypes	Opportunistic	Nantes	50	10.60	6.52	7.02	8.07	10.38	13.48	15.24	16.28
Other genotypes	Opportunistic	Nantes	51	10.49	6.45	6.96	7.98	10.28	13.34	15.06	16.14
Other genotypes	Opportunistic	Nantes	52	10.32	6.40	6.86	7.85	10.08	13.13	14.78	15.88
Other genotypes	Opportunistic	Nantes	53	10.13	6.26	6.74	7.70	9.91	12.90	14.52	15.47
Other genotypes	Opportunistic	Nantes	54	9.88	6.06	6.54	7.51	9.67	12.60	14.15	15.09
Other genotypes	Opportunistic	Nantes	55	9.65	5.95	6.38	7.32	9.44	12.27	13.86	14.80
Other genotypes	Opportunistic	Nantes	56	9.43	5.82	6.24	7.14	9.22	12.01	13.57	14.50
Other genotypes	Opportunistic	Nantes	57	9.20	5.65	6.06	6.98	8.99	11.73	13.21	14.20
Other genotypes	Opportunistic	Nantes	58	9.00	5.53	5.96	6.83	8.79	11.48	12.95	13.97
Other genotypes	Opportunistic	Nantes	59	8.84	5.44	5.83	6.69	8.63	11.28	12.75	13.66
Other genotypes	Opportunistic	Nantes	60	8.73	5.37	5.75	6.64	8.53	11.15	12.57	13.55
Other genotypes	Opportunistic	Nantes	61	8.66	5.34	5.70	6.58	8.44	11.05	12.46	13.45
Other genotypes	Opportunistic	Nantes	62	8.65	5.33	5.67	6.57	8.44	11.04	12.48	13.38
Other genotypes	Opportunistic	Nantes	63	8.70	5.34	5.70	6.60	8.47	11.11	12.58	13.52

Table S9 Posterior expected HR HPV cervical infection prevalence (in %) in major French cities, stratified by type of test, city, and age. Table counterpart to Figure 3A. ***(continued)***

				Posterior distribution							
					Percentiles						
Virus	Test	City	Age	Average	0.01	0.025	0.1	0.5	0.9	0.975	0.99
Other genotypes	Opportunistic	Nantes	64	8.80	5.42	5.80	6.66	8.57	11.24	12.73	13.61
Other genotypes	Opportunistic	Nantes	65	8.95	5.48	5.90	6.79	8.74	11.43	13.01	13.88
Other genotypes	Opportunistic	Nantes	66	9.14	5.57	6.03	6.91	8.91	11.67	13.35	14.20
Other genotypes	Opportunistic	Lille	30	20.59	14.09	15.02	16.77	20.40	24.67	27.11	28.52
Other genotypes	Opportunistic	Lille	31	19.65	13.34	14.32	15.99	19.43	23.61	25.99	27.21
Other genotypes	Opportunistic	Lille	32	18.63	12.61	13.51	15.09	18.45	22.46	24.66	25.96
Other genotypes	Opportunistic	Lille	33	17.59	11.80	12.74	14.20	17.37	21.28	23.33	24.70
Other genotypes	Opportunistic	Lille	34	16.69	11.28	12.06	13.46	16.49	20.24	22.20	23.62
Other genotypes	Opportunistic	Lille	35	15.96	10.77	11.52	12.82	15.77	19.40	21.21	22.53
Other genotypes	Opportunistic	Lille	36	15.30	10.20	11.01	12.28	15.11	18.62	20.42	21.79
Other genotypes	Opportunistic	Lille	37	14.76	9.79	10.62	11.85	14.60	17.96	19.79	21.02
Other genotypes	Opportunistic	Lille	38	14.26	9.52	10.23	11.41	14.09	17.39	19.09	20.33
Other genotypes	Opportunistic	Lille	39	13.79	9.10	9.87	11.05	13.64	16.81	18.53	19.76
Other genotypes	Opportunistic	Lille	40	13.32	8.82	9.51	10.66	13.13	16.26	17.97	19.15
Other genotypes	Opportunistic	Lille	41	12.89	8.60	9.22	10.28	12.72	15.78	17.30	18.60
Other genotypes	Opportunistic	Lille	42	12.54	8.31	8.95	9.98	12.38	15.37	16.83	18.14
Other genotypes	Opportunistic	Lille	43	12.25	8.11	8.73	9.73	12.08	15.02	16.46	17.66
Other genotypes	Opportunistic	Lille	44	12.00	7.94	8.53	9.55	11.83	14.74	16.16	17.30
Other genotypes	Opportunistic	Lille	45	11.78	7.76	8.33	9.37	11.60	14.50	15.85	16.94
Other genotypes	Opportunistic	Lille	46	11.59	7.65	8.19	9.23	11.42	14.21	15.62	16.75
Other genotypes	Opportunistic	Lille	47	11.44	7.54	8.09	9.11	11.27	14.03	15.46	16.50
Other genotypes	Opportunistic	Lille	48	11.32	7.46	7.98	9.00	11.16	13.87	15.30	16.38
Other genotypes	Opportunistic	Lille	49	11.21	7.37	7.92	8.93	11.05	13.77	15.12	16.28
Other genotypes	Opportunistic	Lille	50	11.11	7.33	7.88	8.84	10.96	13.65	15.02	16.17
Other genotypes	Opportunistic	Lille	51	10.98	7.29	7.74	8.74	10.84	13.50	14.89	16.04
Other genotypes	Opportunistic	Lille	52	10.81	7.14	7.62	8.60	10.65	13.29	14.65	15.83
Other genotypes	Opportunistic	Lille	53	10.61	6.98	7.49	8.42	10.47	13.03	14.41	15.63
Other genotypes	Opportunistic	Lille	54	10.36	6.80	7.28	8.20	10.21	12.73	14.11	15.23
Other genotypes	Opportunistic	Lille	55	10.11	6.62	7.15	7.98	9.97	12.39	13.75	14.84

Table S9 **Posterior expected HR HPV cervical infection prevalence (in %) in major French cities, stratified by type of test, city, and age. Table counterpart to Figure 3A.** ***(continued)***

					Posterior distribution							
						Percentiles						
Virus	Test	City	Age	Average	0.01	0.025	0.1	0.5	0.9	0.975	0.99
Other genotypes	Opportunistic	Lille	56	9.88	6.45	6.99	7.83	9.74	12.12	13.50	14.56
Other genotypes	Opportunistic	Lille	57	9.64	6.32	6.80	7.65	9.52	11.85	13.17	14.13
Other genotypes	Opportunistic	Lille	58	9.43	6.14	6.67	7.46	9.31	11.61	12.79	13.79
Other genotypes	Opportunistic	Lille	59	9.27	6.05	6.55	7.32	9.13	11.40	12.63	13.49
Other genotypes	Opportunistic	Lille	60	9.15	5.92	6.45	7.21	9.01	11.30	12.52	13.42
Other genotypes	Opportunistic	Lille	61	9.08	5.91	6.39	7.15	8.94	11.19	12.41	13.35
Other genotypes	Opportunistic	Lille	62	9.07	5.92	6.36	7.16	8.93	11.18	12.45	13.45
Other genotypes	Opportunistic	Lille	63	9.12	5.97	6.41	7.20	9.00	11.23	12.52	13.45
Other genotypes	Opportunistic	Lille	64	9.23	6.02	6.41	7.29	9.11	11.37	12.68	13.66
Other genotypes	Opportunistic	Lille	65	9.39	6.12	6.57	7.40	9.27	11.61	12.91	13.92
Other genotypes	Opportunistic	Lille	66	9.59	6.22	6.59	7.53	9.44	11.90	13.22	14.34
Other genotypes	Opportunistic	Strasbourg	30	17.83	10.83	11.58	13.50	17.53	22.51	25.58	27.45
Other genotypes	Opportunistic	Strasbourg	31	16.99	10.32	10.97	12.81	16.73	21.46	24.41	26.22
Other genotypes	Opportunistic	Strasbourg	32	16.09	9.72	10.34	12.07	15.82	20.40	23.31	24.97
Other genotypes	Opportunistic	Strasbourg	33	15.17	9.07	9.69	11.31	14.91	19.29	22.11	23.76
Other genotypes	Opportunistic	Strasbourg	34	14.37	8.54	9.14	10.72	14.11	18.31	21.09	22.61
Other genotypes	Opportunistic	Strasbourg	35	13.73	8.15	8.68	10.25	13.49	17.50	20.22	21.62
Other genotypes	Opportunistic	Strasbourg	36	13.14	7.76	8.31	9.82	12.90	16.79	19.42	20.76
Other genotypes	Opportunistic	Strasbourg	37	12.67	7.49	8.01	9.43	12.43	16.21	18.68	20.10
Other genotypes	Opportunistic	Strasbourg	38	12.23	7.22	7.73	9.07	12.01	15.66	18.19	19.39
Other genotypes	Opportunistic	Strasbourg	39	11.82	6.96	7.41	8.77	11.60	15.14	17.57	18.83
Other genotypes	Opportunistic	Strasbourg	40	11.40	6.78	7.12	8.43	11.17	14.66	16.93	18.26
Other genotypes	Opportunistic	Strasbourg	41	11.03	6.54	6.90	8.15	10.80	14.20	16.41	17.73
Other genotypes	Opportunistic	Strasbourg	42	10.73	6.33	6.70	7.92	10.50	13.77	15.98	17.38
Other genotypes	Opportunistic	Strasbourg	43	10.48	6.18	6.55	7.72	10.25	13.47	15.61	17.04
Other genotypes	Opportunistic	Strasbourg	44	10.26	6.00	6.42	7.53	10.04	13.22	15.31	16.74
Other genotypes	Opportunistic	Strasbourg	45	10.06	5.90	6.32	7.38	9.85	13.00	15.07	16.35
Other genotypes	Opportunistic	Strasbourg	46	9.91	5.76	6.22	7.27	9.68	12.79	14.85	16.07
Other genotypes	Opportunistic	Strasbourg	47	9.77	5.68	6.12	7.16	9.55	12.62	14.61	15.82

Table S9 Posterior expected HR HPV cervical infection prevalence (in %) in major French cities, stratified by type of test, city, and age. Table counterpart to Figure 3A. ***(continued)***

				Posterior distribution							
					Percentiles						
Virus	Test	City	Age	Average	0.01	0.025	0.1	0.5	0.9	0.975	0.99
Other genotypes	Opportunistic	Strasbourg	48	9.67	5.59	6.06	7.10	9.44	12.47	14.43	15.62
Other genotypes	Opportunistic	Strasbourg	49	9.58	5.52	5.97	7.02	9.36	12.35	14.39	15.49
Other genotypes	Opportunistic	Strasbourg	50	9.48	5.48	5.92	6.93	9.27	12.22	14.22	15.33
Other genotypes	Opportunistic	Strasbourg	51	9.38	5.42	5.85	6.86	9.18	12.09	14.06	15.20
Other genotypes	Opportunistic	Strasbourg	52	9.22	5.36	5.76	6.76	9.02	11.91	13.82	14.97
Other genotypes	Opportunistic	Strasbourg	53	9.05	5.29	5.64	6.64	8.83	11.69	13.56	14.74
Other genotypes	Opportunistic	Strasbourg	54	8.83	5.16	5.49	6.47	8.63	11.43	13.21	14.43
Other genotypes	Opportunistic	Strasbourg	55	8.62	4.99	5.33	6.29	8.40	11.16	12.93	14.08
Other genotypes	Opportunistic	Strasbourg	56	8.42	4.88	5.22	6.16	8.22	10.94	12.67	13.87
Other genotypes	Opportunistic	Strasbourg	57	8.21	4.75	5.06	5.99	8.02	10.64	12.36	13.48
Other genotypes	Opportunistic	Strasbourg	58	8.03	4.60	4.96	5.86	7.84	10.41	12.10	13.23
Other genotypes	Opportunistic	Strasbourg	59	7.89	4.50	4.88	5.77	7.70	10.20	11.90	12.83
Other genotypes	Opportunistic	Strasbourg	60	7.79	4.46	4.83	5.70	7.59	10.08	11.80	12.78
Other genotypes	Opportunistic	Strasbourg	61	7.73	4.44	4.77	5.67	7.52	10.00	11.69	12.66
Other genotypes	Opportunistic	Strasbourg	62	7.72	4.41	4.76	5.66	7.52	10.00	11.68	12.60
Other genotypes	Opportunistic	Strasbourg	63	7.77	4.45	4.80	5.68	7.57	10.07	11.74	12.81
Other genotypes	Opportunistic	Strasbourg	64	7.86	4.55	4.86	5.74	7.67	10.17	11.98	13.03
Other genotypes	Opportunistic	Strasbourg	65	7.99	4.65	4.95	5.81	7.82	10.37	12.23	13.25
Other genotypes	Opportunistic	Strasbourg	66	8.16	4.71	5.03	5.92	7.99	10.57	12.47	13.56
Other genotypes	Opportunistic	Lyon	30	22.91	15.05	16.25	18.11	22.61	27.79	31.20	32.62
Other genotypes	Opportunistic	Lyon	31	21.90	14.40	15.49	17.24	21.60	26.70	29.94	31.34
Other genotypes	Opportunistic	Lyon	32	20.79	13.68	14.61	16.33	20.51	25.43	28.67	29.83
Other genotypes	Opportunistic	Lyon	33	19.67	12.79	13.78	15.39	19.40	24.14	27.19	28.43
Other genotypes	Opportunistic	Lyon	34	18.69	12.00	13.04	14.61	18.43	22.98	25.97	27.19
Other genotypes	Opportunistic	Lyon	35	17.89	11.50	12.40	13.93	17.66	22.03	25.02	26.20
Other genotypes	Opportunistic	Lyon	36	17.16	10.96	11.86	13.34	16.90	21.18	24.03	25.25
Other genotypes	Opportunistic	Lyon	37	16.58	10.56	11.41	12.86	16.29	20.51	23.26	24.35
Other genotypes	Opportunistic	Lyon	38	16.03	10.20	11.03	12.39	15.77	19.85	22.51	23.74
Other genotypes	Opportunistic	Lyon	39	15.51	9.83	10.66	11.97	15.26	19.23	21.77	23.01

Table S9 **Posterior expected HR HPV cervical infection prevalence (in %) in major French cities, stratified by type of test, city, and age. Table counterpart to Figure 3A.** ***(continued)***

				Posterior distribution							
					Percentiles						
Virus	Test	City	Age	Average	0.01	0.025	0.1	0.5	0.9	0.975	0.99
Other genotypes	Opportunistic	Lyon	40	14.99	9.43	10.30	11.55	14.74	18.63	21.11	22.27
Other genotypes	Opportunistic	Lyon	41	14.52	9.16	9.97	11.18	14.26	18.07	20.53	21.55
Other genotypes	Opportunistic	Lyon	42	14.14	8.89	9.69	10.88	13.87	17.61	20.09	21.04
Other genotypes	Opportunistic	Lyon	43	13.81	8.70	9.43	10.63	13.55	17.21	19.62	20.66
Other genotypes	Opportunistic	Lyon	44	13.53	8.49	9.21	10.41	13.27	16.82	19.25	20.21
Other genotypes	Opportunistic	Lyon	45	13.29	8.31	9.04	10.19	13.01	16.55	18.89	19.88
Other genotypes	Opportunistic	Lyon	46	13.09	8.13	8.90	10.06	12.84	16.34	18.60	19.59
Other genotypes	Opportunistic	Lyon	47	12.91	8.06	8.77	9.92	12.66	16.12	18.36	19.39
Other genotypes	Opportunistic	Lyon	48	12.78	8.02	8.71	9.81	12.54	15.98	18.25	19.26
Other genotypes	Opportunistic	Lyon	49	12.66	7.89	8.64	9.70	12.46	15.82	18.10	19.08
Other genotypes	Opportunistic	Lyon	50	12.54	7.84	8.51	9.63	12.33	15.63	17.93	18.91
Other genotypes	Opportunistic	Lyon	51	12.41	7.77	8.45	9.52	12.20	15.46	17.76	18.78
Other genotypes	Opportunistic	Lyon	52	12.22	7.64	8.30	9.37	11.99	15.23	17.48	18.38
Other genotypes	Opportunistic	Lyon	53	12.00	7.45	8.15	9.18	11.77	14.99	17.26	18.11
Other genotypes	Opportunistic	Lyon	54	11.71	7.26	7.94	8.98	11.49	14.67	16.88	17.69
Other genotypes	Opportunistic	Lyon	55	11.44	7.08	7.73	8.73	11.21	14.32	16.50	17.32
Other genotypes	Opportunistic	Lyon	56	11.19	6.87	7.56	8.54	10.97	14.05	16.05	16.96
Other genotypes	Opportunistic	Lyon	57	10.92	6.71	7.36	8.32	10.68	13.68	15.68	16.62
Other genotypes	Opportunistic	Lyon	58	10.69	6.58	7.20	8.15	10.46	13.43	15.32	16.22
Other genotypes	Opportunistic	Lyon	59	10.50	6.48	7.08	8.01	10.28	13.20	15.08	15.92
Other genotypes	Opportunistic	Lyon	60	10.37	6.43	6.99	7.89	10.16	13.03	14.97	15.79
Other genotypes	Opportunistic	Lyon	61	10.29	6.38	6.92	7.84	10.10	12.93	14.93	15.63
Other genotypes	Opportunistic	Lyon	62	10.28	6.39	6.92	7.83	10.07	12.95	14.92	15.61
Other genotypes	Opportunistic	Lyon	63	10.34	6.39	6.97	7.85	10.13	13.04	14.99	15.68
Other genotypes	Opportunistic	Lyon	64	10.46	6.51	7.04	7.96	10.26	13.21	15.20	15.81
Other genotypes	Opportunistic	Lyon	65	10.63	6.61	7.16	8.09	10.42	13.44	15.32	16.28
Other genotypes	Opportunistic	Lyon	66	10.85	6.74	7.26	8.26	10.60	13.75	15.65	16.72
Other genotypes	Opportunistic	Paris	30	25.77	20.69	21.45	22.85	25.71	28.69	30.37	31.27
Other genotypes	Opportunistic	Paris	31	24.66	19.79	20.46	21.87	24.59	27.51	29.18	29.91

Table S9 **Posterior expected HR HPV cervical infection prevalence (in %) in major French cities, stratified by type of test, city, and age. Table counterpart to Figure 3A.** ***(continued)***

				Posterior distribution							
					Percentiles						
Virus	Test	City	Age	Average	0.01	0.025	0.1	0.5	0.9	0.975	0.99
Other genotypes	Opportunistic	Paris	32	23.46	18.76	19.40	20.80	23.40	26.20	27.80	28.61
Other genotypes	Opportunistic	Paris	33	22.22	17.66	18.35	19.67	22.13	24.87	26.38	27.13
Other genotypes	Opportunistic	Paris	34	21.14	16.76	17.37	18.66	21.07	23.63	25.22	25.90
Other genotypes	Opportunistic	Paris	35	20.26	16.03	16.65	17.85	20.18	22.73	24.14	24.99
Other genotypes	Opportunistic	Paris	36	19.46	15.30	15.93	17.12	19.38	21.88	23.24	24.07
Other genotypes	Opportunistic	Paris	37	18.81	14.87	15.37	16.51	18.75	21.14	22.57	23.30
Other genotypes	Opportunistic	Paris	38	18.20	14.36	14.90	15.99	18.15	20.46	21.91	22.63
Other genotypes	Opportunistic	Paris	39	17.63	13.90	14.42	15.46	17.58	19.84	21.19	21.88
Other genotypes	Opportunistic	Paris	40	17.04	13.43	13.94	14.95	17.00	19.19	20.48	21.23
Other genotypes	Opportunistic	Paris	41	16.52	13.01	13.47	14.47	16.47	18.65	19.88	20.54
Other genotypes	Opportunistic	Paris	42	16.09	12.59	13.09	14.11	16.04	18.15	19.34	20.01
Other genotypes	Opportunistic	Paris	43	15.73	12.40	12.79	13.78	15.69	17.79	18.99	19.49
Other genotypes	Opportunistic	Paris	44	15.42	12.01	12.48	13.49	15.34	17.45	18.64	19.23
Other genotypes	Opportunistic	Paris	45	15.14	11.84	12.31	13.25	15.06	17.14	18.37	18.90
Other genotypes	Opportunistic	Paris	46	14.92	11.59	12.10	13.03	14.83	16.89	18.06	18.72
Other genotypes	Opportunistic	Paris	47	14.72	11.49	11.94	12.86	14.64	16.70	17.83	18.52
Other genotypes	Opportunistic	Paris	48	14.58	11.39	11.81	12.75	14.50	16.50	17.63	18.29
Other genotypes	Opportunistic	Paris	49	14.45	11.33	11.70	12.62	14.37	16.35	17.46	18.11
Other genotypes	Opportunistic	Paris	50	14.31	11.25	11.57	12.49	14.25	16.21	17.28	17.97
Other genotypes	Opportunistic	Paris	51	14.16	11.06	11.47	12.35	14.10	16.05	17.08	17.75
Other genotypes	Opportunistic	Paris	52	13.94	10.88	11.30	12.15	13.89	15.80	16.83	17.46
Other genotypes	Opportunistic	Paris	53	13.70	10.66	11.05	11.91	13.63	15.50	16.65	17.14
Other genotypes	Opportunistic	Paris	54	13.38	10.37	10.76	11.63	13.31	15.16	16.23	16.81
Other genotypes	Opportunistic	Paris	55	13.07	10.10	10.51	11.35	13.00	14.84	15.91	16.44
Other genotypes	Opportunistic	Paris	56	12.78	9.90	10.25	11.09	12.72	14.54	15.51	16.13
Other genotypes	Opportunistic	Paris	57	12.48	9.65	10.01	10.85	12.42	14.17	15.21	15.67
Other genotypes	Opportunistic	Paris	58	12.22	9.46	9.78	10.63	12.15	13.89	14.89	15.45
Other genotypes	Opportunistic	Paris	59	12.01	9.29	9.65	10.41	11.94	13.67	14.72	15.13
Other genotypes	Opportunistic	Paris	60	11.86	9.21	9.54	10.29	11.80	13.45	14.55	14.97

Table S9 Posterior expected HR HPV cervical infection prevalence (in %) in major French cities, stratified by type of test, city, and age. Table counterpart to Figure 3A. ***(continued)***

Virus	Test	City	Age	Posterior distribution							
					Percentiles						
				Average	0.01	0.025	0.1	0.5	0.9	0.975	0.99
Other genotypes	Opportunistic	Paris	61	11.78	9.16	9.51	10.21	11.71	13.37	14.43	14.90
Other genotypes	Opportunistic	Paris	62	11.76	9.12	9.45	10.20	11.70	13.38	14.41	14.89
Other genotypes	Opportunistic	Paris	63	11.83	9.15	9.50	10.25	11.77	13.47	14.44	15.07
Other genotypes	Opportunistic	Paris	64	11.96	9.25	9.58	10.36	11.90	13.61	14.56	15.27
Other genotypes	Opportunistic	Paris	65	12.16	9.32	9.70	10.52	12.11	13.86	14.81	15.58
Other genotypes	Opportunistic	Paris	66	12.41	9.41	9.84	10.66	12.35	14.22	15.26	15.95

Table S10 Posterior difference in expected HR HPV cervical infection prevalence (in percentage points) in major French cities, between opportunistic and organised screening, stratified by type of test, city, and age. Table counterpart to Figure 3B.

Virus	City	Age	Posterior distribution							
				Percentiles						
			Average	0.01	0.025	0.1	0.5	0.9	0.975	0.99
HPV16/18	Bordeaux	30	1.69	-0.16	0.17	0.70	1.69	2.65	3.22	3.55
HPV16/18	Bordeaux	31	1.82	-0.09	0.25	0.83	1.81	2.81	3.41	3.77
HPV16/18	Bordeaux	32	1.94	-0.04	0.35	0.92	1.92	2.95	3.56	3.93
HPV16/18	Bordeaux	33	2.02	0.00	0.39	1.00	2.02	3.07	3.71	4.06
HPV16/18	Bordeaux	34	2.07	0.04	0.44	1.04	2.06	3.11	3.74	4.11
HPV16/18	Bordeaux	35	2.06	0.09	0.48	1.04	2.04	3.08	3.69	4.08
HPV16/18	Bordeaux	36	2.02	0.12	0.49	1.03	2.01	3.02	3.60	4.05
HPV16/18	Bordeaux	37	1.96	0.13	0.51	1.00	1.96	2.95	3.52	3.93
HPV16/18	Bordeaux	38	1.91	0.07	0.49	0.97	1.91	2.85	3.44	3.81
HPV16/18	Bordeaux	39	1.86	0.07	0.45	0.93	1.85	2.78	3.32	3.70
HPV16/18	Bordeaux	40	1.80	0.08	0.42	0.91	1.79	2.71	3.24	3.58
HPV16/18	Bordeaux	41	1.74	0.09	0.39	0.88	1.74	2.62	3.18	3.44
HPV16/18	Bordeaux	42	1.68	0.08	0.37	0.84	1.68	2.54	3.07	3.35
HPV16/18	Bordeaux	43	1.63	0.08	0.35	0.82	1.63	2.45	2.96	3.26

Table S10 Posterior difference in expected HR HPV cervical infection prevalence (in percentage points) in major French cities, between opportunistic and organised screening, stratified by type of test, city, and age. Table counterpart to Figure 3B. ***(continued)***

			Posterior distribution							
				Percentiles						
Virus	City	Age	Average	0.01	0.025	0.1	0.5	0.9	0.975	0.99
HPV16/18	Bordeaux	44	1.58	0.05	0.32	0.78	1.58	2.38	2.88	3.15
HPV16/18	Bordeaux	45	1.54	0.06	0.30	0.75	1.54	2.33	2.81	3.09
HPV16/18	Bordeaux	46	1.49	0.04	0.27	0.72	1.49	2.27	2.74	3.02
HPV16/18	Bordeaux	47	1.45	0.02	0.25	0.69	1.44	2.21	2.68	2.94
HPV16/18	Bordeaux	48	1.41	-0.01	0.22	0.67	1.40	2.15	2.62	2.85
HPV16/18	Bordeaux	49	1.36	-0.03	0.19	0.65	1.36	2.10	2.56	2.79
HPV16/18	Bordeaux	50	1.32	-0.03	0.18	0.62	1.33	2.05	2.49	2.75
HPV16/18	Bordeaux	51	1.28	-0.05	0.14	0.59	1.28	1.99	2.44	2.68
HPV16/18	Bordeaux	52	1.24	-0.09	0.11	0.56	1.24	1.94	2.36	2.59
HPV16/18	Bordeaux	53	1.20	-0.12	0.10	0.53	1.20	1.88	2.29	2.51
HPV16/18	Bordeaux	54	1.15	-0.12	0.09	0.50	1.15	1.82	2.22	2.44
HPV16/18	Bordeaux	55	1.11	-0.14	0.08	0.47	1.11	1.76	2.15	2.37
HPV16/18	Bordeaux	56	1.08	-0.16	0.06	0.44	1.08	1.73	2.11	2.33
HPV16/18	Bordeaux	57	1.05	-0.18	0.03	0.39	1.05	1.70	2.09	2.32
HPV16/18	Bordeaux	58	1.02	-0.20	0.00	0.36	1.03	1.67	2.07	2.30
HPV16/18	Bordeaux	59	1.01	-0.24	-0.02	0.34	1.01	1.66	2.06	2.29
HPV16/18	Bordeaux	60	0.99	-0.26	-0.07	0.32	1.00	1.65	2.05	2.28
HPV16/18	Bordeaux	61	0.98	-0.32	-0.10	0.29	0.98	1.65	2.06	2.25
HPV16/18	Bordeaux	62	0.96	-0.37	-0.12	0.27	0.97	1.64	2.04	2.22
HPV16/18	Bordeaux	63	0.94	-0.44	-0.18	0.24	0.95	1.63	2.01	2.21
HPV16/18	Bordeaux	64	0.91	-0.47	-0.22	0.22	0.92	1.62	2.01	2.22
HPV16/18	Bordeaux	65	0.89	-0.54	-0.28	0.18	0.90	1.60	2.02	2.22
HPV16/18	Bordeaux	66	0.86	-0.62	-0.34	0.13	0.86	1.58	2.00	2.24
HPV16/18	Lille	30	1.89	-0.04	0.29	0.82	1.87	3.05	3.75	4.04
HPV16/18	Lille	31	2.02	0.07	0.39	0.93	1.99	3.20	3.95	4.26
HPV16/18	Lille	32	2.15	0.15	0.47	1.02	2.10	3.36	4.15	4.43
HPV16/18	Lille	33	2.24	0.19	0.55	1.08	2.18	3.49	4.33	4.61
HPV16/18	Lille	34	2.29	0.21	0.63	1.12	2.23	3.54	4.39	4.73
HPV16/18	Lille	35	2.28	0.23	0.64	1.14	2.22	3.52	4.34	4.66

Table S10 Posterior difference in expected HR HPV cervical infection prevalence (in percentage points) in major French cities, between opportunistic and organised screening, stratified by type of test, city, and age. Table counterpart to Figure 3B. ***(continued)***

			Posterior distribution							
				Percentiles						
Virus	City	Age	Average	0.01	0.025	0.1	0.5	0.9	0.975	0.99
HPV16/18	Lille	36	2.23	0.23	0.60	1.12	2.18	3.46	4.23	4.55
HPV16/18	Lille	37	2.17	0.24	0.59	1.08	2.12	3.36	4.12	4.46
HPV16/18	Lille	38	2.12	0.22	0.59	1.05	2.06	3.27	4.02	4.34
HPV16/18	Lille	39	2.06	0.20	0.57	1.02	2.00	3.17	3.92	4.25
HPV16/18	Lille	40	1.99	0.20	0.53	0.98	1.95	3.09	3.82	4.13
HPV16/18	Lille	41	1.93	0.21	0.49	0.95	1.89	3.00	3.72	4.03
HPV16/18	Lille	42	1.87	0.20	0.46	0.91	1.82	2.91	3.61	3.89
HPV16/18	Lille	43	1.81	0.19	0.43	0.88	1.76	2.81	3.52	3.75
HPV16/18	Lille	44	1.76	0.17	0.42	0.85	1.72	2.74	3.43	3.67
HPV16/18	Lille	45	1.71	0.12	0.40	0.83	1.68	2.68	3.35	3.59
HPV16/18	Lille	46	1.66	0.10	0.38	0.80	1.63	2.59	3.25	3.50
HPV16/18	Lille	47	1.61	0.08	0.34	0.78	1.58	2.52	3.15	3.42
HPV16/18	Lille	48	1.57	0.07	0.33	0.74	1.53	2.45	3.05	3.35
HPV16/18	Lille	49	1.52	0.05	0.30	0.71	1.50	2.39	2.97	3.28
HPV16/18	Lille	50	1.48	0.03	0.29	0.68	1.45	2.33	2.91	3.20
HPV16/18	Lille	51	1.44	0.01	0.26	0.65	1.41	2.27	2.84	3.11
HPV16/18	Lille	52	1.40	0.01	0.23	0.62	1.37	2.22	2.76	3.05
HPV16/18	Lille	53	1.35	-0.02	0.21	0.59	1.32	2.15	2.67	2.97
HPV16/18	Lille	54	1.30	-0.04	0.18	0.55	1.27	2.07	2.59	2.88
HPV16/18	Lille	55	1.26	-0.05	0.16	0.52	1.22	2.02	2.53	2.80
HPV16/18	Lille	56	1.22	-0.06	0.14	0.49	1.19	1.97	2.49	2.75
HPV16/18	Lille	57	1.19	-0.09	0.12	0.46	1.16	1.93	2.45	2.72
HPV16/18	Lille	58	1.16	-0.12	0.10	0.44	1.14	1.90	2.45	2.67
HPV16/18	Lille	59	1.14	-0.15	0.05	0.41	1.12	1.89	2.42	2.65
HPV16/18	Lille	60	1.13	-0.19	0.01	0.39	1.11	1.89	2.42	2.67
HPV16/18	Lille	61	1.12	-0.23	-0.03	0.36	1.10	1.90	2.42	2.64
HPV16/18	Lille	62	1.10	-0.29	-0.05	0.33	1.09	1.89	2.40	2.64
HPV16/18	Lille	63	1.08	-0.36	-0.10	0.31	1.07	1.87	2.39	2.63
HPV16/18	Lille	64	1.05	-0.42	-0.11	0.28	1.04	1.85	2.38	2.66

Table S10 Posterior difference in expected HR HPV cervical infection prevalence (in percentage points) in major French cities, between opportunistic and organised screening, stratified by type of test, city, and age. Table counterpart to Figure 3B. ***(continued)***

			Posterior distribution							
				Percentiles						
Virus	City	Age	Average	0.01	0.025	0.1	0.5	0.9	0.975	0.99
HPV16/18	Lille	65	1.03	-0.49	-0.18	0.23	1.02	1.83	2.35	2.65
HPV16/18	Lille	66	1.00	-0.56	-0.23	0.19	0.99	1.81	2.31	2.67
HPV16/18	Lyon	30	1.85	-0.30	0.04	0.67	1.82	3.12	3.92	4.49
HPV16/18	Lyon	31	1.99	-0.22	0.15	0.78	1.94	3.30	4.16	4.72
HPV16/18	Lyon	32	2.11	-0.14	0.24	0.87	2.05	3.45	4.34	4.93
HPV16/18	Lyon	33	2.21	-0.05	0.32	0.94	2.14	3.58	4.51	5.11
HPV16/18	Lyon	34	2.26	-0.02	0.37	0.97	2.18	3.63	4.57	5.13
HPV16/18	Lyon	35	2.24	0.02	0.37	0.97	2.16	3.62	4.52	5.09
HPV16/18	Lyon	36	2.20	0.06	0.37	0.96	2.12	3.55	4.44	5.00
HPV16/18	Lyon	37	2.15	0.07	0.33	0.94	2.07	3.45	4.35	4.81
HPV16/18	Lyon	38	2.09	0.06	0.32	0.93	2.01	3.37	4.24	4.70
HPV16/18	Lyon	39	2.03	0.05	0.33	0.90	1.95	3.28	4.11	4.58
HPV16/18	Lyon	40	1.97	0.03	0.33	0.87	1.90	3.17	3.99	4.48
HPV16/18	Lyon	41	1.91	0.00	0.29	0.84	1.84	3.10	3.86	4.34
HPV16/18	Lyon	42	1.84	-0.02	0.27	0.80	1.79	3.01	3.74	4.21
HPV16/18	Lyon	43	1.78	-0.06	0.24	0.75	1.73	2.92	3.60	4.10
HPV16/18	Lyon	44	1.73	-0.06	0.22	0.72	1.67	2.85	3.51	4.05
HPV16/18	Lyon	45	1.69	-0.08	0.19	0.69	1.62	2.77	3.43	3.94
HPV16/18	Lyon	46	1.64	-0.10	0.17	0.66	1.58	2.70	3.34	3.87
HPV16/18	Lyon	47	1.59	-0.11	0.17	0.63	1.53	2.62	3.27	3.74
HPV16/18	Lyon	48	1.54	-0.11	0.16	0.62	1.49	2.56	3.21	3.64
HPV16/18	Lyon	49	1.50	-0.13	0.14	0.59	1.43	2.50	3.13	3.57
HPV16/18	Lyon	50	1.45	-0.14	0.12	0.55	1.39	2.43	3.06	3.47
HPV16/18	Lyon	51	1.41	-0.16	0.11	0.54	1.35	2.37	2.96	3.38
HPV16/18	Lyon	52	1.37	-0.19	0.10	0.52	1.32	2.30	2.89	3.33
HPV16/18	Lyon	53	1.32	-0.20	0.07	0.49	1.27	2.23	2.82	3.21
HPV16/18	Lyon	54	1.27	-0.22	0.03	0.45	1.22	2.15	2.73	3.08
HPV16/18	Lyon	55	1.22	-0.24	0.00	0.43	1.18	2.10	2.66	3.01
HPV16/18	Lyon	56	1.19	-0.28	-0.04	0.39	1.14	2.05	2.58	2.97

Table S10 **Posterior difference in expected HR HPV cervical infection prevalence (in percentage points) in major French cities, between opportunistic and organised screening, stratified by type of test, city, and age. Table counterpart to Figure 3B.** ***(continued)***

			Posterior distribution							
				Percentiles						
Virus	City	Age	Average	0.01	0.025	0.1	0.5	0.9	0.975	0.99
HPV16/18	Lyon	57	1.15	-0.31	-0.08	0.36	1.11	2.00	2.56	2.87
HPV16/18	Lyon	58	1.13	-0.33	-0.10	0.34	1.09	1.97	2.51	2.87
HPV16/18	Lyon	59	1.11	-0.35	-0.12	0.33	1.06	1.94	2.52	2.89
HPV16/18	Lyon	60	1.09	-0.37	-0.17	0.30	1.06	1.92	2.51	2.87
HPV16/18	Lyon	61	1.08	-0.42	-0.21	0.28	1.04	1.92	2.51	2.85
HPV16/18	Lyon	62	1.06	-0.48	-0.26	0.25	1.02	1.90	2.49	2.82
HPV16/18	Lyon	63	1.03	-0.53	-0.29	0.21	1.00	1.89	2.48	2.76
HPV16/18	Lyon	64	1.01	-0.58	-0.33	0.18	0.98	1.88	2.46	2.73
HPV16/18	Lyon	65	0.98	-0.66	-0.38	0.14	0.96	1.86	2.43	2.72
HPV16/18	Lyon	66	0.94	-0.70	-0.44	0.09	0.93	1.84	2.43	2.71
HPV16/18	Marseille	30	1.11	-1.02	-0.65	0.04	1.16	2.13	2.57	2.91
HPV16/18	Marseille	31	1.21	-0.92	-0.58	0.12	1.26	2.22	2.72	3.02
HPV16/18	Marseille	32	1.30	-0.91	-0.53	0.18	1.34	2.34	2.87	3.13
HPV16/18	Marseille	33	1.37	-0.85	-0.47	0.22	1.42	2.43	2.97	3.25
HPV16/18	Marseille	34	1.40	-0.81	-0.42	0.26	1.45	2.47	2.98	3.33
HPV16/18	Marseille	35	1.40	-0.77	-0.40	0.27	1.45	2.45	2.96	3.31
HPV16/18	Marseille	36	1.37	-0.72	-0.39	0.27	1.42	2.41	2.92	3.26
HPV16/18	Marseille	37	1.33	-0.69	-0.39	0.26	1.38	2.33	2.86	3.17
HPV16/18	Marseille	38	1.30	-0.67	-0.38	0.24	1.34	2.27	2.78	3.09
HPV16/18	Marseille	39	1.26	-0.67	-0.37	0.25	1.29	2.20	2.70	2.98
HPV16/18	Marseille	40	1.21	-0.64	-0.37	0.22	1.26	2.14	2.63	2.89
HPV16/18	Marseille	41	1.17	-0.65	-0.38	0.21	1.21	2.09	2.55	2.79
HPV16/18	Marseille	42	1.13	-0.66	-0.38	0.18	1.16	2.03	2.48	2.72
HPV16/18	Marseille	43	1.09	-0.64	-0.38	0.18	1.12	1.96	2.41	2.63
HPV16/18	Marseille	44	1.05	-0.68	-0.39	0.16	1.08	1.91	2.34	2.57
HPV16/18	Marseille	45	1.02	-0.68	-0.40	0.14	1.05	1.87	2.29	2.48
HPV16/18	Marseille	46	0.98	-0.65	-0.40	0.13	1.01	1.82	2.23	2.40
HPV16/18	Marseille	47	0.95	-0.66	-0.39	0.11	0.97	1.76	2.17	2.34
HPV16/18	Marseille	48	0.91	-0.69	-0.42	0.09	0.94	1.71	2.11	2.30

Table S10 Posterior difference in expected HR HPV cervical infection prevalence (in percentage points) in major French cities, between opportunistic and organised screening, stratified by type of test, city, and age. Table counterpart to Figure 3B. ***(continued)***

			Posterior distribution							
				Percentiles						
Virus	City	Age	Average	0.01	0.025	0.1	0.5	0.9	0.975	0.99
HPV16/18	Marseille	49	0.88	-0.69	-0.44	0.07	0.91	1.67	2.04	2.22
HPV16/18	Marseille	50	0.85	-0.71	-0.45	0.05	0.87	1.63	1.99	2.17
HPV16/18	Marseille	51	0.82	-0.73	-0.45	0.03	0.85	1.58	1.94	2.13
HPV16/18	Marseille	52	0.79	-0.75	-0.46	0.01	0.81	1.53	1.89	2.09
HPV16/18	Marseille	53	0.75	-0.78	-0.48	0.00	0.78	1.49	1.84	2.00
HPV16/18	Marseille	54	0.72	-0.77	-0.50	-0.03	0.74	1.44	1.77	1.96
HPV16/18	Marseille	55	0.68	-0.77	-0.50	-0.05	0.71	1.40	1.73	1.89
HPV16/18	Marseille	56	0.65	-0.79	-0.52	-0.08	0.68	1.37	1.70	1.85
HPV16/18	Marseille	57	0.63	-0.83	-0.53	-0.11	0.65	1.34	1.66	1.82
HPV16/18	Marseille	58	0.60	-0.86	-0.58	-0.13	0.63	1.31	1.64	1.80
HPV16/18	Marseille	59	0.59	-0.90	-0.61	-0.16	0.61	1.29	1.65	1.79
HPV16/18	Marseille	60	0.57	-0.93	-0.63	-0.17	0.60	1.29	1.64	1.79
HPV16/18	Marseille	61	0.55	-0.97	-0.68	-0.21	0.59	1.28	1.64	1.77
HPV16/18	Marseille	62	0.54	-1.01	-0.70	-0.25	0.57	1.27	1.61	1.79
HPV16/18	Marseille	63	0.51	-1.05	-0.77	-0.29	0.56	1.26	1.61	1.80
HPV16/18	Marseille	64	0.49	-1.10	-0.83	-0.32	0.54	1.25	1.60	1.79
HPV16/18	Marseille	65	0.47	-1.15	-0.88	-0.38	0.51	1.23	1.60	1.78
HPV16/18	Marseille	66	0.44	-1.21	-0.95	-0.42	0.48	1.22	1.59	1.80
HPV16/18	Montpellier	30	1.70	-0.28	0.02	0.58	1.68	2.87	3.53	3.89
HPV16/18	Montpellier	31	1.83	-0.18	0.12	0.69	1.80	3.02	3.70	4.10
HPV16/18	Montpellier	32	1.95	-0.10	0.19	0.79	1.91	3.17	3.89	4.33
HPV16/18	Montpellier	33	2.04	0.00	0.23	0.85	1.99	3.29	4.01	4.47
HPV16/18	Montpellier	34	2.09	0.02	0.29	0.89	2.03	3.32	4.07	4.56
HPV16/18	Montpellier	35	2.08	0.00	0.30	0.90	2.02	3.29	4.02	4.55
HPV16/18	Montpellier	36	2.04	0.04	0.29	0.90	1.99	3.24	3.93	4.47
HPV16/18	Montpellier	37	1.98	0.04	0.30	0.87	1.94	3.15	3.84	4.33
HPV16/18	Montpellier	38	1.93	0.01	0.29	0.85	1.89	3.07	3.75	4.22
HPV16/18	Montpellier	39	1.88	-0.02	0.26	0.82	1.83	3.00	3.64	4.11
HPV16/18	Montpellier	40	1.82	-0.03	0.22	0.78	1.77	2.92	3.58	4.00

Table S10 Posterior difference in expected HR HPV cervical infection prevalence (in percentage points) in major French cities, between opportunistic and organised screening, stratified by type of test, city, and age. Table counterpart to Figure 3B. ***(continued)***

			Posterior distribution							
				Percentiles						
Virus	City	Age	Average	0.01	0.025	0.1	0.5	0.9	0.975	0.99
HPV16/18	Montpellier	41	1.76	-0.07	0.20	0.75	1.71	2.84	3.50	3.88
HPV16/18	Montpellier	42	1.70	-0.09	0.19	0.72	1.66	2.73	3.40	3.77
HPV16/18	Montpellier	43	1.64	-0.12	0.18	0.69	1.61	2.64	3.29	3.64
HPV16/18	Montpellier	44	1.60	-0.12	0.16	0.66	1.56	2.59	3.21	3.55
HPV16/18	Montpellier	45	1.55	-0.14	0.14	0.63	1.52	2.52	3.12	3.48
HPV16/18	Montpellier	46	1.51	-0.13	0.12	0.60	1.47	2.44	3.05	3.39
HPV16/18	Montpellier	47	1.46	-0.14	0.11	0.57	1.42	2.38	2.97	3.33
HPV16/18	Montpellier	48	1.42	-0.17	0.09	0.54	1.39	2.33	2.89	3.21
HPV16/18	Montpellier	49	1.37	-0.17	0.08	0.52	1.35	2.27	2.83	3.14
HPV16/18	Montpellier	50	1.33	-0.17	0.07	0.49	1.30	2.20	2.76	3.08
HPV16/18	Montpellier	51	1.29	-0.20	0.06	0.47	1.27	2.15	2.71	3.04
HPV16/18	Montpellier	52	1.25	-0.22	0.03	0.44	1.23	2.10	2.62	2.96
HPV16/18	Montpellier	53	1.21	-0.23	-0.01	0.41	1.20	2.03	2.54	2.87
HPV16/18	Montpellier	54	1.16	-0.24	-0.04	0.38	1.14	1.96	2.47	2.77
HPV16/18	Montpellier	55	1.12	-0.26	-0.06	0.35	1.10	1.90	2.40	2.71
HPV16/18	Montpellier	56	1.08	-0.28	-0.10	0.32	1.06	1.86	2.35	2.63
HPV16/18	Montpellier	57	1.05	-0.31	-0.12	0.30	1.03	1.83	2.31	2.59
HPV16/18	Montpellier	58	1.02	-0.32	-0.13	0.28	1.01	1.81	2.28	2.56
HPV16/18	Montpellier	59	1.00	-0.36	-0.18	0.25	0.99	1.78	2.26	2.54
HPV16/18	Montpellier	60	0.99	-0.40	-0.21	0.23	0.98	1.76	2.27	2.54
HPV16/18	Montpellier	61	0.98	-0.43	-0.24	0.20	0.96	1.76	2.25	2.55
HPV16/18	Montpellier	62	0.96	-0.49	-0.28	0.18	0.94	1.75	2.27	2.52
HPV16/18	Montpellier	63	0.93	-0.58	-0.30	0.15	0.92	1.73	2.24	2.53
HPV16/18	Montpellier	64	0.91	-0.64	-0.36	0.11	0.89	1.71	2.22	2.49
HPV16/18	Montpellier	65	0.88	-0.71	-0.40	0.07	0.87	1.69	2.21	2.48
HPV16/18	Montpellier	66	0.85	-0.76	-0.46	0.02	0.85	1.67	2.20	2.47
HPV16/18	Nantes	30	1.85	0.09	0.37	0.82	1.80	2.98	3.72	4.08
HPV16/18	Nantes	31	1.98	0.19	0.46	0.91	1.92	3.15	3.93	4.34
HPV16/18	Nantes	32	2.10	0.27	0.54	1.01	2.03	3.32	4.09	4.55

Table S10 Posterior difference in expected HR HPV cervical infection prevalence (in percentage points) in major French cities, between opportunistic and organised screening, stratified by type of test, city, and age. Table counterpart to Figure 3B. ***(continued)***

			Posterior distribution							
				Percentiles						
Virus	City	Age	Average	0.01	0.025	0.1	0.5	0.9	0.975	0.99
HPV16/18	Nantes	33	2.20	0.32	0.60	1.08	2.12	3.45	4.23	4.66
HPV16/18	Nantes	34	2.24	0.37	0.64	1.11	2.16	3.49	4.30	4.74
HPV16/18	Nantes	35	2.23	0.37	0.63	1.13	2.14	3.45	4.28	4.75
HPV16/18	Nantes	36	2.19	0.39	0.61	1.11	2.11	3.39	4.19	4.66
HPV16/18	Nantes	37	2.13	0.38	0.62	1.08	2.06	3.31	4.06	4.55
HPV16/18	Nantes	38	2.07	0.39	0.61	1.04	2.00	3.22	3.97	4.43
HPV16/18	Nantes	39	2.01	0.38	0.60	1.01	1.94	3.13	3.85	4.29
HPV16/18	Nantes	40	1.95	0.35	0.57	0.98	1.88	3.05	3.73	4.21
HPV16/18	Nantes	41	1.89	0.32	0.56	0.95	1.82	2.96	3.63	4.06
HPV16/18	Nantes	42	1.83	0.30	0.53	0.92	1.76	2.87	3.55	3.97
HPV16/18	Nantes	43	1.77	0.28	0.49	0.89	1.70	2.79	3.43	3.82
HPV16/18	Nantes	44	1.72	0.26	0.47	0.85	1.65	2.71	3.35	3.72
HPV16/18	Nantes	45	1.68	0.23	0.46	0.82	1.61	2.66	3.27	3.64
HPV16/18	Nantes	46	1.63	0.22	0.44	0.80	1.57	2.59	3.17	3.55
HPV16/18	Nantes	47	1.58	0.20	0.42	0.77	1.53	2.52	3.10	3.48
HPV16/18	Nantes	48	1.54	0.18	0.40	0.75	1.49	2.46	3.00	3.38
HPV16/18	Nantes	49	1.50	0.18	0.36	0.71	1.44	2.40	2.92	3.28
HPV16/18	Nantes	50	1.45	0.17	0.35	0.68	1.40	2.33	2.86	3.18
HPV16/18	Nantes	51	1.41	0.14	0.33	0.65	1.37	2.27	2.80	3.08
HPV16/18	Nantes	52	1.37	0.14	0.30	0.63	1.32	2.21	2.72	3.00
HPV16/18	Nantes	53	1.33	0.13	0.28	0.60	1.28	2.14	2.64	2.96
HPV16/18	Nantes	54	1.28	0.10	0.26	0.57	1.24	2.08	2.55	2.86
HPV16/18	Nantes	55	1.24	0.09	0.24	0.54	1.20	2.02	2.47	2.78
HPV16/18	Nantes	56	1.20	0.06	0.22	0.52	1.16	1.97	2.42	2.75
HPV16/18	Nantes	57	1.17	0.02	0.20	0.49	1.14	1.93	2.38	2.66
HPV16/18	Nantes	58	1.15	-0.02	0.16	0.47	1.11	1.90	2.34	2.63
HPV16/18	Nantes	59	1.13	-0.05	0.13	0.46	1.09	1.88	2.34	2.63
HPV16/18	Nantes	60	1.12	-0.07	0.10	0.44	1.08	1.87	2.34	2.61
HPV16/18	Nantes	61	1.11	-0.09	0.07	0.42	1.07	1.87	2.32	2.60

Table S10 Posterior difference in expected HR HPV cervical infection prevalence (in percentage points) in major French cities, between opportunistic and organised screening, stratified by type of test, city, and age. Table counterpart to Figure 3B. ***(continued)***

			Posterior distribution							
				Percentiles						
Virus	City	Age	Average	0.01	0.025	0.1	0.5	0.9	0.975	0.99
HPV16/18	Nantes	62	1.09	-0.13	0.04	0.41	1.06	1.86	2.30	2.58
HPV16/18	Nantes	63	1.07	-0.20	0.02	0.38	1.04	1.85	2.29	2.57
HPV16/18	Nantes	64	1.05	-0.25	-0.02	0.35	1.02	1.83	2.27	2.57
HPV16/18	Nantes	65	1.02	-0.31	-0.07	0.31	0.99	1.80	2.27	2.52
HPV16/18	Nantes	66	0.99	-0.41	-0.12	0.28	0.96	1.77	2.28	2.52
HPV16/18	Nice	30	1.19	-1.05	-0.56	0.09	1.13	2.35	3.24	3.72
HPV16/18	Nice	31	1.29	-0.97	-0.49	0.16	1.23	2.48	3.40	3.92
HPV16/18	Nice	32	1.38	-0.87	-0.47	0.22	1.31	2.61	3.58	4.10
HPV16/18	Nice	33	1.45	-0.84	-0.41	0.26	1.38	2.71	3.73	4.26
HPV16/18	Nice	34	1.48	-0.81	-0.39	0.27	1.41	2.77	3.76	4.30
HPV16/18	Nice	35	1.47	-0.76	-0.38	0.29	1.40	2.75	3.76	4.24
HPV16/18	Nice	36	1.45	-0.74	-0.37	0.28	1.38	2.69	3.67	4.20
HPV16/18	Nice	37	1.41	-0.74	-0.36	0.29	1.34	2.62	3.57	4.08
HPV16/18	Nice	38	1.37	-0.75	-0.35	0.29	1.30	2.54	3.49	3.99
HPV16/18	Nice	39	1.33	-0.71	-0.35	0.27	1.26	2.49	3.38	3.90
HPV16/18	Nice	40	1.28	-0.75	-0.34	0.26	1.22	2.41	3.29	3.79
HPV16/18	Nice	41	1.24	-0.74	-0.36	0.25	1.17	2.34	3.21	3.73
HPV16/18	Nice	42	1.20	-0.71	-0.36	0.22	1.12	2.29	3.11	3.59
HPV16/18	Nice	43	1.16	-0.72	-0.37	0.20	1.08	2.21	2.98	3.51
HPV16/18	Nice	44	1.12	-0.71	-0.37	0.18	1.05	2.15	2.91	3.44
HPV16/18	Nice	45	1.09	-0.71	-0.38	0.17	1.03	2.09	2.86	3.37
HPV16/18	Nice	46	1.05	-0.69	-0.39	0.15	0.99	2.03	2.77	3.29
HPV16/18	Nice	47	1.02	-0.69	-0.40	0.14	0.96	1.99	2.69	3.22
HPV16/18	Nice	48	0.98	-0.70	-0.40	0.13	0.93	1.94	2.64	3.13
HPV16/18	Nice	49	0.95	-0.70	-0.41	0.11	0.91	1.89	2.56	3.02
HPV16/18	Nice	50	0.92	-0.69	-0.42	0.10	0.88	1.84	2.49	2.97
HPV16/18	Nice	51	0.89	-0.70	-0.43	0.07	0.85	1.79	2.44	2.88
HPV16/18	Nice	52	0.86	-0.71	-0.43	0.05	0.82	1.72	2.39	2.80
HPV16/18	Nice	53	0.82	-0.71	-0.44	0.03	0.79	1.67	2.31	2.73

Table S10 Posterior difference in expected HR HPV cervical infection prevalence (in percentage points) in major French cities, between opportunistic and organised screening, stratified by type of test, city, and age. Table counterpart to Figure 3B. ***(continued)***

			Posterior distribution							
				Percentiles						
Virus	City	Age	Average	0.01	0.025	0.1	0.5	0.9	0.975	0.99
HPV16/18	Nice	54	0.79	-0.71	-0.46	0.00	0.75	1.61	2.26	2.61
HPV16/18	Nice	55	0.76	-0.71	-0.48	-0.01	0.72	1.57	2.20	2.56
HPV16/18	Nice	56	0.73	-0.73	-0.49	-0.03	0.70	1.53	2.15	2.50
HPV16/18	Nice	57	0.70	-0.76	-0.51	-0.05	0.67	1.51	2.11	2.44
HPV16/18	Nice	58	0.68	-0.76	-0.52	-0.07	0.65	1.49	2.06	2.43
HPV16/18	Nice	59	0.67	-0.78	-0.53	-0.09	0.64	1.46	2.02	2.40
HPV16/18	Nice	60	0.65	-0.80	-0.55	-0.11	0.63	1.45	2.02	2.36
HPV16/18	Nice	61	0.64	-0.85	-0.58	-0.13	0.62	1.45	2.02	2.36
HPV16/18	Nice	62	0.62	-0.88	-0.61	-0.17	0.60	1.44	2.00	2.38
HPV16/18	Nice	63	0.60	-0.92	-0.64	-0.19	0.59	1.42	1.97	2.35
HPV16/18	Nice	64	0.58	-0.95	-0.68	-0.22	0.56	1.40	1.99	2.35
HPV16/18	Nice	65	0.56	-1.00	-0.71	-0.26	0.54	1.39	1.95	2.36
HPV16/18	Nice	66	0.54	-1.07	-0.75	-0.29	0.52	1.36	1.94	2.31
HPV16/18	Paris	30	1.97	-0.10	0.23	0.86	1.97	3.05	3.67	3.99
HPV16/18	Paris	31	2.11	0.03	0.38	1.00	2.11	3.22	3.84	4.21
HPV16/18	Paris	32	2.24	0.14	0.48	1.11	2.24	3.39	4.01	4.34
HPV16/18	Paris	33	2.35	0.21	0.56	1.21	2.34	3.51	4.17	4.46
HPV16/18	Paris	34	2.39	0.30	0.59	1.26	2.38	3.56	4.22	4.50
HPV16/18	Paris	35	2.38	0.30	0.60	1.27	2.37	3.53	4.15	4.47
HPV16/18	Paris	36	2.34	0.30	0.59	1.26	2.32	3.46	4.10	4.38
HPV16/18	Paris	37	2.28	0.30	0.60	1.23	2.26	3.37	3.98	4.27
HPV16/18	Paris	38	2.22	0.31	0.59	1.20	2.20	3.28	3.85	4.17
HPV16/18	Paris	39	2.15	0.29	0.58	1.16	2.14	3.19	3.75	4.05
HPV16/18	Paris	40	2.09	0.29	0.54	1.12	2.09	3.08	3.66	3.96
HPV16/18	Paris	41	2.03	0.26	0.54	1.07	2.03	3.00	3.54	3.82
HPV16/18	Paris	42	1.96	0.21	0.49	1.02	1.96	2.91	3.46	3.71
HPV16/18	Paris	43	1.89	0.21	0.46	1.00	1.90	2.82	3.35	3.59
HPV16/18	Paris	44	1.84	0.16	0.46	0.96	1.85	2.75	3.26	3.51
HPV16/18	Paris	45	1.79	0.17	0.44	0.93	1.80	2.68	3.18	3.41

Table S10 **Posterior difference in expected HR HPV cervical infection prevalence (in percentage points) in major French cities, between opportunistic and organised screening, stratified by type of test, city, and age. Table counterpart to Figure 3B.** ***(continued)***

			Posterior distribution							
				Percentiles						
Virus	City	Age	Average	0.01	0.025	0.1	0.5	0.9	0.975	0.99
HPV16/18	Paris	46	1.74	0.15	0.41	0.90	1.75	2.61	3.10	3.32
HPV16/18	Paris	47	1.69	0.13	0.37	0.86	1.69	2.54	3.02	3.25
HPV16/18	Paris	48	1.64	0.11	0.34	0.83	1.64	2.47	2.94	3.17
HPV16/18	Paris	49	1.59	0.09	0.32	0.78	1.59	2.41	2.86	3.12
HPV16/18	Paris	50	1.54	0.07	0.29	0.74	1.55	2.35	2.78	3.03
HPV16/18	Paris	51	1.50	0.05	0.26	0.70	1.50	2.30	2.73	2.94
HPV16/18	Paris	52	1.45	0.05	0.24	0.67	1.45	2.25	2.65	2.90
HPV16/18	Paris	53	1.40	0.02	0.22	0.64	1.41	2.17	2.56	2.79
HPV16/18	Paris	54	1.35	0.00	0.18	0.60	1.35	2.09	2.49	2.73
HPV16/18	Paris	55	1.30	-0.03	0.16	0.56	1.31	2.03	2.44	2.67
HPV16/18	Paris	56	1.26	-0.07	0.14	0.52	1.27	1.99	2.38	2.62
HPV16/18	Paris	57	1.22	-0.11	0.11	0.49	1.24	1.95	2.34	2.60
HPV16/18	Paris	58	1.20	-0.12	0.08	0.46	1.20	1.91	2.33	2.58
HPV16/18	Paris	59	1.18	-0.15	0.06	0.42	1.18	1.89	2.32	2.58
HPV16/18	Paris	60	1.16	-0.21	0.02	0.40	1.17	1.89	2.34	2.59
HPV16/18	Paris	61	1.14	-0.24	-0.01	0.37	1.15	1.90	2.34	2.58
HPV16/18	Paris	62	1.12	-0.28	-0.04	0.34	1.13	1.89	2.33	2.56
HPV16/18	Paris	63	1.10	-0.34	-0.10	0.29	1.11	1.87	2.31	2.59
HPV16/18	Paris	64	1.07	-0.41	-0.14	0.26	1.08	1.86	2.29	2.56
HPV16/18	Paris	65	1.04	-0.50	-0.20	0.21	1.05	1.84	2.28	2.54
HPV16/18	Paris	66	1.00	-0.56	-0.28	0.16	1.02	1.83	2.30	2.49
HPV16/18	Rennes	30	2.50	0.65	0.99	1.47	2.49	3.54	4.07	4.48
HPV16/18	Rennes	31	2.67	0.77	1.10	1.63	2.67	3.74	4.29	4.71
HPV16/18	Rennes	32	2.82	0.88	1.22	1.75	2.83	3.92	4.50	4.93
HPV16/18	Rennes	33	2.94	1.00	1.31	1.85	2.95	4.06	4.66	5.13
HPV16/18	Rennes	34	3.00	1.08	1.33	1.90	2.99	4.12	4.73	5.19
HPV16/18	Rennes	35	2.98	1.05	1.34	1.89	2.97	4.09	4.70	5.14
HPV16/18	Rennes	36	2.92	1.04	1.34	1.86	2.91	4.02	4.61	5.01
HPV16/18	Rennes	37	2.85	1.02	1.31	1.81	2.83	3.92	4.47	4.88

Table S10 **Posterior difference in expected HR HPV cervical infection prevalence (in percentage points) in major French cities, between opportunistic and organised screening, stratified by type of test, city, and age. Table counterpart to Figure 3B.** ***(continued)***

Virus	City	Age	Average	Posterior distribution						
				Percentiles						
				0.01	0.025	0.1	0.5	0.9	0.975	0.99
HPV16/18	Rennes	38	2.77	1.02	1.29	1.76	2.75	3.82	4.36	4.80
HPV16/18	Rennes	39	2.70	0.99	1.24	1.71	2.68	3.72	4.24	4.65
HPV16/18	Rennes	40	2.62	0.97	1.20	1.66	2.60	3.61	4.12	4.50
HPV16/18	Rennes	41	2.54	0.94	1.14	1.61	2.53	3.51	4.00	4.39
HPV16/18	Rennes	42	2.46	0.89	1.12	1.55	2.44	3.39	3.90	4.26
HPV16/18	Rennes	43	2.39	0.85	1.07	1.50	2.37	3.29	3.79	4.15
HPV16/18	Rennes	44	2.32	0.83	1.03	1.45	2.30	3.21	3.70	4.03
HPV16/18	Rennes	45	2.27	0.80	0.99	1.42	2.25	3.14	3.60	3.93
HPV16/18	Rennes	46	2.20	0.76	0.95	1.37	2.19	3.06	3.53	3.84
HPV16/18	Rennes	47	2.14	0.73	0.90	1.31	2.13	2.99	3.42	3.74
HPV16/18	Rennes	48	2.08	0.70	0.87	1.27	2.07	2.91	3.34	3.68
HPV16/18	Rennes	49	2.03	0.67	0.83	1.24	2.01	2.85	3.27	3.58
HPV16/18	Rennes	50	1.97	0.64	0.78	1.20	1.96	2.77	3.18	3.47
HPV16/18	Rennes	51	1.92	0.60	0.75	1.16	1.91	2.71	3.12	3.40
HPV16/18	Rennes	52	1.87	0.59	0.74	1.11	1.86	2.64	3.02	3.35
HPV16/18	Rennes	53	1.81	0.55	0.70	1.07	1.80	2.57	2.96	3.22
HPV16/18	Rennes	54	1.75	0.50	0.67	1.02	1.73	2.49	2.88	3.13
HPV16/18	Rennes	55	1.70	0.46	0.65	0.99	1.69	2.43	2.79	3.05
HPV16/18	Rennes	56	1.65	0.43	0.62	0.95	1.64	2.37	2.74	2.96
HPV16/18	Rennes	57	1.62	0.38	0.60	0.92	1.60	2.33	2.68	2.96
HPV16/18	Rennes	58	1.59	0.35	0.55	0.89	1.58	2.31	2.68	2.91
HPV16/18	Rennes	59	1.57	0.31	0.52	0.87	1.56	2.29	2.67	2.92
HPV16/18	Rennes	60	1.56	0.28	0.49	0.85	1.55	2.30	2.67	2.90
HPV16/18	Rennes	61	1.55	0.24	0.46	0.84	1.54	2.30	2.67	2.88
HPV16/18	Rennes	62	1.53	0.19	0.43	0.80	1.52	2.28	2.65	2.87
HPV16/18	Rennes	63	1.50	0.16	0.40	0.77	1.49	2.26	2.63	2.89
HPV16/18	Rennes	64	1.48	0.10	0.34	0.73	1.46	2.24	2.65	2.86
HPV16/18	Rennes	65	1.44	0.05	0.26	0.69	1.44	2.21	2.61	2.84
HPV16/18	Rennes	66	1.41	-0.01	0.20	0.63	1.40	2.19	2.61	2.85

Table S10 Posterior difference in expected HR HPV cervical infection prevalence (in percentage points) in major French cities, between opportunistic and organised screening, stratified by type of test, city, and age. Table counterpart to Figure 3B. ***(continued)***

			Posterior distribution							
				Percentiles						
Virus	City	Age	Average	0.01	0.025	0.1	0.5	0.9	0.975	0.99
HPV16/18	Strasbourg	30	1.43	-0.31	0.00	0.51	1.37	2.45	3.16	3.63
HPV16/18	Strasbourg	31	1.54	-0.25	0.07	0.58	1.48	2.59	3.35	3.79
HPV16/18	Strasbourg	32	1.63	-0.20	0.14	0.64	1.57	2.72	3.53	4.03
HPV16/18	Strasbourg	33	1.71	-0.15	0.19	0.69	1.64	2.83	3.67	4.22
HPV16/18	Strasbourg	34	1.75	-0.13	0.19	0.72	1.67	2.87	3.69	4.25
HPV16/18	Strasbourg	35	1.74	-0.08	0.19	0.73	1.66	2.85	3.67	4.26
HPV16/18	Strasbourg	36	1.70	-0.07	0.20	0.72	1.62	2.80	3.60	4.17
HPV16/18	Strasbourg	37	1.66	-0.06	0.20	0.70	1.58	2.72	3.49	4.09
HPV16/18	Strasbourg	38	1.61	-0.06	0.19	0.68	1.54	2.64	3.38	3.92
HPV16/18	Strasbourg	39	1.56	-0.07	0.20	0.65	1.49	2.57	3.29	3.80
HPV16/18	Strasbourg	40	1.52	-0.08	0.19	0.63	1.45	2.50	3.19	3.68
HPV16/18	Strasbourg	41	1.47	-0.08	0.17	0.61	1.41	2.42	3.10	3.59
HPV16/18	Strasbourg	42	1.42	-0.08	0.16	0.58	1.36	2.35	3.00	3.47
HPV16/18	Strasbourg	43	1.37	-0.08	0.15	0.57	1.32	2.28	2.90	3.37
HPV16/18	Strasbourg	44	1.33	-0.09	0.14	0.54	1.27	2.23	2.83	3.29
HPV16/18	Strasbourg	45	1.30	-0.10	0.12	0.51	1.24	2.17	2.78	3.22
HPV16/18	Strasbourg	46	1.26	-0.12	0.11	0.48	1.20	2.10	2.71	3.14
HPV16/18	Strasbourg	47	1.22	-0.13	0.11	0.46	1.17	2.04	2.63	3.11
HPV16/18	Strasbourg	48	1.18	-0.16	0.10	0.44	1.14	1.99	2.57	3.06
HPV16/18	Strasbourg	49	1.15	-0.17	0.08	0.42	1.10	1.93	2.50	2.98
HPV16/18	Strasbourg	50	1.11	-0.18	0.08	0.41	1.07	1.89	2.43	2.92
HPV16/18	Strasbourg	51	1.08	-0.16	0.06	0.39	1.04	1.85	2.38	2.76
HPV16/18	Strasbourg	52	1.05	-0.18	0.04	0.37	1.01	1.80	2.30	2.72
HPV16/18	Strasbourg	53	1.01	-0.18	0.02	0.34	0.97	1.74	2.23	2.65
HPV16/18	Strasbourg	54	0.97	-0.19	0.00	0.32	0.93	1.68	2.16	2.57
HPV16/18	Strasbourg	55	0.94	-0.21	-0.01	0.30	0.90	1.63	2.12	2.52
HPV16/18	Strasbourg	56	0.91	-0.23	-0.02	0.28	0.87	1.59	2.09	2.47
HPV16/18	Strasbourg	57	0.89	-0.25	-0.04	0.26	0.85	1.55	2.03	2.41
HPV16/18	Strasbourg	58	0.87	-0.26	-0.07	0.25	0.83	1.54	2.00	2.43

Table S10 Posterior difference in expected HR HPV cervical infection prevalence (in percentage points) in major French cities, between opportunistic and organised screening, stratified by type of test, city, and age. Table counterpart to Figure 3B. ***(continued)***

			Posterior distribution							
				Percentiles						
Virus	City	Age	Average	0.01	0.025	0.1	0.5	0.9	0.975	0.99
HPV16/18	Strasbourg	59	0.85	-0.27	-0.09	0.23	0.81	1.53	1.99	2.41
HPV16/18	Strasbourg	60	0.84	-0.30	-0.11	0.21	0.80	1.52	2.00	2.43
HPV16/18	Strasbourg	61	0.83	-0.34	-0.13	0.20	0.79	1.52	2.00	2.39
HPV16/18	Strasbourg	62	0.82	-0.38	-0.16	0.18	0.77	1.51	1.99	2.39
HPV16/18	Strasbourg	63	0.80	-0.45	-0.18	0.16	0.76	1.50	1.97	2.40
HPV16/18	Strasbourg	64	0.78	-0.47	-0.22	0.13	0.74	1.49	1.95	2.41
HPV16/18	Strasbourg	65	0.76	-0.50	-0.26	0.11	0.72	1.48	1.95	2.37
HPV16/18	Strasbourg	66	0.73	-0.54	-0.30	0.07	0.69	1.46	1.98	2.39
HPV16/18	Toulouse	30	1.38	-0.49	-0.18	0.35	1.36	2.41	3.04	3.45
HPV16/18	Toulouse	31	1.49	-0.44	-0.12	0.44	1.48	2.55	3.19	3.66
HPV16/18	Toulouse	32	1.59	-0.41	-0.05	0.50	1.57	2.68	3.36	3.84
HPV16/18	Toulouse	33	1.66	-0.35	0.01	0.55	1.64	2.79	3.54	3.96
HPV16/18	Toulouse	34	1.70	-0.31	0.04	0.60	1.67	2.84	3.59	4.11
HPV16/18	Toulouse	35	1.69	-0.27	0.05	0.61	1.65	2.83	3.55	4.09
HPV16/18	Toulouse	36	1.66	-0.28	0.02	0.62	1.61	2.77	3.50	4.01
HPV16/18	Toulouse	37	1.62	-0.27	0.05	0.61	1.57	2.70	3.37	3.87
HPV16/18	Toulouse	38	1.57	-0.26	0.07	0.59	1.53	2.63	3.28	3.75
HPV16/18	Toulouse	39	1.53	-0.25	0.07	0.56	1.48	2.57	3.21	3.67
HPV16/18	Toulouse	40	1.48	-0.24	0.05	0.54	1.43	2.50	3.13	3.59
HPV16/18	Toulouse	41	1.43	-0.25	0.02	0.53	1.38	2.43	3.04	3.46
HPV16/18	Toulouse	42	1.38	-0.25	0.02	0.50	1.33	2.35	2.94	3.34
HPV16/18	Toulouse	43	1.33	-0.25	0.01	0.48	1.28	2.27	2.86	3.21
HPV16/18	Toulouse	44	1.29	-0.26	0.00	0.47	1.25	2.20	2.79	3.12
HPV16/18	Toulouse	45	1.26	-0.26	-0.01	0.44	1.21	2.15	2.73	3.04
HPV16/18	Toulouse	46	1.22	-0.27	-0.03	0.42	1.17	2.09	2.64	2.97
HPV16/18	Toulouse	47	1.18	-0.27	-0.02	0.40	1.14	2.03	2.57	2.88
HPV16/18	Toulouse	48	1.14	-0.26	-0.04	0.38	1.10	1.98	2.52	2.80
HPV16/18	Toulouse	49	1.11	-0.29	-0.07	0.36	1.07	1.93	2.47	2.71
HPV16/18	Toulouse	50	1.07	-0.28	-0.08	0.33	1.03	1.87	2.41	2.64

Table S10 **Posterior difference in expected HR HPV cervical infection prevalence (in percentage points) in major French cities, between opportunistic and organised screening, stratified by type of test, city, and age. Table counterpart to Figure 3B.** ***(continued)***

			Posterior distribution							
				Percentiles						
Virus	City	Age	Average	0.01	0.025	0.1	0.5	0.9	0.975	0.99
HPV16/18	Toulouse	51	1.04	-0.30	-0.09	0.31	1.00	1.81	2.35	2.59
HPV16/18	Toulouse	52	1.01	-0.31	-0.10	0.29	0.97	1.77	2.30	2.54
HPV16/18	Toulouse	53	0.97	-0.33	-0.11	0.27	0.93	1.71	2.24	2.47
HPV16/18	Toulouse	54	0.93	-0.35	-0.13	0.25	0.90	1.65	2.17	2.39
HPV16/18	Toulouse	55	0.89	-0.37	-0.15	0.23	0.86	1.60	2.11	2.35
HPV16/18	Toulouse	56	0.86	-0.38	-0.16	0.21	0.84	1.56	2.05	2.29
HPV16/18	Toulouse	57	0.84	-0.41	-0.18	0.19	0.81	1.52	2.02	2.29
HPV16/18	Toulouse	58	0.82	-0.43	-0.20	0.16	0.79	1.50	1.99	2.27
HPV16/18	Toulouse	59	0.80	-0.45	-0.22	0.14	0.78	1.50	1.97	2.26
HPV16/18	Toulouse	60	0.79	-0.46	-0.24	0.12	0.77	1.49	1.95	2.26
HPV16/18	Toulouse	61	0.77	-0.49	-0.26	0.10	0.76	1.50	1.96	2.25
HPV16/18	Toulouse	62	0.76	-0.52	-0.29	0.08	0.74	1.48	1.95	2.24
HPV16/18	Toulouse	63	0.74	-0.56	-0.32	0.05	0.72	1.46	1.92	2.21
HPV16/18	Toulouse	64	0.72	-0.59	-0.36	0.02	0.70	1.45	1.94	2.19
HPV16/18	Toulouse	65	0.69	-0.65	-0.41	-0.02	0.68	1.42	1.91	2.18
HPV16/18	Toulouse	66	0.67	-0.74	-0.48	-0.06	0.66	1.41	1.90	2.17
Other genotypes	Bordeaux	30	7.69	3.00	3.81	5.20	7.68	10.20	11.47	12.23
Other genotypes	Bordeaux	31	7.37	2.93	3.72	5.05	7.37	9.76	10.94	11.66
Other genotypes	Bordeaux	32	7.01	2.86	3.47	4.79	7.02	9.30	10.41	11.06
Other genotypes	Bordeaux	33	6.62	2.65	3.24	4.52	6.63	8.81	9.86	10.48
Other genotypes	Bordeaux	34	6.28	2.53	3.05	4.26	6.28	8.37	9.37	9.97
Other genotypes	Bordeaux	35	5.98	2.35	2.94	4.02	5.97	7.97	8.99	9.58
Other genotypes	Bordeaux	36	5.70	2.21	2.79	3.81	5.69	7.62	8.67	9.19
Other genotypes	Bordeaux	37	5.47	2.06	2.64	3.64	5.44	7.33	8.34	8.91
Other genotypes	Bordeaux	38	5.25	1.92	2.50	3.46	5.22	7.04	8.02	8.64
Other genotypes	Bordeaux	39	5.03	1.80	2.34	3.29	5.02	6.79	7.76	8.37
Other genotypes	Bordeaux	40	4.81	1.61	2.21	3.12	4.80	6.51	7.49	8.01
Other genotypes	Bordeaux	41	4.61	1.53	2.07	2.97	4.62	6.26	7.21	7.76
Other genotypes	Bordeaux	42	4.44	1.43	1.95	2.81	4.45	6.05	6.99	7.54

Table S10 **Posterior difference in expected HR HPV cervical infection prevalence (in percentage points) in major French cities, between opportunistic and organised screening, stratified by type of test, city, and age. Table counterpart to Figure 3B.** ***(continued)***

			Posterior distribution							
				Percentiles						
Virus	City	Age	Average	0.01	0.025	0.1	0.5	0.9	0.975	0.99
Other genotypes	Bordeaux	43	4.29	1.31	1.85	2.71	4.31	5.88	6.79	7.35
Other genotypes	Bordeaux	44	4.15	1.23	1.74	2.59	4.15	5.71	6.58	7.16
Other genotypes	Bordeaux	45	4.02	1.13	1.65	2.49	4.03	5.57	6.40	6.99
Other genotypes	Bordeaux	46	3.91	1.03	1.54	2.38	3.91	5.44	6.24	6.87
Other genotypes	Bordeaux	47	3.81	0.96	1.45	2.31	3.80	5.31	6.14	6.74
Other genotypes	Bordeaux	48	3.71	0.89	1.37	2.21	3.70	5.20	6.09	6.61
Other genotypes	Bordeaux	49	3.62	0.81	1.28	2.13	3.62	5.10	5.96	6.58
Other genotypes	Bordeaux	50	3.53	0.72	1.19	2.05	3.53	4.99	5.86	6.43
Other genotypes	Bordeaux	51	3.43	0.66	1.11	1.97	3.44	4.89	5.69	6.27
Other genotypes	Bordeaux	52	3.31	0.57	1.02	1.86	3.32	4.75	5.61	6.09
Other genotypes	Bordeaux	53	3.17	0.49	0.93	1.73	3.19	4.57	5.39	5.90
Other genotypes	Bordeaux	54	3.02	0.43	0.80	1.60	3.04	4.38	5.21	5.70
Other genotypes	Bordeaux	55	2.87	0.34	0.68	1.48	2.89	4.21	4.99	5.48
Other genotypes	Bordeaux	56	2.72	0.23	0.58	1.36	2.73	4.03	4.81	5.28
Other genotypes	Bordeaux	57	2.57	0.08	0.48	1.25	2.60	3.87	4.61	5.10
Other genotypes	Bordeaux	58	2.44	0.04	0.38	1.13	2.46	3.74	4.42	4.92
Other genotypes	Bordeaux	59	2.33	-0.05	0.26	1.02	2.34	3.62	4.27	4.73
Other genotypes	Bordeaux	60	2.23	-0.18	0.17	0.92	2.25	3.52	4.18	4.58
Other genotypes	Bordeaux	61	2.16	-0.29	0.06	0.83	2.18	3.45	4.13	4.47
Other genotypes	Bordeaux	62	2.09	-0.37	0.00	0.75	2.11	3.41	4.10	4.45
Other genotypes	Bordeaux	63	2.05	-0.48	-0.06	0.68	2.06	3.39	4.08	4.45
Other genotypes	Bordeaux	64	2.02	-0.58	-0.15	0.62	2.02	3.40	4.10	4.51
Other genotypes	Bordeaux	65	2.00	-0.70	-0.28	0.55	2.00	3.41	4.16	4.55
Other genotypes	Bordeaux	66	1.98	-0.87	-0.39	0.47	1.99	3.47	4.25	4.68
Other genotypes	Lille	30	7.89	3.01	3.86	5.07	7.80	10.81	12.58	13.59
Other genotypes	Lille	31	7.56	2.96	3.75	4.88	7.46	10.35	12.08	13.10
Other genotypes	Lille	32	7.18	2.88	3.57	4.64	7.07	9.90	11.52	12.51
Other genotypes	Lille	33	6.79	2.80	3.35	4.35	6.69	9.35	10.98	11.96
Other genotypes	Lille	34	6.43	2.64	3.13	4.09	6.34	8.91	10.47	11.40

Table S10 **Posterior difference in expected HR HPV cervical infection prevalence (in percentage points) in major French cities, between opportunistic and organised screening, stratified by type of test, city, and age. Table counterpart to Figure 3B.** ***(continued)***

			Posterior distribution							
				Percentiles						
Virus	City	Age	Average	0.01	0.025	0.1	0.5	0.9	0.975	0.99
Other genotypes	Lille	35	6.13	2.48	2.95	3.90	6.03	8.52	10.06	11.01
Other genotypes	Lille	36	5.85	2.34	2.77	3.70	5.76	8.13	9.61	10.50
Other genotypes	Lille	37	5.61	2.20	2.64	3.54	5.52	7.83	9.27	10.11
Other genotypes	Lille	38	5.39	2.07	2.50	3.36	5.29	7.53	8.97	9.70
Other genotypes	Lille	39	5.17	1.95	2.39	3.22	5.08	7.29	8.65	9.43
Other genotypes	Lille	40	4.95	1.78	2.21	3.07	4.86	7.02	8.28	9.09
Other genotypes	Lille	41	4.75	1.68	2.08	2.92	4.67	6.76	8.01	8.84
Other genotypes	Lille	42	4.58	1.60	1.96	2.79	4.50	6.53	7.74	8.52
Other genotypes	Lille	43	4.43	1.50	1.88	2.68	4.34	6.33	7.54	8.32
Other genotypes	Lille	44	4.29	1.42	1.80	2.56	4.20	6.15	7.36	8.08
Other genotypes	Lille	45	4.16	1.32	1.71	2.47	4.08	6.01	7.20	7.89
Other genotypes	Lille	46	4.05	1.24	1.62	2.38	3.97	5.84	7.00	7.81
Other genotypes	Lille	47	3.95	1.19	1.52	2.29	3.87	5.71	6.85	7.68
Other genotypes	Lille	48	3.85	1.12	1.44	2.22	3.78	5.61	6.76	7.52
Other genotypes	Lille	49	3.76	1.05	1.35	2.15	3.69	5.51	6.70	7.33
Other genotypes	Lille	50	3.67	0.93	1.30	2.07	3.59	5.41	6.55	7.21
Other genotypes	Lille	51	3.58	0.85	1.21	1.97	3.51	5.29	6.44	7.09
Other genotypes	Lille	52	3.46	0.75	1.10	1.87	3.39	5.17	6.32	6.88
Other genotypes	Lille	53	3.33	0.65	1.02	1.78	3.26	5.00	6.16	6.69
Other genotypes	Lille	54	3.17	0.52	0.90	1.65	3.11	4.80	5.89	6.42
Other genotypes	Lille	55	3.02	0.37	0.81	1.53	2.96	4.61	5.69	6.26
Other genotypes	Lille	56	2.88	0.25	0.70	1.40	2.83	4.40	5.48	5.95
Other genotypes	Lille	57	2.73	0.15	0.58	1.29	2.69	4.23	5.25	5.70
Other genotypes	Lille	58	2.60	0.07	0.49	1.17	2.56	4.08	5.11	5.55
Other genotypes	Lille	59	2.49	-0.01	0.39	1.07	2.45	3.95	4.97	5.41
Other genotypes	Lille	60	2.40	-0.07	0.31	0.99	2.36	3.85	4.82	5.33
Other genotypes	Lille	61	2.32	-0.13	0.25	0.92	2.29	3.79	4.75	5.23
Other genotypes	Lille	62	2.27	-0.21	0.16	0.86	2.23	3.75	4.72	5.17
Other genotypes	Lille	63	2.23	-0.29	0.09	0.81	2.18	3.73	4.69	5.16

Table S10 Posterior difference in expected HR HPV cervical infection prevalence (in percentage points) in major French cities, between opportunistic and organised screening, stratified by type of test, city, and age. Table counterpart to Figure 3B. ***(continued)***

			Posterior distribution							
				Percentiles						
Virus	City	Age	Average	0.01	0.025	0.1	0.5	0.9	0.975	0.99
Other genotypes	Lille	64	2.20	-0.40	0.01	0.73	2.15	3.75	4.71	5.19
Other genotypes	Lille	65	2.19	-0.58	-0.08	0.66	2.13	3.78	4.79	5.31
Other genotypes	Lille	66	2.18	-0.70	-0.17	0.60	2.11	3.84	4.87	5.49
Other genotypes	Lyon	30	8.19	2.46	3.49	5.07	8.08	11.43	13.41	14.54
Other genotypes	Lyon	31	7.87	2.49	3.44	4.86	7.74	10.98	12.94	13.95
Other genotypes	Lyon	32	7.50	2.35	3.22	4.62	7.37	10.50	12.36	13.43
Other genotypes	Lyon	33	7.10	2.19	2.98	4.38	6.95	9.99	11.71	12.79
Other genotypes	Lyon	34	6.74	2.05	2.79	4.11	6.62	9.49	11.19	12.21
Other genotypes	Lyon	35	6.44	1.89	2.65	3.89	6.31	9.08	10.71	11.76
Other genotypes	Lyon	36	6.15	1.75	2.49	3.70	6.01	8.74	10.37	11.36
Other genotypes	Lyon	37	5.90	1.68	2.37	3.53	5.77	8.42	10.08	10.97
Other genotypes	Lyon	38	5.67	1.60	2.25	3.37	5.53	8.10	9.67	10.64
Other genotypes	Lyon	39	5.44	1.47	2.10	3.21	5.32	7.81	9.37	10.28
Other genotypes	Lyon	40	5.21	1.36	1.98	3.03	5.09	7.54	9.02	9.90
Other genotypes	Lyon	41	5.00	1.32	1.80	2.89	4.88	7.26	8.70	9.67
Other genotypes	Lyon	42	4.82	1.12	1.69	2.74	4.69	7.02	8.45	9.38
Other genotypes	Lyon	43	4.66	1.02	1.59	2.64	4.54	6.82	8.24	9.22
Other genotypes	Lyon	44	4.51	0.97	1.51	2.53	4.39	6.63	8.02	9.00
Other genotypes	Lyon	45	4.37	0.84	1.40	2.43	4.26	6.46	7.84	8.78
Other genotypes	Lyon	46	4.25	0.79	1.32	2.34	4.14	6.30	7.68	8.63
Other genotypes	Lyon	47	4.14	0.72	1.23	2.23	4.03	6.17	7.53	8.46
Other genotypes	Lyon	48	4.04	0.63	1.18	2.17	3.93	6.06	7.39	8.33
Other genotypes	Lyon	49	3.94	0.51	1.10	2.07	3.84	5.90	7.28	8.17
Other genotypes	Lyon	50	3.84	0.39	1.04	1.98	3.75	5.78	7.11	8.03
Other genotypes	Lyon	51	3.73	0.34	0.95	1.90	3.63	5.66	6.95	7.91
Other genotypes	Lyon	52	3.60	0.25	0.86	1.80	3.50	5.49	6.77	7.69
Other genotypes	Lyon	53	3.46	0.16	0.71	1.69	3.37	5.34	6.58	7.39
Other genotypes	Lyon	54	3.29	0.08	0.59	1.54	3.19	5.15	6.39	7.18
Other genotypes	Lyon	55	3.13	0.01	0.49	1.41	3.04	4.93	6.17	6.86

Table S10 **Posterior difference in expected HR HPV cervical infection prevalence (in percentage points) in major French cities, between opportunistic and organised screening, stratified by type of test, city, and age. Table counterpart to Figure 3B.** ***(continued)***

			Posterior distribution							
				Percentiles						
Virus	City	Age	Average	0.01	0.025	0.1	0.5	0.9	0.975	0.99
Other genotypes	Lyon	56	2.98	-0.09	0.37	1.28	2.90	4.74	5.95	6.55
Other genotypes	Lyon	57	2.82	-0.18	0.24	1.17	2.74	4.57	5.69	6.28
Other genotypes	Lyon	58	2.67	-0.28	0.12	1.05	2.61	4.37	5.52	6.09
Other genotypes	Lyon	59	2.55	-0.37	0.03	0.93	2.49	4.21	5.31	6.06
Other genotypes	Lyon	60	2.45	-0.45	-0.03	0.84	2.39	4.10	5.15	5.89
Other genotypes	Lyon	61	2.36	-0.54	-0.09	0.78	2.31	4.02	5.03	5.77
Other genotypes	Lyon	62	2.30	-0.65	-0.17	0.69	2.26	3.97	4.99	5.67
Other genotypes	Lyon	63	2.25	-0.73	-0.28	0.60	2.20	3.94	5.03	5.65
Other genotypes	Lyon	64	2.22	-0.85	-0.37	0.54	2.16	3.93	5.03	5.65
Other genotypes	Lyon	65	2.19	-1.01	-0.48	0.47	2.15	3.92	5.08	5.79
Other genotypes	Lyon	66	2.18	-1.21	-0.62	0.40	2.15	3.98	5.16	5.90
Other genotypes	Marseille	30	5.92	0.49	1.51	3.14	6.01	8.61	9.88	10.50
Other genotypes	Marseille	31	5.67	0.51	1.47	3.01	5.75	8.21	9.33	10.02
Other genotypes	Marseille	32	5.38	0.54	1.35	2.84	5.44	7.79	8.88	9.54
Other genotypes	Marseille	33	5.07	0.53	1.18	2.66	5.12	7.35	8.36	9.03
Other genotypes	Marseille	34	4.79	0.46	1.08	2.47	4.86	6.96	7.86	8.56
Other genotypes	Marseille	35	4.54	0.34	0.98	2.30	4.63	6.63	7.56	8.17
Other genotypes	Marseille	36	4.31	0.21	0.87	2.13	4.41	6.35	7.23	7.83
Other genotypes	Marseille	37	4.11	0.14	0.78	2.02	4.19	6.09	6.93	7.53
Other genotypes	Marseille	38	3.93	-0.01	0.65	1.88	4.02	5.85	6.66	7.26
Other genotypes	Marseille	39	3.75	-0.08	0.54	1.75	3.82	5.62	6.44	6.92
Other genotypes	Marseille	40	3.56	-0.13	0.45	1.63	3.63	5.38	6.18	6.63
Other genotypes	Marseille	41	3.39	-0.22	0.36	1.52	3.47	5.17	5.95	6.39
Other genotypes	Marseille	42	3.25	-0.32	0.25	1.42	3.31	5.00	5.77	6.22
Other genotypes	Marseille	43	3.11	-0.41	0.16	1.33	3.17	4.83	5.59	6.03
Other genotypes	Marseille	44	2.99	-0.49	0.04	1.21	3.07	4.68	5.42	5.90
Other genotypes	Marseille	45	2.88	-0.58	-0.03	1.11	2.96	4.56	5.30	5.69
Other genotypes	Marseille	46	2.78	-0.60	-0.11	1.00	2.84	4.44	5.20	5.58
Other genotypes	Marseille	47	2.68	-0.69	-0.21	0.92	2.75	4.34	5.10	5.48

Table S10 Posterior difference in expected HR HPV cervical infection prevalence (in percentage points) in major French cities, between opportunistic and organised screening, stratified by type of test, city, and age. Table counterpart to Figure 3B. ***(continued)***

			Posterior distribution							
				Percentiles						
Virus	City	Age	Average	0.01	0.025	0.1	0.5	0.9	0.975	0.99
Other genotypes	Marseille	48	2.59	-0.79	-0.31	0.85	2.65	4.23	5.00	5.34
Other genotypes	Marseille	49	2.50	-0.86	-0.39	0.76	2.58	4.13	4.90	5.27
Other genotypes	Marseille	50	2.41	-0.97	-0.43	0.68	2.49	4.03	4.80	5.17
Other genotypes	Marseille	51	2.32	-1.07	-0.49	0.61	2.39	3.92	4.72	5.06
Other genotypes	Marseille	52	2.21	-1.14	-0.56	0.52	2.29	3.80	4.55	4.90
Other genotypes	Marseille	53	2.09	-1.22	-0.61	0.40	2.17	3.66	4.40	4.77
Other genotypes	Marseille	54	1.95	-1.30	-0.69	0.30	2.02	3.49	4.25	4.63
Other genotypes	Marseille	55	1.81	-1.41	-0.76	0.18	1.88	3.31	4.05	4.46
Other genotypes	Marseille	56	1.68	-1.45	-0.88	0.08	1.75	3.16	3.90	4.28
Other genotypes	Marseille	57	1.55	-1.50	-1.00	-0.04	1.62	3.01	3.73	4.10
Other genotypes	Marseille	58	1.43	-1.59	-1.08	-0.15	1.50	2.88	3.56	3.94
Other genotypes	Marseille	59	1.32	-1.66	-1.18	-0.24	1.40	2.76	3.41	3.76
Other genotypes	Marseille	60	1.23	-1.78	-1.26	-0.31	1.31	2.68	3.30	3.65
Other genotypes	Marseille	61	1.16	-1.86	-1.33	-0.40	1.23	2.61	3.22	3.65
Other genotypes	Marseille	62	1.09	-1.96	-1.45	-0.49	1.18	2.56	3.20	3.59
Other genotypes	Marseille	63	1.04	-2.12	-1.57	-0.56	1.12	2.54	3.21	3.59
Other genotypes	Marseille	64	0.99	-2.24	-1.70	-0.64	1.07	2.53	3.22	3.61
Other genotypes	Marseille	65	0.95	-2.41	-1.86	-0.74	1.04	2.55	3.29	3.66
Other genotypes	Marseille	66	0.91	-2.68	-2.05	-0.85	1.02	2.57	3.36	3.83
Other genotypes	Montpellier	30	7.76	2.45	3.25	4.88	7.72	10.70	12.56	13.97
Other genotypes	Montpellier	31	7.45	2.45	3.16	4.68	7.42	10.28	12.02	13.44
Other genotypes	Montpellier	32	7.10	2.35	3.00	4.45	7.02	9.84	11.44	12.81
Other genotypes	Montpellier	33	6.71	2.22	2.81	4.17	6.64	9.35	10.86	12.21
Other genotypes	Montpellier	34	6.37	1.95	2.64	3.91	6.29	8.91	10.39	11.65
Other genotypes	Montpellier	35	6.07	1.81	2.52	3.71	6.00	8.49	10.03	11.02
Other genotypes	Montpellier	36	5.80	1.66	2.34	3.53	5.72	8.15	9.59	10.59
Other genotypes	Montpellier	37	5.56	1.52	2.18	3.36	5.48	7.84	9.26	10.22
Other genotypes	Montpellier	38	5.33	1.39	2.06	3.18	5.26	7.57	8.87	9.92
Other genotypes	Montpellier	39	5.12	1.30	1.96	3.03	5.05	7.32	8.54	9.52

Table S10 Posterior difference in expected HR HPV cervical infection prevalence (in percentage points) in major French cities, between opportunistic and organised screening, stratified by type of test, city, and age. Table counterpart to Figure 3B. ***(continued)***

			Posterior distribution							
				Percentiles						
Virus	City	Age	Average	0.01	0.025	0.1	0.5	0.9	0.975	0.99
Other genotypes	Montpellier	40	4.89	1.16	1.80	2.86	4.84	7.02	8.33	9.16
Other genotypes	Montpellier	41	4.69	1.05	1.68	2.72	4.65	6.77	8.04	8.85
Other genotypes	Montpellier	42	4.52	0.96	1.54	2.59	4.48	6.57	7.80	8.56
Other genotypes	Montpellier	43	4.36	0.81	1.46	2.50	4.31	6.38	7.58	8.30
Other genotypes	Montpellier	44	4.22	0.75	1.35	2.38	4.18	6.19	7.41	8.11
Other genotypes	Montpellier	45	4.09	0.65	1.26	2.28	4.05	6.02	7.22	7.94
Other genotypes	Montpellier	46	3.97	0.53	1.17	2.19	3.94	5.90	7.03	7.78
Other genotypes	Montpellier	47	3.86	0.46	1.09	2.12	3.82	5.74	6.90	7.72
Other genotypes	Montpellier	48	3.76	0.38	1.01	2.02	3.72	5.62	6.78	7.54
Other genotypes	Montpellier	49	3.67	0.34	0.94	1.94	3.62	5.52	6.65	7.33
Other genotypes	Montpellier	50	3.57	0.29	0.86	1.84	3.53	5.40	6.50	7.20
Other genotypes	Montpellier	51	3.47	0.17	0.77	1.73	3.41	5.30	6.32	7.06
Other genotypes	Montpellier	52	3.34	0.09	0.69	1.63	3.29	5.14	6.16	6.95
Other genotypes	Montpellier	53	3.20	0.02	0.58	1.52	3.16	4.99	6.03	6.74
Other genotypes	Montpellier	54	3.04	-0.05	0.46	1.37	3.00	4.79	5.84	6.53
Other genotypes	Montpellier	55	2.89	-0.17	0.34	1.26	2.85	4.59	5.64	6.30
Other genotypes	Montpellier	56	2.74	-0.28	0.24	1.14	2.70	4.40	5.43	6.11
Other genotypes	Montpellier	57	2.58	-0.35	0.14	1.02	2.54	4.21	5.19	5.83
Other genotypes	Montpellier	58	2.45	-0.43	0.02	0.92	2.41	4.04	5.00	5.65
Other genotypes	Montpellier	59	2.32	-0.50	-0.04	0.81	2.29	3.91	4.90	5.48
Other genotypes	Montpellier	60	2.23	-0.60	-0.10	0.72	2.19	3.79	4.77	5.30
Other genotypes	Montpellier	61	2.14	-0.67	-0.20	0.63	2.11	3.69	4.74	5.19
Other genotypes	Montpellier	62	2.08	-0.75	-0.29	0.57	2.06	3.62	4.65	5.20
Other genotypes	Montpellier	63	2.03	-0.85	-0.41	0.50	2.02	3.59	4.59	5.16
Other genotypes	Montpellier	64	1.99	-0.96	-0.50	0.41	1.98	3.59	4.57	5.18
Other genotypes	Montpellier	65	1.97	-1.12	-0.57	0.32	1.96	3.61	4.64	5.31
Other genotypes	Montpellier	66	1.95	-1.35	-0.71	0.22	1.93	3.68	4.73	5.39
Other genotypes	Nantes	30	7.93	3.25	3.86	5.21	7.71	11.02	13.12	14.11
Other genotypes	Nantes	31	7.59	3.09	3.78	5.00	7.38	10.53	12.56	13.54

Table S10 Posterior difference in expected HR HPV cervical infection prevalence (in percentage points) in major French cities, between opportunistic and organised screening, stratified by type of test, city, and age. Table counterpart to Figure 3B. ***(continued)***

			Posterior distribution							
				Percentiles						
Virus	City	Age	Average	0.01	0.025	0.1	0.5	0.9	0.975	0.99
Other genotypes	Nantes	32	7.21	3.02	3.61	4.75	7.00	10.02	12.02	13.01
Other genotypes	Nantes	33	6.81	2.76	3.40	4.47	6.59	9.49	11.36	12.37
Other genotypes	Nantes	34	6.45	2.55	3.23	4.21	6.25	9.02	10.74	11.74
Other genotypes	Nantes	35	6.15	2.41	3.06	3.98	5.97	8.62	10.29	11.19
Other genotypes	Nantes	36	5.87	2.20	2.91	3.76	5.70	8.21	9.87	10.73
Other genotypes	Nantes	37	5.63	2.02	2.79	3.60	5.46	7.92	9.55	10.36
Other genotypes	Nantes	38	5.41	1.89	2.61	3.42	5.24	7.62	9.22	10.02
Other genotypes	Nantes	39	5.19	1.73	2.46	3.28	5.03	7.31	8.87	9.62
Other genotypes	Nantes	40	4.97	1.63	2.36	3.11	4.81	7.03	8.60	9.27
Other genotypes	Nantes	41	4.77	1.54	2.21	2.96	4.61	6.75	8.28	8.96
Other genotypes	Nantes	42	4.60	1.46	2.10	2.85	4.45	6.54	8.04	8.66
Other genotypes	Nantes	43	4.45	1.38	1.97	2.73	4.29	6.34	7.79	8.50
Other genotypes	Nantes	44	4.32	1.29	1.90	2.64	4.15	6.18	7.55	8.30
Other genotypes	Nantes	45	4.19	1.27	1.79	2.53	4.03	6.03	7.37	8.06
Other genotypes	Nantes	46	4.08	1.23	1.69	2.45	3.94	5.91	7.22	7.97
Other genotypes	Nantes	47	3.98	1.16	1.63	2.35	3.85	5.80	7.12	7.79
Other genotypes	Nantes	48	3.89	1.09	1.57	2.27	3.76	5.67	6.96	7.63
Other genotypes	Nantes	49	3.81	1.09	1.49	2.19	3.68	5.56	6.82	7.57
Other genotypes	Nantes	50	3.72	1.05	1.42	2.11	3.59	5.47	6.69	7.38
Other genotypes	Nantes	51	3.62	0.93	1.35	2.04	3.50	5.36	6.59	7.24
Other genotypes	Nantes	52	3.51	0.89	1.29	1.96	3.38	5.21	6.43	7.06
Other genotypes	Nantes	53	3.38	0.83	1.19	1.87	3.25	5.06	6.28	6.88
Other genotypes	Nantes	54	3.23	0.72	1.10	1.75	3.11	4.87	6.09	6.67
Other genotypes	Nantes	55	3.09	0.63	1.02	1.64	2.97	4.68	5.85	6.41
Other genotypes	Nantes	56	2.95	0.52	0.92	1.53	2.82	4.51	5.64	6.17
Other genotypes	Nantes	57	2.80	0.41	0.81	1.44	2.68	4.32	5.44	5.96
Other genotypes	Nantes	58	2.68	0.32	0.74	1.34	2.56	4.17	5.27	5.75
Other genotypes	Nantes	59	2.57	0.24	0.64	1.24	2.45	4.03	5.12	5.57
Other genotypes	Nantes	60	2.48	0.18	0.57	1.15	2.37	3.93	4.96	5.48

Table S10 Posterior difference in expected HR HPV cervical infection prevalence (in percentage points) in major French cities, between opportunistic and organised screening, stratified by type of test, city, and age. Table counterpart to Figure 3B. ***(continued)***

			Posterior distribution							
				Percentiles						
Virus	City	Age	Average	0.01	0.025	0.1	0.5	0.9	0.975	0.99
Other genotypes	Nantes	61	2.41	0.04	0.52	1.08	2.30	3.86	4.88	5.37
Other genotypes	Nantes	62	2.35	-0.02	0.44	1.02	2.25	3.80	4.81	5.34
Other genotypes	Nantes	63	2.32	-0.17	0.37	0.96	2.22	3.79	4.81	5.35
Other genotypes	Nantes	64	2.30	-0.29	0.28	0.89	2.20	3.80	4.87	5.35
Other genotypes	Nantes	65	2.29	-0.37	0.17	0.84	2.19	3.85	4.93	5.46
Other genotypes	Nantes	66	2.29	-0.49	0.06	0.78	2.19	3.93	4.99	5.54
Other genotypes	Nice	30	6.22	0.26	1.39	3.00	6.11	9.61	11.72	13.06
Other genotypes	Nice	31	5.95	0.34	1.30	2.86	5.85	9.20	11.28	12.54
Other genotypes	Nice	32	5.65	0.30	1.18	2.72	5.55	8.77	10.74	11.89
Other genotypes	Nice	33	5.32	0.28	1.07	2.49	5.23	8.29	10.20	11.32
Other genotypes	Nice	34	5.04	0.24	0.91	2.35	4.95	7.87	9.66	10.80
Other genotypes	Nice	35	4.79	0.16	0.79	2.22	4.70	7.50	9.21	10.46
Other genotypes	Nice	36	4.55	0.08	0.73	2.07	4.45	7.19	8.89	10.04
Other genotypes	Nice	37	4.36	-0.01	0.66	1.94	4.26	6.91	8.59	9.81
Other genotypes	Nice	38	4.17	-0.09	0.59	1.83	4.06	6.67	8.29	9.48
Other genotypes	Nice	39	3.99	-0.14	0.48	1.71	3.89	6.42	8.02	9.15
Other genotypes	Nice	40	3.80	-0.20	0.35	1.60	3.70	6.16	7.69	8.79
Other genotypes	Nice	41	3.63	-0.28	0.27	1.48	3.53	5.92	7.46	8.48
Other genotypes	Nice	42	3.49	-0.34	0.24	1.40	3.37	5.74	7.21	8.22
Other genotypes	Nice	43	3.36	-0.45	0.20	1.30	3.26	5.57	7.01	7.93
Other genotypes	Nice	44	3.24	-0.53	0.15	1.21	3.15	5.40	6.83	7.70
Other genotypes	Nice	45	3.13	-0.60	0.09	1.13	3.03	5.27	6.66	7.51
Other genotypes	Nice	46	3.04	-0.66	0.02	1.05	2.95	5.14	6.55	7.35
Other genotypes	Nice	47	2.94	-0.74	-0.03	1.00	2.86	5.02	6.43	7.24
Other genotypes	Nice	48	2.86	-0.80	-0.09	0.93	2.78	4.91	6.30	7.09
Other genotypes	Nice	49	2.78	-0.86	-0.15	0.86	2.70	4.78	6.16	6.95
Other genotypes	Nice	50	2.70	-0.94	-0.21	0.79	2.63	4.67	6.08	6.81
Other genotypes	Nice	51	2.61	-0.97	-0.28	0.71	2.53	4.54	5.92	6.65
Other genotypes	Nice	52	2.50	-1.08	-0.35	0.63	2.43	4.43	5.73	6.48

Table S10 **Posterior difference in expected HR HPV cervical infection prevalence (in percentage points) in major French cities, between opportunistic and organised screening, stratified by type of test, city, and age. Table counterpart to Figure 3B.** ***(continued)***

			Posterior distribution							
				Percentiles						
Virus	City	Age	Average	0.01	0.025	0.1	0.5	0.9	0.975	0.99
Other genotypes	Nice	53	2.39	-1.13	-0.43	0.54	2.32	4.28	5.56	6.33
Other genotypes	Nice	54	2.25	-1.13	-0.51	0.46	2.19	4.12	5.33	6.14
Other genotypes	Nice	55	2.12	-1.18	-0.58	0.39	2.05	3.94	5.13	5.98
Other genotypes	Nice	56	2.00	-1.27	-0.65	0.29	1.92	3.78	4.91	5.75
Other genotypes	Nice	57	1.87	-1.34	-0.72	0.19	1.80	3.61	4.76	5.54
Other genotypes	Nice	58	1.75	-1.40	-0.81	0.12	1.69	3.46	4.54	5.38
Other genotypes	Nice	59	1.65	-1.45	-0.85	0.04	1.60	3.33	4.42	5.20
Other genotypes	Nice	60	1.57	-1.49	-0.94	-0.02	1.52	3.22	4.30	5.09
Other genotypes	Nice	61	1.50	-1.61	-1.02	-0.09	1.47	3.16	4.20	4.97
Other genotypes	Nice	62	1.44	-1.70	-1.09	-0.15	1.41	3.10	4.13	4.95
Other genotypes	Nice	63	1.40	-1.78	-1.19	-0.24	1.37	3.09	4.12	4.92
Other genotypes	Nice	64	1.36	-1.83	-1.28	-0.31	1.34	3.08	4.13	5.02
Other genotypes	Nice	65	1.34	-1.95	-1.43	-0.38	1.33	3.10	4.12	5.02
Other genotypes	Nice	66	1.31	-2.11	-1.56	-0.46	1.30	3.12	4.18	5.06
Other genotypes	Paris	30	9.40	4.31	5.10	6.50	9.45	12.23	13.75	14.50
Other genotypes	Paris	31	9.05	4.16	5.01	6.30	9.08	11.72	13.20	13.80
Other genotypes	Paris	32	8.64	4.06	4.79	6.06	8.67	11.16	12.61	13.18
Other genotypes	Paris	33	8.21	3.92	4.49	5.76	8.19	10.61	11.97	12.51
Other genotypes	Paris	34	7.82	3.68	4.21	5.46	7.78	10.13	11.42	12.02
Other genotypes	Paris	35	7.48	3.53	4.00	5.22	7.46	9.72	10.94	11.52
Other genotypes	Paris	36	7.15	3.32	3.79	5.00	7.13	9.32	10.52	11.16
Other genotypes	Paris	37	6.88	3.21	3.62	4.78	6.86	9.00	10.12	10.76
Other genotypes	Paris	38	6.62	3.04	3.50	4.57	6.60	8.65	9.75	10.31
Other genotypes	Paris	39	6.36	2.89	3.35	4.38	6.36	8.34	9.39	10.01
Other genotypes	Paris	40	6.10	2.69	3.17	4.15	6.09	8.03	9.06	9.70
Other genotypes	Paris	41	5.87	2.51	3.01	3.97	5.85	7.75	8.77	9.39
Other genotypes	Paris	42	5.66	2.38	2.87	3.80	5.64	7.50	8.47	9.15
Other genotypes	Paris	43	5.48	2.27	2.75	3.66	5.46	7.29	8.16	8.95
Other genotypes	Paris	44	5.32	2.13	2.59	3.52	5.31	7.10	8.00	8.70

Table S10 Posterior difference in expected HR HPV cervical infection prevalence (in percentage points) in major French cities, between opportunistic and organised screening, stratified by type of test, city, and age. Table counterpart to Figure 3B. ***(continued)***

			Posterior distribution							
				Percentiles						
Virus	City	Age	Average	0.01	0.025	0.1	0.5	0.9	0.975	0.99
Other genotypes	Paris	45	5.16	2.02	2.46	3.37	5.16	6.92	7.81	8.50
Other genotypes	Paris	46	5.03	1.89	2.34	3.25	5.02	6.74	7.66	8.41
Other genotypes	Paris	47	4.90	1.81	2.23	3.14	4.89	6.62	7.56	8.30
Other genotypes	Paris	48	4.79	1.72	2.13	3.04	4.77	6.49	7.45	8.07
Other genotypes	Paris	49	4.68	1.62	2.03	2.93	4.66	6.36	7.31	7.97
Other genotypes	Paris	50	4.56	1.49	1.92	2.81	4.55	6.23	7.17	7.82
Other genotypes	Paris	51	4.44	1.42	1.78	2.72	4.44	6.12	7.03	7.66
Other genotypes	Paris	52	4.30	1.26	1.65	2.58	4.29	5.95	6.86	7.37
Other genotypes	Paris	53	4.14	1.16	1.50	2.45	4.13	5.77	6.64	7.17
Other genotypes	Paris	54	3.95	1.01	1.36	2.31	3.96	5.57	6.41	7.00
Other genotypes	Paris	55	3.77	0.88	1.21	2.16	3.77	5.34	6.18	6.78
Other genotypes	Paris	56	3.59	0.71	1.11	2.01	3.58	5.13	5.97	6.48
Other genotypes	Paris	57	3.41	0.56	0.97	1.88	3.41	4.93	5.72	6.24
Other genotypes	Paris	58	3.25	0.40	0.86	1.74	3.25	4.73	5.54	6.05
Other genotypes	Paris	59	3.10	0.32	0.75	1.63	3.11	4.58	5.38	5.82
Other genotypes	Paris	60	2.99	0.22	0.64	1.51	3.00	4.46	5.26	5.66
Other genotypes	Paris	61	2.90	0.07	0.56	1.40	2.90	4.40	5.18	5.60
Other genotypes	Paris	62	2.82	-0.08	0.52	1.32	2.83	4.34	5.11	5.51
Other genotypes	Paris	63	2.77	-0.18	0.44	1.26	2.78	4.32	5.13	5.56
Other genotypes	Paris	64	2.74	-0.31	0.31	1.17	2.73	4.32	5.13	5.60
Other genotypes	Paris	65	2.71	-0.41	0.20	1.05	2.71	4.36	5.24	5.66
Other genotypes	Paris	66	2.70	-0.56	0.07	0.94	2.71	4.44	5.40	5.78
Other genotypes	Rennes	30	9.95	5.50	6.07	7.36	9.90	12.64	14.04	14.98
Other genotypes	Rennes	31	9.55	5.36	5.91	7.07	9.51	12.15	13.40	14.29
Other genotypes	Rennes	32	9.10	5.15	5.58	6.73	9.08	11.57	12.74	13.56
Other genotypes	Rennes	33	8.62	4.85	5.30	6.36	8.59	10.95	12.06	12.83
Other genotypes	Rennes	34	8.19	4.55	5.04	6.04	8.14	10.39	11.51	12.28
Other genotypes	Rennes	35	7.83	4.36	4.81	5.74	7.77	9.95	11.04	11.77
Other genotypes	Rennes	36	7.49	4.12	4.61	5.47	7.43	9.53	10.60	11.27

Table S10 **Posterior difference in expected HR HPV cervical infection prevalence (in percentage points) in major French cities, between opportunistic and organised screening, stratified by type of test, city, and age. Table counterpart to Figure 3B.** ***(continued)***

			Posterior distribution							
				Percentiles						
Virus	City	Age	Average	0.01	0.025	0.1	0.5	0.9	0.975	0.99
Other genotypes	Rennes	37	7.20	3.91	4.43	5.26	7.13	9.20	10.24	10.87
Other genotypes	Rennes	38	6.93	3.72	4.24	5.07	6.86	8.87	9.90	10.48
Other genotypes	Rennes	39	6.67	3.54	4.06	4.85	6.61	8.55	9.56	10.11
Other genotypes	Rennes	40	6.41	3.37	3.87	4.65	6.35	8.26	9.22	9.75
Other genotypes	Rennes	41	6.17	3.22	3.68	4.46	6.12	7.94	8.90	9.38
Other genotypes	Rennes	42	5.96	3.12	3.52	4.29	5.92	7.73	8.63	9.13
Other genotypes	Rennes	43	5.78	2.97	3.39	4.12	5.75	7.51	8.39	8.90
Other genotypes	Rennes	44	5.62	2.81	3.24	4.00	5.59	7.27	8.23	8.71
Other genotypes	Rennes	45	5.47	2.72	3.11	3.89	5.45	7.11	8.05	8.49
Other genotypes	Rennes	46	5.34	2.61	3.01	3.78	5.31	6.98	7.90	8.39
Other genotypes	Rennes	47	5.22	2.54	2.91	3.68	5.18	6.83	7.75	8.29
Other genotypes	Rennes	48	5.12	2.41	2.82	3.57	5.08	6.72	7.62	8.19
Other genotypes	Rennes	49	5.02	2.30	2.74	3.47	4.98	6.62	7.51	8.11
Other genotypes	Rennes	50	4.92	2.24	2.64	3.37	4.87	6.50	7.42	8.00
Other genotypes	Rennes	51	4.81	2.19	2.58	3.28	4.76	6.40	7.25	7.85
Other genotypes	Rennes	52	4.67	2.05	2.46	3.15	4.63	6.25	7.10	7.65
Other genotypes	Rennes	53	4.52	1.93	2.30	3.02	4.48	6.09	6.95	7.45
Other genotypes	Rennes	54	4.34	1.78	2.17	2.88	4.32	5.88	6.70	7.19
Other genotypes	Rennes	55	4.17	1.61	2.01	2.73	4.15	5.69	6.48	6.94
Other genotypes	Rennes	56	4.00	1.54	1.85	2.59	3.98	5.50	6.27	6.73
Other genotypes	Rennes	57	3.83	1.35	1.71	2.46	3.82	5.30	6.04	6.47
Other genotypes	Rennes	58	3.68	1.26	1.58	2.32	3.67	5.13	5.84	6.32
Other genotypes	Rennes	59	3.55	1.16	1.48	2.20	3.53	4.98	5.68	6.15
Other genotypes	Rennes	60	3.44	1.05	1.36	2.10	3.42	4.85	5.55	6.04
Other genotypes	Rennes	61	3.36	0.94	1.25	2.02	3.34	4.78	5.47	5.90
Other genotypes	Rennes	62	3.30	0.83	1.15	1.95	3.28	4.72	5.45	5.82
Other genotypes	Rennes	63	3.27	0.73	1.05	1.88	3.25	4.71	5.44	5.83
Other genotypes	Rennes	64	3.25	0.58	0.98	1.83	3.24	4.73	5.49	5.90
Other genotypes	Rennes	65	3.25	0.49	0.90	1.80	3.23	4.78	5.56	6.03

Table S10 Posterior difference in expected HR HPV cervical infection prevalence (in percentage points) in major French cities, between opportunistic and organised screening, stratified by type of test, city, and age. Table counterpart to Figure 3B. ***(continued)***

			Posterior distribution							
				Percentiles						
Virus	City	Age	Average	0.01	0.025	0.1	0.5	0.9	0.975	0.99
Other genotypes	Rennes	66	3.27	0.30	0.80	1.72	3.24	4.86	5.68	6.25
Other genotypes	Strasbourg	30	6.82	1.96	2.70	4.02	6.66	9.82	11.78	13.13
Other genotypes	Strasbourg	31	6.52	1.89	2.62	3.87	6.31	9.45	11.30	12.60
Other genotypes	Strasbourg	32	6.18	1.76	2.49	3.68	5.99	8.99	10.74	11.93
Other genotypes	Strasbourg	33	5.82	1.62	2.32	3.46	5.64	8.50	10.19	11.33
Other genotypes	Strasbourg	34	5.50	1.48	2.15	3.28	5.33	8.06	9.74	10.90
Other genotypes	Strasbourg	35	5.24	1.42	2.02	3.10	5.05	7.69	9.29	10.44
Other genotypes	Strasbourg	36	4.99	1.33	1.88	2.91	4.81	7.35	8.92	9.96
Other genotypes	Strasbourg	37	4.77	1.26	1.74	2.77	4.62	7.04	8.61	9.57
Other genotypes	Strasbourg	38	4.58	1.19	1.64	2.62	4.42	6.79	8.24	9.27
Other genotypes	Strasbourg	39	4.39	1.10	1.55	2.48	4.23	6.54	8.00	8.94
Other genotypes	Strasbourg	40	4.19	1.00	1.45	2.34	4.04	6.28	7.62	8.62
Other genotypes	Strasbourg	41	4.02	0.92	1.36	2.23	3.88	6.03	7.37	8.27
Other genotypes	Strasbourg	42	3.87	0.89	1.27	2.11	3.73	5.84	7.16	7.97
Other genotypes	Strasbourg	43	3.74	0.82	1.20	2.01	3.60	5.68	6.96	7.80
Other genotypes	Strasbourg	44	3.62	0.73	1.12	1.94	3.48	5.50	6.82	7.63
Other genotypes	Strasbourg	45	3.51	0.66	1.06	1.85	3.38	5.35	6.63	7.44
Other genotypes	Strasbourg	46	3.41	0.62	1.02	1.79	3.29	5.26	6.53	7.33
Other genotypes	Strasbourg	47	3.32	0.50	0.97	1.72	3.20	5.13	6.44	7.16
Other genotypes	Strasbourg	48	3.24	0.48	0.93	1.66	3.13	5.02	6.32	7.04
Other genotypes	Strasbourg	49	3.16	0.47	0.89	1.59	3.05	4.94	6.19	6.96
Other genotypes	Strasbourg	50	3.08	0.38	0.83	1.52	2.98	4.85	6.11	6.89
Other genotypes	Strasbourg	51	3.00	0.34	0.77	1.46	2.90	4.73	5.98	6.74
Other genotypes	Strasbourg	52	2.89	0.29	0.71	1.39	2.81	4.60	5.83	6.58
Other genotypes	Strasbourg	53	2.78	0.24	0.63	1.29	2.69	4.47	5.66	6.42
Other genotypes	Strasbourg	54	2.65	0.17	0.54	1.20	2.55	4.29	5.44	6.18
Other genotypes	Strasbourg	55	2.52	0.05	0.46	1.10	2.41	4.09	5.28	5.93
Other genotypes	Strasbourg	56	2.40	-0.03	0.37	0.99	2.30	3.93	5.09	5.75
Other genotypes	Strasbourg	57	2.27	-0.10	0.27	0.91	2.17	3.79	4.90	5.59

Table S10 Posterior difference in expected HR HPV cervical infection prevalence (in percentage points) in major French cities, between opportunistic and organised screening, stratified by type of test, city, and age. Table counterpart to Figure 3B. ***(continued)***

			Posterior distribution							
				Percentiles						
Virus	City	Age	Average	0.01	0.025	0.1	0.5	0.9	0.975	0.99
Other genotypes	Strasbourg	58	2.16	-0.20	0.18	0.82	2.05	3.63	4.72	5.38
Other genotypes	Strasbourg	59	2.06	-0.24	0.11	0.76	1.96	3.49	4.60	5.19
Other genotypes	Strasbourg	60	1.98	-0.32	0.04	0.71	1.87	3.41	4.50	5.09
Other genotypes	Strasbourg	61	1.92	-0.42	-0.03	0.64	1.81	3.32	4.43	4.97
Other genotypes	Strasbourg	62	1.87	-0.47	-0.10	0.57	1.77	3.27	4.37	4.97
Other genotypes	Strasbourg	63	1.83	-0.53	-0.14	0.53	1.73	3.25	4.35	4.98
Other genotypes	Strasbourg	64	1.81	-0.65	-0.24	0.47	1.71	3.28	4.36	4.97
Other genotypes	Strasbourg	65	1.80	-0.75	-0.33	0.41	1.70	3.32	4.44	5.00
Other genotypes	Strasbourg	66	1.79	-0.89	-0.41	0.33	1.69	3.36	4.49	5.06
Other genotypes	Toulouse	30	6.65	1.36	2.35	3.88	6.54	9.61	11.27	12.11
Other genotypes	Toulouse	31	6.37	1.48	2.33	3.75	6.24	9.19	10.79	11.53
Other genotypes	Toulouse	32	6.04	1.48	2.18	3.57	5.94	8.75	10.28	11.10
Other genotypes	Toulouse	33	5.70	1.32	2.04	3.33	5.58	8.24	9.71	10.48
Other genotypes	Toulouse	34	5.39	1.30	1.95	3.11	5.28	7.84	9.25	9.95
Other genotypes	Toulouse	35	5.13	1.25	1.78	2.93	5.01	7.46	8.90	9.59
Other genotypes	Toulouse	36	4.88	1.01	1.63	2.79	4.77	7.14	8.49	9.26
Other genotypes	Toulouse	37	4.67	0.99	1.49	2.63	4.58	6.88	8.20	8.91
Other genotypes	Toulouse	38	4.48	0.88	1.40	2.48	4.40	6.61	7.91	8.62
Other genotypes	Toulouse	39	4.29	0.79	1.30	2.36	4.22	6.37	7.62	8.26
Other genotypes	Toulouse	40	4.09	0.73	1.17	2.21	4.02	6.12	7.34	7.97
Other genotypes	Toulouse	41	3.91	0.58	1.07	2.07	3.83	5.87	7.07	7.63
Other genotypes	Toulouse	42	3.76	0.48	0.98	1.97	3.68	5.69	6.87	7.37
Other genotypes	Toulouse	43	3.63	0.42	0.90	1.85	3.55	5.50	6.65	7.14
Other genotypes	Toulouse	44	3.50	0.34	0.85	1.77	3.43	5.35	6.51	6.99
Other genotypes	Toulouse	45	3.39	0.32	0.80	1.67	3.32	5.20	6.33	6.82
Other genotypes	Toulouse	46	3.29	0.27	0.73	1.58	3.23	5.08	6.16	6.69
Other genotypes	Toulouse	47	3.19	0.20	0.67	1.51	3.13	4.96	6.02	6.55
Other genotypes	Toulouse	48	3.11	0.15	0.62	1.46	3.04	4.85	5.91	6.38
Other genotypes	Toulouse	49	3.02	0.10	0.57	1.38	2.96	4.76	5.78	6.29

Table S10 **Posterior difference in expected HR HPV cervical infection prevalence (in percentage points) in major French cities, between opportunistic and organised screening, stratified by type of test, city, and age. Table counterpart to Figure 3B.** ***(continued)***

			Posterior distribution							
				Percentiles						
Virus	City	Age	Average	0.01	0.025	0.1	0.5	0.9	0.975	0.99
Other genotypes	Toulouse	50	2.94	0.05	0.50	1.31	2.88	4.63	5.66	6.20
Other genotypes	Toulouse	51	2.85	-0.07	0.40	1.24	2.79	4.52	5.56	6.08
Other genotypes	Toulouse	52	2.74	-0.16	0.32	1.15	2.68	4.39	5.39	5.92
Other genotypes	Toulouse	53	2.62	-0.25	0.24	1.06	2.57	4.26	5.21	5.75
Other genotypes	Toulouse	54	2.48	-0.39	0.14	0.95	2.44	4.06	5.00	5.56
Other genotypes	Toulouse	55	2.34	-0.49	0.06	0.85	2.30	3.89	4.83	5.36
Other genotypes	Toulouse	56	2.21	-0.56	-0.01	0.74	2.18	3.72	4.66	5.14
Other genotypes	Toulouse	57	2.08	-0.67	-0.11	0.64	2.05	3.57	4.45	4.97
Other genotypes	Toulouse	58	1.96	-0.81	-0.20	0.54	1.93	3.43	4.29	4.81
Other genotypes	Toulouse	59	1.86	-0.90	-0.28	0.45	1.82	3.32	4.15	4.64
Other genotypes	Toulouse	60	1.77	-0.98	-0.37	0.38	1.74	3.21	4.06	4.56
Other genotypes	Toulouse	61	1.70	-0.96	-0.43	0.32	1.66	3.13	4.00	4.46
Other genotypes	Toulouse	62	1.65	-1.07	-0.53	0.27	1.59	3.10	3.98	4.44
Other genotypes	Toulouse	63	1.60	-1.19	-0.59	0.20	1.55	3.07	3.95	4.44
Other genotypes	Toulouse	64	1.57	-1.27	-0.70	0.14	1.52	3.09	4.00	4.48
Other genotypes	Toulouse	65	1.55	-1.34	-0.77	0.04	1.50	3.10	4.06	4.53
Other genotypes	Toulouse	66	1.53	-1.49	-0.91	-0.04	1.48	3.16	4.10	4.62

Table S11 **Posterior percentage of postcodes with a greater expected infection prevalence under opportunistic screening, than under organised screening, stratified by age and genotype group, as of November 2023. Table counterpart to Figure 4A.**

		Posterior distribution							
			Percentiles						
Virus	Age	Average	0.01	0.025	0.1	0.5	0.9	0.975	0.99
HPV16/18	30	97.18	57.72	72.90	92.79	99.85	100.00	100.00	100.00
HPV16/18	31	97.75	64.86	78.97	94.58	99.88	100.00	100.00	100.00
HPV16/18	32	98.11	70.76	82.61	95.54	99.90	100.00	100.00	100.00

Table S11 **Posterior percentage of postcodes with a greater expected infection prevalence under opportunistic screening, than under organised screening, stratified by age and genotype group, as of November 2023. Table counterpart to Figure 4A.** ***(continued)***

		Posterior distribution							
			Percentiles						
Virus	Age	Average	0.01	0.025	0.1	0.5	0.9	0.975	0.99
HPV16/18	33	98.36	74.42	85.45	96.10	99.92	100.00	100.00	100.00
HPV16/18	34	98.51	76.23	86.59	96.44	99.92	100.00	100.00	100.00
HPV16/18	35	98.59	76.76	87.55	96.67	99.92	100.00	100.00	100.00
HPV16/18	36	98.64	78.88	87.87	96.80	99.92	100.00	100.00	100.00
HPV16/18	37	98.64	78.97	87.77	96.87	99.92	100.00	100.00	100.00
HPV16/18	38	98.64	78.73	87.67	96.93	99.92	100.00	100.00	100.00
HPV16/18	39	98.62	77.87	87.42	96.85	99.90	100.00	100.00	100.00
HPV16/18	40	98.57	78.00	87.12	96.77	99.90	100.00	100.00	100.00
HPV16/18	41	98.51	77.14	86.46	96.50	99.90	100.00	100.00	100.00
HPV16/18	42	98.44	75.87	85.75	96.30	99.88	100.00	100.00	100.00
HPV16/18	43	98.38	74.61	84.75	96.14	99.88	100.00	100.00	100.00
HPV16/18	44	98.31	73.11	84.55	95.92	99.88	100.00	100.00	100.00
HPV16/18	45	98.23	72.04	83.61	95.74	99.88	100.00	100.00	100.00
HPV16/18	46	98.14	70.64	83.11	95.51	99.87	100.00	100.00	100.00
HPV16/18	47	98.06	69.52	83.13	95.19	99.85	100.00	100.00	100.00
HPV16/18	48	97.94	68.92	82.12	94.83	99.85	100.00	100.00	100.00
HPV16/18	49	97.85	67.68	81.73	94.40	99.83	100.00	100.00	100.00
HPV16/18	50	97.71	65.90	80.71	94.13	99.82	100.00	100.00	100.00
HPV16/18	51	97.58	64.45	79.79	93.67	99.80	100.00	100.00	100.00
HPV16/18	52	97.43	64.15	78.92	93.05	99.78	100.00	100.00	100.00
HPV16/18	53	97.23	63.50	77.21	92.46	99.75	100.00	100.00	100.00
HPV16/18	54	97.00	60.57	74.69	91.76	99.72	100.00	100.00	100.00
HPV16/18	55	96.75	55.99	73.63	91.02	99.69	100.00	100.00	100.00
HPV16/18	56	96.49	55.41	71.14	90.00	99.64	100.00	100.00	100.00
HPV16/18	57	96.15	52.64	69.07	88.91	99.59	100.00	100.00	100.00
HPV16/18	58	95.81	50.06	67.08	87.75	99.54	100.00	100.00	100.00
HPV16/18	59	95.43	48.62	64.48	86.37	99.47	100.00	100.00	100.00
HPV16/18	60	95.01	44.11	61.30	84.87	99.42	100.00	100.00	100.00
HPV16/18	61	94.53	41.95	57.65	83.72	99.35	100.00	100.00	100.00

Table S11 **Posterior percentage of postcodes with a greater expected infection prevalence under opportunistic screening, than under organised screening, stratified by age and genotype group, as of November 2023. Table counterpart to Figure 4A.** ***(continued)***

		Posterior distribution							
			Percentiles						
Virus	Age	Average	0.01	0.025	0.1	0.5	0.9	0.975	0.99
HPV16/18	62	93.98	37.41	53.77	81.45	99.32	100.00	100.00	100.00
HPV16/18	63	93.30	32.11	48.47	78.85	99.20	100.00	100.00	100.00
HPV16/18	64	92.51	26.80	43.74	74.77	99.09	100.00	100.00	100.00
HPV16/18	65	91.58	22.10	37.81	71.11	98.99	100.00	100.00	100.00
HPV16/18	66	90.50	17.11	31.43	67.41	98.82	100.00	100.00	100.00
Other genotypes	30	99.92	98.77	99.34	99.85	100.00	100.00	100.00	100.00
Other genotypes	31	99.92	98.94	99.35	99.85	100.00	100.00	100.00	100.00
Other genotypes	32	99.93	98.94	99.35	99.85	100.00	100.00	100.00	100.00
Other genotypes	33	99.92	98.84	99.32	99.85	100.00	100.00	100.00	100.00
Other genotypes	34	99.92	98.76	99.27	99.83	100.00	100.00	100.00	100.00
Other genotypes	35	99.92	98.62	99.17	99.80	100.00	100.00	100.00	100.00
Other genotypes	36	99.90	98.49	99.17	99.78	100.00	100.00	100.00	100.00
Other genotypes	37	99.88	98.26	99.07	99.75	100.00	100.00	100.00	100.00
Other genotypes	38	99.88	98.06	98.96	99.72	100.00	100.00	100.00	100.00
Other genotypes	39	99.87	97.56	98.87	99.69	99.98	100.00	100.00	100.00
Other genotypes	40	99.83	97.23	98.71	99.62	99.98	100.00	100.00	100.00
Other genotypes	41	99.82	96.87	98.44	99.57	99.98	100.00	100.00	100.00
Other genotypes	42	99.78	96.42	98.16	99.50	99.98	100.00	100.00	100.00
Other genotypes	43	99.77	95.91	97.96	99.44	99.98	100.00	100.00	100.00
Other genotypes	44	99.72	95.91	97.56	99.34	99.98	100.00	100.00	100.00
Other genotypes	45	99.69	95.24	97.31	99.27	99.97	100.00	100.00	100.00
Other genotypes	46	99.64	94.60	96.85	99.17	99.97	100.00	100.00	100.00
Other genotypes	47	99.59	93.90	96.42	99.02	99.97	100.00	100.00	100.00
Other genotypes	48	99.54	93.32	95.84	98.87	99.95	100.00	100.00	100.00
Other genotypes	49	99.47	92.34	95.47	98.72	99.95	100.00	100.00	100.00
Other genotypes	50	99.40	91.84	94.93	98.48	99.93	100.00	100.00	100.00
Other genotypes	51	99.30	91.18	94.36	98.24	99.92	100.00	100.00	100.00
Other genotypes	52	99.19	89.56	93.49	97.93	99.90	100.00	100.00	100.00
Other genotypes	53	99.04	87.85	92.29	97.58	99.88	100.00	100.00	100.00

Table S11 Posterior percentage of postcodes with a greater expected infection prevalence under opportunistic screening, than under organised screening, stratified by age and genotype group, as of November 2023. Table counterpart to Figure 4A. ***(continued)***

		Posterior distribution							
			Percentiles						
Virus	Age	Average	0.01	0.025	0.1	0.5	0.9	0.975	0.99
Other genotypes	54	98.84	85.76	91.18	97.03	99.83	100.00	100.00	100.00
Other genotypes	55	98.61	82.89	89.41	96.22	99.78	100.00	100.00	100.00
Other genotypes	56	98.33	80.37	87.45	95.28	99.70	100.00	100.00	100.00
Other genotypes	57	97.98	77.41	85.36	94.33	99.62	100.00	100.00	100.00
Other genotypes	58	97.60	74.69	83.09	93.32	99.50	100.00	100.00	100.00
Other genotypes	59	97.15	72.40	80.66	91.96	99.35	100.00	100.00	100.00
Other genotypes	60	96.68	71.01	77.71	90.52	99.19	100.00	100.00	100.00
Other genotypes	61	96.19	67.06	75.39	88.84	99.04	100.00	100.00	100.00
Other genotypes	62	95.64	61.40	72.75	87.29	98.84	100.00	100.00	100.00
Other genotypes	63	95.01	56.41	68.84	85.25	98.61	100.00	100.00	100.00
Other genotypes	64	94.30	51.33	64.25	82.84	98.41	100.00	100.00	100.00
Other genotypes	65	93.49	43.54	58.86	80.14	98.24	100.00	100.00	100.00
Other genotypes	66	92.49	41.07	52.69	76.79	97.94	100.00	100.00	100.00

Table S12 Posterior distribution of the difference in the number of postcodes with a higher expected prevalence under opportunistic screening versus organised screening at each age, relative to females aged 30, as of end of November 2023. Table counterpart to Figure 4B.

		Posterior distribution								
			Percentiles							
Virus	Age	Average	0.01	0.025	0.1	0.5	0.9	0.975	0.99	Proba < 0
HPV16/18	31	34	-21	-8	-1	0	83	363	595	0.12
HPV16/18	32	56	-47	-16	-2	0	140	606	1,010	0.15
HPV16/18	33	71	-72	-30	-3	0	174	784	1,272	0.17
HPV16/18	34	80	-100	-44	-5	0	196	915	1,466	0.19
HPV16/18	35	85	-149	-58	-7	0	210	1,016	1,559	0.21
HPV16/18	36	88	-185	-84	-10	0	219	1,060	1,623	0.23
HPV16/18	37	88	-246	-115	-14	0	223	1,087	1,689	0.26

Table S12 Posterior distribution of the difference in the number of postcodes with a higher expected prevalence under opportunistic screening versus organised screening at each age, relative to females aged 30, as of end of November 2023. Table counterpart to Figure 4B. ***(continued)***

		Posterior distribution								
			Percentiles							
Virus	Age	Average	0.01	0.025	0.1	0.5	0.9	0.975	0.99	Proba < 0
HPV16/18	38	88	-306	-158	-18	0	227	1,103	1,732	0.27
HPV16/18	39	87	-384	-196	-26	0	236	1,143	1,854	0.28
HPV16/18	40	84	-462	-219	-34	0	232	1,155	1,857	0.30
HPV16/18	41	80	-542	-254	-43	0	233	1,130	1,878	0.31
HPV16/18	42	76	-607	-304	-50	0	236	1,126	1,908	0.33
HPV16/18	43	72	-700	-341	-55	0	232	1,118	1,921	0.34
HPV16/18	44	68	-762	-398	-65	0	235	1,121	1,903	0.35
HPV16/18	45	63	-869	-443	-81	0	231	1,136	1,907	0.36
HPV16/18	46	58	-914	-491	-89	0	229	1,133	1,907	0.37
HPV16/18	47	52	-908	-525	-99	0	227	1,117	1,905	0.39
HPV16/18	48	46	-1,030	-571	-112	0	220	1,124	1,902	0.40
HPV16/18	49	40	-1,104	-652	-133	0	220	1,128	1,900	0.41
HPV16/18	50	32	-1,154	-713	-146	0	209	1,115	1,908	0.43
HPV16/18	51	24	-1,297	-791	-162	0	207	1,113	1,886	0.44
HPV16/18	52	14	-1,376	-839	-182	0	191	1,076	1,886	0.46
HPV16/18	53	3	-1,479	-892	-208	0	187	1,023	1,895	0.47
HPV16/18	54	-11	-1,569	-1,001	-245	0	174	979	1,898	0.49
HPV16/18	55	-26	-1,645	-1,133	-304	-1	163	951	1,842	0.51
HPV16/18	56	-43	-1,850	-1,275	-352	-1	150	951	1,878	0.52
HPV16/18	57	-62	-2,086	-1,353	-406	-2	139	911	1,854	0.54
HPV16/18	58	-83	-2,271	-1,536	-450	-3	129	876	1,804	0.56
HPV16/18	59	-106	-2,325	-1,623	-515	-4	108	848	1,774	0.58
HPV16/18	60	-131	-2,510	-1,807	-611	-5	101	780	1,676	0.59
HPV16/18	61	-160	-2,712	-2,001	-719	-7	93	762	1,588	0.60
HPV16/18	62	-193	-3,054	-2,179	-818	-9	85	731	1,565	0.61
HPV16/18	63	-234	-3,556	-2,458	-971	-12	76	701	1,516	0.63
HPV16/18	64	-282	-3,899	-2,726	-1,195	-15	73	697	1,485	0.64
HPV16/18	65	-338	-4,244	-3,146	-1,420	-18	65	657	1,432	0.65

Table S12 **Posterior distribution of the difference in the number of postcodes with a higher expected prevalence under opportunistic screening versus organised screening at each age, relative to females aged 30, as of end of November 2023. Table counterpart to Figure 4B.** ***(continued)***

		Posterior distribution								
			Percentiles							
Virus	Age	Average	0.01	0.025	0.1	0.5	0.9	0.975	0.99	Proba < 0
HPV16/18	66	-403	-4,592	-3,597	-1,653	-23	61	636	1,467	0.66
Other genotypes	31	1	-6	-3	0	0	0	6	15	0.10
Other genotypes	32	1	-13	-6	-1	0	1	8	22	0.14
Other genotypes	33	1	-19	-10	-2	0	1	10	25	0.19
Other genotypes	34	0	-28	-15	-3	0	1	11	28	0.22
Other genotypes	35	0	-41	-19	-4	0	1	11	32	0.25
Other genotypes	36	-1	-50	-24	-5	0	1	11	35	0.28
Other genotypes	37	-1	-64	-30	-7	0	0	10	34	0.31
Other genotypes	38	-2	-77	-37	-8	0	0	10	33	0.34
Other genotypes	39	-3	-91	-45	-10	0	0	10	31	0.37
Other genotypes	40	-4	-110	-57	-13	0	0	8	30	0.40
Other genotypes	41	-6	-129	-71	-17	0	0	8	31	0.43
Other genotypes	42	-8	-150	-85	-20	0	0	8	32	0.45
Other genotypes	43	-9	-178	-103	-24	0	0	7	29	0.47
Other genotypes	44	-11	-210	-120	-29	0	0	6	27	0.49
Other genotypes	45	-14	-239	-143	-34	-1	0	6	29	0.52
Other genotypes	46	-16	-277	-163	-42	-1	0	5	22	0.55
Other genotypes	47	-20	-326	-191	-49	-1	0	4	18	0.57
Other genotypes	48	-23	-357	-216	-58	-2	0	3	13	0.59
Other genotypes	49	-27	-432	-244	-66	-2	0	2	11	0.61
Other genotypes	50	-31	-446	-276	-79	-3	0	1	11	0.64
Other genotypes	51	-37	-514	-315	-94	-4	0	0	6	0.66
Other genotypes	52	-44	-591	-362	-113	-5	0	0	6	0.68
Other genotypes	53	-53	-713	-426	-138	-6	0	0	3	0.70
Other genotypes	54	-65	-811	-513	-169	-9	0	0	1	0.73
Other genotypes	55	-79	-995	-598	-217	-12	0	0	0	0.75
Other genotypes	56	-96	-1,136	-716	-269	-17	0	0	0	0.78
Other genotypes	57	-116	-1,324	-837	-323	-22	0	0	0	0.80

Table S12 Posterior distribution of the difference in the number of postcodes with a higher expected prevalence under opportunistic screening versus organised screening at each age, relative to females aged 30, as of end of November 2023. Table counterpart to Figure 4B. ***(continued)***

		Posterior distribution								
			Percentiles							
Virus	Age	Average	0.01	0.025	0.1	0.5	0.9	0.975	0.99	Proba < 0
Other genotypes	58	-140	-1,484	-981	-392	-29	0	0	0	0.83
Other genotypes	59	-166	-1,622	-1,122	-476	-37	0	0	0	0.84
Other genotypes	60	-194	-1,730	-1,295	-564	-47	0	0	0	0.86
Other genotypes	61	-224	-1,948	-1,442	-663	-56	0	0	0	0.87
Other genotypes	62	-258	-2,235	-1,626	-753	-69	0	0	0	0.88
Other genotypes	63	-296	-2,579	-1,854	-872	-81	0	0	0	0.89
Other genotypes	64	-339	-2,914	-2,134	-1,017	-94	0	0	0	0.89
Other genotypes	65	-388	-3,335	-2,459	-1,185	-104	0	0	0	0.89
Other genotypes	66	-448	-3,514	-2,821	-1,380	-122	0	0	0	0.89

S12 Difference in expected HR HPV cervical infection prevalence, between the two groups of genotypes

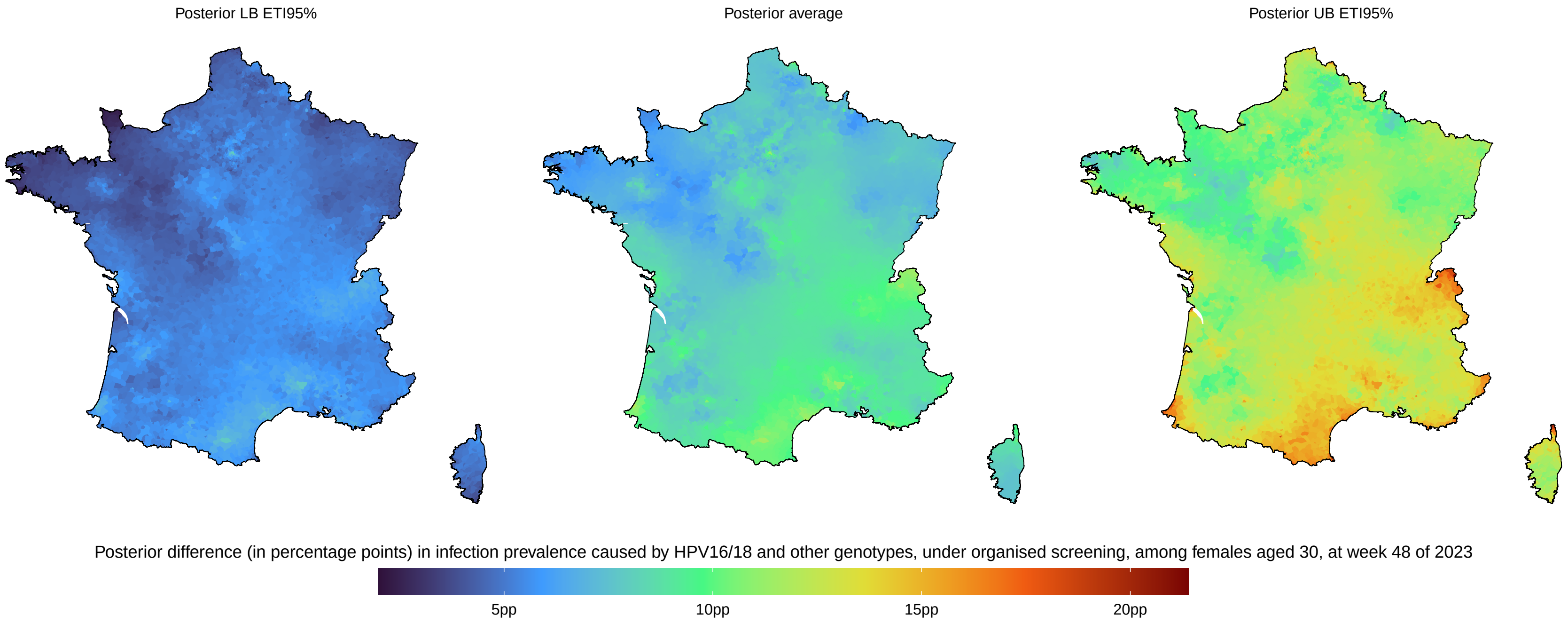

Figure S34 Summary of the posterior distribution of the difference (in percentage points) in infection prevalence caused by HPV16/18 and other genotypes, under organised screening, among females aged 30, at week 48 of 2023.

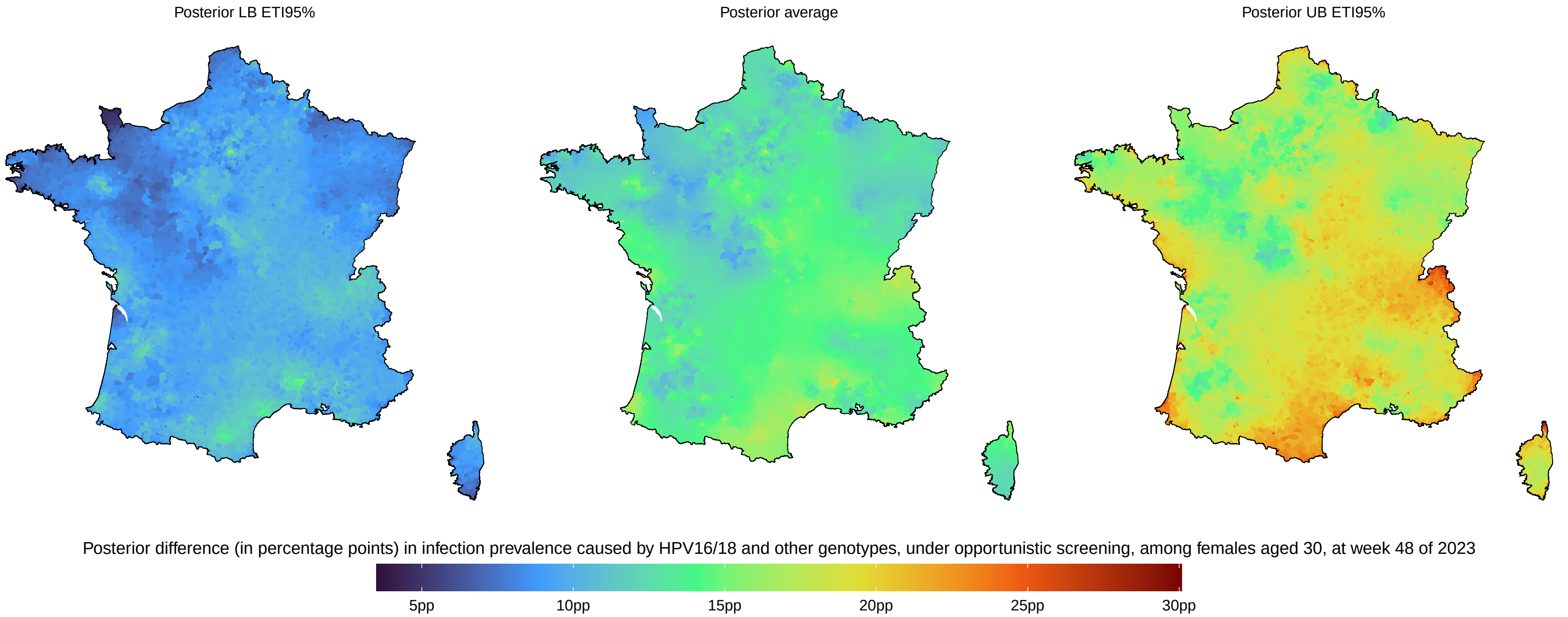

Figure S35 **Summary of the posterior distribution of the difference (in percentage points) in infection prevalence caused by HPV16/18 and other genotypes, under opportunistic screening, among females aged 30, at week 48 of 2023.**

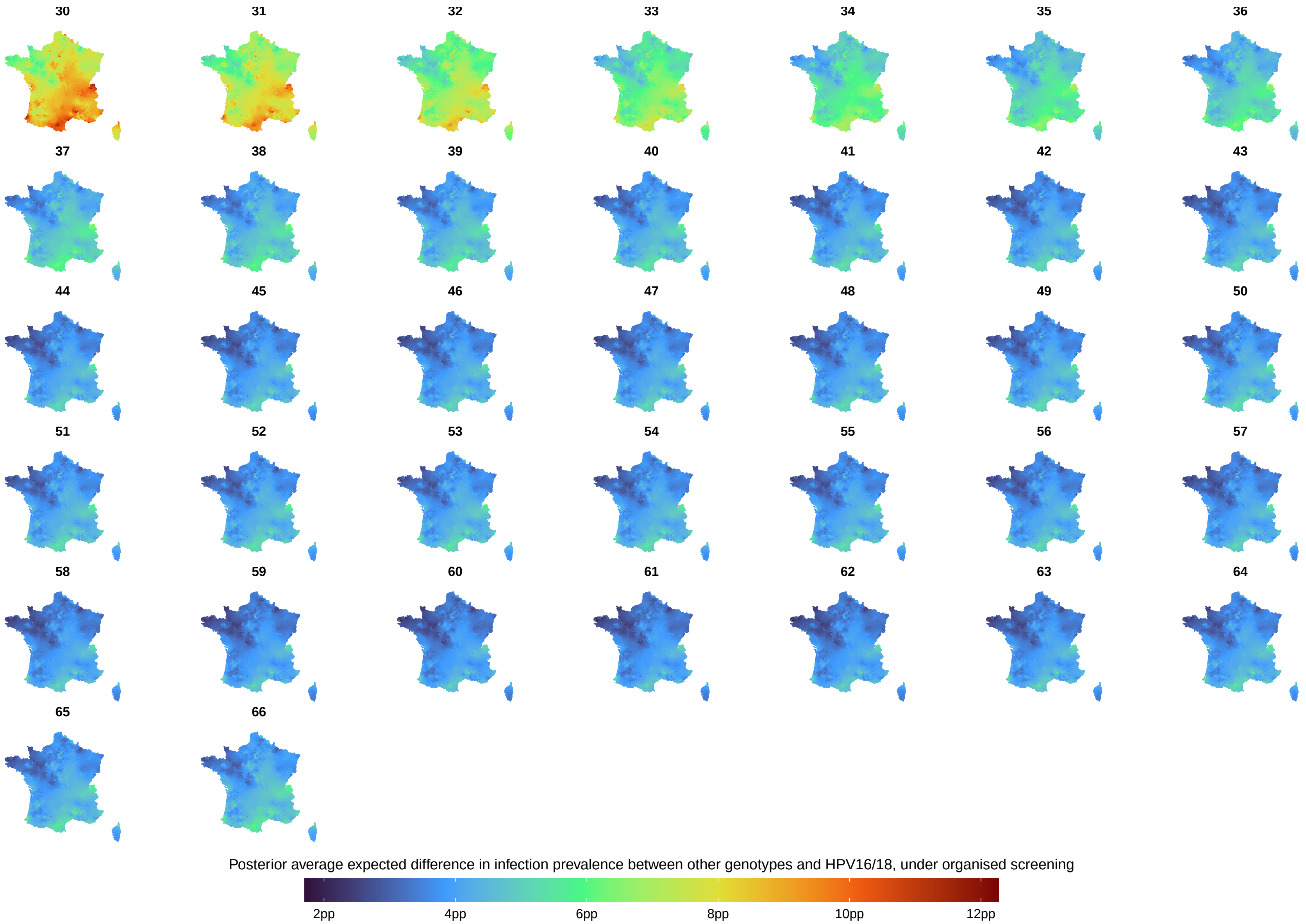

Figure S36 Posterior average expected difference in infection prevalence between other genotypes and HPV16/18, across metropolitan France, stratified by age, under organised screening.

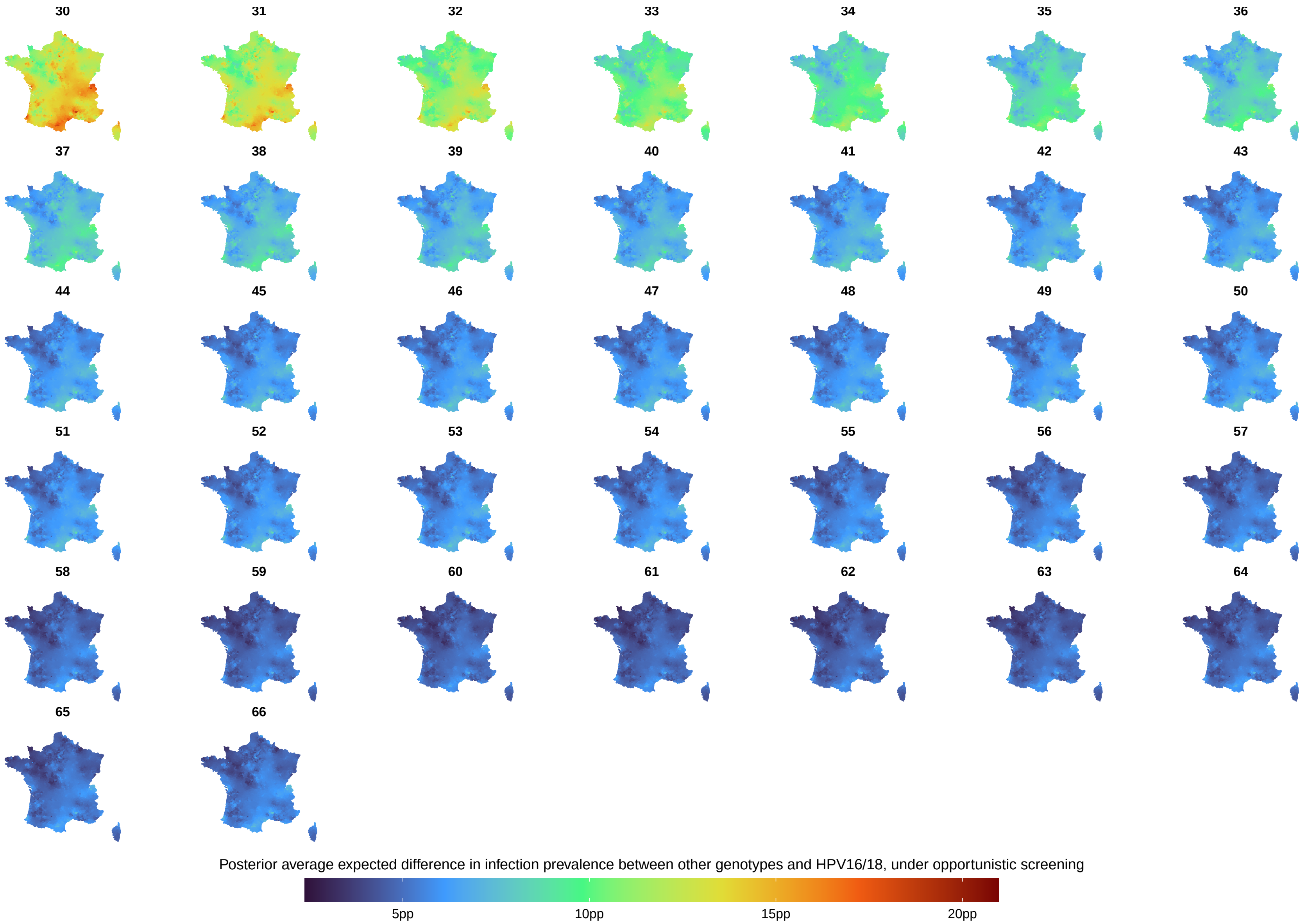

Figure S37 Posterior average expected difference in infection prevalence between other genotypes and HPV16/18, across metropolitan France, stratified by age, under opportunistic screening.

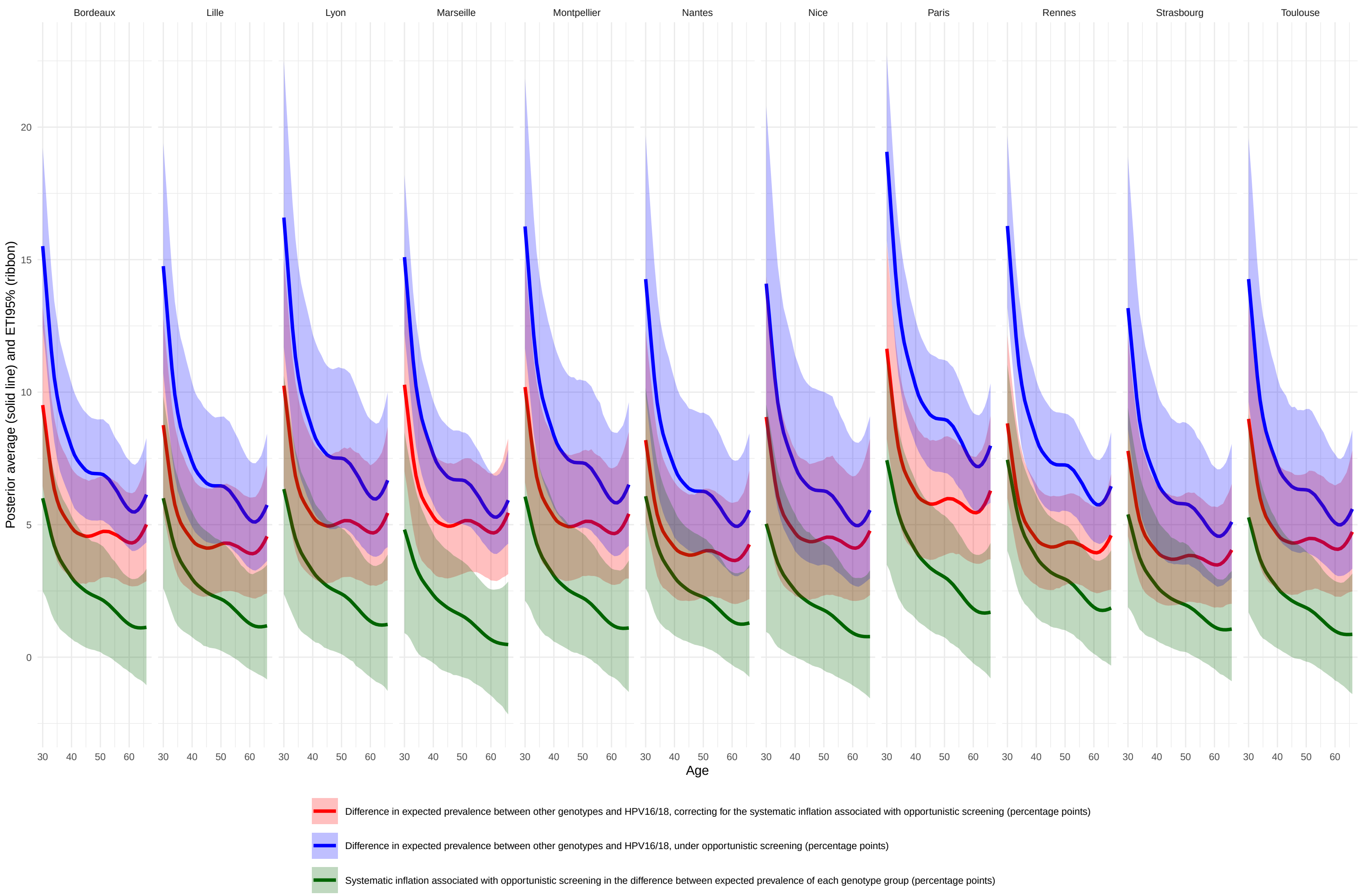

Figure S38 **Summary of the posterior distribution of the difference (in percentage points), between opportunistic and organised screening, in the expected HR HPV cervical infection prevalence, in major French cities, at week 48 of 2023, stratified by age and genotype group.**

S13 Difference in expected HR HPV cervical prevalence between the last and first week of the study period, stratified by age

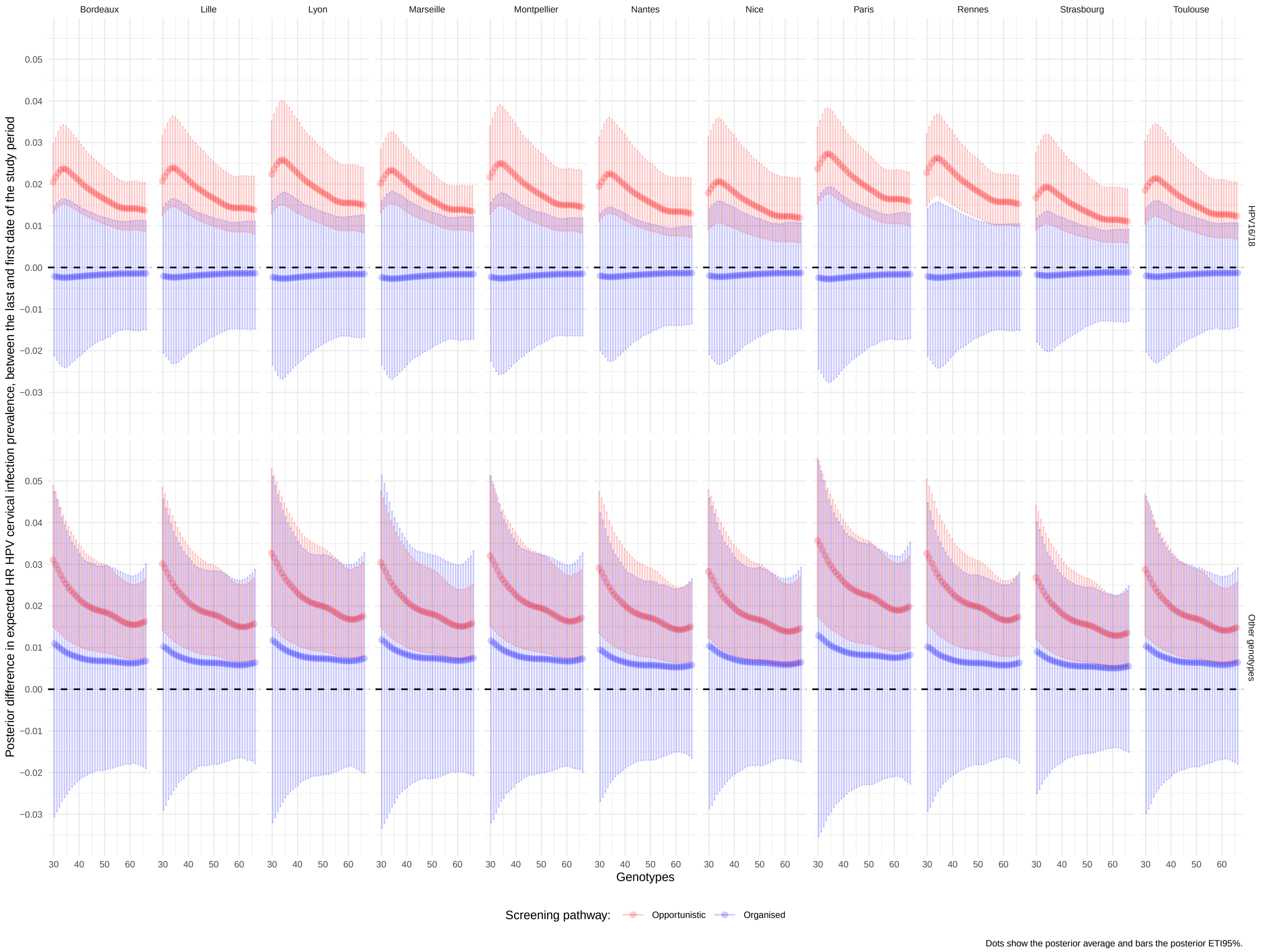

Figure S39 Difference in expected HR HPV cervical infection prevalence, between the last and first week of the study period in major French cities, stratified by screening pathway and age, for HPV16/18.

S14 Figure 5B and C in table format

Table S13 Posterior expected HR HPV infection prevalence (in %) assuming all data would have been collected with opportunistic or organised screening, not stratified or stratified by age, year, or age and year. Provides values from Figure 5B in table format.

			Posterior distribution							
				Percentiles						
Genotype	Year	Age	Average	0.01	0.025	0.1	0.5	0.9	0.975	0.99
HPV16/18	2020		3.19	3.05	3.08	3.12	3.19	3.27	3.32	3.34
HPV16/18	2021		3.65	3.54	3.56	3.59	3.65	3.70	3.74	3.75
HPV16/18	2022		4.03	3.86	3.89	3.93	4.02	4.12	4.17	4.19
HPV16/18	2023		4.50	4.31	4.34	4.40	4.50	4.61	4.67	4.70
HPV16/18	2020		3.44	2.38	2.50	2.80	3.39	4.15	4.61	4.81
HPV16/18	2021		3.31	2.66	2.77	2.94	3.30	3.70	3.95	4.10
HPV16/18	2022		3.08	2.72	2.76	2.87	3.07	3.29	3.41	3.47
HPV16/18	2023		3.13	2.69	2.76	2.88	3.13	3.38	3.51	3.60
Other genotypes	2020		9.45	9.23	9.26	9.32	9.45	9.58	9.65	9.69
Other genotypes	2021		8.80	8.64	8.67	8.70	8.80	8.89	8.95	8.97
Other genotypes	2022		9.30	9.07	9.11	9.17	9.30	9.44	9.51	9.54
Other genotypes	2023		10.95	10.66	10.70	10.78	10.95	11.11	11.21	11.25
Other genotypes	2020		7.17	5.49	5.72	6.14	7.13	8.27	8.89	9.26
Other genotypes	2021		7.02	6.09	6.20	6.49	7.01	7.57	7.87	8.04
Other genotypes	2022		7.47	6.88	6.94	7.13	7.47	7.81	7.97	8.07
Other genotypes	2023		7.75	7.07	7.16	7.34	7.74	8.15	8.40	8.50
HPV16/18		[30,39]	4.24	3.24	3.37	3.59	4.50	4.65	4.70	4.73
HPV16/18		(39,49]	3.49	2.71	2.80	3.00	3.67	3.81	3.86	3.89
HPV16/18		(49,59]	2.83	2.28	2.34	2.49	2.93	3.05	3.11	3.14
HPV16/18		(59,66]	2.69	2.16	2.24	2.40	2.74	2.89	2.97	3.06
Other genotypes		[30,39]	10.64	8.03	8.20	8.53	11.33	12.38	12.45	12.48
Other genotypes		(39,49]	7.60	5.89	6.00	6.27	8.19	8.66	8.73	8.76

Table S13 **Posterior expected HR HPV infection prevalence (in %) assuming all data would have been collected with opportunistic or organised screening, not stratified or stratified by age, year, or age and year. Provides values from Figure 5B in table format.** ***(continued)***

			Posterior distribution							
				Percentiles						
Genotype	Year	Age	Average	0.01	0.025	0.1	0.5	0.9	0.975	0.99
Other genotypes		(49,59]	6.67	5.46	5.55	5.80	7.03	7.34	7.42	7.46
Other genotypes		(59,66]	6.17	5.23	5.33	5.60	6.31	6.57	6.68	6.73
HPV16/18	2020	[30,39]	3.87	3.67	3.71	3.76	3.86	3.97	4.04	4.07
HPV16/18	2020	(39,49]	3.18	3.01	3.05	3.09	3.18	3.27	3.32	3.35
HPV16/18	2020	(49,59]	2.53	2.39	2.41	2.45	2.53	2.62	2.65	2.68
HPV16/18	2020	(59,66]	2.35	2.17	2.20	2.25	2.35	2.45	2.50	2.53
HPV16/18	2021	[30,39]	4.46	4.30	4.33	4.37	4.46	4.54	4.58	4.60
HPV16/18	2021	(39,49]	3.65	3.50	3.53	3.57	3.65	3.73	3.78	3.81
HPV16/18	2021	(49,59]	2.91	2.77	2.79	2.84	2.91	2.99	3.02	3.06
HPV16/18	2021	(59,66]	2.70	2.53	2.55	2.60	2.70	2.80	2.86	2.89
HPV16/18	2022	[30,39]	4.89	4.67	4.70	4.77	4.89	5.01	5.08	5.11
HPV16/18	2022	(39,49]	4.01	3.82	3.84	3.90	4.01	4.11	4.18	4.21
HPV16/18	2022	(49,59]	3.19	3.03	3.05	3.09	3.19	3.29	3.34	3.37
HPV16/18	2022	(59,66]	2.95	2.73	2.77	2.83	2.95	3.08	3.14	3.17
HPV16/18	2023	[30,39]	5.45	5.20	5.23	5.31	5.45	5.59	5.66	5.71
HPV16/18	2023	(39,49]	4.50	4.27	4.31	4.36	4.50	4.63	4.70	4.74
HPV16/18	2023	(49,59]	3.58	3.38	3.41	3.47	3.58	3.70	3.76	3.81
HPV16/18	2023	(59,66]	3.31	3.05	3.10	3.17	3.31	3.45	3.52	3.56
HPV16/18	2020	[30,39]	4.09	2.77	2.94	3.27	4.03	5.00	5.56	5.90
HPV16/18	2020	(39,49]	3.39	2.33	2.43	2.73	3.34	4.11	4.55	4.79
HPV16/18	2020	(49,59]	2.80	1.92	2.04	2.28	2.76	3.36	3.73	3.89
HPV16/18	2020	(59,66]	2.71	1.91	1.99	2.21	2.68	3.25	3.62	3.82
HPV16/18	2021	[30,39]	3.97	3.11	3.24	3.44	3.95	4.51	4.86	5.08
HPV16/18	2021	(39,49]	3.28	2.60	2.67	2.87	3.27	3.70	3.94	4.09
HPV16/18	2021	(49,59]	2.71	2.17	2.25	2.40	2.71	3.05	3.22	3.33

Table S13 **Posterior expected HR HPV infection prevalence (in %) assuming all data would have been collected with opportunistic or organised screening, not stratified or stratified by age, year, or age and year. Provides values from Figure 5B in table format.** ***(continued)***

			Posterior distribution							
				Percentiles						
Genotype	Year	Age	Average	0.01	0.025	0.1	0.5	0.9	0.975	0.99
HPV16/18	2021	(59,66]	2.64	2.10	2.16	2.30	2.63	2.97	3.20	3.28
HPV16/18	2022	[30,39]	3.67	3.09	3.19	3.35	3.66	4.01	4.18	4.27
HPV16/18	2022	(39,49]	3.03	2.60	2.67	2.79	3.02	3.28	3.41	3.49
HPV16/18	2022	(49,59]	2.50	2.17	2.20	2.30	2.50	2.71	2.82	2.90
HPV16/18	2022	(59,66]	2.42	1.98	2.05	2.17	2.42	2.70	2.84	2.93
HPV16/18	2023	[30,39]	3.73	3.12	3.19	3.36	3.73	4.12	4.27	4.40
HPV16/18	2023	(39,49]	3.09	2.62	2.69	2.82	3.08	3.36	3.51	3.61
HPV16/18	2023	(49,59]	2.55	2.13	2.19	2.32	2.54	2.80	2.95	3.04
HPV16/18	2023	(59,66]	2.46	1.92	2.02	2.18	2.45	2.76	2.92	3.06
Other genotypes	2020	[30,39]	12.31	11.99	12.03	12.12	12.30	12.49	12.59	12.63
Other genotypes	2020	(39,49]	8.65	8.39	8.42	8.50	8.64	8.79	8.87	8.91
Other genotypes	2020	(49,59]	7.31	7.07	7.10	7.16	7.30	7.45	7.53	7.58
Other genotypes	2020	(59,66]	6.48	6.16	6.22	6.30	6.47	6.66	6.75	6.81
Other genotypes	2021	[30,39]	11.58	11.33	11.36	11.44	11.58	11.72	11.80	11.84
Other genotypes	2021	(39,49]	8.12	7.91	7.94	8.00	8.12	8.23	8.30	8.33
Other genotypes	2021	(49,59]	6.85	6.64	6.68	6.74	6.85	6.97	7.04	7.08
Other genotypes	2021	(59,66]	6.12	5.84	5.88	5.96	6.11	6.27	6.37	6.41
Other genotypes	2022	[30,39]	12.15	11.82	11.87	11.97	12.15	12.34	12.43	12.46
Other genotypes	2022	(39,49]	8.47	8.23	8.25	8.33	8.47	8.62	8.70	8.75
Other genotypes	2022	(49,59]	7.17	6.89	6.95	7.02	7.16	7.31	7.39	7.45
Other genotypes	2022	(59,66]	6.36	6.06	6.10	6.19	6.36	6.54	6.64	6.69
Other genotypes	2023	[30,39]	14.28	13.88	13.94	14.05	14.28	14.51	14.63	14.67
Other genotypes	2023	(39,49]	9.99	9.67	9.71	9.80	9.99	10.18	10.28	10.32
Other genotypes	2023	(49,59]	8.45	8.15	8.20	8.28	8.45	8.64	8.73	8.80
Other genotypes	2023	(59,66]	7.49	7.12	7.19	7.28	7.49	7.70	7.82	7.88

Table S13 Posterior expected HR HPV infection prevalence (in %) assuming all data would have been collected with opportunistic or organised screening, not stratified or stratified by age, year, or age and year. Provides values from Figure 5B in table format. ***(continued)***

			Posterior distribution							
				Percentiles						
Genotype	Year	Age	Average	0.01	0.025	0.1	0.5	0.9	0.975	0.99
Other genotypes	2020	[30,39]	8.83	6.68	7.04	7.56	8.78	10.24	11.05	11.49
Other genotypes	2020	(39,49]	6.53	4.93	5.15	5.54	6.50	7.56	8.21	8.55
Other genotypes	2020	(49,59]	6.00	4.59	4.77	5.09	5.95	6.94	7.51	7.87
Other genotypes	2020	(59,66]	5.78	4.45	4.58	4.94	5.75	6.63	7.17	7.59
Other genotypes	2021	[30,39]	8.69	7.45	7.62	7.98	8.67	9.47	9.92	10.11
Other genotypes	2021	(39,49]	6.42	5.48	5.60	5.85	6.41	7.02	7.36	7.53
Other genotypes	2021	(49,59]	5.90	5.08	5.17	5.42	5.90	6.41	6.64	6.79
Other genotypes	2021	(59,66]	5.74	4.91	5.00	5.24	5.75	6.23	6.49	6.62
Other genotypes	2022	[30,39]	9.20	8.28	8.39	8.67	9.19	9.77	10.04	10.22
Other genotypes	2022	(39,49]	6.77	6.11	6.19	6.38	6.76	7.15	7.39	7.51
Other genotypes	2022	(49,59]	6.24	5.62	5.70	5.87	6.23	6.61	6.80	6.87
Other genotypes	2022	(59,66]	6.03	5.30	5.40	5.58	6.02	6.49	6.75	6.88
Other genotypes	2023	[30,39]	9.57	8.59	8.70	8.95	9.56	10.20	10.54	10.77
Other genotypes	2023	(39,49]	7.01	6.27	6.38	6.59	7.00	7.47	7.71	7.88
Other genotypes	2023	(49,59]	6.45	5.75	5.85	6.04	6.44	6.86	7.08	7.19
Other genotypes	2023	(59,66]	6.21	5.38	5.50	5.72	6.19	6.73	6.99	7.13

Table S14 Marginal Difference in Expected Prevalence (in percentage points), not stratified or stratified by age, year, or age and year. Provides values from Figure 5C in table format.

			Posterior distribution								
				Percentiles							
Genotype	Year	Age	Average	0.01	0.025	0.1	0.5	0.9	0.975	0.99	Pr(MDEP>0)
HPV16/18	2020		-0.25	-1.60	-1.38	-0.96	-0.21	0.40	0.71	0.85	0.33
HPV16/18	2021		0.34	-0.47	-0.29	-0.06	0.35	0.72	0.89	1.00	0.86
HPV16/18	2022		0.95	0.52	0.61	0.73	0.95	1.17	1.28	1.35	1.00
HPV16/18	2023		1.37	0.89	0.96	1.11	1.37	1.64	1.79	1.85	1.00
Other genotypes	2020		2.28	0.19	0.52	1.21	2.33	3.31	3.73	3.95	0.99
Other genotypes	2021		1.78	0.75	0.90	1.21	1.80	2.32	2.62	2.73	1.00
Other genotypes	2022		1.83	1.20	1.29	1.46	1.83	2.20	2.38	2.47	1.00
Other genotypes	2023		3.20	2.40	2.51	2.77	3.21	3.63	3.84	3.96	1.00
HPV16/18		[30,39]	0.71	-0.16	-0.02	0.25	0.73	1.14	1.34	1.45	0.97
HPV16/18		(39,49]	0.53	-0.11	0.00	0.18	0.54	0.87	1.03	1.09	0.97
HPV16/18		(49,59]	0.34	-0.18	-0.08	0.07	0.35	0.60	0.72	0.78	0.94
HPV16/18		(59,66]	0.22	-0.34	-0.27	-0.08	0.23	0.51	0.62	0.69	0.83
Other genotypes		[30,39]	3.33	2.14	2.29	2.64	3.35	3.96	4.23	4.39	1.00
Other genotypes		(39,49]	1.99	0.98	1.16	1.48	2.01	2.47	2.70	2.82	1.00
Other genotypes		(49,59]	1.19	0.42	0.55	0.77	1.21	1.60	1.78	1.88	1.00
Other genotypes		(59,66]	0.58	-0.23	-0.12	0.13	0.57	1.03	1.26	1.35	0.95
HPV16/18	2020	[30,39]	-0.23	-2.05	-1.64	-1.15	-0.17	0.60	0.96	1.12	0.40
HPV16/18	2021	[30,39]	0.49	-0.63	-0.44	-0.06	0.51	1.02	1.24	1.40	0.87
HPV16/18	2022	[30,39]	1.22	0.59	0.67	0.86	1.23	1.55	1.71	1.82	1.00
HPV16/18	2023	[30,39]	1.72	1.02	1.14	1.35	1.72	2.11	2.26	2.38	1.00
HPV16/18	2020	(39,49]	-0.21	-1.59	-1.35	-0.93	-0.18	0.44	0.76	0.88	0.36
HPV16/18	2021	(39,49]	0.37	-0.42	-0.29	-0.07	0.38	0.79	0.97	1.06	0.87
HPV16/18	2022	(39,49]	0.98	0.51	0.59	0.72	0.99	1.23	1.35	1.42	1.00
HPV16/18	2023	(39,49]	1.41	0.87	0.95	1.13	1.42	1.69	1.83	1.90	1.00
HPV16/18	2020	(49,59]	-0.27	-1.34	-1.21	-0.84	-0.24	0.25	0.49	0.61	0.27
HPV16/18	2021	(49,59]	0.20	-0.43	-0.35	-0.14	0.21	0.52	0.67	0.75	0.79
HPV16/18	2022	(49,59]	0.69	0.27	0.34	0.46	0.69	0.90	1.01	1.06	1.00

Table S14 Marginal Difference in Expected Prevalence (in percentage points), not stratified or stratified by age, year, or age and year. Provides values from Figure 5C in table format. ***(continued)***

			Posterior distribution								
				Percentiles							
Genotype	Year	Age	Average	0.01	0.025	0.1	0.5	0.9	0.975	0.99	Pr(MDEP>0)
HPV16/18	2023	(49,59]	1.03	0.58	0.64	0.78	1.04	1.28	1.43	1.51	1.00
HPV16/18	2020	(59,66]	-0.37	-1.48	-1.27	-0.91	-0.33	0.14	0.38	0.44	0.19
HPV16/18	2021	(59,66]	0.07	-0.59	-0.51	-0.29	0.08	0.41	0.54	0.61	0.62
HPV16/18	2022	(59,66]	0.53	0.00	0.11	0.24	0.54	0.81	0.93	1.01	0.99
HPV16/18	2023	(59,66]	0.85	0.26	0.33	0.52	0.86	1.17	1.33	1.41	1.00
Other genotypes	2020	[30,39]	3.47	0.76	1.29	2.09	3.51	4.79	5.28	5.54	1.00
Other genotypes	2021	[30,39]	2.89	1.44	1.65	2.12	2.91	3.62	3.99	4.14	1.00
Other genotypes	2022	[30,39]	2.95	1.84	2.06	2.35	2.96	3.51	3.79	3.98	1.00
Other genotypes	2023	[30,39]	4.71	3.51	3.70	4.06	4.71	5.33	5.65	5.78	1.00
Other genotypes	2020	(39,49]	2.12	0.04	0.44	1.07	2.15	3.10	3.51	3.71	0.99
Other genotypes	2021	(39,49]	1.70	0.60	0.75	1.10	1.71	2.26	2.53	2.65	1.00
Other genotypes	2022	(39,49]	1.71	0.98	1.05	1.30	1.71	2.11	2.30	2.41	1.00
Other genotypes	2023	(39,49]	2.98	2.06	2.22	2.50	2.99	3.43	3.66	3.76	1.00
Other genotypes	2020	(49,59]	1.31	-0.51	-0.18	0.36	1.36	2.20	2.55	2.71	0.96
Other genotypes	2021	(49,59]	0.95	0.01	0.21	0.46	0.96	1.44	1.70	1.79	0.99
Other genotypes	2022	(49,59]	0.93	0.21	0.35	0.56	0.94	1.30	1.49	1.61	1.00
Other genotypes	2023	(49,59]	2.01	1.23	1.35	1.55	2.02	2.44	2.65	2.76	1.00
Other genotypes	2020	(59,66]	0.70	-1.12	-0.70	-0.17	0.73	1.54	1.92	2.08	0.84
Other genotypes	2021	(59,66]	0.38	-0.55	-0.40	-0.11	0.37	0.90	1.15	1.26	0.84
Other genotypes	2022	(59,66]	0.33	-0.55	-0.44	-0.17	0.34	0.81	1.03	1.12	0.80
Other genotypes	2023	(59,66]	1.28	0.26	0.46	0.75	1.29	1.81	2.08	2.23	1.00

S15 Sensitivity analyses

S15.1 Change in the variance of priors assigned to the (latent) correlation parameters

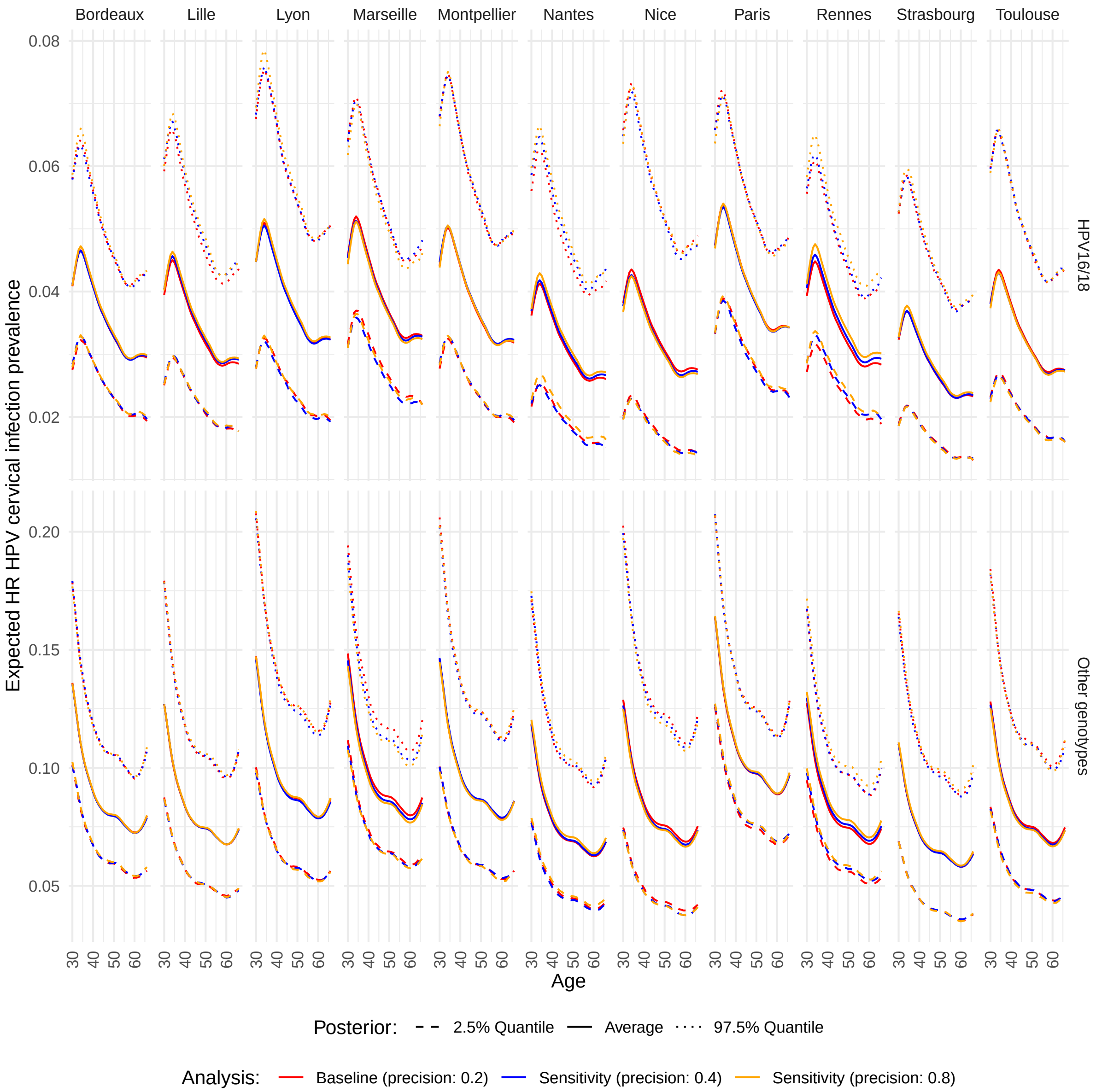

Figure S40 Posterior average [ETI95%] for the expected HR HPV cervical infection prevalence in 11 major cities in France. The precision is defined as 1/variance: the greater the precision, the lower the variance. The baseline case, precision = 0.2, has a U-shape. A precision of 0.4 looks like a uniform distribution on [-1,1]. At a precision of 0.8, extreme values for the correlation are very unlikely.

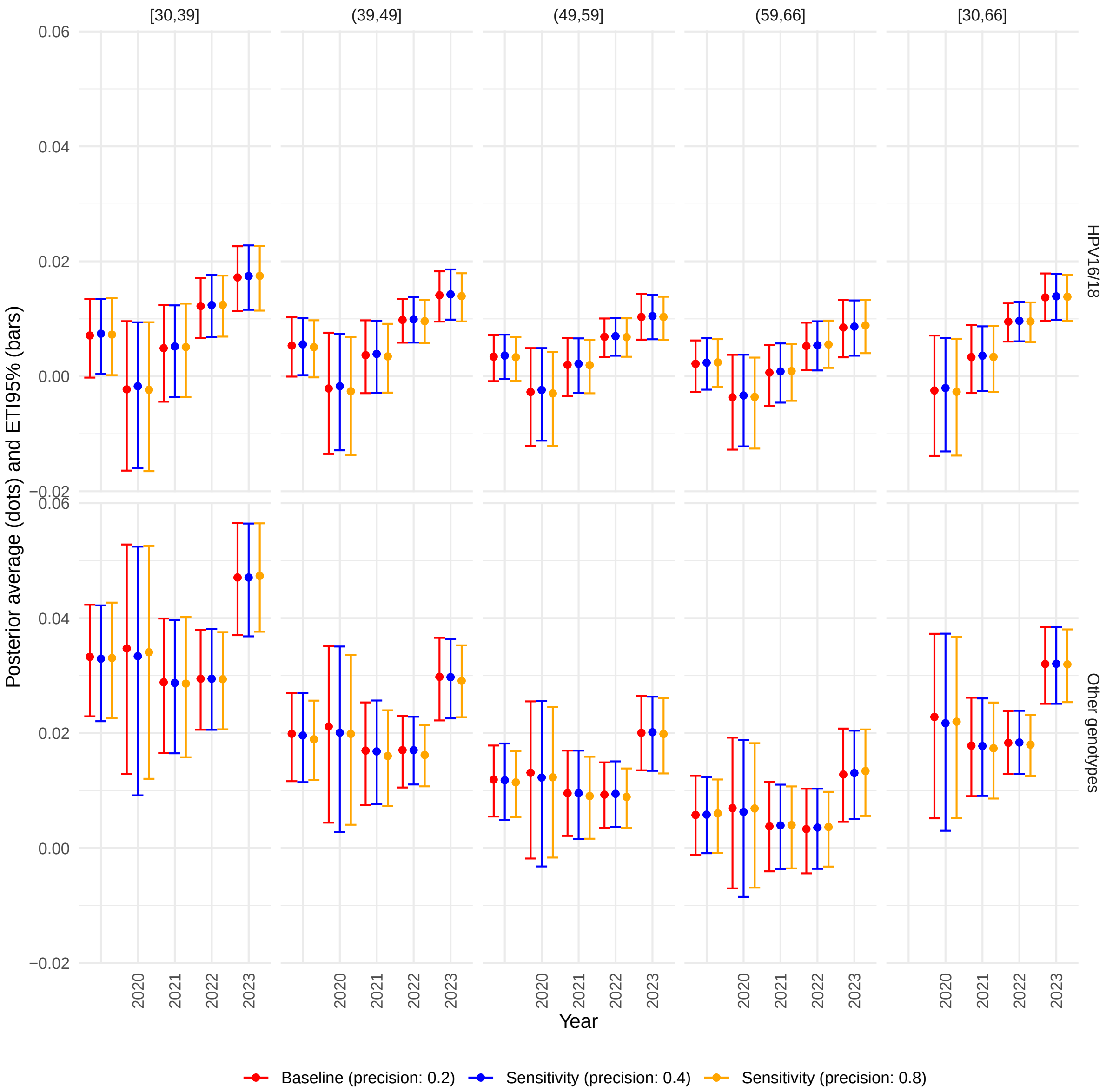

Figure S41 Posterior average [ETI95%] for the Marginal Difference in Expected Prevalence, stratified by precision for the priors of the latent correlation parameters, age, and year. The precision is defined as 1/variance: the greater the precision, the lower the variance. The baseline case, precision = 0.2, has a U-shape. A precision of 0.4 looks like a uniform distribution on [-1,1]. At a precision of 0.8, extreme values for the correlation are very unlikely.

S15.2 Non-selected competing models

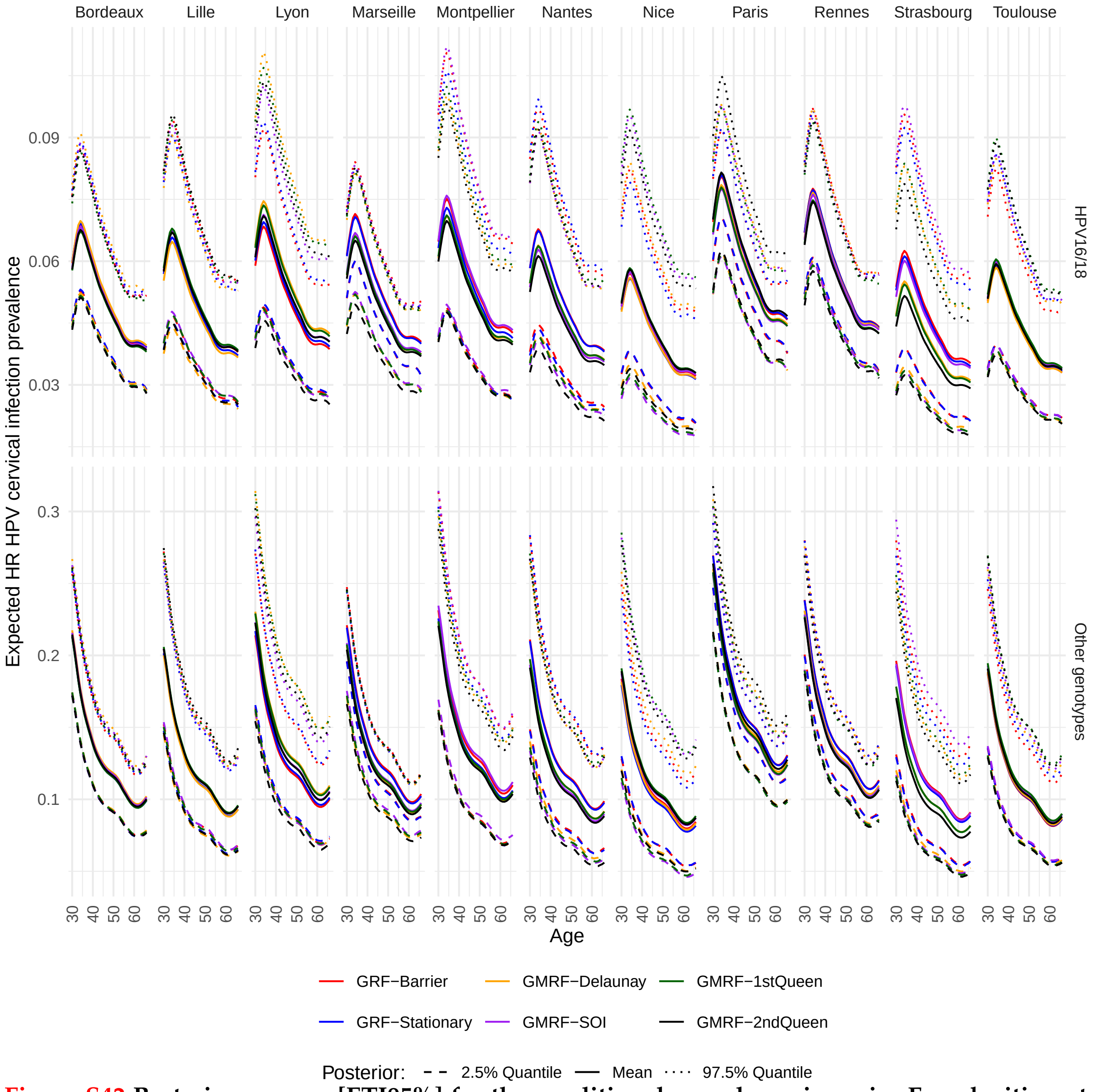

Figure S42 Posterior average [ETI95%] for the conditional prevalence in major French cities, at week 48 of 2023, stratified by genotype group and type of Matérn covariance function (stationary or non-stationary model).

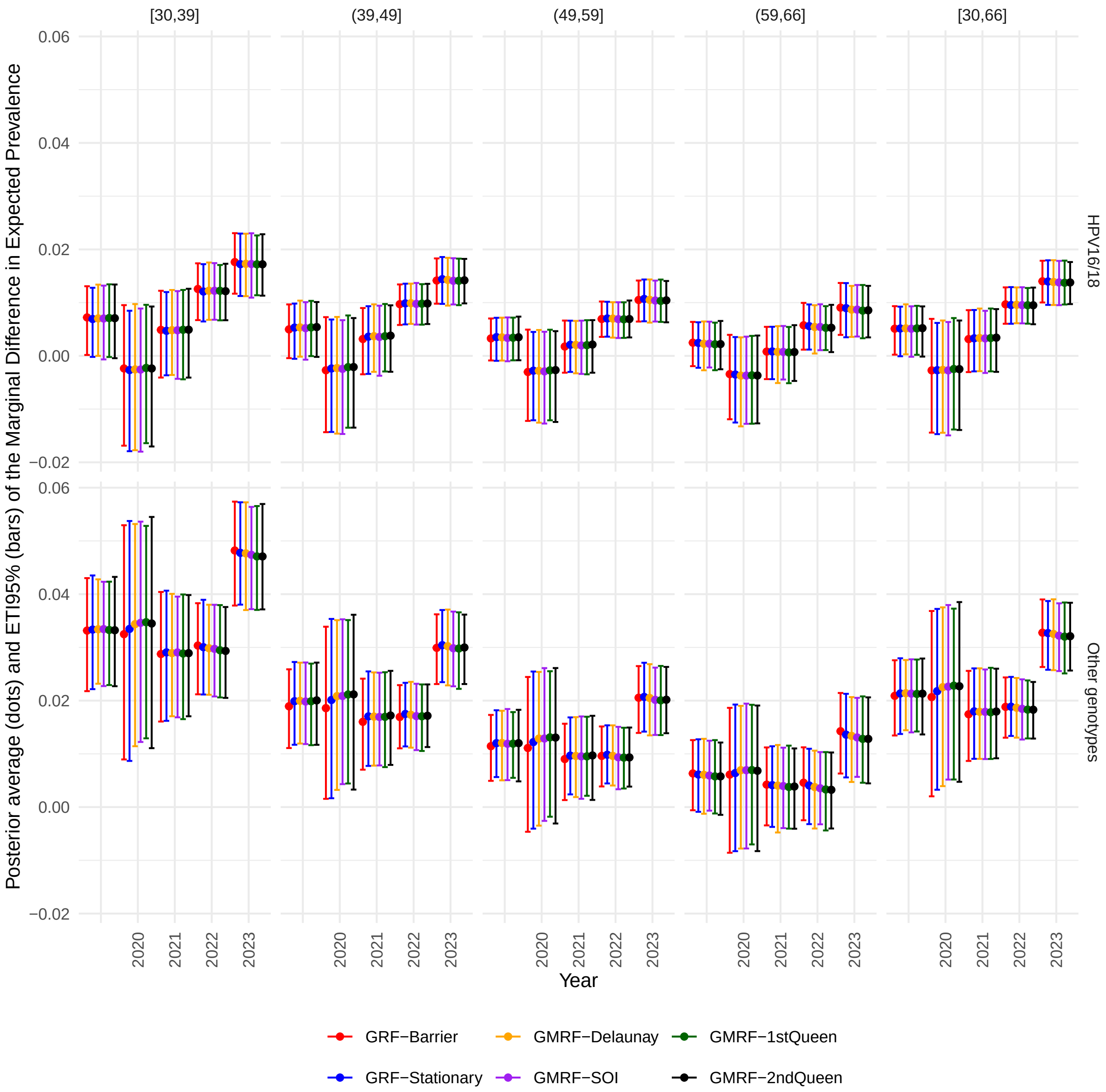

Figure S43 **Posterior average [ETI95%] for the Marginal Difference in Expected Prevalence, stratified by the type of spatial component, age, and year.**

S16 References for the Supplementary Files

1. Le Bihan-Benjamin, C., Audiger, C., Khati, I., de Bels, F., Jean Bousquet, P. & Barré, S. Cervical cancer screening pathways in France in 2015–2021, a nationwide study based on medico-administrative data. *Preventive Medicine Reports* **36,** 102429. ISSN: 2211-3355. http://dx.doi.org/10.1016/j.pmedr.2023.102429 (Dec. 2023).
2. Waheed, D.-e.-N., Olivier, C. W., Riethmuller, D., Franco, E. L., Prétet, J. L., Baay, M., Munoz, N. & Vorsters, A. Prevention and control of HPV and HPV-related cancers in France: the evolving landscape and the way forward – a meeting report. *BMC Proceedings* **17.** ISSN: 1753-6561. http://dx.doi.org/10.1186/s12919-023-00271-0 (Aug. 2023).
3. Brun, J.-L. *et al.* Conduite à tenir devant une femme ayant une cytologie cervico-utérine anormale: actualisation des recommandations INCa après la mise en place du dépistage par le test HPV. *Gynécologie Obstétrique Fertilité et Sénologie* **53,** 54–75. ISSN: 2468-7189. http://dx.doi.org/10.1016/j.gofs.2024.11.006 (Feb. 2025).
4. World Health Organization. Agents Classified by the IARC Monographs, Volumes 1–135. https://monographs.iarc.who.int/agents-classified-by-the-iarc/ (Viewed: 2024/04/07).
5. Haute Autorité de Santé. La HAS recommande de vacciner aussi les garçons contre les papillomavirus. https://www.has-sante.fr/upload/docs/application/pdf/2019-12/fiche_synthese_de_la_recommandation_vaccinale_vaccination_contre_les_papillomavirus_chez_les_garcons.pdf.
6. Santé Publique France. Cancer du col de l'utérus : la couverture du dépistage et de la vaccination doivent progresser pour une meilleure prévention. https://www.santepubliquefrance.fr/presse/2022/cancer-du-col-de-l-uterus-la-couverture-du-depistage-et-de-la-vaccination-doivent-progresser-pour-une-meilleure-prevention%20#cancers.
7. Rasmussen, C. E. & Williams, C. K. I. *Gaussian Processes for Machine Learning* (The MIT Press, 2006).
8. Lindgren, F., Rue, H. & Lindström, J. An Explicit Link between Gaussian Fields and Gaussian Markov Random Fields: The Stochastic Partial Differential Equation Approach. *Journal of the Royal Statistical Society Series B: Statistical Methodology* **73,** 423–498. ISSN: 1467-9868. http://dx.doi.org/10.1111/j.1467-9868.2011.00777.x (Aug. 2011).
9. Lindgren, F., Bolin, D. & Rue, H. The SPDE approach for Gaussian and non-Gaussian fields: 10 years and still running. *Spatial Statistics* **50,** 100599. ISSN: 2211-6753. http://dx.doi.org/10.1016/j.spasta.2022.100599 (Aug. 2022).
10. Rue, H. & Held, L. *Gaussian Markov Random Fields* ISBN: 9780203492024. http://dx.doi.org/10.1201/9780203492024 (Chapman and Hall/CRC, Feb. 2005).
11. Riebler, A., Sørbye, S. H., Simpson, D. & Rue, H. An intuitive Bayesian spatial model for disease mapping that accounts for scaling. *Statistical Methods in Medical Research* **25,** 1145–1165. https://doi.org/10.1177/0962280216660421 (Aug. 2016).
12. MacNab, Y. C. Bayesian disease mapping: Past, present, and future. *Spatial Statistics* **50,** 100593. https://doi.org/10.1016/j.spasta.2022.100593 (Aug. 2022).
13. MacNab, Y. C. Revisiting Gaussian Markov random fields and Bayesian disease mapping. *Statistical Methods in Medical Research* **32,** 207–225. ISSN: 1477-0334. http://dx.doi.org/10.1177/09622802221129040 (Nov. 2022).
14. Lang, S. & Brezger, A. Bayesian P-Splines. *Journal of Computational and Graphical Statistics* **13,** 183–212. ISSN: 10618600. http://www.jstor.org/stable/1391151 (2024) (2004).
15. Bakka, H., Vanhatalo, J., Illian, J. B., Simpson, D. & Rue, H. Non-stationary Gaussian models with physical barriers. *Spatial Statistics* **29,** 268–288. ISSN: 2211-6753. http://dx.doi.org/10.1016/j.spasta.2019.01.002 (Mar. 2019).

16. Su, P. & Scot Drysdale, R. L. A comparison of sequential Delaunay triangulation algorithms. *Computational Geometry* **7,** 361–385. ISSN: 0925-7721. http://dx.doi.org/10.1016/S0925-7721(96)00025-9 (Apr. 1997).
17. Dwyer, R. A. The expected size of the sphere-of-influence graph. *Computational Geometry* **5,** 155–164. ISSN: 0925-7721. http://dx.doi.org/10.1016/0925-7721(94)00025-Q (Oct. 1995).
18. Moraga, P. *Spatial Statistics for Data Science: Theory and Practice with R* ISBN: 9781032641522. http://dx.doi.org/10.1201/9781032641522 (Chapman and Hall/CRC, Nov. 2023).
19. Riebler, A., Held, L. & Rue, H. Estimation and extrapolation of time trends in registry data-borrowing strength from related populations. *The Annals of Applied Statistics* **6,** 304–333. ISSN: 19326157, 19417330. http://www.jstor.org/stable/41713451 (2024) (2012).
20. Dawid, A. P. Some Matrix-Variate Distribution Theory: Notational Considerations and a Bayesian Application. *Biometrika* **68,** 265–274. ISSN: 00063444. http://www.jstor.org/stable/2335827 (2024) (1981).
21. Wang, Y., Sun, Z., Song, D. & Hero, A. Kronecker-structured covariance models for multiway data. *Statistics Surveys* **16.** ISSN: 1935-7516. http://dx.doi.org/10.1214/22-SS139 (Jan. 2022).
22. Simpson, D., Rue, H., Riebler, A., Martins, T. G. & Sørbye, S. H. Penalising Model Component Complexity: A Principled, Practical Approach to Constructing Priors. *Statistical Science* **32.** https://doi.org/10.1214/16-sts576 (Feb. 2017).
23. Simpson, D., Rue, H., Riebler, A., Martins, T. G. & Sørbye, S. H. You Just Keep on Pushing My Love over the Borderline: A Rejoinder. *Statistical Science* **32.** ISSN: 0883-4237. https://doi.org/10.1214/17-sts576rej (2017).
24. Fuglstad, G.-A., Simpson, D., Lindgren, F. & Rue, H. Constructing Priors that Penalize the Complexity of Gaussian Random Fields. *Journal of the American Statistical Association* **114,** 445–452. https://doi.org/10.1080/01621459.2017.1415907 (July 2018).
25. Liu, Z. & Rue, H. *Leave-group-out cross-validation for latent Gaussian models* 2022. https://arxiv.org/abs/2210.04482.
26. Adin, A., Krainski, E. T., Lenzi, A., Liu, Z., Martínez-Minaya, J. & Rue, H. Automatic cross-validation in structured models: Is it time to leave out leave-one-out? *Spatial Statistics* **62,** 100843. ISSN: 2211-6753. http://dx.doi.org/10.1016/j.spasta.2024.100843 (Aug. 2024).
27. Gneiting, T. & Raftery, A. E. Strictly Proper Scoring Rules, Prediction, and Estimation. *Journal of the American Statistical Association* **102,** 359–378. ISSN: 1537-274X. http://dx.doi.org/10.1198/016214506000001437 (Mar. 2007).
28. Bachl, F. E., Lindgren, F., Borchers, D. L. & Illian, J. B. inlabru: an R package for Bayesian spatial modelling from ecological survey data. *Methods in Ecology and Evolution* **10** (ed Freckleton, R.) 760–766. ISSN: 2041-210X. http://dx.doi.org/10.1111/2041-210X.13168 (Mar. 2019).
29. Lindgren, F., Bachl, F., Illian, J., Suen, M. H., Rue, H. & Seaton, A. E. *inlabru: software for fitting latent Gaussian models with non-linear predictors* 2024. https://arxiv.org/abs/2407.00791.
30. Lindgren, F. & Rue, H. Bayesian Spatial Modelling with R-INLA. *Journal of Statistical Software* **63,** 1–25. https://www.jstatsoft.org/index.php/jss/article/view/v063i19 (2015).
31. Van Niekerk, J., Bakka, H., Rue, H. & Schenk, O. New Frontiers in Bayesian Modeling Using the INLA Package in R. *Journal of Statistical Software* **100** (Nov. 2021).
32. R Core Team. *R: A Language and Environment for Statistical Computing* R Foundation for Statistical Computing (Vienna, Austria, 2017). https://www.R-project.org/.
33. Bolin, D., Simas, A. B. & Xiong, Z. *Wasserstein complexity penalization priors: a new class of penalizing complexity priors* 2023. https://arxiv.org/abs/2312.04481.
34. Guttorp, P. & Gneiting, T. Studies in the History of Probability and Statistics XLIX on the Matérn Correlation Family. *Biometrika* **93,** 989–995. ISSN: 00063444. http://www.jstor.org/stable/20441340 (2024) (2006).
35. Porcu, E., Bevilacqua, M., Schaback, R. & Oates, C. J. The Matérn Model: A Journey Through Statistics, Numerical Analysis and Machine Learning. *Statistical Science* **39.** ISSN: 0883-4237. http://dx.doi.org/10.1214/24-STS923 (Aug. 2024).

36. Bivand, R. & Wong, D. W. S. Comparing implementations of global and local indicators of spatial association. *TEST* **27,** 716–748 (2018).
37. Bivand, R. R Packages for Analyzing Spatial Data: A Comparative Case Study with Areal Data. *Geographical Analysis* **54,** 488–518 (2022).
38. Bivand, R. S., Pebesma, E. & Gomez-Rubio, V. *Applied spatial data analysis with R, Second edition* https://asdar-book.org/ (Springer, NY, 2013).
39. Sørbye, S. H. & Rue, H. Scaling intrinsic Gaussian Markov random field priors in spatial modelling. *Spatial Statistics* **8,** 39–51. https://doi.org/10.1016/j.spasta.2013.06.004 (May 2014).
